# Genome-wide identification and functional prediction of salt- stress related long non-coding RNAs (lncRNAs) in chickpea (*Cicer arietinum* L.)

**DOI:** 10.1007/s12298-021-01093-0

**Published:** 2021-11-11

**Authors:** Neeraj Kumar, Chellapilla Bharadwaj, Sarika Sahu, Aalok Shiv, Abhishek Kumar Shrivastava, Sneha Priya Pappula Reddy, Khela Ram Soren, Basavannagouda Siddannagouda Patil, Madan Pal, Anjali Soni, Manish Roorkiwal, Rajeev Kumar Varshney

**Affiliations:** 1 Division of Genetics, ICAR-Indian Agricultural Research Institute, Pusa, New Delhi 110012 India; 2 ICAR-Indian Agricultural Statistics Research Institute, Pusa, New Delhi 110012 India; 3 Bioserve Biotechnology Pvt Ltd, Hyderabad, 500 076 India; 4 ICAR-Indian Institute of Pulses Research, Kanpur, 282 004 India; 5 Centre of Excellence in Genomics, ICRISAT, Hyderabad, 502324 India; 6 Present Address: ICAR-Indian Institute of Sugarcane Research, Lucknow, 226002 India

**Keywords:** Chickpea, Salt tolerance, lncRNA, Target gene

## Abstract

LncRNAs (long noncoding RNAs) are 200 bp length crucial RNA molecules, lacking coding potential and having important roles in regulating gene expression, particularly in response to abiotic stresses. In this study, we identified salt stress-induced lncRNAs in chickpea roots and predicted their intricate regulatory roles. A total of 3452 novel lncRNAs were identified to be distributed across all 08 chickpea chromosomes. On comparing salt-tolerant (ICCV 10, JG 11) and salt-sensitive cultivars (DCP 92–3, Pusa 256), 4446 differentially expressed lncRNAs were detected under various salt  treatments. We predicted 3373 lncRNAs to be regulating their target genes in cis regulating manner and 80 unique lncRNAs were observed as interacting with 136 different miRNAs, as eTMs (endogenous target mimic) targets of miRNAs and implicated them in the regulatory network of salt stress response. Functional analysis of these lncRNA revealed their association in targeting salt stress response-related genes like potassium transporter, transporter family genes, serine/threonine-protein kinase, aquaporins like TIP1-2, PIP2-5 and transcription factors like, AP2, NAC, bZIP, ERF, MYB and WRKY. Furthermore, about 614 lncRNA-SSRs (simple sequence repeats) were identified as a new generation of molecular markers with higher efficiency and specificity in chickpea. Overall, these findings will pave the understanding of comprehensive functional role of potential lncRNAs, which can help in providing insight into the molecular mechanism of salt tolerance in chickpea.

**Supplementary Information:**

The online version contains supplementary material available at 10.1007/s12298-021-01093-0.

## Introduction

Rapidly increasing soil salinity is important environmental problem leading deleterious consequences on agricultural productivity worldwide. More than 6% of global arable soil is directly affected by salinity (Yuan et al. 2016). Which accounts 20% of irrigated and 2% of dry land areas (Munns et al. 2008). Additionally, loss of about 1.5 Mha arable land along with amount worth $27.5 billion incurred annually attributing to salinity stress (FAO 2015; Qadir et al. 2014). Excess salt stress can adversely disrupt normal plant growth and metabolism by inducing osmotic, ionic, and nutrient stress (Yang et al. 2017, Cui et al. 2018).

Chickpea (*Cicer arietinum* L.) is most widely cultivated legume which is considered as important source of dietary proteins and fibers (Jukanti et al. 2012). It is grown on over 17.8 million hectares area with a production of 17.2 million metric tons worldwide and India is the leading producer with a total production of 11.4 million metric tons, approximately 66% of the total global production (FAO 2018). Earlier considered as orphan crop, chickpea is now enriched with availability of modern genomic resources which provides an opportunity to perform advanced genomic research (Varshney et al. 2013, Jain et al. 2013, Garg et al. 2011). Chickpea has been reported to employ multiple response mechanisms related to salt stress, including accumulation of osmolytes, ion exclusion and compartmentalization along with scavenging of reactive oxygen species, and other molecular mechanism mediated by salt-tolerance genes (Garg et al. 2016, Mantri et al. 2007, Soren et al. 2020).

Serval studies have revealed the role of non-coding RNAs in enhancing the salt stress tolerance in plants and modulating the gene expression in grapes, sorghum, wheat etc. (Jin et al. 2020, Sun et al. 2020, Shumayla et al. 2020). These Non-coding RNAs play vital role in gene regulation and are classified into sncRNA (small non coding RNA) and lncRNA (long non-coding RNA) based on their length. MiRNAs (microRNAs) are important class of small RNAs which are 20–24 nucleotide endogenous non-coding RNAs derived from single-stranded stem loop precursors (Seitz 2009). These miRNAs are crucial for gene functioning and their regulation under biotic and abiotic stresses, they are involved in silencing of crucial genes (Kohli et al. 2014). Whereas, in contrast, lncRNAs are longer than 200 nt, which lack coding potential and can be nuclear or cytoplasmic (Liu et al. 2012). They can be intergenic, intronic or overlapping with coding genes and can be in both sense and antisense direction. They are potential regulatory molecules which can modulate the gene expression at transcriptional, post-transcriptional, epigenetic levels to influence various biological and metabolic processes that enable plants to tolerate various abiotic and biotic stresses (Kim and Sung 2012). Recent studies have suggested that lncRNAs can influence the expression of target genes through cis- or trans-regulation and can be cleaved by miRNAs to generate siRNAs to silence the target genes (Wang et al. 2018, Fu et al. 2019, Rizvi and Dhusia 2019).

Numbers of lncRNAs have been reported in serval plant species, and their regulatory mechanisms have been at least partially revealed for their role in stress response, phosphate homeostasis and male sterility in plants (Ding et al. 2012, Wunderlich et al. 2014, Li et al. 2014). Genome-wide analysis of lncRNA revealed its significance and potential role in flower development in chickpea (Khemka et al. 2016). Salt stress-related lncRNAs have also been identified and reported in *Arabidopsis*, *Gossypium hirsutum*, *Medicago truncatula*, *Triticum aestivum*, *Glycine max*, *Pistacia vera* and *Sorghum bicolor* (Di et al. 2014, Wang et al. 2015, Shumayla et al. 2020, Deng et al. 2018, Chen et al. 2019, Jannesar et al. 2020, Sun et al. 2020). Specifically lncRNAs like, DRIR in *arabidopsis*, Mulnc1 in mulberry are reported to be associated with salt stress response (Qin et al. 2017, Gai et al. 2018). These studies have highlighted the role of lncRNAs associated with regulating complex gene regulatory network and acclimating plants toward elevated salinity. There exist a great need to escalate the lncRNA research under elevated salt stress environments in chickpea which is still lagging.

In this current study, we attempt to analyze high throughput RNA-sequencing data from 08 root tissue samples exposed to elevated salinity to identify differentially expressed lncRNAs under salt stress. Different features of lncRNAs and their comparative analysis were carried out. The target genes regulated by lncRNAs or miRNA-mediated manner were identified. Functional enrichment analysis revealed that lncRNAs function in the regulation of multiple processes. The results presented under this study may be useful for understanding and elucidating the complex regulatory framework involving lncRNAs underlying the salt tolerance in chickpea.

## Materials and methods

### Plant material and treatments

Based on our previous studies (Kumar et al. 2020, Kumar et al. 2021), we selected the salt tolerant (ICCV 10 & JG 11) and salt sensitive (DCP 92–3 & Pusa 256) chickpea lines as the experimental materials for the RNA-seq analysis. Experiment was performed at the National Phytotron Facility, ICAR-Indian Agricultural Research Institute, New Delhi, India. The four chickpea lines were cultured at 22/18 °C (± 2 °C) day/night temperature; 10/14 h light/dark photoperiod, and 45 ± 5% relative humidity described by Kumar et al. (2020) in hydroponic media. Our earlier investigation proved that 150 mM NaCl is an appropriate concentration for detecting significant differences in physiological parameters among tolerant and sensitive chickpea genotypes. Therefore, starting at the three-leaf stage, the plants were treated with Hoagland nutrient solution and salt- stress was imposed on 18^th^ day of transplanting seedling to hydroponic media with 150 mM NaCl salt solution and control was maintained without NaCl. After 72 h of stress, root tissues from stressed and control plants were harvested using sterilized scalpel blade and preserved in *RNAlater™* stabilization solution (Ambion) for RNA extraction.

### Data Sets used for the identification of lncRNAs

High throughput RNA-seq data obtained from our previous study deposited in SRA database as Bio Project ID: PRJNA579008 was utilized for identification of lncRNAs under elevated salinity (Kumar et al. 2021). These RNA seq data generated from eight different root samples at seedling stages in salt-stress and control treatments (ICCV 10; JG 11; DCP 92–3 and Pusa256). Root of all genotypes were exposed to 150 mM NaCl for salt stress and 0 mM NaCl for control. These RNA seq data were analysed for the identification of lncRNAs (Table 1). The data utilized in this study can be accessed via (NCBI) (https://www.ncbi.nlm.nih.gov/bioproject/PRJNA579008).

**Table 1:** Raw data summary of RNA- seq results

Treatment	Sample	Total reads	Total data (Gb)	Number of bases (Mb)	GC %	Total data > = Q30 (%)	Read length
Control	ICCV 10	62,487,962	6.24	6248.8	47.03	90.88	100 × 2
	DCP 92–3	66,379,710	6.63	6637.98	45.65	93.89	100 × 2
	JG 11	66,190,320	6.61	6619.04	44.86	94.2	100 × 2
	Pusa 256	66,302,916	6.63	6630.3	45.31	93.3	100 × 2
Salt- stress	ICCV 10	68,403,062	6.84	6840.3	45.31	93.2	100 × 2
	DCP 92–3	67,787,988	6.77	6778.8	45.01	93.89	100 × 2
	JG 11	63,274,182	6.32	6327.42	44.83	94.35	100 × 2
	Pusa 256	69,672,986	6.96	6967.3	47.66	92.4	100 × 2

### Genome-wide identification of lncRNA

Adaptor-polluted reads, poly-N sequence and low quality reads were filtered data via Adapter Removal (*version 2.2.0*). From these processed reads, ribosomal RNA sequences were removed by aligning the reads with the Silva database using Bowtie2 (*version 2.2.9*) (Table S1) to obtain clean reads. Trinity assembler was used to assemble the clean reads, processed via TopHat2 (*version 2.0.13*) and Cufflinks assembles programs using chickpea reference-based analysis using *Cicer arietinum* genome and gene model (http://cegsb.icrisat.org/gtbt/ICGGC/GenomeManuscript.html).

For the identification of lncRNAs, transcripts were selected that lies to the specific class (like unknown, intergenic transcript, transcription fragment falling entirely within a reference intron, genic exonic overlap with a reference transcript, exonic overlap with reference on the opposite strand) in merged.gtf file generated during RNA-Seq analysis using Cufflink packages. Cuffmerge analysis was done to obtain the transcriptome assembly to retain unique and non-overlapping sets of transcripts. For identification of lncRNAs, pipeline included different filtering criteria like, transcripts with length less than 200 bp and open reading frame (ORF) length of more than 100 amino acid were removed from transcripts. After this the obtained chickpea transcripts, were subjected to BLAST search in SwissProt and hmmscan in Pfam to eliminate transcripts having probable coding functional protein. The coding potential calculator (CPC), coding-non-coding index (CNCI) and Transdecoder (*version 5.5.0*) were then used and all transcripts with a CPC score less than 1 and a CNCI score less than 0 were considered. Finally, the filtered transcripts with one or more exons were identified as lncRNAs and were considered for downstream analysis.

### Expression estimation and differential expression analysis

LncRNAs, identified were considered for quantification of transcript expression levels and differential expression analysis from the 8 root tissues samples (4: salt stress; 4: control samples) using Cuffdiff (*version 2.2.1*) in the Cufflinks package. Expression analysis was performed based on read count using Edge R (*version 3.28.0*). The EdgeR package was used in the interest of prediction of statistically significant p-value. The statistical values obtained from the cufflink results were significant for the differential gene expression or mRNA. So, the methodology was followed as per Jain et al. (2021) for the prediction of p-values of DElncRNA. Differential expression analysis was performed using P value cut-offs < 0.05 and Log_2_ fold-change up to (+ 2/–2) separately for up- and down regulated genes. Expression values were recorded in FPKM units for each of the transcripts. The comparative expression analysis of lncRNA transcripts of various chickpea samples were analyzed and expression data were visualized using heat maps generated via hierarchical clustering explorer 3.5 (http://www.cs.umd.edu/hcil/hce/).

### Identification of SSR bearing lncRNA

SSRs (simple sequence repeats) are microsatellite markers, important for molecular characterization and gives valuable information about genetic diversity in plants (Misganaw and Abera 2017). They are hyper variable, co-dominant, and evenly distributed throughout the entire genomic regions (Oliveira et al. 2006). The *Krait tool* was used to find the frequency and distribution of SSRs (mono, di, tri, tetra, penta and hexa) on the identified putative lncRNA. The default parameters of Krait tool with respect to frequency of repeats were employed for predicting SSRs, viz., 10, 7, 5, 4, 4 and 43 for mono, di, tri, tetra, penta and hexa nucleotide repeats respectively.

### Cis-acting lncRNAs and GO analysis

*Cis-*acting lncRNA regulate the expression of nearby genes on the same chromosome in an allele specific manner. *Cis-*acting lncRNAs recruit various chromatin remodeling complexes and transcription factors to change the transcriptional status of nearby genes (Lim et al. 2018). In general, *cis* acting lncRNAs work with the neighboring coding gene. Thus, 10 kb each upstream and downstream flanking sequences of lncRNAs were extracted and search for the presence of genes (CDS) on it. The blastx program was run against the protein sequences of chickpea for the functional annotation. Gene Ontology (GO) terms were allotted and assigned to the identified target genes with AgriGO (http://bioinfo.cau.edu.cn/agriGO) and enrichment analysis was done to analyze the potential functions of the target genes (Du et al. 2010).

### Identification of lncRNA as endogenous target mimic (eTM)

The known miRNAs of chickpea from various literature (Kohli et al. 2014, Garg et al. 2019, Jain et al. 2014, Tiwari et al. 2020, Khandal et al. 2017, Srivastava et al. 2015, Hu et al. 2013, Yogindran et al. 2016, Jatan et al. 2019a, Jatan et al. 2019b) and identified lncRNA were considered for the analysis. The psRobot, a standalone tool was used for the identification of eTM by using parameters like: no bulges permitted other than at 5′ end 9th to12th position on miRNA sequences, only three nucleotide bulge in eTMs were allowed, with perfect pairing of nucleotide at 5′ end 2nd to 8th position, and the total mismatches and G/U pairs within eTM and miRNA pairing regions were set less than three except for the central bulge for eTM analysis. Putative eTMs were identified using psRobot, the miRNA target prediction software. The psRobot was executed run with optimum parameters like: penalty score threshold = 2.5, three prime boundary of essential sequence = 17, five prime boundary of essential sequence = 2, position after which with gaps permitted = 17, and maximal number of permitted gaps = 1 (Wu et al. 2012). Further, to predict the stable structure having stem loop of eTM-lncRNA, RNAfold of Vianna R package with temperature 37 °C and no lonely base pair as parameters were used on the basis of folding energy. The stability of the structure is contrary to the minimum fold energy (MFE), lower the MFE value, higher will be the stablity of structure.

### Quantitative real time RT-PCR

For DElncRNAs (differentially expressed lncRNAs) validation using qRT-PCR, five lncRNA target genes were randomly selected from the panel of salt stress responsive eTMs (Table S9). Sample was prepared after mocking similar salt treatment in roots tissues as done for RNA-seq studies in three biological replications for all the genotypes under stress and controlled environments. To validate and compare the expression of putative lncRNA affecting genes by miRNA-lncRNA interaction specific lncRNA primers were designed using Primer3Plus software (Table S2). RNA extraction from root tissues was done using RNA-isolation reagent NucleoZOL™ (TAKARA®, Cat.740406.50) and quantified in a Nano drop Spectrophotometer. A standard AccuScript High Fidelity cDNA Synthesis kit was used to convert RNA into cDNA. Normalization of the cDNA samples was done to equalize their concentration. The RT-qPCR reactions were performed using ultra-Fast SYBR Green- QPCR master mix in the Biorad CFX 96 Real-time PCR, with β-actin as a reference gene to normalize the data. The relative transcriptional levels in terms of fold-change were determined using quantification method 2^−(ΔΔCT)^ to calibrate the expression level of target gene. Analysis of variance and significance among various mean values were statistically computed using Microsoft excel and R Programing language software.

## Results

### Identification and characterization of lncRNAs

High-throughput RNA-seq data of chickpea under salt stress/control conditions have facilitated comprehensive identification of lncRNAs involved in salt stress response. A total of 530,499,126 high quality clean reads were produced from 08 root tissue samples with total data >  = Q30 more than 93.26%. The details of the raw data and pre-processed filtered data generated are given in (Table 1 & S1).

A total of 3452 transcripts were retained as putative chickpea lncRNAs (Supplementary Data 1). Distribution of lncRNAs did not show any obvious bias across chickpea genome. The maximum was 486 on Chr06 and the minimum was 128 on Chr08, for each chromosome, lncRNAs were evenly distributed (Table S3).We further investigated different features of identified lncRNAs (3452). The length of lncRNAs varied from 200 to 7506 bp with mean length 495 bp. The lesser mean length of predicted chickpea lncRNAs was similar to that reported earlier in other plants, such as rice (800 bp) and cucumber (322 bp) (Hao et al. 2015, Zhang et al. 2014). Various characteristic features, including genomic location, length, GC content etc. of all the chickpea lncRNAs have been given in Table S4. Similarity search was carried to understand the species specificity of predicted lncRNAs of chickpea in our study. BLAST analysis was performed with known lncRNAs database of 44 different plant species using two parameters: similarity > 70% and e-value < 1E-5. The result depicted that merely 60 lncRNAs out of 3373 were showing similarity with lncRNAs of other plant species (Table S10). Among them 40%, 17% and 5% were showing similarity with *Medicago truncatula*, *Glycine max*, *Arabidopsis thaliana*, etc. (Table 2). Consequently, from the results it can be concluded that most of the lncRNAs identified in this study are species specific.

**Table 2:** Number of Chickpea lncRNA showing similarity with other plant species

Plant species	Number of lncRNA
*M. truncatula*	24
*G. max*	17
*A. thaliana*	5
*A. comosus*	3
*C. clementina*	2
*P. trichocarpa*	2
*R. communis*	2
*A. lyrata*	1
*B. distachyon*	1
*G. raimondii*	1
*M. domestica*	1
*P. vulgaris*	1
Total	60

### Analysis of differentially expressed lncRNAs

**Fig. 1:**
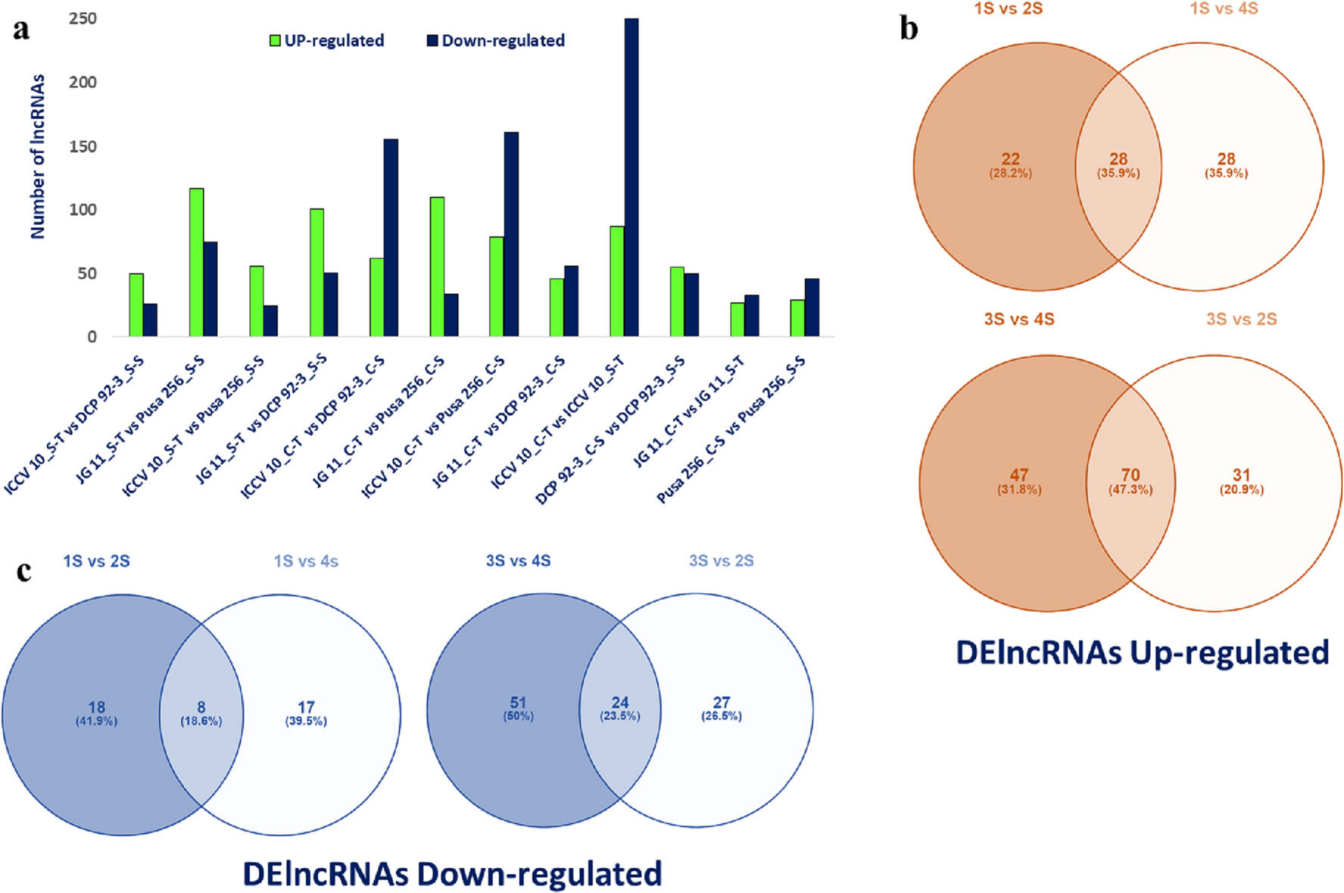
Data **a** Overview of DElncRNAs in various combinations of salt-tolerant (ICCV 10 and JG 11) and salt-sensitive (DCP 92–3 and Pusa 256) genotypes in the control and salt stress treatments; **b** common up-regulated DElncRNAs; **c** Common down regulated DElncRNAs identified by comparing each tolerant genotype with both sensitive genotypes under salt stress (1S: ICCV 10, 2S: DCP 92–3; 3S: JG; 4S: Pusa 256)

To determine the functions of chickpea lncRNAs, their expression profiles were explored in all the 08 root samples using RNA-seq data. For estimation of expression level, FPKM values (fragments per kilo base of transcripts per million) mapped reads for each lncRNA was determined using Cufflinks. The number of lncRNAs expressed in each tissue varied in different root samples compared (Fig. 1a, Table S5). Based on the expression values, lncRNAs were divided into different categories (Table S6). The expression profile showed that a large number of lncRNAs were expressed at very low level, while smaller number of lncRNAs expressed at higher expression level. The expression pattern of chickpea lncRNAs showed similarity with that of lncRNAs identified in different plants, like cucumber and rice (Zhang et al. 2014, Hao et al. 2015).

**Fig. 2:**
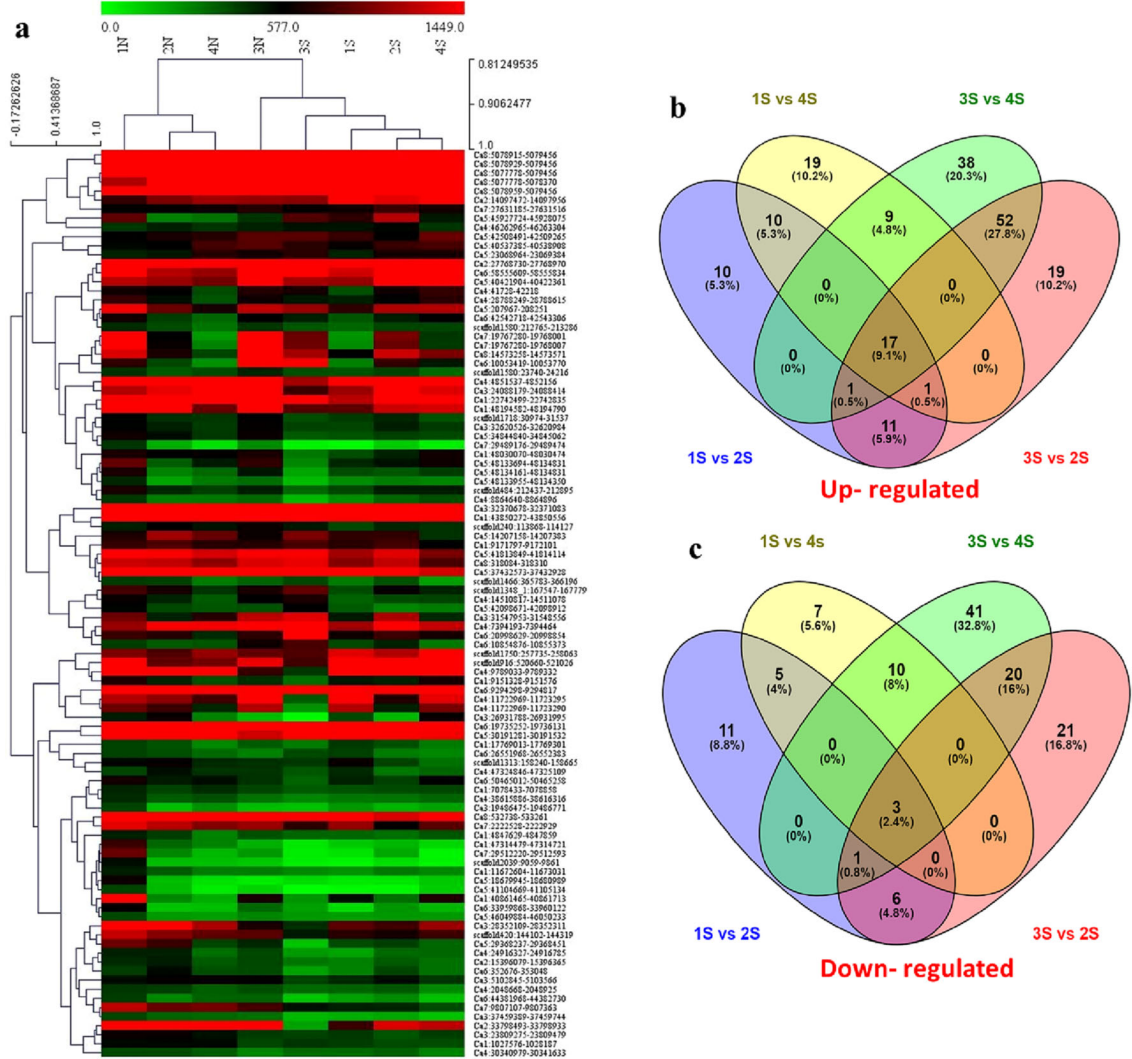
**a** Heat map and clustering of identified DElncRNAs in stress and control root tissues of tolerant (ICCV 10, JG11) and sensitive (DCP 92–3, Pusa 256) chickpea genotypes; Venn diagram comparing both tolerant genotyped with both sensitive genotypes under salt stress **b** up- regulated; **c** down- regulated (1S: ICCV 10, 2S: DCP 92–3; 3S: JG; 4S: Pusa 256)

In total, 4446 DElncRNAs were detected across all samples compared, of which 1887 were up-regulated and 2559 were down-regulated (Table S5). The comparison between salt-stressed tissues of each chickpea genotype had the following number of DElncRNAs: ICCV 10 (1S), 341 (87 up-regulated; 254 down-regulated); JG 11 (3S), 60 (27 up-regulated; 33 down-regulated); DCP 92–3 (2S), 105 (55 up-regulated; 50 down-regulated); and Pusa 256 (4S), 75 (29 up-regulated; 46 down-regulated) (Fig. 2b, c). Comparative DElncRNA analysis was performed to understand common lncRNA regulated under stress by comparing each tolerant genotype with both sensitive genotypes under salt stress (1S vs 2 s and 1S vs 4S; 3S vs 4S and 3S vs 2S) (Fig. 1b, c). We further analyzed the expression profiles of the DElncRNAs regulated under stress treatments compared with control across all genotypes. The heat map clustering generated on the basis of expression estimates, revealed that all lncRNAs showed different expression patterns in the samples for salt stress treatments and control. Based on their nature (tolerant or sensitive) and treatment, the genotypes were grouped into similar groups displaying different expression patterns for control and stress (Fig. 2a).

### qRT- PCR validation of lncRNA expression

**Fig. 3:**
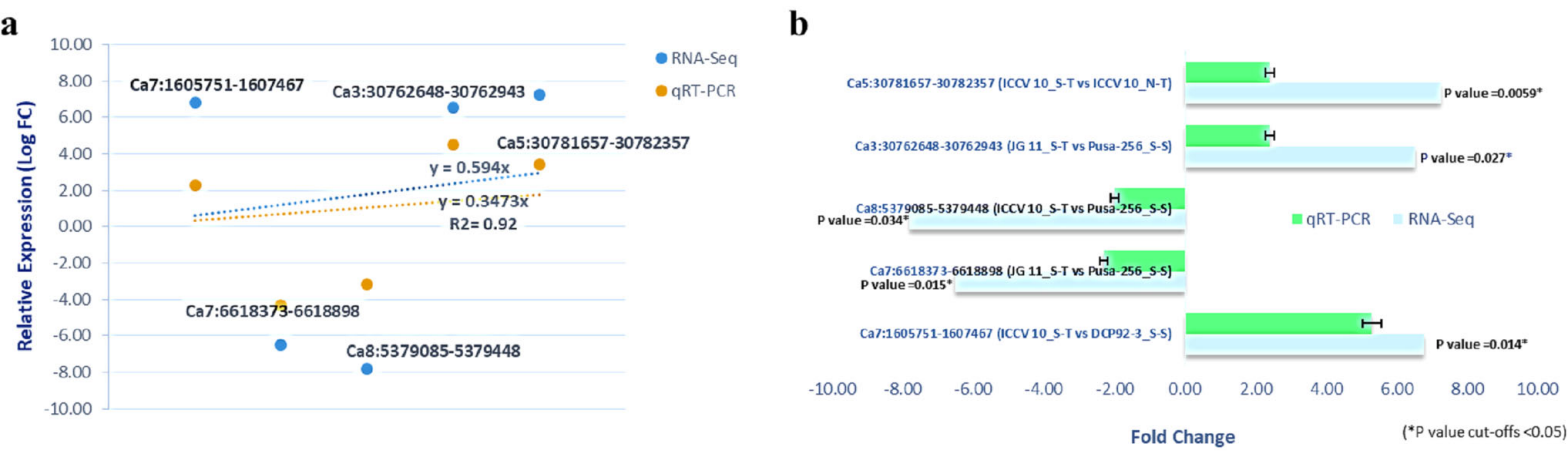
qRT-PCR validation of the lncRNA-seq data. **a** Pearson’s correlation coefficient, **b** relative fold-change expression values of 05 DElncRNAs for qRT-PCR and RNA-seq data under salt stress

We performed qRT-PCR analyses to validate the RNA-seq results from five lncRNA target genes randomly selected from the panel of salt stress responsive eTMs with lncRNAs Ids, Ca7:1,605,751–1,607,467, Ca7:6,618,373–6,618,898, Ca8:5,379,085–5,379,448, Ca3:30,762,648–30,762,943 and Ca5:30,781,657–30,782,357. We observed similar trend of expression pattern in qRT-PCR analysis as that of RNA-seq data for the selected lncRNAs (Fig. 3a). The expression results of Ca7:1,605,751–1,607,467 (myb like transcription factor) was upregulated under elevated salinity compared to tolerant genotype (ICCV 10) under both control and stress condition. Whereas, Ca3:30,762,648–30,762,943 (Histone family protein) and Ca5:30,781,657–30,782,357 (protein kinase receptor) were upregulated in tolerant genotypes (ICCV 10 and JG 11) as compared to sensitive genotype (Pusa-256 and DCP 92–3) under stress. Of these salt stress responsive lncRNAs Ids, Ca8:5,379,085–5,379,448 (ERF transcription factor), Ca7:6,618,373–6,618,898 (LRR serine/threonine-protein kinase) were down regulated in tolerant genotypes and upregulated in sensitive genotypes. Overall, qRT-PCR data, was showing a positive correlation with the deep sequencing RNA–seq data and the results of qRT-PCR analysis were in good agreement (r^2^ = 0.92) with RNA-seq data (Fig. 3b).

### Functional annotation of cis-regulating neighboring genes of the identified lncRNAs

**Fig. 4:**
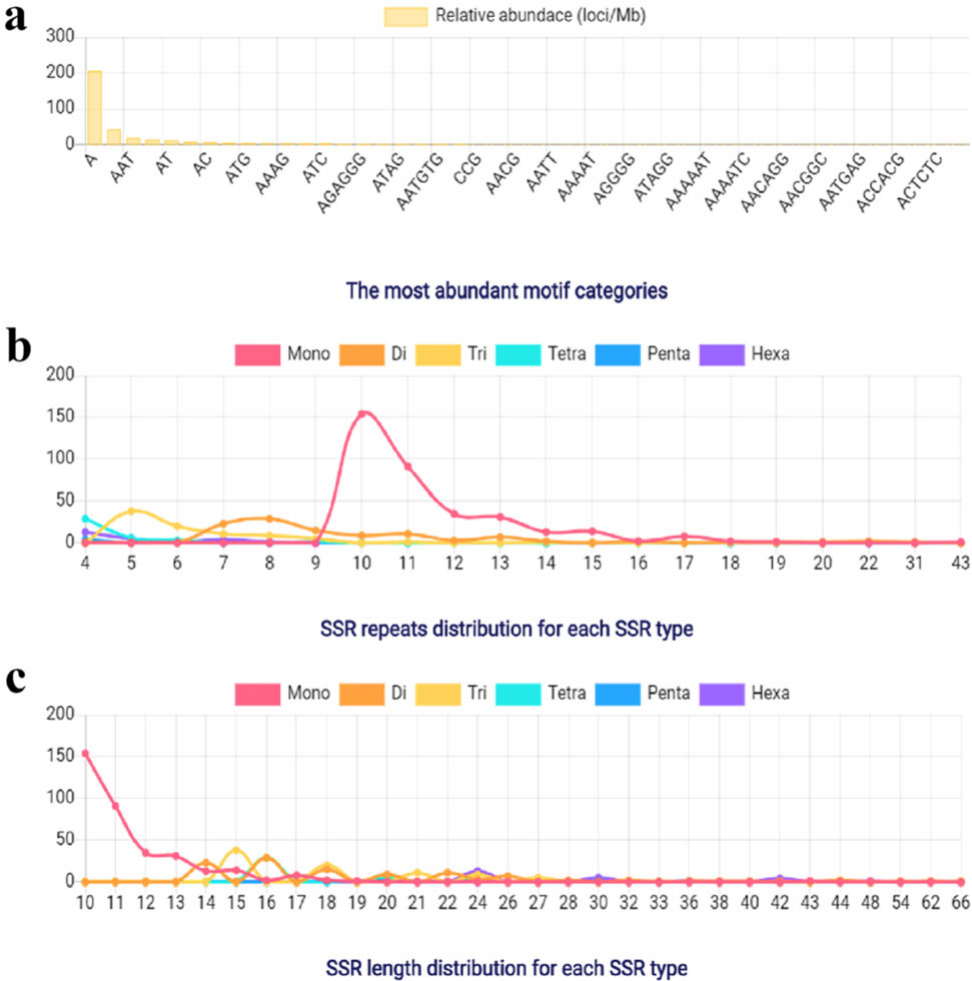
Characterization of identified lncRNA-SSRs **a** abundance in motif distribution; **b** repeat distribution in SSRs; **c** SSRs length distribution

**Fig. 5:**
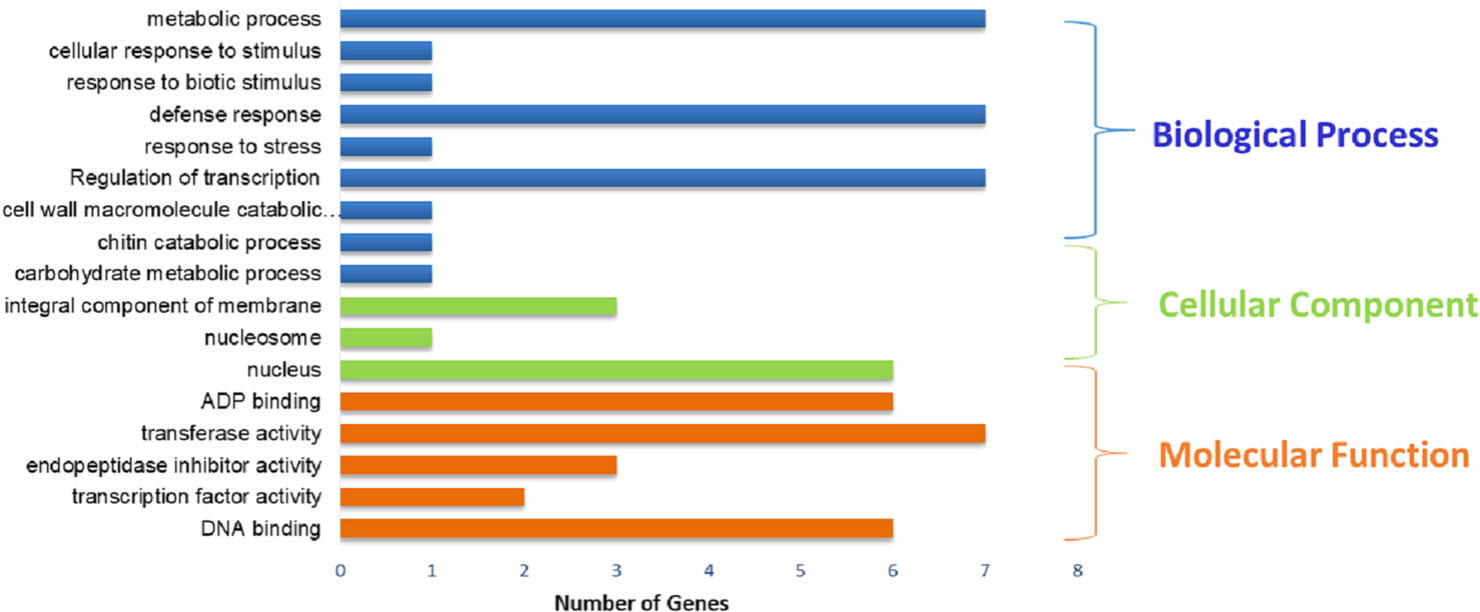
GO term assignment of differentially expressed target genes identified in chickpea

To investigate the functions of chickpea lncRNAs, we analyzed the potential targets of lncRNAs in cis-regulatory relationships. We investigated for known protein-coding genes located within 10 kb up and downstream of all the identified chickpea lncRNAs. In our study, 3373 cis-acting lncRNA were annotated and involved in variety of metabolic processes like stress tolerance, disease resistance, regulation of cell cycle, cell morphogenesis etc. Specifically, we observed many differentially expressed chickpea lncRNAs targeting salt stress response-related genes like potassium transporter, transporter family genes, serine/threonine-protein kinase, aquaporins like TIP1-2, PIP2-5 and transcription factors like, AP2, NAC, bZIP, ERF, MYB,WRKY (Table S7). The functional annotation revealed predominance of different GO (Gene Ontology) categories for the analyzed target genes, it revealed that total 22 biological processes, 08 molecular functions and 23 cell components were significantly altered in response to salt treatment. Most significantly altered biological process includes the GO term like, response to stress (GO: 0,006,950), cell wall macromolecule catabolic process (GO:0,016,998), defense response (GO:0,006,952),cellular response to stimulus (GO:0,051,716) and metabolic process (GO:0,008,152) (Fig. 4). Among molecular functions GO terms like, DNA binding (GO:0,003,677), transcription factor activity (GO:0,003,700), transferase activity (GO:0,016,758) and ADP binding (GO:0,043,531) were significantly changed. Further, for the cellular component the GO terms like nucleosome and nucleus (GO:0,000,786, GO:0,005,634) and membrane components (GO:0,046,658,GO:0,016,021) were regulated (Table S7; Fig. 5).

### Detection of lncRNAs containing SSRs

All the transcripts of lncRNAs identified in this study were used to find potential microsatellites by Krait v1.1.0, which is robust and ultrafast tool with user friendly graphic interface for the identification of microsatellites throughout the genome (Du et al. 2018). It was observed that out of 3452 putative lncRNAs, 614 lncRNAs were having SSRs (Table S8). The distribution of SSRs on lncRNAs are mono, di, tri, tetra, penta and hexa are 352, 107, 87, 39, 5 and 24 respectively (Table 3)*.* Among the microsatellites, mono-nucleotide (A) motif was most abundant (57.33%) followed by di-nucleotide motifs (AG) 12.5% were the most abundant types (57.6%) (Fig. 4).

**Table 3:** Summary information of identified lncRNA-SSRs

Type	Counts	Length (bp)	Percent (%)	Average length (bp)	Relative abundance (loci/mb)	Relative density (bp/mb)
Mono	352	4022	57.33	11.43	206.05	2354.32
Di	107	2116	17.43	19.78	62.63	1238.62
Tri	87	1707	14.17	19.62	50.93	999.21
Tetra	39	692	6.35	17.74	22.83	405.07
Penta	5	110	0.81	22.0	2.93	64.39
Hexa	24	714	3.91	29.75	14.05	417.95

### lncRNAs as candidate eTMs (endogenous target mimics)

**Fig. 6:**
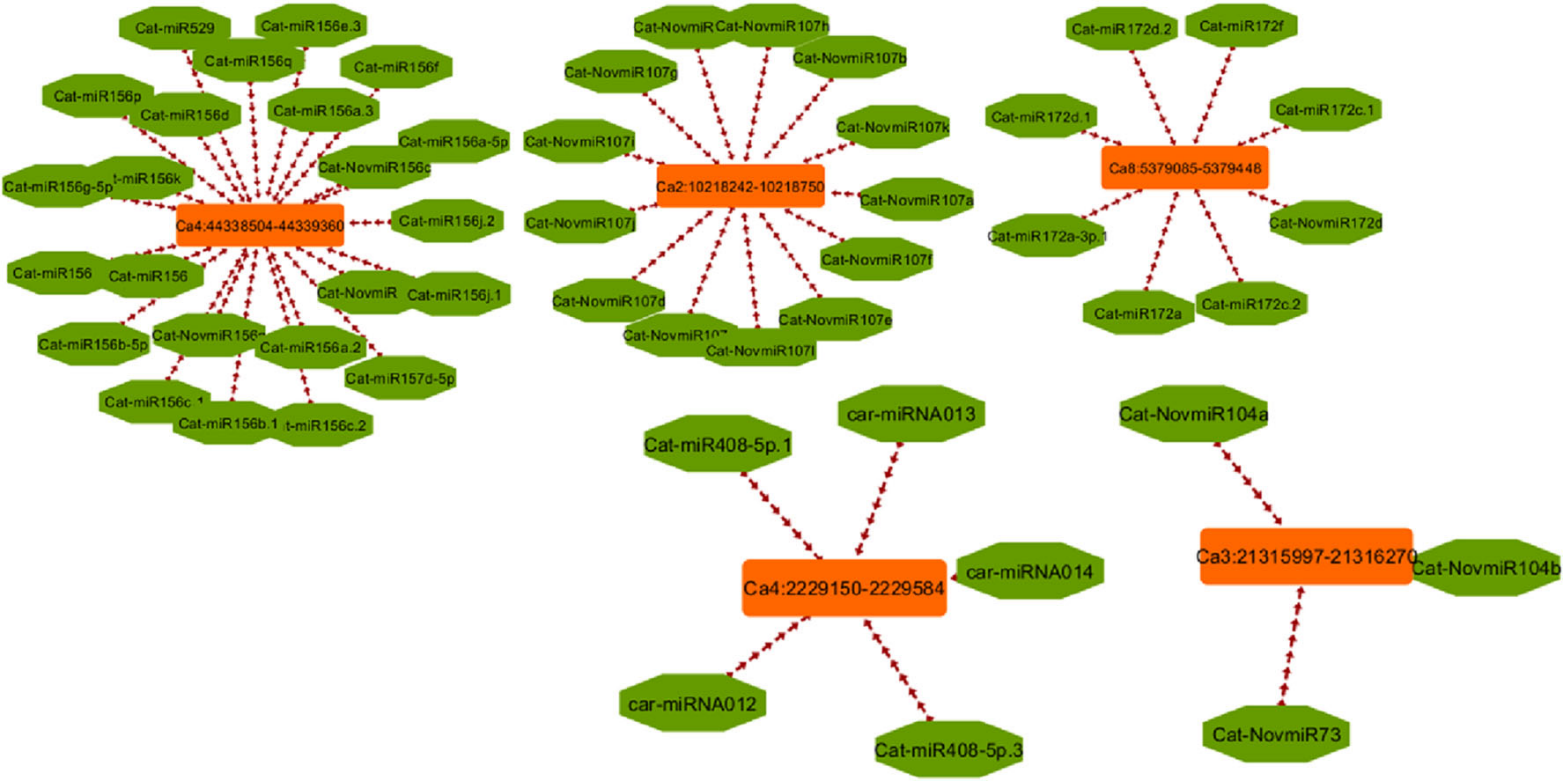
miRNA-eTM interaction network. The network shows miRNAs as green in octagon shape while eTM are shown as dark orange node in round rectangle shape. miRNA targeting genes were indicated by separate arrow the length and maroon separate arrow represents putative eTM-miRNA interactions, the length of which correspond to their respective scores

**Fig. 7:**
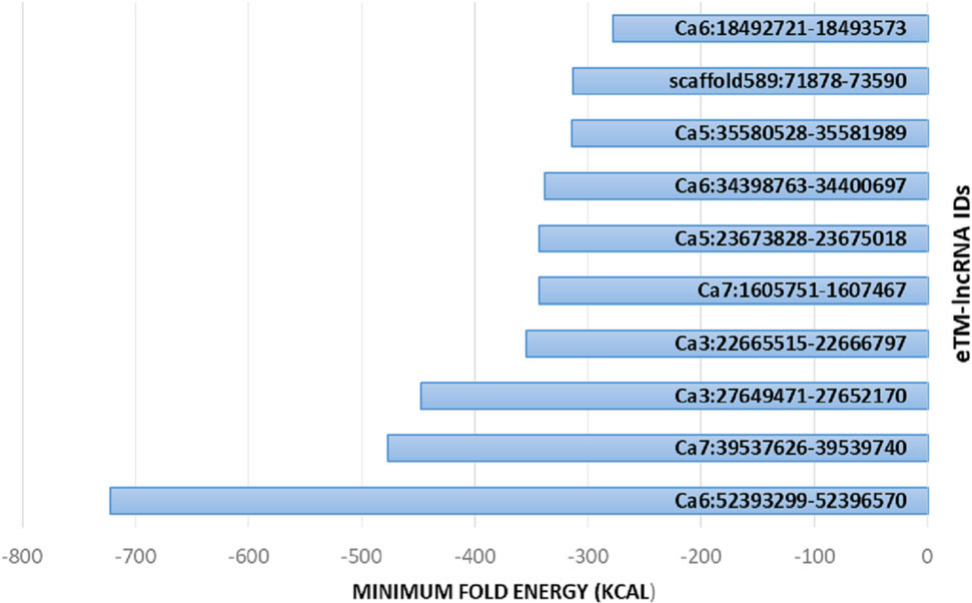
Top ten list of eTM-lncRNA with MFE (minimum fold energy) values Kcal

**Fig. 8:**
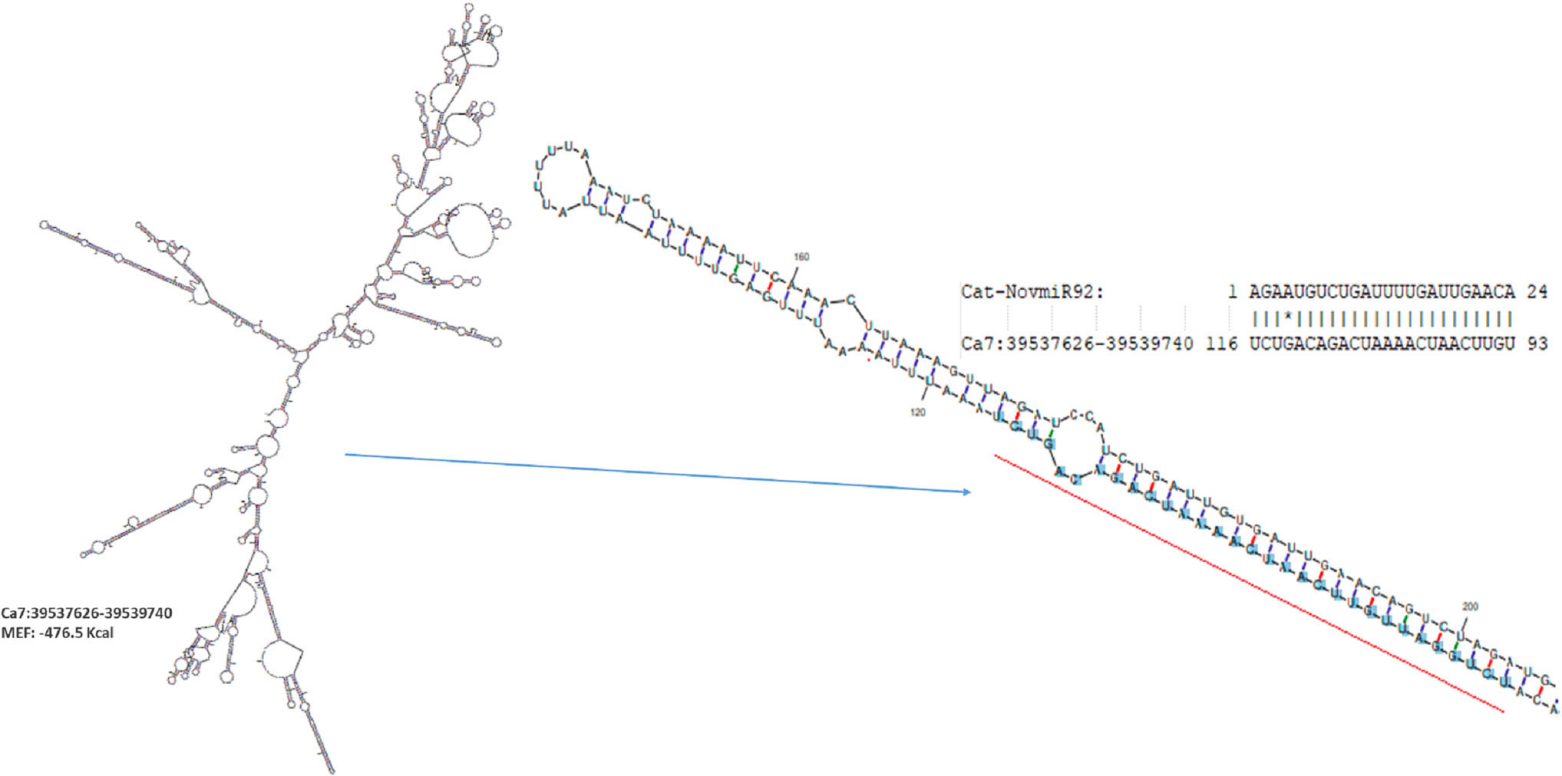
eTM – miRNA binding site prediction, representing secondary structure of eTM-lncRNA id, Ca7:39,537,626–39,539,740 with miRNA Cat-NovmiR-92 (magnified image on right shows binding position and sequence of eTM and miRNA)

lncRNAs generally acts as miRNA targets and eTMs, which plays vital roles in the competitive endogenous RNA regulatory network. The results of psRobot were analyzed and total numbers of hits were observed as 276 on the basis of score <  = 2.5. Out of hits, 80 unique lncRNAs were observed as interacting with 136 different miRNAs (Table S9). (Fig. 6). These unique lncRNAs were considered as eTM-lncRNA, were studied for their secondary structure which showed the stable structure have MFE (minimum fold energy) is less than − 15 kcal and stem loop structure (Table S11). Top ten MFE of eTM-lncRNA (Fig. 7) were analyzed for the secondary and lncRNA- miRNA binding site prediction. The representative example of eTM-lncRNA id, Ca7:39,537,626–39,539,740 with binding site of Cat-NovmiR-92 having MFE − 476.5 kcal is given (Fig. 8).

Further, target of miRNA for mRNA were analyzed using psRNA target server in which cDNA library of chickpea is already embedded. It was observed that miRNA: car-miRNA015, Cat-miR159g-3p and Cat-miR172c.2 were targeting auxin response mutant (AXR4), Myb and Ethylene-responsive AP2 transcription factors respectively under stress along with several other interaction is shown in Table S9. We observed that several target genes of miRNAs were reported to play important role in salt stress like MATE efflux family protein (Nimmy et al. 2015), Pentatricopeptide repeat-containing protein (Jiang et al. 2015, Chen et al. 2018), Peroxidase (M'barek et al. 2017, Jin et al. 2019), Ribonuclease (Zheng et al. 2014), Squamosa promoter-binding-like protein (Hou et al. 2018, Wang et al. 2019a). This shows that identified lncRNAs in this study, play important role in indirect regulation of various salt stress genes. miRNA-eTM interaction has been depicted diagrammatically using cytoscape (*version 3.8.2*) where miRNAs are shown as green node in octagon shape while eTMs are shown as dark orange node in round rectangle shape. miRNA targeting genes were indicated by separate arrow the length of which correspond to unpaired energy (UPE). Here maroon separate arrow represents putative eTM-miRNA interactions, the length of which correspond to their respective scores.

Multiple set of interaction were detected, several eTMs were predicted as precursor of numerous miRNA, (Ca4:44,338,504–44,339,360, Ca2:10,218,242–10,218,750, Ca8:5,379,085–5,379,448, Ca4:2,229,150–2,229,584 and Ca3:21,315,997–21,316,270) (Fig. 6). Further, these identified lncRNAs as eTMs regulate the gene expression and ultimately the numerous biological processes by acting as target mimic or decoy of miRNA under salt stress.

## Discussion

There are successful examples and reports regarding the involvement of lncRNA in response to abiotic stress in various crop plants like, *Gossypium hirsutum* for salt stress (Deng et al. 2018), *Brassica juncea* for drought (Bhatia et al. 2020), *Medicago truncatula* for heat and salt (Wang et al. 2015, Li et al. 2019) and *Vitis venifera* for cold and salinity (Wang et al. 2019b, Jin et al. 2020). Studies in cotton and *Medicago* under salt stress specifically suggested, lncRNAs are associated actively in plant roots as compared to leaves. There is no information till date regarding the regulatory functions of lncRNAs under salt-stress in chickpea plants. In the current study, we performed an RNA-seq analysis for four chickpea genotypes under control and NaCl (salt) treated conditions to explore the role of lncRNAs in response to elevated salinity. In order to accurately recognize lncRNAs, a stringent bioinformatic pipeline was set up which resulted in the identification of 3450 lncRNAs which were differentially regulated amongst chickpea genotypes under salt stress (Table S4). We analyzed the function of lncRNAs affecting the expression of protein-coding genes through the *cis*-regulation on neighboring genes and indirect regulation mediated by miRNAs. In light of the above regulatory patterns, a total of 3373 cis acting lncRNAs were annotated and 80 unique lncRNAs were observed as interacting with 136 different miRNAs (Table S7, Table S9).

Salt stress induces osmotic and ion imbalances leading to retarded plant growth and metabolism (Kumar et al. 2018, Liang et al. 2018). But by regulating transcriptional and post-transcriptional elements plants have capacity to adapt under salt stress conditions (Feller et al. 2011). Recent studies indicated that lncRNAs can regulate physiological metabolism as well as growth and development by contributing to the regulation of histone modifications, nucleic acid structural modifications, nucleic acid methylations and RNA interactions (Matsui and Seki, 2019; Qin and Xiong, 2019).

The dynamics of lncRNAs expression in salt tolerant and sensitive cultivars leads to identification of stress responsive lncRNAs that might play significant regulatory roles under elevated salinity. Interestingly, the results of target gene enrichment studies of cis regulating genes sequences and eTMs of identified lncRNAs indicated the involvement of serval GO terms towards abiotic stress response like, response to stress, integral component of membrane response to stimulus, regulation of transcription, transcription factor activity and response to chitin (Fig. 5) similar to earlier studies in grapes and sorghum (Jin et al. 2020, Sun et al. 2020).

Among the cis – regulating target genes (Table S7), we observed a set of transcription factors families such as AP2, NAC, bZIP, ERF, MYB and WRKY. Several reports indicated that all these transcription factors are involved in response to salinity stress, for example numerous studies have established the important roles of WRKY proteins in various physiological processes and it act as an important regulator in plant stress responses towards salinity (Erpen et al. 2018, Karanja et al. 2017, Jin et al. 2020, Sun et al. 2020, Jannesar et al. 2020). For example, WRKY, NAC, and ERF transcription factors widely participate in stress resistance (Erpen et al. 2018).

Additionally, some target genes of salt-induced grapevine lncRNAs have been demonstrated to be related to abiotic stress tolerance like, potassium transporter, ABC transporter family genes, serine/threonine-protein kinase, aquaporins like TIP1-2, PIP2-5 via cis regulating action. Along with changes in the expression of transmethylases and ubiquitin enzymes suggested the existence of lncRNA-mediated epigenetic modifications (Table S7) (Lai and Shiekhattar 2014). Further, investigation of miRNA binding/interaction with lncRNA sequences with 80 lncRNA sequences were identified as eTMs mostly annotated with stress responsive genes. The lncRNA-miRNA regulatory network suggested that lncRNAs could be combined and cleaved by various miRNAs (Table S9). Additionally, Multiple set of interaction were detected, several eTMs were predicted as precursor of numerous miRNA, which were likely to be involved in stress tolerance like, Cat-miR159g-3p, Cat-miR156j.2, Cat-NovmiR100a via competitively inhibiting the degradation of mRNAs (Jin et al. 2020 Sun et al. 2020).

These miRNAs have been demonstrated to play roles in the regulation of the primary target, influencing various abiotic and biotic response in chickpea (Kohli et al. 2014). In particular, NAC, MYB transcription factor, AP2/ERF transcription factor are reported to be regulating abiotic stress response in various crops (Jin et al. 2020, Sun et al. 2020, Jannesar et al. 2020). These TF families play active role in root development, cell cycle, primary/secondary metabolism, leaf senescence and phytohormone signaling along with regulating the expression of downstream genes via activating or repressing them during elevated stress conditions in various crops (Wang et al. 2016, Ambawat et al. 2013, Yu et al. 2012, Cao et al. 2007). The miRNA-eTM interaction (car-miRNA015, Cat-miR159g-3p and Cat-miR172c.2) were also reflecting the role of these TFs under stress. More than 16 target eTMs were annotated with SQUAMOSA promoter-binding (SBP) protein-like proteins (SPLs) in our study (Table S9). These protein family are plant-specific TFs aided with conserved DNA binding domain SBP-box consisting of two zinc finger structures (Moreno et al. 1997). This protein family has essential roles in plant development and morphogenesis (Chen et al. 2010). Recently, their role has been investigated and validated in abiotic stress tolerance in many plants (Hou et al. 2018, Wang et al. 2019a). These SPL families mainly regulate the genes involved in signal transduction, anthocyanin metabolism, reactive oxygen species (ROS) scavenging and proline synthesis under elevated salt/drought stress conditions (Wang et al. 2019b). ABC transporter family gene were also regulated under salt stress condition in our study which are reported to affect Na^+^/K^+^ homeostasis and play a role in response to salt stress (Mondal et al. 2018, Kang et al. 2011). Therefore, lncRNAs widely participate in the regulation of abiotic stress responses by altering gene expression in plants.

Serval other eTMs like, MATE efflux family protein were also playing significant role under salt stress. MATE gene family mostly encodes transporter genes involved in various physiological process in plants (Nimmy et al. 2015). Role and function of MATE efflux family under abiotic stress has been studied in various other crops like, rice (Du et al. 2021), soybean (Liu et al. 2016), tomato (Santos et al. 2017), etc. It is reported that this gene family can improve gene transcription and enhance tolerance of plants to adverse stress conditions (Peng et al. 2018, Yokosho et al. 2011). eTM targets (Cat-NovmiR105, Cat-miR171h.4) annotated with PPR (Pentatricopeptide repeat proteins) were enriched in our study have also been reported as regulated under abiotic stress. Expression of this protein family enhances, ABA regulation which induces stomatal closure and restricts stomatal opening, and plant withstand to multiple abiotic stresses including salinity and drought. It also provides high tolerance to salt stress in germination and post germination stages with no negative effect on plant growth (Jiang et al. 2015, Chen et al. 2018, Xing et al. 2018).

lncRNA regulating Peroxidases and Ribonuclease are reported to play key roles in plant physiological functions, including hormonal regulation and response to defense & mechanical wounding (M'barek et al. 2017, Zheng et al. 2014). It is reported that overexpression of *AtPRX3 and GsPRX9* enhanced plant salt tolerance (Jin et al. 2019; Llorente et al. 2002). These gene families affects the lignin and xylan accumulation in the cell wall via reactive oxygen species (ROS) signaling (Cosio et al. 2017). Thus, play prominent and important roles in antioxidant responses and salt stress tolerance in plants.

We also analyzed the lncRNAs for SSRs, Out of 3452 putative lncRNAs total 614 lncRNAs having SSRs were identified. The presence of junk DNA, duplications and repeats has led to high rate of evolution in the eukaryotic genomes (Joy et al. 2013). Even though SSRs are evenly distributed throughout the genome, the transcription sites are observed as hot spot regions for SSRs. The formation of SSR is the consequence of replication slippage during gene expression (Li et al. 2002). Given the important role of SSRs as a molecular marker for genetics and biological researches and the key regulatory role of lncRNAs maybe it’s time that to focus more on lncRNA-SSRs as a new generation of molecular markers with higher efficiency and specificity. Among the identified SSRs, the mono-nucleotide (A) motif was most abundant (57.33%) followed by di-nucleotide motifs (AG) 12.5% were the most abundant types (57.6%). The slippage does not occur in trinucleotide repeats, however it is common in mono and di nucleotide repeats (Moxon et al. 1994). Trinucleotide repeats are more varied, interesting and biased in genomic distribution (Young et al. 2000). Our findings constitute a comprehensive resource of chickpea lncRNAs and also provide a valuable salt stress responsive lncRNAs and miRNAs for future research in this direction. Due to the important regulatory role of lncRNAs, it is necessary to use lncRNA sequences to create a new generation of lncRNA related markers for crop improvement. Further in-depth research is required to explore the salt tolerance mechanism of candidate genes and lncRNAs. Despite several limitations, our study provides valuable molecular resources for understanding mechanism of salt stress tolerance in chickpea.

## Conclusion

In this study, a total of 3450 lncRNAs were identified in chickpea using RNA-seq data following stringent criteria. Out of which 3373 lncRNAs were identified to regulate their target genes in cis-regulating manner and 80 unique lncRNAs were observed as interacting with 136 different miRNAs, as eTM targets of miRNAs and implicated them in the regulatory network of salt stress response. Functional analysis showed that lncRNAs might regulate salt tolerance through regulating several transcription factors, potassium transporter, serine/threonine-protein kinase, aquaporins and methylation pathways. This preliminary study will serve as an important resource for studying lncRNAs regulating salt-stress tolerance in chickpea to have in depth insights into regulatory functions by focusing on individual lncRNAs.

## Supplementary Information

Below is the link to the electronic supplementary material.

Supplementary file1 (DOCX 523 kb)

Supplementary file2 (XLSX 1044 kb)
