# Supplementary material for: Genome-wide identification and functional prediction of salt- stress related long non-coding RNAs (lncRNAs) in chickpea (Cicer arietinum L.)
Source: Physiol Mol Biol Plants. 2021 Nov 11;27(11):2605–19. doi: 10.1007/s12298-021-01093-0 (PMC8639897; doi:10.1007/s12298-021-01093-0)
Supplement: Supplementary file 1 — Supplementary file1 (DOCX 523 kb) [file 12298_2021_1093_MOESM1_ESM.docx]

**Supplementary data 1: Identified lncRNAs and their sequence information**

>C11160518:11-683

TCCTCTATATATGCATAAATGAGTTTTCTAAAATAGCTTCAAAAAATAGCCTTGTTTCTTTATGGATCATCATATATGCATTATTAGTAGTATATTATGGTGCTAGTCATTAATTAATTACATTACAACTATGATTATCTTAATTAAGGAGTTTTATTATTATTTTTAAATATTTGTTTCACAGTTTTAACTTTCACGTCCAATAAAATGTGGGAACCCACGTGAAGAAAATATATCAAAGACTGGAATTGGAAATGAATCTATAGTACCCCCCAACACTTATGGGGAGTGATGGAGACATGTCCCATGAAAGATTTCTCTTATTTATCTATATAGATAATTTGATACTCTCAAAAACTCTTGTATTATATTCAATATGATTTATCAATTTCCAAACAGTTTTATTTATCTAATTCTCATTTTGTTGCCTTAGATTTGAAAGAAAATATTCACAATGGTCCCAATGTTGTTATTAAATGTAGCATTGCTGGCACTGCAGACACGCAAATAGTACTCTTTATTTTTCAACTGATCTTCATTGTTTGTATTTGAGTCAATAAATTAGTGAGACAAAACCTTACTCACACACATTGGCTAGAGATGTAGCTTACATTCAGCTTCTTCATTGTTAGAAATAATGATATAAATAAAGCTTATAAAGCTTGATGGTGT

>C11167104:8222-8666

CCACAATTTTTTTCCTTTGACATTGATTCATTGCAGCAACGAAGAAACTATGGAAATGCAACAATGTAACTCATCAACAGTTACACTGATAAATAGAAAATAGTAGTATTAGTAGTATTAATTAGCAAATATCATATGCACAATATTTAACTGTGGTCTTATACATGCAATGACACTTATAAAATAAACTCCAATGTCCTCAATTTCAAAACAAGCCTCCCTGAATAACCTTCCATCCAAACGCTAGAATTGCAAATATAGCTAAATTCCTATGTTTTTATATTTTATTTTTACAAGAAAAAAAAAAACATATATCAACATGTAAACACTCTAATTCTTTAAAAATATCTCCAATAAAGAACAGACCAATTTATAACCAAAAAAAATAAAAAATCAGAACCTCTTATCTCTGTCACGTCATCAGAGATAATGGTAATAAACTGC

>C11176802:11556-11996

GAGAAGCTGAAGATTCATGTGAGAAAGTGAACTGAAGAGTAAAAGAATTTGCACTGATTCAGGAAAAAATTATGCAGGAAAGCATGGAAACGGTGATGAACCCTAGCAAAACCACATTAATGGAATTACCGAATGGAACAAAGAAAAGCTTAATCCTTGATGGGTTATGTAATTGAAGAAGAACCAAGATGAAGCCCTATGAAAAAAACGAAACTTGGTTATTGGAACACTATGGAAAAACGAAAAGGGTAATGAGGTGGGTTTTTGGTTGAGCAGTTTTTGGTGTGTTTCATGTGGGAGTGGAATTCAATGTTTGGACTGAAGTTGCTGATTCAAGGATTTGATCATGGCTACGTAGTCGACAATGTGTGAAGGGTCCTTTTGTCTTTATTGTTTTGTTGTCCTTCACTACTCACTCAGTGGAGTGTAGAATTGGTGAG

>C11178328:11590-12078

TGGGTTTTGACAGCAGAGAAAAACTAAATCGGAGGAATACCCTGGCAAGTATCCTTTCTAATGTTTATAATAGGATGTGGCTTGGTTAAGTGTTCATTCAATACTAGAATAAGTTTAGGTGTTATGTGCGAATAAATCCTTAATTCTAGGTTGTTTAAATAACGGATTAATTCTTAGACGTGTAACAGGTAATCATAGACTCTAGATTGACATATCTTCCCCTACACAGCCTTTCTTTTCGATAGCATAAATGTAATACAAGGAAAATACCAGGACTAAAGTAGGTACTTGGACTCTGTCAAAGAAAATGATAATTGAGAACTTTTACTCTAAAACATGCTACAAAATTAATAACTATACTAGAAGAGTCTTGGTGTCAAATGGATATTACTAATGCAAACATGATAAATAAAGAACATTACTTGAAAGAAATACTAGGACAGAAATTCACGAATACGAACAGGCCTTTTGACTGCATTTGTAAAACC

>C11180336:17022-17464

CTGTATATGATCCATGGCTGCAAGGTTAAAACCAAAGATATCTTTCCAACCCTGACACAAGGTTCAAACAAGAAGTTTGTCAAGGAGCACGTATCATAAGAGTGGAATAACATAACTACTTAATTTAAATTGGGATAAATCACTTCCCCACTATAGAGTGAAAGGCATCAAATGTATGCATTCTTAAGCATTTCATGCATTTCAAACATGAGAATAAGAGATAACTAAAAATCATCAATAGGGTACTTTCTGTCAACCGAGTTTAGCCTGTGCAAAACCGTCCTCTGAAAAGATTTCTAGTTGCCCCAATTTTAATATAATTTTTTTAAAACAAAAGGAATTGTATACAAGTCTTCTGTACAATAGCCATATATCGCATCTTCTACAAGACAGATCAGAGAATCACAAATGGCCAAAAAAATTATCCTATCATGCACAAATT

>C11181782:13524-14313

ACGATTATTTCAAATACATAAATTCCATTACGCAAACAAACACTTCAATAAGGAAGGGAGAATAAATAAAATTCTTAAATAAAACCCTAAGCTAATTATTAACTCTAAATAGGGATGATAGGGTATTGAGGATTCACAGGTGGCATCAAGCCTCATTGCATTCGACTGCCAATCCAAATCACCGCTCAATCACATCATCGACGTTATGGCAATTCCCGTACGATTCAAAACTCTAAAAACCTGAGACGTAAGCCCGACCCACAATAATAGTAGAGGGTCACGATCTAAAAACAACATACATACAAAAATACAAAGGACAGAAGCATAACATGACATGACATCAATTGATAAACAAACATGCATATAAATATTATCATATAGCAAACATGACTCACGTGTCAAGCACCTTAATGCAATGCTTATATGTCAATGCACATGATTCCAGACTTTTCAAACCCTCATTGAGGGGAGTAAATCGTAAATGTACCGCCTCTCACAGACCAATGTTAATTACCGAGGTGCCACCTATCACAGATCAGTACGATTTACTAGAGTGCAGTTTATCACAAACCTACATTACAAATAAATCCTCATAAGGATAAGATAGACCAATCAACTCATGGAACTATCCCATGGCCCATCACGGTCACCACTAATTATGAAATGCATGAGGATACTCTAACTCTTTCACAACAATCATTATGTAAATGTAGCTATTAAAAGACTCTCCTTTTCATACTCATTACACACTAATAATTTTGCATACTTTTCACAGAGCATACTCAAAAC

>C11181782:13524-13764

ACGATTATTTCAAATACATAAATTCCATTACGCAAACAAACACTTCAATAAGGAAGGGAGAATAAATAAAATTCTTAAATAAAACCCTAAGCTAATTATTAACTCTAAATAGGGATGATAGGGTATTGAGGATTCACAGGTGGCATCAAGCCTCATTGCATTCGACTGCCAATCCAAATCACCGCTCAATCACATCATCGACGTTATGGCAATTCCCGTACGATTCAAAACTCTAAAAAC

>Ca1:34855-35141

CTTCCAAAGCTATCACATTTGCAAAGTGGCCATCCTTCTTGAAACCAAAAAATGACAAAATAAGAACAACAACATATCAAGACACACAAAGGCTAAAATAACACAACTTACACTAAGATAATATTGGACCTGCTCTTGAGTTCGACATGACATTAACTCCCGAGTTCCTGTGAAACTTGGATCGGTAGAAATGTTGTAATCTTGATAAACAACTTTTGTTTTTAAAGACTTGGCAAAACGCAATAGCTAAGAGAAAGGTTATTGACCCAGAAATAGAAAAACAGAG

>Ca1:166009-166302

AAGAGAGACGCCGTCATAGGAGTGACACGTAGGTATAAAGCCGAAGCAAGTGGTTTGTCGACGAAGCGGAAGGTTCGGACGACACAGCTGGAGACAGGCTGTCACCAACAAAGTCCCACTCACCTCTTTCTCTTTCTTCTCTTTCTTCTTTCCCTTCTTCTCTTTGTTCTTCTCTCTCTTTCTCTCTTTCTCTCTCTCTCTCTCTCTCTCACCCAATCTCTCAAATCAATTAGGGTTTCCTCGTCCCTCCCATCCCCATACCGCTTCATCACTGGATCAACATTTTCTGCACC

>Ca1:166082-166302

TTCGGACGACACAGCTGGAGACAGGCTGTCACCAACAAAGTCCCACTCACCTCTTTCTCTTTCTTCTCTTTCTTCTTTCCCTTCTTCTCTTTGTTCTTCTCTCTCTTTCTCTCTTTCTCTCTCTCTCTCTCTCTCTCACCCAATCTCTCAAATCAATTAGGGTTTCCTCGTCCCTCCCATCCCCATACCGCTTCATCACTGGATCAACATTTTCTGCACC

>Ca1:248630-249114

ATTTTTTTATCCATTCTGAAATGATTAGATTAGATCTCTTTTAACTAAACTATAGATTAGATGGCCCCAAATGAATACTATATTGGAAGATAAATCTCGGAGACAACGAGGGGCAAAGAGGATTTACATGATGATGAAGACACAGTGAGACAACATAATTAGTGTCACATTAAAGAGATCAACGTTGGGGTGGTAGGCTCATGATTGTTCTTTTGACTTAAGGAAACACAATTGATGAAAGCCAAGCACAAGAAAGACAAGGCTCTAAGGGGTTTTCATGCTTGCTTCCTTTGGACCCCTTTAGCACTTGCCTGCCTTAAAGTTTTTTCCACCAAACATATATAGCTTGTGTTAACTTCAAGGACAAAAAGAAGCATTTTTAGAAGGTAGCACTTGGACATGATCACTCTTATATGTATATATGTGCATACGTTGTGTTGGAAAATGTTGGAATAATTTGAAGCTTATCAAAATTATGATGGTT

>Ca1:487719-488226

CTACTAAGAATACGACATGAAAGAGAATTATGGTTTTCCAAGTTATAGCCAAGTGATCAGAACATTTGCTTGCATCATTGAAGTTGGGCCAAAGGATAATGTTACCAAATCTCTAACAAAGGTGGGCAGCAATGAGCAGGGGAACTTGGGAGTCGATCTCCAAGATTAGTTTCTTGATTGAAAGAATGTGTTAAGGTGAAGCCACGGTAGAGGCCTCATTGAAAAGATTCATAAACTGCTGGTCAAGCTCAAATGAAATCACCTGCATTTTATGCTCGATGATGATACCTTCATTATTTTCTCGATAGTATTATTACCATTTACTAATCCATCAATATTCGATTATTATGTTTTTGTTGTAATGGCATTTTTGGAGAGCAAGCTTCGAGGTTTAGTTAAATAAGGACTATGATATTTAAGAACAAAAGAAGTTTTTTCTCTAAATAATTTGTTAAAAAATAAATATCTGTAACATGCGGCTTTATTCAATACATATCACCACTTGTG

>Ca1:582825-583313

GATGTTGACCCTGACATGCTGACAAGGTTTCACTTCTTAATCTTTGCCCTTCCTGTTTTGTCCTGTGGTTATTGCTTGATGTCTTGGTACATTTCGCTTGGTATATGTGATTTCACCAGTTATTTTCTCTCTGCAAATATTCTCATATGATGACCAGCTTGAATCACAGGAAGGTTATTAAGATCAGCAGTCTTCTGGGCGATCGAGTTATTGTCTTTTGCTCAATATACCACACAATGAGTTAGACGCAAACATAATGTGACTCGGCGTTAGGAGGAAAATGATAGCTTAGATATAGTGAAATTTTGACAACACATTGCTGTGTTTTTGTAGTCAGTTTGTGTTCTTTGCAACATTCCTAGCTAAATTTTATGCTCGAAGTTAAAGCTTGTCCATGCAAAAGTCTGTTTAATTGTTTTAATAAAATTTGGAATCCAATGTCTGAGTCGTGATTTGTGAACATGATGGATCGAAGTGTTCTGTGCAAG

>Ca1:582463-583313

GTTTTGGTTTGGTGATCTTTTGTCATGAAAAGAGAATCAAGATGTGATTGGAGGAGATCACATGATTCAGTTATATATGTATATGCCTAACAGCAACGATTTGGATTTCAGCACATGTACCATTAAAACGTACCTTTTTATTAATTTTAATATGTTTATCATGGCATCTAAATCATCAGTAGGCTCATAAGTCATGGCACTTACAAGATCATTGCGTTTGCATCTCCCTGGTTTTTGTGAACTCACAATTCTATTTTTGGTGTTGTAATTCCAAAGGATGTTGTAAAATTTTATTTGGTTTGCTGCTTTAAAAGTATATTAATGAATTATTGATGAAAAGTTATCACATTTATTATTGGCAGGATGTTGACCCTGACATGCTGACAAGGTTTCACTTCTTAATCTTTGCCCTTCCTGTTTTGTCCTGTGGTTATTGCTTGATGTCTTGGTACATTTCGCTTGGTATATGTGATTTCACCAGTTATTTTCTCTCTGCAAATATTCTCATATGATGACCAGCTTGAATCACAGGAAGGTTATTAAGATCAGCAGTCTTCTGGGCGATCGAGTTATTGTCTTTTGCTCAATATACCACACAATGAGTTAGACGCAAACATAATGTGACTCGGCGTTAGGAGGAAAATGATAGCTTAGATATAGTGAAATTTTGACAACACATTGCTGTGTTTTTGTAGTCAGTTTGTGTTCTTTGCAACATTCCTAGCTAAATTTTATGCTCGAAGTTAAAGCTTGTCCATGCAAAAGTCTGTTTAATTGTTTTAATAAAATTTGGAATCCAATGTCTGAGTCGTGATTTGTGAACATGATGGATCGAAGTGTTCTGTGCAAG

>Ca1:654146-654478

GTAGTTCTTGGTGGCGAGCATTGCTCATTTCACAGCTTTGTCTATGAGATTTCAGATTGAAGCCTGGTTTATCATGTTGGAATTATTCCATTGCTTATGGTAATTTCTAAATTTGCTTCACTATTGAGAGGTCTTGACTGTTCTCACATCAAATTTGTGCTGCTTATTGTGTGCTTGCTGCACTAATTCATACTTTAAGCAGTCTAGCATTCAGGCATGGTAAGGTGTAAATTATGGTTCCATTAAAAAATTTAGTAGATAAGAGGTGTATATATATAATTTGTGTGCTAGTCACATGCAAAAAATTACACTGTATATATGTTAATGTGCAT

>Ca1:804600-805201

AATTGATACGTGCTGATGCGTTGCGGAATTGAGGGGAGAGGGCCATCCGTTAGATGGAGCCGCCCCTAAGGGAAAGGCCACCCTTAGGAGGAGAGGGCTATCAATGAGATAAAGCCGCCTCTTGGTTCTAAAAAATTTGTATTGTTATTTGTTTAACTTTTATAATGATTTTAAGGTTGTTCATTTTGATTTCTTTTCGTTATTCACTGGATTTTTAATATTTTTGTCAAGCGATTAAACAGTTACTCGTGTCTTTTGTGTTTCCCTTCTAATTGATGGTGGTTGTTGTCTCCTCACTAAGTCTTTGTGACTTACCCCTTTATGTTGCTTATTATTTTTCAGATTCGCAAGCAGCTGAGTAGGAATCTATTGTTTGATTCAGGTCTCAACAAACTTCTTTTTTTTTGGTAGGCCTCTTTGATTGAGGACCATTTTGTAATATTTGTTGAGTCTATGTAATTGCAGCTATTTTGGAGTCTAGAAAGTCTATTTAGTTTTTTTGAGAAGTGTAGTAAGCAATTTGATTATGTTATTTGAATTATGACTGGATTGAAAGCTGAACAGGGATTTTATAAAATTTGGGAATGTTGAAGTTACAGAG

>Ca1:850224-850473

GTTGCACCAAAGCTATTCGTCAAACTGAAGTAACGGGAGAATATTTTGGTATTATAATTTATGTTCCGTTTTATCTTCATTCCTAAATAACTACTCCTGAACATATGATTTTGTAACCAAATTCCATGCCTTGTATGGCTATTATTTTGCTGATTGGGAAATTTTGATCCCATGGAGACCTTGAATGAAAATTAATAATAACATTATGCCAATTTGAAACTTTTAGACTTGATAGGAAGTATGCTGGTA

>Ca1:864650-864901

GTTGCAACAAAGCTATTCTCCAAACTGAAGTAATAGGGGCAAATTTCATGGAATATCATTATTGCGTTCTGTTCTTGTTTCATTCCTAAATAACTGTCTCTAAACACTTGATTTTGTATCCAAGCTATATTCCCTGTACGGTTGTTATTGTGATTACTGAGAAATTTTGAGCCCATGAAGGCCTAGAAATATAATTAATAAGAACATTTATCCAAATTGTAACTCTTGTGATTTGGAAAAACGTTTTCTGG

>Ca1:1027576-1028187

GTACTCATTTAACGAGGAAACTGGCACCTACGACGAAATTCATGAGGTTGCACCCAATTCAAGTTGAGGGGCTTGTTTCAGGGGAATATGCACAATTTATAGCCATATCAACAATCATTGTCTTCCGATGGTTTATGGACAGACAGAAGCATTATTGATCGTTTTGCACATACTTGTGGCCGTGTAGGTCCTTGCTGTAGCATATCCACAATCATAGGTCCTTGCTACTGCCAGCAGTTTTTCTTCTGCCTTTATCTCTTTTAAGGTTGCTGATGATGTGTAGTATTGTTAAAGATCACCTAATTTGATCTCTTTAAGTTAGTAATAATAGATGCTTACTCTTTTGACTTATGATTCCCATAGCATGTTTATTTCTGCGTTGAAATTGCAGTCGAAACCTTGTAACCGTCGAGGCTAAAATTATGGAGCTTCATCCTGGACATGCATTGGAAACTGGAAACCATGTGGCTGCATAAACAAACGTAGCAGTAGTCAGTTTGCACTTCAAAATTGCAAAACTATTTTTGCTCGTACAATTGGATAATTGTTTTGTTGGCTTCAAAATTAGAATCATGCTAATGTGTTGCTTGCTGTTGAGTACTGGTCACTCC

>Ca1:1381423-1382248

GATCCAGTCCCCAGACGCCGGAGTTAATCTCTGAACTGGGTTAACAAGTATGTGTATTCATTTTAGCATTACAAGTTTCAAATTTTGCTTAAAACTGGTCATTCTCAATTTCAGTCAAAAAATCGAAAATGTTCTTGACATTGATCTGATCAATATCCGTTTTTTGTTTTCAATCATTATGCGTTTTTCTGGACAAAACCAAGAACGATCTTGATTTAATTATTCGTTGATCTCCGTTTTTCACAACAAGGTGTCAGTTTAATGGTAAAAATTTGACTTTTTAATTTCAAAGAATGAAGTTCAAACTCCACTAGAGACAATTGTAAAATAACATTGGTGTAACCGTTATTAATAATAATTTTTCTTCCCTTAACTTAAAAAAAAAAATCTCCAAATAAAATAAACTACCCTTTTTTGTTAGCAATATAGCATATGCATATCTTAATGACCTTTTATATGTTGATTATGGATCCATATCCTCATACATGCATTTAAGGACATATGTAATTTGTAACCATTGTTGATGGCAAATATAAATTTAGACGAGTAGTTAACGACGGTTGGTTCAGTTGGTAAAAAGTCGGTTCATACTCGCATAAAAGTGTAGTAAAGTTTAGTAAGTTTGTTTGTTATTGTGCAGTTAGTTAGCTTGTGCTCTTTTGGTAGTTGGTTTAGTTTTTTCTGTTCTATCCCTTTTGCCAACTTTGTTTATTTAAATAGCAATTGCTATGTGAATGTGAAGTAGATTTTCTTTTTCCTCAATCACAATAGAAACTCCAACTGTTAAATAAGTCTAGATGAATGCATTTACCAAAGAAACACCAT

>Ca1:1728865-1729681

TTTTAGACTCACTATAGAGTTAAAATCTACAATTTATAAATTTAATGTCTCCCTATTTATCATTGGACCAAACTATAGAGTTAATTTGTATCATATTCTGGACCCATATGAATGTCAGTATAAGCGAATTTGTCTCTACTTTGAAATTTTAATATGATTTGTATTCAAGTTTTCTAAATTCTAAAGAGTTGTTTGTACGAGTTGTTATAGCTTAGGCAAAAAATATTAAAATAGTTTAAATATTATGTAATGTAGCATTTGCATAAAATAGAGGTAGCTTTCAATATCACAGAAATTTTATGTTTTAAATCATCTTTCCTCATATTCAATTCAAATTTGTCTCCACTTTGAAATTTTAATATGGTTCGTTGAAGTTTTCTAAGTGTTATTAGGGGCAGGAATAGGCCACACCAGATCAGACTGTGAAAGACTTGACCTGACATTTTTAAAAGTTTGGTCTGAAAGCCTATTTAAAAGCCTACTTAATAATTTTTAAAAAATATGAAACAAACATAATTTAAACTCTAAAAAATTATTTTTTTGTATCAAATAATAAATTTTTTTTTCTGAAACAAACACACTAATTATTACTTCTTAAATAAATATGGAACGACCGTTGACTGATTGACTAATTGAACGACTATCGAATATGGAATGACTACCAAATATAAAATCGCTACCGAATATGAAACTACTACTGAATATGGAATGCTCAATGAGTGATTGCCTAATTAATAGAATTTAAAATAGGAAATAATATTATTAATATAATAATAATAAATAATATTAATAAATAATAAAATATATATATGCGCC

>Ca1:1817200-1817624

TTAAAAGGAGATTTTTACTTGTATATATTTAAATTTGAATTATTCAAGTAAGTATATGACAATAATCGTTTAAAAAAATCTATATAAGTAAACCATATTTTTCATGTGTTGGTCTAGATATATTTTCTACATGAATTTTTTTTTTAGAAAGCATTACTACATTATTTTCTTATTTTAAAGTTATTTAATATACACACGTTGTTGTATGGTCGATGATGGTACATATTAAATATAGCCATTTTTTGATTCCCTTATTTGGATAAGTACATTTTTTAGTTTTTGCTAATTACTAATTAACGTTTGGATAAACAATGAACAACTTCTTTTTTAATTTATTCTTGTGCATCCTTTATATGGTAGAATTCAGAGTAATCATGCACGTAAAGTCAGAGAATTACGTTATGCTTAATTATGTTGAGAGTTC

>Ca1:2045192-2045425

GTGTGCTTAAACAAAAAAGACCATGTCATAAAATTTATTGAAGAGAATTTCAAAATCAATTCTTTGGAGAGATCAATCTTAGTTTGTGGAGATTGTAACTGGAGAGACAGCACCTCAGCCCTGTTATTTGCGTGCTTCATGTTCTGTTTCCAGAAGTTATTCAAGAATCTGTGTGATAAACAAGTTTTCTTTATGATATATGTGGAAGTGGAAGTATATAAGAGTATATTAAG

>Ca1:2054573-2054992

GTGTGCTTAAACAAAAAAGACCATGTCATAAAATTTATTGAAGAGAATTTCAAAATCAATTCTTTGGAGAGATCAATCTTAGTTTGTGGAGATTGTAACTGGAGAGACAGCACCTCAGCCCTGTTATTTGCGTGCTTCATGTTCTGTTTCCAGAAGTTATTCAAGAATCTGTGTGATAAACAAGTTTTCTTTATGATATATGTGGAAGTGGAAGTATATAATAGTATATTAAGTTTTCTTAAGGTGTTGTGAACTTTACTAAATATTATTGTTATGATTTGTTTATTTTAGGATTTGTAATAGAAAATACAGATAAGGAGATGTTATTTTTTGTTTATTATGCTTTAAACTTTATATAATGCTTCAGAAAAATGTTAACTTAAATCAATGACCTTCAAAGTTCAAATAATGTTTGCTTA

>Ca1:2347649-2348235

GGATGGATGAGAATTGAAATGGGGTATGGTCCAGCAAAGCTGAACTGACAGAATGTGATGGAAGATTCTTATGGATATATGTTGGAGTGATGATATAACAATGATTCATCACAACCACTTGTTCAGAATCATTCTTTGCTTCTGTGCCACAAAGAAAGAATTCTGCTTGCTTTCTATTTATTTGTCATTTTATTTTTTTATTTTATATTAAGAACACATAGATATCCCTCCACCTCCACTTAGTTACATACGTTAATTTTTCGAAATAACTGAAATTTAAATGTTTATTTTTTAATATATATAGTTTTATAGGTACTATTAGAGATTAAACTCTTAGTTAAATAGTTAAGATATTCAAACATTTATCATGATATTCTATTATAATCGTGATATGATGTTTTCATTTTTGTTATAGTTAAATTAATATAAGTAACAGTGTAGGGTATCTTTTGTTTGCCCCACAGTGTGTTCCAAGGTTTGTTTTTGTTTTGTATTATTGTTATTGCTGAGCCATATGAACCAACAAAACAGAAGAGCACCCTGTAGTGTAGTACTACATGAATATTCTTATTCTCATTTCTCATTC

>Ca1:2394636-2395163

GTTATACAATATCAAGAGCCAAACTTTGGTACATGACATTGTCGTTGATCCAATCCATTAAGGAAGGAGAGGCTGATTTTGATTGTCTCCGATATCAGTACATCACCATGCTTCCCATAATCCCATTGTATCATTCATCTGCCACATGTTGTGAGTCTCTGTACTCTAGTAGAGGAATATCAGGCTGTGAATATCTGTATATATTGTCCAATATCTAGTGCAACCTACTTATTAGCTAATTAAGATGAACAAGAGACAGCTATGCTTCCTAGTTAGTTTTCAGTTAGGTAAAGACTTTAAGAATGCCTTCTTGCTTGGAGATTTGTGTGAGTACTATGACGATTTTCTGCTCCTAAGTTGGCCAAGTTTTGCAAGCTTCTTAAATCTCAATGGCCTCAAATAGGCGACTTGACAATGGCCCGACAAACTCACCATCTCTTGACAATGGCCCGACAAACTCCCCATCTTTATGAAAAGTGGAAAACTACTCTCTTCGTCTCACAATGAGCGTCGTTTAAAATTTGCGC

>Ca1:2394597-2395163

GTTATACAATATCAAGAGCCAAACTTTGGTACTTATCAGGTTATACAATATCAAGAGCCAAACTTTGGTACATGACATTGTCGTTGATCCAATCCATTAAGGAAGGAGAGGCTGATTTTGATTGTCTCCGATATCAGTACATCACCATGCTTCCCATAATCCCATTGTATCATTCATCTGCCACATGTTGTGAGTCTCTGTACTCTAGTAGAGGAATATCAGGCTGTGAATATCTGTATATATTGTCCAATATCTAGTGCAACCTACTTATTAGCTAATTAAGATGAACAAGAGACAGCTATGCTTCCTAGTTAGTTTTCAGTTAGGTAAAGACTTTAAGAATGCCTTCTTGCTTGGAGATTTGTGTGAGTACTATGACGATTTTCTGCTCCTAAGTTGGCCAAGTTTTGCAAGCTTCTTAAATCTCAATGGCCTCAAATAGGCGACTTGACAATGGCCCGACAAACTCACCATCTCTTGACAATGGCCCGACAAACTCCCCATCTTTATGAAAAGTGGAAAACTACTCTCTTCGTCTCACAATGAGCGTCGTTTAAAATTTGCGC

>Ca1:2481029-2481283

GAAAGAGGACATGTGAAAGGGAAGAAGGAGAGAGGAAGTGGGACTTATGATGATGAAAAGGAAGAAAAAGATGGGAAATATAGAACGGAAAGACAAGGTGTTGAGATGAACTCTTTATGCCTCTTGTTTGTTTGTATGTGTTATGTGCATGATTTTATGTGGACTTTGCAGCTCATGGTTGGTGGTGAATTTATTATAATTTTGAAACTTAACATGTGCTCTGTTCGAATGCCCTTGCCCTTGCGTTCAAGCTC

>Ca1:2885851-2886476

CAATTATTATGGTTCAGGCAAATTGCTATGGTTATGCTGCTTCACCTTAGAAGTCCCAAGTGCTGTGCTTTCAAAGATTCATCCCACAATGCCCACAACATCACTTCACCTTCAAAAAACCTTGTCCAAAACATGTCGTCTGGAAGCATTGCAGTGTCAAAATGATGTGCAGTATTTCTACATTAGTTGTTTGCTGCCTATGTTATGAATTCTGTTTTGGGGTGATAGGCTCTTATGCATGAGAATACTGAAGTAGGGAAATGATGGGTGCTGGATATAGGAACGGTTTCTATCTCTGTAAGGGCGTTCTCTCTTCATCAGAGCGTCATTATCATTTCTGCGAGTAGATCATCCCAATTCTTTTGTTTTCCTATTTTGTGTTGGCTCATAACGTGAGAGATTAATTAGTCTATTTCATTTCGATCCATTGTTATGAATTAAATTTTTGTTGTTGTTTTATTTCCCTTCATCACAACTTTCATCCCGCTGAACTATAAAGAAGGGAGTTAATGCTACCACCAAATTGGATGCAAGCTCATAAAAGCTGCGTTTTTAATCTTCTTTCTTTTCAGCTGGTGACTTAATTAAGGATTCTCATTCTATTGACTCTTGGGTTAGTGTTCTG

>Ca1:2809488-2809916

GTGTGCTTAAACAAAACAGACCATGTCATAAAGTTTATTGAAGAGAATTTCAAAATCAATTATTTGGAAGAGAACAATATTAGTTTGTGGAGATTGTAACTGGAGAGACGGCACCTCAGCCCTGTTATTTGCGTGCTTCATGTTCTGTTTCCAAGAATATGTGTGATAAAGAAGTTATATTTATGATATATGTGGAAGTGGGAGTATATAAGAGTATCTTAAGTTTTCTTAAGGTATTGGGAACTTTACTAAATATTATTGTTATGATTTGTTTAGTTTAGAATTTGCAATACAAAAAATACTGAGAGGGAGATGCTATTTTGAGTTTATTATGTTTTAAACTTTATATAATACTTCAGAAAAATGTTAACTCAAATCAATGACTTTCGAAGTTCAAATAATATTTGCTCATAGAAATGTTATCTGAC

>Ca1:2907054-2907378

GCGACTGCCAAATTGGAATGAAGTCACATAAGAGCTGGTTTTTGGTGACAGTGCCCTGAAGCCTGAAGTGAAGGGTCACCTTGATTGCAATGGATCTATGGAAGTTTAAGGCCTTTTTGTATTGAAAAAACTAAATACTATGTTTTTATTCTGTTATCACTTTTAGTTACAAGATGCTACTACTTTATATTCACTGTGTTTTTTGCTTTTTAAGTTTTGTACAGAAAATTGTAATAACAATAAAAGTTGTTTTCACTGTTTATATGACTTTATTTGAAAAGTGTATTTTTGTAAATAAAAACGAAGATAGAGAGCATATTATCG

>Ca1:2981632-2982084

CAATGAAATACTATTATTTATGTCATTATTGGAATATTTCCACATATATATTCAAAAGAATTAAAAGTAAAAACTTATAAAATATAAAATGTATTTGTGAAGTGATTGTTACAAACGTGAAGAGGAGAAATGGGCGATTGATAGGTACAAACAGGGGGAAATAACAATTTTGTTGAATGATATGAATCTTTTTTTCAATAGTTAATTGACTCCCCTCTAAGTAACTAGCTACCCTAACTATTCCTAGCTACTTTAACTCCCCTTTATTCTTTATCGTAACACAGTCTAACATGGGTATGTACAGTGTCGGATTTTGCAATTGTTTTGAAGTATATGTGGTTCCATGGTTCCTATGTCATTTGTTGTCATTACCTCTTCTGATGACATATAGCCAATTCCTGTGAATGTTCAGGGGTATCATTGATGCTCACATGACCTGATCTCGAAAAACA

>Ca1:3228943-3229312

GACACAAGATTGACTTACTTGGATCTTGGATTGGAAACTTTACGACTGCTAGAACAAGAAGTATCATCTGCACTTCATGGACTGAAAATTCATCACAGGAAAATATTGATGGATCCCAATTTGTAAAACAGAACATCACAATAATCTTTTTGTAGCAAGTTTGTTCAGAGTAAGTCTTAATTAAATATATGTACAATAATCTTGTAAGTTTATCACATATTAAACCTCATCTTCAAAGTACAAACGATAATTAAGAATGCTGCTAGCTTTACAAGTCATATTATCTTCTTTGAGATCTTAAAATATGTAATATGTTGCTGCTTAAGGCATGTTGTTTTGAAAGAGTTGTCTTTAAGACCATTCAGTGTC

>Ca1:3997848-3998472

GTGGGAACGCAACAATTGTAATTAGACAAAATACAAAATACTAATAAGTGTATTGAAGAGAGGGAAGAGGTAAAGATGAGAACACAAATAAAGTGTGTAAACGGCGCAGTGTCTTCTGAACCGTTCTGTCTATTAGTACTACGTTTACAGATAAGCCCGTTGATTGTTTTGTATTGTTGGAACCAAACAAGGAACAATCTCTTCTTAGTTCTTCCTTTCTTACTTTCATTTCATGTTTCATGTTACAGTTTTGAGACTTTGAGTTGAATCTCTACTCTCTCTCTCTATCTACCTCTCTACACGTGTATCATATCATCACCCTTCAAAACAACGGTTTCATGTTCTTCTCTTCTCTCTCCCTCACTCTATAGCAACGGTTTAATTTCATGTTCACAAATTCAGCTCAAAATATGCTTCTATGTTTTTAAATTCTGATTTTTCTTTCAAATTCATATAAATTTAAATTGAATGAATTTTTTTTGGTGGAACTGATCTTTAATGATGTATTTTTAATTATGAGCTGAATTAAATCTGCAAGTTCTGAATTTTTTGTTTTCTCTTTTTGGCTTTGTTTCATTTATGGAACTTCAGGTGCACAGATCTATGAGCTGTGAAAACAACTTG

>Ca1:4003506-4003822

GAATTCTCTTTCGTGTCCTTTCAATATGGGAGTTGCAAAATTGAAGGGTACGTTGTTGTCAAGTGTTGGTATTTCTTCTCTGACCATTTTGAGTCATCTTGTCAATTGTCTTTTGGAGAAATGTAAAAGAATATTGATTCAACTTATTATTCTTTTGATTTCTATAATTATTTTGAAGTTTATGTACTGTGGTTTCAGAAATTAAAAGACTTGTAAATCATTCAGCCAAAACGAACAAAACCTTATAATGTGTGCCTCCATAGAGGTTTTTACCCTAAAAAAGTTTGAGTATTTAGGTTTGTTGATTTAGTGAGCT

>Ca1:3997881-3998181

CAAAATACTAATAAGTGTATTGAAGAGAGGGAAGAGGTAAAGATGAGAACACAAATAAAGTGTGTAAACGGCGCAGTGTCTTCTGAACCGTTCTGTCTATTAGTACTACGTTTACAGATAAGCCCGTTGATTGTTTTGTATTGTTGGAACCAAACAAGGAACAATCTCTTCTTAGTTCTTCCTTTCTTACTTTCATTTCATGTTTCATGTTACAGTTTTGAGACTTTGAGTTGAATCTCTACTCTCTCTCTCTATCTACCTCTCTACACGTGTATCATATCATCACCCTTCAAAACAACG

>Ca1:3998237-3998472

TTCACAAATTCAGCTCAAAATATGCTTCTATGTTTTTAAATTCTGATTTTTCTTTCAAATTCATATAAATTTAAATTGAATGAATTTTTTTTGGTGGAACTGATCTTTAATGATGTATTTTTAATTATGAGCTGAATTAAATCTGCAAGTTCTGAATTTTTTGTTTTCTCTTTTTGGCTTTGTTTCATTTATGGAACTTCAGGTGCACAGATCTATGAGCTGTGAAAACAACTTG

>Ca1:4085750-4086059

GTTAGATTTGATTTTTAATCAGATTTATCCTTTTCGTGTGAAACTAGTAACCTATAAAGCGCGTGGTTTTCTTTCCATCTATCTGCTCTACTGTCCTTCATATAATAATAATAATAATCAATTCTTTCATTTTTCATTTTTTCTCTCATCNNNNNNNNNNNNNNNNNNNNNNNNNNNNNNNNNNNNNNNCTTTCCCTTTCCTCTCTAGCACTCTTTATCTCTGTTTCTTTCTTGTTCTAGAAAGCAAACTCTCTACAATCTTGAATCTCATCTCATCTCTTACTACATACACACTAGTTTACCTTTCTT

>Ca1:4847629-4847859

TAAATCAAATCAACGCAGGTATTTATTTTCCTTTAACAAAGTTGTGTTACATCAATATCAGAGTCGATAGACTTCTTCAGCTATATCTATCCATAAATCAATACAACACATATTTAACTAGGCCTATTGATTGGTTCCTACCCCCTTGGCACTCCTACGACAAGGTCTCTGGAGTGTTCATGAGGCTAAAATATGACAATTAGTAGCAGTAAAACCTTATTGACTAAGGG

>Ca1:5149037-5149319

AGTGGACATGATGCTGTGAAGACTTAGCTTGGGGTCATCAATTCAATTCTACTAAGTGCAAGAGACGACTTTGATAACCAATGTACGTGTTGGTATGCGTCCTGCATTGTTATCAAATGGTCCATGATTTGTAGGAATTTAAACTATGAAACGTATCATCAATTTATAGAGCCCAGTGGATTTTTCATCCATGTTTATTGTTTTTTTTTCTTCTTTACATGCCAATACTTGATTCGCTTAATGAAATAATAATTTAGATTTTAGAGGTTCTCTCCTCTGCCA

>Ca1:5778333-5778955

TTAATTTTGTTAAAAATAGAAGATGAAAAACTCATATAGAGGGTGGTACCACGATCATTAACAAGAAATCTAAAACCTTACCAATTTGTCAAGTGGCCAATAAATATAGAAAGGCCTCTAAATGGCAATTCCTATCTTTTTTTATCTTTTCGTTTTTTTTGGAAAATATGGAAAATGACCATTATCTTATAATTTTCTATATAAACCCACACACCCTCTAACACTTTAAAGTTTGTGTTGTGATTTTGTCTTAGAAAAAAAATGAAAATTATAGCATGTGTTGTTTTGGTGATGTGCATGGTAGTTGCAGCAGCACCTATGGCAGAAGGTGCAATCACATGTTCAAGTGTAATTGGTGCTCTTGATACATGCACTAGTTAGTAGTTATCTTCAAGGTGATTCTGGTCCTTCGGATGATTGTTGTTATGGAGTGCATGAAGTTATGGATTCAGCTGGCAGCATCATTGTCCATTGATCAGAGGAATATTTGTTACTGCTTTTCATATGGGATATTTCTATTAATGCTCCTAGTTTTGACGTACCAAGCAAATGCCGATGATCTTCTTTTCAACTGTGGTGTTACAAAGCCCTACAAGATTAGTCCCAATACCAACTGTGCTAG

>Ca1:5779298-5779597

CATTCCGTAAGATTTTACAGATGAGAATAATGGAAACTTCTCATTAGTAAATGAGAATGCCAAGTATTGCTCTATTAAGGAATAAGAGTGTTTGTACCCAAAAATATTCATAAAAATGAGAGTGATGGAGGCTCATGTTAAATAGGGCCCTATTTTCTTATTTTAATTTCAGTTTCTGAATAATCGTCACATTATCTGTTGAAAAACAAACGAATAATATGTTGTACTTTTCTATAAATAAAGCAAAAAGGTTAGGTTGTATTATTTGAAGTTAATACTCATTATTAATTTATTGTTAG

>Ca1:6051574-6051931

TCTCATCAAAATTTCGGATTTCGCTGTCTACAAAGGTTTCTTCGCGAACCCTGATTCACCAACAAAATTAACTTTTACAAATATTCCACTGCAATTTATATCTCAACATCAAGGTTTTCATGTCATTACAATTGTAATATATATGTAATTGAGGATGCATTATCTACAACCAGTTTATGCAATTCAATATCAAGAATTGTGACACAACCACAATTTTAAAACTTTGTATAGTAGTATTATGTGTTGTATAATATCAATATTATCTCTATAGGTAAATCATGTATTCATGCTGTTAGACATCGATGAATATTGAATTGAGATTGAACTATTTACATAACATGTGGATTTTGACCTTTT

>Ca1:6834844-6835101

AGAGGATGAATGAAGTTATTATGTGGGCCATGGCCCAGTAGTACTTCACACCACCCCAAAAACGCTCAAAGCCAGAAGGTGCAACCTGGTTGGCCCAATATCCGTCAACTCATAAAAAAGTTTAAATGGAATGCTAAGGAGTTTTCTGCCACCATTGTACCAACTGCTTGATTCAGCTACTTGTTATTTGGCATGGCCTCCTATCCACAACCATAATGTCACTCCTATTAGAGTAGTCTAATGAGAGTGCTGTGCAG

>Ca1:6835498-6836157

GAAAATTTGCAAAAATTGAGCTTAGGATCAAAGATAAGCTTTTGCAGGGTTGGTGGAAGCTCAGAAAAAGATTATCTTGCCTCTATTACTTCAGTACTTAGTACTAGTTTTTCTACTCTTATGCACTTTGAGTTCAAAGAATCAAAATTTACAGCTTCGATGGCTTGACACCCATAATAAATGTCTAGACACCTGAAAATGATTCTCTTGTGAGTCACGAGGCAAGAGAGTCCAGTGAAATTTGGACCTTCGAGAAGTAAAAGGGGGAAACATGAGTGTTTATGGTGAAATTTTATTCCGAGCTAATCCCGACCCGTAGGCCCTCAGACCTGGAAGTGAGTTTTCTCCTCTGTATTTTGTGTATCTACGCGCCCCAAAATCAATGCTTGGGCACCCAGAGAATTCTTTAAATGTCATTTAGTGTGAAAAGTAGTTGCACAACACACTTGTTACAATTAGCAATTTTGCCGTTATTTTTTCTGCTGTATTCAGTTAGTAGTTGCCTTCAGTCAACCATGAACCATAGTTATCCCTTTCAGTTACTCCATGTAATTCTGTTAGGCTATTTCAGCTGATATAAATATCAGAATGTGAGATTTTGTAATGCTCAGTTGAAATAATCCAATTTATCAATCAATTTTGGCGCTGCCATATCCAAT

>Ca1:6834844-6835102

AGAGGATGAATGAAGTTATTATGTGGGCCATGGCCCAGTAGTACTTCACACCACCCCAAAAACGCTCAAAGCCAGAAGGTGCAACCTGGTTGGCCCAATATCCGTCAACTCATAAAAAAGTTTAAATGGAATGCTAAGGAGTTTTCTGCCACCATTGTACCAACTGCTTGATTCAGCTACTTGTTATTTGGCATGGCCTCCTATCCACAACCATAATGTCACTCCTATTAGAGTAGTCTAATGAGAGTGCTGTGCAGG

>Ca1:6834844-6835103

AGAGGATGAATGAAGTTATTATGTGGGCCATGGCCCAGTAGTACTTCACACCACCCCAAAAACGCTCAAAGCCAGAAGGTGCAACCTGGTTGGCCCAATATCCGTCAACTCATAAAAAAGTTTAAATGGAATGCTAAGGAGTTTTCTGCCACCATTGTACCAACTGCTTGATTCAGCTACTTGTTATTTGGCATGGCCTCCTATCCACAACCATAATGTCACTCCTATTAGAGTAGTCTAATGAGAGTGCTGTGCAGGT

>Ca1:6918086-6918543

TAAAAATATAATTTGATAATTAAAATATAAAATGCACATTCCGACTTTTTGTCTCTCTTCTCTGATAAAAATAGACTCTCATGTACTTGATAGAATGCAATACAACCCTCACGAAATGGCATATTTGGTGGGAGTGTGGGACCCTGATTCACCGAAAGTAAAGATAACTTGGGCCACAAGCCCAAATATATCTGGACCACCAGAAACATCAACTAGCCCATGAAGTAGAACAATCGCCAATTGGGCCATTTAGAAGACACGCGCCGCGGATATTAATAAGACACTGTCACGCTCCACCACATACTTTTTTCCTTTACATTTCTCATTTTCTCTCTCTCTCTCTCTCTCTCTGAAATCGAACCCTCGATCGAAAACGAACACACCTTATTTCTTCTCCTATATCTTCTCCACCACCATCGCTCCTAATCAAATCTTCTACTACCACAACTAACACATG

>Ca1:6918239-6918543

GAAAGTAAAGATAACTTGGGCCACAAGCCCAAATATATCTGGACCACCAGAAACATCAACTAGCCCATGAAGTAGAACAATCGCCAATTGGGCCATTTAGAAGACACGCGCCGCGGATATTAATAAGACACTGTCACGCTCCACCACATACTTTTTTCCTTTACATTTCTCATTTTCTCTCTCTCTCTCTCTCTCTCTGAAATCGAACCCTCGATCGAAAACGAACACACCTTATTTCTTCTCCTATATCTTCTCCACCACCATCGCTCCTAATCAAATCTTCTACTACCACAACTAACACATG

>Ca1:6992215-6992464

TGTAGCTACCCACTTCAAATGCACTAAAATTTGCACTAAATAAAGTGGTCCTCTTTCACTTCATGCTAGCTCACAAGATCCAATGTAGATAGATCCCTCTCTGCTCTTTTTCCCCTAAATAAATAGTAGGATGCTATTTTATGCCATTTATTATATCTTAAAGCACGGGTGGGTTTTTACTTTTTCTATGTGAATTTTCCTTTTCCCCACTTAAATAGTACTAGAGACTCAACCATTCAAAACAATGCC

>Ca1:6992608-6993368

AGATAATGTGTTAGATTTATATTTTAATTTTTTTAATAAATTTAATTTTGATTTTGCAAATATTCCTATCTCTATATGTGGTTCACAACTTTGACCACTTTTTGAATCACAACACAAGATCTTCCAAATTGGTTGGCACATGAATGGAACCTTAATTTGAAAAAGGAAAATAAAAGATAAAAAAAGTAAAACAATAGTAAACAATTGTTGATGATCAGTGTCAATTGATGTTTCTCCAAACTTCAAAGCAAAATTTACATTATTTCTAGAAAAAAGTGTTTTTGGAAACTATGAGGAAAATTTGAATATTTTATTAGCCAATTGAAAAAAATTAATAATATGAGTAGTGTTCTTTCCACGTTAATGATGAAATGGACATTCAAAATAAAGTACATCAATAATCTAGACACACTTTGACATTTAGTGGCATGTCTATGTATATGTTTTCAATTCCACATGTAACAAGCAACTGCAACCAAACTTTGTCTTTGCAATTAGGTTTTGGAGCTGTCACTATAAACGACAAGATTACTTTAGCTACCAAAGACATCTCACACCCATATTCACATTTCAAGCCCACACTTTGATGCATTTATAGAAATTTTCTATGGTAATTTAATAATTGTACTTATCTTTAGATTTCAATGAGGACAATGTAGGCTTGCATTGGAGTAATCAACACAATAACGTATATATGTAGCATCCATGAGATAGCACAATAATAATAATAATAATAAAATCTTGTGTTTTTTTTTAAGGG

>Ca1:7315636-7316166

CTGCATTGGTCCTTCTCTATCTATTTCCTGTTTGATGTTTTTTTTCCTTTGCTCATCATCTGCGTTTTTTTCTATACCTCTGATGCATTTATGATTTTGATTTATTTGCTTTGATATGTTCTGTTGCTGAGCTTTAATTTCTCCCTTAGTTTATTTTTTAAACTCAAAATTTTGTCTCTTTGTTCTGCACAAAGTGTACCAAGACTGATTTAAAAAAAATGTTTTTATGTTTCTCTAATTAGTTACAGAGCAAAGGTCAATGACAACTACATCTGAAACAGAAAGGCAGCAACAAAATAGAGCCAAACACTGCATCAGAAACAGAGAGGCAGAGAGGCAACAACAGCAACAAATTAAAAATTATTGTAATTTAATTTAGTTTGGTTTAAATCTTATTGTAATTTAATTTTGCCTTTATTTTAGTTTGTAATTTGATTTGGTTTTAACATTCTAAATAGATTTGAAATTGTAAACTTATAATACATAATGAAATTATTTATTGTAATTATTTTATTTCCTTTTTTAAATAC

>Ca1:7508261-7508503

TATGGGACCAGCTAAGCATTGAATTCCCGTAATCTTATAGAAGATTGGAACTTTGGACATTCCAAAAGCCCCAAGTTTTTTTCGTGGAGAGAAATTTTGTTGTGTTTATCGTTTTCTCCATTGAATTCATTATTACTAGTGTATGAAATTTATGACTAATAGTCACAACTGTATCTGGACCAAAATATGTTAGTCTTGTTTAAAATATTGCATTCAGAATTACTGATTTAGGCTATCACAAG

>Ca1:7678534-7678854

CCAAAACCGTGATTGCTCATGCAGCAAAACCACGCCCAAGTGCCAGACTGTTAACGACCGGATTCCAACGCGAAAACAGATTATTGTTTAGATTCTGCGTAAACGGACGGTCAAGATCGTCTGCCGCATGCCTAAAAACAGTGCTCACTCAACTCAACACGCAAACACTACCCCCAATCCCTTTCCATACCTCAACGTGGAGAACGGAACAGCGCCGTAACTTAACTCAAACGGCGCTGTTTTCTTCGTTCCGAGTCAGACGCACGCTTTTCTTTATTAGTGATTTTGATATTACCTGCATTGACGTCTTCCAGTGTTCG

>Ca1:7678563-7678854

CCACGCCCAAGTGCCAGACTGTTAACGACCGGATTCCAACGCGAAAACAGATTATTGTTTAGATTCTGCGTAAACGGACGGTCAAGATCGTCTGCCGCATGCCTAAAAACAGTGCTCACTCAACTCAACACGCAAACACTACCCCCAATCCCTTTCCATACCTCAACGTGGAGAACGGAACAGCGCCGTAACTTAACTCAAACGGCGCTGTTTTCTTCGTTCCGAGTCAGACGCACGCTTTTCTTTATTAGTGATTTTGATATTACCTGCATTGACGTCTTCCAGTGTTCG

>Ca1:8372626-8372832

CCGTGGACCAAATTAAACCTTCCTCTCTCCTCAGAAATCCTTAAACTAAAACTTGATCCAATTCCCCCACACATTCTCTTTTTCACCTTTCTTGCGTTTTTTTTTTTGTTTAACGCAAAACACTTTCTTCAATTTCAAAAACCTATTTCCCCTTTCTCATTACTCCATCAATTTCAGTTTTATCGCACACCCCCAATTTTTTTCCG

>Ca1:8723855-8724218

CAAACGGCAGATGCTTTACTTGAGTCAGGTTATTCTCTGCTCAAAGAACAACAGTTGATAATGGAAACTAAACTTTCAAAGGAATACATGAAGAACATAGAAGACTAATATAAAGGAGGTTGGTCCTCTAGTGCAAATCCTCAGGAATTATAATTGTGAAGTTGCCACCTTCGATGAGGTCCACTTTCGAACAGGCTGCATCTCTTTATCGTTTTTTCACTTTTTATGCGTTATTTTTCCGGATATAATCCATTTAAGACGAATAAATGAGTGAAATATTATCCAACAATAGTCTAATGATATCAGGAATGAAATCTTATGTTGTGTGCTGAATTGTCCAAAAAAATAACTCAATAAATTCTG

>Ca1:8839304-8839873

GGGAGCCACATACGTCCAGTTGTTCCGCGGCCCAATAGAGCAATTTAGTCAAATTTCCAGATACTTTTCCGCGGTTTGTGTTTCCTTCTTATCTTAATGCTTGCTTGGTCAGAGACACCACAACACCATACTCATTAATATCTTTCTTTATTAATTAACCTTATTCTTAGTCAAAATTACAACCCTAATATTAGCGCAAAGCCGGTAATTGGCAGAAACCCTATTCCCCCTTTCTAAGAAACAAAATTGATATAAATCTCCCCATTCCCTTTCATCACTCTTCACCCCTCAAACAAACAACCCTTCCTCCTTTCCTGCATGCACCATGAAGATCTTTAACCCCTCCAAATCCTTCCTCCAAAATGTCGTGTGTCCCTTTGACACCATCTTCATTAAACACACCAAATTCCTCTCCCTTGGTGCTTCTCACGTTAAACAGTTTCATTTCCTAAGCTGCAACTGAAACCATCTTTCATTCATTACACCCCCCTTCCAATCTCCCCTCCCCTCCCCTCCCCCCCTTTTCAATCCAAAACATGGATCACCACCGTCACAACAAAAAAAGAAAC

>Ca1:9151328-9151576

GTTGTGTTGTGTCATTGAAGATGCAATCCACTTACAGCACACAGACATCACAATGGACTCAGCAGTTTCACTACACAATAGTTAAATACTTACATTGATGAAAAAACTACCAATAAAAGTCAACAGCAATCTCCTTTAAATATCTCAAGCTTATTAGCATTTTTGAAAAGAAAAACAGTTTCACAGGACCATTTCCCCTCCATTGCTCACTCTCACACCATAAGTAATATATTTAGTGAACAAAACAT

>Ca1:9171797-9172101

GGATATTTGGAAAATACAGAAAAAGAAAGAATCAAGAGACTGATAAGGTTGAAGCCTCCTACCGAGTTCTGAATGAGAAATCCTGACAGTCATTAGAATCAGCAGTTTGAGGTGTGTTGAGCATCTTGTGAGTTATAATGGCAAGACACCGAGGTCAGGCAAACCTCTGAATATGATAATTTTTTCTCTAGTATGGTGCATGTGAGGATTTTGCTCGGCCAATAAATATTTGTATAACATTCTCAAATTATACATCTTTTATGTTGTAACATCTATATTGTTGATTTTATTCTACCTTTACCTT

>Ca1:9236768-9237165

AAATAATTGGACACAAAAACTTTAGTATTTCTCTTCAAAAAATTCTAAAATCTTTCATCACTGCGCCCTTCCAATTGTCCACCAAAATCCATTTTCAATCTAAATCCTCCTTGCACAAGCTCTCAACAAACTCTACTCACACAACAATCTTCGTGTAACGCTCCTTGCGCAAACTCTTTCATTCTCACGCAAAACAATCTTCATTGTCTTCTCTCTCAAGAACCCTCTCGACATCGCTCGACCTGAATGAGAAAGGGGAAAAACATGATTAGGGTTTCTGTGTCGAGTACAGTTTTCTCGTGATTTTCCCCCTTTTCTCGCAACTCTACATCGATTAGGGTTTTGGCATCTTGCGAGACTTGCAAAAGTTTTACCTCTTCGTGACTTGGTGAGCTTG

>Ca1:9236781-9237502

CAAAAACTTTAGTATTTCTCTTCAAAAAATTCTAAAATCTTTCATCACTGCGCCCTTCCAATTGTCCACCAAAATCCATTTTCAATCTAAATCCTCCTTGCACAAGCTCTCAACAAACTCTACTCACACAACAATCTTCGTGTAACGCTCCTTGCGCAAACTCTTTCATTCTCACGCAAAACAATCTTCATTGTCTTCTCTCTCAAGAACCCTCTCGACATCGCTCGACCTGAATGAGAAAGGGGAAAAACATGATTAGGGTTTCTGTGTCGAGTACAGTTTTCTCGTGATTTTCCCCCTTTTCTCGCAACTCTACATCGATTAGGGTTTTGGCATCTTGCGAGACTTGCAAAAGTTTTACCTCTTCGTGACTTGGTGAGCTTGGTATGTATTTTCATAGTTGCTCTATTATCGCTCAACCTTTGCATCAATTACGTTTGCATTACTCTAAAAATAACTCTATAATCATCTTCAAATGATTGATTTTGATATCGCTCTTTATTCTATTTCCTATTTGCTGTTTTTTAATCTATGACATCTTGTGGATTCTAAAATATGAGAAATTCATCTCTCTAATCTTAGAAACACTTTAAAAACTTGAAGCATTAAGGATGGGTATATGGTTATTTCTGTTGATTGTTCATATAACTTGAGATATTGGTTGTTTATTTTAATCTCTCTGCACTATGTTAATTAGTGGGAAGAACCATCATAGTTAGAG

>Ca1:9657285-9657535

ATTCTTTATATAGAGTAGTACTGTCCTACTGTTGAGTATAGATTCCTTTTTCACTCAGTAAGCAAAAAAAAAAGTAGATCTGAAACCCATCTTTCTATCAAGAACCCCCAGCTCCATTTCCACGCCCCATCTCCGGCCTCCGCGACACATATATCCATTTTCGTGCTCTTCATCTCCTAAGCTTTCATTTGAACCGAATAAATCAACTTTTGAAGCAACTTCGTGGTCAACCCATTTTCTTCCCTTCCCG

>Ca1:9659326-9659599

AGTTGCAGCTGCAGGAGTGCAATAATATTTCAAAATCATCAAGAAACAACATAAATGATAATGGGTGCATGACATGATGTCCAATTTTTGTAGCTCATGAGGGAGTTGCAAATTGTTGTACTGTTTTTGATTTATTATTTAGGTTTATGGTCTTTAGGAAGATACTACAAGTGTTATGAATCAATATCATATGTATGATTCAAAAGCAAAACAAGTTGATACCATAATATCATTTTTAAGGATTAAACACAAATAAAACAAACTTTAATTGAA

>Ca1:9836317-9836622

CTCCAAGAGCCATGGAAAAATCAAGGAGCCAAAACTAGTGCAGAAAACCTCGTTGGACCAAAGTGGAGTATTTGCTAATGCATATATAAAGGTTTACAAAGAAGTTTGAGTTTATGGTTATATTCCAAATAAAATATATATATGTAACTCAGTGTCATAGTTTTTGTGCATTTATAGTCTAACAAGTTTTCAATCAGTAGAAATTTAACTGGAGAGAATTCGTTTACATAGTAATTCGTTTGCATCTCTGTTTTGTGTACCTTTGGATTCTTGTTGCTATATGAAAAGAATTGCTTGTTGTGCTT

>Ca1:10191517-10192124

ATTTTGTTGTGCAGTTAATAATAGGCCTATATGTCCTGCTTGCTTGTGTTGCACTCCACCACCCCCTGGCTCTTGCTGCACAAAGTGTTGTGCTTCGGTACCAGTTTCATAATAGGGAAATCAAATGTCCATTTACATATAACATCACATATAGTTATTAATGATATGTTTGTAATTTTATATGTTTAACAAATCATTATCATGATGTTATCATATATTGTATGTTACCATCATCAAGGACTGAATGCCATGTATGATATTGGATCTTAAAAGTTATAAATCAGTTAACAGGACATTAAATTATTTACCTATTAAAGTATGTCTCATTCTTATCTTTCACTCACCTAAAACATTATATTAACTTGAACATCATAGTGTTCTTTGACACTATGTGAATGAAGGTTTTTCGGTCGGAATCTTACTCGAAGAGGACCTATCTCTAAAGCTCAAGTTAATATAATGTTTGAGGTGAGTGAGAGGTAAAAATAGATTTCCCTTAATAGGTGATAGAGTGAGACTTATATAGCTATAATAGAGCGGTATTGGACTTGGGCCATCTTTTGGCTACTCCCAGACAAATGGTTTCTATTGTTTAAGTTTTGAAGAG

>Ca1:10191531-10192124

TTAATAATAGGCCTATATGTCCTGCTTGCTTGTGTTGCACTCCACCACCCCCTGGCTCTTGCTGCACAAAGTGTTGTGCTTCGGTACCAGTTTCATAATAGGGAAATCAAATGTCCATTTACATATAACATCACATATAGTTATTAATGATATGTTTGTAATTTTATATGTTTAACAAATCATTATCATGATGTTATCATATATTGTATGTTACCATCATCAAGGACTGAATGCCATGTATGATATTGGATCTTAAAAGTTATAAATCAGTTAACAGGACATTAAATTATTTACCTATTAAAGTATGTCTCATTCTTATCTTTCACTCACCTAAAACATTATATTAACTTGAACATCATAGTGTTCTTTGACACTATGTGAATGAAGGTTTTTCGGTCGGAATCTTACTCGAAGAGGACCTATCTCTAAAGCTCAAGTTAATATAATGTTTGAGGTGAGTGAGAGGTAAAAATAGATTTCCCTTAATAGGTGATAGAGTGAGACTTATATAGCTATAATAGAGCGGTATTGGACTTGGGCCATCTTTTGGCTACTCCCAGACAAATGGTTTCTATTGTTTAAGTTTTGAAGAG

>Ca1:10312634-10312894

CTCTTGTAGACCCACACATTCAAGTGGTCACTATTGTAGAGGATTAAATTAAATATTAGGCCACATCAAATACAAATAACCATGGATGGTGACTAGGGGGAGAGCTAAAAAGTGCATGACTATGTGGCATACTCATCCTCTCTGTGAGATAATTTCCAGTGATAATCGTTAGATGTTCATGTCTCTTATATCTCAAACAATTTGTTTGCAACACTATTACAAGGGACAATGTATTAATGTAATATGTTGTGCATCTGTCA

>Ca1:10312545-10312894

GCTCTGGAAAGACAATCAATATTAAAGAAATTGTGGATTGAGAATTTGAGATTGTTACTGATCACTCTTCAATTTTTGTATCACTCTAGCTCTTGTAGACCCACACATTCAAGTGGTCACTATTGTAGAGGATTAAATTAAATATTAGGCCACATCAAATACAAATAACCATGGATGGTGACTAGGGGGAGAGCTAAAAAGTGCATGACTATGTGGCATACTCATCCTCTCTGTGAGATAATTTCCAGTGATAATCGTTAGATGTTCATGTCTCTTATATCTCAAACAATTTGTTTGCAACACTATTACAAGGGACAATGTATTAATGTAATATGTTGTGCATCTGTCA

>Ca1:10799548-10800074

ACAGCTACTTGCTTTCTAAGGTTACAAAGATCAACAGTTTGTTTATGTTCTGCCGTTATGCAGCCACTCCCACCAGTAGCTATCATCCTCAATAAGGATCAGTGACACTGTTGCATTGGTTTAACCAAAGAAGAAACATAAGGATAACTCACAGTTTGCTACGATAGAATAAAAAAACATTGAAGTAAATGAAAATCTCTCTCAACACACCACAGCTAGCTACCATCTTAGAGGAGCAGAACAATAAAAATAAGAAATGTATTAAAAGCTCTAAATAACAAATGTAAGAAATACATTAAAAAGTACATGTTAGAATATATTATACATGATCACAATAACATAAAACATAACCATAGTTGCAAGCACCTACTTTAGATTGAGCAAAAAGGTCAATTTTTTTAAATGATTCATTTATCTATGATTATGATTTATGTTATTGTCATGATGTACAACATATTCTTACATGTGACACTAGAGCCTTTTAATTTATTTCTTACAATTATCACTCATATCAGAGGAAATAAAC

>Ca1:11048621-11048833

ATCCTATCTGAACCAAACAGGTCAAACTATTAATAAATCTGCGGGCGGTGCGATAGGCAGTAAAATGCAATTACACAACCAACCTCATATAATGTTAGGGCCAGCTCCATACTATTCGCTATCTATCTTGTTATTTCCCATTTTACCCTTGCAGTTGCCGCCTCTTCATTTCACATCGATGCTACTCAACACCATCTTCACTCTCACTTTCG

>Ca1:11094947-11095158

TCATGATTCATGAATTTGAAACTTTTTTCTGAACAAAAAAACATTTTTTTTTTAATAACAGCACCTGGATGATGTGAATGCGGCAAAAGCAGAACATGTATGAGAGAATTGGTGACAAAATCAAAAGTAGAAGAAAACGCCCAACACTCTCTCTTTCGCTAATTCTACCGTTGTTCTTCACTCTCCACTATTTTTTATTTATCATCAAAAG

>Ca1:11960962-11963061

ACAATCCTAGGTTTATCTATTTGGGGCTTCACTGCCTGTTTATGTTAGCTTTCTTTCTTTTCTTTATGTCCTCACTTTACAGATCTATATATCAATCAATATTCTTTTTTTTATATATATATATATATATCCCCTTATCCAATCATAAACTATAGAGATATATGTTTTGTATTTGGATCACTCTACTACCGTTCTGTTTTTATGGTGTGTTTGCTTGAAGATAGATGAAGGAGAGATAATATTTTTAATTTTTTTTTATTTTGATTCAATTTTTAAGAGGGGATGAAGTCAAAATTTCTCCTATAGCTCGTTTTGCTCTCCCTCGATTTTGGAGGAATTTGAAGGGAAGAGTTTAATATAACTAAAAAAATCATTGAAATTTTTTTCATCTACTCTTTTGAACCAAACTTGTGTTAACTTCTCCTTTCTCCCCTCTATAGAACCAACCATGACTCTTAAATAGAGGAATTAGTAGATAGATATTCATAATCGAAAATTATTTTTGGACACTCATTCACTCAATGTATACCTTGACAAAGAACTTAATAGAGAGAGTGATATGATAGTAAAACTAATATGTTAGTGATGTGATAGTAAGAGAAAATGCAATGTTGAGAAATTTGAGATATTCAAGTATCATTATAGATACAAAATAGCATGATAGAGCTCTTGCCAAAAGGATATATATAAGTCTATACTTAAGACTTCTTTGGATTAAATTTTTTAAGTTTCTCTATTCACATAAATATTTGTGGCTGTATTTACAACAATTTTTAAAACCCTCTCAGAAAATCAATTTGTGAATGCTAAAATCCACAAAAATTGAGGAAAACACCATCAAATTTGACAAGTTTATTTTGTAGTTAGAGATGATTGATAGATGATATCGAGAAAAAGAGAAAATTAAGAGGGAGGCAAATATGTTAAGAAGATTTATTTGATAAAGTTGTATCAATTATTTTTGTTTTGATTACAATTTCTTTGATGCACCTCTCTAAATCAAGGATCGTAATGGAGTAATTGATACGTCTATAAATCACTAATGTGTAACTACTGTGAATCATGATCTGATAAGGATTATAATTTATTATCGTGATTATTTGGTACATTGAACATAATTGAGCCATTAAATGTTTTAATTATTCCATCATGACCCTTGACTTTTTTTATTAAATAAATAAATAGAAAATTAAACTGGAGAAAAATAAAGATAACAATTATATTTGGTATTTGGGAACTATAGAACAATTATATTTTCTCTAATTTAGTTATATGTCACGAGGCTGGTTGAGAAAATTAATGTGCTTTCTTGGATATGATTCTTGGGTAAGTAAAGGACATTGTCTATAGTACAAATGATGTGCATATATAATATATAATATATGATTGTAATTTTAGATAGTATGTATATATACCTTAACAAGCATATAGTCTCTAATATTGTCCTATATATACAAAGTGTATTTGGTTAAATTAATTACTCCTACACATTTTTTAATTTTTATTAATATTATTTGCTTTTTCAAATCTCTACATTCAGCTGTTTTATGGTGCCTAATTGTATTGGATATATATAATTGTTAATTACATTGAGATGCAAATTCTAAAAGCCATTGGTTTGGGAAAGGGGGTGGAATTTGGGTTCCAATGACCACTAGTCCTTTTTTTTTTTGAACTCGTGACCACTAGATCTTGTTACTAGATACATTTAATATTAATTATTTGTAGTTAGAGTTAATTTGAAGTATGGACATTGTTCCAAGTGGTATATGCATATGAGGATGAATGAAAATTGAAGCTTGGTTGGGAATTGAATAATGCAAAATTCACGGAGTACATTGTGTCAGTTTCGTTCATATTTTTGTCACAAGGCTTCTTTGGCTTTAGCATGCACTGAATTCATCACTCACTCACTAGTCAAGCAAACTATAAGGAAGTAGTACTCTTGCAACATTGCTAAGTCATCTCAAATGCAGAGATATTTCAACTCATCAATTATTCAATTTTTATTTCAACTGTCTCAATTGTATTTTTTATTTGGGTAAACTATCATTTTAGTTTAAAAAAATATAGGACGCTGTGTCATTTTAGTAGTTGAC

>Ca1:12211744-12212457

ATTCTATCTCGACTAAATGTAATTGCAGTGAGGTAAAATGGTTTTTTATTTAGAACTAAAATACCATCATTTCACCAAAGGTTTTTGAAAGAAAATAAAAAAAATAAAAAAAATAAAAAAACCACAAAAAACAAAAGGGTGGAGTTTTGCTACATGGGAACTTTGGAGTGGATAAGACTGAAGAGTGAAGTCACGAAAAGAGGCGGCAGTTTGGAGAGAAAGATTGAAATGAAGAAGGGTAAAATCGTAAATGAGAATGAATATGGGTAATTGTTACGAGGAGATGAGGGATAGAGAAAAGAGGAAGTGGTCCTTGGTTCGTAGGTATTTAATATTATCGATGACACTTGTGAAGCGGAGGTTTGGTGAGAAATGGGAGGAGGGGCGTGATAGCACGTGAAGGAAGTAGACCCTTCACTTGGATCAAGTGCTTCAAGTTCCTTTCCTTTCTGCCACCTTACTCCTAAATTATTGTCTTCTCTTAATGGGACATCCCAATTATTTCATCTCTTTATCGTTTCAAACTCCTAAAATTATCCTCACCCCCTTTGCTCATTTGACGCTGCTAATCTATCCACATGGACCCATCTCTTTATTCATTTCTCATTGCTCACTTTTTTTGTCCTCTTCTCACTTTATCTAAGATTTTTTATTTTTTTTTATTTTACTTTATTTATCACATATGTCAAATGTGTATAGAAAATGTTAACATG

>Ca1:12785013-12785263

GGCAGGGGCAATATTGTCTACCAGATATGAAAACATGAGAAGATTAAGATGGACACATATTGATGATAAACAAAACTTGTCTTGTTAGATTCAACTTTTCTGTGGCTTTATTTATTTTCTCTCCTTATTGTAGCCATTAACATTGGTGCTTTTTAAATTTTCAAACAATTGAAAGTGGGTTTCACTAGTTAAATCAATGCTGCTAGATGTATTTAATTACTACTATGTATTGTTTCATGATAGTTTGCTC

>Ca1:12879580-12879930

GTGTGACGCAACGAAGAGATCGTTGAAAACTTGAGAGACAGGTGCTTCCCTATGTTTGCTGCTGTCTTGGTATTGAGCTTTAAGTTCCTTTGTTATGGTTGTTCTAGAGAAGTGTTTTCACACAAAATTGCTAAGCCCAATAAGTTAAAAATAATATAATTGAGAATACTAAATATACCCTGTATCATCTAATATTTGCATAATTTTATTATTTGATTGTTGCTGATGGTACCTTGTTTCTTACACATCAATATTCTGATTATGTTGTGTATTACTCATGCGTTATGCAAGATACAATTAGATGCATGTGTAAAACTTTTTTATATCGTACTTGTATACTTATTAAAGAG

>Ca1:13383227-13383462

CCCCTACCGAGAAAGGCAAATTGGCAAACCAAAAATGAATACTAACGACAGGGACCTACCAAACCAACTGCCACCAACTTATTAATCAGGTCCTACACAATCCTTATCTCATTTATTAGACCTCCTCTTTTGACTTCCACTTGTCACAACTTCTGTCCTTCAAAACAACTTGACGGTCACAGATCCATAAAAAAAAACCTCTAAGAGGTTCCGACTTTAACCACACTACTATAAG

>Ca1:13593010-13593220

ACAATGAGTTCAAGGTGATGCAAGGTATGGTCCCTTCCAGCTAGTGCAAGATGAGAGTGGTGTTTTCCCAAGTTCAGTTCCATACTTAGCTATCATGAAAAAATTGACACAAGTTCTTTTACTATATATTAATCTATTATTATAGCATTTATAGAACAAACTGAATATGTCACCAACTATTTCTTTTCAAGCCAACAGTACTACTACAAC

>Ca1:13593010-13593699

ACAATGAGTTCAAGGTGATGCAAGGTATGGTCCCTTCCAGCTAGTGCAAGATGAGAGTGGTGTTTTCCCAAGTTCAGTTCCATACTTAGCTATCATGAAAAAATTGACACAAGTTCTTTTACTATATATTAATCTATTATTATAGCATTTATAGAACAAACTGAATATGTCACCAACTATTTCTTTTCAAGCCAACAGTACTACTACAACAATCCAATATTTGTTTGTGAAATTTTCACTCATGAAATTTCCACATCTAAATTTCATCTCATAATCTATTGTGGGCCACCAAAATACAATCTTAGTTAAATTTTCACATATGCAATTTCCACACCTAAATTTCATGTATCAACCTTGGTAAGGGAATTTGTGTTATATCTAGGACAGCAGCCAATAATCCCTCCCTATATAAACTAGATCATTCACCTGAAATTAATTTTCATAAAATCTTCATTTCTATATTCTTTCAAACTCAATGTAGCAAATTGCACCAATTTTAAATTATAAATACATAGTTTAGATAAGATATGATAACAAACATTTAACGGGCATAGAGAATGTGAACATAACTTGATTAGGACATTACAACAAACAGTTTGCAGGCAAATGAAGCATAAATTAAAAACTTCGATTCAACTTATCTGTGATGAGAAAGAGCACAAGCACGATGAACATTGATACTTCACCAT

>Ca1:13593818-13594130

ATGAAGAAAAACAGAGAGAATAAGGAATGTGGAAAGGGAAGAGATCGGCGCAGCAAGTGAGATATGAAAATGGAAGATGAAGCGGAAGAGAAGAGAGCTCACCACTGGGAATTGGTTAGGCGAGCGTTTCAAGCGAAAGGCACGATGCTAAGCTGCCACGTTGGCTACTTGATTTAACAGAAAACGGTCATATGCGATGTGAATTTGTCTCAGTAATTCCGCGGGTACTATCCCATTAGGGCGTGGGAAACAAAAATGATATTTAAATGCCTCTGATTTTTTTAATTATGGAATTCAAAGCTTTATTTTTTT

>Ca1:13865146-13865511

TTTTGTTTTCTAAACATTTGCTTTGGTTTCAATGGATTGCTACTGACTTTATTTTGATTTTATAATTATTATTCAATATTTGAAGCCTGGGTCACCTTGGATCACTTGTTCTATTTGATAACCCTATGAAGCAATTAGAGCCTAGTAGCACCATTTCATGTCTATACCTCCATTGAAGTCTAATGATATTAAGGAAATAAGGAATTTACACAATAAAATCATCTAATAATTCTGTTAAATAGTTAAACAAGACCTTTTGAGAACAAAATAATTCTTTTTATCTTCTGTTTTTTCAGTCATGGATATGGATGCAACAAGCAATGCACCCTGAATTGAATTACAATAGCAGTTTAAGGAAAGAATGG

>Ca1:14106695-14106960

CTTGCACATCTCAATAACCACCCCTGCCTAAATAGTGTGGTCAAAGCTTGTGTCCCCTGTTTGTGCCCCAAGATTTATTGAGGTATGATTGTTGGGGGTTCTCTATTTGTAGGTTTAGTTTCTTTAGGTTTGTCTCAGTTGATTGGGAAGGTAAGGAAGAAAAATCGCCAACTATTTAAATATAATTTCTGTTAGATTTGATTGAAATTGAACTATCTTAAAAATATTTTATATTGTCTACAATTGTATGAGAATAGAGAAGCCA

>Ca1:14179030-14179498

GTTGTGATGGATAGTGTGCTCATTATAATGAAGGCCACGATGGAAAGAAACATGAAGCTAAGTGTGGAATCATTGATGACTTTTAATAAAGATCATCAAAGAAAGTGGTCATCATCATAATATCAAACCAAAATGTACCCTCTTGTACAGGACAAGGATAAAATAACCAACCAAACAAATAGGTTGATGTTGTTGTGTTCTTGTGCTGTTGCTGAAGAGAGTTCCCCTTGACAACTTCAAAATTTGACTTTGTTTAAATTAATCATTTGCTGATCACTCAACATGATCACGGCTTTAACCTCTTTTTGTTCTTCATCATTTTAATAGTACTCATGAGTTTTATTTTAAATTTGGAATAAAATGTAATAAATATATTTTGATATTTTAATTATTGCCGTGAGAGATTATAGTTTTTGCTGTTTGTTTGTTAGAAATTAAGTTTGCTGAACTTTGAATTTCTTGTTTGAG

>Ca1:14245652-14246199

ATCAGTTGTGCTAGAAAATGATATTCGCTTTGTTCTTATGTTTTATTTAGAGTAAGAAGGTCATGCCAGAAAATAAGATTCTTATTATTTTACTAATGAGGATTAATGTACATAGTTATATATCAATTTTTGTATCCATTTATCATGTTGCAATTTTTGCTCTCCCTCCCCACCCCCACCCTAGTATAGTTGTTTTCTTAATATTAATCCATTTGGCTTGATCACACTTCAAGTTATTTATTTGTCTCTCGAATAATTTCCATGGATATTAGCAAGGTTGGTTTTGGAAGCGGTGGCGGTGTGAATTGTTGGTTATACAGTGAGTCTCCAGTGGGTTCTTTTCTTTATCGACACTCTTCTTCTTTTACTTGTTCTTCATGCCTACGTGATTGCTCTTCTCACCCTTCTTTTACTTGTTAAGCGTTTGTTCTATATACTTTTATCTATATCATAAAATATTTTGTGACCTAGACTTCTATTGTTGGATTTGTTTGTGATAATTGCTAACTCGGTGATACAAATCATAGTCAACAAACACATTGCTACG

>Ca1:14378033-14378716

ATGCAAAAGGTCATTGAAGATATTTATGGCGAGATGATATTGCTTGATGTTGCTGTGGCTATAGCTTGATGGGGTGTACAGTTTGAAGAGGCAATGTTCGACTCACAACCTGTATACCTGAAACTCTGATGGAGAGACTCAATTATCATTAATGATGAATCAAATCAAAGTCAAAAGAGAAAACAAAGAGAGATAAGTATATATACCTGCATGAGTTAAAAAAGTGAGGCAGGTACTTTTCTGGTCAAGTGTATTTAGTTGGTGCAAGTGCTTTTAAGTAATTGCCATAGAAGATTGTTTAGGTAGTTTTTGTCTTCTGTTGGTTTTTTGTACATATGAAGCGTGTAAACATATTGGTAAGTAAGAAGCTCCAAAGGTGTACTTCTTTTGGAACAAACATGAGCTGTTGCCTAAACATGAGGCAACAGTGACTGCAGTGATATATATATTAAAAAAAAAAAAAAAAACTGTCCAAATGTGGGCAATTGCTTGTGCATTCTGCAATTTATCCTTTTTGCATCTTTGCTATTTTCCTTTTCTGGTTTCTTAATTCTATTGGCCCCCCTTCTCCTTCTCCTTTTGCTTTTGTTCTTTTTGGGTCTTTCTGTTTACACACGCGATCCCTTTTCTTTTGTGGAAACTGGAAAGTATCTGATAGTGGCATTAGTTTTATTGTATTGGAC

>Ca1:14603857-14604238

CTCCTTCTAATTATGTTTTTATTTTGTGACTTGGTTTATGTTTTTGTATAAGTTCATAATTCTATTCTGTGAGAGCAATTGCTGCATTGAAGAGACGCTAATAACTCATCCTCGTGGAAACAGTGAAAAGAACACCGAAAGCATTGATGCTTTTTTGAATTTGTTGAATCTATTGTAGTAAATATTGGTTAATGTGTCAATATCACATGAGTGTTCATGACTTGTTTGGTGATACAATATTTTTTATTTTTTTTTATATCTTCCTTGGTTGTCAAATTATGAATTATGTCTCTAAGATTCTAATAATAGAGGGCTGTTTTGTTTAGTTGTTTGTGTGTGTGTTTATGCAACTCATCAATGAAAAATCCAGTATAATGAAAA

>Ca1:14725106-14725625

AAGAAAGAGAGCGAGGAGTGAATAAAAGGAGCGCAAAACCTAACTTAAATCTAAACACTACTTGATCTTATCTGAGCAGAGAGTAGCCGCCGCCACGGTTTTCACCTTTCCGAGTCTATCATTTTCTATCTCACTCTCGTCGGATACGCTGACGGTGGAGCCTCCGTTTACGGCGGACTCTGCCCTCGTACCCGCACTGACCGTGAGATGGAGATTGTATCTATCGGGTTTAAAGGTTTGACTGGTTTGCCGTCGGTAACCTGCGATGAAGACTGCCCGCGGTCAGAGCCCCTTTCCTATTGGTTGGGGCTGTGAAGGCCACGCGGGCTGCCCAAATATCATCAACTTTTGCTTTTGTTTTTTTTTTCTTTTATTAAATGAATGTAAAGATTTTCGCATGTGCTTATTAATTTTGAACTTTATTTTACATTTGTTTTATTTGTTTTTGCTATTTGGCAATTTATTTTGTGTTGCTGAATTCAACTAATTCATTTGGACTTGTTAGGCCCTCTTTTTAAG

>Ca1:14725717-14726010

GTTCATATGCTGCTTGGTGTGCAGCAGTACTTTGAGAGTGATCCTTGGAACTTAGGGCTGAGTTCTCGAGTGTTTTTAGGTTCCCAAATTCATTTTCATGTTTTCGTTAATTTGTTATTCCTTGTACATATAAATATAATAAATTTTCACGTCAATTGAATATACTATTTATCTGTTATGTATGATCCAATTGCTATGTTCCAGTGGATATTCTACAAGTTAGTCAACTCACGAAATGCAAGAATGCAACTTGTATCATTTTGATTGACTTCAATATATTTTCAACTTACCCT

>Ca1:14725170-14725489

ATCTTATCTGAGCAGAGAGTAGCCGCCGCCACGGTTTTCACCTTTCCGAGTCTATCATTTTCTATCTCACTCTCGTCGGATACGCTGACGGTGGAGCCTCCGTTTACGGCGGACTCTGCCCTCGTACCCGCACTGACCGTGAGATGGAGATTGTATCTATCGGGTTTAAAGGTTTGACTGGTTTGCCGTCGGTAACCTGCGATGAAGACTGCCCGCGGTCAGAGCCCCTTTCCTATTGGTTGGGGCTGTGAAGGCCACGCGGGCTGCCCAAATATCATCAACTTTTGCTTTTGTTTTTTTTTTCTTTTATTAAATGAAT

>Ca1:15020247-15020500

GGTAGGTGTGAGATTTGACCGAACGACCACAAAATACCATCCCTTCAAGACATGGTACGCTTGCTTCACTCTTCCAATAATATTTATTTAAACCCCTTAACCACCACCACTTCTCTCAGCTTGACGTTCCCTTATCAAAAGAGAACACCAAGCTTACAACACAATAACTTAATAAATATCATACACTATTTCCCTTCCCATTTTTCTTTCTTTCTTTCTTATTTTTAAATAAATATGCACACTCTTCCTCCGC

>Ca1:16079874-16080184

GCTCTTGATAGATAATGTTAGTTGTGAAATTGTGTGAAAGAGGTTTATTTATTTTTTATGTTGTTATTTTCCTTTGAGAGCACAACCACACCCACCCAAGTCCAACATAGGCATAATTCCAGGCTAAATATTTTGTTTTTGAATGTTAGAATTTGTCGATGTTATCCCCGAGTCAGGATCAAACCCTCGACCTCCTTATCACTTCACTTTAACTCCGGAATTTTCCATCCTAAACAAGCAAGCCAACCTTATATCTCTTTCCAGGTTTCTATGCTTCAAAATCATTAGTATCATTTAAATGTTTTAGGCC

>Ca1:16544408-16545134

GGTGACTATGCTGAAGTCAAGTCTCATTGAGTATAACAGGTTGTTGTGGCTTGCATTTTCTGGTGCTTAAGGTGCAATGAGTGTAGTGAGCAGAATAACACATTGTAGAGAGGATAATAGGAACATAGTGAGCAGAGTAGCATTACAATTGTAAAATAAGTTTTGATAGTGATGTTTCTCTTTCTCAGGTCACACCTCTAATATTTTTGGTTTCCTAAAACAGATTCTTCAATCTTCTTTTCTTGTTACAACTTAACTATTGTTAAATCTTTGCCAACAACGAGCTCGAACTGCATACTAAAAAGCTCAAGGATTTAATCGAGTAAGTGTACAAATCATTCAGGATACAACATGCTCCTCTCGTCAGCGCATGAAACTATGTGGTATGGTATACAATCGTTTCAATTGCCTTTAAAAGTTTTTCCTGTGTTGTGTGTGTGGTAATGGTGGCAATGCCATTATTGGGAGGTGGAGTGACGGAGGAACTGTGATCGTCGGCCATAGAAATTCTTGAGATTATTGTGACTCTTGTTCTAAACCAAATGAAGCCAAAAGCCCTTGCTTGCCCTTGCAGGTTCACACTCCTTTCAGTTGGTGTCTTCAATAATTATGTGTATGATCAAACTACTCGTTGAGATTAATCGTGTGATAATTTGATATAACATTTTGCTTATCATGACTGTCAAAATAATTATATGAAAGGTTTGTTGTAGAAGATCAGGGATG

>Ca1:16588658-16589194

AATGAACCAATCTTAGAAGGTGATACTGCAGGCTTACCAACATTACAAGGACCCGATCAAAAAACAGTTCCCTTTCCCACTTACATTTCAATTTCCAAACAATACAAAATCACCAGCAACCCCCTTAACCGGTTAACCGGTGAACCCTTCAAACTTTCAAAAGGGTTTATACACACACAGACACCGCCGTACACACTATATAAAACACGTTCTTGTGTAGCATCAAAAACACAACCAACAACGAAGTTGGGTCCCATTAGCATTTTCAGTTCACTATTTCTCTCTCTCAAACACCGCTCAATCTCATCACTCACAAACAATGCCATTAAACGCAGAAGAACCTTGCCGTTTTACTCTGACACAGCGAAAATAGCAAAACGACGAATCAAAACGCGTCTTCTAAATAGAAAGAGAAAGAATCAACAACTCTACCCGTACCACAGACTCATCCACTCTCACTCACTCATGAATTTCTTTGCTTTTTCAATTTCATCATGAAAATTTGGATCTAATTCCTATTCTTCAATTTCTTAACT

>Ca1:17522316-17522592

CAGCTGCTTTATTATCATAAAAAAAAAAAAATATGCAACCGCTCCCAATTTGCTTTCGTAGTATCTATCTATCAGAAACAAGTTTGATTTGAGTCGCACTGAACTCACTCAACTTCCCGGTAAACGCTGCCGGAGTTCGTTTGCCGATGCGATTTACGGCCGCGATAGCAGATCTCTCCTCCTCTCCATAGAATTGCGTCGCAGCAGATTTCGATTGAGAGTATCACTCACTTCAACTGAATGAAATGTATCTGTAATAATTGGTTACGTTTGAAG

>Ca1:17522363-17522592

ATTTGCTTTCGTAGTATCTATCTATCAGAAACAAGTTTGATTTGAGTCGCACTGAACTCACTCAACTTCCCGGTAAACGCTGCCGGAGTTCGTTTGCCGATGCGATTTACGGCCGCGATAGCAGATCTCTCCTCCTCTCCATAGAATTGCGTCGCAGCAGATTTCGATTGAGAGTATCACTCACTTCAACTGAATGAAATGTATCTGTAATAATTGGTTACGTTTGAAG

>Ca1:17769013-17769301

AGCATATATTATTGATTTTATTTTTTTCTGCTATAAATTGTGAACTGATGGATTTTTACAGTGAACCAGTTATAGATACATTCTTTACAAGAATATGGATCTCTCAAGCACTCACTTCTCTCTGACCTCTCGATCCTCTCCGTGTCTCTCATAGCTTCCGTCTTGCTCTCCAGTATCCTCTCTATCTCTCCACTCTCTCCCCCTCCCAACTTTGTCCATCTCAGCACATCATAATCCCCCCTACCCCAATTTCCCAACTCATTTGCAGAATTTGTAATAAAAAGAAAC

>Ca1:18017134-18017813

ACAATGGGACCTGAAAGATCAACAAGAAGTAAACTCATAAGGAGATGAAGTGGCTGCAGCTATTGAAGTGAACTTAAAGACACAGAAACTTCACTAGATGTGAACCCAGATGGACAAAAAGTGCAGCTATTGTAGTGAACCTAGAAGGACAAGCAATTGCTGCATCTGCATGACTGCACCCTACCAAAGATGAATGACTCGGTAGGCCATATAGCTGCTTCTAAATGCGCTGCTGGTTAACCATAATAGCTATGCTTCTGGGTTCACTTCATGCTGACCTCTAATTGTCTTAATGCCAATGCCAATTATTAGAAGAAAAAAAAGGAACAGTGTTTGTGATTGAATGCAATAATTATTTTTACTTTATCCTTTATATATTTACATAATAGCAACTTAGGTCATTTCTACTTTACCCATTTTTTCACACTTCCATTGTCCTACACAGCATTTGTGCCGATCATGCAATAGCTTTGGCTGTGAGCTGGGGCAGAACGTGTAACATATGACGGCAAACCCCATAATATCAAGTTGGTATATTTTTCAGGTTACCTTATTCAAATTAACAATGCCTCTTAGACTTTAGGACATCATTGATCAATTTCTGAGGGATACATCAAATTGTTGTAGCCAACTCTAAAGATGAGTTTGCAAGAAATGTGGCACTATCTGAGATTTAAAT

>Ca1:18680613-18680958

CGCTTCTCAAGCACCCTTAGAGCCCCCTCTAGACAGGTGGAGGTGGGCGACTAAATGTCGTGCGTCATATAGGGTTACCGTGTTGGCTAATGGATTATTGTGAGGTGGGTTCCAAAAAAATTTGGATTTCTTTAATGGTGGTGAACAAGCGGCTTTGGACTATTGTAACAATAATTCCAATAGATCGAATTGTGGTAATACTTTAAAACTCATTAGACGTGTTAAGGTTGTATGAAGCACTTGACAAGAATGAATGAAAAGTATCATAGCCAACTTATTAAAGGAAATGGAGAAAGTCAAAAAGTTTAGAGGCAAAGTATATCTATGGAGTCAGATCAAAATCAA

>Ca1:18687000-18687296

AACATTATCCCTTTCTTCTTTTTCTCACCGATCCCTTTCGTCTTCGTTAGTGTCCTTCATCCTCCCAATCTTTATCTTCAACCTCTTATTTTCCTTATTTTCCTTCATTTTTCAAATCAGTTAAACCAAAAGCTTTTAGGACCATTTTTAAACATGCATGTCTCATCTGTTCATAACCAGATTCAACCAAGGAAACAACCAGGGATAATAACCCAAAAATGAAGAACTTGAGTTTCAGGAAAAAAGGGAGCTCAACAATCAATCTAATTAGAACGTATTCTCCCTTATCACAAACT

>Ca1:19178502-19179303

TGATACTCCATTGTATCCTGAAGCTTTCACTGTTCATTTGGATGAACCATCTACAGCTACAATTCCACTCATGGATTTATACCAACAAGTAGAAGAGACTCAAATTGTCGATCTTATTCAACTAGCTACAAATTGGCTTCAAACTTTTCAACCAATCTTCTTTCCAAAGGTAAGAAACACCTTAACCTTGGATTAGAGGCTATGATTCATTTCATTGTGTTTCCTAGTCCAATTTATAATTTATCCATTCCTCCACCTCCACGAAAAACAAACATCACAACCACCAAGCCTCAACCAAACATCTCTGCAACCTTTGACACTTCCATCTTCAAAGAAGGGTGTGTTTCTAAAAAAGGGAAAACCACTTAAAAAGTATAGGAATAGAAAGTTCCTTATCCTAAGTGCAATAAGCTCGAGAAAGAAGTGAGCAAGACAATGAAGAATCTCAAAAAGATGTCTGAGTGCAAAACGCTTTGGTAGTTGATTGGATTACAAGGAATGTGCTTCCAAATTTATATTTTGTCACTAAAGGATCTTCCTCGTTTATCCACTTTTTCATCCTCATATGACTCTTCTCCTTCCAAGTGAAATGACTTTTTGTTGGATCCCTCATCTTTTTGTTGATGCCAAATGAGGGAGAAGAATTAGGGGAATGTTGGGGAAGTTTTACTTTCTAACTATTGAGGTAGCTCAAACTTAATCTTTTTGTTGACTTTTGATTAGCTCAGTCTTTTGTAATTGGTATATTAAGCTAAAATTAATTAATATTTATTTATGTTTTAATCATCATGCTTATGGGAT

>Ca1:19182751-19182965

CATACATAAACTTGTTTATTATTCATCTATCTCTTTTCAAAACTTTGAACAAAATCATTGCTAAATTGCACCATGAATTCTAAAGACAAATCCGCTGGTAGCTATCTTTGTCCAACTACTAGGCCATCATCATCATCATCCTCAACATCCACTACTGGAACTAAACATCCAACAACGACAAACGTCTTTGCCAAGAGAGAAACTAAAAGTAAAA

>Ca1:19193194-19193398

CGTGTTTATTATTTATCTATCTCTTTTCAAAACTTTGAACGAAATCATTGCTAAATTGCACCATGAGTTCTAAAGACAAATCCGCTGGTAGCTATCTTTGTCCAACTACTAGGCCATCATCATCATCATCCTCAACATCCACTGCTGGAACTAAACATGCTACAATGACAAACGTCTTTGCCAAGAGAGAAACTAAAAGTAAAA

>Ca1:19283340-19283614

AGTACATTTCATTTTTTTTTTTGGATAAAAAATAACTGCATATATCGTATATCATAGATATATATGGTTGTCCTGATTCCGAATCCATGAGCATTTAATCCCGAGGGCCACATATTACATACGATACACTATATTCGCCACTCTCAATCATATAAATCCAAATTAAACACCAAAAATAAAATAGTAAATAGAACTAATAAGGAGTTAAAAGCAGAAAGAAGGATTCAGCATCAGAAGCAACGAAGAAGAAAAAGATTATCACTTTTTTTCAAAA

>Ca1:19284019-19284251

TGATTATGTTATTTTCTTTTGTTCTTCAAGAATTTCTAACAAGTCAATTGTTTTGTTACTTTTCCCCTTTGATATTCCTTATCTTGATAATGTTGAATTTTTTTGATTTATATGTTTTAGAGTTTTCATCCTCTATTTGCTTTGTTGTGCTTTTATAGTTTTGAGATATGTGTTGATTTGAACTTTGAAGTGTATTGACGCATTGTGAATCGGGGACCTTGTCGGTATAACG

>Ca1:19365075-19365539

ATTCATCCAAAAAAAATATTCACTTATTCACCCAAACATCAATTCACCCAACAATACATTTTCCCTTTCTTCCCCATTTCCATCTTAGAATTTTTTTTTTCTATTTCTTCTCAAATTTGCTCAAAAGGTCGAGGAATTATTAAAGTTCTTCTAAAAAAATTCCTATTTCATCTTAGAAATTATTGTCCCAATTTCTTCATTGATTCTTACTTTTCCTTTGAAGAAGTTTTTCTGGATTTCTTCATCATTCTTTAACCTTGAAGAAGTTTTTCCCGTTTTGTTAAAAAAATTCAATTCTCTCTTCAATTGATTCCTTCAATTTCCTCTCCAATCGTTTTCCTTTAGTTTCCTCTTCAATATGTATTCATCTTCAATCTTTTTCCTTCAATCAGATTTCTCTTACGATTTAGTGATTTACGTTTTACGATTTTTGATTCACGACTAAGCAGTTCAAGGATTCATGA

>Ca1:19660199-19660419

GTTTATAACAAGTAAACTCCACTACTACAAGAAAGCGCAAGTTTGACAAGAGAAAAATCCCTTAAAAATGCATAAAACCCCTTTGAAAAGAAAAAAAAAATCCTAGGGATTTCATAGGAGTCAAAAATTTCTTTGAAAACAACTCATCGAAAACTTTTTTGAGGGGTTTACTCATGAGAAATTGATTTTATTCGATTAAGATAACTGTTGTACTATCCCG

>Ca1:19917105-19917530

CTCTTAGTCATGTTCTTGTTTCGATATACTTGAAAACCGGCCGAAACAGAAGTCGACTTCCCCGGATCAAAACCAGAAGGGGGGGTATTTTGTTTGGATTCTCACGGACAACTTTGACTTTTGCCTGAACCGGTTCAGGTAGTTTGCTTTTTCTTTTTGTTTTTTTGTTTTCTTTTTGGATTGTGGCTCAGAGTCCATTATGCTGCAAAATTATATGGAGAAATTTTTTAGAATCAAGAGGGTTTACTAGTCACAAATGCATGCAGAAATACTCGATGAAAATAAAATTAACAAGTCTTAATTGAATCGATTATGAAAAAATTGAAAAGAAAAAAGGAAAAGGTGGAATTTCTCACGATATGTGGCTAACTACGAGGAACTCCGGCGGCGATTTCAGGTGAGTTGAATTCAGTGCGAGTCGGACT

>Ca1:21777573-21777776

AGTGGCCGCTTTGGTGTTTACAGAAAACACAAGTAGAGTTGGTGAGAAAGAAGAAACAAAGAATGAAGGAGAAAGAGGCAGAAAGATACCTGAATGAGGCCGCGAGAAGAAGACCGGAATGAGGCCGCGAGAAGAAGACCAAGGAGGATGGTGCGGTCGCTCCAAAAGGCACCGGTTCCAACGACGAGGGTGACGATATGAAG

>Ca1:21778147-21778415

TTACAAAGCAAAGGTCAATGACAACTACATCTGAAACAGAAAGGCAGCAACAAAATAGAGCCAAACACTGCATCAGAAACAGAGAGGCAGAGAGGCAACAACAACAACAAATTAAAAATTATTGGAAAATAATTTAGTTTGGTTTAAATCTTATTGTAATTTAATTTGATTTAATATTTATGGTTGCAATTTGATTGTAATTGTAATTATAACTTGATTTAGCATTGTAAATTGATAAACCTACTTATTGTAAATATAATTACTTCCT

>Ca1:23691016-23691926

GGGACTTTTGAATCTTTCATAGTTTTATGGATCTTTCTTCCTCCTCTTTATACTTCATGTGTTGCTGTAAGTGGTCTTTGTGTGGAATTCTTTGTATTTGTGACTCGTTTTCCTCATGGTTTTCAGCTTCTTCGTATTTTCATTTGTGCAACTTGGAGTGTTATTTTTTCCAACTTGAGATGTTTAGCTATTTCTTTGCTTTGTTTCCACAATAGACATGTGAAAATGTGTGTGTGTATTTTCGCTTGAGTGAGAACCATTTATGGTTTCATGTTCTTGTACGGTGGCACCTAAGATGCTCTTTCACCCTTTCACCCTTTCGTGTTCTTACACTTTTTCTTTCTATTGCATGGTTACAAGATATATAAGATGTTGACTTTGCAGAAGATGATTGAGAGGAAGCAAAATAGGAATTCCTAGAGTCAAGGCACTTCTATGTTGGAGCCTCAAGGCAGCCACGTCCATGCAATTCTTTTGAAATAGATGTCGATGGTTGAGCACAAAAACACACGAGACTAAAATCATGTCGACCTAAAGCATCTTCAACTTCCTACATCAGGCACTACGCGTCAAAGTAGCAATTTTCTCTTTTTGTTTCTTAGATGACTGTGTTGCGTGGCTGACATGCACCCCCCCTAAGCCGTTAGAATGTATCTTCTATAAAAACTCATCGATGGCTCTCAAATAAGGGCTTTTTTATAATTTCAAATCAAGCTCCTTAAATATATCGTTGGTAAGATTTGATCTTTTCACTATTTGTTCCTCAAAATATTGGATTGGTATCATAACTCATATGTACTTGTTTAAAACATGTGTTGGTTGATAGTTGATTACTCAAAGTTGTACCAAACAATTTATAAAGTTTCTTATTCGATACTGAAATGCATTGACCAAGTTTTATAAGCGTCGT

>Ca1:23946004-23946364

AGGAGGTCGCCGAGGGTGTCTGATAAGGGAAGAATTTGCTTTGATTACAGAACAATGGCAGAGTTTCGAGGGTCTGTCCAACTATTTCCCTTACACAAACAAAATACATTACTAATCCATTAACCAACTAAGTCTCTTAGCTGCAACAGATAGACCTATGACCCTCAATGTTCACAGAACCGCTAAGGTCTATGAAAGAAGGAAGAAGAGGTCGGGAGTAGGAAATCAAAACCAAGAAAGGGAGAGAGGAGAAAATAACTTACTGCTGAGGTAGTGGGGCGTGTGGGTGTGCCGAGGTGTCTCCGAGGCACTAAAAGTGTGAGTGAGCTAACAATAAAAAGGTGGAAAGAGACTGAGAAG

>Ca1:23946004-23946481

AGGAGGTCGCCGAGGGTGTCTGATAAGGGAAGAATTTGCTTTGATTACAGAACAATGGCAGAGTTTCGAGGGTCTGTCCAACTATTTCCCTTACACAAACAAAATACATTACTAATCCATTAACCAACTAAGTCTCTTAGCTGCAACAGATAGACCTATGACCCTCAATGTTCACAGAACCGCTAAGGTCTATGAAAGAAGGAAGAAGAGGTCGGGAGTAGGAAATCAAAACCAAGAAAGGGAGAGAGGAGAAAATAACTTACTGCTGAGGTAGTGGGGCGTGTGGGTGTGCCGAGGTGTCTCCGAGGCACTAAAAGTGTGAGTGAGCTAACAATAAAAAGGTGGAAAGAGACTGAGAAGGTTGGCAGGGAAATATTCATTTGGTCTTGGGAGACCCTAGGCTTCTCTAACTGCCTAGTTTACTTTCTTCTATTGCATTTAAGCTTTCTTTTGTAATCACGGAATTGCTATCACACT

>Ca1:24446156-24448136

GATGAGCGAAAGAAGATAAAGGTTTACTATCCAACACATTGTGATTTGAACTTTGAGCTATTAAAAGTTGAGAAAAATGAAGTGTCTCTCACCTGGAGAAAAATAATTTTGGGGAAGTTTCTTGCACTCCCTTGTGTCAATTTGTTTCTATATTTGTACAGCCAGTTTGAGTTAGCCACAATTTAGTTCTTTTGTAACTTTGAGATAACACGTAAAGTTCTTTCATGAGATTATAACATTATGAACCTAAGTGGATGATACATGAGACAACATAGGTGTAGAAGTGAAAACATTAGGGATAAAGTTGCGTACCATCCAATGTGGAAAACATGGTGGAGTCTTGTTAGATGGTTTGAGCATGTGTGGGGAAAATCTAGAAAAGCTTCAATAAGGGAGGGTACATCATATAGAGGGTAATCCAATACTTTGAGGTTGAGAGAGATTAAGGAAAACTATAAGCTAAATTTATGAGTAGCTATTTAGATTTAACTGTCATTAGATATGATCTATGACAAATATTATACTTTTACTTGATCCATCCATCCATCCATCCTATAACCAATCCTTCCTAGTGGGAATTTTTTGTCCTAGTAGCTTTGGATTAAGGTGAATAGTATACATCAAATGGGAAAATGAGATCATTCACCATTGAACAAGAATTCACATTCCCTACTGCTATAATGAATACGGTGTTCTAAATGTGTTGTCATTTGGGATGTTTTATCTGCATAAATAGATGGTCAATTGCCAGTCGTTCAATAACAAGAAATAACATGATCAAGTTCTTAGTGCAGGTTATGATACAGTATTCATTTTTAAGCAGTGATGCGTTATGATATTTTGCTGTTATCATTTTCGGCTACCGTGAGTTTTTATTTTCACAGCTTTGGTTGATATGCTGATAGAACACTGCAAATTAAGAGGAATCAGGGCCCGAAGAGTGGGCCCCACTCACCCAAGTCACACACCTCACACACATTTAGTTTCTAGAAGGATGAATGGAGGAAATAAAAGCTAGTTAATAATGTCTCTTATTCTATTGATTATCCATTAAAGGAATGAAGGAAGGGGAAGTGGGAAAGGCATTCAGTTTCAAGAAGGATGAATGGAGGGAATAAAAGCTAGTTAATAACTCTCTGGTTAGGAGCAATTTTGCTCTGGTTTAGGAGTTCTTAGAAGTTAAATCTCTGGTTTAGTTTTTCTTTTTGCATCTTCTCCTGATCTTGTCATTGTAATTTTAAGAAATAATATACCATTTTCTTCCATTAAAATTCTGAGTTCTATCATATGCACTGTCATACTAAATCATATATTTATAAACCATCATAGTAAATTTTGATTTTATCTTCAATAAGATGACTTGCACTCTTCAGGCTCCGACTGAATGTTTTCTCCTTATCTCTTTTTCTTTTTTTATAAATTATGAATTGTATTTTGTTTGCCTAGTTATGTGTTTTGATTGATACCACATTCATCCCTCTATTGCATACATGCCTCTAACATAGTATTGTTTTTAGCATTTGTAATGTGAGCTTGTGGCAGAGGCCTTCACTCTTTATCCTCAATCTCTAGTTTATTTAACTGTGGCAGATTTCTGATTGAATTATTGTTTGCAACCTGGCATTCAATTCACATCTGTTTTGAAACAGATCATTATCTTGCACTGTATTGAAATTAGGCAAGATATCTTTATTATGAAAGCGATATGGAATAGGACTGCAGTTTGGACATTAAACTAAGTACTACTCTGGACTAAGGACTCAATTATAAAAAAATAACTATTTCAACACTTAATAACGATGATAGTTAAATGCGTTTAGTTTTCATGATTTCACGCAAAAATGAATACATTTACTAACCTACGCCTAATTATTATGATTATCTCGTCACAACATTATTAGATTAGTTTAAGGGTATTTTTGAAATAATAGTATTCAATATTTGTAGATTAGTTGACTTTTGCTTATATTTAAGTCCAAC

>Ca1:24592111-24592359

ATTTATATATTAAGATATTATCATGAAGGCGTACATTTTGAATTCGAGAGAACAGATCCGAGAGATAAGAGGGATGAGTAGTAAACTATTCTCATTTTCATTTAGAGTTTAGAGCACTAGGTTAACCGTATAATAGGATAACAATTAAATAGCTAGACGGTCGAGGGCAGAGACCTCTCGTTGAGCTTAACTAAATTATTCAATCCATCAAAGTCTATTCATAAATAATAACATAACACATATTCATC

>Ca1:24845353-24845811

GTTACGGTTATGAGAACGTTGTGCGAGGAAGAGTGGTTTGGTGAAGAATTGGTGTTTCTCATTCCCAGGGAGTTAAGATTAGAAGCAGAGGCCATTTTAATTTGTTACACTGAATTCTGTTTTTCTAGTGAAAAGGAAAAGAATGGAATAATGATGGCATGTAAAATGTGTGTGTGTTTGTGTATAGAGAGAGAGAGAGAGGAGGTGTTGAGGTTTGGTTGTTATGGTAAAGTTGGTGGAAGGCATTGGTGTGGTTCTTTTGGAATATGGGATTGAATAAACAGAACAAGTAATTGTGAGCAGGTTGTCTCTTTGGGACTGTCCTTGTCTTTTTCCCAAAATGTGCTCTATGCATCAAATTTCTTTTGTTGATTTGCTTCCTTCATATGTTTGGTCCCTTTTCATTTTTCCCTTTCTTTTGTTGATTTGCATCAAATTTCTCTTTACTTTCTTATAGA

>Ca1:25083428-25083722

CAACGAGATCTAGAGACAGATGACTGATGAGGTTGCGGCGAGTAGAGAGGCAGCGACGAATGGCCACGGACAGAGGCAGTCAGGCAGACGAACGATTGGCCGCAGCGAGCGATTTCGTGAGCGAGCAGACGAGCGATTATTGTAGAGAGACGACCGCACGTTTGTCTTGATTCATGAATCTGATTATGGAGGAGGGAGAGAGGAAGGCTGGAACTTGTAAGCAAAAGGGAGAGAGGAAGGCTGGATGGAAGAGAGAGGAAGAATGAGAAAACTGAATTAAGTTTAGGTATACTT

>Ca1:25083428-25083714

CAACGAGATCTAGAGACAGATGACTGATGAGGTTGCGGCGAGTAGAGAGGCAGCGACGAATGGCCACGGACAGAGGCAGTCAGGCAGACGAACGATTGGCCGCAGCGAGCGATTTCGTGAGCGAGCAGACGAGCGATTATTGTAGAGAGACGACCGCACGTTTGTCTTGATTCATGAATCTGATTATGGAGGAGGGAGAGAGGAAGGCTGGAACTTGTAAGCAAAAGGGAGAGAGGAAGGCTGGATGGAAGAGAGAGGAAGAATGAGAAAACTGAATTAAGTTTAG

>Ca1:25083447-25083714

ATGACTGATGAGGTTGCGGCGAGTAGAGAGGCAGCGACGAATGGCCACGGACAGAGGCAGTCAGGCAGACGAACGATTGGCCGCAGCGAGCGATTTCGTGAGCGAGCAGACGAGCGATTATTGTAGAGAGACGACCGCACGTTTGTCTTGATTCATGAATCTGATTATGGAGGAGGGAGAGAGGAAGGCTGGAACTTGTAAGCAAAAGGGAGAGAGGAAGGCTGGATGGAAGAGAGAGGAAGAATGAGAAAACTGAATTAAGTTTAG

>Ca1:25083902-25084339

TGAAACGTATGGTTACCTTGACCTCCCATTTTGTGTAACAGGTAACTGTCTTATTAATTTTTGCTTCAGCTTTTGTTATTCATTACTTCGATTAAACTTAAAGTTAGTATTATTAGTGTGATAACATGTTATATTTACACAGATTTGTTTCTAGTTTATTTAACTCTTGTTTAATAACAAGAGAAGACTGAAGCGCTTGGTGAGGTGTTGAATGGAGATCGCCTTGTTAGTGCTCCTTATGAACTTCACTTTAGAAACTAATGGATATGAATATTAACAAAGGCAATGAATAATGCTAATGAGAATAAAGAATGATAATGAATGATGATGTTACCTTTCAAACTCTCAACAGCAAATCTACACCAAGCGAGTTCTTTGACGGACAAGTCTAGCAATTTCAAACATGTTGAAATGGCGTTGGAGAAACAAAAGTGGTC

>Ca1:25694428-25694669

GACGGTCTTACCGGTTCGTACCCTACGGACGGGAATGGAGATGGGAACGTTTGACTGCAGTCACGTTAGGAGGATCTCACGGGGCGCGTGGAGATACTCAGGGTGTATAGCTTTTTGGTAGGATGATCAGATTAAGATGATGTATAGGGACTAGGTGTCCTTCTGTTTGGATTGGAGTATTTTTAGTTTGGGAAAACTGTATTTATACTATCATTGTCAGTTCGACTTCTTATGTGAATGG

>Ca1:26609923-26610144

CATTTTATTATGTTATTAAAGTTGTTAGCAACCTATAATGACTAAGTTGTTAGTGTTGTGCTAGAAAATGCTCTACAAAAAGCCACACTTCTCCTAAACCTCAAAAGGAGATATTGCAATTCTTAAACCTCAAAAGGAGATATTGCAATTCTTTTTCTAGCAAAGTACGAAGTGCTATAACGAAAGAAATTGGTGATGCCAAATTTATTTTTTTAGTTGAT

>Ca1:26610341-26610613

CCTTATGCTTATTATGTTTATTGTATGGCTCGTAGCAAATGGCACTGGTTATATCATCTAGAGAAGTTAAGCAAGTTCTAAAGATGTATGCTAAACCACGTGTTGTTAATGACCCATCTCTCATGCCGATGTCGACGATTGGTACTAGTGGTAGAGCTATTCCTCTATATCCTGAGGTCCCTATAGAGCCTTATACCATAGACGAAATAATGGAACCCGGATGTGTTGATTGCTACAACCGCATTGTCATCATTCCAAAAGGATATGAGTAT

>Ca1:26612189-26612596

GTCATCTTCACATCAACTTCCTCAACCTCAACCAGATCAAACTGCATACCGACCAACTATGGCAACAGTTGGCTTGATTAAACCAGATTTAAATCAACCTCAACCACCTATGGCTCCTCCTTCGACTAGTCGTGGTGTAATTGAAGATGAAATCCAATTTCAAACCATGATGTCTCCTCCAACACAAATTAATAAAAATTTACAGGGACTTCTGTCGTCGGGTAACATTGCAGGAAATAATGATGGTGGAAGTGGAGCTGATCAAAGGAATTATTATGAGGATTGATATGTATTTGAAGAATTTCATAATTTTATTTTTGGTTTGTATGAACGCATATTTATCTTGTCCTAGTTAATTATTGTGTGTTTAATTATTGGTTTGTAAGACATTATTTATTAATTTGGGA

>Ca1:26719938-26720562

AATTTAAAGCAGAGGAAGAAAATCAATACACACGTGACTCTTCTTTGGGTGCATGGCTTGGATCACATGAAGGACTATTTTATATGCACTGGTTATGAGTGTGAAAGGTCCTCTGATACTATAGTGCTTGTAGCTGCTGCTGGTCTTAGCATGAGGTGATTAAAGATTTAAGATTGTAGATATGCTGATTCGTTTAATTTCTTATTAAAGGTTTTTTAATTTGATTAGCGCTAGAACTTTGATATAGCAAGTAGCAGAGACGATAAATTACTTCATAGAAGTAATTATACTAGTTTTTAGACATGCACACATAATTTTGCTGCTATGGCTTTTTGGCACCAAGGTTTCTATGTAGATCATCTTGTTCGTGTAACTGTGAGATTCACGTTTCATCGTATACGGCCAGCCATGGTTGAACTGGAGACAACTGACGACTGTGTCATAACTGCATTAACATATTCAACAGCTCAGCATTATCCTGTCTTTGCTGAACAGATTGGTTGCGTAAATACAGTGGATTTCTGACTGTAGGGAATTCGGATTCTGTCTACTATATTGTCATATTTTCGCTTTGATAAATATCTTTTTCCTTTTAAATTTTGAGAACAAATGTTTTAAAAAAAG

>Ca1:26720166-26720562

CGCTAGAACTTTGATATAGCAAGTAGCAGAGACGATAAATTACTTCATAGAAGTAATTATACTAGTTTTTAGACATGCACACATAATTTTGCTGCTATGGCTTTTTGGCACCAAGGTTTCTATGTAGATCATCTTGTTCGTGTAACTGTGAGATTCACGTTTCATCGTATACGGCCAGCCATGGTTGAACTGGAGACAACTGACGACTGTGTCATAACTGCATTAACATATTCAACAGCTCAGCATTATCCTGTCTTTGCTGAACAGATTGGTTGCGTAAATACAGTGGATTTCTGACTGTAGGGAATTCGGATTCTGTCTACTATATTGTCATATTTTCGCTTTGATAAATATCTTTTTCCTTTTAAATTTTGAGAACAAATGTTTTAAAAAAAG

>Ca1:27794238-27794456

TCCGTCTTGCCTCCTGTTGGGTTTCTAAGAAAGCTAACAATTTAAAGGCGAACTTAAAGTTGAACTCAGCCTCGCCGAACTCAACCTCGCCGCCATCAAATCTCAATCTCGCAGCCACACCGCCATTGCTTCTCCACTTTGCCGTTGTCGCCTCCATCGCATCTCAACCTCTCTACGTCATATCTCAACTTCACCTCCATCGTAACCTCGTCGTTGTC

>Ca1:27795247-27795504

GTCTGTTCATGCAATTTTGTGGTTGTTTTGCATCCATGCCATCATAAACCAGTGGTGGTTGTCATGATTCTTCATGGTAGCAGAGTTCTTGGCATGTGTTGCATAGAGAGTATTGAGGCAAGAGGCTGTATGCAAACGATTCCAAATCCTTAAGCTTTGCAGCAAACTAATTCACATAACAGTGAAATCAACATATAACGCTTGTTTTTTATTTTTCGCACAACCTTTTATTAACACAGTTAATGTTCTAAATGCCA

>Ca1:27797871-27798092

ATCTGCTGTATTAGAGCTAAATTAAGAGAATAGCAATTGTTTTGTGTTGCTTGTCATGTTATAATAAGCTTGTCGATAATGGTTGTAGTCTTCCTATTATTTATTTTAACCAAAGTTCACCAGTTACCAGTTCATCGATAATGCTTCTTGTCTTCCAATTATTTATTGTAACCAAAGTTCACCAATTCAGATAGAAACAATTGTTTATCAAAATACTCCTA

>Ca1:27884411-27885317

AAAATAAATGGAGAGGAAAAGTCCAACTAAGAAAAAAAGGAAAAAAAAAAACAAAGAGAATGTTAGAGAGAAAGAGTACAAACGTGAGAGAGAGAGAGTGATGACAGAGGACTTCGTGTACAGTGGTGGCCTGCACCAGAGTGGTTCATAGAGGGGGGTCGTGGTGGTCGGTTAACAAAGGTGCGGACGCTCGATCGAAGATGGAAGTTGTGGTGGCATAAGGTCTGGAAACAGAAGCCGCGATCCTATCCCGACGACAGTAAAGTTCCTCCCTCTTCTTCTTTTTCTATAGTTTCTTTGATTTTTGTTGAATATTCTGATTTTAAGAGTTTTTCTGATGAGATACAAAAAACGGATTTATTTTTATGTTTTATTTTTCTAAGTTTTTGTTTTCATGGTGGAATGTATTTGTTGATGTATTTAAAGAAATAAAACCTATTGAGTTTCTAATTTTTTGTTCAGAAAAATCCCCTTCTTATGTTCTTTTTTGTTTTCTTTGTAACTCCCTTTTTCTTCTCATTTAATTAATTTTTTTGTCTCAATTTATAGACTAAAGTGGTGAATCTTCGTGTTCATTAGATAAAATTATGTTATTGATTTTTTTATTGTTTTGCAGCAAAAACAAGCAACCTCAAATAATTAAGGAAATAAATGACTTATCTTTTTTATTTTGATTTAGTGTTCAAATAGTGAAGATATAACCTTATTAGGATTAAAAAAAAAAACATATTAAAATGGGAAATGGAGTTGCTTGCAGACAAAAATGGAGGAAGGTGTAAGGAAAGTGCGGAAGGAGGAAAGGGCACTGGTGTACTTCTATTTGTTGTTTTTTTTTTCCGCTAAATATAATGTAATTGAAATCCATATAAATACATACTCAAAATTTGGATTATTTATAAAACGAAA

>Ca1:27884411-27884670

AAAATAAATGGAGAGGAAAAGTCCAACTAAGAAAAAAAGGAAAAAAAAAAACAAAGAGAATGTTAGAGAGAAAGAGTACAAACGTGAGAGAGAGAGAGTGATGACAGAGGACTTCGTGTACAGTGGTGGCCTGCACCAGAGTGGTTCATAGAGGGGGGTCGTGGTGGTCGGTTAACAAAGGTGCGGACGCTCGATCGAAGATGGAAGTTGTGGTGGCATAAGGTCTGGAAACAGAAGCCGCGATCCTATCCCGACGACA

>Ca1:27885027-27885317

CAAAAACAAGCAACCTCAAATAATTAAGGAAATAAATGACTTATCTTTTTTATTTTGATTTAGTGTTCAAATAGTGAAGATATAACCTTATTAGGATTAAAAAAAAAAACATATTAAAATGGGAAATGGAGTTGCTTGCAGACAAAAATGGAGGAAGGTGTAAGGAAAGTGCGGAAGGAGGAAAGGGCACTGGTGTACTTCTATTTGTTGTTTTTTTTTTCCGCTAAATATAATGTAATTGAAATCCATATAAATACATACTCAAAATTTGGATTATTTATAAAACGAAA

>Ca1:27884425-27884670

GGAAAAGTCCAACTAAGAAAAAAAGGAAAAAAAAAAACAAAGAGAATGTTAGAGAGAAAGAGTACAAACGTGAGAGAGAGAGAGTGATGACAGAGGACTTCGTGTACAGTGGTGGCCTGCACCAGAGTGGTTCATAGAGGGGGGTCGTGGTGGTCGGTTAACAAAGGTGCGGACGCTCGATCGAAGATGGAAGTTGTGGTGGCATAAGGTCTGGAAACAGAAGCCGCGATCCTATCCCGACGACA

>Ca1:27885090-27885317

TGTTCAAATAGTGAAGATATAACCTTATTAGGATTAAAAAAAAAAACATATTAAAATGGGAAATGGAGTTGCTTGCAGACAAAAATGGAGGAAGGTGTAAGGAAAGTGCGGAAGGAGGAAAGGGCACTGGTGTACTTCTATTTGTTGTTTTTTTTTTCCGCTAAATATAATGTAATTGAAATCCATATAAATACATACTCAAAATTTGGATTATTTATAAAACGAAA

>Ca1:28033490-28033708

ATATGCATCTCTAGCTTCAAGCACTATTGAAGGTACATGGTAGAATGGTAGGAAGAAAGCGATATTGGAGCTACAATTTCTTCAGTTATAGTACTAGTTTTGTAAAAGAAATGTATGGGCTTGATGTGTTCAATTAGTTTTCTTGGAAAACATTTGAGGTGACTCACTTCTGAATTAATGTTTTGTTTTATAACTCTACTCCTGTTTCACTCTGTAAG

>Ca1:28323205-28324094

CCTTCCACTGTTCCCTCTCACTCCAAAAGTCAATAACCGTGTTATCTTAGTGCTTGATTACTGATAAGACATATTCTAAGCTCGAGAAATGAGCAATGAGTGTTTTTGAAGACAATGACCCCACATGTGCTGTTCTTAGATGCCAACAATGCCATCATCAATGAATATTCAATACCCATTTATTATTTAGGAGTACCTTACCATACCCCAAAAAGTTTCATTTTATTTTTTTATTTTATTTAAACTTCTTACAGGAAGAAGGAGGAGGAGTCAATGCTTCACTTTGCTTATGTCTCATTCACAGAGAGAGAAAGAGAAAGAGTCTTTTAACCACACTGTTACTCATTTTGTACCGTTGTGAGTTGTGAATTGTTTTATTTTGGGTTGGTTTGTTAGTTGTGAAAGGTGAATTTTTTATTTCTTTTTGTTTTGGGAATTTGGAATAGCACCAAACTCGGTTGTTGACCTAAACTTGGTTGATAGATAGATAGATACATAAGATAACCAATTTTATGTTTTTAGGGTTTTATTTGTGGTGGATTTTGCAAATGGGTCTTTTTGTGTTGACAATGGAGTAGTTTTAGGGTTTTCAAGTGGTGTTGTTGAATCTGGTTTTGGATTTGGTTTTGTTGGGTTGCTTGGAGTTTCTCTAAAGGTTGTGTCTTTGGGGGAAGAATTGAAAGAAGCTACATTGTTAGATACAACACTGAATTTTGAGTCCAAGTCTTTTCGTAATTTGGAAGGCTTTGTTTGGGTGTTTCAGAAGGTTTTTAATTGACCTTAGATCTGTTCTTAACTGTGTTTTTGTTAAGTTATTGGGATTTGAGTTAATGGCTTGCATGAAATTGGGTTCCAAAGCTGATGCATTTCAGCGTCAAGGCCAAGCATG

>Ca1:28800664-28800959

GTCTAACTTCACCACATTGTATATTTTGAATCTTGAGATCACAACCAGGACTGAGTTATGCAGCATAGACATTCAATGTTGAGGATGAACCTGACCAGAACTGGAATCATGTATTCTCTACATTTCATGCACCTTGCGCTGTTGGACTTGAACTACTAGGGACTTGCTAGTTTTTATTTTATCACGAAAAGTGTAACTTGAATGGCAAATTACAATAGTTTTGGTCGAATATTGACCTGGAGAATTCATCGAACAAATTGTGGCAATTTCTTTTCTGCTTGCTACAAACCGTGAG

>Ca1:31172461-31173154

GTTTTTAGTTCGATTTCTGAGTTCCTCTTCAGCTGCGGAAACTAATTTGCCTTAGGCTAGCAAAATTGATACGGGTGTCTTCACCTCTGAACTATGATTCTTATGTTTCAATCTTTGATTCATTGTTTAGATTCGGATGTGTAGATACCCGCATCTGTTATGCTAGCTGTCTTTTTCTATGTTCCTCTTATTCGATTAAAGGAAGTATCCAACAATATTTTCCCTTTAAATTTTATGCTCTTGTGATTGTTTTTTTTTTAAGTTTTGTATAATTATATGAATATATGTAATTTTCCAAAATTTGTTCGTCTCAGATCTAACATTTTGAATCGGATTTAATGTTTGGATTTATGATTTATGAAATCGAAGAAATGTAAGGATATTATTATATATTAGCATGCAAAAAGTCAAATAGATTGTGAATCTATGTATACATCTTTTATCTAATTTTCTGAATTAATTGTTTTAATACAAAAACTTCCAAGTGTCACTCTTTGAAAATTTATATTTCCATTTCTCCTTGATTATTTGGTCTCACATTTCAATCCGAGGTTCTAATATTTCGATCTTCAAAGCTATGGTCTGGATCACATACACCATAGATCTTTGAATCTTTAGTGGTCAACTATAGAAAAGTACATAAGTTGTTCTAGGTTATTACTGTATTTTGTGCACGAGGAAACTGAAACTTGC

>Ca1:34301814-34302027

CTTAAAATAGGGGATGTAAGAGTAAGAAGTGGTAAGAGAAAGAGGTCGCATAGAAGGGGGGAACGCAAAGAACAGAGTCAGCGTCGTTGCATTTGCAATCATTAATCATACTTAATTATTATTACAAGTGAACGACGCCGTTGCATTCCCATGGCAGAGGATGGAAAGCCCGATGCTCAGCTCTTTCAGCTTCTCTCCAATCTTCTCCTTGAG

>Ca1:37356880-37357319

CATGAATAGAAGAACAGAATGTGGATGAACTTTAGGATAGCGCGTGTATGGATTGAAGAAGGGTAAGAAAGATAGAAGGATCCGCTCAATGCAAACATGACAGTCCATCAGCATGAGGAAATTATGTTTACTGAGCATCATTTATGTGGAGTAATTGATGTCATCAGTGAACACATGATCGAAGTTTACTTTCATGTTGAATATGTTTGTTGGTTTAATTCATTAAGATGGGAGAAGGTAGCTTTTTTCTTTTCACTGAGTGAGTAGTAGTCTCATGCGGATATATAAATTAGTAACCTAATACTTTTATTTAAATTAGTGCTGTGTGTTTACCCAGTTACTCCGAATCTCTCAAAATCATTTTTTATACATTTGGAGTTGCGTCTGATGTCTGTTGTATGAAAACTGATAACTTCATATTATTCTGACATATAAAGCA

>Ca1:38285017-38285453

GTATCCACTCATAAAAATGGTTGATATTTTGAGAAAGGGAACATGTAAAGAGAAAATAAAAGGGGTGAGAAAAAGAAAAAGAGGCATGTGTATGGGCATTAAAAGCCACGAATATGTAAGATAGGCAAAAAATTCAGTGTAGGTCAGCCTTGAGAGAAAGCTATGCTTAAATGGGGGTATCAAGAATAAAGCCAAACAAAGTGTTCTAGTGGAATGTCAATGATCAAAGGGATCATATTCCATCTATGCATTATGGTTCATTGCAAGATCGATTCATTAAGAGAACAGATAACAGATTAGAGTATTAAGCAGAGCAGTGTTATCTGTAGCAGAAGTTCTAAATCAAGCTGCACTTATTATGTACTAGATTGAGAGATTGCATTTCACCTATGCTGTATAAGCTTAGTTGATTATATGGTGTCCAACTATTTGAGTG

>Ca1:39600859-39601867

CTTAATTTTAAAATTATGATTGGACGGATGCATAAAACCAAATTTCTGTTTAATATATAAACTAAATTCTTATAAATAAAAAAAGGATTAATTTAACAATTTAAGAATTATGAACTGACAGTTCAAAAATTAAATAACTAGACACAAAAACTTTAGTATTTCTCTTCAAAAAATTCTAAAATCTTTCATCAGTGTGCCCTTCTAATTGTCCACCAAAATTGATTTTCAATCTAAATCCTCCTTGCAGAAGCTCTCAACAAGCTCTACTCACACAACAATCTTCGCGTAACACTCCTTGCGGAAACTCTTTCATTCTCACGCAAAACAATCTTCACTGTCTTCTCTCTCAAGAACCCTCTCGACATCGCTCGACCTGAATGAAAAAGGGGAAAAACATGATTATGGTTTCTGTGTCTAGTACAGTTTTCTCGTGATTTTCCCCCTTTTCTCGCAACTCTACATCGATTAGGGTTTTGGCATCTTGCGGGACTTGCAAAAGTTTTACCTCTTCGTGACTTGGTGAGCTTGGTATGTATTTTCATCGTTGCTTTATTATCGCTCAACCTTTGCATCGTTTGCATTACTCTAAAAATAACTCTATAATCATCTTCAAATGATTGATTTTGGTATCGCTCTTTATTCTATTTCCTATTTGCTGTTTCTTAATCTATGACATCTTGTGGATTCTAAAATATGAGAAATTCATCTCTCTAATCTTAGAAACACTTTGAAAACTTGAAGCATTAAGGATGGATATATGGTTATTTCCGTTGATTGTTCATATAACTTGAGATATTGGTTGTTTATTTTAATCTCTCTGCACTATGTTAATTAGTGAGAAGAACCATCATAGTTAGAGCATTAGTATCCTTAATCTGCATATGAAGTGTTTGCAAAATGTCCTAAGTAAGCAGAGGTACTTTATTATGCATGAAGGTGGTAAATAGTAGCATTGCAGAAATGAACTTTTGCATCTCAACATTGTTAGATGCTCTTTGATAAAACGAG

>Ca1:40136258-40136462

TTTTCTTCCTGAGACTACAAACTCTGTTTTACATGGCAAATATAACCGATTCTTATTTTCTACGCTCTCGCTCGTTTACTTTACCTGAAGTCACTGAATAAAACAAGTACTTTTTAGTTCATCCTCACTGGTTACAGATTGTGTTGGCAGTTGCAAAGGTTTCTCACTACTCTTCGGCTTCGCACACAAAGAACCAAATAAAAG

>Ca1:40452114-40452578

GAACAGAGCAAAACCTACACACTCACACTTAAGCATATGTTAATAAATAAATAAATAAAAACCACACACCACTTTTCCCCTTCTTCTTTTCTTTGACTCATTCCTTTAGGTGGTGAGGTGGTTGACTAGCACCCAAACTCCATTACTACAACTCACAATATCCTTCTCTTTCATCTTTCTTCTTCCATTCATAATCAATCCCATAACACCACTTCCTTCTTTGTTCCTTCCCATTTTCAACAAAACTCTTCAATCTATTCCTTTACTCTTTTTTCTCTCTCAATAAAACACTCATCAATAGCCCCATCTTTGGTTCTTCATCCTTGCTCAAATCTATGGTAAAAGTATAGTTCTAAGTCATTTTTGGTGCATGCATTATGAGGAGGTGGAGGGCAAACTAACAAATGTCAAGGATTCAATAAGAATAAAGTCTAGGATTTTATTTTTGGTGGTCTTTTGAAAAG

>Ca1:40453263-40453673

GAAGGATGTGATAACTCAATTTATGAGAGAACTCTACTGTAAAGTCTTTTGATAAGACACATTATTTAAGTGCTAACCAAGGGACCTAAGTGTCAACGTTCGTCACTATTCAAACTCTACAAATTGTGCAATATGGAGTATGTGTCGTGCTTGAGAGACGCTTCAAATAGGGGTTACAAAGCTGTAATCCTAACCATACTAACGATGGGAAAAACTTAATCACCTATATTACTAACATTATAGTATTTAAAAATCAAAATACTATATTATGAATGTTATATACTCTATTTGGTATGTTTGAGTTGCTTTGAAAATTAAGTCTCACTGAGATTTTCATCTCATTACTTTGTTCTCTAAGTTTCAAGAATCATTACAAGAATGATAGTATCTTGATGATCTATTTCTTTTGG

>Ca1:40745432-40746160

CTTTATTAAAATTACAAAATTACAATAATGCCTAAACTCTAAATCATATCCTCTCTCTCATGCGCACGCTGCTCTCTCTCACTAGAAGATCAGAAATTGCCGCTAAGCTTTTCTTTTAGCTCTAACTCATCCTGGGCGATTCTTATTATTCTTGGCCACTAAGGTGGCTTTTGACCAAGAGAACCAGCTAGAAGAAAAGGTACCACCGAAAGAAGGTCGACCTCACCCCCTTTGCTAAACCGAAGCAGAGTAAGAGGAAGAAGCAAAGCTAGGCCATTTGTTCTCACCTGTGTGTACAATCCTCAAGAAGGAATATACAAAAGAACCTTATTCTTTTTTGCATCTCCCAAGTTCGAATGCTTTTATGCTGTTAACTCTTGCTGCATTGCGTCGCGCCGCCGCTGTGTCGCCCTTGTCTTTCCGTCTTGCGCCGCTGCTGCCTCGCCTCGCCTGTTTGTCGTCAAAACTCCAAAGGTTTGGAATTTGTTGATTTGCTGTTTTATCTGATTTTGTTGATTTGCTGAACAAGATGTTTGTGCTGATTTGTTGTTTTTCTTGATTTTGCTGAACAAGATGTTGTGCTGATTTTTGTTGTTGATTTGCTGGTTTTTGTTGCTGATTTAAAATATTGGCTGCTTATTTAGAATGATGGCTAAATTGGTGTTGCTAAATTGGTTGATTTAAATTGTTGTTGCCGATGGCTGCCTGTTTATGGAACAAGATATACG

>Ca1:41132336-41132738

CTCTATTCCAGCCTTCCTCTCTCCCTTCTCCAGATTCAGATTCACGAATCAGTACAAACGCGCGGCCGCCTCACTCTGCCTCCAGTACAGATCGCTCATCTGCTCGTGGCGGGCCTGCGACCGCCTCTGCCTGACTGCCTCTGTGGCCGTTCTTCGCTCTCTGTTCGCTGCAACCTCATCCTTCTCCATTTCGCGTTGTTGTTTTGTTCAAAAGGTTCAGATTTCTATTTTGTTAGTGCTGATTGTTGTTAGTTAGTGTTGATTTTTGTTAGTTAGTGTTGATTTTTGTTAGTTAGTTTTGATTTGGAATTCTGTTAGTTTTGAATCGTCGGTCCTTTGTTAGTTAGTTTTGATAGTTCGCCGCTCCTCTGACTCTGTTAATTTCAATTTTTCTTAGTTCGT

>Ca1:41504751-41505181

GTTGAGCCCAACACCCTGGCAAGCCATCAAAACCAAGGTTAAATCAAAACAGCCTGGCAAACAAGTTGACATAAAGCAAAGATATTTGAAGATATGGTATGAAAGGAACCGTAGCTGAGCAAGAAAAATCCCATTTTAAAGCTTTGTTAACATAACAAAAAAAATGATTTCATCCAAAAAAAACAAACATTCACACAACTAAAACAAAACTCCATCTTCCTAGTTTTAAATTTTAACTTCCAATCTAAGCAGCAAATATGAAGATAATAAGAAGATACATGCCTTTTCAGATACTGATGAAGACAGAGACGGTGGTTCGCGGTGGAAGTTGAAACGGCGGTGATGCTTTCGGTTTCTTCTTCTCCTCTTTCTTTTTATGTGTTTTTTATCAGTATTAATCTCCGATTTCTCCCCCTCAAAAAAAATGTCT

>Ca1:41823949-41824355

GTCCAAACACTCTAAGTGTGCTTTCTGGATTTCAGTCAGTGTCTACTGTGAATCCCACAATCAAGTGAACACTAGTTGGACTCCTTTCACTTTGATATACCATCATTTGTTATCTTTTGTATTCCTTTTCAATAACCTTTTATATCTCCTGATTTTACTTATTTAGTTTCACAGCTTAAGCAGCATCTTATATCATTTATCTGAGTTTGGCATGATTATTCAGGATTGCTGAGAGATTCTTGGTTCGCAAAATAGTTATCTAGTTACTTTTGTTAGGAAACAAGCCAAACTAGTGGCTCATTTGCTGTCTAGGGCTTCTATACTCTTACCTTGTTGTCATGATCTACATTATATTCCTAGTTTTATTACTGATATATTTTATAGTGAAATGCAATAAGCTTTCTGC

>Ca1:41983715-41984064

ATTTTTTATATATTCAAAAATTTAAATGATATTTATTGAGGAGATAATATTTATTTGAATGTTTTTAAGTATTTATTAAATATTATTGCCAAAGATTCATAAAATAATGTGGTCTAGCACTTGTTAAAATAAACCCTTTTTCAGGTTGATGATGATGAGAAGCGTGCGTATTTGATCAGAAGGGTGCAGAAGAATTAATGAATAATTAAATTGTAGAGATCAAAGGGTTTTCATAGTGATTCAAAGGGGTATTATCTTATCTGGTTAGGCGCGGATCTCGATAGAAAATTTAATCCTACGGCTTGAATTGTGCGGCTAGGTCGACCGAATGAAGAGAGAGGGTCGTCGT

>Ca1:41984197-41984527

GCGGCACCGACGAAGAAAACGCTGCGTAGAAAGAGAGACCAAATCAGAAGCGGGCGTGTTTGGAGGTGGGCCCCAAACGAGCATTCCGTGTTGACTATGTGCCTGTCGGAGTTCATGTTGTCTTCACGTGATGTTTCAGCGCCCCCATCTTTCTTCTGTCTACACCTTTTCCCACTCCTCTTTCTTTCCCACTTCCAAGTGGAGTACGTATAACATTTTTATTATTACAATAATACAATTATTTTCAAAGTAAACAATGCGTCCTCCCTGAATGCTTTAGAATTGATGCGTATAAAACATATATTTAATCTGAAGTTAATTAAGTTGGAG

>Ca1:41996641-41996974

CCGACAAGGGGCAATGATTGAGGGGCACATTGGAGTGATTATCATGGGAATCTGATACGGAAGCAGAAGCGGTGGTGATGACTGATGATATGTGTGAAGAGAACAGACAGACACATTAAGTGGGGAAGTCGGCGCGTCGACGTTGGACTGAAGATACAAGGAAGTGCCAGGTGCTGTCTAATACTACTCATTTGTTTTTTTGTGATGTGAGTGACCACTTTTGATTCAAAACCTTTCTGCCTGTCTAAATGTTCTAATTCAATAGTGTGTTGACCCAAAACCCAAATCTATAAAACTGAAAATGAATCTGTCCAACTTAAAATTATTTCGAGA

>Ca1:42284683-42284951

TTTTAAGTAAAACCTAAAAGACGAAAACTCAGCGGCTCCAAAAACACAGTGTATTTGAGTCACATCGACTGAACTCAACTCAACTTCCCGCAAAACGCAGGATTTCATCGCCGGCCGCTAATTATTAAGAAATTTCTTCTTCGAACCCAATCCCCCAAATCGATTTCACCTTACTCTGCCATTCTCTCCGGCGGCGGCGGCGCAACTCCTCCGGCGTTCTTTCTCTGTGCGCGACTATTCTCTTTACGCTTTATCTTTCTATACAAAG

>Ca1:42289526-42290416

GCTTGGGCTGTTTTCTCTTGGACTGCTGTAGGCCAAGATAATTCAATGGGAATTTTGGTGCTACTCTGATTCTTCGTCTATGGCATTGCCATGGGACTAGAGTTCAACTAGGATAGCTGTAGGACTAGTTTGACTTCTCTGGGATTGTGAGGCCAAGTTGCATCTCGGATAGTCGTAAGGTCAAGTTACGTATGTGACAAGATTGCTGGATGGGTCACAAAAGGTTTCAAAGGCTCTGGGTGAGGTGAGAGGAAAGAGAAACATCACTGAATAAGGGTGGTTTAAGGAAGACAGTGTGGCGTGTCGTGTAAAAAAAATTGCCAAACAAAGACAGATGTGAGGAAGGGAAACTTTGGCAACCTGGCCATGTAGCAGAAACAGTCAGGATCCCGTGGGAAGATGTCCAACCATATACCAGATTAAAGAATCCAAGGCAGATCCTTGTAGTCCACATGCCATGTGAGGTGTAGTTTGTGATGCACAGGAAAATAAGTACCTTGAAGAAACAGTGCGTATTAATGCGTGGCTTGCAGATTTTTGATGAAATTTTAGGAGTGTGGAGATCAGCTCCCTGCAAGTCTGTCGATGGTGGCTTTGGTAAGAATATTCATTGGAAAAATCTTCCAGTGATGGCAAGCAATGACTTGAAAAGTAAACTAATCAATCTTGCTAGAAAGATGACACAAAAAGCAGACTTTAAAAACTGGAGATGAAACACAGTTGAAAAAGCTGGCTAGAATTTGTGCACAGTGAACAAAAAGAAAATAAGCATAGAGATAATGGTATTATTATAATGGAGTTCAAGACTTCCGATAACTCAGCAATTGAACAATGACGGAATAGGTTAGAGGATGGTTCTGATTTGATACAATGTTGAAGTTATATGGTTG

>Ca1:42291141-42291487

ACGTGTCATGACTTTACGCCGGATCAAATAATGTAGCTGTTGACAACAACCCCTCTTCATATATCCCAGGGTTATTCACATCAATGTTTATATCCATGAAGAGGAGATTTGCCAAAATTTTCTGCACTATCGGTATATAAATCATAAAGCAGCGGGGTGTGCATTCAAGTTATAGTTGTTATTGTTATTGAGTAATTTAAACTTATAGCTTCCATATATCCCAGGGTTATTCTTTTAGCTTCCATAAGAGACAGAGAGAAGTTTATGTCATTGATAGACAACATTAACATTCAAATATATAATTTTAGACAAAACTGGTTTGAAGGTCAGCATATCTCATTTAACG

>Ca1:42421368-42421872

CCTGGGTTCTGGAGGGAACAGCTTGTGCGTATTTTTCTGCCGTATTTCTTCTGCGGCAATGAAGTACCAACCAAAATCGGAGAAATTCCATTGATGGGTTGAAGCTGATGAAATGATATAACAAAACAAACTTACATAATTGATGATAAACAACAGAACAATCCAAGTAAAGCAACTACATTTTCCTTTTTTTTCTTTCATTTATGAATAATAATAATTTTAAAGACACCAAACTTGCGCACCACAATTGAACACGTATAACCACCAAAACTGTGTTTGTCGTTTTCCTTCGAAAAAATAAGAAGAGAGAGAGAGAGTAAAGGGGGTGTAGTAAGAATTAAGAAAGGGGTAAAAGTGAAACTGAAAGAAAAAGACAGTAATAATATATAAACTTTATGAGATAAATCTGAGTACCCAGAAAGGGTTTATCGTTTCACAATAAGAAGGAACCCAAAATCTCTCATCTCATTGCAACTACAAATCCTCACTTGTTGTTTTGAAAAG

>Ca1:43604888-43605183

CATGATACTTAGCATTTAGCTTTTGGAGGAGTGCCTAATGTAGACAAGAAGGAACTATTAAAACACTTTTAAAGACAAAGTCCCCACTGACAAGAAAGTGTGATAGAAAAGATGAAGAATAGCAAAGGCATAATCATTTTACCCAAGATGGGGGGAGTTGGGTTTGTGACCACAAACTCCAAAAGTCCATTAATGGGATGAGTAAGCCTAGGTGGACCTTGTCACATGGAAATTATGTTAGTGGGGAAAGGTGATTGTTACACTTGCACACACACAACTATTTGTCTAAAGGCAA

>Ca1:43638848-43639327

GAAATAATGAGTGTGGGAAAAAAAAAAGAGAAGAAGGGATAGAAAAAGAGAGAAGAGACAGGAAAAAAGAAGGAAGAAAAAAAATGGTGAGAGAGAGTGTGAGAGGATCTCGGAGAGTGAGAGGATCCCTGAGAGGGAGACGAAGGAGAAAGAACCCATGAGTGGGTGAATAAGATGAATATGAAAGAGAAAGATGTAGAGCTCCAACAAGAAAAAGAGGAAAAACTCAAAACAAAACTATGACACTCTAATCTCTTATGTAATTTCTCTAAGGTAACATAGAAAACTCTCTTAAAACCTCTCTTAGGTGTGCTTAAATATTTTAATTGCATATTGCTAATAATTGTTCATGATTTGCATGTTTGAGTATTGTGATAATTCTATTTTATTTAGTATCATTGATTATTAAGAATGCATGCTAGGTGTTTGTATTAATTACTATGAATAGATGTTGTTTTATTATAATTGTTGAGTGTTGT

>Ca1:43638848-43639121

GAAATAATGAGTGTGGGAAAAAAAAAAGAGAAGAAGGGATAGAAAAAGAGAGAAGAGACAGGAAAAAAGAAGGAAGAAAAAAAATGGTGAGAGAGAGTGTGAGAGGATCTCGGAGAGTGAGAGGATCCCTGAGAGGGAGACGAAGGAGAAAGAACCCATGAGTGGGTGAATAAGATGAATATGAAAGAGAAAGATGTAGAGCTCCAACAAGAAAAAGAGGAAAAACTCAAAACAAAACTATGACACTCTAATCTCTTATGTAATTTCTCTAAG

>Ca1:44074075-44074544

NNAAAATTTCATAGTTCATTGCCTATCCCTAAGGGATTGGATAGAGTAAGCATTTTGTAGAAGAAAACTGGAATGGATTGCTGAGATCAAAACTATAGAAGGAATGGCTAGCAGCCTAGCAACACTCAGAACAAGATTTGCAGTACCATATGAAAGATAGATGAGTCATGATGAAAAAGCCTTGTGATTAATTATTAGAGCCAAGGTTGCCAACTTTCTTTACCACATTTCATATCATAAATATATTTATACACTAAGCTATTAAAAGTGAAAAAACCTTGGTTTCTATGAGTTTCTCACATTGCTCATATGGTCCTTTGAGGTTTTTAGAATTGAGCATGAAGCTGGTTGACATTGATATGTGGCCTATTGTATCATTGTTCTTATATTAGGATTTAGAAAATGCTATTTTCAATGGTTACTAAGGATTACTTTTGGTCCTTTGTACATACATGCCTAGTAATAAAAG

>Ca1:45127835-45128771

ATCTTCTTCTTCCCACACACCAACAACAACACACATCCCCATGACACTTTTTCTTTTAAACTTCTCTCTTCTTCCACAATCTCAATAGTGCATCGTTTGCAACACTAGGAGTATATTCGGGACCCTTGTAACCTCCTCTTTCATTCCCACTAAGAATCAAAACTCGAAAGGTTATATTTGTCGTACCGAAAAAACAAACCACCAACTTCATACCGACATTCCGGACACAACCAATGTTGTTAGTGAAAACCGAGACCGCCTAAGAAATCCACACAACACCTTATTTCATCTTCTATGTACGAAAACACCAGCCATTACACTTTGAAAAATGACATTTTGCCACTGATGCGGTTCTGTTTAGTTCTGATCAGCTCGTTCGTCGTTTTTGAAAAACCTACTTTGTTTTTGGAATATTTGCGCCGAAACCTTTGAGACGAGAAGAACTATTAGTTTGCGCGTCATTCGAAAGTTGACGTCGAACCATTTCGAGGTTTTGGTGAGACCTTTCGTTGACTTTTGTTTTGTGTCGTTTTTGTCGTTGGTGCTGAAAGTCTCCCTTGAGTTTGGAGGTTGTATGGAAGCTTTGGAATGAGGAGCAAAACCCACTCGAGGTGGTAAAGCTCGATTTGATTTAAAGTTTGAACTCTATTGATTGTAATAACTGATATAGTACTAGTGAGACAGGTGAGAATGTCTCATTTCCGTATGTATTTTGGGGTGAAGACAAATGTCGAGTCATATAGAGTGTAACAATGAAATAGGTTTTTGTAAGATTTTGATTTTTGTTAGGATTTTGAATTTGATTGTTTTAATTTATACCTCTTTTTATATGAGATTTTATGCTCTGATTAGTGTTTAGTTGATCTATGGGGCGTACTGCAATTTATCTATATTTTTAATTTGAATTTGAGTGTTAACGTTTGGATTTAAGCTGTG

>Ca1:45127835-45128464

ATCTTCTTCTTCCCACACACCAACAACAACACACATCCCCATGACACTTTTTCTTTTAAACTTCTCTCTTCTTCCACAATCTCAATAGTGCATCGTTTGCAACACTAGGAGTATATTCGGGACCCTTGTAACCTCCTCTTTCATTCCCACTAAGAATCAAAACTCGAAAGGTTATATTTGTCGTACCGAAAAAACAAACCACCAACTTCATACCGACATTCCGGACACAACCAATGTTGTTAGTGAAAACCGAGACCGCCTAAGAAATCCACACAACACCTTATTTCATCTTCTATGTACGAAAACACCAGCCATTACACTTTGAAAAATGACATTTTGCCACTGATGCGGTTCTGTTTAGTTCTGATCAGCTCGTTCGTCGTTTTTGAAAAACCTACTTTGTTTTTGGAATATTTGCGCCGAAACCTTTGAGACGAGAAGAACTATTAGTTTGCGCGTCATTCGAAAGTTGACGTCGAACCATTTCGAGGTTTTGGTGAGACCTTTCGTTGACTTTTGTTTTGTGTCGTTTTTGTCGTTGGTGCTGAAAGTCTCCCTTGAGTTTGGAGGTTGTATGGAAGCTTTGGAATGAGGAGCAAAACCCACTCGAGGTGGTAAAGCTCGATTTG

>Ca1:45127835-45128519

ATCTTCTTCTTCCCACACACCAACAACAACACACATCCCCATGACACTTTTTCTTTTAAACTTCTCTCTTCTTCCACAATCTCAATAGTGCATCGTTTGCAACACTAGGAGTATATTCGGGACCCTTGTAACCTCCTCTTTCATTCCCACTAAGAATCAAAACTCGAAAGGTTATATTTGTCGTACCGAAAAAACAAACCACCAACTTCATACCGACATTCCGGACACAACCAATGTTGTTAGTGAAAACCGAGACCGCCTAAGAAATCCACACAACACCTTATTTCATCTTCTATGTACGAAAACACCAGCCATTACACTTTGAAAAATGACATTTTGCCACTGATGCGGTTCTGTTTAGTTCTGATCAGCTCGTTCGTCGTTTTTGAAAAACCTACTTTGTTTTTGGAATATTTGCGCCGAAACCTTTGAGACGAGAAGAACTATTAGTTTGCGCGTCATTCGAAAGTTGACGTCGAACCATTTCGAGGTTTTGGTGAGACCTTTCGTTGACTTTTGTTTTGTGTCGTTTTTGTCGTTGGTGCTGAAAGTCTCCCTTGAGTTTGGAGGTTGTATGGAAGCTTTGGAATGAGGAGCAAAACCCACTCGAGGTGGTAAAGCTCGATTTGATTTAAAGTTTGAACTCTATTGATTGTAATAACTGATATAGTACTAGTGAGACAG

>Ca1:46462153-46462472

CTTTTATAATCAAACTATAAATAATACCTAAACATAACCCTCCTACTCATGCATTCTAATTTCAAAACCCTTATAATAATAAACGCACTAATTACGCCATCTAAACTAACTCAAAGCCTCTTCTCCATTTCAGATGCCAAAAGCAAAATATTGAACGACCCAACAAGCTTTGTTCTGTTCTTTTACCTTCACCTTTCTTCCATAACACTCTTTGTTCCCTCACACTCAATGGCCATCCCATGCGAGCAAACAAAACCCTTCTTCGCGTGTATCACGCAATCCAGTATCATCATCATAAACAGAGAAAAACAATTTTACA

>Ca1:46869780-46870236

ATTTGTCGTACCGAAAAAACAAACCACCAACTTCATACCGACATCCCGGCCACAACCAATGTTGTTAGTGAAAACCGAAACCGCCTAAGAAATCCACACAACACCTTCTTTCATCTTCTATGTACGAAAACACCAGCCATTACACTTTGAAAAATGACATTTTGGCACTGATGCGGTTCTGTTTAGTTATGATCAGCTCGTTCGTCGTTTTTGAAAAACCTACTTTGTTTTTGTAATATTTGCGCCGAAACCTTTGAGACGAGAAGAACTATTAGTCTGCGCGTCATTCCAAAGTTGACGTGGAACTATTTCGAGGTTTTGGTGAGACCTTTCGTTGACGTTTGTTTTGTGTCGTTTTTGTCGTTGGTGCTGAAAGTCTCCCTTGAGTTTGGAGGTTGTATGGAAGCTTTGGAATGAGGAGCAAAACCCACTCGAGGTGGTAAAGCTCGATTTGAG

>Ca1:47060211-47060719

ACAACATAAAAAAAGATAGAAAAAAAAAAGAAACATCTACAAAATGTAGAACTTTGTCTGTATCACACCACCACACTTCTATTCTCTAGTCTTCAATAATCCTAATAATACAACCCATAATATTTGAGATAGATGTAAAGAAACTAAAGAATAACAATTAGAAGAAAGGGAAAAGGAAAAATTTGTAACCACCACCCACTGTATCAACCAACGTGGAATCAGCTAGAGCCACCACCATCTTTGACCAACCGTGTACACCTTCGTCAAACCTAGCCATCGCCGATCACCTCAAGCTCCGCTCTTGTTGTCGTGCAACAGTCATTGCCTTCACCAAGTTACCGCCCACGACCTCCATGGCTACACCCCAATTGGTTTCTTTCCTCATTTTATCTTCTTTTACTTCACTTTCTTTAAAAGCCATATCTCTCTCAACAGCACACAACCGCACTACTTCTTCTGTCATTCATAATGATGTATTCTTATGGTTTTCTTTTACCATTCCAGTTTG

>Ca1:47189590-47190196

TTTTCACTTATCTCTTTAGATTGCAACTCTAAACAAGAAACCCTTCTTCTTCTTTGAGCCACCGCTCCGGCCACAACGATTCAGATCCGGCTTCGACGTTTGTTCTCTCTCATTCACATGTTCTCTCTCTCGTTCTGTCTCGTTCGCTCTCTCTCTCTCTCGTTCTGTCTAGTTCTCTCTCTCGGTCTCTCGTTCTGTCTCATTCGCTCTCTCTCACTCTCTCTCTCTTTCTCTCGCTCATTCTGGTTTGCTCTCTCTAATTTGTTCTATTTTTAGGATTATTGGTTGAGTAAAGGAAGCAAGAAATGATTTCTTAATTGATTTGCAAATCCTCTTTTCTTTTTCTCAGCTCACTGAAATCCCTAACATCTCAAATTCATTGTTGCATTCTAGAACACATTCTTCTTCACTTGTAAGAACTTGAAAATCCCTAACATCACCAATAGTGACGTAATGTGTATCGGGGCTTGTCTTTTGTGATTTCTTCAACCACGTCGACCTGGACAGAGCACTCTGTTAGTTGTGACCAAACTGAAATCAGTTCCCTTCGTGATTCCTTTCTTTGTTGTTGATCTTGTTTCCTTTTTTTTTTTACCGTTGCTAGAG

>Ca1:47193197-47193415

TTTTTGTGTGACATTTGCACTATCAATTGAAACTTCATGAATTGTGGTACTATTGAAGATGAAGGTATCAGAGTTGACATTTGTACTATCAATTGAAACTTCATGGTTAATGTTGTGAACAATTTGGTTAATGAAAACTTGACATTGTAATCAAACAAGATACAATTAATTCCTTGCAAAACTTGGTTAATAGATTTAAATAGAATATTCATTCTTAT

>Ca1:47189592-47190196

TTCACTTATCTCTTTAGATTGCAACTCTAAACAAGAAACCCTTCTTCTTCTTTGAGCCACCGCTCCGGCCACAACGATTCAGATCCGGCTTCGACGTTTGTTCTCTCTCATTCACATGTTCTCTCTCTCGTTCTGTCTCGTTCGCTCTCTCTCTCTCTCGTTCTGTCTAGTTCTCTCTCTCGGTCTCTCGTTCTGTCTCATTCGCTCTCTCTCACTCTCTCTCTCTTTCTCTCGCTCATTCTGGTTTGCTCTCTCTAATTTGTTCTATTTTTAGGATTATTGGTTGAGTAAAGGAAGCAAGAAATGATTTCTTAATTGATTTGCAAATCCTCTTTTCTTTTTCTCAGCTCACTGAAATCCCTAACATCTCAAATTCATTGTTGCATTCTAGAACACATTCTTCTTCACTTGTAAGAACTTGAAAATCCCTAACATCACCAATAGTGACGTAATGTGTATCGGGGCTTGTCTTTTGTGATTTCTTCAACCACGTCGACCTGGACAGAGCACTCTGTTAGTTGTGACCAAACTGAAATCAGTTCCCTTCGTGATTCCTTTCTTTGTTGTTGATCTTGTTTCCTTTTTTTTTTTACCGTTGCTAGAG

>Ca1:47189596-47190196

CTTATCTCTTTAGATTGCAACTCTAAACAAGAAACCCTTCTTCTTCTTTGAGCCACCGCTCCGGCCACAACGATTCAGATCCGGCTTCGACGTTTGTTCTCTCTCATTCACATGTTCTCTCTCTCGTTCTGTCTCGTTCGCTCTCTCTCTCTCTCGTTCTGTCTAGTTCTCTCTCTCGGTCTCTCGTTCTGTCTCATTCGCTCTCTCTCACTCTCTCTCTCTTTCTCTCGCTCATTCTGGTTTGCTCTCTCTAATTTGTTCTATTTTTAGGATTATTGGTTGAGTAAAGGAAGCAAGAAATGATTTCTTAATTGATTTGCAAATCCTCTTTTCTTTTTCTCAGCTCACTGAAATCCCTAACATCTCAAATTCATTGTTGCATTCTAGAACACATTCTTCTTCACTTGTAAGAACTTGAAAATCCCTAACATCACCAATAGTGACGTAATGTGTATCGGGGCTTGTCTTTTGTGATTTCTTCAACCACGTCGACCTGGACAGAGCACTCTGTTAGTTGTGACCAAACTGAAATCAGTTCCCTTCGTGATTCCTTTCTTTGTTGTTGATCTTGTTTCCTTTTTTTTTTTACCGTTGCTAGAG

>Ca1:47202467-47202711

GTGCTGATAAAACCCAAAATGGTTGGTCGATCAGCGAAAGATAGAGGTGAAGATAATGAAAAGATGGATTGCTGAATATTGTGCTGCTGAGTTTATGTAAAGATTAATGAGAACTATGTTTGTTGAATTTTTAATGGATAAGATCAAATTTTCTGGTAGAAATAACTTTTGGGCAAAACCAATTGCCTCTTGTTAAGGTGTAATATTCATTGCTAGTAAATAATATTTCTTAAGGCGCATCAGA

>Ca1:47189598-47190196

TATCTCTTTAGATTGCAACTCTAAACAAGAAACCCTTCTTCTTCTTTGAGCCACCGCTCCGGCCACAACGATTCAGATCCGGCTTCGACGTTTGTTCTCTCTCATTCACATGTTCTCTCTCTCGTTCTGTCTCGTTCGCTCTCTCTCTCTCTCGTTCTGTCTAGTTCTCTCTCTCGGTCTCTCGTTCTGTCTCATTCGCTCTCTCTCACTCTCTCTCTCTTTCTCTCGCTCATTCTGGTTTGCTCTCTCTAATTTGTTCTATTTTTAGGATTATTGGTTGAGTAAAGGAAGCAAGAAATGATTTCTTAATTGATTTGCAAATCCTCTTTTCTTTTTCTCAGCTCACTGAAATCCCTAACATCTCAAATTCATTGTTGCATTCTAGAACACATTCTTCTTCACTTGTAAGAACTTGAAAATCCCTAACATCACCAATAGTGACGTAATGTGTATCGGGGCTTGTCTTTTGTGATTTCTTCAACCACGTCGACCTGGACAGAGCACTCTGTTAGTTGTGACCAAACTGAAATCAGTTCCCTTCGTGATTCCTTTCTTTGTTGTTGATCTTGTTTCCTTTTTTTTTTTACCGTTGCTAGAG

>Ca1:47189623-47190196

CAAGAAACCCTTCTTCTTCTTTGAGCCACCGCTCCGGCCACAACGATTCAGATCCGGCTTCGACGTTTGTTCTCTCTCATTCACATGTTCTCTCTCTCGTTCTGTCTCGTTCGCTCTCTCTCTCTCTCGTTCTGTCTAGTTCTCTCTCTCGGTCTCTCGTTCTGTCTCATTCGCTCTCTCTCACTCTCTCTCTCTTTCTCTCGCTCATTCTGGTTTGCTCTCTCTAATTTGTTCTATTTTTAGGATTATTGGTTGAGTAAAGGAAGCAAGAAATGATTTCTTAATTGATTTGCAAATCCTCTTTTCTTTTTCTCAGCTCACTGAAATCCCTAACATCTCAAATTCATTGTTGCATTCTAGAACACATTCTTCTTCACTTGTAAGAACTTGAAAATCCCTAACATCACCAATAGTGACGTAATGTGTATCGGGGCTTGTCTTTTGTGATTTCTTCAACCACGTCGACCTGGACAGAGCACTCTGTTAGTTGTGACCAAACTGAAATCAGTTCCCTTCGTGATTCCTTTCTTTGTTGTTGATCTTGTTTCCTTTTTTTTTTTACCGTTGCTAGAG

>Ca1:47201559-47201869

CACTTGCTAGTTAATACTAAAAAACATTAATTTTTATTCAATTTTCCTTTTATTTGGACTATTAAATTATTACTAAAACATGTTAATTAATTACAGTCCCTTGGAAAAGTATGATGAGTTGAAAGAGTACTTGCAAGTAAATTACGTATACACAGCTCAATACCAAAACCTCTTGGTTACGTTTATATGTGAAACCATTCTCAGTCAAATATATGGAGGTTGTGTGGCTTTAGTAGAATACGACAAATGCCTTGTTAATTTCGATAAATATGATAACAATGCACATAAATACTTCGGTTGGGATTGACAG

>Ca1:47242071-47242386

GTATGTACTGAAATGGTAATGCCTGTTGGCATAGGAAAGGGTACCATGTTTCAACCCGGCACTTTTAACTTGAAGAAATTTGTTCTACAAAAAGTTTGGTTACTCCAATTTGTCTCGTTCTCATTGGATCACTACTTATTATGGAGGCCATGTATGAAATATTGTTTAAATAGTAAGATTATATTATTAGCAACATAAAACTAATAGAGCTATTCATTGCTTGACTAATGGAAGAATATAAGTTTGGTTCTTCAAAAAATTTGCTAGCAACATCATATTTTCGAATGGACTAAAAGATCCTTACATTAGTGTCAG

>Ca1:47242619-47242937

GATCTCATGTCTTGGATACTCTAGGTGCTGATAAAAACGACCAAAAATAGTTGGTCGATCAGCGAAAGAAAAAGGTGAAGATAATGAAAAGATGAATAGCTGAATATTATGCTAATGAACTTGTGTAAAGATTAAGGAGGACTATGTTTGTTGAATTTTTATTCAAATTCTCTGGTAGAAATAACTTTTGGGCAAAACCAATTGCCTCTTGTTAAGGTGTAGTATCCATTGCCAGTGAATAATATTTCTTAGGGCGCGTGAGATACATGATTATATTTATAAATATATTTCTATTTATATTGGAAAAGAATTATGGTG

>Ca1:47323482-47323716

AAACTTACATGGTAATTGTCATAGATTTTTTAGAGGAAGACTTGTAATTGGGCGTTGGAAGCATTGTTGGCATATGGATGAAACTACCAGCAGCAAGAGCCATGATGAGAAATAATAAAGAAGAAGCTTGCTTATACTTTATAGTACAAAATGTTAGTGTGTTCAATCTATGTTTGGTTGTAGTATCTGATCATGTGATTTTGAAGGAGTGATTTATAGGGAATGAAGTGTGTA

>Ca1:48030070-48030474

GGGATGATGATGATGATGGCTTTGTGGTTGTCGAGAATTTTGTTCTATTTATGTTTTCCTGGAGTTAATGGCATGGTGATGGCTGCGAAGAAATTTTCCTGTACACTCGCTTTTTATATCATTCTTTATAAATGTTTTTTTTAGATCTTTTTTGGACCCATGGTATGAATTGTGGGAACTGCTTTTATTTAGATCAATTTTGGACCCATGTTATGAATTGTGGGGGCTGCTGTTTGGATTTTGAGTTGTTTATGTACTCGGAAATATTGGCAACTGTGGTTGTGTTTTTTGGAAGTTTGTTGTTTTGTTTCGAATATGATGGCCGTTTCCCTTTAATGTTTTAATGTTCAATAGCAGGGTAATTTTGTTCTTTTCATGTCAGATCATTTACAAAGTGAAACCAG

>Ca1:18071-18325

GGCATGCTGAATTAGGTAGACACAAAACAGAAAACATGTTGAAAATGGGGTAAGAATCCAAATATTTTAGTTTTTAGTCCTAACCTGGAGCAACAAGCAATCAAAGCATATCCACTTGACCCGTAGAAATGAAAGTAAGAAAGCTAAAAAAATATAATGACCACTTCACTTTTGCACATGCAACCATAAAAGTAGGAAAGCTAAAAAAAATATAACGATCACTTCGCTCTTGCACATGCGACCAGGTACCCGTA

>Ca1:18442-18673

TTTTGTTTTGGGGATTCACAAAAAGGTTTGTAGCCACTTAATGAAGGGAATCTTAAAATGCTCATAACAAAAAGAGAAAGTTGTGGATGTCTTTACAATGCCGAGCAAATCAAGTATTCATATATCAAATCCCTAAATAAATCAATCCATTGATTTGTCATTTCGGCTTCCTCAATTGATCCTACATTTGAACTGTTTTCCAGCTTGATCCTCAATGACTATTTCAAATTT

>Ca1:18788-20370

CAACAGAACCGAGGAATTAAGCGAAACAAGCATAACACAACATAATCAAATCCATTGAATAAACATATTTATATAACTGGAAAAGAAAAAAAAAAAAAAAAACATCAGTCCGCAGCATTCGCCACACAACATGAACCATGATTAAATAATGATTCTCATTTCTAAGTTATAAAACTAGTTCTTAGGTAGCCAAGCAACTATCCTCTTCACAATACTAAACCAACTATGTTGACAACTATAATTATATACATTATTTTTAAAAGAATTGGCTGATGACTAATTCAACAGCTATAATGAGAGGGGAGAGCTACTTTGGAAGGAAAAAACATTCAGTCTTTTGCTTAATTATTCAGTATACATATTTATTTTCTCTGTGTATGCTCTCTATTCAATAAGCTTTTTAGCTTTTTAGCTCTTTAGTACTCAGATGAAGTCCAACAAAGAAAAAGATATTCTATAAGAAAACTAAAGCATAAAAAGTCTAATGCAGAAGTAGAAAACTGGCAAGGTACTATTGAAAAAAATGACACGAGATATGAGAGAGCATGGGATCAGGTACATATTTCAAGAGAATTTCCAAAAAATTATTTGTCATTGACTATATCAAAAATTGTGTAGCTCATTACCACGCTTCAAGCCTGTGCACCTAATCTTCCGATGCAAATCATAATCAAGCTTAGTGAGCATAGAGCCCAACCCAACGACACAACCTCTACTTCAAACTCATGTTCACATACCGATATGTTTGCACAATGATATCATAATCAGAGGAAATATATGGTAACATCAATACGATGGCCTGAAAATTGTATCAGCATAAGATCTATCACGCATAGGTATGGTAGTAGTATCAGTTACTGGTACCAGTACGGGATATGGTATTTTTTTTTAAAAAAATTAAGCTATGGATACATCAATGAAAAACATTAATTTTTTTATATAATTTAACTAAGAGAGTTAAATTGTTCAATTCTCAATACAAAATCAATATAAATGTCTAAAAACACACAAATTGTGTTCGATTACAATAACAAATAAACTCAAATAAGCAAATAATATTAAATTATTATATAACAAAATCATGAATTTCACTCGAAATAAAATAAATAAAAACAGCATACCCACAAATATGGTACAAGTACTAGTACTAGTACTAGTACCGGGTAACCACCAAGTATGGGTACTTCACCATTTTAGAAGTACTCCATGCTTTATAGCATAAGATGCATAAGGAATGTTTCAGCCATTATTTCGTATATAAAATGCAAGCAATATCCCAATCATCTTCCACATCATTAATTAGAACAAAATATAAAATGACAATAGGTATTTAGCTCTACAGACAATACCTGGATAGATGCTTTCCCATTCTTTCAACAAATATTGAATAAAAGTCACAAACCAAAGGTAGGTGGCAGTTCACAATTTTATGCTCTGAGACTATGTAGATCAGATCAAGAAATATTGGGTAGCTGTTTTTACCAATTTTAAGGCTGACAGATATGGTGACAGTTTGGTTTGATAAAGCTGAAATGCATATATTAGTCTACGCATATATCACAATATTTCGGAAGTCAACGTG

>Ca1:19414-20370

CACGCTTCAAGCCTGTGCACCTAATCTTCCGATGCAAATCATAATCAAGCTTAGTGAGCATAGAGCCCAACCCAACGACACAACCTCTACTTCAAACTCATGTTCACATACCGATATGTTTGCACAATGATATCATAATCAGAGGAAATATATGGTAACATCAATACGATGGCCTGAAAATTGTATCAGCATAAGATCTATCACGCATAGGTATGGTAGTAGTATCAGTTACTGGTACCAGTACGGGATATGGTATTTTTTTTTAAAAAAATTAAGCTATGGATACATCAATGAAAAACATTAATTTTTTTATATAATTTAACTAAGAGAGTTAAATTGTTCAATTCTCAATACAAAATCAATATAAATGTCTAAAAACACACAAATTGTGTTCGATTACAATAACAAATAAACTCAAATAAGCAAATAATATTAAATTATTATATAACAAAATCATGAATTTCACTCGAAATAAAATAAATAAAAACAGCATACCCACAAATATGGTACAAGTACTAGTACTAGTACTAGTACCGGGTAACCACCAAGTATGGGTACTTCACCATTTTAGAAGTACTCCATGCTTTATAGCATAAGATGCATAAGGAATGTTTCAGCCATTATTTCGTATATAAAATGCAAGCAATATCCCAATCATCTTCCACATCATTAATTAGAACAAAATATAAAATGACAATAGGTATTTAGCTCTACAGACAATACCTGGATAGATGCTTTCCCATTCTTTCAACAAATATTGAATAAAAGTCACAAACCAAAGGTAGGTGGCAGTTCACAATTTTATGCTCTGAGACTATGTAGATCAGATCAAGAAATATTGGGTAGCTGTTTTTACCAATTTTAAGGCTGACAGATATGGTGACAGTTTGGTTTGATAAAGCTGAAATGCATATATTAGTCTACGCATATATCACAATATTTCGGAAGTCAACGTG

>Ca1:23192-24390

ATTCTCAATCTCTTTTCATATGAATAAAAAATTCATATTCAAGGCAACATTTACTGTAAAAAATCTATCTCAATTGATTTTCAGGTCATCCAAAAAACATTATGCCAATTTTATATGAATACAAAATTCATATTACAAACGACATTTTGTAGGCCACATCTAAATCAAGAATCTATCTCATAGTTAAATTTGAGATCATCCAAGCATAGCGACTGACTTGTCTTAAGGGTACATATTGCAACTTTTCAATTTTTATTAACCTTCTCAGTCAAGAATAATCAAGAATCATCAAGATTATACATTCTTGAATAATCAATTATAGTGCGTTAATGTTGCAAACAAAAACCCACCCCAAAACATTTGTGTCAAACACAGTTTTGGAGACAGAGAATGTTTGCTTAATAGAAGAAGAGAATCAATAGATCAGAAACCAAAGGATAATGGGTTGAAAATGAAATAGAGAGAGATTTATACCTCTCTTTGGAATGAAGCAAATCATGGACAGCAGAAACTTGATTCCAAATAACAAAGTATGTGACTTCGTTGTCATCTTTTTAGGGCACCAATTCAAATCAGAATTCGAGGATTCGAATGACCAAGATTCATAGAGATCGGGCGTTCTTGGTTGAATTTCAAGAACAGGAACGGGAACGGAAAGCTTTATATTTTATTTTGTATCACCATCTCTCTCTTTCTTGTGACTATGCAATCATCTTTTTTTGGCACCAATTCAAATCAAGATTTCTAGGGTTTGAATTTCAAGAACATCGTTACCATTACCGTCGACGTCGCAACGAATAAGAAAAACAAATTGGCTCAAAACATCAACACCAAACACGAAGATGATACCGGTCGAATCGATTAAAGCAGAAGATTGAAAATTGATAGGGAGAAAAAGAGATATCGAATTCAAGCGATTAGGGTTGAGAGCACACACCTCTGTTTTTCTTTTTCTGGTTCCGTCAGCATAATTGAATTCAACACAGAGATTCTATTTTTTCCGATTTCGTGTTACGCCTCTTTTTCTCCCACCGATAAAGCAACCACAAAATTGAAACCCAAGGAAGGATATGTCAAATTGGTACTGGTCGGAATGCAAAGGTTCCAAATCCTTTTAGTCAAAGAAAAAGCACTCCCTTGAAACTCTTATCACCAGAAATTAAAACAAAATAAATTGATTTGTCTTCTTTCTCTAACT

>Ca1:178020-178228

CGTATCGCACGACACTGTGAAAATTTGAGGAAATGGAGGGAAACCCTAACCTATTCGCACGAGATACCAATCAATCTATTCGCACGAGTATAACATTGTATTCGGTTTTAGTTTTGCTTTTCCATTCATCCACCTTAAACGCACCGTTTTGTTTCCATCCACTTTTGTTTTTCCTCCTAACATTCCAACTTCTTCCTCGCCAATCATC

>Ca1:178020-178254

CGTATCGCACGACACTGTGAAAATTTGAGGAAATGGAGGGAAACCCTAACCTATTCGCACGAGATACCAATCAATCTATTCGCACGAGTATAACATTGTATTCGGTTTTAGTTTTGCTTTTCCATTCATCCACCTTAAACGCACCGTTTTGTTTCCATCCACTTTTGTTTTTCCTCCTAACATTCCAACTTCTTCCTCGCCAATCATCATAAACTAAAAATGGTGCCAATTTCG

>Ca1:178020-178256

CGTATCGCACGACACTGTGAAAATTTGAGGAAATGGAGGGAAACCCTAACCTATTCGCACGAGATACCAATCAATCTATTCGCACGAGTATAACATTGTATTCGGTTTTAGTTTTGCTTTTCCATTCATCCACCTTAAACGCACCGTTTTGTTTCCATCCACTTTTGTTTTTCCTCCTAACATTCCAACTTCTTCCTCGCCAATCATCATAAACTAAAAATGGTGCCAATTTCGAT

>Ca1:388363-388752

AGTAGATAAAAGTAAGGTACTCAAAAAATTGTTTGAAATCCTTTTGTTTTTGATATTCTCATACACAGAAAAGCAAACAAATAAAATAAAGAAATAATTAAGAATAACAAGAGAAATTTCCTCCTATACAAACTCTCTCTCTCTCTCTCTCTGTCTGCAAAGTCCCTTTGGACCAAAATCAAACGGTAATTAAATAACAAACCATACCATATCATCCATCTAAACAAATCAACTCCCTCAAAATTAAGAAAATAAAAAGTGACAAGATTTTCACAACTAATGCAACATCTATGAAAAACTCAAGTCCTTTGAGCATGCACAGCTAGCATATGAATCCATGGAATGAATTTCCAAAAAGCAATAAGTTATAAGAAATTCAACACAGAACC

>Ca1:608736-609059

AAATAAACATATATATATAATTTCCTAGCTAATAGTACGTTTGTTTTGGTCATGTTTTAGGACTTAGAATTGTAATGCCATTGCAACCAAACAAAGACAAAATATATAGTGGTGAATACTCTAGTATAAATAAAAATTGAATTGATAGAAAAATATAAAATGTGCTATAGTTTTAGAGATTAGATCCATATACTACACTTTACTCAGAGTGATCAATATCAATCTCTGAGACAAATAAAGAAAAAGAGACAAGAAGAAAGCAAAAAAGAACTGCAATTTGCAGCCGCCACCTTGTTCCTAAGATCAAAAGAAATAACTAATTT

>Ca1:608736-609033

AAATAAACATATATATATAATTTCCTAGCTAATAGTACGTTTGTTTTGGTCATGTTTTAGGACTTAGAATTGTAATGCCATTGCAACCAAACAAAGACAAAATATATAGTGGTGAATACTCTAGTATAAATAAAAATTGAATTGATAGAAAAATATAAAATGTGCTATAGTTTTAGAGATTAGATCCATATACTACACTTTACTCAGAGTGATCAATATCAATCTCTGAGACAAATAAAGAAAAAGAGACAAGAAGAAAGCAAAAAAGAACTGCAATTTGCAGCCGCCACCTTGTTC

>Ca1:608736-609631

AAATAAACATATATATATAATTTCCTAGCTAATAGTACGTTTGTTTTGGTCATGTTTTAGGACTTAGAATTGTAATGCCATTGCAACCAAACAAAGACAAAATATATAGTGGTGAATACTCTAGTATAAATAAAAATTGAATTGATAGAAAAATATAAAATGTGCTATAGTTTTAGAGATTAGATCCATATACTACACTTTACTCAGAGTGATCAATATCAATCTCTGAGACAAATAAAGAAAAAGAGACAAGAAGAAAGCAAAAAAGAACTGCAATTTGCAGCCGCCACCTTGTTCCTAAGATCAAAAGAAATAACTAATTTCTGTCAAAACAAAAAACAAAAATATATAAATATATATGTTACCTGAAAAAGCAAGGCAAAGTATTTTGATAGCATGAAATTTGATAGCAAGAAAAGGCAAGCTAGGAATCTGAAACCCTAAAGATGCTACATAATTCTGAAACATCTACTGACAAAATGCAGAATTTATTTTAAAGCTTCATGTGTTTCTTAATACACTCCTCTCTTATGCCGCCTTTAATATAAGAAAAAGTTTAATTAATCAAATATAATATAAAATTTAGATAAATATATTTATTTTGTTGACTCATTATATTTTAATAAGATACAGAGTATTTACTGCTTTAACTTTTCAATCAGAAAAAAGCTATAATACTGTTAATAACAATCACTCACTAATCATAACCGGAGAAAAATAATTATGAATAATCTAGATTTCTGAGCCTCCTTTAAACAGTTAAAATCACATGTTGAAAGAGATAAATATATGAAGCCGCAAGAAAGAAGACAAATTAAGAAAAGCAGTGGCAAAGGTGCAGCAACCAACCAACCAACAACAAAGTCAGAGGCATCTTCCTCATCTCTTTGCATCC

>Ca1:955925-956166

CTTAAAAACAGAAGAGAATTATGGGTGTGGGAATGATGGGGAACAGTTGGAGAGAGATTCCTCGTGTCGTGTGTGTGTGTGTGTGAACTGAACACAAGTTGTTGAGCAACAGTAGTAGCTTGCTTGCTTATTCCATGGAGAAATCATCTGGTCCGTTCATTTTTTCATATGACGAAAGAGAAACAAAGAAAGAACCATTAGATGAGATGAGATATTATTATTCTTTGTAACAACCTCACCC

>Ca1:1036808-1037065

GTCGAGAAGCAATTGAAATTATAGAAGTGGAAAATCAATTTGGCACAAAAGAATGCATGAAAATCACCCAGAAGAAATCATGTGTATTATACACACAAAAACCAACTTTACATCAATGAGTCCACAAATTTCTTGATAACTAGGGATTAATTGCTAATTTGAAATTCAACATCAGTATATTGTACACCGTGCAGGGCCACCTGAGCACTCACATGGCTCTCCAATAATCACCACAAGTAGCCTCAGAGAATCACCTA

>Ca1:1036808-1037062

GTCGAGAAGCAATTGAAATTATAGAAGTGGAAAATCAATTTGGCACAAAAGAATGCATGAAAATCACCCAGAAGAAATCATGTGTATTATACACACAAAAACCAACTTTACATCAATGAGTCCACAAATTTCTTGATAACTAGGGATTAATTGCTAATTTGAAATTCAACATCAGTATATTGTACACCGTGCAGGGCCACCTGAGCACTCACATGGCTCTCCAATAATCACCACAAGTAGCCTCAGAGAATCAC

>Ca1:1043560-1044202

GTTTATATTGAATCCAAAGTAAGTAATTCAAGTAATCAATGGTGGTGGTTTCATAAACTATCCCTAGTTGAAATGGTTTAGATGGTGTTATTCCCCCATGCTCCATAGTGTTGCATCCGTGTTAGTGTTATTTTTTCATTCCTCTATTTAGGGCAGAATAAATACAATAAATGTTAATTGATACGAGTTGCTAATTCAGAAACTCATTAAAACTGCAATATGAAAAATAACATTATTCAAAAAAAAAAATTACAAATTATATTGGATACATAACTTTTTTTGCTTTTTTTGTTCTTGCATTCTATTAACTTTTGTTGAACGTGATTCAGATATCACTTTTTCTCTATTGTTAAGACATAGACTTTGTAAGTTCGGTCTATATACAGATGTCACCGATTTAACTCAATTAAGAATTATATTAATCGATGTCACCGTTAAGACATAGACTTTGTAAGTTCGGCCTATATACAGATGTCACCGATTTAACTCAATTAAGAATTATATTAATCGATGTCACCGTTAAGACATAGACTTTGTATGATATTTAATCAAAGAAATCACAGCCGTAACAAATATGAAGAATTCGGTCAAACAAATCATAGACAACACACACGTCATCTTCAGATCTTCAACCACTGCACA

>Ca1:1044560-1044823

CTCGTAATTGAGCTGCAAAACAGAGGTTAGGTCAACGATTGGCTACATTGGATCTGAAGAATGAAGGTGAAAGTAGTTTGCTACAAATGGAAAAGGGTGCGGCGGCAAGAAAAAGAGGTTAGATTTTGAAACCCTAATTAGAAAGAAGGTGAGGTGTAGAAGTATGAGAGGAGGCAAGACTATAAATAGATCGATTAGAGTTTAGATGAGTGAGGATTGAATGAAATGAAATTGTCAGTTGTAAGTGACACGTGATTGTGAAT

>Ca1:1249554-1249932

CACGTCTGTGTAGTTGGTTTCACTCCCTTTTACATATTGGATGCTAATGCTATATATATAGCTAGGATGGTGGCTTCACGTCAATTGATACTTCGGCATTAAACATGTGCTCAGTGCTATCTGTTGGTAGTACGGTTAAGTTCACTTTTTTATTTTTGATTGTAACATTACATAAATGCTTAGTGGTAAAAAATGTTCGCTGTAGCTACTTGTCTCAGTATTGTTATGTAGATATGGCATCAAGAAACAAACCACAAGGCACATGTTTAATAGTAATTTACATTTTGATCCGAAGGGGATCTCGTACGGTGGGTGGTTGGAGTAGCTTGGAAGCGGTGCAAGACGGCAGCATGAAATTCTAGGAGGAGGTTGCTGTAC

>Ca1:1250004-1250355

CTCTCTTATGAGTGTAGATGTCCTTTTATAGTAGGGCTATTCAAGGAAATTGGAGACAATTTTGTTGAGAGGTTGAGCAATATCCGCCATATCACCGAATCACACTTTGATCTCCATCAGACGGTGATCGCCTGTAGAAGGGTGAAATGAGCGTGTGCTAATGGATTTGGCATGTGCAGAGAGGACCCTCATGATAGAGCCAAAAGTGGACAGGCCTTGGACCAGTTCGAAACATGTTTGAATACAGGAATCAACACTTGTGAGCAATTAAGAAAGCAAAAATATTGCTGACAGAACACGGTTCTAGGTTCATGTTAAGCATTTCCTTATTGCCTCCCTCCTTCATGCTAC

>Ca1:1386302-1386987

CGTTCCTACAGATTCGTACACAGCCAATAGTGGTGTAGGAAAAATCATTCCTGAGTCTTCCAGGAAATCCTCATTCCATTTGGCAGAGAATTCAGCTGATTGGTGACATCACTGACAGTATGACTATAAGAATTAAATGAATTGCAACCGTATTCAAAAAATTGAGATACAAACTAAAATAATTCGGAGGAAATGGCTATGGCTGATAGCACTTACTTATGCCCAAGCAAAGTTACAACATTTTACAAGCATTTAACATCAAAGGTTACGTGTTTAATTTTTCCTATTTTCCCTAAACCCTATATCAAGTGGGACAAGCCCCATGATCTCACCAAAACCCTGCAGAAGTGCCACAAGGGCAACAGTATAGGCCAGTAAATGAAGACATTTACCTTCTGAAAAGCGATACAATCAGCAGTATCACATCAGATGGTTAAAACAATCACAGTATAAACAATACAACTGAAGAGATAGTGGTAAGACCTGTTATAGCCCTTATTTTTTTACACAATATTTACACATAAAATTAGTATGAAATTGCCAATAATAACGATAAAGTTGCTATTGTAGATGCATCAGGTGTGCAATCTAGGCCTATACTCTCATGGGTCAACCTTGGATTACTATACATATGAAATTCAACAGAAAAAGCAAGGTCATGCGGGTGAAAGCAAAATAAAAGATC

>Ca1:1396736-1396976

CTATAGTGAAAATAAAAGACCAGATCTGTGTATGTTATTTGCTTCCTCGATCTCTTACTAAGTACATCCTGTCCACACAATAAGGAGGAGAAGTGAAGGGGAAAAGAAACCAAAGGGTTATTAAGTTAGAATTAAGAATGATTCATAGATTAGAACTTAATTAGAAGGGGAATAAGAATAATCATAATCAGCATTCTAATTCAAACAGGTTATGGAGTTAAGGAAAAGGCATGGAACACT

>Ca1:1396736-1397036

CTATAGTGAAAATAAAAGACCAGATCTGTGTATGTTATTTGCTTCCTCGATCTCTTACTAAGTACATCCTGTCCACACAATAAGGAGGAGAAGTGAAGGGGAAAAGAAACCAAAGGGTTATTAAGTTAGAATTAAGAATGATTCATAGATTAGAACTTAATTAGAAGGGGAATAAGAATAATCATAATCAGCATTCTAATTCAAACAGGTTATGGAGTTAAGGAAAAGGCATGGAACACTATGGAAACAGCAACGTGGTAGAGAGAGAAGAAAATATCTAAGGTTTAACGAATATTAAAT

>Ca1:1388077-1390837

CACAAATCAATCAGGCCCTAAAATTTTACAAATCTCTTTTTCATAACAAATATATTATCAAGAAAGGAAAGAATACAAGACAAGCAAGCCTCTTGAAACTCAACAGAATCAGTAATAATTTTCAAAATAGTTTGAACAAGTTGATACCCAATTGCTGTCACAAGCCCAAATCAGCTATTTGACAATTCTCTATATGAGCTATATCTTCAAGCATCGACATTCATATACCTTTGTATGACTGCCATCTCTAAATATACGACCTCATTCACTAAATCACCAGAATTAAGAAAGTTGGTAATACCGTAATAGTATTAAATTTGTTAGCAATTGACACTATTTTTACAAGTTTACCCTTTAAGAGAGAATTAGTTATTGTTCCCACCATAGTATTTATTGTTGGTTGCAGAAAATAATGTAAATTAAATGTTAGTAAAGAAATCATTCAAAGGGGGGACATATATTTAGAGACAGAGGTAGTAATTTATATTCTCTTCTTAATTAGGATACCTCAGTTGTAGGTTTAATTGTTATTTCTTCTTAATTAGGATAAATGTGTATATATGGCAATTAGCTAATTTATTGGGCCAAAACCAGTTTGTCAGATTGTAATTAAACACTATTTATATGAGAAATCTTTGGCTGGAGAACAAGTATTTGGCCAAAGTTTTCTGGTATCAGTGTTTTTGCTACTCTTGAGGTCACCATGGAGGAATGCAAATTTTACATCCATTGGATGTAATGACCAAGAGAGGGATGCAGCAAGGGCATGAATTATTTGCATAGTAGTCTATCCGTCTCATATTAAGCATCGTGTTAGAAATATTTGTTTCTCCACAGATACTCTTGTTGTTGTATCTCAACTTACCTAACTACTCCACTAACTTTACTAATGAGAGCATATAAGTCAATAAAGCTCATTTTAATTAGTACATGGAATACTTTCCTTAAGAAGTGTGCATAACATTAAATGGCACTTAATATGATACGGAAAGTGGTCCTCTTGGCATCCGGAGCAAAAGTCATGATAGTCAAATCTGCATTCTTGGCACATCTTCACCAAATACATGGTACATCAAATCCACAATTTCCTATTTGGCATATTGATGTTTGTAGACTAGCCAGCATCAATTTGAGGGGGGTGTCATGTTAAATATTTTGGTCATTAGAACCAGTTGGGTGATGATTATACATTTATACATTTGTAACTGCTATTCTTTAGTTAATGAAGATACCTTGGGTATAACCATTTCTTTAAATGGCAAATATTAGTTATTAGTTGTTAGAGACAGAAGTTGAGTACACGACCCCTTAGCTCTCCAGAAGTCATACATCAGGCGTAACAATTATTCTCGTCTTAGTTTTGGATTAGTCTACACATTGATTTATTTAGTTTCATACTTTCATTTTTCAGTTGACAACAAAAAAATCTCAAGACAGGCCCAACATTTACATATTATAAGAACAAATCAATGACAAAAAAAGAACTAAGGAAACTACTCTTAAGAGATATTCAAAGGTCCTACTCCTCTAATGCAAGAAACTTTATCTTACGAGATAATCCAAGATATATTTCTATCTTTTAACACTTCAACTCAAGTTTCCTAAGCTTGTTACATACTTTACAAAAGAGAAAAAAATAAACAAATGATATAATTGCATCCGGAAAAAGAATCAAACCCAAATTGCAATTGGGATAGAAGTTGGGATTAAACCACAGTTGAGACAAAAATGTGTCAAAGTACAATTAAGACAAAACCACCAGGACGAAACAAATGTCAAACCTCAACCGAGAAAAAACCAGTGCTAAACCACAACTGAGTTACTGAGACAAAACCAACATTTTCAAAGAAACACAACATACCATAGGTCATCTCAGAGAAACAAAGGTAGATTCAAGGAGATGGTGTAGAGATGCTGCTGCAGTTACCAACAGTGGCAAAACCATATTCAAATTAGACGCACGAGATGAAAGTGGCCTTGATAAAACAAGGAAAGAACGACGAAGAACAAAACTTGACTAGCAGCATGGTTAACAAGCAGTATGAAGGCGACACGTGGACTTTACATGCTGACTAACACCAAAAACTTAATTTGATGAAAAGTGAAGGATAAGGCCAAAAAAGGATTCTGAGTACGACTATTATAATAGAGTTCCATGCATTAGGGAACTTGGTAGCATAACAGCGAAGTTGCAAGTCACAGAGGATGGTTGCTACTTTGATACTATGTTGAACTTATAGGGTTTGTTTTACTTTTAGATAAGAACAGGATCAGAATTAAAAAAATTAAAGACTTTGTATTATAATAACATGGTTGCTACTTTTAAAATGATGATTATGGTTTGATACAAAAAGACTAAACATTAACTATTATTTATATTAATACAAGACTCCAAATCCTAATTACTTAGGCTTTGTTTGGGAGTTTGGAGGGGACAGAAGGGGAAGAAAATAGAGAGAAATCTTCCACCTTTTTTAGAGTTATTTTGTAGAGAATGCTTAAGATTATAAACACATTTTTTGAGTTTTCCCAAACTGGGGGATTTTGTATTATTAAGAAAAATAAACTCTCCAAAGTCCTCCCTTCCCCTCCTCTTCTTCTAAACTCCCAAATAGACACTTAGGGAAACAAATCATGAAAAAAGAACATAAATAGGAAAACTAATCTTAAGAGATATTTGAAGACAAGAAACTAATCCTACAAGTAATGAACTCATTAATTTCAAAGACATAGTATACTATATGAGAGAGAACCGTATAG

>Ca1:1464941-1465347

AGCCAAAATAGTTGACAACAAATAACGAGTCACAAGCATATGTTTGGATTTACACTGAAATTGATATAAACCCATTAAACTTACAAATTTGACAGAATTAGTACACTTCAATTTTTTGTCAAACTCAACGGTTCTTCAAAAGCACGCGGTTAAAATATCAGCCGTCCAATTTAACTCGCAATAGCTTAAACTCTGCACCGTCAGATTGCAATCCATTTTCAGAAATCAGCACCGCGCTTTCTTTCTTTCTTTCTTCGATTCCATCGATCCATAACACAAATCTTAAACGATTAATTAAAGACTCAAATCAATGGCTCTGAGGATTCAGCATATTCCCAAGCTCTTCTTCTCCGTCTCACCAATCACAGTCTTCAAACACAACAAACCCAAGACCCGAATCTCATGC

>Ca1:1511268-1511633

CTGGTTTAGAGACCAAATATTTTGCACATTTTATGTAGACAACATATTTATATTTCATTATTTATCCTCTATTTTGGCAATGATTTATTTTTTAAAATTTATTAATCACATTTCTTGATAACAATTCATATTTTATTTCCTTCTAATGAAGGTTACTTATATCTATGAAACATAATGTTTTCTTCAAACAAGAAGAAAAGCTTTAGCTTCCTAAAGATCGTAACAGCAACAAATTAAAACATTATTTTCCTTTGTACGACATTGCGATGTCAGATAGCCTCGCTTAGTGCAAAATGTTTCACAATCAGGGATTTTGGCACAAGTGTCAAAGCAGACCTCTGTTGTAAGTACAACTCTAGCCATTC

>Ca1:1967482-1968766

ACCTCGTCTTTGTAAAACCTAGTAATGTATCAAACCAATCATTGCATGGCCAATGAGTTAGAGGGTTTGGTAATTTCCACACTAAATTCATGATGACAACTATTATAGATTGAGTCACAAGTTGGAAATGATGAAGCTAGCTCATTAATTAAAGAAAAGACAAACAAGTACAAAGTCTAGTTTAAGTAAAAAGTTCAGCCATGACAAAAAAGTACAAAGAAACTTAACCAAACAACATTAGCAAGAAATTCTCAGATAGCTCATAGTCTCATACCTCATCCTTGCAACAAAGTAAAAAATCATTCATGATCAACCACACACACACACAAAAATTTGTAGTACTAATTTGTAATATTCTGCTTAAATACAAACTGTCACCAAATATTAACCCAAAAAAATAGCCAATTTTAAAATAAAATAAAAAAATGAAAGAAACCAGGAAAAACACAAACAAGTGTATTTACAAACATACCCTCACTCAATCATGTACCAGCTAGCACTGCTGTTCTGTCAACTCATCAGCAGCTTGCAATCCAATCCATTCCACCATTTTGGTTTTCCCCTCAAAATCTTGTGACATGACAAAATGGTGAATTATATTTATTGAAAGAAGCATAGAGAAGTTAGAAAGCAAAGGGAGAAGAACCTGATCTTCCATATAGCAACCATGTTGAGAGTCCTCATTAAGAAGACAATGGCAGACTCAGAGTTGAGCAAAGTTATGTCACATACATACATTCCTCTCTGGTGCTAGTTTGTGTTTGCAAAGCTTAACCTCAATGAATGATATTGACATTGTTGTTAATTACTTGTTACACAATACTGTGAGTGAGGATTAATGGAGAAGAGTATGGGACACGCGCATAGAGAAAGAACAGAACAGAACAAAGAAAGAAAGAAAGATAGATAGATCACTGATCACATCATGTGTATCAAAAACAAAATGGATCTAACATAATAACATAAGGCATTAATGAAGAAGGAGAAGAAGAAGAAAAAGAAAGGGTGGTTAGTTACATTACATAATAAAGACCCAACAGAAGAAAACGTTTAAAAATGCAAACTTTTTGTCAGAGAAACACAGTAATAAGGATTGGCCATTCTGGGTTTTCTCTAATCAACAAATTTAATTAATGATGTTTTTCTTTTTGTCATTTTTGCAGCATGGAATGAAAAAAGAGGGAGAGACAAAGGAAAGAAAGAAATGAATGATGAAGAAGGCATGAAATGAAATGAAAGCATAATTATGAATTGAAATAATGATGAGTAGTAGTTAGATGTTAG

>Ca1:2514479-2514959

TTCCGTCTTATTTTTCAATATATTTATTTAATAAATGGTTGCTCATTTAGTTATTGATCCAAAGAACAATGATAATGAAACCTTTCATTTTTCATCAGTACCGGTATACATTAAAATGGGAGGAATTACATGAAGAGAAAATGTTTTAAGAGAATAAATTCTAAAAAATTCATATGAATTTTCTCTTTTCATGATAAATAAATGGATAGCAACCAAATCTTCCTTGTTATGACTTATGAGTAATGATTAATTAGGAGCATAGAAATAGATCTCTTATAACAATTCCACGGTGCAGCAGCTGCGAAGACAGGTCAGGACTTTTTGTAAAGGACTCTTGGCTTTCTTCTTCTCCTTGTCCTCATCCTTCATGTTCTTTGGTGTGATGAAACAAAAACATGAGCAACATGGGGACACCAACCAGAACTTCATTTCTTTCACAACCTGCTCTTCTGTGTGTCTAATATGTTTTTTTTATATAGA

>Ca1:2648701-2649548

ACGTGACCTTTATTATTACATCCGTATCTGTAGGCATACACAGTGTGGCCATCTGCTAGCTGCAGTCACTCACTCTGAATCACAATCACCCACCACCCCTGACTTTCACCACTCACTGAACTTTTTTCTTATCACTCTTTTTCATTTTCTCTTTTTTGTTGATGAAAAAAGAATAGTAGTATTAGTAATTAGTAATAATAAAGCGAGCCCAACGGAGTCAAACACGCCAACTATCGAGACCAAGCCCATCTTCTCAAGGTGGCCCAACTTCACCTGTTCCCTGCTACTCAGACACCATTCCACGTGGGAGCTACTTTCTCTCACTGCACCAACACCAACCCCACTTATTCCCCAACCAATTATATTCTCCCACGTCATCACCCACCTTTCCCTTTCAATTACGTCAACTCAAATTCCCATAAAACACCTCTTTATTACAAACTCCCCTCCAATAAAACAACACACTATTCATATTTATCTCAAATCTCAATCCCCAAAAAAACACACACTATTTCAAACTAAATATTTAAATTAAAAAATATGTCAGGCGCCGTTCAGAAAACGCCGTACAATATCAGTAGGGCATACGTCATTCTTATCACCCGTTTCCACCCCACACGTCTCATCACCCGGCGGCTCTCCCAATAGGTCCCACCACTTTCTTTCTCCATCAAATCACAACCGTCCATCATAAATCAACGGCTCTCCTTTATTTTATCCAAATTTGCTTCCCACCCCGTGTGACATGGTTTGTCTATATAAACAAAAGAAAAAGAGATGCAGAGGAGGTGGTGCCGGCCAGCCGACAATTACGGGAGCAAATGTGGGTCCCATCACTTTTATAAAC

>Ca1:2855127-2855488

CTACTTCACATTTCTCATGTATAATGAGCCTATCACATCAAAAAAGAATTCATAGCAGAGGCAGCAAACAATTGAAGTAGAATAACTAGCATCCTCACAGGAACATTTTATGACTGCAACACTTCTTGATGACATGTTTGTGGACAGGTTTATCTGAAGGTGAAGTTATGCTGTGGTATTGTGGCGTGTTATTATCAATGTGACGAATCTTTGAAAGCACAACTCTTGGGACTCCTGAGGTGAAGCAGTAGAATCATAGCAATTTGCCTGAACCAGGATCATTACTACATTATTCTTTAGATTTATTGTTGTGACCCTGCATTTTAATGAATGGAGCGCTCATTTAACCTTGTACATTGAG

>Ca1:2854882-2855488

GCACCTGAATAATTAAAAGTTATATAGTACATAAAAAAGGAAACAAGAGGAACCTAAGTACAAGATGTGCCTAGGTTCAGCAAAAGTATGTAAAAATACAGTAAGAAGTTCTGCATAACTGTAAAAAAAAGAAATAAACTAACTAATCCCCCACGTCATGAACCAACACAAAATAACAAAACAAATGAACAATGAACCGAAAAATAACAATTGATCAGTTCCTATATCCAGCACCCGTCATTTACCTACTTCACATTTCTCATGTATAATGAGCCTATCACATCAAAAAAGAATTCATAGCAGAGGCAGCAAACAATTGAAGTAGAATAACTAGCATCCTCACAGGAACATTTTATGACTGCAACACTTCTTGATGACATGTTTGTGGACAGGTTTATCTGAAGGTGAAGTTATGCTGTGGTATTGTGGCGTGTTATTATCAATGTGACGAATCTTTGAAAGCACAACTCTTGGGACTCCTGAGGTGAAGCAGTAGAATCATAGCAATTTGCCTGAACCAGGATCATTACTACATTATTCTTTAGATTTATTGTTGTGACCCTGCATTTTAATGAATGGAGCGCTCATTTAACCTTGTACATTGAG

>Ca1:2888599-2888811

CTAGTAACGGTGGAGGAAAACAAGTGACAAACTCTGAGAACGACGAAAGGACGACAGTGGATTCCGGAGATGGTGTCGGAGACGGCGCGCGATTATTTGCTCGGCAGCGAGAGCGAGAGATAACGGAGGAAGTTGAGAGAGAACTATCTTTGTCTAGGGTGTGTGGTTGGTTTGTTAATATGTAATTATATGTCAGGGATTTTAGTATAATG

>Ca1:3230608-3230891

AAGGAGACAAGACATATAGAAGAGTTATACATTGGAGCAAATTCTAAGTGGGGGCCCGAATTAGGCTTTAGTACAGTTGCTGAATTAATTTATAAGGGCTAATAACAAAATTAAAAGCTAGCTAAAAGAATCCAAAATTACTCTTCCACAAATTTCTATTTAGAGATTCCCTCATGACAGTATCAGCTGAGATATTTACTTTCCCCTGACTATAACCAAAGCAAGATGGCATTCCGAGCAGCAGCATCATCGAACCATCGCCCACTTGGCTAGCTATACATGT

>Ca1:3240515-3240821

ATGGAATTCAATTCTGTGAAGGATAAGAACTTATATCATAAGGGTATGGAAAGTGATGGAAGCATTATGTCATGCTCTGGTGTTAAAGAATGACAGACACCAAATGAATATAGAACAAAGCAGAACACAATTGTTTTTTCCCTGTGTGAATAGAGGAATAACAGTGATCAACGAAATGAAATACTCCAATGGTAGCTAGCAACAGCATAACATGAACCATGTCTCAATCACATTCTATTATCATTATTATCCGATACAGATTTCCAATCATTTTTTTATCTTTCCATTCTTCAACTCCGAACCTTC

>Ca1:3240515-3240888

ATGGAATTCAATTCTGTGAAGGATAAGAACTTATATCATAAGGGTATGGAAAGTGATGGAAGCATTATGTCATGCTCTGGTGTTAAAGAATGACAGACACCAAATGAATATAGAACAAAGCAGAACACAATTGTTTTTTCCCTGTGTGAATAGAGGAATAACAGTGATCAACGAAATGAAATACTCCAATGGTAGCTAGCAACAGCATAACATGAACCATGTCTCAATCACATTCTATTATCATTATTATCCGATACAGATTTCCAATCATTTTTTTATCTTTCCATTCTTCAACTCCGAACCTTCCTTCATCAAAGAATTAAAAACCAAAACAAATTCAAACTCTTTACACACTTCTTGCCTTATCCCTTTC

>Ca1:3314332-3314798

CACAATTTCTCAGCCTTAAAACTGAACTTTATTCTTGAAAATAGTTACTTTGTAAGAATTAAACAACAATCCACAAATATAACCAACTACCTATGTATATAACCAAACCAGGTTTTGATCTATTTCATGGGCTAATTCCACAGAAAACCAATACTTGAAAAATTGTCTGAAATTACAAGGGTGACTAATGTCGGTTCTCAGACATAGAAGTTTAAGATCCTCCTTATCGGAACGTCTGATATAGTGACTTAATCAATGTCAACTCAACAAAAACAAGAGAAAACAAATTACGACTTTAAAACTTAGTTCATATTGCTTTCTTTTATTTTTTGTCAAAACAAACACTGCAATTAGGTCATCATCTCAATCGTGAGGGCCAAGAAGACTGTGAGTTTCTGCTTCTAAAAGGCGGCGTCAATCATCGTTGATATTGAGTAATTTTCAGGAACCGGTCCAAGGTGGTCCA

>Ca1:3515395-3515839

ATAATAAACAAAGAAGTTGCCTAGATGTAATGTAATGTAACCAACCACCACAGTACTCCGTGGGCCTAAAAAAGAGGGTAATTCATTGATTTAGTACACAACTTTCTCTGACTCTACAAACTATAAAGCAACAAAAAAGTTGTTAATTGTCACAAGATTTATGGCGGGTTTTCTCTTTTAAAACATAAAATTTTAGTTAGATGAAAGTGAGGAAACAATAGGAAACCCACGTTCCTATTTAACATTCTAACCGTATCACACATGTCTTTCACCACTCACATTATCTTCCACTTCTCTTCTTCAACCTAGTATTTACCAAAACATTGCAAACAACAAACAAACAAACACATAATCATGAAATGGGGTGGAAGAAAAACTTCAACTTCATCTTCATCTTCATCTTCTTTCATTTCTTATGCCTCTCCTTTTTCTTGGCTTTCAAAG

>Ca1:4016705-4017205

AGCAGTTGCTAAATATGACAAAGTTGAGGGCAGTAAACTGTAAGTGCAGGAATAACTATTGCATGGTATGATTGACTCATCCAGAAGTTATTTAAAATTACAGAAGCAAAAGTACATTGCTCATTTAACTTGAGAGAAATATATCACAAAAGCAACCCAAACAGATAATAACCTAGAATATTCACAGAGGGAGACCTGAAAAGGGGGTATGACTACAAACTCAAAGATCGTAATAGATCATCCTAGTTGCAGGTCTTAAAAAAGCATTGCCTCAAATTTAAAATACCAGCACATGCATTCAGAGTTTCTCAGTTCAAGAAAGATAGGTAAATGCTTTGCAGCTCTTTTAATTACTACTACTCATATGGAGGTGCAACTTGATCAGAAGCTGGAAAGAGCAAGATTCAGGTGGCCGGGCTGCTTCCAAACAAAATGTGTTTTCTGATTCCTTCTATATTAGGAACTTCATGTTCCTCTATTTTCAAATGATTCTATAATCC

>Ca1:4023591-4023986

TCCTTCTCTTGAGTTTGATGAATGTTGTATTTTACACCCAAAAATATGAATATCTCAATACATACTTATAATCAAATTATGATTTATGGTGAGAAATTGTTCCTTCTCGGGAAAACATTGTAAAATGCAAATTGACTTGTACGGTTTGTTAGAATTTTGCACAAAAATCGATTCAATGATGAAAAGCTATTTTGGTTGTGATGTAACTAGCATGTTGCAGTGTGGTCCATATTCTTGTGGATTGTGTTGTGTAACAAGTTGCCGTCCAAAAAAGGTCTTCTTGTTCAAGTAGTCTATTTTAGACTCATGAAAATGTTAATGATACATGATAATAATACACCTACAACTAATAATAATAATAATAATTTGTCACATCCACGAAATATTTAATAACA

>Ca1:4346784-4347020

AGGCAAGTAAATTAGCCTTATTATTATACATTATAGTAGCATACATTTATATAAACTCTGCTATTATACATATATCCTATAGTTTTAATACACCCTGCCGGCCTTATACTATATATAATCTCACCATTATACAAAGTAACTAACCAAAACAGATTTATTATACATAGTAACTCATTATGAGAATTTGAAATCAAACCCAAAGTGCTCCTGCTCTTCTAAGCATTGGAATCAAAGAC

>Ca1:4760433-4760799

CTAGAAAGAAGGCAACCGACAAAAAACCACTAACCACAAATAAACGATATCGTTACCTTAACTCACCTTAGGGTGTTGAAAGATCCAAAAAAAGAAGACGGAAGTGATGTGTTAAGCAACGTCCACCGTTAACGGAGGGTCCCACGTTAGCAAAGAAAGCGAGACTTGGTCTGGTCGGCAAACATCATGTGTCTGTCAAAAACATTTCTTTCTCTTTCTTCTCTCTCTCCTCAAACCCATAGCCGTACCCACCCCATTTTTGTCTCCTTCCTCTTTCACTATATATCACCCTTTTCCTACCACACCATTTTCCCCAATTCACCTTTTCTCTCATTACCATTTTCCATTTCCAATTTTCAATATTCC

>Ca1:4886032-4886567

TGCACATACAAAATAAGTTACTAGTAGATCACAATAATTGTTGGTTTCAGGGTCACAGGTTACATTTAACTAGATGTCAGACACACACACACACACAAGTTATTTCTTCATGACGAAGCAATCAATCAAAGATCAAAGGATTGATTGCCTCATAACTTACAAATAAGGCTGTATCTGTATGTATCTACAAATTGTAGTAAGTTGCATACGCACATCATCAACAACACTTATCTTTGAAGGGATTTCCACTATGACCTCATATCATCACTACCTCAATTCCTCAGCTTTTAAATTTGATTGGCGGTGAATGGGTGTGTCTACCTCTGACACTAACTACTCAGTGCAGAGCTTCATCTTCATCCACAACATGTATGTGTCGTCTTACATAACTAGTATGTGATAAGATTAGTTTTAACATTCTTATCATGGTTGTGTATATGCCCCACAATGTCATGCAGCCACATCATTCTTTCCCTCTTTGGATAATATAACCGCATGTTTTGAATTGTGTACTTGAAGTGAATCTCTCACAAGA

>Ca1:4886032-4887094

TGCACATACAAAATAAGTTACTAGTAGATCACAATAATTGTTGGTTTCAGGGTCACAGGTTACATTTAACTAGATGTCAGACACACACACACACACAAGTTATTTCTTCATGACGAAGCAATCAATCAAAGATCAAAGGATTGATTGCCTCATAACTTACAAATAAGGCTGTATCTGTATGTATCTACAAATTGTAGTAAGTTGCATACGCACATCATCAACAACACTTATCTTTGAAGGGATTTCCACTATGACCTCATATCATCACTACCTCAATTCCTCAGCTTTTAAATTTGATTGGCGGTGAATGGGTGTGTCTACCTCTGACACTAACTACTCAGTGCAGAGCTTCATCTTCATCCACAACATGTATGTGTCGTCTTACATAACTAGTATGTGATAAGATTAGTTTTAACATTCTTATCATGGTTGTGTATATGCCCCACAATGTCATGCAGCCACATCATTCTTTCCCTCTTTGGATAATATAACCGCATGTTTTGAATTGTGTACTTGAAGTGAATCTCTCACAAGACTATTAATTGAGAAAACGACAAAGCAAAATTTAGAAAATTTTATCCTGGTATATCCTTTCCTATCATGAGATGGATATGTGCTAAAGTAGTTGTAGATAAGTAACAATTTGCTTTGCTGTGTTGTAGTTTCATTATTCGTCCAAACAACCAATATATTGACTATTATTGTAAAGTTTAACATGAAGTGATCATGACTTGAGATAAACTTTGTTTATCATAATTGCTTACAAAAATATCACAAGTGTTCTGTGAAAGGTGCTAATGGGAATATTCCACTATAGAAAACTGTTTTCAACACAATAAAAAAATACAGAATAGTTTAATAGGGAATTATCATCTACTTTTTTTAATCCTAACAAATTTCTTATGTTGTTAGTTGAACTTCTCCTTAATTGAATTATTGTGGAAGATAAGTTTCCATAGTAAAATAAGGTGCGGTGGACTACACTCTTTTGGTGGTCCAGTACTTGGTTCACCAGTACATCCCTAGAAAAAATTAACTCACTGCAAGGAAAACAATTAACAC

>Ca1:4905149-4905570

ATAACATTTCCATAAACAAACATTATTTGAACTTCGAAGGTCATTGATTTGAGTTAACATTTTTCTGAAGCATTATATAAAGTTTAAAACATAATAAACTCAAAATAGCATCTCCCTCAGTATTTTTTCTATTGCAAATTCTAAACTAAACAAATCATAACAATAATATTTAGTAAAGTTCCCAATACCTTAAGAAAACTTAATATACTCGTATATACTCCCACTTCCACATATATCATGAATAAAACTTCTTTATCACACATATTCTTGGAAACAGAACATGAAGCACGCAAATAACAGGGCTGAGGTGCCGTCTCTCCAGTTACAATCTCCACAAACTAATATTGTTCTCTTCCAAATAATTGATTATGAAATTCTCTTCAATAAACTTTATGACATGGTCTGTTTTGTTTAAGCACAC

>Ca1:4954579-4954915

ATCACAATCCTAAAAACAAAAACATAGTATTACAAAACCCGTCACAGCAGAGTCCAGAAAGAGATCAGACCTATTGTAAATCAAGTATAGATATCTGAATTTATAGTATAAATCAGACCTATTGTAAATCAAGTATAAATCAAATGCACTTTTACTCAAATTACTTCAGCCGATCAAATTGCAGAGGTTTGCACAAATAACAATCCCTTCCCTTGGAGTTGGAGGAGATTTGACATTTCGAATGGGAGAAAACCACGTGCTAGAGCAAAGTTTGCAATCCAACCCCTCCTTCCTCTAATAACACCTTGAATTTCAATGAAGTTCATCCCCAATTTC

>Ca1:4954579-4954919

ATCACAATCCTAAAAACAAAAACATAGTATTACAAAACCCGTCACAGCAGAGTCCAGAAAGAGATCAGACCTATTGTAAATCAAGTATAGATATCTGAATTTATAGTATAAATCAGACCTATTGTAAATCAAGTATAAATCAAATGCACTTTTACTCAAATTACTTCAGCCGATCAAATTGCAGAGGTTTGCACAAATAACAATCCCTTCCCTTGGAGTTGGAGGAGATTTGACATTTCGAATGGGAGAAAACCACGTGCTAGAGCAAAGTTTGCAATCCAACCCCTCCTTCCTCTAATAACACCTTGAATTTCAATGAAGTTCATCCCCAATTTCCTAA

>Ca1:5038663-5038944

CAGCTTATGAAATGGAATAACGATGAACCACATGTTATTTGGAGTCAATTCAGTAAGATCCTTCAAAAGTTTATAGACACTCCTGGACAGTGCAGATACAATACAAGGAAGGATTGTTAAAACTACATTGCTATTTTCTTTCTTAAAAAATTGTTCTGAAATGAAACTTTTCAATTAATGGGTAACCATGAGAATGATAATGGTTTAAGTATAAATGAGATGCCAGCACAGTTGATCAACGATGATCTCCTCCTCCATCAAGAGTCGCATTGGGGAGAGAC

>Ca1:5038663-5039062

CAGCTTATGAAATGGAATAACGATGAACCACATGTTATTTGGAGTCAATTCAGTAAGATCCTTCAAAAGTTTATAGACACTCCTGGACAGTGCAGATACAATACAAGGAAGGATTGTTAAAACTACATTGCTATTTTCTTTCTTAAAAAATTGTTCTGAAATGAAACTTTTCAATTAATGGGTAACCATGAGAATGATAATGGTTTAAGTATAAATGAGATGCCAGCACAGTTGATCAACGATGATCTCCTCCTCCATCAAGAGTCGCATTGGGGAGAGACCTGCTCAAAGCATTAAAATCAGAGGTTCAGAGTTACCATTCAGAGAAGATATGTTAAAGTCCAACGAGCATATCTTCAAGTCTTCAATAAATTTGAATTAGTGGATGGCCAATCACTT

>Ca1:5729535-5729870

GTGAATCGTCCTGCAGCAAAGAATGTGCCTTCATTCGCTCTGATCGAACAGAAGCTTGGGCAACATATTAATATGCATGGAAATCAGAAAGAAGGCAGAAAACTCATTTGCATGGTTCTCGATGAATTGCATGCAAACTCCAGTGTGTTAGCATGGTTATAATGATTAAAAAATCAGAGTTAGACCATTATGGTTGGGCAATTTGTAATACAAATAGATAATCAACAGACTTCCAATACTTAATGGGATACTTAAGAATTTCAATCACATGTTAAGTTCATATAAGGTGCCCCTTTCTAAAGCTAATTTTTAGTTTGTGAGTAAATCAATAAAAC

>Ca1:6657261-6657505

GTAGCTAATATAACCAACATTCAACTAATCATTAATAATAACTATCTACTAATTGTAACAATTATATGGTAGTACTAGTGTCTAGTAGCATTTGACAACTACATAATTCACTCACTTTGTCATTAATATTTGCAGTACTTGAACCACTCTTTCATCACTACTCAATTTCCCTTCACTAGCCAGGCCCCCATGAACTCCATCAATGCATTGTCTTCCCACCCCAATTCCAATCAAACATGGACCT

>Ca1:7025626-7025868

CACAGACAAATTTTGGTTTCTCTTCTTCCAATGTTCATTTTTCATTCTTTTAATAAGAAAGTGATCATGAACAAATTGTTCACCCTAATAAAATATCCATGAACCTTAGGTCAAAGATATCAAAGTGAAGAACATAAAAGAAGTCACATGACATTGTTATTACACCTAATGAGAAAAATGAGCAGAGTGAGAAACTCTTTATCCATGTGATTGTATGAAGTTGATGAACTAACTATCTGCAT

>Ca1:7069085-7069292

AGAGCCACCAAAACAGCATAATCAAACCAAACAGAAAATTGATCCAAAATTGAAGAAAAGAAATTCAAAATAAAAATAAAAAAATTTTGAAACCCGAGGGTAGAAAACAAAAGAGTGAATATTTTTGATGAGATCGATGAAAATGAGAAAAAGGTTGGAAGTTTGAAAGAAGCATGGAAAACGAGAGAGAAAGAGGGAAGTCAAAAG

>Ca1:7078433-7078858

GTATGTATTGGGGCGGGGTGAACAATTTTCTATTGAAATGGACAAAGCTACATATGTTTCATAAATTACACAACAATGATAAAATGTCAAGCAAGCAATCAAAAAACAAACTACAAATTAAAATAAACCATCATGAAGAACAAAATTCATAAAGGTTGGTTAAGTATTTTGATGTATTCATGAGACGTTTATTTTGCAACCGAGAACAGTATCATTGGAGTGAATGCATTTTATGTAATTTAGAGTATCTAGGACGTAACAGCTCTTTAGAAAATGAAGTCTCCAAGTGATAACATATAACATGTTCTTATCGCCCTCATCTCACAGGAAAATGCAGCATTTCTACTGAACCACCAAAGTTCTAGCATATGACTACGACGAAGAATTCAGATTCTTTAGCTTGGCTTTGGCTGAATCAAGTTGTT

>Ca1:7790883-7791341

ATATGAGAACTTTAACATATATAAAGCAATCATGGAGTATATATAAGAGCAATATAGCAAAGTGCACTAATTGCTATAAGATTGCTTACACAGATTGGTAACTTATTGTGAATATAGGATACAATAATATTATTGCCAAAGGATCAGAGGGCTGCCCTTCTAAGTTAAGATCTTGACCTAATTTGTAGGTCTAACATGTAGCACCATTTTGCCTTTTATTTTTCTTCTTAATTAGCTTCCATTTTTGATGCAAGTGCTGTGGATGTTGAGATCTGAACTTGAGATTCATTTTGAGATATGTGGCTGAAAGAAATAAATTAAAGGTTAATTGATGCATATATAAATGCAACACTTTGGTGATGAGAGCAAAAAGATGAGAATAATAGTGGTGCTCAAGTGAGGGAAACATGTTAGTTTTTTGTAGTGGCTTTTGTAGGTGAAAAAGAAAGTGGAGGAAA

>Ca1:8779728-8780123

GCATATAATTTGTTGAAAGGTCTCAATTAAGATATAGTAGTATAGAATAAAAAATTGTTGCTAAAGAGTTGCCTAATTTTCATCATTGGACGTATGAGTTATTATATAAGTATGACGTGGTAGAGACCACTTAATAGAAATGTATGAAACCCAAAATAGATTTATTAAACAATGACCATTGGATAGGAGATGCTCTAATATTGAAGGGTTACAATATCACACCCGATATTAACGGAGTTCATTCTGCATCCAAGGTTTAGAAGCTGTATTTTCTACTTTAGTTGTTTGGAATCGAATGGGTGAAGAAAGCAACAAATTCAATATCGCAAGTTACATCTTAGCATGGTGTCCACATCGTGAAGGGCCAAAGCTAATGATTTGAATTTGCAATATAC

>Ca1:8793207-8793745

GTACAAAGAAAAAAACAAAGATGCAATATATGTTGATGTTATTGTACCCATGTACAGCAGAAAATTGTATTTCTGTTTTAGTCTAAAGCAAATGGACAAGTTAAGAATATAATGTTTATATCCCAACTTGAACCAGAACTATCTATATCTTATTCGGAGCACACAAATATAATTACAATGCTAGTAGCAAAAACTAGTAGCACAAAATGCAGAAACTGAGACAAGAACTAATGTTACTCGAAATAATGCTTATTAGGGAGACCATATGCCGGTAACATCAGGGGGTGTCCAACAAATAGCAGCTAAGAATAACCATGCAAGCAAGCCTCAGATAACTTCTATCATTAAAATATTCTACCAGAGGACTTCAGTCTTGTGAGGTTCATTTGTGTATCATCACCAATAAATTAGCACTAGCACAAGCAGAAGGACTTCAGTCATGAAAGGTTTCCATCATCAATTCACTGAACTGGGAATAGGGGACCATTCTACTAACCATAACACAACTCTTTTTCACTGTGCTTGAGGTCCTCAAATA

>Ca1:8871881-8872135

CTCATATAGTTGACGTTTCTGATTCTTTAAATTTAAGGTGGTTGATTTTCCATTCTTAAACCAACACATTTTTGGTCAAATATTTCAAACACCAGTTTGGAGATTTACTCTTAACATAACAATACTCAACGGAATGCTCCCGATTTCCATCCATCAAATGATTGAACACGATCCTCTGCAGCAGTCCAATTGGTGTAATGAAATCTCAGCCGCTAGATTCGGATTGCAGCTGGATCATATGCACAACTAGGCTT

>Ca1:9259372-9259609

CCCATAATTTGTTTAAAATAAAAAGAGATAACACCGAAAACATCAATACTATAAAAGATGCAGCAACTTTGCTTAATTGGAGATAAACAATGGACTCAAAGGACATAGTGAGTGCTTTAGCCATGATGGGTGAACAACATCTGAAGGAAGATAAAACCTTTTTTTAGTAACTCAATTTATTGCAATGGTTGCTTGTATGCAGGGATGTGGTCGTCGTCGTCCGCGTTTACTTTAGTA

>Ca1:10122910-10123205

GGGAAAATGATTGTTGTGATTTTCAACAATATAAAATGAAACACAACTGCCCATTTTCTCAAATCAATATTGCAAGGGAACACTTTCAGAAGAGTGCATTGATAATAGCACAACACTGATACACACTATCCCTGCTTTCTCAAAAAATAGCATATACTTTTATCGCTGATCAAATAAATTTATAAACTAATAACAACAGAAACAATAAGATCTAACATAAACGAATCAATTTAACAAAAAATTATTCAATACCAACGAATCAAATTGTAACGAAGAAGAAACTATTAGTTGATTG

>Ca1:10905044-10905258

CAGGCGTTCTCCTTGAACCTTCATATTTGCTTCTCTTTCTATCATATTCATAGTCATAATACCTACTACGTGAATCTCTTTCTGAATAAGACTCCTTATTTTCATAATCACTTCTATGTCGGACCCGCCGGCTATTACTTCTTGAATCATCCCTAGAGTGCCTCCTCTCGCTCCTATGATCATCCCTGTCATATCCAGAAGGTGAAGCAGAAAC

>Ca1:11055129-11055485

CTTTGAAGATTTGGGATCGACGAATAGAGAGAGAAAGCAAAGACGGAGAAGGAGGAGAGAGAAAGAAATGGAAAGGAGTGCTGTCAGTATCTGTTTCTCTCTCCTAATTTTTTTTGCGTTCGAAAAGGCTTTCTGTCCTTGTGTTGCCAACGAACGCTCTTTACTGATTGGAGGCGACGTGGGAGATGGTTATTGGGTGTGAGTTTTATTGTGTATTTCCGATATTGTCCCATGAGGAATTTCTATTACCGGTTTTGTCAGCGTAGAAGTGTTTGGGAGACATGGGCATGATGGTCCACCTAACTACTCATTTATTATTGGGTGCTTGTAATGTTTAAATGGAAGATTTGGTGAGG

>Ca1:11283632-11284059

GTGGTGATGAAATAAAATTTCAAAGACAGAAACAAGAAAGAATTGTCATTGTAAAACTAACTGTAAAATAGTCATCTATTTCCAAAGTAAACAAACTTGTCAACTTAGTATATAGTCTATTTCACAACAGATCCTACAAAACCTTTTCTTTCACCAACTTATTTATAGACATACTGGTCAGAGAAGGAAAAAATCTTTAAAAAAATAAACATTTTGATTGCTATTATTAATGCAGGCATGCTAAATTGCCAACAGCCTAAAAAGGACACTATTACTATATCAGGCACTCTTGTAACTGCGGGATTTGATGCGAATCTGGCAAGAAGTTCTGCCACAATTATATGGAGTTAATTGGTGCAAAGCTGGGAATAATATTCATCGTGCACCTGAAGGTTAAGCAAATAGAGCATAGCAGCCATATCTGAAC

>Ca1:11445785-11446126

CATGCAGCTTCGAGTGAAGAAGGGGTATATCACTTCCTTCCATATTTATTGAAACTCACGTTTCAAAGATATATTAGTACTTTTTTTAGACCCCCCAAACACACGTCTAACTTAATGGAAAATTTTCTTGAAAGGTGAAAGTTTTACCTTACAACTTTCTTTTTTTTTCAAGCTTAATGACTTATGAGTTGTGTCAATTGACAATTTATTTATCATCAATAACGCGGAAACCCAGAAGCTAGAGCCACAAAAATTAGATGTAGCTAAGGTTTATTAATGTAAACAAAGTATCACTATCTAAGGAACCGAAATTAAATATAATTTTTTTGAAAAAGGATAAG

>Ca1:11445932-11446280

CTTACAACTTTCTTTTTTTTTCAAGCTTAATGACTTATGAGTTGTGTCAATTGACAATTTATTTATCATCAATAACGCGGAAACCCAGAAGCTAGAGCCACAAAAATTAGATGTAGCTAAGGTTTATTAATGTAAACAAAGTATCACTATCTAAGGAACCGAAATTAAATATAATTTTTTTGAAAAAGGATAAGTTAAAAAAGAGAGAGCTTTTGAGGTGTTGTGTGATAAGGAATACGTTTTAAGGAAAAGAAGGGTTGTAGGGTGTGGCAAATTTTTGGAAATTTTGGTTGGAGTGTAAACAAGTACAATTGGAGTTGGAGAGCAAGTCACATGGGTTGGTAAAGG

>Ca1:11524498-11524757

CCGCAGTCAAAAATAATTATAGCATTGTAATTTCCAAAATGGATAATCATATATTTGTACAGTATAATGTCCCAAATTGGATGTTTGAAGAATAAAACAGAGGAGCTTTAAGCAAGAACATAGAGCGGATGTACTTTGATCGTGTGTGATTTGACATCTGAAAATTGAATTGATCTTAAGATTTGAGAATGGCAAACATTACATATCACGAGTCCTTTGATTTTGCAGGTTCAACGAAAATCAGCCTTCCATTAACTAT

>Ca1:11672604-11673031

ACCACTAATTTACCAACATTGGCCTATCAAAACAAATAATGATCATCAGAGGAAAAAAAAACCTAATCCGTTTTATTTCCCTATTTGGGGGCCATATTCCTCGACAGAACATATAAACATAAACTATATTTATGTATAGGGCAGCTACTAGACACTGCTAGGAACAGCTACGTTACCCCCACGTCTACTCTTATAACTATAATTCATATTTGGGCCCAAGAGATAACAGCTCTAAGTGCATCGAGATTTGATAAAATCTAAGAGAAACGCCAAATTATAGTTACACTAATACTGTAAATTGGCTTCCATCATAACAAATTATATCCATACATAAAAAGCTTTACACTGAAGGTTTTGTATCCTCTCCTCAATGCTGTGGCAAAGACGGTCTCTTTTCCACCATTTCATAATGAAGAACAAAATTATC

>Ca1:11678459-11678662

CGGAAAACACGACCTGCGGAATTTTGAAGAAGAAAGAGAGAACGCTGTGAGCTTTCGACGAGTGGGATGCGGCGGGCAGAGCAGAATAGATAGATGGGAAATTGGGAAATGGGAGATTAGGGCAGACACTGATTTAACTTTGGGCTATCATTTTTTCTGTTTTGGGGACATCTGTTTCAATAATTAAAATGGTTTATCTTTAT

>Ca1:11678459-11678668

CGGAAAACACGACCTGCGGAATTTTGAAGAAGAAAGAGAGAACGCTGTGAGCTTTCGACGAGTGGGATGCGGCGGGCAGAGCAGAATAGATAGATGGGAAATTGGGAAATGGGAGATTAGGGCAGACACTGATTTAACTTTGGGCTATCATTTTTTCTGTTTTGGGGACATCTGTTTCAATAATTAAAATGGTTTATCTTTATAGAATA

>Ca1:11678472-11678685

CTGCGGAATTTTGAAGAAGAAAGAGAGAACGCTGTGAGCTTTCGACGAGTGGGATGCGGCGGGCAGAGCAGAATAGATAGATGGGAAATTGGGAAATGGGAGATTAGGGCAGACACTGATTTAACTTTGGGCTATCATTTTTTCTGTTTTGGGGACATCTGTTTCAATAATTAAAATGGTTTATCTTTATAGAATAATTTTTTTTTTTTTTAA

>Ca1:11748581-11748983

CTGACACAAAGGTAATTAATATTATGTCCTATTCATTTCGATTGACAGATTTATTGGATGTACTACCAACATCATGTCTTAATTGAACCGTGTATATATATACACATAAAATAAGGAATTTGATATCAATACAATAAATAGCGCCGCTTGAGCTTCTTGATCCATCACACAAGAAGCAAAATGATATCATTTTATATCGAGACCAAAAAGTATTATTATTTATGAAAAGTAAAGCCACAAAAACATTCAGCTAATCTAACCAGATTCCATGATCTTCAACAAGATCACATTCTATCCTATTCTTTCAGACCGAATCAATATACAGAGATACTGCACTGCATTATACACAAGCACAGCTACAACCAGTGGGTATTGAACTCTTGAATGCGGATGGACCGATGC

>Ca1:11753696-11754089

CTGAAGAATAGCGAGCGTGGATGAAATTGAAGATTGAAGGAGAGAGAAGGGAGTATAAAAAAACATGGAAGGAAGAAGAAGATTGAAGAGAGTGGCGCAGTTTGATTGAGAATGAGAAAACGGAACCCTAAAAGGGTGAAAAAATGGTTATATAGTGAAAGAGAAAAGAAAGGGGTGAGGCAGAAAGGGAAGGTGGGGAGGGTATGAAGAAGACGAAGGTGACGTGGGAAGAGATGGACCGTGTGATTGGATTTGGAAAAAGGGAGAGTGTAAAAAGACGAAAAGGGCCACAGCAACAAACAGAGAGATGAACGCAATGTTATGTTAATACTTATGCAGAGAGAGAAAGAAGCAACGAAAAGTGAGAAAAAAAAGGGTTTGTGTGTTTTAGAG

>Ca1:11768827-11769192

CTCAAGGTGTGTTGTGCTGATATATATTCTGTTACTTGATCTGATTTCTGAAGCAGCACGCTTGAATGGCTTGATATAATTACTACTATAAAGTTGATTTTTCTAAAAATGGCAAAAGAAAAAGCTGTCGGAATCTAAATCATTGTAAAAGAAAATATTTAACCAATTCCCATCTCTGTGTGCTGTTATTCACATGGCTCCTGCATTGATTCCCATCATTGAGACATCAAATTCAAGACCTTACTTTCAAGAAACAGAGAAAAAAAAGTGACCAATTTTAAGTTTCTATAGACTTCAATTATGATTTGTGTTGTAAGAGAGAGGAATATGAAAGTGAGTTGGAAAGAAGGAAAAGCAATAAAACC

>Ca1:12275475-12275729

CTGAAAGTGAAAGTGAAGAGAAGATGAAGATCTGAGAGAAATGAAAGGAATAGCGTCAAAGCATGTTGGCGACAAGAGAAGCTTGCAAGTTTGGACAGAAAAATGAAGTGTGTGGTACTTTATTATTATGTGATTGTGAGAACAGAGAGAACACCTCAAACTTCTTTTATATAAATTATTTTAATTATTAATATTACAACTCATACAATTCTTTTACTTTGCTTATTTCACCCTACATTTTACCCTAATTTCAT

>Ca1:12543555-12543898

ATATATACAACTAATAAATGAATAACATTATAAAAATAAGTACACTTGCCCAATTGCTCAAGTTGTCTTGGGAAAGACCATAATGTAAAAAAATAGTAACACATGTTGCTATTGTTTTTGGAAATGGTGGTCCCTCTATTTTGTAGACAGGAAACCTCGGTGTCTCCCTTCATTTCGTATATGTTATGCCAATGCCAATGCCAGTGACAGTAAAAAGATGGATCACGCGCCATTGTCTGTCTACAATGCCAGCACATGTTTTACATGATCCGAATGCCCAACTGTTGCTTTACTATATTTTACCATACTAATCACGTACAAATTACCATACTCAAGGCAGAAG

>Ca1:12758774-12759055

NNAACGCTTCGGTGTTTCTCTCTTTCTCTCTCTGCAAACGTGATATCCTCTTATTGCATCACAGATGTACGGCAACGGAAAAGTTCTTCTGTGGAACCGTGAAACGGAACGCGTCTGGGGGTGTACCACAAAGTCTAACCCGCGAACGATAAATGCATCAAATTTTGGGGAAAATAACTCACGCTACCTTTTGGAGCGTGTAATGCACGTACGAATAAGATGTTGTTTGGCTTTGGTGTTGTTGGTAACGCGGCACTTGAAGGGAGAGAGTCTATTGTGGC

>Ca1:12799213-12799606

ATAATAAATAAACTTTTTTCTAGTCTACATGTACATTCCAAATCGTTCACAACAAATTAAACTGCTCAAATTGTGGAAATGCTTAACAAGACCTGTACGTACGTCTTAATTATGATCATGAAGATACATAAATCTTGTCTTGCAAAAACTCGCAAGTAAATAAATATGCAATGGAAATGTCTCTAAAATCTAAACAGAAATCGACATGCATAAATAAATAAAAATAAACAAGACATTAAGAACTAATATATATTGTTTATGATATAATAAATATCAATGCATGCTAACTAATGCTGTCATTCCCCATATACTATAAGAACCCAGCCATGACGACAACTATAACATTAAGCCTTGTTGTCTTGTGCAATCTATGGATAACTGCAAGTGCATGTC

>Ca1:12819199-12819615

ATAACTTGGTCAAAATTACACAACATATGTACCATAATCCAAACTATATATGCACATGTAATAATGAAAGAAACATAACCTAGTTAAAGAAACATAATTCACATTACTAAAACACATTTTACACAAGTAGACAACATCAGTGTCAAGTAGAACATTGCTTACTAACAAATTTCATTAACTTCATGGCGACTACCAAACTATCAATCTTCTTATTCAGATCAACCTTCCACAAATCTTCTATTGCATTATCTCTAGCATTTGTCATCACAATTTGCCTTCCGGTATCTATTCTACAATTCCCTCCATAAATAAAATGTTCTTAAACATCTTTCCAAATCAAATAAGAGTACTCTAAACGATTATCCTGAAAATTTTGAAAAAATTAAAATAGTTTCATCAGAACAAAGAAAGATATA

>Ca1:12821598-12822105

CTTCAGAATTACTTTTATCTATTCAACAAATTTGTGTCATCTTGATATAAATCCTACTTAGCTGAAATAACAACAAATTGCGACAAGCAAAATACAACAAACAATAAAGGATTGGATTGCTTCTCAAATTCTCAATCTCAAACAACACATGCTCAGAGTATAATAAAGGCATGCTAAAAAAAACAGTCTACAAAGTTTAGCAGGGAATCAAAAATATGTACGTAGTTTTTTTCTACACTATTGTCAATTGGAAAAGATAACTAAACACCGGAATAATAACTAGGGTGTTAAAGCCTTTTCCACAAGAACAACAATTTACGTTACAAATTGAGCGATGTTACGTGTTGAGAATATGTCTCCTGGTCATGTTTTGTATTCAGTGATTTGAGATGCCATCTTGTGAAAAATTGATGTCAGATCCAGAGGGTAAAAACAAAGGATATTGGTAAAGAGTCTCTATATGACAAAATGCTTTTATTTTCATCCTTGGAAGAGAATTCAACTAAT

>Ca1:12895206-12895521

CTCATTATAAAAACTGGCCCAACCTGCCAATCATACATCATGTTTTGTTGTATCATTGTTCTCAAACACATAGCATTCGCAATTAGCCACATAAAGTATAAGGCTAATACTAAATCACAAATTCATCAGCAACATAAATCACAGTTACATGACAACACAACCAATACAAATTACATCCTTAAGTATAAATTACATCCTTAAGTATAAATTACATCGTGATCAACTTAATTAAAATTCGACATTAACCAAAGGAAGACAATATTGCTGCATCATGAGAATTTACTAGTAATTTCAGCAGCATGAATATTTTTTCTT

>Ca1:13471054-13471753

GGCTAAAGAGCATAATAGTTATGAAAATTAATAGTGTAGATACCAGGCAGTTTCTAATTTAATGAGGGGCTGAATTGGAAATACTGCAATTTGGGATATAACTAATAATAGTTATATATAGGGGTAATGGTCCCCAAGGCAAACACAACAAGACTATTCAGTTTCAAAACCCTAAAGTAGAAAATTATCTGGTTCTCTCTATCCCCATCAAGAGAGCAAGGTCTTCAGGGTGTTTTGCTGAGGAAGATCACCCAACCCATTGGCTCTATCATCATCATCCCAGGATTTCATTAATGGCAATTCCCACAGGTACGCTTCCGCCTTTGGCTATTTATCATGTAATTGACATGGATTGTTGAGCACAACGTTAGTAAGGTTTTTCCAACATTAACTTCCTTACAATGTTTTGATCATAAGATAAGATGAATCATATCATATATACTAATAAATCCTATGCACAAATTTCTATTTCAGGAAGCAACAAAAGGATGATTCATTTCATTCACTTATTTGTTTCAGACAATGAATCTTGATCAAAAGCTTTTCCCTTATTTGCTCATCTAATTCCTTCAATAATACATTCAATTGAATATTGAATAATCAAAATATTTTGTACATAGATCTTAATTGGGACCCCAACAACTCAAGTACATAACAGTTCTCAAAGATTCCTCTGATTGTTTTGCATTCTGTTTTTGT

>Ca1:13568099-13568434

GACCCTTTTTGTCACAAAACTCAAAAGCATCTAACTTAAGCATAGCTTTAAAAAAAAATTAATCTTTGTATTTTTTTTTATTGATACCTGAGAAGGGGTTGCAGGGGATAGAGTGGTGGATGACATGGTTGAAGCTGTTGAGGACAAAAAAATTGTTGGTTTGAGATTGAAAGGAAAATGTAATGTAATAATATTAAGATGATGTTGGGTTTAAAATCTTAAATGACAACCACAACTGTCGATATGATAGTTAAATGGATAAGGTGCTAAGGCCATGTAGGTGCCACATAGCCACATATTTTTCATCCAACAAGAACCAGCAACTTCAACTTGAG

>Ca1:13592850-13593741

TGGCAGTGACTAATTAAAGATCAGAGAAGAGAAGATTGTTGAGCCTAAAAAGAAATTACATCAGTCATATTAGTGTAGTAATTCAATGAGCTAATGATATTTCCTTTAAAACGAAATTACAACTGCTAACCATTTTACTCAACATCATCATCATCTGCAGACAATGAGTTCAAGGTGATGCAAGGTATGGTCCCTTCCAGCTAGTGCAAGATGAGAGTGGTGTTTTCCCAAGTTCAGTTCCATACTTAGCTATCATGAAAAAATTGACACAAGTTCTTTTACTATATATTAATCTATTATTATAGCATTTATAGAACAAACTGAATATGTCACCAACTATTTCTTTTCAAGCCAACAGTACTACTACAACAATCCAATATTTGTTTGTGAAATTTTCACTCATGAAATTTCCACATCTAAATTTCATCTCATAATCTATTGTGGGCCACCAAAATACAATCTTAGTTAAATTTTCACATATGCAATTTCCACACCTAAATTTCATGTATCAACCTTGGTAAGGGAATTTGTGTTATATCTAGGACAGCAGCCAATAATCCCTCCCTATATAAACTAGATCATTCACCTGAAATTAATTTTCATAAAATCTTCATTTCTATATTCTTTCAAACTCAATGTAGCAAATTGCACCAATTTTAAATTATAAATACATAGTTTAGATAAGATATGATAACAAACATTTAACGGGCATAGAGAATGTGAACATAACTTGATTAGGACATTACAACAAACAGTTTGCAGGCAAATGAAGCATAAATTAAAAACTTCGATTCAACTTATCTGTGATGAGAAAGAGCACAAGCACGATGAACATTGATACTTCACCATGTGAGTAAAGCTTTTCATCCGTGGACTTTCTTAACCTGCTAG

>Ca1:13595254-13595641

CAATTGTCCTGTAATTTCTCATATATGTGTAAACATAATTGGAATTACATGTATCCTGAAATTCTGTCTATATAACTAAATGAAATTTTGATTACATGTATCCTGAAATTCTTTATCTAAATGAAATTTTAAAAAATCTTGAATTTCTCTATTTAAAAAGAAAAATCTAGCCTCTTCACAACTCTGCACAATAGTTGACGCAAAAAGTTGGATTCTTCTTCCATTCATCCTTCTTTTGACTGTTTCACTGCAATCAAATTTTCTATAGTATCTACATTGAAGACAGAGCCATGTTCAATACAAGGAAGCCATGGTCCTGGCTTTTTCTTCTCAACATCCTCTTCTCTTTACACTCCTATACTTCTGTTGCAGCTCTAACTACCATCT

>Ca1:13681208-13681449

CTGAGTGGAGATTGAAAATATTTGGGTAGGGGAAATTGGAAACGGTCCTTAGGGAGCAGGAAAGTTAAAAGAATGAATGTGAATATGATGGGTGCATATTGGGATGTGAAAAATGACGAAGAAAACTGAGGTGGGAGTTTTCCAAGACAACAAGACAAAACAGCACCGACAACAGACTAGGGTTCTTGGAGCCGCTAATCATCGTTTTCGAGACTAGACGTGTATGGATTGTTTGGTTTTG

>Ca1:13843162-13843996

GATAATGTCATTATTTAAATTTTCTTTGAACAAAATATTAAAGAAATTGAGAAAATTTTATAGATTTTATTACAGATAGCCACTTCACATGTGTTATTCTTTGAGCTTTTATCCCTCTTCCTTGATCTCGCCCATGGCCTCTTTCACTTTTTTCTTCTCTTCCTCACTAGTCCTCTAACTCTCCACCTTTTCCTTTCTTTTTCCTTTTCTCTCTCTTTTTTTAGCCATATAAAAGAACAAATTTCAATTTAACGAGAATGCCCAAGTTGATGAGCATGTCATAAAATCATCCCAAGTCCCAAGTTGATGCTCATGTCGTAAAATCATCTCATAATATAAAGTATTTAATGAGAATGCCACTATGTACAATTTCATAAACAATGAGTATTGCACATAATATATACAGACAAACGTAAATGCTAACCATTAAACTAGGTAAAAATCTAAATATATGATTTTATATACGTTTGATCTAATCAAAATAATAGCTACCTATACCTTCACTTATTATTTCAATGATATTTGATAGCTACGTATACCTTCACTTGTTATTTCAATGATATTTGATTGTAGACATCATCAACATAGTACCAGTAGATGGCAAACTAGGATTGGAAAAGTTGCATACAAACAACTGTAAACAAATAACTGTATATATATGCATAAACAAATATCTGTATATATACATACTAAATTAACATAGACAAATAACATACTAAATTAAAATTAAGTTATGCTAATTGATAAAAAGGAATTATCTAATCCAATATTCTATAACTATATATATGCATGTTCACTAAACTGCATATCCACACATGCACATAGTTCTTTTAC

>Ca1:13845069-13845284

CTTCCAATCTTGTACGGTCAATAGGATGGACGCGCCATCTCGACTCGAGAGAGGGTTACACAAGATTTTAGAGCAATTGGGAGCTGGGTGGGAACGCTGTGGATGCGTGATTTGGATGAAATATTGGGAGGATTTTGGAATTTGGGGAGAAAAAAATATGGGGTTTTAGTTGACCCAATTATTCTTAACACCTTATTTGATCTCTTTCTTAAAAC

>Ca1:13843162-13844527

GATAATGTCATTATTTAAATTTTCTTTGAACAAAATATTAAAGAAATTGAGAAAATTTTATAGATTTTATTACAGATAGCCACTTCACATGTGTTATTCTTTGAGCTTTTATCCCTCTTCCTTGATCTCGCCCATGGCCTCTTTCACTTTTTTCTTCTCTTCCTCACTAGTCCTCTAACTCTCCACCTTTTCCTTTCTTTTTCCTTTTCTCTCTCTTTTTTTAGCCATATAAAAGAACAAATTTCAATTTAACGAGAATGCCCAAGTTGATGAGCATGTCATAAAATCATCCCAAGTCCCAAGTTGATGCTCATGTCGTAAAATCATCTCATAATATAAAGTATTTAATGAGAATGCCACTATGTACAATTTCATAAACAATGAGTATTGCACATAATATATACAGACAAACGTAAATGCTAACCATTAAACTAGGTAAAAATCTAAATATATGATTTTATATACGTTTGATCTAATCAAAATAATAGCTACCTATACCTTCACTTATTATTTCAATGATATTTGATAGCTACGTATACCTTCACTTGTTATTTCAATGATATTTGATTGTAGACATCATCAACATAGTACCAGTAGATGGCAAACTAGGATTGGAAAAGTTGCATACAAACAACTGTAAACAAATAACTGTATATATATGCATAAACAAATATCTGTATATATACATACTAAATTAACATAGACAAATAACATACTAAATTAAAATTAAGTTATGCTAATTGATAAAAAGGAATTATCTAATCCAATATTCTATAACTATATATATGCATGTTCACTAAACTGCATATCCACACATGCACATAGTTCTTTTACCTGTAATATATGCTATTATTGTTAATCTCATACAGCGGGAGAGCCGCTATTGTAAAAACTAAAAATCAGCTTACAGAATAAAAATCAATCCCAATGGGATACATAAATACTCAAAAATAGTAAAATAAACAAAATTAAACGGGATAGTCATACATAAAAATTATAATTTTTATTTATTTATCACCAACTTTTAAACATCATATTTAATAATAACAATAATTACTATTATAATTCTAAAATTAATCAAAACAGAGAAAGAGGTAAAACTGAAAACAGAGGTTGGTATTTTAAATGATGAGGCCATTGTTGAGTGCAAGCAGAGGTGAACTTTAGGAGCAGAATAGAGAGGAAGCAATACACCGGAGTAGAAAGGACAAGAATGTGAACACAGAAGGAACGGAGAAACAAAATGGAGACTAGCAATGCAAAACCTCGAAGGTGGTTCGGTGGCCCAAAGCGAGGCACCGGACCACTGCACTCTGACACAATTCCGCTATAGCATGCCATCACAGCGCTATTGACAACATAGAT

>Ca1:13843162-13844539

GATAATGTCATTATTTAAATTTTCTTTGAACAAAATATTAAAGAAATTGAGAAAATTTTATAGATTTTATTACAGATAGCCACTTCACATGTGTTATTCTTTGAGCTTTTATCCCTCTTCCTTGATCTCGCCCATGGCCTCTTTCACTTTTTTCTTCTCTTCCTCACTAGTCCTCTAACTCTCCACCTTTTCCTTTCTTTTTCCTTTTCTCTCTCTTTTTTTAGCCATATAAAAGAACAAATTTCAATTTAACGAGAATGCCCAAGTTGATGAGCATGTCATAAAATCATCCCAAGTCCCAAGTTGATGCTCATGTCGTAAAATCATCTCATAATATAAAGTATTTAATGAGAATGCCACTATGTACAATTTCATAAACAATGAGTATTGCACATAATATATACAGACAAACGTAAATGCTAACCATTAAACTAGGTAAAAATCTAAATATATGATTTTATATACGTTTGATCTAATCAAAATAATAGCTACCTATACCTTCACTTATTATTTCAATGATATTTGATAGCTACGTATACCTTCACTTGTTATTTCAATGATATTTGATTGTAGACATCATCAACATAGTACCAGTAGATGGCAAACTAGGATTGGAAAAGTTGCATACAAACAACTGTAAACAAATAACTGTATATATATGCATAAACAAATATCTGTATATATACATACTAAATTAACATAGACAAATAACATACTAAATTAAAATTAAGTTATGCTAATTGATAAAAAGGAATTATCTAATCCAATATTCTATAACTATATATATGCATGTTCACTAAACTGCATATCCACACATGCACATAGTTCTTTTACCTGTAATATATGCTATTATTGTTAATCTCATACAGCGGGAGAGCCGCTATTGTAAAAACTAAAAATCAGCTTACAGAATAAAAATCAATCCCAATGGGATACATAAATACTCAAAAATAGTAAAATAAACAAAATTAAACGGGATAGTCATACATAAAAATTATAATTTTTATTTATTTATCACCAACTTTTAAACATCATATTTAATAATAACAATAATTACTATTATAATTCTAAAATTAATCAAAACAGAGAAAGAGGTAAAACTGAAAACAGAGGTTGGTATTTTAAATGATGAGGCCATTGTTGAGTGCAAGCAGAGGTGAACTTTAGGAGCAGAATAGAGAGGAAGCAATACACCGGAGTAGAAAGGACAAGAATGTGAACACAGAAGGAACGGAGAAACAAAATGGAGACTAGCAATGCAAAACCTCGAAGGTGGTTCGGTGGCCCAAAGCGAGGCACCGGACCACTGCACTCTGACACAATTCCGCTATAGCATGCCATCACAGCGCTATTGACAACATAGATCTTAAAATGTAC

>Ca1:13843162-13843376

GATAATGTCATTATTTAAATTTTCTTTGAACAAAATATTAAAGAAATTGAGAAAATTTTATAGATTTTATTACAGATAGCCACTTCACATGTGTTATTCTTTGAGCTTTTATCCCTCTTCCTTGATCTCGCCCATGGCCTCTTTCACTTTTTTCTTCTCTTCCTCACTAGTCCTCTAACTCTCCACCTTTTCCTTTCTTTTTCCTTTTCTCTCT

>Ca1:13845069-13845288

CTTCCAATCTTGTACGGTCAATAGGATGGACGCGCCATCTCGACTCGAGAGAGGGTTACACAAGATTTTAGAGCAATTGGGAGCTGGGTGGGAACGCTGTGGATGCGTGATTTGGATGAAATATTGGGAGGATTTTGGAATTTGGGGAGAAAAAAATATGGGGTTTTAGTTGACCCAATTATTCTTAACACCTTATTTGATCTCTTTCTTAAAACCTAT

>Ca1:13843162-13844880

GATAATGTCATTATTTAAATTTTCTTTGAACAAAATATTAAAGAAATTGAGAAAATTTTATAGATTTTATTACAGATAGCCACTTCACATGTGTTATTCTTTGAGCTTTTATCCCTCTTCCTTGATCTCGCCCATGGCCTCTTTCACTTTTTTCTTCTCTTCCTCACTAGTCCTCTAACTCTCCACCTTTTCCTTTCTTTTTCCTTTTCTCTCTCTTTTTTTAGCCATATAAAAGAACAAATTTCAATTTAACGAGAATGCCCAAGTTGATGAGCATGTCATAAAATCATCCCAAGTCCCAAGTTGATGCTCATGTCGTAAAATCATCTCATAATATAAAGTATTTAATGAGAATGCCACTATGTACAATTTCATAAACAATGAGTATTGCACATAATATATACAGACAAACGTAAATGCTAACCATTAAACTAGGTAAAAATCTAAATATATGATTTTATATACGTTTGATCTAATCAAAATAATAGCTACCTATACCTTCACTTATTATTTCAATGATATTTGATAGCTACGTATACCTTCACTTGTTATTTCAATGATATTTGATTGTAGACATCATCAACATAGTACCAGTAGATGGCAAACTAGGATTGGAAAAGTTGCATACAAACAACTGTAAACAAATAACTGTATATATATGCATAAACAAATATCTGTATATATACATACTAAATTAACATAGACAAATAACATACTAAATTAAAATTAAGTTATGCTAATTGATAAAAAGGAATTATCTAATCCAATATTCTATAACTATATATATGCATGTTCACTAAACTGCATATCCACACATGCACATAGTTCTTTTACCTGTAATATATGCTATTATTGTTAATCTCATACAGCGGGAGAGCCGCTATTGTAAAAACTAAAAATCAGCTTACAGAATAAAAATCAATCCCAATGGGATACATAAATACTCAAAAATAGTAAAATAAACAAAATTAAACGGGATAGTCATACATAAAAATTATAATTTTTATTTATTTATCACCAACTTTTAAACATCATATTTAATAATAACAATAATTACTATTATAATTCTAAAATTAATCAAAACAGAGAAAGAGGTAAAACTGAAAACAGAGGTTGGTATTTTAAATGATGAGGCCATTGTTGAGTGCAAGCAGAGGTGAACTTTAGGAGCAGAATAGAGAGGAAGCAATACACCGGAGTAGAAAGGACAAGAATGTGAACACAGAAGGAACGGAGAAACAAAATGGAGACTAGCAATGCAAAACCTCGAAGGTGGTTCGGTGGCCCAAAGCGAGGCACCGGACCACTGCACTCTGACACAATTCCGCTATAGCATGCCATCACAGCGCTATTGACAACATAGATCTTAAAATGTACCTTCATATTATTGGGGTCATTACACTTTTTTTGATATGTTCTAAATATTTACGTCATACATAAGACTTAACTGTTAAAACAGGTACATCTTAATCTAAACCATAATTTAAAAATATGCATACACAAAAACCCAAAGTAGTACATATAGTAGAAATTCTACCTAAACAATCTCAAGGAGCTTACAAAATCACAAACATATAAACATGCCAATGAACAAATACATACATATATACACCCCGAAAACTCACAAGGCAACAATAACATGGAAGAAATAATGAAGTAATTAGGAAAGAAATAAGGAATACATACATACCGTTTGAAATATCCTTACTACCACTTCC

>Ca1:14300839-14301136

CACATGAAAGAAAACCAGGCATTGGACACAACATCACAGACTAGCAAACCAAATTTTGTGTGTCTCTGTGCTGTAACAAACAGCTACTCTCTTACTCATGCATTGATTCCATAATCTCTCTCTCTCTCTCAGAGACGCCATGTATCGCCACATCTACACCGCCCTTCTTCTTTTCATTCTCTCCATTTCCCTCTCTTCATCATCATCATCATCATCATCATCACCAACGAGTCAAATCAACTCCAACTCAGTTCTACTAGCTCTTCTCGACTCACATTACACTGAACTCGCCGAACT

>Ca1:14726022-14726539

GTGGTTCCTTACATAGTGATGCATGTTTCGTTTTATTTCGTCGGCAATGGTACTGTACCAAGTAGCAACACTATTTTAATATTGTCAACCAACGGTTCACATTCCCCACTTTTACCTTAAAAGATAACACCATTGCGAAGTAACCGAGTAGACCAAACAATTGCCAACCAAATAATGTGTCTCACTTTCCACATATTGTCTTTAAACATCCCTTAAATTGAAGAAAATGGTTATCTCTATGGTAACAATAAGCCCCCATCAATCACAAACCACATAACACAAGAAATATTATGGGACACATCATTAATGCGCCTCACCGCAAGAGCCTCCAAGGATGGTAACAATTTTGTAAGAATCTCCACACAAAGATTAACACCTTCGATGGCACTTAATATTTCCACAAAAACTGAAGTGAATTCAATAACCGAACATCATAAGTCCAAATCCAAATATCCCACCTCCCTAGATTTTGGACAAATTGGTCAAAGAAAAGTTTCCAATTCACTGAACACTCCTC

>Ca1:14801829-14802074

CGTTACGTTGAATTGTTATCGAATCTCCGAAATTCGAAGAGGTAAATAGAAACGAAATAGAAGACGTTGATTATTGGAATTTTTATTGCGAAGAAAATAATATGGAAGAGAGTGAATTGAAGAAAAGACTTATAAAGAAAAGAATGTGCAGATACACGAGAGAAATTGAGATACGTTTAGTTTTTGCGTAAGATTTGATTTGATATTGTGCGAAACGGAGCGTATGCGCGTCTAATGGTTTTGCG

>Ca1:16326878-16327498

ATCAATACCAAAGCAACCAAGTCTGGCGCGCAAATTACAACATATCAATATTACATTTGAAACGATCAGAGTATCTGGTTGCATCACCATGTGACAACTTTCAAGTTTAATTCTTGGAAGTTAGAAAACCCTTTTATCTATATGCCAACTTGCAATCTTCCATCTGAACCAAGCCACACTTGACAATAACTTACGGCTATTTGGATATATTCTCCTAAATTTTGGCATTTGTACTCTTCCATGGTCTCCAAAGAATAGTAGACATTTCCTATCATTGACTAGGAAAGAAGAGACTTAAAACCATGCCTTATAACAACTTCCATATGCTAAGCCAAAAACATTTATGATGTTTTGAAGATATCGAACCACTTGATTCTAATGACAAAACAATCTGATCTTCTGTGTTCTTGAACATGTGTATTTCTGTGGATTTAGTATAAAACAAGTTGTGAATCGACTTCTGTAAACTTGACACTTGAGAAGCATAATGCATCAATATTAATGATCATCCTCTTGTAGATTTTGATCACCATCCTCGTTAAGCATCTCAACATTATCCTCATGGACCAAACAAATATTACATCAAGTATTTTGAAATAAGTAGAAACCTTTCATCTATA

>Ca1:16330260-16330555

AGCCTAATTGAGTCATTGTCACATTCATGATTTGCATTCATTGAGAAGTCACGCACTTAATAAAGAAAGGGAAACAACAATTTTATTTTTGGTTTCTCATAGGTTCGCTCTACTAGAGGTAATTCTGTTTGAGGCATTGTCATACATCAAATGTGGGTGTCTCACCCATCCAAAATTCAAATTATGGTTCGCCATGTCGTAGAAGTCTAACCTCTTCAGCTAGACTCAACCTCCGTTAATAAGGGAAACAACACTTTAATCTACGACATTCACATTGGTTTTTCTGGACTCCAGG

>Ca1:16330693-16330919

GGAAATGTTTGCAACAACAGTATCTACAAGAATCTTATTATTACTTCAGTGGAAGCCCTTGCAATCCTTAAGGAATTCAAAACGCTCGATCCAAAGTCGCTACACTTCAAAACAACACAAATCAGTTAGTGCAAATGAGGATTGCAACAAACACATTTGTAATGTTGATTAAAGCTTTAAAGAGAAAGGATTTTCCCATGAACCATTGGGTTCAGATTGCTCCTCG

>Ca1:17135361-17135764

GTCAGTATATGTAGAAAATCTTTACACTATCAATGCGTTTTAATTAAATCCTATATGTTAACATTTAAATATTACTAAAAATGATTTTGTAGAGTATATATTCTTTGTCTATAAATTAGTACATATTACGTTAACATACGGCTCACTATAAGATATCTCCTTTGTTTTTCTTTGTAAACCAAACTCAGTTGTAATGACCACTTTGTTGAAGATGCTCTTCAAATTATTGATTTTAGTCATTGCTATTATAGGTTCTTTGGCTGGTAACTCTCAAAGCGTACAAACTCAAGATTTACCACCTTGTTGTACAGCTTATGGAGATTGCTTTCCAATTTCTTGTGCTAGATATCCATGTTGTCGTCCATGTTGTTGCCCTCAAATTTGTTGATTTCCGACCCTAAAA

>Ca1:17138769-17139033

ATTGAGAATCGACAATAGATGACAGCGCGACGACGACACTGAACGACGACGGGTTCGAGAGGGTCACACGTACGAGAGGGGGACAGCGACGACACTGAACGTTTGAGAGTGACGGTGACGGTCACAGGTTTGGTGGCAATACTCACAGCATTGAGAGGAATGAGAATCGGTGTTGAGTTCTAAATATAGGGTTCAATGAAGAGAAACAGTGAGAGAATGGTTCAAAACGTAGGAGAAGTGTTCAATCTTATTGGATTTTCTAAG

>Ca1:17135361-17135758

GTCAGTATATGTAGAAAATCTTTACACTATCAATGCGTTTTAATTAAATCCTATATGTTAACATTTAAATATTACTAAAAATGATTTTGTAGAGTATATATTCTTTGTCTATAAATTAGTACATATTACGTTAACATACGGCTCACTATAAGATATCTCCTTTGTTTTTCTTTGTAAACCAAACTCAGTTGTAATGACCACTTTGTTGAAGATGCTCTTCAAATTATTGATTTTAGTCATTGCTATTATAGGTTCTTTGGCTGGTAACTCTCAAAGCGTACAAACTCAAGATTTACCACCTTGTTGTACAGCTTATGGAGATTGCTTTCCAATTTCTTGTGCTAGATATCCATGTTGTCGTCCATGTTGTTGCCCTCAAATTTGTTGATTTCCGACC

>Ca1:17266402-17266823

CATTGATGACACATATTTTCAATTAACAACTCACTATATCATCAAAATACATATAAATTTATTGGTGCAATTTTGGCATATTAAAACAAGAAAATAGCATCTATTATCACCCCGATTTTTTTTTTGAACTAAATCCTCCATCAAGTTTGTTCAATAACAAGAGTTTTTTTTACAATCTTCCTCTTCTAACATCCTCTATGTATATATTCTCCAAGACCTCTGGTTGATGCACAATTTGTGACATGACATGAGAAGCACTTGAAGGATTTTAACATAATGATCATCCATACAAAATTAGGATGTAGCCAGAGGCAAAGCCATATAGACCTTCGAGTTGAGTTAAAGCCAAAATAGTGGAAATGGAGACACCAATTCTTTGTGGGTCCTTGCAAACCAAGATAATATTATGCCACATTCTCAC

>Ca1:17267567-17267900

CTGTTCCTGTAAATAAAGGTTGTGGAGCTAACATTCCCATGAACTTAACATGTTCCTGAGCTACATCCCGTTGAATTAGCATCGGGTTCCTGGTGAATCACAGCAAACCATCAAGCCACTCCATTGAAAATGGCTTTATGCCTTTATGTTACTTAAATCATTTGCACCAAGAAGTGAGACGACCCATACTTTCTCTTTGTCTTTCCTCGCGGGATTATTTTTCCTCATTAAAGTCGAACAAACATTTGTTTACTTTGTAGCCTTCAGGTCATATGAAAGTATAATCATTGGCCTAAACCAGTGGAAGACTATCTCTTTCATTGAACCCAACAC

>Ca1:17272854-17273065

CTGAAGGGTGTTTCTGTCTCTGTCACAGTTGAAATTGAAATTGAAATTGAAATTGAAATTGAAATTGAAATGAAATGAGAGAGATAAATCATGAATGAATTGTTCTGCTTCAGTTTTCAAAGGATAAGGAGTAAAGAAGATCAACGGATAGAGTGATGATTGGGGAGATTAGATCGACGGTAGAGAATGCTGTTGCATAGACATCATCGAC

>Ca1:17272854-17273155

CTGAAGGGTGTTTCTGTCTCTGTCACAGTTGAAATTGAAATTGAAATTGAAATTGAAATTGAAATTGAAATGAAATGAGAGAGATAAATCATGAATGAATTGTTCTGCTTCAGTTTTCAAAGGATAAGGAGTAAAGAAGATCAACGGATAGAGTGATGATTGGGGAGATTAGATCGACGGTAGAGAATGCTGTTGCATAGACATCATCGACACAAATCGCGTAATTTGATGTTCAGGTGGACGACCCTTGTTTGGTTAACGAAATACAAAGTAAAAAGAAAGGGATTGATTTGGATATTTT

>Ca1:17267272-17267900

GTACTACTATACTATGTCAAACTCAAGTACTACGCTATTGTATAATACATAGACCATATATCAAGTGAGAAGACTAATTAACAGCCATTAGATTGAATGGAGGGCTGAGATTAAAATATATTTTTACGTGTCGCATGACACAAAAAACTATCTGGTTCTCAGAGCCCATACTTTGCTGCTACCTCACACCTAAACGTTCACAAATTAAACAGACAAGAATATGTTCAACAACCAAAAAAGACAAGAATATAACTAGTTTTTCTATTGTATCTCTCACATGTCATTGTTTCACTACCTGTTCCTGTAAATAAAGGTTGTGGAGCTAACATTCCCATGAACTTAACATGTTCCTGAGCTACATCCCGTTGAATTAGCATCGGGTTCCTGGTGAATCACAGCAAACCATCAAGCCACTCCATTGAAAATGGCTTTATGCCTTTATGTTACTTAAATCATTTGCACCAAGAAGTGAGACGACCCATACTTTCTCTTTGTCTTTCCTCGCGGGATTATTTTTCCTCATTAAAGTCGAACAAACATTTGTTTACTTTGTAGCCTTCAGGTCATATGAAAGTATAATCATTGGCCTAAACCAGTGGAAGACTATCTCTTTCATTGAACCCAACAC

>Ca1:17402655-17403058

GTGGAAACTATATCATTACATAAAAGCTCTGACAACAAATGTTTAATCACGTCAAAGAAGGCTTTATCATTTAAGTTACACACTTCATCTTGCATCAGAAGCATCCATCAAACAGCTTAATTAAGCATGAAAGCTTCAAAGATGACCCATTCAAGGTATTAAACAAATTTGAAAGGGTCATAAGTTCAATTTACACACTAAGGAATTTACAGAATCTACATGCTATACAATAAATAAATAAAACAACAAAATATCACTAGTTTGTTACATTACACAAATCTAAAGTTGAATTCATCCCAAAAACATAAACCTTTCATGAGAACATTGTTTTGAAAAGCTATGATGTCTTTGTTGTTAACATGTCCACTTCATAATTGATGATTTCTTCTAATTCAAGTTCAAG

>Ca1:17811779-17812218

CACAGTAATTTTAAGGATACAACTTTCCAATAGTACAATATTTTTTATTAACAGATTTCAAATTCAAAATTTTAATTAAATTGATAATTTGATAAACTGTAAATCACGTGAAAAATTATAATTTTTTTCATACGTAATTAATCAACCATCAAGAGTATAAAAAAAAATAGGTGTCCACGTAGGAAAATATAAATGACCTCAATATTTTGATAGCGTTGTCATATTTTGTAGGCACAATATGATTATATCAAACCCCAACCGCCATGGATCTCGGCAAAGAACCTTTGGAGAGGTCCCCTACTACGTAGCTTTGTAGTAGTAAGATTCTTATCATAATAATGAATTACACACTTCGAAGGTTTTCTCCCTTAATTTCTTTGTTTCATCTTAACAATAGATTTGTCGCAAGTTGCATATAAGCGTGTGTTCAGCCACATAC

>Ca1:17814246-17814447

TTTGGGAGATCGCAACAATAGAGGAGGAGAGGAGTGTGACAGTGAGGAGAGCGGTAGTGGAGAGGGGGAGGGAGATGAGAGCGACAATTGAGAGGGAAAAAATAACAACACGCTGAAGAGGGAGAGGTAGAGGAATACATACAAGTGAAGGTGGAGAGGGAGAAATAAATTAATGTGTCTGGGGAAGAGAAATGAAATGAG

>Ca1:18007210-18007569

AGGTTCATCTCCTCTATAAACACCATTCTCTTAAGATACCAAAAAATCTCCTATTTATCTTTGTTCCAAATAGATAAAGAACCCACCATGGCCTAAAGACATGAATATAGAGTACCTTTGATTCAATAGATTCTTATGTTTGAGCTAGTGACACCCTCTCACCCTCACATTTAACTCACAATCACAAAGTGCTTCTCTATTGTCCCTTTCACACTTTTTTATTCATTTCCAAGTCAGTCTTCCACGTGCTTCACTCAATAACACCCTTTCCCCTTTCACACTAATTCTCATTCTCACATTCTAACTTTCTTCAACACCCTTTTCTTCTTCCATTCCTCTCTCTCTTTCATCAAAATCAA

>Ca1:18050223-18050753

CTTACCAGACAGCAAGAACGATACACATAATTATGTCAAAAATCAAACACAATCTGATTATTGCATGGCTTCCTCGGCAGACAAAATATTCATTCATTTGAATGTAGCAATAGCTTGCTTTCACATTTATTTTTTTTACAATAGTGTAATATGCATACTTCAACTGTGTTTAAGTTGTTTGTTACCCTCAAGAGTATTATAAGCCCGGTTCATGAGCTTGCAAGGACCTCACCACTGATACTCATAACTTGTGCAATTATTTGCTGCCATCTTCTCCACTGTTTATAACCCTCAACAAGATGAAACACATTCTTTATAACACTCAACAATATGAAACATATTAATCAAAAAAACATCTCAGAGTAAGGTCAGCATGGCACTGGCAGCTAACAAGTTAACATTCTGCTTGTAACAAGGGCCGCTCCTACAGATACCTTATACATCCTGAAGAATTAAGATGAGCATAAAAGATGAGCAACTGATACAAGAATTCCTCAAGCCAAGCAAACAATATCCATCAGTCACTTTTG

>Ca1:18050223-18050667

CTTACCAGACAGCAAGAACGATACACATAATTATGTCAAAAATCAAACACAATCTGATTATTGCATGGCTTCCTCGGCAGACAAAATATTCATTCATTTGAATGTAGCAATAGCTTGCTTTCACATTTATTTTTTTTACAATAGTGTAATATGCATACTTCAACTGTGTTTAAGTTGTTTGTTACCCTCAAGAGTATTATAAGCCCGGTTCATGAGCTTGCAAGGACCTCACCACTGATACTCATAACTTGTGCAATTATTTGCTGCCATCTTCTCCACTGTTTATAACCCTCAACAAGATGAAACACATTCTTTATAACACTCAACAATATGAAACATATTAATCAAAAAAACATCTCAGAGTAAGGTCAGCATGGCACTGGCAGCTAACAAGTTAACATTCTGCTTGTAACAAGGGCCGCTCCTACAGATACCTTATACATC

>Ca1:18432412-18433149

ATGCTAAGCATCATCATTGTCATCAGGCAAAAAGAAAAATAAGCATCATCATTGTCATCAGGCAAAAAGAAAAATAAGCGTCATCATTGTCAAGATTTTTTAAATACAAGGTAAAATTTAAACAAAGTTTTTAAGAAGAGGTGGACGATGAGGGTTCTGTTTGTTCAGCCCCATCAGAAGTAAAACCTTCAATCCTTGATGTCTCCTAGCTAGCTCAATAGGAGGCACTTGAGTGCTTTCTCTATATGCTTCTTTATGTTGTCAAATAGTGGTTATCACGATGTTATAACACACTACAGTGTAGTGATATTTTAACCAACCGTTAACATTCTGGGATACACGATATAATTTAAAATACTGTCAAATAGCGGTTATAGTACTGCCAGTGTAACAACATTTGAACAAATTGTTATTTTCTGCAACACTGGGCTTTCTACATCACCAGCAGTATAAACCACAACTGAGAGATAATCAATAGAGATAGACTCATTTGAAGGCATTAAAGAGAAAGCTCTTGCGAAATCACGTTGAATCTCGTTTTGATGTTTGTATCAAATTTTGATCGGCTATATCTTGATTCTTGAGTGAACCTTAGTGGCTTAAGAAGGCAACCAAATTGTATACTAGAGAAACTACACTAAACCAAATGAAAATTATTCGGAAGAGTAACTTACAAATACAAATCAGAAGCATAAGCCTTCCACTGGAAAATGTGCAAGTCTGAAATGAGTTTATAC

>Ca1:18444574-18444955

CACACAAATGTAGTAACTATCCTAGACCATTAAGCATTGTTCAATTCCTGATTTCTAAAAACAAACAAATTTCTAATATTTTCATAAGTGTGCAACTTATGATTGAAGTTTGTTATTTCTAATCGAATACAACTTCTGTAACTTATTATTCAAATTGTTATTTGTAATAGAATACAACTTCTGCTATCCATAGTTTTTCAAAAAATAAATCACTATTTATGCAACCTTAAAATCAACTTTTTAATGCTTAAACAATTGAGTTCAATGTTAAGCATTCAAAAAAATGAAACACAACCTAATTTTAGCAATAGCACTATCTTTCCAACTTTGTTCTTGGTGTGAGCTTAATGGAAGAAAGAAACATCTACATCTACACTGTTC

>Ca1:18457208-18457524

TCAAAACTGATTTCCATTCAATTTTATTTCAAGAAATTCTGTCCCATAACTCTGCTACAAAATATGCAAAGAGATTTCATACAAACATGTTCTGTTAGAAGAATCAACTCAAACAAATTGAAGACCATATAAATTCTTCCTAGCTTCATAATCAAATTGAGGGCCATGTACTTCTTCCTAAATGGATGAGACTAATTCTTTCTGCCGCATATTGCAGCTCTGCTGCTGCTGGTACCTGTATACGAAACAGGATTTTCTTGAAATTTATTTCAAGGTCTTCGTCGTCAAGGTGTGCACAATTCTTGAGATGTTATCT

>Ca1:18457208-18457530

TCAAAACTGATTTCCATTCAATTTTATTTCAAGAAATTCTGTCCCATAACTCTGCTACAAAATATGCAAAGAGATTTCATACAAACATGTTCTGTTAGAAGAATCAACTCAAACAAATTGAAGACCATATAAATTCTTCCTAGCTTCATAATCAAATTGAGGGCCATGTACTTCTTCCTAAATGGATGAGACTAATTCTTTCTGCCGCATATTGCAGCTCTGCTGCTGCTGGTACCTGTATACGAAACAGGATTTTCTTGAAATTTATTTCAAGGTCTTCGTCGTCAAGGTGTGCACAATTCTTGAGATGTTATCTCTGCAA

>Ca1:18460017-18460314

CATGCAGTTCGAATTCTAGCTAATTATTAGGTATTATCAAGACCAATGTACATCACGACTTTGAGAACACTTCAATCAAAACAAAACTAACATTTGTCAGAGTAGGAAATGACACAAGTTAGCTAAACTAGCTATAATCACGGTCTGGACCAAAAAGTATTCAAATCAAATAAAAACTGAGATAACAAATTAAGGCTAAAACACCTGAAAATTTGTTCATGACATGCTGGTTTTGTCCCTGGCATGTGCAAGATTGAGTTTTTCTTCATAAAGGTTCAACAGATAAGAAGATTCCTC

>Ca1:18468125-18468653

GCTACTATAGAACTTATCATTCAACTTCAAATGAAAGTAGATGATGATGCTCATACTCTTTCAGCAGAATATCATCACTCTTCTGGCAATGGGATCCATTTCCATTTTTTTTTAAAAGGAAAATATGTTATTCAAAAGAAAAGAGAAAATCGAAGAATGAAATAAAACAATGAACCTTGGTGGCCCAAGAAGACAATTTTTTTTTATATCATTGTACATAAGTAATAGAAAACTACACTAAGGCTCGTTTGGTTGCATGACGTGTTTTTTCACGTTTAGCCAAAATAGCGGCAAACTTTCCGCGTCAAGGTAGAAGCTTGGCTTTCTAGCTTCTGTGAAATTGCAGGGAAAGCTTGGTAGAGCACACAATTGGTGCCTCTGGCGCCAAACCAAACATACTATAAATATTCCGTAGAAATAAGGATGTGTAGAGAACCAAGGGAAACCTATTCAGGAATCCAGTGGAATTACTTATCAATATAAAGCCTAAGCCTTCATCTGGATTATGAAAATTGTGTGAACCTGCAA

>Ca1:18468125-18468647

GCTACTATAGAACTTATCATTCAACTTCAAATGAAAGTAGATGATGATGCTCATACTCTTTCAGCAGAATATCATCACTCTTCTGGCAATGGGATCCATTTCCATTTTTTTTTAAAAGGAAAATATGTTATTCAAAAGAAAAGAGAAAATCGAAGAATGAAATAAAACAATGAACCTTGGTGGCCCAAGAAGACAATTTTTTTTTATATCATTGTACATAAGTAATAGAAAACTACACTAAGGCTCGTTTGGTTGCATGACGTGTTTTTTCACGTTTAGCCAAAATAGCGGCAAACTTTCCGCGTCAAGGTAGAAGCTTGGCTTTCTAGCTTCTGTGAAATTGCAGGGAAAGCTTGGTAGAGCACACAATTGGTGCCTCTGGCGCCAAACCAAACATACTATAAATATTCCGTAGAAATAAGGATGTGTAGAGAACCAAGGGAAACCTATTCAGGAATCCAGTGGAATTACTTATCAATATAAAGCCTAAGCCTTCATCTGGATTATGAAAATTGTGTGAAC

>Ca1:18551703-18551989

ATTTAAAAAAGGAAATAAAATAATTACAATAAATAATTTCATTATGTATTATAAGTTTACAATTTCAAATCTATTTAGAATGTTAAAACCAAATCAAATTACAAACTAAAATAAAGGCAAAATTAAATTACAATAAGATTTAAACCAAACTAAATTAAATTACAATAATTTTTAATTTGTTGCTGTTGTTGCCTCTCTGCCTCTCTGTTTCTGATGCAGTGTTTGGCTCTATTTTGTTGCTGCCTTTCTGTTTCAGATGTAGTTGTCATTGACCTTTGCTTTGTAA

>Ca1:18552532-18552832

CCCTTGGACGATGTTGTTGTCACCATCCTCACCGTCGCCGAGGTTGCCGTCACCGTCCTCACCGTCGTTGTTCCCTCACATCGTTTCTGTTTTCGCATCCTGGTTCCGATCGTGTGTTTGGCCGCCTCCTTTGCAACTTCGTGTTTGGCCGCCTCCTCTGCAACTTCATGTTGGCCGCCTCCATTGCAACTTCATGTTGGCCGCCTCCTTTGTGTCTCAAGTTGGCCGTTTTTCCCCAAATGAAATTAGGGTTTCTTAGTTTCAGTTTTGACCCTTTTTGATTATTAAGTTGGGTTGGGC

>Ca1:18556079-18556296

CTGTTTGGGAGAAACTAGAAATCGACATTTCTCGACGGCAACGCTTCTCGACAGATGAAAGGGGGAGACAAAGAAAATTGTTGCCCGTACGGAAGCAGAAATTGTAGACTCGTCGGTGCTGAAGATTTCGCCCATGAAGGAGGGAGTGAAAGCAAAACTTAGGGCATACGAAGTTGAACGTGAACGTGAATGTGTTTCATGATGTTGGTTCACGAAG

>Ca1:18554771-18555491

GTCAATTAACAAACAATCAATAATGCATTATGTTCTAATAGTATTAAGAACAACAATAAACAAACCAAGTAGAAATTGAAAAGTTACTTCTAAATTCAATAAGATTCTGCACAAAATTATCATTGGAGCACTGCAAGTTTGTTAGGTTTATCTTACACAAACCCATTGATAATATTAGGTTAATGATTGTTGCATACAGTGAGGAGTGTGTATAACCATAGCTATTCAGAGAGCATTGCAGTGAACTCAGTTTGTTACTTACCATGTGGCTAACAAAGTAATCTGAGAGAGTGTGTAAGGAATTTTTACGGTTATAACTCATAACTAACAAGGACGGTTTCTGCTTGTACAGGAGTAGTGAACACTGATTAAAAAATGTTTACAAGTTCCATTATGTGCCCTGCTATCTGCATTTGATGCTATGAGCCTCTTACAGTTGTCATAGTAGGGTCCAGGGAAGGATTTGACCCATTTTGCCATAGTAATAAGACCAGTACAAGACATCACCAAACACCAGTGCAAGTTAAAATAACAGATGAAAAGACATCACCAAACAAGTAATAATAACACTAAACACTGACAACACCAAACAAGTAATACGATGAAGAGACAACACCAACACTAAACACTCTTCAGCCTTTTAATAACACCAGTACAAGACAACACCAAATACCAACAAATAAGTAATAAGAACACTAAACAGAGAAGTAGAGAATAACA

>Ca1:18557157-18557780

CTTCTATTTCATGCGGTACCATTCTAATTATTAGGTACTTGGTTAGGAGGTTTGATATTATGTCACCACACAAGTGGGCGAACAATTCAAATTGACTATGTTCTCATAGATTCTCTCAACTCAACCTGAGATACATATACCTGACAATCAAGGTAAGCATGTGTTGCATGGTAGCTCCCAAAATTTGGAAGGCTACCCCCAAGTCACATAAGAATCCCCACCAAAATACTCTCAAGGTCAAGTTATCTTGCCTTAAGGCTCAACAATAGACCTCCTTGATCAGACTCATTAAATTGTACTTTCCAAAATCACTGGACTTGTCAGATCCTCTCTATATAATAACGATACACTCAACTAGGTCATATACACTAACACAGTTCCCTCCTCTCGCCAGCCTAGGCAACTCCATCATGAGTACCATGAGTTGGATTCATCACAATTTCACTCAATCAAATTTGTTTATTAGATAACTCGAATGCAATGTACACATATTGTTCATAGTCATTTGATAATAACTAGTCAATAATTCACAATATACATTCCAAAATGTTCATTAAGATACATATATGTCACAATCAATTTTGAGCATACTTTAACATCTCATATTTACATCATCATCATGG

>Ca1:19785240-19785616

AGGTGGAGTTATCATTGACACAGGGAGTACACTAAATAATATAGTAGGGAGTTATCATTGACACATTTTTCTTAAATAAAGAATTACAATGCAAATGCATCACGTGTTTCTCAAGATAATACGACTACCCTTTTCTAAGAATCAACTTCTTCTCTCAGCCTTCCCTAAAATCCCTTAACAACATAAAGACAGTTACACAGAAATATGCAACAACCATCAAAATTTACAAACAATATTTTTACCAAGTTACACAATGGAGTACAAAAGTTTGCAAACATTTTTATAACATAAGACACTGTAACAAAACAGCAGCATGGTCAGTAGATGGCTCTTTGGCAAATTCTTGATGAATATGGTACATCCAAACAAATGGTAC

>Ca1:19786384-19786748

CTTTTCTCTGCCACAGGCAAAGTGGCAAACCATCACAAAGCTCTCCCTGTTCGCTTCATCAGTCGTCAAGTATCACTGCAAACAACACAGTCCAAAAGAGGAGAAGACTTTGCATCTGTAAAATGCTTAAATGTAAACAATCTCCTCCCAAATAATGTGTAGATTCCTTTGCTGTAAACGGAAGGGCCAAAATGTTCCATAAATAAAAACTAATTACCAATATACAGTGAAAGATTGAGTGAGATTGTTGAAACATTTTGGAAAATGTCCAAAACAACAGTAATTGTGAATGTGAAACTGTTCAACCAGGGATATCTCCATTCACCAATATTGCAATCCTCCACGAAATATTAGTGCTCGGAAT

>Ca1:19932388-19932740

CTTTTCTCAGGTTCATTATTTTAAGACGATATATACAAAGTGGTGATAATAAAATTATATTTCACACTTTATGGTCTGGTAATGGTGATGTGTGTTCCAATTTGTCTTTATTGCAATGACGAAGAATACTATGATAGCACGCAGGGAAATTAAGATAGAGTTAAATAATTGAACTCCGAGTCCCCTTAAGTATCTCAGTCCACTTACTCTTCACCTATAACTACTATTAGCCATTCATCTTAAATCTAAAATACTTTTGTAACCTTTAGCATTGATGTGCTCTAATCAAAACCATTTAATGTCATCATGACTATACAATGAAAAGAGTGATTTTGAGAATGAAACAATAGCT

>Ca1:19938491-19938739

TAGCCTATATATTGGAAGAAAAAAATCAAAACGAGAAAGGAAAAACGATCACAAGATATAAATTGGGAGAGTTTCATTTCTTTGCTTTCGGTTTCTAAAAAAAACAATAGAAAGAGAGAATGTTACCGCAAAGAAGAAAGGAAGATCTACCATCAGTTTGCTTCCCATCAACCCTAGTCTTCAAGCCAATTCTAGCAACAAGAGAGGAAAAGGGTATTTTTCTCATTCTTTGTTGTTTTGTTCAAAAT

>Ca1:22130643-22130887

TTCTCATCATAAAATAAAAGTTTCTCATCATATATATTATAAATTAACAAAAACTCTTACAAGCATATCAAAATAGCTAAATCATATTAACTTTAGAGTATTTACTAATAATACAAGAAGTGTCTTACTGACAAAATAATAGGCTTACAAAGTTTTATATAACAATCTCAACAACAACAAAATTAAATATCCATTTAATTGCCAAAACTCTCTCTTCAACGGCAAATAAATTTTAAATCTTCAC

>Ca1:22131630-22132845

CTCAACCAATGGATGGTAGTTCAGCACCTCAATTGCAACTAGAATAACTCTTATTTCAACATAACATCGTACAACATAATAACCATGCACAATTATAAAGAAAAATCATTAGTCGTAATATAACTTATCTAAGTGGTATCATGATGATTATGAGAAATTGTTAGCCATAAATTATTATCATGTCTTCAATCCAACACAACTTAAGCCAAATAGAAATCATCAGCCATAATGTAACTTATCTAAGTGGTATCATGATGATTATGAGAAATTGTTAGCCATAAATTATTATCATGTCTTCAATCCAACACAACTTAAGCCAAATAGAGAGATAACTATTAAGTGTTGTGGGTGGAAAATAAATGTAGAGGAAATTGTCTTCAAAAGTTGCTTCTTTACGTAAATAGTTTAGTACAAATGCATTTCGTGAACAAAGGGTTCTAGTAAGTAGTAGATTAAATAAAAGGAAAGAAGAGAAAAACCAGATAAACTTCCCTACCATGTGTACACACAATTTGCAAAATAATAACCGGCAACAACAAATACTATTTACAGCAGTATACTATCCTAATTTGCATTGTAACTAAACAAAAAAGATTTACTGGTCTCCATACTCCAAATTTGCAGTAAACAATTAACCGAGACTTTTTCTTACAATCAAACAGGTCAGATCTAAAAACATAAAGGATACATCTCTTCCACAAATGCAAGCTATAGGATTCTCAAACAATATTTAAGACATCCATGAGCAACAGAATGGAGAAAAATGTGCCAAGCACTAATGCTCCATAATTATTTTATTAATATGCGGCTCCAACTCGTTTAGGCCCTTTTCTCATCAAATGCAACATTACTTACTACATCAGAACAAGTTAATTCTATTTGGCAACTTAAAACTAACAAAAACAATTTTCAACAGATTAACATTTAGTTCATTAGTTCCTTATCATCTAGAAATCCAATTCTAGCATTATAATACTATGTTTTAGCTTCGAGAAGTTTTTTTTTGTTTTCATAGGGAAAAATACATCTTCACTAATTTCTAGCACAATCACAAACCAAAGAAAAAAATCCAAGCACAACTAACAACATCACCAATTGAGATACAAGTTGAAAGATAGGTATTCACAGTAAACAATTCAGTAACGATAGATTTAAATGAAATGATAAACAAAAGAACTCGAAACCTTTTAGTTGAGTTTCCCTTTAGCTCAAAAC

>Ca1:22133005-22133261

CGATAGAGAAATGATGGGGTGGTGTGAAATAGATTGTGAGAGAGGAGGCGATGTCGTCACAACGGTTGCTGTAGCCAAGGAATAGCGCTGCCATCGTGCAAGTGGTTGTTGCCGTCGTCGTGGTAGTTGTTGTTTCTATTCGTGGCGTTGATGTTGCAGAACTAAGAGGATTTAGGCATTCCAATGATAAAATAAGTAATTTAAGTATTTTGGCTCTAACCTAAAATTTGGGGTTTTGTAGGCGGCAATCCAGAAT

>Ca1:22133005-22133280

CGATAGAGAAATGATGGGGTGGTGTGAAATAGATTGTGAGAGAGGAGGCGATGTCGTCACAACGGTTGCTGTAGCCAAGGAATAGCGCTGCCATCGTGCAAGTGGTTGTTGCCGTCGTCGTGGTAGTTGTTGTTTCTATTCGTGGCGTTGATGTTGCAGAACTAAGAGGATTTAGGCATTCCAATGATAAAATAAGTAATTTAAGTATTTTGGCTCTAACCTAAAATTTGGGGTTTTGTAGGCGGCAATCCAGAATTTTGGGTAGATGAAAAAAA

>Ca1:22133005-22133338

CGATAGAGAAATGATGGGGTGGTGTGAAATAGATTGTGAGAGAGGAGGCGATGTCGTCACAACGGTTGCTGTAGCCAAGGAATAGCGCTGCCATCGTGCAAGTGGTTGTTGCCGTCGTCGTGGTAGTTGTTGTTTCTATTCGTGGCGTTGATGTTGCAGAACTAAGAGGATTTAGGCATTCCAATGATAAAATAAGTAATTTAAGTATTTTGGCTCTAACCTAAAATTTGGGGTTTTGTAGGCGGCAATCCAGAATTTTGGGTAGATGAAAAAAAATTTCACCAAACAAAAACTGGGGGAATTTTTTGTTGGTGCCTCTTTTTTTTGTTGTTG

>Ca1:22131630-22132489

CTCAACCAATGGATGGTAGTTCAGCACCTCAATTGCAACTAGAATAACTCTTATTTCAACATAACATCGTACAACATAATAACCATGCACAATTATAAAGAAAAATCATTAGTCGTAATATAACTTATCTAAGTGGTATCATGATGATTATGAGAAATTGTTAGCCATAAATTATTATCATGTCTTCAATCCAACACAACTTAAGCCAAATAGAAATCATCAGCCATAATGTAACTTATCTAAGTGGTATCATGATGATTATGAGAAATTGTTAGCCATAAATTATTATCATGTCTTCAATCCAACACAACTTAAGCCAAATAGAGAGATAACTATTAAGTGTTGTGGGTGGAAAATAAATGTAGAGGAAATTGTCTTCAAAAGTTGCTTCTTTACGTAAATAGTTTAGTACAAATGCATTTCGTGAACAAAGGGTTCTAGTAAGTAGTAGATTAAATAAAAGGAAAGAAGAGAAAAACCAGATAAACTTCCCTACCATGTGTACACACAATTTGCAAAATAATAACCGGCAACAACAAATACTATTTACAGCAGTATACTATCCTAATTTGCATTGTAACTAAACAAAAAAGATTTACTGGTCTCCATACTCCAAATTTGCAGTAAACAATTAACCGAGACTTTTTCTTACAATCAAACAGGTCAGATCTAAAAACATAAAGGATACATCTCTTCCACAAATGCAAGCTATAGGATTCTCAAACAATATTTAAGACATCCATGAGCAACAGAATGGAGAAAAATGTGCCAAGCACTAATGCTCCATAATTATTTTATTAATATGCGGCTCCAACTCGTTTAGGCCCTTTTCTCATCAAATGCAACATTACTTACTACA

>Ca1:22133005-22133231

CGATAGAGAAATGATGGGGTGGTGTGAAATAGATTGTGAGAGAGGAGGCGATGTCGTCACAACGGTTGCTGTAGCCAAGGAATAGCGCTGCCATCGTGCAAGTGGTTGTTGCCGTCGTCGTGGTAGTTGTTGTTTCTATTCGTGGCGTTGATGTTGCAGAACTAAGAGGATTTAGGCATTCCAATGATAAAATAAGTAATTTAAGTATTTTGGCTCTAACCTAAAA

>Ca1:22140699-22140946

ATGGAATTGAATAAAAAATCCAAAAACTGAAACCACTATCAAAGACATCAAGTTTTGTACTAATAATGGATGAAATCCAATCCACCAAAAGGAAAAAAGAAAGAAAAAAGCCCACTAGCTCAAACACACTACAATGAAGGCTTTCCTTTAGTTGGAAACAATGTGCTACTGCTACTATTCTTCCTTTTTATCTTCTTAGCAGATCATAGTAATGCTCTGACTTTCTGTAATCTGACATGCAGATAGC

>Ca1:22742499-22742835

ACTGTCAAAAATAGTATCTAAGACAGATCTATAAATCAAAGTCGCAATAAATCACAACATATCCCCAACTATATAACCACATTTCTCCAAAATACTGAGCAAAATGGGAGAATGGGCTCTATAAATAAATTCAAACTATAAGATTCAAGGGAGGAAAAATAAATTCAAACTATAAGATTCAATGGAGGAAAAATAAATTCATGGACCAGGAAAATTGAAATCCACTTTTTCATTGCGCAACATAGGACCCAAAACAACCGTCTCCGAAGAAACAAATTACTGCTGCTTGCACTCTGGTGGTCTTTCGCCCTGAGCATCGCTGAGCTGTGCACCGCT

>Ca1:23307443-23308558

GATAATATTACATAAAATCATGATAAAACTTCAAGTTAGCTGCCTACTAACTAATGCAGTCCTCCTCTTATTTGACCTCAGGATCAGATAGGTGTATTTGGATTAAAAAAATTGAAATAGGAAAAGTTAACCAGAGCTGAATAGGTGTGTTTGAACAAAATTAAAGATGTCTGCAGGGTCTAGGAATTATGGGTGTTCAAAAAAACCAAAAACTGAACTAAACTGCCGAACCGAACCAAATTGGTTTTGAACTGAACTGGTTTAAGAACCAAACTGTTTTCGAACTGATTTTGTAAAACTAAAACTAAACCGAACCGGTTTTTCAATTCAAAAAACAAACCAAACCGGTTATTTGTTAAAAAAACTTGAACCGAATTTTTTTTAGAAGTAATTAAAGGTAATTATGTAAATTTTGACCTATATGAACAAAAAAATTAAGTTAAACCAATTCAGTTCGATTCAAAATAATAAACCAGTTCACCACTTTTATAAACCGGTTTGATTCGGTTCTTTGAACTGAAAACAGGTTCAGTTTGATTTTCCAATCTAAAAACCAATTCGGTTCAATTTTCAAACGGTTTTAGTTCAGAACTGAACTTTGCCCACCCCTACTAAGAATAGCCTCTTTAAGGCACAATATACAACTTCCCTCGAAGAGGAATATGCAAATTCTCAACTAGAGGTAGACTTTACCCAATCGGCCATACAGTATACTTATTTCATCTTCTCTTGTATGGGAAACTCCTCTCTATATATCTATCTCTCTCTTGTACTTTATATATGCCCAGAAAGAATTAACAAATTCATATGGTTTTCCTTCAGGTCCATGTCGAAAACTATTGTTACAACCTCGGAGACCATGGGAGCCAAAAAAGATGAATAGAAAACCTTATTTCAACAGAAGAATTTAAACCCAAAGGCAACCATTTAGCCAATGATAAATTGAACTTCATGCGTGAAATTTTGTTCAACATGACACACCATATCTCGTTAAATATTGGTGATATCCGGCGAGCTTTAAACTTGTAAACAATCGATCTGTACTCATTGACTGAATCGTGAAACTCGGACCTCTCCAATTACTGCAAAAATATAAGTGTGCTTTGTCTCTGAAC

>Ca1:23690274-23691831

TAGAGTCCAAGCACAATAATACATCTTTTTGGCTATATAAGCCTTACATAATCCTTAACAAGGTATGTCTGTTTAATCTTAATCTCCACCCAAACATGTATTAATTTTGAGCCTTAGAGTGTTTGAAGGTAGTTTGTAGGTCATATTCACCTCATATTACGACCACACGTCACTCTCTAAGATAATTTCTTTCGCCATGACCACCTTGAACTTGAGTCCCGATCAGAACAAAATTTATAGAGAGTACTAAAATTTATTTATTATACTAAAATTTATAAATATTTTAAATGAAATTTTATTATTAAAAAAAGGACTAATTTTAAGATAAAATTGATTATCTAACATATATACTCTGGTATAATAATATAAGTGGCATATTAATAAAAAGAATTAAGATAAGTGTCAATTTATCTTTTTTTTCAAATATAACATTAATTGTGTAATTGTGTTATCTAATTAATACTGTGTGCACAACTATTAAACTAGTCTTGTACTTTACATATTTTTAAAATGCTAAATATATCAATGATTAGTAATATTAATTTAATAAAATTATTTTTACTCTTGTTTTTATGCAAAACTAAGACTAAGCCATTTCATTAGCAATCATAAAATCAATCCAACTGACGCGTTGATTACTAAAGGTTGTGTCCTCTCGATTGCATATGCCATTTGAGTGCTATAGATGTGTTGATTCATGGCTTCCTATGAAACATTGGATGAAATTCGACTCTTGTTTGGAGGGACTTTTGAATCTTTCATAGTTTTATGGATCTTTCTTCCTCCTCTTTATACTTCATGTGTTGCTGTAAGTGGTCTTTGTGTGGAATTCTTTGTATTTGTGACTCGTTTTCCTCATGGTTTTCAGCTTCTTCGTATTTTCATTTGTGCAACTTGGAGTGTTATTTTTTCCAACTTGAGATGTTTAGCTATTTCTTTGCTTTGTTTCCACAATAGACATGTGAAAATGTGTGTGTGTATTTTCGCTTGAGTGAGAACCATTTATGGTTTCATGTTCTTGTACGGTGGCACCTAAGATGCTCTTTCACCCTTTCACCCTTTCGTGTTCTTACACTTTTTCTTTCTATTGCATGGTTACAAGATATATAAGATGTTGACTTTGCAGAAGATGATTGAGAGGAAGCAAAATAGGAATTCCTAGAGTCAAGGCACTTCTATGTTGGAGCCTCAAGGCAGCCACGTCCATGCAATTCTTTTGAAATAGATGTCGATGGTTGAGCACAAAAACACACGAGACTAAAATCATGTCGACCTAAAGCATCTTCAACTTCCTACATCAGGCACTACGCGTCAAAGTAGCAATTTTCTCTTTTTGTTTCTTAGATGACTGTGTTGCGTGGCTGACATGCACCCCCCCTAAGCCGTTAGAATGTATCTTCTATAAAAACTCATCGATGGCTCTCAAATAAGGGCTTTTTTATAATTTCAAATCAAGCTCCTTAAATATATCGTTGGTAAGATTTGATCTTTTCACTATTTGTTCCTCAAAATATTGGATTGGTATCATAACTCATATGTACTTGTTTAAAACATGTG

>Ca1:24051710-24051986

TTCATAATTCAGACCTTCGCATCTTCAAACAAAATAACATAAATTACTTGTATATATACACATAAAAAGGATATTTTTGTAAGCTGCTCAGGAATAGTCTCACCACAGTCGAAGGTATAATCCCCATCTTCGAGCTACCACCGGAGGACTTTCTTCCTAGTCTTTGTCTTTGTCTTTGTCTTTGTCTTTGTCTTTGTCTTTGTCCCAACATGAAAAGAACTGCTTGTCTATAAGGGCTTCCAATGTGATTGTTCCTACGCAGTTCTCTGCTTAAGG

>Ca1:25053017-25053220

TCTCACTTTAAAAATGATTTTTATCTTTCAAATTTATTTTGTTCCATTTTAACCTTACTAATGAGCTTTGTAAAATGAAACATGACCATGACACCATACATCAACAACAAAGAACAACATATCAATTTAAATTTATAAATAAAATATTAATTTCTTCAAAATAATGTGATTCACTTCATGCATCAATCTGAAGCACCATCAAC

>Ca1:25422601-25422893

ATCCTCATAGAGCCATCTTCGGTGTCCTCGATCTAGTGACTCTTGTAGGGAATGCCTCGTGGTTTTGGGAGTTGGGTCCCCACCTTTTAAGTGAAGTGTCATAATAGAGTAATCTCACCGAACCTTATATGAATTACGACGTCTTTTGGGATGTTACCACCATAGGGGCGATTGGTTGTCGGTTCTCCACGTTGCTGAGATGATAGTTATGTTCCATGATCACTCGGGATTGCTCACAAATTAGAGTGACGAGCTCATTGGAGGTTGTTCCTCTATTCTAAACTTCCACAAG

>Ca1:25471087-25471479

CATTGCTTGAGGTTCTGATATATATGAACCCTCTAGAAAAGGTTAATTTCAAACTATTATATATAACTCAAAGTTCAACCATTACATATAACCCAAAGTTCTTTCATTGAGATCATGTACACCCTAATGCATATGAGTAGAATATGAAAACATCAGTGTGATCACAAACCACTATTCTATAAGATGTCATTCAATCCGAGTTTTTGAATGCACCATCTCCATTGAAAGTCGTTCGACCATCATCTAGATAATGTCAAGCAAACCAGCGTTGATGGAGAGTCGACAAACTTGCAATACGAGCTTGAATTATATGAGTATTCCATATAGAAGTCGTTTGATACTTGACTTTGGTGGCTATTACTTGCCTTAGTGGTCTTATCCTTGATCCCATC

>Ca1:25894188-25895256

CACAGCGACAAATAAGTCAACATAATTTACAAGCAGCGGAATAGTTTTTGAAATACGAATCCAAAGTATTTACACCTCAAAAGTGGTACATGGAACCCATCGAAAATAAAATGTCAAGCTGACAATATTGGTGTAGGTACAATATTCCAAATTAAAATGAACTCATCCCAAAAGAAAGACTTCTAGTCCCTATGCAACCACCTAATCTGATCATGCTACAAAACTACACCACCCTGGTGATCTCCACGCGCCCTGCAGGATCCTCCTGACACAGCTTTGGTCAAGTATGACTAACTACTTTCCCATCTCCAGGGTACTAACCGGTAGGATGATCCTGGTTCTTATCTGAGGGCAAAGCCCAGATTTCCACAATAGTTGTAAAGGTCACCAACCGAAATTAACAAACATCACATAACAAAATTCTTATATTTTCAAATGCTCAAAAGAACCTTTCGTCTTAGCATACTCCTCGAAAGGGTTTTCATATGTCCAAAAATGCATAAACAATGTTGAAAAATGAAGTTTTGACAAACTGCATTGTCGTAGCCGAATACAATTGAGAAAAGACAGATTTTCAATTCTAAAATGGCTCTGAATCAATCAACACTTCAAACATTCATGTTATCAACTATGAGCACATAATCAACACCAAATTAATCTCCACAAATTCACACCTCAATCACACATCGAATATTCATTAAATCAATTATGAACACATAAATACATCAAAACTTGACCAACCTTACTTCACACCTCAAAGTAATTACGACAAATCACAACAACAACCGATTATGTATATACAATCATCAAAGTATAACAAACATGACTCGCGTGCCAAGCACCCTAATGCAATGCGTATATGCCAAAATGCATCATTTCAGAATTCCAAACCAAAACCCTCATCGAGGGGCGTAAATCATAATTGTACAGCCTCTCACAGACCAATACTAATTACCGAGGTGCAATCTCTCACGGATCAGCACGATTTACTAGCGTGCAGTCTCTCACAGACCAGCACGACATACAAGAGTGCAGTCGCTCACAGACCAGCACAATTTTCTAGAGTGC

>Ca1:25896018-25896704

ACGTAACTCAAAATCATCAAATTTAATTATCGCCAATTCACACCGACAACAATCAATTCAATTCCACAACACATATATAACACCTCAATTAAATTTCAACAATTCAAACATAATTCACCTAATTTCAAAACTCCACATTTTTACACATAAACCACCAAATTTCATCAATTCATTTTCTACAAAATCCACAAATAAATCACATCTCAAAAATTCATAATAATAATTATAAACATATAAATCACACCAAACTTGAATCACCACAAACCACACCTCAAATTTAAATTCCACTAAATTACACATAATCACATTAAATTAATTTTTCACTAAATTACACCTCAAATACATCAAATTTCTTTTCCACAAAATCACAATTAATGTCCTAAATGCAAACTAAAAGTTTGGAAGGAGCCCTTACCTTAACGATAGCTCTAACACGCGATTACGGTACCGTAATTAATTCCGGTAAAATTTAAGTTCGTGATGTCGCGTCGAAAACTAGCTCACTAGCACCGTAGCGTGGTAATGAGCAACTTTCTCTTCTGTCACTTCTCGATACGAAGCTTAGATCTAGGAGAAAAGTGAAGGTTTTTGTGTGTTGTGGGTTTTGAAAATAAAGAAAATTTAGAAAACAGAGAAGGAGGAAGAAGATGGAAGGCACACGGGAAAGCTACAGGGCAAAATGTTAT

>Ca1:26807010-26807462

ATCGTCAGTTCCTGTGTCGTTCTCCATGTTTGGTTCTTGAAACTTAAACAGATCAATCTCACTGGACTCCGTACAGCAGGACAAAGAGTAACATGTGCAGTTATTACCAAAAGCTAGCTCCCAGATTAAAAATTGAAAACAGGTCAGAAATAAATGTCCTACCTTATTATGATGTTCCAAGCAGTTTTTTTGCTTTACCAACATGAGGATGTACATATGGTAGTCAAAATCGCAACCCAACACAGAATCATGCAAGCCTGCGATAGACCAGAGAAAGAAAATATATAACTAATGCATACTGACCGGCCTCTGAAAAATTTAAACACCCGATTTTTCAAATATGCCCGTGCAGCGGCCAACGACAGCCTGATTAACGCCTCCAACTGCCTAACTGTCATACGATATGTATCACTCCTACGGAGAGTAAGAGTCTACCATGGTATCTGAATGAG

>Ca1:26916655-26917000

CAATTGGGTGAAAATGGAGTGGCGGGCGGCTGAGGTTGTTCCAATCATGGTGGCCCATAGATTCCTATAGTCTCTTCTTATGCCACCCTCTTACAGAAGAAAGAAAAATCTTTTCTTTTGCGTGATACTTTCCTCTGCTTTCTTTGCCGTTTGACTGAAATATCTCAGAAGAGGGAAAGCTCTATTGCAGTGAACGGAACTCTGGTGTGCGAACGCGCGCAAAGTCGTTCTCTGTTTCGCGAATCGTTGGTTGTTTTCCCATATGTTATTTCTGAATTTTCGTTTGGTCCCTTTATTTTTATCTGTTTCGTTTGTCATATTTTTCTTTCATTTTTTATTTATAAG

>Ca1:26921151-26921522

CTAATTTTAATTTTAATAAATATATTCAAACATAATTCACTTTATTCAACATACTTTTAACTAAAATCTATTTAACAAAATGAATTCATTCAAAATTAATTTTATTCTCTGTTAAATCAAACTCACCCTAAATTAAATTTAGTAGTAACTCTTTAGTACTACATTATAAAAAGTATAATAAGGGGCAACTTAGGTTAATATTTCATGATAATCAGATGGGCGGCAGTAGTGTTGTCAGCAGCATCAAGGGCATCAGTTCACTCAGCAAATCCTCCTGCATCGTCACTGATGGGCCCCCAGACATCAATAGCTAATCCAGCAGCAACGGTGCTCAACATCTTAAGGGCAGCCACCACAATACCATAGCAAAA

>Ca1:27099177-27099666

TTCACAAATGATACTTGTCTACATAGAAGAAAAAAAAGAATCACTTAAAAAGTATTTATATTGGATTTGTGACTGCTTTTCTACTAGCTTAGCTACAACACTTTCAGATTATACAAAACTGATGTATATTAGCCTTCAAACACACGGCCGGAGCTAAGCTACAACTACAAGCTACTGTGAAAACTTCTTTCTAAGTTTGCTGATGGGCAGACATAACTCAAACATTCCATTGCAAGAGTGGTTGATGAAAGAATCAATCAGCAGATTTCAGAATCAAGAATGTTCATCAAAATAGAATCACAGGCATTTGATACTCCTTTTGGCCAGACAACAACTTGTTTAGCATTATCAAATACCTTTTTCAATTAAAGGGCATTGACACTAATTTGTGCAATTGCATGATTATAAAGAAAAAGTGAGCGACAGTTGACAACGGATAACAATGTAGCAAAAGCAATTGGTAACAATGATAAGTTACAAGCATAGTAC

>Ca1:27122066-27122289

CTCCAATAGTACAGTCAATACCTCCCGGTGGTTGAAGGCGACAATGGTGGCTTCACGGCGACGGTGGCTCGCGGGAGATGATTAATGGGTAAGAGACGTTCAATCAATACTCGGCGGAGGGTTCAAATATTCCTCCACCCAACGTGTCTTTGGTTTAATCGATGGACGAATTTTAAATGATTTTTTTTTTAGTTGCTTTGATTTTCTGTCATTTTTCTTCTAG

>Ca1:27119235-27119724

TTCACAAATGATACTTGTCTACATAGAAGAAAAAAAAGAATCACTTAAAAAGTATTTATATTGGATTTGTGACTGCTTTTCTACTAGCTTAGCTACAACACTTTCAGATTATACAAAACTGATGTATATTAGCCTTCAAACACACGGCCGGAGCTAAGCTACAACTACAAGCTACTGTGAAAACTTCTTTCTAAGTTTGCTGATGGGCAGACATAACTCAAACATTCCATTGCAAGAGTGGTTGATGAAAGAATCAATCAGCAGATTTCAGAATCAAGAATGTTCATCAAAATAGAATCACAGGCATTTGATACTCCTTTTGGCCAGACAACAACTTGTTTAGCATTATCAAATACCTTTTTCAATTAAAGGGCATTGACACTAATTTGTGCAATTGCATGATTATAAAGAAAAAGTGAGCGACAGTTGACAACTGATAACAATGTAGCAAAAGCAATTGGTAACAATGATAAGTTACAAGCATAGTAC

>Ca1:27115106-27115324

CCACCATCAATTAGAAGGGAAACACAAAAGGCACGAATAACTGTTTAATCGCTTGACAGAAATATTAAAAATCCAGTGAATAACGAAACGAAATCAAAATGAACAACCTTAAAATCATCATAAAAATCAAACAAGTAACAATACAAATTTTTAGAACCAAGAGGCAGCTTTATCTCATTGATAGCCCTTTCCTCCTAAGGGTGGCCTTTCCCTTAGGG

>Ca1:27130517-27130773

TCACCATCTTGCTTCACTAGTTGGTTTTTGCAAAGAAAATTCACAAATGATACTTCACTATGACTATATAGAAAAAAAGGTCTACTGAAAGAAAGAACAAAAGTATTTATATGGAATTTGTGACTGCTTTTCTACTAGCTTAGCTCCAACACTTTCAGATTATACAAAACTGCGGTGTATTAGCCTTCAAACACGCGGCCACAGAAAAGCTACAACTGCAAGCTTCTGCGAAAACTCCATTGGCTCCAATGCAAGA

>Ca1:27135413-27135958

CTCAGAGAAGAGCAACGAAGAGTGAAATTCACTATGAACAAATTAAATAAATAAGGTTCATAAATCAAACCTCAATAATATCTACACCCAATGATTGATTAAGAAGGTTTAGCTCTCCATTGATATGAGAGAACACATGGAGTTACAAATTACAAAAGAGAGAAAAAATTGAAAACTTAGATGAAGTATAAGCTACAAGAGAAAAGCTCTCCCAATCTTCTCCTTTTATCCCCCCTTTTTCGTATCTGCCTTCAAATATTTTCTTTTCTCCTTCTCAAAGTAGACTGACTGAGTATTTTCATGATCCCTAACGTGCCCCTCTGAATTAGCTTTCTAAAATGCTATTATAAACCTTAATTGAGTCTAAGATGAGTGAGTTATGGCCAATTATATGAGGAGAGGTGTTGGGCTTGTGACATCAACGACCCAACCTTGATTTATGCCAAATCTGATTAAGTCTGAAAAACAAAATTGTAGTACTCTGTCTTAGCTTTTCAAGGGTACAAAGATCACCAAAATCCAATTTTTATAGCTCTAGATATGTC

>Ca1:27221104-27221487

GCAGTCTATTACGGACCTACATGACGCGAAAATCCCCACAAGGAAAAGATGAACCAACAGATCACATGGCATTATCTCGTGGCCCATCACGGTCACCATTTTCACCATGGCCCATCGCGGTCACCATGTACTATGAAATGTATGAGCATACTAAAAACATACTAAAAGTTTGAAAGGAGCCCTTACCTCCGTTTTAGCTTTAACAATCGATTACGGCACCGCAATTAATTACGGTAAAATTTCCGCTCGCGTCGTCGCTTAAAAAACTAGCTCACTAGCACTGCAGCGTGGCAATGAGCAACTTTCCCTTCTGTCACTTCACGAAAGAGAGTTTAGATTTAGAAGAGAAAGGAAGGTTTGTGTTTGAGTTTTTTTGGTTTTCT

>Ca1:29211766-29211977

GCTTCAAAATTACAAAAGAACACAACAAAAACACATTGATAATATAAGATTTATCATTAATTAACAAAATTTGGAAATATTGTTAGCTATTCTTTATTCTAGATCCATGAACCCTTCAATTATTCTCAATTTGATGTGGTTGTGGCCTTCCATAAAAGCATTGGTGGATGTGGATTGATTGCCTTGAATTATCAATTGTTGTAGTGGGAGA

>Ca1:29219022-29219465

CTATCTATTTGTTTGTAATTGCTTAATATGTGTTCACTGTCTCTAAACATTAACAATTAAGTAATGGACAAAGTAATTAGCACCCCAATTGGATTATTATGCAGACTAGAATCATTACATCATATTTTTCATGCAGAATGATCCATTCTTGTCAACGGCTTCTTATCCTGTTCTTTTTCTTTAAAAATGGAATCAAGGGACTAATGTCTTGATTTTGTAATTAAATTTATAAGAGACTATTTGTAAGCTTTTTTTTTTTTTAGAGAGATATTAAACTAGATGCCACATTTTTTGTCCAGACTAGACTGGAAACATGAAAGGATGAAAAGAAAGGTAAAGGAATTTGCAAGCAGATACTATACTGGTATGTCAAGTCAGCTTTATTCTCTAGAATAATGCTCCAAGCCTCCAACCAACAAGTTCAGGGGGGATGTTGAGGGAGC

>Ca1:30445108-30445491

CCAAAGTTATGATAGCCACAAGCTAGCTATCTAGCCGAATGAAATATAAATGTTGTGTCTAGGGGTTTTACTAGCAATTAATATCCTTGTTCAGCATTGTATCCTCGTCAAGACATGTGGGATTTCTTTATTTCCTTCTACATCAGCCAAGCAGAACATTTATCAAGGAGGCCGATGGATCAACCACAACAACATATGATTGATCCTTTGTAGAGGAACCATGAGTTTTGGTTGCGGGTGTAGAGTCTAGAAGTCTCATTTGTGCCATCAACAATTTGAAATTTTCTCATTCTTGTTGATAAGTCTCCAGTCCTTCATTCTTGTTATGAAAATCCATTTTCATGTCGGGCATATGTTATAGTAGACGATTCAGATTGCCATCA

>Ca1:31187253-31187673

GCAGCACAATAATTGTCGAAATAAAAATCCAAAGTATTCAAACATCAAAATACATCGGGGCACTGAGGCCTATCAGACATAGCTCACACCTCTGGGGTATTCTCGGCAGACCTGTACAAAATCACTGCCCCAAGAATTTTAACTCCCCACACGTCACATCAAAAAGTGGTACATGGACCCATCAAAATAAAAGTCAAACTAACAATATTAGTATCGGTACAATCTTCCAACATACAATAAATACATACACGAATGAAAGACCTCTAGCCCCAATACAACCGTCTAATCTGATCACACTAGAAAAACTATACGACCTGAGTGATCTCCACACGACCCGCAAGATCCTCCTAACGCAGCTCCAGTCAGGTGTGTCCAACTACTTCCCCATCTACATGATACTAACCGGTGGGAAGATCCTGG

>Ca1:31300276-31300776

TGCAGGATTCAAAAGGTAGTTTCACAAATATTTATTTCATGCTAAAATTTATGATTTCCCGTACCCTCAAATTTTATGAAACTCCATAAATCATTTTAACTCTTTAATATTCATTAGAATCAACACCAATAAACACCATAAAAAAATAAAAAAATTTAAAGAACTCATTGCTTTATACAAAAGTTTTCTCTTCGATTCTAGTTGATTTGTGGTAGCTATCTACTTCCCAGTTTTCTACCTCTTCGGTTTTCGCACCTTGCTCCAACTTTTTTGAAGTTTTTCCTTGTTGTTGCTTATCTTCTCCTCAATACGATTTGATCGATTATTGATGGTTGAGGGATGAGATCGTGCATGCAATCGGTCAAAGCAAGTCTGATGAGGTTTAGTAAATTAAAATTTTTGGAGATTTTGAAAAAGAAAAAGGAATAGAGGATTTGGGGAAGATGATGAACAATACGATTTTGGGGTTTTCTCCCCGTTCTTGGGATCGAGAAGGAAGA

>Ca1:32964589-32965064

TGAAAACTTTGAACGTTACAGAAGTATACAAATAGAAAATACATCGGGGCTATGAGACCTATCAGACATCGCTCATATCGCTGGGGTATTCTTGGCAGACACTTGCATAGAAATACTACTCCAAGAATGCTATATTCCCCATGATCACGTCAAAAACATGGAACATGAACCCACCAAATAAAAGTCAACTATCAAAGAATGTCATAGGTAAAGTCATCCTAAAACAAAATAAGTACAACCACCAAAAGAAAGACATTAAGGCCTTATACAACAAAAAACTAATTTGATCATCCACTAAACAATAACATGGGCGACCTCCACACACTGCACAAGTTCCTTTTGACATCAGCCATGGAAGGATCAATCTTTTCAAAGGTTAGGTCCACCGATGAGATAAGTGGCTTCGTTGGTGCAGGAATCTCAAAACAGCTCACTATTGAAAGTGGGAGCTCTTACTCCACCCATATATGTAGCC

>Ca1:32990072-32990480

CTCAACATCCAGAACCAGAGCATTGCTTGCATTATCGGAGTCAACCATCATAGAGTGTCGCTCATGTCACTTGAAGCGCGTGTTAGAGAACTTGCATTAGACTTAAAAAAAAACAAAGTGAAGCGAAGTCTTGTTGTTCTTTTTCAATTCACTGATCTAAGACCTGCACACTTAATCAAAATATTAGTGTATTAAATTGTTCCCACAAACTAATTTTGTTATCATCTAAATAAATAATGAGCATAGTTCTTCAAACCAACGTTGTTCAGACAATGTCCACAACTCATATGGAGATTGTGAATAAATAATGGATGGTGTTGCTGCTTTGCATATTCATGCAGAATAAGATTTATGGTGGGCTCTTGATCTAATAGGAAATTCAGAAGTGTGCTTTTGTCACAAATTTAT

>Ca1:33400096-33400378

TGTTGGTGATAAAGGAGAAGACAATGTTCCGATTAAATTGGTCGTTGAACTCCCCTTTTGCCTGAATTGCCACTTTGACTCTTCTTCATTTGTTGGTTAGTATCCTCTGTTGCTTTGATTGAAACCGGTTACTAGTTACAAATTGAGACATGCAAGGTTTAAAAATGGGTTTAAGGAGCTTTGCCAAGCTTTTGGGTTTGATTCATTTAAAAAAATGAAGGAAAATGAGAAGTTGAAGATTGGGAGGATGGGAGGATGGATGCTCAGGTAACGAAGAAGAAA

>Ca1:34159828-34160046

GTGGAATTTGCTAATTTACTAACTCATATTTTAGTTGGTATAAATTAATATCTTACACCTTTATAATTTACACTAAAAATCCAAGTAATTTTTTATATAAAAAAACTAGTTAATTAAATGGATATTAAGGTAATTCCATTTTTTTCTCTCTCTATCCTTGATCGTCCAGTCGCGCCCACGTGTCTGTAACCGTCACCTCCACATATCATGGTTTCGCC

>Ca1:34163213-34163459

CGGGTTAAAATTAATAGAAATTTAGGGAGGCGATGATCATGAAATTGAGAGAAAGAAAAGGGGTGAGGTTTTTGGGGGTGATAAATGGGTGAATGTGAAGAACAAAGTTTTGATGTTTATAGAATTCAAAGAAGCTTTTTGAAATCTAACGGAAATATTTCAGATACCGTTTCCATTCTCCCATCAACAAACAACAGTCATCCTCATCACAAAACTAACACTGCGTTTGATTCGTATTGAAGTTAG

>Ca1:34401971-34402233

CTTTAACTTTGATCAATAATAGTTCCTCTTCCTGCTATGATTCAATTCAATTTGACCTTGATGAAGTTTCAACTATATCATCCTCTAACGGGATATATATCAAGTTGACTTTACTGATTGAACCACAAAATAATTGTTGTTAAGTTTTTGAGGGTTGATCATGAAGAGTTTCATTATCATTTGGATCTTCTTGGCTTTATATCCTTTCTTATATTTCTTGCCCTGAACACCTATACTTTTTCCTCATATCAAAACAGAATAC

>Ca1:34477269-34477648

AGGAGGAGAGAAGAAGGGTGGGATGTGAAGCCTAACATAATGATGAGTGTATGCTTTTTTTTTTTTTGTGGTGATTAAGAATGTGATGATGATAATTAAGGTTTTTGTTGTGGTTTAGGTTTTTTGTTTGTGTTTTTTCTTGCGGTTACCCCAGAGTTTTTGGGTTTGCAGGGATAAACAGAGGAACAGAGTGGGTGGAAGAGGTAGAGATATTATTGCTTTATTATTTGTTTGATGAAATATGGTCGACATTTCACATATTTACACACTTCAGCAACAAACAACATACTTACTTGGATGGGTATTCGTTCGTAGGCCCTCTATATAGCCTTTGTCGCTTTTGAAACGATTTCTTACCTAAAATGCCCTTACCTCTTTC

>Ca1:35359714-35360016

CTTCCATGAAATTAAAATAAAAACTGTTTATTTCTTGTAGCAATCTAAATCTTAATATTTACTTTGTAGAAAAGAAACAAAATTGTCAATATATAGTACTCGATATTGGACTTTAGGATTTAAAAACAAGTGATCCACTAATTAAAATTATTACAAAAGTCTATATTACAAAATCTAAAATTTAGGCACAGTAAAGTTCGGAATTGGGCTTTTATTCCATCACAAAAGTTGTAGCTCTGATTTTTAGCTTTCCAATGTCTGCTCATATGCGCCAATCCGATATCTAGAACTCAGGTTATGAT

>Ca1:35374058-35374265

GTGTATGTGAGCCCGCATGTGTGTGTGTTATTGCAAAGATAGATAAATATGTGCCATAAGTTAGTATCCAATCAAGAATATAATTAGATAAATAAAAAATAAAGGAAATATGTTATTTAAAATTCAATTTTTCAAATCAACATCAATTAAATTACTATCTTAAGTATTATAATATCTTAAGTATTATAATAGGTAGAACTTTTAACT

>Ca1:35374403-35374641

GTATATATATATTAAAGTTCAAAACTTTGGAAAAATTACAAAAGGAAATATGATGTGTGTCAAAAATTGATCATGTTGAGATAAAAAATTATTGTTTTTAATGATTATAATTAGCAATGTGGTTTGATTTTTATGACGTTAATAAAAAAATTAATTTGATCCAATCTAATGAAATACAATTTAAATTGAATTAATTTTTGATCCGTTCAATTTACGATTATAATTTTTTTTTATAAAT

>Ca1:35742638-35742887

CTCGACAAAATAAAACAGAGATGGTGGCGAGCCGTGACGGTGGCGAACACAAGAAGGCCGAGTCGTGACCGTTACGGTGGTCGGAGAATGGAGGAGAAATTGGACTGAACGGATGAGAGATTAAGTTCTGAATTCGTCATAGATCACTCTCACTCTGAAAATGGCAAGTGTCGGCGCTGGGATGTGTGACGGTGGCGGTGACGACGACGATGATGGTGGCGAGCTTTGACCGATGGCTACGGTGAGAAG

>Ca1:36807660-36808313

GACAGTGGTTCATGGAAGACTCGAAACAATATTTATGGGTAACCGCGATGACACTGAAATTCAAAATAAAAAATGCATCAATGTCGAACATTAACTATCAATATAGGTCCGAAATAGAATAAACCACGAACATAAACGACGAACACGAGATTTGTCGGAAAAATATTTACCGTAGGGCCGGTTGAGATGAGTTTTCATGAACGTTGGTAAAAATATAAATGAAGGGTGTAGGACGATGATTTCGATGCGTAGATCATAGAAATATTCTATGTTGTTTTAGACCAATTGTTTTAGACCACCACTACGACGATCATAAGGCCTTGCGACCTTGTTGAAAGTGAACAAATAATGGGTCAACAACTTGATTCTTCATTCACGTGGCAGTTGAATGTGGGTGATTTTGTTGAAGATAATCAATAGAAAGGAGGTGTGTTGATCATTCAATCATTGAATAGGAACACCTTTTGATACGCTTTCTAAGAACGTTGCATCATTCGATTCGAAAAAAGGTGGAAAAAAGGTCATAGCACCAGCACCTGAACGCTTACTCTTACTGCTTCAGGTTCACCTCTTTTCTTAGGATTGGCTGCCCTGAGGTTCTTGTTTGGAGTCATTTGTTTATCCTAGAACAAGTCCCCCAGTCACTGCCATAG

>Ca1:37067011-37067282

TGTTTGTGACAAGGAAGAAGATGATGTTCTGATTAACTTGGTTGCTGAGCTCCCCTTTCGCTTGAATTGTCACCTTGACCCAAGTTCTTCAATTTTGGGTTATTCGTTGTTGAAGCTCATTAGCATTAGTTACAAATTGAGACATGCAAAGTTTAAAAATGAGTTTGAAGACCTTTATAAGCTTTTGGGTTTACTGATTTGAAAAAAAAAAAGAAAGGAAATGTGAGGTTGAAGATTTGGGAGGATGAGGGAGGTGGTTCACAGTAACACA

>Ca1:38269402-38270017

ATATCGTTGAAGCATCGATGTCAATATGTCAATCATAAACATTTAGTAGAAGTCGAGGTTCGGTTAAGTAGAACTAGTAATGATGCTATGTAAATATAGATCAGCTCATCAGAACACAACTTAGACACTGGCAAAATATTTTAAGGCTCTTCCATGTTAATACAGCAACACTTAGATGATAGAATCCCTCAACAATCGCATTCCCTAACTACAACCATGTTGCCAGGAAATAATATCATCTAATGTAAAGCAAAAAAACCATGAACAAAATGTTTCAGTGAAAGGAATCCACTTTAGCAATACAGACATAACACAAATGAAACCTGAATTCTACAATGATTTTATTATGACCCGAGCGAGGGGCATTTGGTTTAAGAGAATGTGGTTTCAAGAACCAACATACAGGGAGTCGAGAAGAGGAAGAGAAATGCACGTTACTTGTGCATTCCATCAGTTTATTGTATTTCTTGCTATAGCACTACAACTAGCTAGTTGGAGTTCCCCAAAAAAGAAGGGAAAAACAGAAGAAGCATAATCGTCTGGTGCAGATTTACACTTGATAAGACGATCTATGGTGATACAACTTCAATATTATTATTTTAGTACACTATATAC

>Ca1:39865093-39865363

CTTCTTCATATATATGACCAACATGGTTAAATCCAAAAGTCTCAATAAAATTTGAAAACAAGTAAACTATGATATTACTCTAAAAACAAGCAATTTTTATGTGATTGACACTTATTTCATTACTAACCAATTCAAGTTTATTAATTAGAACCAAAATATACACAATTTACAAGAAAACCTATGTCATAGCCTCAACTACCTCTTTGAAAATAATGAAGGCTTGTTGGCCTTATCATTTCCATGAACATTCACGAAGGCTGTTGCCAAATC

>Ca1:39865093-39865372

CTTCTTCATATATATGACCAACATGGTTAAATCCAAAAGTCTCAATAAAATTTGAAAACAAGTAAACTATGATATTACTCTAAAAACAAGCAATTTTTATGTGATTGACACTTATTTCATTACTAACCAATTCAAGTTTATTAATTAGAACCAAAATATACACAATTTACAAGAAAACCTATGTCATAGCCTCAACTACCTCTTTGAAAATAATGAAGGCTTGTTGGCCTTATCATTTCCATGAACATTCACGAAGGCTGTTGCCAAATCCTGTCATAA

>Ca1:40588377-40588848

ATGAGGAAGTAGCATACCAATGATGATACATCAGTAGCAGTCAATGTATTGAACAAACAATTAAAAAAAGTTCTTCAACATGTTTATCATTACTTTGATCCTCATCATTAGAAATGCTAACTGGCACTTTCTTTTCACCTAGATTAGACTTCGATTTCAACTGCACAAGTGTGAGCCTTGGAAGTTGGAACAACTTTTGTCGGGAATGGACATGTATGTAGAGTAAGGTTTGCATAAAGAATCATTGTCTAAAATATTAATATAGCTTCATCAAAGAATCTTTAATACATAAACATCAACAGTATTCAACCATATTCAACGAGAAAAAAGTTCATGTACATCTTCCCTGTGGCAAGATTTTCCTGTCTTAATTTTTCGGTTCTGGCCATAATGAGTTGCTCCATTCCAACGTCCTCTCATTCTCTCAAATTTCTTCAAACAAAGGCAGTAAATATGATGGTAGAGATCAAG

>Ca1:40589935-40590332

CTGCTATAATCATCATTTTCATAACTGTCCATGCATACAGACTCACTCAACACATAATCTACCATAGACTAGTTTTGAACCGTTTTCAAGCATCGGTAACAGTGGAATTTGCCTAATTTGACATAATAAAAACTACAAAACCTAAATCTTAAAAAAAAAAAAAAAAGGAAATAGTTATTTGAAAAGAGGACACATGAACAAATCTCATTGATAGTTGTTAGGGACAATAGAATAGTAGAATATTTGCAATTACAAATAATAAATTAAGAAAGTGGGAAGGAAGGTCTCAGATGAGTAAGGTTGAAAATCAAATCCTGAAAGAGAAAACAAACCACCATCAGTGTTAAAACTGGGCAGTTATTGAAACAGGATCTACAATATATGAATCTTCATTACC

>Ca1:40861465-40861713

AGAGTTTTATAGAGATAATAATATTGGTTTTTTTTATCATAAACACATAAACATCGATCACCCATGTTATTTGTTCTTATGATTGAGTTTTGAAAGGACACGGTGATACATGAGAACCAACATACTTATATATAAACAAACATAAAATAAAATATATAAAAAAATCCACATATGACAGGAGATATAATACAAATACATAATGAAATAAAATAAGAAAATTGTGCGATACACAAACACCCATGCAGTAG

>Ca1:40987372-40988061

GCAATATTCAAATCATCCAAGACCTAAAACTTAAATCTTCGAAACTTAAGAAACAGATAAACTTAACTTAAGTACCCAGATTAACAAATGAACAAGCACATTAGGTTAGGGTGTGAGAAGGTGGAACAGGGACTAACACCCTCACAATCCACTTTCGTTTTGTCACAAATTTAAGGGAAAGTGAGGGAATTATATTCGGTGGATGCATTTACTCTTGGCAACCATCCGATCATGATTGAATGGTCGAGATCGTTTAAGGTTAATGTGAGAAATCAGGGTCAGTTTGGGTGCCTCAACGAATCCATAAACATGAATGCAATAAAGAATAATTAAATGTAGGGCAAATTAAAAATTTTAAAATTTAAACATGAACAGAAAAAACATGTAAAAATTAAGTCTGCCCCAAAAGTAAATTAATATTGGCCGGGTGAAATAGAGATCTCAAAACATAGTTTACTGAGAACCCATTGGATCGGCCGAAAACACAGTTTGAAAATCAATATGAAATCAAAATAAATAAATAAATCTACGATAGTGACATTGAAAATTTAACGAAAGCTATGTATAAATCCTTTCAAGGCTTCTGCAAATTTGAAAATGAATTGCAGAAATGAGAGAAAAGGAATGCAGATTTGATTTATGCCCGCAACGGAACTCGGACGCAAATGTTTGGCTGCTACGCCCGGTTC

>Ca1:41054865-41055163

CAGAAAGCACTATTTAGTATCAAGTAACATTTAATAAAAGGAATTTGGTTGATCTTTCAAAATTGGCCCAATTACAATAATGTATCTACACATATTCTAAACACTCTTGGCAAAACAATACTGCATAATTGATAGAATATTTTTTGAATCTTCAAGAACTTTTGTTTCAAATTGAAATTATATATATAATTTCCATATTACTCTAAGCATAGTTGGACATCAACTTTCATATCTCACACTATTGACTTCAGAGGCTTTTTCCTTTCATTTTGTTAATGGTGTGTATATTCACATGTTC

>Ca1:41055287-41056047

CTTTGCACCTGCATTCACAAGTTGAACCTTTTGACTGCTAACTATAGTTAGTCATCGGAGAAACTTTGCTGAGATAACTTCACTTTGATCACAAAATTTTCTTCTTTAATATTCAACTATTACCAGCCATTCACATTAAATACTAAAAATTAGAAATAAACACCATGTTTTGCTTGTGTGCTCCTTTTCTCTTCATTGTTCTGTTCAAGTACTTGATTTATTTAATCCTTCCTCTTCATTCAAACCCTAGTAGTTACTTCAACAACCTCATTCCATGGATTGGCCTTAAATAGCAAAAACCTTTCAAATATATTTCTCAAAATCTCATTTTAATGTTTTAAAAAACAAATTGGACGTTGAACCGGGCACACTAGACTATGGTTCAACTATTTTAAACCAGTTGAACCATCCGGTCCATGTCGATTTTCAAAACATTGGTTCAAATTTTTGAACCAATTCTCACATGATGGCCATATAAAAGGAATGATCAAGCAAATAATGGAAGAGACTATCATATAGTTGCAACTTTGAAAAGAATAAACAATAGAAGTGCATGTGTGTGTAAGTGACCAATGGAATTGAAGAATGGCCATTCCTAGTGTTGAAAATGAAGGATTTGATTGATTTTGAGGTGTGACTAGAACAGAACAGTGAAGAGTAGAGAAGCAAACATGCAAAACAAGGTGCTGAATTTCATGTACACGTAAAGTGAATGGCTTGTAATAGGTAACTATGTGAAAGAAGATGAAGAAAAAGGTAG

>Ca1:41832111-41832652

GCATTATTGAAATGGTAATTTATTTTCCACTTAAGCATTTACTTACAAATGCAATTAATGTGCATTTCCAAGCTTATAATAAAAAAGCTTGTCAATGCTAAAAGGCCAAATTAAAAAACACAAAGCATACACAAATTATTGTGCACATATGCTTAATTAGAATATTGATAAGAAACATAAAAGGGAAACAAATTAACATTAAATAACCAAAGAAAGTAATTTGCAATGGAGTTACATGTCAATCTTTCTTTTCTTCAACCTTTTCTTCTTTTTTTTCTTCAACCTTTTCTTCTTTCTTAGCTTCTTTCACTTCCTTGACTGCCTTCACCTTAGCCTTCCCTGCCTTTGGCTTCATGAAACAAAAGCATGAGCAGCAAGGACACTGGAACAAGAACTTCATTTCCAATCAAGTGCTCAGATCTCAATTTCTGAACAGAGAATGAAAATGTTCAGCTTTTCTGTCCTCTTTTTATCTCCTTTCTTGCTTAATAATTTATATATTTAATTCTCTTTTGTCTTTTACTTTTTCCTCTATTTTTTC

>Ca1:41892384-41892705

TCATTTCTTTACATTTTCATCTACAATAAGATAAATAAGATTCATTTTTCTTTGTTGCTAAAAGTAAACAAGATGCTTTTGTGTACGACAATTAGGAGCTCAATTACCTCTAGTTTTACAATGATAGCAGATACTTCTATTAATTTTGTTATTATGTCCACATTTTCTTTTTCATTCTTTTCAATATTTTTTTTCATTCTTGGGAAAATAATGTGTTGTTGTGTATTCCACCGTTATCATGATTCACGATTAAGACCGCAATCGAGACCACGACTACGACCACGGATTTGCCATTGTGGTCGTGTGTTGCTATATTCACTT

>Ca1:41892912-41893479

TCGAAAGTCAATGTTTTGTAAGATTTGACATACTTAGTATTATTGTAAAAGTGTAAAATTAAGCAAGCTAAATGAAAATATAAAATTGAGAACAAGAAAAAAAAAATTAGTTAAACAAAATCAATCAAATTCAACTAAAAAGTATAGTCATGAACAATTAAGCTACAAACCGATATGGAGCAACAAGAGCATTACTCAACTCTTTAAATTTTCAAATTTTCAGCATGTCATTAGATCGATGAATTCAGCAAAATAGAATAAAACACACCGAAAATTCCTCTAAATTTAAGCAACACCAATATATTCTCCATATATATACCTCATCAATCTAAGAAGAATCAAATACTTAATAAAAACAGAATTATTGTATAATTGGATTTAATAAATCAAAGCAAAGTAGACAACATAACAAGTAAAAAGGATGAAAGACAATATCAAAGAACAAACAAAGCAAACTTACTAGTAGGAACCAATATTATCTCATGTAAATAGAGCTTCGTGATAACGTATTAAAAATAAACGAAAAGAATATGAATAAGAATGATAGATGAGAGTTTTGTATTATTC

>Ca1:41940414-41940697

TTTAAATTAATAAATTTATAATCTATTTTATAAAAATAATGTAACATAGTTAGGGAGAAATTTGAGATCAATTACGTCAAATATAGGTATAACTATTTTCTAACTGAGAATAAATTATGGTTACAACATGAAGTGTCACACAATTTCAGCACTGCTCTGCCTTGCTCCCAACATTAAGAGCCACTGCCCTGCCTTACCAATCTATAATTGGGGAACCACTTGAGGAAACCTTTAATATGGCATTAAACAGTCTATAAAGATGCTTCAATACTTCAAACACAAC

>Ca1:42401735-42402151

CAGCGGAATAGTTTTTGAAATACGAATCCAAAGTATTTACACATCAAAGAGTAGTACATGGAACCCATCAAAATAAAATGTCAAGCTGACAATATTGGTAAATGTACAGTCTTCCAAATTAAAATAAACACAACCCAAAAAGAAAGACATCTAGTCCCTATACGTCAACCTAATCTGATCATTCTACAGAAAACTATACATCCCAGATGATCTTCACGGGCCTCGCAAGATCCTCCTAACACAGCTCCAGTCAGGTGTGTCCAACTACATTCCCATCTACAGGGTACTAACCGGTAGGATGGTCCTGGTTCTCATCTGAGGGCAAAGCCCAGATTTCCACAATAGTTGTAAAGGGTCACCAACCGAAATTAACAATTAACACATAACATTTAAGTTTTTAAATGCGCAAAATAACC

>Ca1:43638858-43639156

GTGTGGGAAAAAAAAAAGAGAAGAAGGGATAGAAAAAGAGAGAAGAGACAGGAAAAAAGAAGGAAGAAAAAAAATGGTGAGAGAGAGTGTGAGAGGATCTCGGAGAGTGAGAGGATCCCTGAGAGGGAGACGAAGGAGAAAGAACCCATGAGTGGGTGAATAAGATGAATATGAAAGAGAAAGATGTAGAGCTCCAACAAGAAAAAGAGGAAAAACTCAAAACAAAACTATGACACTCTAATCTCTTATGTAATTTCTCTAAGGTAACATAGAAAACTCTCTTAAAACCTCTCTTAGG

>Ca1:43850272-43850556

AGCCATATAAATATATCAGTCAGTATACCAAACCAGTACATGCATCAATACCAAAAAATAACACTCAAAAACAATTGTCTTGGAGACCAGTACAGCTAAACTATGTCTTGAGAGAAGCTTCCCCTCCCAACCAAACCTCACAACATTAGACTGGAACTATCTAACAAGTCATGAAGCACGTCATAGAAGGATGGAAAGTATGTCTTCTGCCAATCGACCTACTTGGATCTCTGTCTCATCTTTCGGCGCTTCCTCTTCAACCTCCTCATGCGCTTCTTCTTCCA

>Ca1:43893170-43893494

CTACAGAACTAGATAAGCTCAACATAATACCACCACAGATCTCGATATAATTTTTTTAATTAAAAAAAAAAAGCAAAAAAGAAACTTCAACAAAATGTGAGAGATTCCATATGTTTACAAACAGTAACAATGAAATTGCACTGAAAAAACTTACCTGAGTTAGAAATAAGAACTAGATCCTAAATTCCTAATCATTAACATAAACATAAACTTAATCAACAGCAACAACATCTGAAAATCCCACCTCATCCTGCTTATTTACATTAATCTAAATCTAAACGTGTTAATAAAAAACTAACCCCAAATTTAACAATGTGCTTCCAT

>Ca1:43959474-43959719

ATGCATTACATCCCAAACATCACAGTTCACAGTAGCAATTTTTAAGTCCCTAAGGTATATGGGAATCTACATGGTGTGGAATGTTCAGATTGACAAAATAGCAAACAGCACTTAACCTTTTGACATGAAGGGTAACATGTAAAAATCCGGCATAACAAGAAAAGTAACATAGTTGCCAATAGCTTTTGACAGTGGCATGCTGTCAGATGGTCATCAATACCCACAATCTTCTTCAACTTGACCAT

>Ca1:44063176-44063809

TGGCGTGCGTGAAGTATGTGCAGCGTATCAGCTTAGCAGGTAAACAAGGATTCAAAATACAAGCAGAAACAGATTTAGTACAAAACTAACCCAATTACACATCAAAAATCTCAACCAAAACTCTATTCATACACATCAAAAACAAAAATTCAATTTTTCAATAATATACAACAGCAAACCTATAAATGAGGAAAATACCCTTTTCCGTGGCGCGGTGGTGGGTTGATTCTCGTCCTCCGCTTGTTGTTAGTGATCGATTATGTTTAAGGTTAAGGTTATGGGTTGATGGGAAGAAGTATGATGGCAGATTCTTCTTCTTTGATATTGCTCTATTTCTATTGTATTTTGGAAACCAGAAGGAAAATCCTCAATTTTATCGTCTGATCTCCTCCTCCCCTTATTCAATTTTTGTTTCAATATATAGACCAAAACGAGGAAGGGTTGTATCCATTGTATAAATTGATTTCTGGATTTTAAACTGATTTGGCAACTACATCAAGCAATCTGGTACAAATTTAAGGGGTGGTTGTTGATCTGTATTTTATGGCAACTACATCAAGCAATCTGGTACAAATTTAAGGGGTGGTGGTTGATCTGGATTTTACAGGTGTGGGTTGTTATTCTGGATTTTAC

>Ca1:44106347-44106736

GCTTGAGCTTGAGCTTGAGCTTTTGCAGGCTGGGACTGAGCTTGTGCAGGCTGGGACTGGGACTGAGCTTGCTGGAACTGGGACTGAGCTTGTTGGAGCTGGGACTGAGCTTGCTGGGACTGGGACTGGGACTGAGCTTGCTGAGACTGGGACTGAGCTTGTTGGGACTGGGACTGGGACTGAGCTTGTTGGGACATACCTTGTGCAGATTGTAGTTTGTTGGGTCTTCCTCTGCGTTTGACAGGTTGTGTGGGTTGGTTTTTCTTGGGTCTTCCTCTGGTTTTGATAGGTTGTGATTGGGCTTGGGCTTGGGTCTGGGCAGCTGTAGTACCCTAATTTTGTCCATTTTGAAAACAAATATAAAAAAGGAAGTATGTATTTAAAAAAAG

>Ca1:46462083-46462413

AACACTATTGCATTAAATAGCAGAACTACAACAGTATAAGTGATCATAGCACAACAGAATAAATTTACTCCTTTTATAATCAAACTATAAATAATACCTAAACATAACCCTCCTACTCATGCATTCTAATTTCAAAACCCTTATAATAATAAACGCACTAATTACGCCATCTAAACTAACTCAAAGCCTCTTCTCCATTTCAGATGCCAAAAGCAAAATATTGAACGACCCAACAAGCTTTGTTCTGTTCTTTTACCTTCACCTTTCTTCCATAACACTCTTTGTTCCCTCACACTCAATGGCCATCCCATGCGAGCAAACAAAACCCTT

>Ca1:46462083-46462420

AACACTATTGCATTAAATAGCAGAACTACAACAGTATAAGTGATCATAGCACAACAGAATAAATTTACTCCTTTTATAATCAAACTATAAATAATACCTAAACATAACCCTCCTACTCATGCATTCTAATTTCAAAACCCTTATAATAATAAACGCACTAATTACGCCATCTAAACTAACTCAAAGCCTCTTCTCCATTTCAGATGCCAAAAGCAAAATATTGAACGACCCAACAAGCTTTGTTCTGTTCTTTTACCTTCACCTTTCTTCCATAACACTCTTTGTTCCCTCACACTCAATGGCCATCCCATGCGAGCAAACAAAACCCTTCTTCGCG

>Ca1:46462083-46462410

AACACTATTGCATTAAATAGCAGAACTACAACAGTATAAGTGATCATAGCACAACAGAATAAATTTACTCCTTTTATAATCAAACTATAAATAATACCTAAACATAACCCTCCTACTCATGCATTCTAATTTCAAAACCCTTATAATAATAAACGCACTAATTACGCCATCTAAACTAACTCAAAGCCTCTTCTCCATTTCAGATGCCAAAAGCAAAATATTGAACGACCCAACAAGCTTTGTTCTGTTCTTTTACCTTCACCTTTCTTCCATAACACTCTTTGTTCCCTCACACTCAATGGCCATCCCATGCGAGCAAACAAAACC

>Ca1:46531522-46531971

AAGGGTATATTTTCCTAAAATCATTTGTTCGGCCAAATAATAACATTTGTTCGGCCATGTAACCAACCAACCTATTTACATTGACCTTCAAAAGAAAACTAAACTAAGATCTAACTAACACTTTTCTGAGTCCCCTATTAACACCTCTATAGTATACCACCCTTATGTACACCAATAATATATAAGGGAATGAATGACTTGATCATAAAAGGAAGGTAAATGTTCCAGTAAAATCATTGAGACATCAAATCAAAGTATCTGACTAAATCAGATCACGCCTTTTATTCATCAAAATCTTATATGGTGTTAGGAGAGACTTGGGTCACTAATTAACGACTAGTCTGGTTTTCCTGTTGTTCATATGGATCAATCCAACATTGGTCCTTCTTTGCTGCATCTTTCCATCTTTTACCAAAAACCTGCATTTCAATGTATGACTGCATCCTCAC

>Ca1:46773879-46774268

ATCAATATTTACAAGTAGCGGAAATAGTTTTTGAAACAAAAATTCAGACTATTTAAACAGCAAAATACACCAGGGCTATGAGACATATCCGACATAGCTCATACCACTGCGGTATTCTCGCCAAGAATCTCGACTTTCTCCATGTCACATAAAAAAGGGGAACATGACCCATCAAAATAAAAGTTAAGATGACAATATAAGATATATGTAAAATCATCCAATTTGAAATAAATACAACCACAAAAGAAAGACATCTAGCCCCTATACAACCATCTAATCTGATCATGCTACAAAAACTATAAAACCTGAGTGATCTCCACGCGCCTTGCAAGATCCTCCTGACACAACTTTGGTCAGGTATGACTAACGGCCTTCCCGTCGCCAGGGTA

>Ca1:46775742-46776128

CTTAGTTACCGTTTTTTCATCCGTTTCCGCTACCGCGCTCAAGTACGGTGAAAAATCTCGCTCGCGTCGTCACTTCAAAACTTAGCCCCCAAGCACCGTAGTGCAGAGAGGAGCAACTTTCCCTTCTGTTACTTTTCGAATGGAAGCTTAGATCTAGAAGATAAATAGATGTTCGTGTGTGTGTGGTTTTGAAAACCACCAACACTTTTTCTTTGAAACCGATAAAGAAGAAGAAAAGGAGAAGGCGAGGTGCAACAGGGAAAAACATTCTTCTTTTTCACCCACCAACACTTTGGTTTCTTTTCTTGAAGCAAAAGAAAGAAAAAGAAAGAAGAAGGAAGAAGGAAGAAGAAGGAGGCGTGAAGCAGGGAATGTTTACGTTCCCC

>Ca1:46775102-46775324

ACCGCCTATTACAGACCAATACTATTTACTGAGATGCCACCTATCACAGGTCAACACGATTTACTAAAAATGTGCAGTATATCACGGACCTATACGACACAAAAATCCCCGCAAGGATAAGATGAACCAACGAATCACATGACATCATATCGTGGCCCATCACGATCACCACTATCACCTTGGCCCCATCGCGGTCACTATGTACATTAAAATGCATGATCA

>Ca1:47060286-47060659

CTTCTATTCTCTAGTCTTCAATAATCCTAATAATACAACCCATAATATTTGAGATAGATGTAAAGAAACTAAAGAATAACAATTAGAAGAAAGGGAAAAGGAAAAATTTGTAACCACCACCCACTGTATCAACCAACGTGGAATCAGCTAGAGCCACCACCATCTTTGACCAACCGTGTACACCTTCGTCAAACCTAGCCATCGCCGATCACCTCAAGCTCCGCTCTTGTTGTCGTGCAACAGTCATTGCCTTCACCAAGTTACCGCCCACGACCTCCATGGCTACACCCCAATTGGTTTCTTTCCTCATTTTATCTTCTTTTACTTCACTTTCTTTAAAAGCCATATCTCTCTCAACAGCACACAACCGCAC

>Ca1:47314479-47314721

ATAAAGTAAATACATGTCTTTTAAAGATATATAGGAATGCCAATATCATGAAATGATGAAGCACACATTGATAATTATAGATGACAAGAGCAGAATCTATACTTTTTATCTTATAATCAACTTATTGTCATAATTAATTTTTGTTGTTTGCCAAATTCTCATTCTTCTTCTTTGTTCCAATGTTATTATTTTGTGCACCACCCCCGTTGCTTAGGTCTGATCTTCTTGGTCCACCTCGCGTT

>Ca1:47365504-47365735

CCCCATAAAGTAAATACATGTGGAATGCCAATATCACGAAATGATGAAGCACACATTGATAATTATAGATGACAAGATCAGAATCTATACTTTTTATCTTATTAATCAACTTATTGTCATAATTAATTTTTGTTGTTTGCCAAATTCTCATTCTTCTTCTTTGTTCCAATGTTATTATTTTGTGCACCACCTCCGTTGCTTAGGTCTGATCTTCTTGGTCCACCTCGCGTT

>Ca1:47817095-47817349

TAAGCATTTAATTTTCATATAATAATGATGAATACAGTACAGGGACTTACTCAATATTGCAAACTCAAAATTCTTCCTCAAACTTCATTCTAAAATACTCATTCAAATAACAAAATACACCCCTTGAGTCATAAAGACCATATGGAAGGGTTAAAACTTAACTTACGTACCCAAGAATATATATGATGGACAATTGATAACAAACAACCAGAATTTCCATGACTTTAGCTAGCATCAAGTAGCACATCCAAGGC

>Ca1:48061573-48062445

CTTCTTTGTCTTCTTGCTCAATGTTTAATAATTTTGTAGCCTTTTTCACGTGAGCCTGTTGCTCCGCCATTGTGGTCTGGTTGCTTCAGCTCTGGAGCAGTGCTCTTCCTTGTCCCTTGTTGATTGCCTGCTCCACCCTGTCCCTTGCCTGCCGCCACGGCATGCGGCGACTCCCTTTCTCCACAACTCCCAACCTTGTACCACACTCCCCTCCGCCATACCGGAAGGAATGAAGGTTAGGTAGATGAAGCAAATGGGAACCAGCGTGCAAAACAAAATGGTAGAGACATGATGCGGGGATAAATGCACTGGAAATAAGTAGAAGCAAAGTAGAGAAATGAGTAAGAGGGGCCATGAGAGATGATATTTGCTAGAGGGAGCAAGGGCCATCCTAAAGGTGTGACAGGTTTTACCACATTCTTGCGACAGATGCGTGGTGGGAAGCCGTAGTTGCAAAGGCAAGAGTGTGTAGAGGCACACTTGTGTTTCTAGTGTGCAGGTGGTTGCTTGCTGCGGATGAGACCGCGATGGCAAGTTGATTTGCATGAAGCTAAAGGAACATATGTTTACTGTGTTGTCTGCGGGCTTGTGAGGAATGATAGTCCAAGGCTGCTGTTAGGGCTGATGATTGTATCTCATCCGACAAAGATGAGAGGCAGAAATACTACTACTAGAGTTGCAGAAGTGTGTGGAGGAGGTTGGGGCCAACCCATTGTAATGCCATACAAACCTTTACTTTAGTCAGTTTAGTGAGCTTCTTTCTCTCCATCGACATCACAAAAGTCCTCAATTTTTGTCTGGATTGGCTAATTCGAAAGTGCCAACCCGAACATGTTTTTGAGAATTTAAGGGGAGAAGAAGCAACCCTTACT

>Ca1:48194582-48194790

TGCACATGGAAATTCCTATCGATATCGTAGGAATGCTATACTAAATTCACACTTGGCCAATACAACTAGTGTAAAATGTACCAAAATTGCAAGGGATTACAAAGGAAAATCAAAGGGTTTTTTTCAGGCAGACTCATTCTTGCTCTTGGGTTGAGGCACCATTTGTTGCATCCATGGGCTCAGCTTGGGCTTCACCTTCGGCACCCTT

>Ca1:48357121-48357495

ATCCAATTATTTAATTGAAGGGCATTTTCGTCAACTGAGTAAAAACAGCAATTCACAACAAGACCCTACACACACACACACACCTACATCCACTAGGTTACATTTTGCTAAAGACAAAACAGCATTTGGTCACCTTCCATATCCAATAAACCCTTTTCCCCACTCCTTAATCTCTCACTCACACTCTCACTTTGCAATCTATCCCTCTCTGCATAAACCACCATACAAACCAACCAAAAAAATAAAAAATCCTTCATTTATAATAATACCCTTTTAATTAACTCTCCTTTTACCATACCCAAAAAAAACTTTGCTTTTTCACTCAATCAATACCCATGTCTTATCAAGATTCATTTTTTTGAAAAAAGAAAAAG

>Ca2:162087-162302

CCCAAAAATATTCTACCTCTCACTAAATTCTCAAATCGCGACGCACTCTCATTTCCACCACTGTGCTCCACCGGCGCCTTCCTCCGGCCACCGCGAACACCACCAAGCTCCGATTACGACCATCAGACCGCTCTGATTTCTCCCTGTTTGGATTGTTCCCTCCGGCCTCCCCCTCATCCTTCTTCATCTGTTGCTGTGTTCTCTTCTTCCCATTG

>Ca2:167380-167838

GGGCTGCTAGTGCGCATACAGCAGCTGGTCCTAGAACTAGCACCATTGTTTGGACAAAACCAGTCCCAGGAAAATTTAAATGTAACATGGATGCCTCTTTTTCTTCGCAACACAATTTGTTGACCAAATTTACTGAAAGGAGAAGCAATTGAATTGCTAATGGTCATGCGGTGGGTATGTGATCTTCAACTACAAGGAGTTGGTTTTGAGTTAAATTCTAAATTGTTGTGAATAACACTACATTTAAATAGAAAAGGTGCATTTGAATTGGCAGCTATTTTATATGATTGTAGAACCTTACTTCAAATATGATTTTAGAAACTCTCTTGTCGAGTTTGTAAGGAGACAAGCTAATGAGGTAGTTCATGCTCTAGCAAGGGTAATCATATTCTTAGTTGGTCCCCATATTTTTTTATCAATATTCCTATATTTATTCAATCCAAAGTATTACTCTCTCT

>Ca2:162089-162302

CAAAAATATTCTACCTCTCACTAAATTCTCAAATCGCGACGCACTCTCATTTCCACCACTGTGCTCCACCGGCGCCTTCCTCCGGCCACCGCGAACACCACCAAGCTCCGATTACGACCATCAGACCGCTCTGATTTCTCCCTGTTTGGATTGTTCCCTCCGGCCTCCCCCTCATCCTTCTTCATCTGTTGCTGTGTTCTCTTCTTCCCATTG

>Ca2:162091-162302

AAAATATTCTACCTCTCACTAAATTCTCAAATCGCGACGCACTCTCATTTCCACCACTGTGCTCCACCGGCGCCTTCCTCCGGCCACCGCGAACACCACCAAGCTCCGATTACGACCATCAGACCGCTCTGATTTCTCCCTGTTTGGATTGTTCCCTCCGGCCTCCCCCTCATCCTTCTTCATCTGTTGCTGTGTTCTCTTCTTCCCATTG

>Ca2:167204-167838

GTCACAGTTGTGTATGTGTCAACCTCTCACATACTCAATGCCACCTCTCTTTTTTCATAATATAATTGTCGACATTTTAATCAAACAAAACCGAAATCATTCAATCTCAAACAACAAAGAATAATTTTTAACGGTTAGAGAACAAACCAATATCAGGCTGTGTGTGGATGAAACAGGGGCTGCTAGTGCGCATACAGCAGCTGGTCCTAGAACTAGCACCATTGTTTGGACAAAACCAGTCCCAGGAAAATTTAAATGTAACATGGATGCCTCTTTTTCTTCGCAACACAATTTGTTGACCAAATTTACTGAAAGGAGAAGCAATTGAATTGCTAATGGTCATGCGGTGGGTATGTGATCTTCAACTACAAGGAGTTGGTTTTGAGTTAAATTCTAAATTGTTGTGAATAACACTACATTTAAATAGAAAAGGTGCATTTGAATTGGCAGCTATTTTATATGATTGTAGAACCTTACTTCAAATATGATTTTAGAAACTCTCTTGTCGAGTTTGTAAGGAGACAAGCTAATGAGGTAGTTCATGCTCTAGCAAGGGTAATCATATTCTTAGTTGGTCCCCATATTTTTTTATCAATATTCCTATATTTATTCAATCCAAAGTATTACTCTCTCT

>Ca2:192436-193019

CTAACAGATATGAAGAGTTCTAATGAATATGTTTCCAAATTTGAAGTGAATAGAGAAAATGTGTTTAGTGACCCTCAAAGGCTTCCGCTAATCTGCGTTTCAAATCGAATTCTAACATATTCGAAGAAATATAGAATAGCGGTTGACCTGAGAGGGTTCACTGAAGACTTTATTCTTTGTATTTTGATTATTCAGTTAATATAAGAGATATTAAATTTATCTATCGACAATAAAAGTATTCGGTTTAATAATCCTAAAAAATTTCAGGTCATTATGTGCTTTTGTACTTGATCTTTACTATTAGTTTGTTTTATATTTTTGATGAATAGAAATCATACTTAATTAGTATATCTTATTTTACGTACTAATACACATTGTCTACTAGCCACTTTTGCTACACCGAGTCACAACTAGTTTTTATTTAAATCGATGTGATTGATTTTTTTTAATCAACTCTTATAACTCAACATTGTATTTTGAAGTGGATTCTTCAGCTCAGAAGACTCAAGCATTTACAAGAAAGCAACATCACTAGGTGTGTGTATTCAAATATAAACCTTAATTAAAGATGACTCAGAGTGTG

>Ca2:1689822-1690418

CTCAATGCCTTTTTTCAATGTTTGCTTGAACTTTCTTTTGGTGATCCAAGGTCGACTGATAACTCTTACAAATAAGAGTTATCTTACTTCTTTTCTAAAAATATTATTTAGTAATTGGGTCTTCATAATATTAGGATTTAGTAGCTTTTAAGTCAAGCACAGTTTTGTGCTTTAAAGAATATATATTATCCCTTGAGTCTTGCCTCTTTTAAGGTTACAATTATGGTTGGCCTAGAAAGAAAAATGGCAGAGTATTATAAATAGATTTTGATGTTCTAACCATGTCTCAGATCTGATTTGGCTGCACCTCCATTACTGCATTTAATATTAATTCTCATCCCTTCCATTTTCATTCATTGAAGATTGGAGCGAGGTGTTGTATATCTGGCTGGAGCAAATAGTGGTTGACAAATCTTTAGTCATCTGTTAGAGACTCACTTAGATTATCTATTTATTTCTCACTTGTGGTTGTAATTGACTTATAACTAACTTCCTTGTCTTTGTTTATTGTCTTGTTCACTATTGGGTAAAACAATTTGTTAGATTATGTTGAATTTTATTATGAACTGGTATTAATCACGTGGTGTGTAATGGTG

>Ca2:1812789-1812993

CAGAATAGTACATGATGGTTTTTGGAGAGACAGAAGCATTGATGAACAGATCATGTGATGGTTTTGGGAGGCAGACAAATCATCTTTCATCATATCTGTTGAGCCAGCAACAACTAGGATTCATGCCCTTGATTGTATGAGTATCCTTGTGAAATGTAAAAATAAAATTATTTACCTATAAAAAATTAAATATTAAAAGTAAAC

>Ca2:1812792-1812993

AATAGTACATGATGGTTTTTGGAGAGACAGAAGCATTGATGAACAGATCATGTGATGGTTTTGGGAGGCAGACAAATCATCTTTCATCATATCTGTTGAGCCAGCAACAACTAGGATTCATGCCCTTGATTGTATGAGTATCCTTGTGAAATGTAAAAATAAAATTATTTACCTATAAAAAATTAAATATTAAAAGTAAAC

>Ca2:2066856-2067065

CTATTCTCTATTACTAAAATATTATTTATTTTAAATTTGAATAATAATATTAAAAATATACCATCTTTATAACCAAAGAATCACATTCTTCTTCATCTTTTTTCTTCTACTATCATATTCTCACTTCAATGCTTTCAACAATAGTTTCAAATTTAACATCCTAAATAAAAAATTATTCTCCAGCTACTACACTATTTTTCATCCTTCAG

>Ca2:2596937-2597245

ATCAGAACTTCTATGGTAAGGAAAAGAATATTGTTTTCACTTTGATCATGGATAAGAGGCAACAATCTAGAGAGACACACGATCATGCACTCAGGTGATCTCAATCGTTCGGGTATAATAATTTTTAGATTTAATATGTGTACATTGAAATTGTGTTTATTTAACTCTTTATATCTCAAACTATAGTTTTAGGATATGTTTGTGTTGGCATTGATTCTTGTTATATAAGTGATAATGTTATGAGATTTCATTTGACATGTTTGGTTTTGATGCATATTAACTATTTTAGTAGTATAATTCATAGAGAG

>Ca2:3817901-3818236

TTTGTTTTTCATTTATTAAGACTTTATTATTATTTATGAAAAATTGAAAATAAATAAGCTAAAAGTTCAACTCTCTCTCTCTCTCGATTTTTGTAACAAACAAATGCAGTGAAGAGACACACCCCACACCGAAACCAGAAATAATCAAAACAACACTTCCTTGCTTCCACGTTCTCTTTCACTGGTCCCTTCTTCTCCTCCCTTTTCTTTTTCTTCTTCTTCTTCTTCTCATTGAGCCAACACTATTCACCTTCTTACACACTCTCTTTCCTTTTTTGTTTCCTTTGCTTTCTGATCAAGATTCGTCCTTTCTCTCTCTCTTTTCTGAAAACAAG

>Ca2:3829707-3829982

ATTGGCATCTTATCCTCACAAGAAGTTTGGTTGAATTGCTCATGGTTATATTTCTTCCTAGGGACAACTCAGAAGTACCATTGTGATTTTATTCAAACCATTATGCCTGATGTTGCTATCTCAACACATGCTAGCATTTTCTTCAAAAGGGCAAAATTTTACCAACAATTTTTGTTAATGAATCAGATACAAGGATGTTATTGTGAACTATGGTTATATGATGTATATTGATGTCTTGATATCTGAACCTGAGTGAGTGATCCATTTGGTAGTGC

>Ca2:4755600-4755940

GCATTTTTAAAGAAGTCACCTGTTAACAACAACAAAAAAGAGGTACCAACAAAAAATTCCCCCAATTTTTATTTGGTGAATTAATTCATTGCCGCCTACAGAATCCCTAAGTTTAAGTTAGAGCCAAAATTCTTAAATTACTTATTTTATCATTGGAATGCCTAGATCTTCTTAGTTCCGCAACATCGACGCCGTCGAATAGCAGCAACGACAACAACCTCGACGGCGGCAACAACCACTACCACGATGACATCGCTATTCCTTGGCTACAACAACAGCTGTGACGACATCGCCTCCTCTTTCACAATCTTTTTCACACCACCCCATTATTTCTCTACCG

>Ca2:4758313-4758590

GTTATTAATGAAGAAAGAATAACATATATTATTCACATGGCTTAGATAACCAAAAGCTTTGTAGAAATGGAGAGCAACACGTGTACAAATGAAGAACAATGAAACTATGGAAGACCCATGTAAAAAGAAGTTTTTAAATGATTAAGATTGTAATTTTCATACATCTACTTGGATTTGAGTTGTATCTTGTTTGATTATTTTGAAGCTAATTCTTGATGTGGTACACATTACTTGAATCTCTTATGTGGTGTATTAATTTTATGATAAAGAAAATTCC

>Ca2:4755611-4755940

GAAGTCACCTGTTAACAACAACAAAAAAGAGGTACCAACAAAAAATTCCCCCAATTTTTATTTGGTGAATTAATTCATTGCCGCCTACAGAATCCCTAAGTTTAAGTTAGAGCCAAAATTCTTAAATTACTTATTTTATCATTGGAATGCCTAGATCTTCTTAGTTCCGCAACATCGACGCCGTCGAATAGCAGCAACGACAACAACCTCGACGGCGGCAACAACCACTACCACGATGACATCGCTATTCCTTGGCTACAACAACAGCTGTGACGACATCGCCTCCTCTTTCACAATCTTTTTCACACCACCCCATTATTTCTCTACCG

>Ca2:4755626-4755940

CAACAACAAAAAAGAGGTACCAACAAAAAATTCCCCCAATTTTTATTTGGTGAATTAATTCATTGCCGCCTACAGAATCCCTAAGTTTAAGTTAGAGCCAAAATTCTTAAATTACTTATTTTATCATTGGAATGCCTAGATCTTCTTAGTTCCGCAACATCGACGCCGTCGAATAGCAGCAACGACAACAACCTCGACGGCGGCAACAACCACTACCACGATGACATCGCTATTCCTTGGCTACAACAACAGCTGTGACGACATCGCCTCCTCTTTCACAATCTTTTTCACACCACCCCATTATTTCTCTACCG

>Ca2:4755632-4755940

CAAAAAAGAGGTACCAACAAAAAATTCCCCCAATTTTTATTTGGTGAATTAATTCATTGCCGCCTACAGAATCCCTAAGTTTAAGTTAGAGCCAAAATTCTTAAATTACTTATTTTATCATTGGAATGCCTAGATCTTCTTAGTTCCGCAACATCGACGCCGTCGAATAGCAGCAACGACAACAACCTCGACGGCGGCAACAACCACTACCACGATGACATCGCTATTCCTTGGCTACAACAACAGCTGTGACGACATCGCCTCCTCTTTCACAATCTTTTTCACACCACCCCATTATTTCTCTACCG

>Ca2:5165362-5165698

ATCGTGAAAAATAACGGTGTTCAAATTCTGCTACACAAGTGCTATAGCTCAACTATAACTTATATTTGACAAAATTTTGTACTAAATAGTGTAGTGTGGAAAAATAGCGATTTATTCAAATTTCATTTTGCTATGCTTTAACGCCACTATAACTATTATTTAATAACACTGGTACAAGGAGAAAAAGCAATAGGATATGGAAATCATCTAAACTATGTTGTTGTTATTGATCTGAAAAGATTATATTAATATTTTGTAACCTTTAATTTTCATTGAAAGTTGGTATCCTAGCTTTTGAGTTCAGAAGAATCCATCAAATAATGTATTAAAGTTTAC

>Ca2:5513000-5513380

TAGATCTAGTTATAGCTCAAGGTTAATATGTCTATTTCCAAAGATGCTTCAGATCTCAGATCCACCACAAATCTTCGACTTCTTATGTAAATTCGGCCACCAACATGATTATTATGCAAATAACTCTCTTTTTACCCCAAATTCAGCCAGCCGGGGCACTCAGGTATGTATTCTTTTAGGGAAATGTATTTTTTTAGAGAAATGTAACAAGTGTTGTCTTGATTTTACAAGAATGACTCTCCCTAACATTTTGAAATTGTGACATTACTCTTTGATTAGTTAAAAAAAACTATATACTACACATGTTTAGTGACATTACTCTTTGTAGTGGTTGTATGAAAGAGATAATCTCATTATAGTATATAGTTTTTCTAACCATC

>Ca2:5948239-5948459

ATTGTACATTTCACAATACAAACAACTTCCTTAGGATTCTATATAAACATTGTATACACATAGCTTCTCTTATACCATCACTATCCTCCTTTGCTCTTATCTTATCTACCTTTTATTTTATTTTATTTTTTCTTCAACATGTTCAACAAATTACCCCTGCTTTGCCTCATATTATTTATGGTTTTCTCATGTGTTGCATCTTCTTCTATCCAAACTTCAA

>Ca2:6931451-6932001

CAATCCATTTTGCTATTTGCACCCTCCTTTTCCAACCACAATCACCGCCTCACTTCAATCCATTTTGCTATTTGCACCCTCCTTTTCCAACCACAATCACCGCCTCACTTCACTACTACTACTACTACTACTACTCCTATTTCTTCTTCTTCTTCTCGATCGCTGAGCAAAGGAACAATACATATAGCTACAACGATTCTTCAAAGGGAGTGAAATCCAAAATCAGAGAGAAAGAGATAGCGACTTTGTTTTTATTTATTTATTCATTTATTTATTTATTTATCTTCTTTTTCTATAATTTTCATTTTGAGAAAAAATAGAAAGTGCTGAATCTGTTTCTGTTTTTGGGATCCTTCTTGGCTTTGGATTCAACGGACTTCTCAGATTTGCCAATTAGACTGAAATTAGGGTTTCTGTTTGTAGGTATTCTTCTTTCTCCTTCCATTATAGGATCTTGTCTTCTTTTTTATTTTTATTTTTTATGTGCGAATTATAAATATCTTTTGTACGAATCAGCGACGAAAAACACGTGGCAGTAGATATTTTCACA

>Ca2:8153512-8154326

TGAAAAAAAGATTATAACATAACATAACACCACTTCTTATTCTTCTTCATATACTTCTCAACCCATCAAGTATTTAAAACTATGTTCATACACACCAAATTCTTCACTATCTTTCATAGACACCAAAGTTTGTTTTAATATAACAAAACTTTGAATCTTTGATTAAGCAATGAAGCTTTTTTGGTGGCAAAAATCAATCCATGGTTCTTAAAACAAGCCTGGAGAGAAGTGTGAGAAAGGGTATATATATTAAATTACTAATTTAACCCTTTTCATACTTTCCCTCCTCTTCTACAACAGTTTGCTTTTGTGCTTTAGCTATTGGCTTCTCATATCACAGAGGGATTATGTTTTCATCAAACGTGCTTTTTCTGAGTAGCAGAAGCAATTAAATAAATTAAGCATTTTTCTCTAATGGATCAGAAGCTTTTTGGTTATGGTATGGCAAACCGGCAAGTCACAAAAAGGCAATGGATTCCAATTTTTTTGGGGTCTTTATGGCTATGTATCAATTAATCAATTACTCTATTATATTATTATTATTATTATTATATTTATCTTATTGCAGTTTAGATTAATGTAATAATAATTAACATGAAGATGGTATTTTTTTCTTTTGAGAAGTTGCCAACTTTGTGTTATGTTCAAATCTCACTCAATTTGCAGCTGAATAGAGTTTTGTTTCAGTTTCTGCAGATGAGTTTAAGGTAGGTAAGTACTGTTATTATTCCTAGTTCTTTTTCTTCTGTGTTCTTTGTTTGTTTGTTTGTGTTATGACTTATGACTTTATGAGAAATAAAAAAAATGGAGACTT

>Ca2:8375221-8375759

TGAAAACACCCCAAGTAAACAAAAGCTGCACATTCCTAGGGCCGTGAGAGTGTTATGTGGGTGTTGATGCTCCAAATCTTACTTTCTCTTCTTACAAAGCAATCACAAGAGTAAAGAACACAAAGAATATCTTTAATTTCCTTCTTTATTAAAGCAAGTTCCCCTCCTCCCCCTTTTTTAAATTCAAAAAATATTCCTATTTATTTACATATCCTCTTCTCACACACCAAATATACTTGAGTAATTGAGTTAGTCTAAATTTACCTTAATCTACACTCAAAGAACAAGTACTTCAACCTCAAATATCAAATCCAAATCCAAATTTCCACAATTCTCCTAATTCAATTCCTCTCAACAACAAAATTTTCACATTCTCTTATCTAACTCTTAAGATGTCTTTACAAGAACAACCAATGCAGCAACAACAACAACCACAACCACAACAACAACAACAGCAACCAGTTCAAGTGTATCCAACCACTGTCACATACCAATCACCACCTGAACCTGACCACAATTCTTCAAATGGTTCATTTGG

>Ca2:8375431-8375759

ATCCTCTTCTCACACACCAAATATACTTGAGTAATTGAGTTAGTCTAAATTTACCTTAATCTACACTCAAAGAACAAGTACTTCAACCTCAAATATCAAATCCAAATCCAAATTTCCACAATTCTCCTAATTCAATTCCTCTCAACAACAAAATTTTCACATTCTCTTATCTAACTCTTAAGATGTCTTTACAAGAACAACCAATGCAGCAACAACAACAACCACAACCACAACAACAACAACAGCAACCAGTTCAAGTGTATCCAACCACTGTCACATACCAATCACCACCTGAACCTGACCACAATTCTTCAAATGGTTCATTTGG

>Ca2:8762375-8762815

GAAACACAAACACACACCATACAATTGGGGAAAGGCTATTGCAGAGGAGAAAAAATAAATAAAAGGGGTAGTGAGTTTGAGAGAGAGGTAACGAACAATAGAGAAAAATATATTGGAGTAAAGGGAGGTGAAGATATGGTGCATAAAGAATGAAAGATTGTGATAGTGGCGGAACTCGTCGTAGATCTGCATTGGATTTGTTGGCAATATAGCGAACTCTTTGGTGCTTGCAGTGGCTGACGATGGTCGGATTCAACGTAGGTGCACATGGTCGATAAGAGACGATGGTGAATGGAGGTTGTGTCGGCGAGGAACAGGAAGAGAGAGGTAAGAAGAAAGTAAGAGATAAAAAATAAGTTTGATATTTGATCTTGTATATTTATATTTTTTGTAGAAAAAAAATTGAATGTTGTTTAAAAAGACAATTTTACATATTATTG

>Ca2:10274738-10275560

GAGAGCTGGAAATTCTGGTAGACCACCTGGTCAAAGTTAGTCAACAAACATGCAGAAGATGCAAAAGGGCTAATGAAAGTGATGATTTCATTTATATCCCTAAGCTTCTTAGGATTTCATGCTTGTATGCAAAGGCTAATCACACTTATGGAAATTTGATTTGTTAAGGGAAANNNNNNNNNNAAAAGAAGGCTTGTCCCAAAAGAAAATAAGTTCAAAAGAATCAAAAGTTTGTTTCATGAAAAACTGAAATTTTATTGAGTGTTACATTTTTTTGAACTTATTTTGTGAAGATGGGGTTGGAGCTTGAGAGAAAAGAAAAAAAAAGAGTCCCAAATTTCAATGGAATTGAAATCAGTGCTTTTATGTAAAGCAATTAACACAACTTTAAAGCATGAGAGATTTATCATTTATGTACAAAATGTTGTAGCTATAGCATTAGCCATGCATGTATTTTTGAGGACTATGTTAGGGTGAAAAAAAAAGAAATTTTTGGCTTTGGTTTGCTCAATATAAAATTTTGGAAATTCTTTTTTTTTTTTGAATTATTTATTGGAAAGAATAGATGTGAGTTCTTAAAGTAAAAGTAAAAATTCCACCATTTAAAGGTGTTGTTGGGGGAACATGTAAAAAAGAAGGGTGTTTGCATCTTGTGTTGGTCTTTTTCACTAGATTCTTTTGTGGGGGTGGTGAAACTGGCATGTTGGTCTTTGGTCCATTCAAATATTTTAAAGATGTATTTTTATTCTAATTATTATAAGTTGTAAGAAAAAAGAAAATGAGATTTTATATAGTGCAAATGATAAAGGGGGAAACAAACAT

>Ca2:10456515-10457058

GGGAGAGAGGAGAAGGAGTAGGAGGAAGGAGAGGAAGGAGAGAAAGGGAATGGTTAATGGTTTGGGTTGTTGTGGAGACATGGTGATGAATGAGGAGAGAAAGAGGGTTGGGGAGTCATTAATTGGTTAGATTAAGATGGGATCTAGTTTTGATGTTGAAATTGAAATTGAAATGGAAGGAAGGTGGTGAGTAGGATAGTGGGAGGAATAGGGATAGCTACTTGGTAGTAGTAGAGAGTGGAGATGAGAGAGAGAGTAAAAAGGTTCTTGGAGATGGGAGAAAAGAGTGAAAAGAGAAAGACAGCTAAAAAAAGGGTCACGTGCAGGACAGAAAAAACAAGTAGGTTACCCAAATAGAGAGACAACTCTAAAACCATAAAGAATTCCTCGGCAAAAAAAAAAATTTGAGTTTGATGACCGCATACTCTGTATAAATAGTTTTATTATGATATCCAATTAAATTCAATTACGTGGAAATTTTTGGTATATATTTCACTTTTTATTTGTTTTAAATGTTTTATTCTATAATTATTATATTAAAAT

>Ca2:11216516-11217078

GGAAGAGTTGGAAATCTATCAAAAAGAAGATGCAGAAAGAGAAAGAAGGAAACGTATATAGCAAAGGAAAGAGAAAAAAAATAAAAGAGAGAGAGAAAGGAGAGAATCATGAGCGAGAGTGAGAAAGCAAACATGAGAAAGGGAGGGTGATTCACTAAAATCTGTGATGGAGTGACGAAGAAGAAACGACAGTGCACAATCGTGTTGAAAATACAATCTATTAGAGATGGTGGTGAAGGTTACGGTTCCTCTTCTTTCTTTATCTTTTTCTTTGTTCTGTTTTTTTGTTGTTGAATATTCTAATCGAAAGAGTTTTTTCGATGAACTACGAAAATGACTTTGATTTTTATGTTTTATTTTTTTGGGTTTTTGTTTTCATGGCGGAATATATTTGTGGATGTATTGAAAAAAAATAAAATCTATTGGGTTTCTAAATTTTTCCAAAAAAATCCTCTTCTTATTTCTTATCAGCCCCAAATTTTTTCTACTTTTTTTATTGATTTTTTGTCTCAATTTATAGGTTAAAATGATGAATCTTTGTGTCTATGTAATAAAATTGAGC

>Ca2:11484673-11485499

AAATAAATTAACTTCTGTGGCTGTGACTTGTTGCTTTCAAATTTGCATGTTCTTTCCTTAATCTTCAATGACAAACCAGCTAGCAAGGCTTTGAGATAATGTATCACTTTATAGGTAAAAAAGAAGAAGAAATTATGTTTGTTCAAATCATGGTGATGCAGGTAGAATGTATTTGAACTTTGAAGGAGAGGGACCACTTGAGCTAGAGTAACTGACAAACACTGTTCAAAGCTGAAGTATGTATAATTATGCAGTTGATGTTACTGGAAAATGCTAAAGAAAGATCGAGTTTTGATTTAGATGGCTTATTCTTCCACTCTCAACGCTCTCTCCATTAACGGTCAATTCAGATGTGTTTTTTGTGTGTGTCAAAATACACTTTTGGATTGACCGATTCAGACGTGTTAATTAGAGGTTCTTGAGAACACAAGGGACTGAAAAAGTAAGCTACTTTGATTATTCCCTTATGGAATATTGAGCTTGCTAATCCATTTTTTTGATCCCAAATATGAAGGACATAAACACACTAAAAAACCTTGTACGTTCGTTCGAATTTTGGTGCTTACTCGACTTAGTACATTTGTTCAACTCGTTAGTTATTTGCTTGTTTGCTTCATTATTTGCTCAGTTGATAAGATAATACATTAGTTAACTTGCATTTCAAACAATGATATTTCATTTTTGAGATGATTATGTTGAGATAGGAGACTTTGTTTCTCATCTAAAGATTCCTAGACTATTCTAAAAGAACTTTGTTTTTGCAAAGACAATTTCAAAGGATGTTTAATTTAGAAGGTTGAATGACTATCAAAATAACACACCCTCT

>Ca2:11484834-11485499

GTAGAATGTATTTGAACTTTGAAGGAGAGGGACCACTTGAGCTAGAGTAACTGACAAACACTGTTCAAAGCTGAAGTATGTATAATTATGCAGTTGATGTTACTGGAAAATGCTAAAGAAAGATCGAGTTTTGATTTAGATGGCTTATTCTTCCACTCTCAACGCTCTCTCCATTAACGGTCAATTCAGATGTGTTTTTTGTGTGTGTCAAAATACACTTTTGGATTGACCGATTCAGACGTGTTAATTAGAGGTTCTTGAGAACACAAGGGACTGAAAAAGTAAGCTACTTTGATTATTCCCTTATGGAATATTGAGCTTGCTAATCCATTTTTTTGATCCCAAATATGAAGGACATAAACACACTAAAAAACCTTGTACGTTCGTTCGAATTTTGGTGCTTACTCGACTTAGTACATTTGTTCAACTCGTTAGTTATTTGCTTGTTTGCTTCATTATTTGCTCAGTTGATAAGATAATACATTAGTTAACTTGCATTTCAAACAATGATATTTCATTTTTGAGATGATTATGTTGAGATAGGAGACTTTGTTTCTCATCTAAAGATTCCTAGACTATTCTAAAAGAACTTTGTTTTTGCAAAGACAATTTCAAAGGATGTTTAATTTAGAAGGTTGAATGACTATCAAAATAACACACCCTCT

>Ca2:11776828-11777104

AATTAGAACAACGAAAGCTTCCATCTGATTTGCAGAGAGTTTTTCTCAAAGACACAATCAAGGACACTGTTTAGAGAGAGAAAGAGAAAGTGATTCTTTCTGTGAGTAACATGCAATAGCTATAGATATAGTTATTCTTTATATATTATATACAAAAAATGAGAGAGAGAAAAAAAGAAAGAGTTGAAGATTGAAGTTCAATTTTTAGAATTGTTCATCAATTACCCTTTTTCTGTTTTTGATTTTTTTGGTTGGTTTTGGTTCCTATTCAACCCT

>Ca2:12257226-12257753

TTTGGAACTGTCCAAAGCATAAATTCCCCACTTTATCACAACTGGGTGACATATTTTTCTTAAGATGAAGGGTGACTGGTCATAGTCCAAGTGAAACAAACAACCAACGCATGAACAATTTATGTTCTCTCTCCTATGTTTTTTGATTTTTATACTTTTTTATCATCCAATTAAAGTGACAGTTTATGTGACTCGTTGCTTTCCATTTTTATTTTTTCTTCCTAACGTTTGAAACTTCCTTTTGAGTGTCACCTTGTTGCAAACCATATGCCCTTAAAGAATATGCTTCTTTATTTTCCCTCTGATTAGTTGTTAATTTACCATTGTATCGTAACATGTGACATTCTAATGTGGAATGCATTGTTACTAGATTGTTTGTTACATAACCTTGCTTTGATTGGCACAAGCACATATGACAATGGGGCGGGGCGGGTATGTACGAGTAGTGTTATCGCTTGTCCCTTATTCGTACAAATATTTGTTATGCAAGGTTTTGGTAAATTTGTCGAATACATTGGTATCCATAG

>Ca2:12252796-12253520

GGTGATGGTACATGCAACATTGTTGTCAAATAGCACGGTGGTATTCCCCTGTAGTAGTGTAGTGGAATTTGAACCAAATGCTATTGTTATGTGAAACACTACTGTACATAGTGTTGTAAAATAAGGGCTATAGTGGTTTTATAGCGTTGTAGAGTAAACAAACCACTATTTTATGCTATTAGCTATCGACAACACTGACATGCAGTTATGAAGGAGTAGTCTAGATATCTACTTGTAAATAACTGCCCTTATTCGACATCTTTGGAAAATTAGATATATCAACTAAATGGTATTGTACAGACAAGTAACTCTTTTGGAAAATTAGATATAACAACTAAATGGTATTGTACATGGACTCTGTTTGGCATAAGCTATTTCTATAACAAAAGATAAAATAACGTTAAAACTGTTTTTGTTCAAGTTATAAACTGTTTTTATAAACTATCCTAGAGAGTCTATGAAATTAGCTGAAAAAAACTTATAAACATGTGATAAGTTGATTCCACAAACTCTCAAAAGTTTCACAAGTGCTTATGCAATTATGTCAGTAGATAAATTCAAATAGGTCAAATCAAACTGGTTGATGTTGTATCAAATGGGATTTTGTTGTTTTGGTTTACACATTAATTGTCAGATCTTCTGTAGTACCATGAATTGGTGAAAAATATTACTGCTATAGAAATTTAGGGTGGACCGTTCTTCCTAGTCTTTATTTGATATAAGG

>Ca2:12255349-12255707

GCCTGATACATGCTTTGGTTGAACAGATATTAAGAATCCAACAAAGAGAAGAAGAATTATTCTAGAAGGATGGAACACCATATTCACTTCTCTCTAAAACAAGAATAAAAGGTATGTTATTTCTGTTTAGAGTGACACAAGACAAGAGAGTTTGCAGCTATCTGCAAGTTCTATCTGTGTTCTTATGGTCCATTCCTGTAGATATAAGCTTGTTATATATGTCCCACTAGATTTGGTGGAAGCTTTAAAGTTTAAAGCAGGGAGGGCCTACGGGAATTTTAGATTCACCTCGAATCAGAAGCCTGAAATCTTAACTAATTAAGGCTGTTATTTAAGAATCTGACAAGAATGAATAAAG

>Ca2:12252511-12253520

ATTTGACCTAGAAAAAATGGAAATAATTATTGGCGAAATAGAAATAATGTTTTTCCATATTCACTTCTGGTTGTTGATACTGTAAACAATGATGGTTTGATTTCAACAATTTGATGATGTAGAGTAACTTTGTAGCTGGTAAGAATCATGGCTTAATGGTCTTGTAACGACTGTGGTTTTAACAATGTCATTTATCTTGCTTATTACTTTAAAGAGTTGGTTTTCTTCTTGTATTGTAAATTGTATTGTTAACTGATAGATTCTTTATATATGAATTGTTGGTAGGGTGATGGTACATGCAACATTGTTGTCAAATAGCACGGTGGTATTCCCCTGTAGTAGTGTAGTGGAATTTGAACCAAATGCTATTGTTATGTGAAACACTACTGTACATAGTGTTGTAAAATAAGGGCTATAGTGGTTTTATAGCGTTGTAGAGTAAACAAACCACTATTTTATGCTATTAGCTATCGACAACACTGACATGCAGTTATGAAGGAGTAGTCTAGATATCTACTTGTAAATAACTGCCCTTATTCGACATCTTTGGAAAATTAGATATATCAACTAAATGGTATTGTACAGACAAGTAACTCTTTTGGAAAATTAGATATAACAACTAAATGGTATTGTACATGGACTCTGTTTGGCATAAGCTATTTCTATAACAAAAGATAAAATAACGTTAAAACTGTTTTTGTTCAAGTTATAAACTGTTTTTATAAACTATCCTAGAGAGTCTATGAAATTAGCTGAAAAAAACTTATAAACATGTGATAAGTTGATTCCACAAACTCTCAAAAGTTTCACAAGTGCTTATGCAATTATGTCAGTAGATAAATTCAAATAGGTCAAATCAAACTGGTTGATGTTGTATCAAATGGGATTTTGTTGTTTTGGTTTACACATTAATTGTCAGATCTTCTGTAGTACCATGAATTGGTGAAAAATATTACTGCTATAGAAATTTAGGGTGGACCGTTCTTCCTAGTCTTTATTTGATATAAGG

>Ca2:12253691-12255278

ATTTTTTGTTTTCATTTCATAGAATTTGTTTTAATTTTACCGGTTGATAAGAAAGTAAGAGATTTTATGAAGTGAACAATTGTCTATATTTTTTAAAACAATGAAAATATCTAAAAAGTATTTTCATTATTCTCGGAAATGTATCTTCATTAACTAGTATTTCATTTTCTCTCCAGGACAATGTTTTACTTAAATTGTTTTTCATCAAGATAAAATGAACACACATTTTAGAAAATGGAAATATAAAATATTTTCACAAAGACACAAACTATAAGGGATCTTAGTGTTTCTCAATTATGAAAAGGATATTAACTTTGGTTTTTCTGTTCCCACTATTTCCAGCATCGTACAATACATTCAAAGGATGTTGAAGCAACACTTCCTTGAGTTTGCATCACATATTCTATTATTATTATAGCTGTCAAAATATGTTTCTGTTTAATATCCCTCAAGTATATGGATTCAGTTATATTTTAATTCTTGACAATAGCTTGTATATGACTTTGATTGATATCCATTATAGGAGTGAATTTGTGTTGGCTGACATGCTAGTTTTCCATTTTGATTTTAAGAACACCAAAATATTTTGAAAATGTTCATTATATTTTGTTGATAAAAGTAATGCCTTGCTTTTGGTAAATTGGTAAGTTGCATATCCTGTCTCATGCATGTGAGTCTAGCATGCTCTTTATCGTACTAGTATGAACTAACTTATTTTGTTTTGTTAACAATACTCTTATTATTTGTTCTGTTTTCATGGGCAAACCATATTTGCTTTTCACCAAAACATGCACACCCGAATCACAAAAACAGAAGAAATAAGAAGAGAAAGACTGTAAAGACAAATTCCTAATTTTCTATAAAATACTGTTTTACATGATGAGACAGTACAGGTGTTCAGAATTCTGATAAGAATATATAATACTCAACATCTTTACTTTTGTTGGTTAAGCAATAAGACTTGTCTGTAGGGTCCAGAGATTGAAGATACGAAAGTTCGTTAGATTCGAATACTTCTGAAACATGACATTACAAAAGAATTTCACTATAATCTCGCCGAAACTGAATACTAGTGCAAATGAACATTTTCTGTTTGAGGAGCTGGGATCATCATCTATACTGTCATTTTAGATGATGCTGGTTCATTGAATTGCTTTCCAAAATTTATGTAGTAGCCTGACTGTACAAAATACATGCCCGATGTCTCTCTATCACAAAACTCTTAAAACATAGATTGAAACTAGAGAAAAAACCAACCAACTGTTGTGAAGAACTTTTGAGAATGCAGGTACAACTCACTATGTTTCTAACCTGCTCTGCATTTCCAAGCATGTCAACTTCTGTTTCTCACACTTCTGGATGTCAGCGACTTAGCTTAGTTACGATTTCTCTCCAAGTCAAGCGAACAGTATATGTTAAGTCCTGTTGTGAATAGGATCTGATCCTCTTCGCACTCTTCTTTTGCTCGAATAATTTGGATCAAGCCTGTTGTTAAAATTTGCTGAACTCTTCTTTGTGGGATCAGACACATTGTTGACATTTGCTGTATTCTTCTTTGTGTTCAACATTTCCTTTGTAGCTTTCAGA

>Ca2:12742423-12742731

GCTCTCCAGACTCTATTTGGTTATGAACCATATCCAGGATTTCATTCAATTGTGCAAGCAAAAGTTGAAGCTAATATCGAGGAATGGAAAGAAAGTAAGCTGAGACTAATTTCATAGCTTCTGCTTGGATTTGTGTATCTTTAATAATTGTTTGATGTTAAATGATCTCACATCAACAAAGGAATAATAAAAAGCTACAGATGATCATGTATATATTACAAAGGTATTCATGCTTTTTCAGGATAATTAATTCCTCATCTGTTTTCATAGAAATTATAATTTATTAGCCATACATCTTTGTGAAGAAA

>Ca2:12933542-12934042

CCTTAACTAACAAAAACTTCCATTTCATTTCACCAACCAACCGATTGCACCCTTTTTTCCACCAACAAATCCAACCTTATCTCAAAACATTTAAAAAACATCAACACCGTACCGTTACTGTTCATCGTCTCTCTCGTCCATCAATTTTTTTCTTTTTTCCAAAATTCTCTAAAGTCTTCTACACATGTCTAACCCATTGCATGTCCCTATCTTCATCTCAAGATCCATTAGCTGACCACACTAACGCTAAAAGAAGATAACAAGCAACATTTGGGAGATCTATAAATTCGAACCAGAGTATAAAGGGAAGAAGAGCTAAGAGCTGAGAAAGCAAGCAAATCCCAAAACCAATTCGAAGTATAGGAATCCCTTCTTGGCAAATCGTGAGTTCTTTTACATTAATGATTGACATACCTTGTATTTGGTGTTACAACATGATTATTAAAAAATTGACCTTGTTAATGAAATCAAATTCATATTATTAATAATAATAATTCTTG

>Ca2:13420312-13420659

GATAAGTTAAATTTGTCTATGTCATAGATGAAGAATAAAGTACATTTTGTGAAAAGGGATGTTAAAACTAGAGATGGAGTGAGAGTTTATAGAAGGTACCCTCAAAGCCATGTGATTTCTTTCCTCTAAGCGTAACTCATCTCATCATGTTCTGCATTCATCTGTCTATTACCAAGTTTTGATTAATGGTCAGCCTAGCAAATCTTATGTCTCGAAGGATCGACTCGCCAAGGTGACCCACTTTCTTCATATTTATTTTATGTTCTAATGTTTTGTCTAGCTTGCTGCATAAAGAAACTCAGAGTATGAACCTACATGGTATTCAAGTAGTAAGAAAGAAGCGTGCC

>Ca2:13747665-13748162

TTTGTTTTCTTTTATGATCAAATTTAATTGTATATTGAATTAAACTTTATTTTATTTTAATTTTTAGAATAATTTCTTTTTAGCCTTAAGTGTTGGTGAATTCAGTAATTTTCCATTTCCATGTTGGTTTTTGGTTTTCATGTTTCTGTGAGAGGGTATAGAGTGTGGTAGAAAGGAAGATTTGAATGTGAAAACAAGCATGGAAATGGAACTTTTTTGGGGCAAACTTTGAAGAGAATGTGGTTGGAGTTGGGGGGGTCAAGAGGACCCCATGAGTTTCATTAATATTATGGAGCCACCAAAGGACAAGGATGTATTTATTTATGGTTAAATGTCAAAATGGGTCCATCAATTCCCATTGGTACCATGTCGGTTATTTGTTTCAAATTTTAATTATTCATTTATAGTTGGTAATTGAATTAATTGATAGGACTTTGTATGTATTATTATTATTATTTTTTAGGAGATGTGCCACATTTATTATTCTTATCTCTACC

>Ca2:16630432-16630732

TCTACATTCCTACCTCCTCCTCTCCACAGCACGCGGCCATACAAGAAAGGAATTGTGCTCCCCAAACACGTTTTCTCCCAATCTCGCGCACGACCTTTAGTCTCCATTGCACCGTTTTCACAAATCAGCATCTCTGTCGTGGCAAGCTTTAAATCATATTTTTCATCTTTTTCTTCTATTTTTCGCTATAAAACTCCCTTCCCCTAATTCTTAACCTTCTTCCATTGGCTATGTTCTTATGTAACTTTTTTGTTGTGGGTCTTTTCCTCTTTTGTTGGTCGTGATTGTTTCAGGAGGTGA

>Ca2:16689329-16689743

CGAAGGCAAAGAACATGATGGCTAAAGTGGTCAAGTCTTAATTAACCATGGTTAATTCGCTTAACCTGACTCAGCGCATATGGTTAAAGCAAGAACTTTCCTAGCGCGAGCGAGAGCAAACATAAGAAAGAGAAACATAAAAAGAGGGCTTTGTCGTTCGTTCTAGTTGAATTCAATATTTTCTTTATTATTTTTGCTTTATACTAAAAATCCTTCTGAGTCGACTCGGACGAGTTGTCTCATCGTACGGACTCAGTCTTCTTTGTTAACCCTAACGATTCTTCTTTTTTGTTTGGTTTTTCCTACATCGTTTGTTTCTTCCTCGTTTCTTCAGATCTCGCATTTATTGCAGCGAGTTCTAATTCGAAGCTCAAGCTCAATCTACACCGTTCAATTTGATTACCTTGATTTAGG

>Ca2:16689400-16689743

CAGCGCATATGGTTAAAGCAAGAACTTTCCTAGCGCGAGCGAGAGCAAACATAAGAAAGAGAAACATAAAAAGAGGGCTTTGTCGTTCGTTCTAGTTGAATTCAATATTTTCTTTATTATTTTTGCTTTATACTAAAAATCCTTCTGAGTCGACTCGGACGAGTTGTCTCATCGTACGGACTCAGTCTTCTTTGTTAACCCTAACGATTCTTCTTTTTTGTTTGGTTTTTCCTACATCGTTTGTTTCTTCCTCGTTTCTTCAGATCTCGCATTTATTGCAGCGAGTTCTAATTCGAAGCTCAAGCTCAATCTACACCGTTCAATTTGATTACCTTGATTTAGG

>Ca2:17056827-17057711

TGTTAATCTTTTTGTTGGTTGAGGTTTATTAATATTTGAAGCATGACATATAAAATGAATATATTTAGAGGTATACGTATACTTTGCAATTTCTTCCAAATTTAGCTTAAAAGATTATTGGTAGAATTACTTGTTATATTGTGTTAGATAATGTTATTATATATTAGTAGTAAGGGTATTTTAGTCATTTCTATTCTTTTATATATATACTCATTGTAACCATATTAACTCAAGTTTGGTTCATTCAATGTTATGAAACCTTAGAGTGATTGTCTCTCTTCTTCCTCTTTTCATTGTTAACATGGTATCAAAGAGCTTTGGTTGATTTTGGGATCTACTATAAAGAAGAGAGAGACTATTTTGATTGGAAAGACAACCACCGAAAGAGAAAAGAGAAGAGTAACCCTATTCCCGATTTTGTTAAATCAGTCTCACAAAAACATTGATTGCGCATTGATTAACCGTTGGATCAGACTGATTTTTGGACAGCAGGTTCGCAACATTCAGGTCTTCGTCTTCAACGGTCGGATCGACGAAATGACGTCTGGAGGGGGAGAAATCGTGCTCGCACAACACCAGATTATTCCTAATTTATTTTGTCTCAGTGATTGTGTTTGTCGTATTTTCCTGTCATTATTTATTATGGCTGGTGCTAAGGATGATTCTCTTCAAGCACATTGAAAAGCACATTGTCCGAAATTGGGGAGAGCACATAAGAAGAATTTTAGGGGGACATCGTCCAATGTTGTTGCTTCTGCTCCTCATACCATTGACTCTAGTTCTAGTTCTGTATACTCATCTGAGACTGCTTTCCAAATATCTGATATTGCAGAACAACTTCAAAAACTTCTTGTCACTCAATCACATGCCATGTCTGCCACCTC

>Ca2:20015497-20015752

TATTGAAGATCGAATTTCAGTGTCAACAACAAAATAACTAATGAAGATCGAAGAAAGCTAGAATTATGGCAAATTCTCACCATTTCTTTTTTATTTGACTATATAATGTAGCATATATAGTGGAAATTCCACTTTTATTATTATTATTATTATTATTATTATTATTATTATTATTATTATTATTATTATTATTATTATTAAAAGCAATTTAAAAATATAAAATTGTTTCTATCATTGTATTGGATATAAAGTAGC

>Ca2:20466055-20466274

GGTGATTGAGTTTTCTTTTGTATGTTTTATGTATCTGTTTTTTCTTTATTTCTCACTGTGAATTGCCTCTTCTTTCAGTTGCAAGATCTGAGATCTACGGATCGATTACTTGTCGACTTTGTTCTGGGGGCATAAATATAATTTCAAACCAAAATCTTTTGGGAACTCGCATTTTTCCAGATTTACGTTTTTTGTTCTTCTGGATCCCATGTTTTGGTT

>Ca2:23033460-23033696

GTGGTAATAACTTCTTATATGCGGTGAAAGCTGAAATAACTTTCTATATAAGTCAAATTAAATATAGTTTGTGGTACCTCTAAAAAACAAATATAACTTGATGTCGCATCTGTAACATTTGTGAGTACAAGAACATGCATGGTGTTTACTTGTTAACAATTTGTGTAGGTTAACAATGATTATAATGTCATTTTATGAATTAAACAAAAATTTATTAAAGTGAGTAAAAATAAGAG

>Ca2:24739964-24740187

GCGAGTGTTTTGGTTTCTATAAATACTCGGACTCATTCTTCTTCATTCCTTGCTTATTTCAATTTTTTGTTAGCATAGAGATAGAGAGAGAGAGAGAAAGAGGAAGAGAGAGAGAGTTAAGTTTTTATTTATTTTTCTTCATTTGTTTGGTGTTTCTCTTTCCTCATTTTTGTAGGTATATTGTGGAGGAAGACTTTGAAGTTTGACTTTGTTGAATTAACAT

>Ca2:24743018-24743319

ACTGTGTCTTTGAGGTGACATTGAACAAATAAAGGGGTGCCGTCACCAAGAACATATATCATTGTTGTCACTACTACTCTGTGATATCTGCATATTGTGTTGAAGGATTGAAAGTAATAGGCTGTTTTTGAAAGTAAATTCATCTCTGAATTGCCTTGTTCATGGTATCTGCTAAATTTCATACTTTGTTACATTCTTGGCATATTTGTGAAGCTAGAAGATTGTAATATCAATTTTTCCTTCAACTTTGATGTATTGAAAGTAATAAGTATTCAATCAATGAAATCCAGAGTTGTTAATT

>Ca2:24739987-24740187

TACTCGGACTCATTCTTCTTCATTCCTTGCTTATTTCAATTTTTTGTTAGCATAGAGATAGAGAGAGAGAGAGAAAGAGGAAGAGAGAGAGAGTTAAGTTTTTATTTATTTTTCTTCATTTGTTTGGTGTTTCTCTTTCCTCATTTTTGTAGGTATATTGTGGAGGAAGACTTTGAAGTTTGACTTTGTTGAATTAACAT

>Ca2:25147931-25148212

GCACTTTGAAAATTTGAAAACCAGCAAGGCATACATGTGTGTGGAAGGTACTGATACTGGTATTATATTCAAAGCAAATAATTATTTTTGAAGCCATAATTATTCCTTCTAGGCCGATTAGTAATTATTATATGGTACAAAGTGAGATTCAAATTATAATAATTTTATGGTACAAAGTGAGATTCAAGAAGAGTAATGATATAATTGTAACCAAAAAGAGATTCTAGAGTGGCTTGATATGGTTGTAATACTTTGTCAAAAGAAAGAGTGATTAGATATGC

>Ca2:25697482-25698485

ATCAAAAGAGAATTATACCAGACTAAAGAGTAACTAAAGTATTTTGTAGTTGTACTGTAATTGTTTGGTTTTTTTTTCTTTTCAAAAATGTTTTAAGAAATAAGAAATCACACCCTTTAAATCTTATTTTTGTACAAAAATGTTGATGTCAGATCTTTTAATTTTTTTACGATACATTTATTTTGGAAATTTCAGATGATAAAAATTTAGATATATTGAATACTAGTTATTACAAAAATATATTTATAAAAAAATTACCTCTGAAAATTATAATTTTATAAAACTGATTTATTTACTATTTTGTTTGAAGAATTGCAGTTTTGTGATTTTTGAATATGCATTACTTGAAATGTTGTTTTATTACATGTTCTTTAATAAAATATTATATTTTAGTTATGCTTTTATGAAGATAATTGTAAAAAAATGAATTATTTTCATTGTATTAGTTTGAGATGAGTTAATGCTCTATATGAATGAATCTTGTACATGAATGTTGAGTCCATATAAACTTCACTCGATGAGTTTTGTACCCTACACTAGATGACTATGTGCTTTACATGATGAGTTAATACTCATTTTGTAAAAGTATAATAATGGACATCATTGAATTTATTGATTTAATTTTCGTAGAATAAACGTGTGTATTTGATTTTAAATGAGTGAATTTTAAACTCAATTGGTAGTGAGAATCGGTTCCGGTTATTGTGTCGGTTGAGAAAAATACTTCTCAAGTTGTTTGTTTCAATATACACTAGATGACTAGGTGCTTTACATGATCAGTAAATACTCACTTTGGAAAAGTATAATAATGGACATCATTGAATTTATTGATTTATTTTTCGTAAAATAAACATGTGTATTTGATTTTAAATGTGTGAATTTTAAACTCAATTGGTAGTGAGAATGATAAAGAAAAATTATTTAAATTTCTAAAGTACTTTTGGAAATATGTAGGTTGAGACAAAATCTCAAATTAATGTTTTGATGATAATTAAACAAAAAG

>Ca2:26036351-26036568

ATCGAAGTTTAATTAATTCTATCTAGGGTTTTGCCATTGGTAGATTGAATTGAATTTTCATTTTTCATTTTTTCTTTTACATGTTCTATGTGAGACTGAGTGAGAGAGATAGCGTGCGGTGTGGTGTATGGTGGTGTGGACCAGTTCGGACAAATGTGCTAATGCACTGGGGTCTGGAACCAGGGGCACGTTACCGCCTTGATGTCTGAGACACGAC

>Ca2:26726416-26726740

GGTAAATGACAACCTTAAAAATCACCACCATGGTGACATGGGTTTGAACCATTGATTATATACTTTTATACTCTAAGAGCTGAGATTTTAGCCGAATGAGGAGGCTTCACTGATTGCTGTATTAGGACAGTTGTAGGACAGTTGTAAAATACTAAAAGTTGTGTAACAATATGGGGCAATAATATGTTTATGACGATAAATAAAATTTTTATAGTTTGACATGAGTTTCGGTGCCCTTCTTTCCTTGTCTTTCAACTGCTGCTGAAGTCTTAGAAATTGGAGGTAGGGGAAATTAGAGAAAAATGAAACCATAGTTGAAGAGCT

>Ca2:27542423-27542642

CAGTAGGTCTCGGAAGAGTTTAGACAGTATGTCTTGCATCTGTTGGAGAAACCTACGCTCAGGCCAGCTTCCATGAAGCTGCTCTAAAGATACTGCAACCGACTCTACTTGAAAGATACCCCAAAAGATGTATGTCATTTGTTTTTAAAAAAGCCCTCTATTTATTGTTGATTTCATTCTTGATCTATATTTATATTATATATGCTATATATGATACGG

>Ca2:27800079-27800603

AGGGTTTTCATTTTTTATTTTTTATTTTTTTTGTTACAAGGAAGTAGTAAAAAATAAAGAAATACAACTAGAGTTTTTGTTTCTCTTATATTTTGTTATGAATTTGCAAAAGTCATCAAAATTCCCTAACTTGGAAAAGAAAAAGTTTATCTTTGCTTACCTTTGTTTTCATTTCTTTTGGAAAATAGTGCTTTTGCCAGATTTGTGTGCTTATTTATTAGATCTTCAATTCCTTTCCTTTCTATGACTCTATTGAAGACAGTGACACCTCTCTCTTCACTCATTTTTTCTTCTTTCTCCTTTCATTTCCTTTGCTTTCTTCTTTTATCATCTTGTTTATTTAATTTGCACTATGTTTATTATTTGATTTAATTAACTTACTCATTTTCTCTTTTTAGTTCATTTCTATTTATTTATACCCTTTTTGTCTTTACTATTGTGGTTCTCATTCTTTCAGTAGTATCATCCTTTTGTTGAAGCTTAATTTGGATTCATGATGGGATCTTTCACTTTATGAGAATATG

>Ca2:27801669-27801910

GTGGAGTCACCCCACATGCGATCAAAGAGTGAGGAATCCATGTGATGTTGAGTGTGTAATTTATGAAGCTAATAGAATGAAAATAGTAGTGGAGGAGTATATGTTAGGAATTGAGATTGAATGAAGCAAGCATATTGATTGTGGTGTGTTGTGTTGTGTTGTGCATGCAATGTGTCGGTTACTTTATTCCAAAACAGCGAATAAAAACAGACTTTGGGAAGATAAATAAATATTGTTGCAC

>Ca2:27801664-27801910

TATAGGTGGAGTCACCCCACATGCGATCAAAGAGTGAGGAATCCATGTGATGTTGAGTGTGTAATTTATGAAGCTAATAGAATGAAAATAGTAGTGGAGGAGTATATGTTAGGAATTGAGATTGAATGAAGCAAGCATATTGATTGTGGTGTGTTGTGTTGTGTTGTGCATGCAATGTGTCGGTTACTTTATTCCAAAACAGCGAATAAAAACAGACTTTGGGAAGATAAATAAATATTGTTGCAC

>Ca2:27800117-27800619

AGGAAGTAGTAAAAAATAAAGAAATACAACTAGAGTTTTTGTTTCTCTTATATTTTGTTATGAATTTGCAAAAGTCATCAAAATTCCCTAACTTGGAAAAGAAAAAGTTTATCTTTGCTTACCTTTGTTTTCATTTCTTTTGGAAAATAGTGCTTTTGCCAGATTTGTGTGCTTATTTATTAGATCTTCAATTCCTTTCCTTTCTATGACTCTATTGAAGACAGTGACACCTCTCTCTTCACTCATTTTTTCTTCTTTCTCCTTTCATTTCCTTTGCTTTCTTCTTTTATCATCTTGTTTATTTAATTTGCACTATGTTTATTATTTGATTTAATTAACTTACTCATTTTCTCTTTTTAGTTCATTTCTATTTATTTATACCCTTTTTGTCTTTACTATTGTGGTTCTCATTCTTTCAGTAGTATCATCCTTTTGTTGAAGCTTAATTTGGATTCATGATGGGATCTTTCACTTTATGAGAATATGGTAAATCTCTAATTAA

>Ca2:27800139-27800603

AATACAACTAGAGTTTTTGTTTCTCTTATATTTTGTTATGAATTTGCAAAAGTCATCAAAATTCCCTAACTTGGAAAAGAAAAAGTTTATCTTTGCTTACCTTTGTTTTCATTTCTTTTGGAAAATAGTGCTTTTGCCAGATTTGTGTGCTTATTTATTAGATCTTCAATTCCTTTCCTTTCTATGACTCTATTGAAGACAGTGACACCTCTCTCTTCACTCATTTTTTCTTCTTTCTCCTTTCATTTCCTTTGCTTTCTTCTTTTATCATCTTGTTTATTTAATTTGCACTATGTTTATTATTTGATTTAATTAACTTACTCATTTTCTCTTTTTAGTTCATTTCTATTTATTTATACCCTTTTTGTCTTTACTATTGTGGTTCTCATTCTTTCAGTAGTATCATCCTTTTGTTGAAGCTTAATTTGGATTCATGATGGGATCTTTCACTTTATGAGAATATG

>Ca2:28177280-28177574

ATTGAATTGGGCGAGAGCATCATCATTGATCAAAGAGAGCATAGAGTAGTTGCATGAGAAAATAAATAAATAAACATTTATTTATCTGGTGCAACACAAAAGCATATTCTGTTTATTTCCAAAAATAAATTTCACTTGACACTAGCGTTTTTGTTTTGGTGTCAATTTCTCATTACACCATATCACAATTCACCACCACACCCTTCTCTTCAAATTCAATCACACACGCATAAATCACCTTCTTCTCCCTCTCTTCTCTCTACACATACACACATTCATAACCTCTCTATCGCA

>Ca2:28556997-28557396

CGGATTTGCAGGTAACAAATTAGGGTTTAAGTGGGTTGATGCGGGCGGGTCCGGGTCCATGATTTCAAACCGTTGGTGACCGACTCGCCCGTATAAAAATATATATTATTATTAATATTAATAATTAATCATTTATTTATTGGGCTGGGACACTTACCACTCAAGCCCAACCCCAATTTCATTGTCATTTTTCTAAACCCTAGCCAACATTTCACTTTCTACGGCGGTCCACTTTCACAGGCTCCTCTCATCAGTTTCTCTCTCACTTCCACTTCAACCGTTCACACTTCCGTTTCTCTCCCACACTCAGTCTCACTATCATCTTCAACGATTCACTTGTGGCTTTCTCTATCGCTCAACCCTAAAAGGAGCAAGTATCTTGAGTCTTCAACGATTCAG

>Ca2:28576921-28577171

GATTTTAGGTTAGTCACGGAAGAAGTAGTAGCAGCAAGATTAGAAGTAGATGAGTAATTTTCCGGTTAAATGCTGGTAACTAGATTAATCCATAGAATTATAGTTCTAAATTTTTCTCTTGCATATTCAACTTTAATATTGCTGTAAAATGATTCAGTTGTAATTGCAATTCTTCACTTGCTATGCTGTTGCGAATACTGACGTTAATGTTGCAATTTAGAACTATATGCATGCAATGATCATCGGTGGT

>Ca2:28809651-28810280

CACGTGGTGGAAGTTAGGGTCGGCGACGGTGGTGTTGGGCGAGGCAGTGGTGGGAGAGGCGGTGTTGGGCGAGGCAGTGGTGTTGGGAAAGGACATTTTCGGAGGACTGTGGAAGAATGAGGAGAATGAGAATGAGAATGAGAATGATGAGAATGAGAATGAGGATGATGAGAATGAGATATGTATATATATAGGATGAAACCCAGGGAGATGAGTTTGGATGTTTCATTGTGAGAGAAGGGGTTATCAACAACAAAGTGTTTGAGCTTCTCCGATTTAGCCATTGCAAAATTTGGAAGTGGACAGAAAAGGAAAAGAAAGAGATGAATACTGTGAAGAAGGAGGAGTGGCTATTTCTATCTTCTTTAATTCGTTACCAATTGCGGAATGGGAGTGGTTGCAACTGCACACTATCGTACAAACTCAAACCTTAAATGTGCCTAATAAATTCATTCGAGCTGGACCACTTCAAAATATATCTGAATAATCTAAAATTCTCAATTATATATTAGATTTATTAATTTTAATTATAATTCAATTATAAAAGATATAATCTTAATTCAGTTAGACGTAGCAGATATGCAGTTGTGTTAAGTTAAGCACAACAAAGAAAATTAATTTTCTTAGGG

>Ca2:29537985-29538470

GAAATGTCAGATTTCGCAGAAGATGGAGAATGGGAAACTTGCTGGATGGCCCCAACATGAAACAGCTGAGCATGTTTAACATTTTTCTAAGCTTTAAGAATAATATTGCTATTGGATCTGTAGCCTGCTGAGAAGATGTAAAAAGTAATCTATTAGTACTTGTAATTTGAAAATAAATAGAAGTAAAAATTTCAATTATTTATTTAGCTGTTTCTGGTTTTCAGCTTTTAATTGCATTGGTTAGTGTCTAAGTTGGTTTATAGTATCATTTTATTTTATAGCTGGTGAATATGGTACTGTATAGATTGTATTAGAGTTGCGACTGGGTTCAGGATGTGTGTCGAAGCCTTCTCAGGATATGCTAGAAAAGAATAGAGGAGTAACTGAGTAAGCGCATTGACCATAATTGATAATACAGAAACCCATGTAATCAATACTGTACATTTTGAAATTCTTAATCAGCTGGTGAGTCATAATAAGATGTG

>Ca2:30178405-30178611

GATGAAGATCGACTACATAAGTTGGAGAATGCTCTATTTAGTTATATGAAAATACATAAGTTAAAGTAATATAGTTTAGACCGTATGAGTTATACCCTTTTATCTTACTAGTTGGGTCTATTTTTGTCCCAGATATTGGGTATATATAATTAGCTAAACCGAAAATGTAGCTTGGGGCCAAGTCTATTGTGTGCTTTGTATTGTAG

>Ca2:30258507-30258906

AGCAAGCTTATGGAGGCATATTAGAAAACAAGGTTAAATCAACATGCAAGAGATCATTCAACCATCAGACACAAGCTCATATTCACCAGCTAACCAGAGACATTCACAAGCACTAGCCATCAACTCGTAGTCACTAACTAGACACAATCATCAACTCACAGTGACCAACCAGCCAGACACAATCAAGAGCTCATAGTCATCAGCCATCGGCTCATAGTCAACAACCAGGCACAATCATCGACTCATAGTTACTAGTCATAAGTCCATGAGCCACCAACTGTTTTTGCTCATTAATTGTGTATGTTAAATAGGATTGTTAAGTTAGTTATTCTTGTTATTCTTCATGTTTTGGTTAGTTAGCAATTATAGCCGAAATTAAAAGTCTCTTTATATAAGGAT

>Ca2:30695793-30696048

ATATGTGCATGAAGTCAGCAGCACAGTCACCTGTAGTATCATGGCCCTGCGTATCATCAAATCAGTGCAAAATTGGCAATTTTAGTTGCCAATGTATTAAAAATCAATGTGTTTGTGGAAGACCCCCTGTTAATGATAACAATTCTATTCCAAAACGCTCAATTGCAAATTGAGTAATGAGATATATAGTGCGTCAAGCATAGTGTATAATAAGAAGGTTAATTTGTCATAAAAAATAATAAAAAGGTTGTCGAG

>Ca2:30865742-30865991

CTTTGACCAAAGAGAAAGAGGAAGGAAGAAAAGTCACACTTTCCTATCTTCTTCCTTTACCTTTCTCTTCTCATACATACATGTTGTTGCTGTTCCTCTTCAGCTTACAATAAAAAGCCCTTCTCATCCATTCCTTACTCTCTCATCGTATTTCAATCGAGTTCTTCTCGATCTCTACTCTTACTTTCAACAACAACAATTAAACCTTATTTCATCCACTGTTACTATTTCATTTCTACAATTCTAAAG

>Ca2:31214181-31214615

GATTTGAGATTCAGAGAATTGAGGCTGGTTTTTCGATGAGAATCAGAGAATGAGACTCCTGTTTAATGTGCCTCTTTTTGTTCTGAATTTGTTATATACTCCCTCTGGAATGAAAAGTAAGCGAAAATATATTTGAAAAATAGATGTATTTGATATCAAAAATAGATACAACTTTTCAATACTTTTATGCTTATATTTCATTCTAAATGGAGTATTGTGATTTAATTCTAATTGGCATGTTTAGCATTTTCAAGTTTCTGGAAACCTTCATAGGGGTGGATTGTAATATGGGTAAATTGGGTTTAGAAAAAGATGGTTTTGTGCTGTAAGAAGAAATTCTGAAATCTTACTTGACTATAACTTGTAACTATATTAAAGCCTTAATGCAAACAAGGAATTAAAGTGGCTTCGTGTGGAATTTGACTTCTCAAGTG

>Ca2:31534185-31534576

TGACAAGCAAGACTAGCTAATAGGAAAGCAACGTGAACAGCTAATAGGAAAAAGTATCGTGAACAGCCTGGCAGCAGCCATTCATACAGGCTGGCAAGCAAGCATGCTTTCCTAAAGCCTATCAACAACAAAAAAGCAATCTTGGCATAAATTGGAAAATTTGAAGCTAAAGCATCCAATAGAAAATAAAACCGTTTTACGCTTTACTATCACAACTAAAATGATCTCAACCAAAATCAACATCCACACAAACAAAATAAAATTTCATCTTTTAAATTTAAATTTTAGCTTCCAATCTAAGCAGCAAACATGAAGAAAATGAGAAGATACATGCCTTTTCAGATACCGATGAAGACAGAGACGGTGGTTCGCGGTGGAAGTTGAAACAACG

>Ca2:31534947-31535295

TTAAAAGAGGAACAGATGTACCAATAAGCATTGCTTGTGCATCAATGGGAGTCATCATCAACATAGCAGGGTTTGGCAGTGGACAAAGTCAACATAGCATGGCTTGATCTGATGTAATTTATTTTTGCCTTTATTCTTAGTTTGTACTTTGATTTGGTTTTAAAACTCTAATTAGATTTGAAATTGTAAATTTATAATACAAAATAAAATTATTTATTGTAATTATTTTATTTCCTTTTTTAAATACATATTTCTTTTTTGATTTATTTTTAAATAAATTGGACAAAATTAGGGTACTACAAACATTAAAATGTATCATTTGTCCTTTACATTAGTTTAATTTTGGCT

>Ca2:31534185-31535295

TGACAAGCAAGACTAGCTAATAGGAAAGCAACGTGAACAGCTAATAGGAAAAAGTATCGTGAACAGCCTGGCAGCAGCCATTCATACAGGCTGGCAAGCAAGCATGCTTTCCTAAAGCCTATCAACAACAAAAAAGCAATCTTGGCATAAATTGGAAAATTTGAAGCTAAAGCATCCAATAGAAAATAAAACCGTTTTACGCTTTACTATCACAACTAAAATGATCTCAACCAAAATCAACATCCACACAAACAAAATAAAATTTCATCTTTTAAATTTAAATTTTAGCTTCCAATCTAAGCAGCAAACATGAAGAAAATGAGAAGATACATGCCTTTTCAGATACCGATGAAGACAGAGACGGTGGTTCGCGGTGGAAGTTGAAACAACGGTGATGCTTTCTGTTTCTTCTTCTTCTCTTTCTTTTTATGTGTTTTACATCAATATTAATCTCAGTTTTCTCCTCCTCTCTTTGAAATTTGATAAGGTGTATTTATAGAGTTTTTTTATCTGTTTAGTTGCTTTCTTTGCCTGTCTTGGTCCTTCTCTATCAATTTCTTGTTAGATGTTTTGATTTCTTTGCTCATCATCCGCATTTGTCTGCACCTTTGATGCCTTCATTATTTGTTCTGATATGTTCATTGCTGAGCTTTTATTTGTCTTTTAGTTTTTTTTTTTTAACTCTAAAATTTGTCTCTTTGTTCTGCACAATGTGTACCAAGACTGATTTAAAAAATTATGTTTTTATGTTTCTCAAATTAGTTAAAAGAGGAACAGATGTACCAATAAGCATTGCTTGTGCATCAATGGGAGTCATCATCAACATAGCAGGGTTTGGCAGTGGACAAAGTCAACATAGCATGGCTTGATCTGATGTAATTTATTTTTGCCTTTATTCTTAGTTTGTACTTTGATTTGGTTTTAAAACTCTAATTAGATTTGAAATTGTAAATTTATAATACAAAATAAAATTATTTATTGTAATTATTTTATTTCCTTTTTTAAATACATATTTCTTTTTTGATTTATTTTTAAATAAATTGGACAAAATTAGGGTACTACAAACATTAAAATGTATCATTTGTCCTTTACATTAGTTTAATTTTGGCT

>Ca2:31756524-31757141

TACAAAACATACCTAAACCTAATTCAGTTTTCTGTTTATTTCTCTCTCTTCCATCCAGCCTTCCTCTCTCCCTCTCTCTCTTTTGCTTACAAGTTCCAGCCTTCCTCTCTCCCTCCTCCATAATCAGATTCATGAATCAAGACAAACGTGCGGTCGTCTCTCTACGACAATCGCTCGTCTGCTCGCTCACGAAATCGCTCGCTGCGGCCAATCGTTCGTCTGCCTGACTGCCTCTGTCCGTGGCCATTCGTCGCTGCCTCTCTACTCGCCGCAACCTCATCAGTCATCTGTCTCTAGATCTTGTTGCTGCTTTGTTCAAAATGTTCGAATTTCTATTTTGTTATTGCTGATTTTTGTTAGTTAGTGTCGATTTTTGTTAGGTTTGATATTTTTTAGCATAAAATTTAAAACTTATATTGTTATTGTTGAGTTTAGAGTTTTGATTTGGAGTTTGAAATTTTGATTTGGAGTTTTAATTAGCAGATTTTTGTTTAGAGTTTTTGTGTTGCACATAAGGTGTTTGAATTTATGTCAGTATAAAGTGTTTGAGTTTTTTTTTGTCTCACATAAGGTGTTTGATTTTATGTGTTTGATTTTTTTTTTTGCATACAAAGTGT

>Ca2:32338609-32338972

GAAAAGTTTTTCTAAGCATCTCTTATTGTACTATTTGCAACATTGATGAAGAAAGGTAGGAGAAGAAGTAAATGATGTGATCCACGAATACAAACGAGTTTGAAGTTCTATGTATCAATAATTTAAATAATTAATTTGTGAAGCATATGTTGTGAGATACTATTAATATTGTTATTATGAAAACATGTTTTATAATCTAATTATTAGGATAACATTTTGATGTCATTCTTAGTCCAGCTTATTTTTCGATAATAGTTACATGTAAGTTATTTGAAGAATAAGTAGCAACCAAAATTTCCCATTACTAAATTGAAGGAAATGGTTTAGAAATAACTAAAATGTTTGTGAATCACTATATTTTAT

>Ca2:32431137-32431457

AAGGCCCAGATCTTTCTCGAACAGAAGGAGCCAACTAACCAAGAACACTTATAAGGAAGGATTCAACAACTCAAACTCAAACTTCATTCTTCATTCATCGATTAATTTATAATCAATTAGTCATAATCTCTTTTTCTTTTCTTAACCAACACGAGAGAGAGAGAGATGGATTTTGAACTTCGAAGAGCGAGCGAGAAGATAGCGAAAGAGCAAAAGGAGAGAAAGGAAAAAGCGAGATTGAAACTGCAAAAGGAAAAGAAAGCGAAAGAAGATGCTCGAATACAAAGCGAAGCAATCGAAGCTGCTCAAAGATCTCGTNN

>Ca2:33936096-33936433

TGTTGACATGTTTTGGACAACGACGATCGACATTGCCAAAAAATTTTCCTTTCTTTCCTTTGTTTTCTTCACTTCTCAGTAAGGAACCAAAAGCAAAGAAACAAAAACCCTTCTTCCACCATATATCGTCGCCGCCTAGCAAAAGGAAACTTGCAAAACCCTTCTCTGTTTTATTTCCGTTCATTGTCGTTATCTCGTTTGCTCTCTCTAATTTGTTCTATTTTCACGATTATTTGTTGTGTAAATGAACGTTTATTAACTTATTTGCAAACCCTAACTTGTTCTCTCGTTTTATTCTTGTACAATTCATTGAAATCCCTAACATCTCATTTCACAG

>Ca2:33936105-33936433

GTTTTGGACAACGACGATCGACATTGCCAAAAAATTTTCCTTTCTTTCCTTTGTTTTCTTCACTTCTCAGTAAGGAACCAAAAGCAAAGAAACAAAAACCCTTCTTCCACCATATATCGTCGCCGCCTAGCAAAAGGAAACTTGCAAAACCCTTCTCTGTTTTATTTCCGTTCATTGTCGTTATCTCGTTTGCTCTCTCTAATTTGTTCTATTTTCACGATTATTTGTTGTGTAAATGAACGTTTATTAACTTATTTGCAAACCCTAACTTGTTCTCTCGTTTTATTCTTGTACAATTCATTGAAATCCCTAACATCTCATTTCACAG

>Ca2:33937812-33938141

ATGCATCTCTGGAGATTCTTGGTTCAGTTACTCTATATCCTTTACTGACCAAGCTGATTTACCAATCAAGGATCACAAGTTCACAACTGAAGAGGTATTTGTTGGGTATATAAAATGAACTCAAGTTTTTTAGTAGTCTGTAGAGTTTTGTACAATGCTTAAAATAGTTTGTAGAATTTTGTAGTCTGAAGAGGTATTTGTTGGGTATATATTATGGTTGCAGTCAATGAGTGTAGAGAGAAAGATGGAAAGCTTATATGTCTCAATTACGTTGATTCTCAATCTGTCATCCTGGTAATAAGGCCATATTGCTGCTTTTTCTCATTCCG

>Ca2:33936135-33936433

AAAATTTTCCTTTCTTTCCTTTGTTTTCTTCACTTCTCAGTAAGGAACCAAAAGCAAAGAAACAAAAACCCTTCTTCCACCATATATCGTCGCCGCCTAGCAAAAGGAAACTTGCAAAACCCTTCTCTGTTTTATTTCCGTTCATTGTCGTTATCTCGTTTGCTCTCTCTAATTTGTTCTATTTTCACGATTATTTGTTGTGTAAATGAACGTTTATTAACTTATTTGCAAACCCTAACTTGTTCTCTCGTTTTATTCTTGTACAATTCATTGAAATCCCTAACATCTCATTTCACAG

>Ca2:34828403-34828659

CAACAATCTTCGTGTAACGCTCCTTGCGCAAACTCTTTCATTCTCACGCAAAACAATCTTCACTGTCTTCTCTCTCAAGAACCCACTTGACATCGCTCGACCTGAATGAAAAAGGGGAAAAACATGATTAGGGTTTCTGTGTCGAGTACAGTTTTCTCGTGATTTTCCCCCTTTTCTCGCAACTCTACATCGATTAGGGTTTTGGCATCTTGCGAGACTTGCAAAAGTTTTACCTCTTCGTGACTTGGTGAGCTTG

>Ca2:34830404-34831240

ATATAATAATTTATGATATTATTTTTTTCACTCCTTGGTTCCCCACGGATTGAAAACCGTTTTCCTTTTGAATTTTTCATCTGTTTGTTAATTATTAGTTGTCTAGGTCCTATATTACATGAAACTATTCTCTTTTTTATTTATTTATATTATATAGAGGGGTGGTTGTTGTTTTCTTAGGATGAAAAGACTGGGATTTGAATTTGTTTAAATAAATATTTGATAAGGGTTAGAAACCGAGTATTAAGTCTAAGATGTTGTGTTAAGGGAAAATGACAGGGATACCCTATTTTGACTTTCATCATTGTGCTGCTGTTTGTGCCTTTGTCGTCACAAATTTGGAAAAAAGGTGAAGGGGAAGCCTGCAAAACTGTCAACAATGCTTATGTCTAATTAAGCTTATGTCTAATTTGTTTTTTATTCTTGTAAGATTGATTTTTGATCATACTGAAATTTTGTTTCATTAATGGAAGATGAATGCAATGTCCATTTTGTGATTTTTCAAATACTTGTTACAATGAGAGATCTGATGCTTACATGCATCAGTAACAGAAAACAATGTGGATGTATACTTTGAAACTAAACAATACAAAAAGGAACTCAAAGCAGCCGATGCCATTTTGAAAAAAATTCCAGATCATGGAGGTATACTTTTTCGTGCTTGCTTTTACTTCGTCATCTTGAATGTTTTATTTCCTTGTTACATGTTTCAGTTAAGTAACACACCACCTATCTTTTTTACTTAAGTTACTTATCTTCTCTCCATTTCTTAATTATCATGATTCTTTGGGTATTTTTTTTATTTATTTATGTGTCTGTTCCTCCTTCTGGTTGAT

>Ca2:34844313-34844667

GTTTGTGTTCTCAGAAATCCAAAGCAAAACATTCACAAAAAAGCTTCCACAACCAATAATAAGCAAAAGCAAAGTCTTCCAATTTAATTTTCCCGTTTTTCTATCAAATGGTCTCCAAGGGACCAATCACACTTCTGAATAGTAAGCATCATAGTTCTTCTATAAACAGGTTGATTGGTTTTTTTTAATTTTTATAGACAAATGTTAGTTGTTACGGTAAATTGAGTTTAAATCTGATTTGATTCTCAAACTCTTTTGGATGTTTCAACTCAACAAATTGATATTGTAATTTGCTTTGTTCCAATTGTATTTGTATTATTTATTTTTGTTGCTTGTTCACAACATAAATTTTCA

>Ca2:35154922-35155195

TGAGATGAAGGTCTCCTCCATTAAAAGCACCTCTTCATGTGTTTTGCTAACACTAGAGAAGTTTTACGCAGCATTTGATGTGACCCCAAATAAAGATGTAGTTTGTTTTGATCGATGTGTTTTGTCCACATGTTTGCAAGCAGATGTGTGCTGTGTCAGTCGTAGATGTTGAAAAGGTTTTTGATGTAGAAGAATACATGTATCAAGAATATCAAGATATCTATCATTTTCTTCCTCTGCAACCTGTACTCTGTTTAATAAAATGTTTGTTCC

>Ca2:35207448-35207822

CTATTATCGTGTTATTGGCATCACGTTAAACTGTTTAATCATAAATGTGAAATTTAAATAAAATATTTAAATTTTTGTATATTTTATTGAATGTACTGTATATGTGTATGACATATAATTCATATCCTTATCATAAAATAATAATAATAATAATTCATAGCATACCCAATATAGCAATATTTGTGTTATGTTATATAGTAGTAGTATATGTACTCATCTCCAAATTATACAAACACAAGAAAAACACTGACTCACATAAGTTCCCGACAGTTCCAAATTACTAGGTACCCGTTGATTGTGGGATTAGAAACAAACAACATACTAGGAATATATTTCACTCTGATTTTCACTTAACTGAACTTTCCTTAACACAG

>Ca2:35386534-35386830

TGAAGATAATCAATAATATTTGACAAAACATCGAAAGTGATAGCACACTTATCATCTTGAGACATCTTATCTTATCATGTTAAAAGAAAAAGATATGACAATAGCAAATAAAGAAAGATACATTTAGGAAATATGCAATTTTAATTAAATAGTGTCATTTTATATGCAGTGGCGGAGCTTAGTAGGGACAGGGGGGCCGCGGCCACCCCAAGCCTTCCTCTCTCTTCCAAGCCTTCATCTCTCTCTCCCCCGCGGCCACCCCAAGCATTCCTCTCTCTTCCAAGCCTTCATCTCNN

>Ca2:35386968-35387665

CACCCCAAGCATTCCTCTCTCTTCCAAGCCTTCATCTCTCTCTCCCTTTTGCAAAGTTACAACCTTTCTCTCTCCCTTCTCCAGAAATCACAACAATCGCGTTGCCGCCTCAGATCACTTATCTGCTTCTCTGCCAGATCAGGAATCAATCGCTCCTCTATTCGCTGCAGGCCTGCGGCCGCCTCTGTCTGACTGCCTTCGTGGCCGTTCGATCTCACCTCTCTGTTCGCCGCAACTTCATCTGTCTCCAGATCGTGTTGCTGCTTTTATTAGTACTGATTTTTATTAGTTAATTTTGATTTGGAATTCTGTTAGTTCGAGTCGCTCCTCTGTTAGTTAGTTAGTGCGTTTTGATTTCAATTCTGTTAATTTTCAGATCGTTGCTCCTGTGTTAGAATTTAACTTATATGGCAGTGCCCCATATTTGTGTGTAATTCTAGCTTATTTGTTCAGTTAGCATGAAATTTAAAGGTTATTTTGTTAGTGTTGACTTTGAAGCTTTGATTTGGAGTTTGAAATTTTGATTTGGAGTTTCAATTAGACATTAGTGCTGATTTTTTGTTTAGAGTTTTTGTGTTGCACATAAGGTGTTTGATTTTATGTCCATATGAAGTGTTTTAGTTTTTTTTGTTGCACATAAGGTGTTTGATTTTATGTTCATAAAGTGTTTGAGTTTTTTTTGCTGAATATAAGGTGT

>Ca2:35544181-35544864

AATGGTTCTATAAACTTTTTATTTGTTGGTTTGGTTTCATGAGAAACATTGTTTTGGAGAATCAACAAGTATCGTGTCATGTTTAGCACATGCTTTCGATGATTTCAAGGCTTTGATGTTGTAAAATATTGATGCAAAACAAACACAATTGCTATCTCCAAGAGAATCAGTTTCATATAATAAATAACAAAAGGTTTCATGTCAGTGTGAAGAAGAAAAAAAAAAATTGGATTTTTGAAGTGGAAAATAGCTTTGACCATTCAAAAACCAAATCAACAAATCTCACACTCAAAGTTTGATGAAGAAATGTATTAATTGCTTTGAAAGTTCTTCAAGCTATGATCTTTTGAATCTTCTTCATATATATAGTAGTAGCAAAAAAAAAACCAAAACTTTCCCTCAGCTTCTTCATTGTCACTTTTTAACAACATTTTACCAAAAGGGTAGCAAGAAAGAACCTTAAAGTTATCAACTTCAACCTTAAAAGCCTCTTTTATAACCCCTTTTGTCCAACATTGAAATTTCTCTATAGTCTTTATTTCTTATTTTCACTCAATAGTACTTCTTAGTTCTTATAACCTTTGTTGGGTTATTTGGATCCTTAAAAGTTTGGTTTTTGACACCACAGAATATGATGATGATAATGGAGAAAGTAACCAAGCAACAAATCTATCACAAGCTAG

>Ca2:35601513-35601877

AAAAGTTGGTTTTGCTGCATACATAGTTCTCCAGCAGAATCGTAGCAATGATTGGTCATATGTACATGGTTGAATGGAAGTCGAGTCATGTGTAACTAGCTCCTAATGTTTTCTCAAGGATATGAGCTGTTAAGCATAATTAATTCTTCTTGGATTCAGCTTTTGTTATGTAAAAGTAAGCAGCAGTGGGGGAGAGTAAAATAACAGACTATTTTTTGTATGTATTTATATATGAATGTCGTGGTTTACTATTAGGAGCAAAGGTTGCTCTCTTTTTTGCCTTATCTTGCTCTATTGGTTGTAGAGGTTTACTTGTTCTATTATACTTCATGTATTACAATACATATCTTGTATCTTATGAGCT

>Ca2:35697420-35697734

AATGCAAAAATATCCAATCCTCAAGAGGAATCTGCAGTATCTAAGGCGAATAATTTTTTAAGTAAAATTATTTATTGCTTTCATTTTGGTCCTGCAAAGAAGCTTTAAATCACGCAATGTTCATCATTTCAACATGTTGAAGTTGGTTGTAAAGATTCAAATCTAAGCACAACAACCCAAATGAGGATGATGGAAATGAAAATGGCCCAATAAGGACGAGTCCAACCCAAAGAAGGTGCTAGTGCTACTACAGTACTACTGCAGGGGAAGAGAAAGATACTTCCTCAGAAATCAGAACCTCCAAAATTACATGC

>Ca2:35697420-35697936

AATGCAAAAATATCCAATCCTCAAGAGGAATCTGCAGTATCTAAGGCGAATAATTTTTTAAGTAAAATTATTTATTGCTTTCATTTTGGTCCTGCAAAGAAGCTTTAAATCACGCAATGTTCATCATTTCAACATGTTGAAGTTGGTTGTAAAGATTCAAATCTAAGCACAACAACCCAAATGAGGATGATGGAAATGAAAATGGCCCAATAAGGACGAGTCCAACCCAAAGAAGGTGCTAGTGCTACTACAGTACTACTGCAGGGGAAGAGAAAGATACTTCCTCAGAAATCAGAACCTCCAAAATTACATGCATGTAAGAATGAGAAGAGATCATGTTACTAATAGCCAAATTCTTGCAAAATGTGCAAGGATTATTGCCTTTGCTTGGCAATGGCACAAAAATATGTACTTGACATTGTATAAACTTGCCTTAATATGGGTACAGGAGGAGAAAATCAGTGAGAGGATGAAGATTCTCCAAGATCTTGTACCAAGTTACAATAAGGGGTGCAG

>Ca2:36074841-36075166

GTCATGGTTCTTGCCAATATGACTTCATTCCCTAATGAGTACTAATTGCAACTGTCAATGATGAAGACTCAAAACAATTGCACCAAAAATTAAAGTTGGGCTATTTTTGTTTTTCTTGTTGAATACAAATCAAGCAATGTGCACTTCACAATGTCTTAAAATATGTTGATTTTGTCTTTTCCCTTGTGGTCCTCTCTCCCATTGGATTGGTTGGAGGTTTTTCACATGCTAGTATGGTATTAATATTGGACTTTTCTTTATAAAATTGGAATTTATATTCTTGAAATTTAAAATAAATAAAAAAGGGGAAGATTCTTCTAATAGC

>Ca2:36202653-36202919

CTTTTTTACTTTACTGTCAATATGTCATTTTTGTTTTCTTTGCTATTTCTATAGATCTCTCTCATTTCATACTGCCTTTGATATTCACTCTCATTTCACTCTTCTCTACATTTATATTACTTAACTACTAACTCATTCAAATTCCTAGATCTTGCTTTATATATTCCATTTTCTGTTGAATGATTTTCTGTTTTTGGTCTGTGTGTGTTAAGTTTTGCAAGATCTGGTTTACACATTTTGTTAGGGCAATTCTAGACAAATCTTGA

>Ca2:36385665-36386593

AGTAATCCATTGGATGTTAACTAAAAATTGCAGAAGGAGAGAAGCATTGAGATTTGAGAATGTGGTGGAGTGCATTTTGATTGTTTGGGGTGGGCGGTGAGGAGCCAAGAAGATTAGTTGGGTCGCAAACAAAATATATATGAAATTGTGAATGAGTGAGTGTATTTTACTTGCATTCTTACCGTATCAAATAAAATGAAAGTCTAACGATAATAACCACAATTCATTACTCCCAAATTTTATTATATTGTGAGTTCAAATGAAATGATAAAATATATAAGTACCTTGACATACTGCAAAGCACTCTACTAGTACATACATGAGTTCTCTTGTCGAAGAACAAACCTTGACGGTCACTTGTGGAGATCAATTTGGATTGGGGCTACGGTAGAGGTAAAAATCTGTTTCCTCCGAGTCTCAGGTACAAATGCGGGGAAATAGAAAGAAAGTTGAAGAAACTATGGGCAATGGTTCTTGAATCATTGTTTTTTACATGTACATCTAAATACTCCAAAAATTTCACATTAAGAGCCTCAATGGCTATCCCGTCTTATACTATACTATATGAAGTAATATGAATAAAAAGAATGCCTTATCAAACCTTCCCACAGATTTGAGTTTCAGAAGCTTCAACATATCAGTGGCCTATATTTACAGTTGCTACAAGTCTACAATCTAGACTACATTTTTTAGGGGAGCTCCACATCAAAATCTAATCAACTATGTACAAAAGGTTTATTTTTCAATACTGTCTGAGAAAAGAAGTTTGAAGATCTTCTCATTGCAAGAAGACGAAAAAACAATCAGGGATAAGTTGCTATATTTCAGGGTTAGAGATGCTATGAATTGGAACATCTAATCTGAGAAGAACTCTTGCACTTCAGCTTGCTCATTTCCCATTAGAAGAGGGATTGAATCAATTTTAGTT

>Ca2:36552288-36552716

CAATGACAAGTTAGTTGAGGTTTGTACTCAGAGTTGCTTTCTGATCTCTTCGCAAATGTGGAGAGAAGAAATTTTTTAGGTATCAAACAGCTGATTTTGGGTATCTCTCTCCTCCTTAACTCTCTCTTCTCTATTTCTACTATAACAAACACACGTACGCAATTACTGTTTTCTTGGTAGTTGTAAGGATCAAAATATTTTCACAGAACAACCATCAAAATGACGGAACCTTAGCCCCAAATCTAACCCCTAGTCCCCCAATCGATTTCACCTTTGCTATCCCACTCTCTTTCCGACGGAGGCGCAACTTCTCCGGCGTTTCTCTGTGCGCGACCACATCTCCGACGCAACCCTCTGTGCGTGACCACGTATCCGGCGCGACCCTTCTCTGTGCTTAACCCTTCTCCGGCGCGACTCCGTCTCCGGCG

>Ca2:36609194-36609662

GTTGGTATCTGGCTTAAGCATTGCTGGAAGGATAGGTGATGCCAGTGTTGTTATGTCACAATTGAAACGGGCTGGGTTCAGTCTTGATACATATGAGGCAGTTATACATTCATTCATATTGTAATTTGAGCTATAAGTTAAATGCTTAATCGTATAAATAGTAATTTTTTTCTCTTTATATAATATATTCAAGATTAGATTACACGATGCAAATACATTTTTTAATACCATAAATAATACAAATGACATCAATTTATGCAATAGATTTTGAGTGTAGTTTACAAATCCTTAAAAGAACTGATTTTTTACTATATAAATTATTTTATTGTAATTGTTTATTCACGGCTATTATGGTAAAAAATGTGTTGACATTCCTCAATTAGGATAAGAAAGAATCTTAGAAAACATGTTGGGGACATTGATGCTGGAGCAGTCTCACGTGGTTTTGGACGTGCAATTGGCAACAAG

>Ca2:36610250-36610574

GAAGAATGTCAAGATCTGTATCAGAGAATGAGAAATAGTGTTTTCAAAAAGCCACCAATAGTAAGATTGTTGTGTGTGAGTGATTTTTTTTTTTTCAATAAAAATAAAAAATTATGCTTCATGATCTGATCAATTATGAATAAGGTCCTTGTAAATCTTTTCAGATTTTAATAGTAGGAATTATTATTATGCAGTGCAATATTATGGTGGCTGTACAAGATAATATCTCTAAAAGAAAGGGCAAATGAAGTTCAATAATATAGTAGTAATAACTAATAAAAGTAATACATCTAAAGAAGCTTTTTAGCAGAACATTTACATCAG

>Ca2:36609773-36610078

GAATAAACAGATCTTCAAAGAAAGAAGAAATCAACCAAGAAATTATGCCCCGGCTTCTTAATGTGCCAAAAGATGTGTTCCTATCTGCAAAATTTCAGACGATGATTTTTGAGGTATTTGTATTGTGCTAATGCTAACATAATTTTCTGCTGCAATTTCCCCTGTTTTTGGAGACCTGGTTGTAAACGAAGTAGCTGTTTCCTAGTAAAAGTGGATAACTCAATTTCCAAACCATTTTTCTACAAGATGTTGAATATATTGTATTATTTCCTGCATCAGAGCATAGCTGGGTTTCTGATGTGAAG

>Ca2:36613008-36613310

CATTGATGTATCCATACCTTAATTTTTTTTAAAAAAATACCATATCACGTACTCGTACCAGTACCTGATACTAGTACCGTACCTATGCGTCATAGATCTTATGCTCATACAATTTTCATGCCACTGTATTGATGTTACCATATATTTCCTCTGATTATGATATCATTGTGCAAACATATCGGTCTGTGAACATGAGTTTGAAGTAGAGGTTGTGTCGTTGGGTTGGGCTCTATGCTCACTAAGCTTGATTCTGATTTGCAACGGAAAATTAGGTGCACATGCTTGAAGTGTGGTAATGAGGT

>Ca2:36614997-36615667

TTTATATTTTGTTCTAATTAATGATGTGGAAGATGATTGGGATATTGTTTGCATTTTATATACGAAATAATGGCTGAAACATTCCCTATGCTATAAAGCATGGAGTACTTCTAAAATGGTGAAGTACCCATACTGGGTGCTTACCCAGTACTGGTACTTGTTATTTGTGGGTATTCTGTTTTTATTTATTTTATTTCGAGTGAAATTCATGATTTTGTTATATATTACTATAATATTATTTGTTTATTTGAGTTTATTTGTTATTGTAATCGAACACAATTTGTGATTTTTTAGACATTTATTTTGATTTTGTATTCTGAATTGAACAATTTAACTCTCTTAGTTAAATTATATAAAAAAATTAATGTTTTTCATTGATGTATCAATACCTTAATTTAAAAAAAAAATACCATATCACATACTCGTACCAGTACCTGATACTAGTACCGTACCTATGCGTCATAGATCTTATGCTCATACAATTTTCATGCCACTGTATTGATGTTACCATATATTTCCTCTGATTATGATATCATTGTGCAAACATATCGGTCTATGAACAGGAGTTTGAAGTAGAGGTTGTGTCGTTGGGTTGGGCTCTATGCTCACTAAGTTTGATTCTGATTTGCAACGGAAGATTAGGTGCACATGCTTGAAGTGTGGTAATGAG

>Ca2:36616722-36617047

TAACGGTACCTGGTCGCATGTGCAAGAGCGAAGTGATTGTTATATTTTTTTTTTAGTTTTCCTACTTTTTGGTCGCATGTGCAAAAGTGAAGTGGTAATTATATTTTTTTACCTTTCTTACTTTCATTTCTACGGGTCAAGTGGAAATCATATTCTTTGGTTGCTCGTTGCTCATTCAGGTTAGGACTAAAACTAAAACCTTTGGATTCTCACTCATTTTAAACATGTTTTATGTTTTGTGTCTACCTAATCCAACATGCCTTAAATTATTTAGTACTTGGGTTACACATTATATTAAAGGTTTTGTCTATGGGTTTGAAGTCGG

>Ca2:36632416-36633015

ACATGTTCTTCTTCCCACACACCAACAACAACACACATCCCAATGACACTTTTTCTTTTAAACTTCTCTCTTCTTCGACAATCTCATTAGTGCATCGTTTGCAACACTAGGAGTATATTCGGGACCGTTGTAACCCCCTCTTTCATTCCCACTAAGAATCAAAACTCGAAAGGTTATATTTGTCGTACCGAAAAAACAAACCACCAACTTCATACCGTCTTCCCGGCCACAACCAATGTTGTTAGTGAAAACCGAGACCGCCTAAGAAATCCACACAACACCTTCTTTCATCTTCTATGTACGAAAACACCAGAAACATTTGTTGTTAGTTCTGATCAGCTCGTTCGTCGTTTTTGAAAAACCTACTTTGTTTTTGGAATATTTGCGCCGAAACATTTGAGACGAGAAGAACTATTAGTTTGCGTGTCATTCGGAAGTTGACGTCAAACTATTTCGAGGTTTTGGTGAGACCTTTCGTTGACTTTTGTTTTGTCTCGTCGTTGTCGTTGGTGCTGAAAGTCTCCCTTGAGTTTGGAGCTTGTATGGAAGCTTTGGAATGGGGAGCAAAACCCACTCGAGGTGGTAAAGCTAGATTTGAG

>Ca2:36634160-36634496

GATATGATTTTGGGGAGGAAAAGCGAATTCCTCACTGATGTGATAGAATAATGTATCGTGATACTCGGGCAGCTGCCGTGTCCTCAATTTTACAGTATGGGACATTGTATGACATCAGATTTTTGATCCAGAATACGAGATCACTTCTGAGGGGCAGTCTATTGTCAAGACTCATGGAGAAGCACTGGATTATATCCGAAGAGCTGCACTTGGGCTTTCCTAGATGGCTTGAGGTACTTTATATTTTACTTGGTTATTTATGTTACTTTTGTAGGTTAGCTATTGTAAGAAAATACATATAATAAAAAGATAATAATAACATATAATAAAATAGAG

>Ca2:903569-903862

TGCAAGTTTCATTCCTCAAAACAGCATAACCGGCTTGTATATTCTTGTTCACATTGAAAACTTCACAACAATATGAGCCTTGAAGGATCAAACATATAGAGCTACCATATATAGATTACAGTTGACTACCAATTTCAGTAAATGATGCTTACTTTCCTTATGGCAAACTATTAGAAAAGCTAGCTTAAGATTACATTCAAGCATAAATCATCAGAAAATTTCTAATACCAAGGTAACCAATAGACTAGGAAGCAACAGTCAAATTCAAATATCCTTCTGCAATAATTAAGGAG

>Ca2:1105217-1105460

ATGCAAATGTTGTTTTATAAGGAAGTACTACAAGATTCAAAACATAGAATGTGATTGGTTGTGGTCGAAGCCTGAGACAACCTAGGCCCATTTTGATTAAGAATGCCTCCCAATATGTAGTTGATGGAACTTCTTTAGTTGGGCTTACGTCTGATTACATTTTGCCTTACTAATTTGCACCGTCACCTTTTTCTTATACACTCCTCCACGATTTTCTATATGAAATATATTTAGGAATAAGAG

>Ca2:1471947-1472430

GTAGAAATGAAAGTAAGAAAGGTAAAAAAGTATAATTACCACTTCACTTTAGCACATGCGACCAAAAAAGTAGGAAAACTAAAAAAAAATATAACAATCACTCCGATCTTGCACATGCAGCCAGGTACCGTTACTGCAGAAAGAAAATCTATTTTAGATTCAAGTTTTTCATGAAGTTATACAAAAGTAATTTCAATTAAAAAAAGTAATGAAGTAATTTATATTTACGAATGAATTTCATATATTACTTATGTTTTGGTTATTCACAAAAAGGTTTGTAGCCACTTAATGAAGGGAATCTTAAAATGCTCATAACAAAAAGAGAAAGTTGTGGATGTCTTTACAATGCCGAGCAAATCAAGTATTCATATATCAAATCTCTATATAAATAAATCAATCCATTGATTTGTCATTTCGGCTTCCCCAACTAATCCTACACTTTAACTGTTTTTCAGCTTGATCCATAATGACTATTTCGAATTT

>Ca2:1478220-1478585

GTCATAGTATCGTAATAGAAAGTTCATGATCAACCCCTTCAAAGTTCAAACAGTTATTCAATCTGATAACCAAATAAAGTAATGTGATTCCACAACATATTACTAAAATAATATGGTTTGAAAATGAGCTCATAAGAGTACGAAAGTTTGGAGCTTAGACAATTAAAAGCTAAGCATTTAACTTATATCTCAAGCCTACTACTAACAGAGTGACTTCATGGCCTCATCAACATTTTTCTTTAAACAATATGAATGAATGTATAACTGCCTCATATGTATCAAGACTGAACCCAACCCATTTCAACTGTGACATAACAGCACTGACATCACTTATCCTTCCAGCAATGCTTAAGCCAGATACCAAA

>Ca2:1592389-1593067

CACTTTTTAAGATATCAAGGCATTGAGTTCACAACTTCCAAAGATATATCCTCTTTTGATCTCTTAACAAGTGTCCCAAGAGCACTTGTCCTAAAATAAAACAAGGCCACCACATCGGTTCACCTATGATGATACAGGATAAGTTGTCACTTCACTGCCTACGTTATATAATACACCGTAATGCTACCAATGGCATTGCAAGAAAATCATACAAGATTTTTCATCTAAGAAATATGAAAGGGGTCGCACACAGTAGTGTCATACTATGTATGATGGCACCTAACCTAAGGCACTAGTAACAAAAATAATAAACTTTCAAATAAGATTAGATCGTATCAGAAGTAGTATTGCGCAATATTACGTTAGTTGTCGAACAGAGTGAGATCTCTCCAGATTAAGCTTATAGACAGCACTGTGCAACCATCAGTAGAGGGCCAGCACATCTCATCAGCTTTGGTCTCGTTACGTGTATATGTAGTCGAAGAATCAGCAGCATCAGACTATCAGTGCCGCCACTTATCATGACTCATAAGTGTTAACAGCAACAAATAAATGTACATGCATGACAGGGCTTCACTCGATGCCAAAAGACAACGCATCAAACTATTCAGTCAGAGTCATGAAACATCCTGAAGTGCCAGATTCAACTCATGTGCAAATTTTGCAATTGATCCTCCT

>Ca2:1592389-1593070

CACTTTTTAAGATATCAAGGCATTGAGTTCACAACTTCCAAAGATATATCCTCTTTTGATCTCTTAACAAGTGTCCCAAGAGCACTTGTCCTAAAATAAAACAAGGCCACCACATCGGTTCACCTATGATGATACAGGATAAGTTGTCACTTCACTGCCTACGTTATATAATACACCGTAATGCTACCAATGGCATTGCAAGAAAATCATACAAGATTTTTCATCTAAGAAATATGAAAGGGGTCGCACACAGTAGTGTCATACTATGTATGATGGCACCTAACCTAAGGCACTAGTAACAAAAATAATAAACTTTCAAATAAGATTAGATCGTATCAGAAGTAGTATTGCGCAATATTACGTTAGTTGTCGAACAGAGTGAGATCTCTCCAGATTAAGCTTATAGACAGCACTGTGCAACCATCAGTAGAGGGCCAGCACATCTCATCAGCTTTGGTCTCGTTACGTGTATATGTAGTCGAAGAATCAGCAGCATCAGACTATCAGTGCCGCCACTTATCATGACTCATAAGTGTTAACAGCAACAAATAAATGTACATGCATGACAGGGCTTCACTCGATGCCAAAAGACAACGCATCAAACTATTCAGTCAGAGTCATGAAACATCCTGAAGTGCCAGATTCAACTCATGTGCAAATTTTGCAATTGATCCTCCTCTT

>Ca2:1602643-1602998

GCTTTATATAGGAGCAAGTAAAGTAATACTTGATACAGAATTCGTAAGCAAAGCAAAGCAAAGTGTGATTGATGCGTTGTTAAGCATAAAAAGATTTGGAGCAGGCAAAGCGAAGCGTAATGGTAATGGTAATGGTAATGCCAGCAGCGAGGGTTGAATTTCCTCCAATCCAACAGCACTGCAACAGCATTTGCTTTTAAGGATCTGTAAAAAGAGTACAAACACACCCCACCGACGAAGAAGATAGTAAAAAGCCGCTTTCACGATCTGTGAGTTGGGACTCTCCCTCAGTAATGATAATGTTTGCTTCGCTTGCTTACGTTCAAATTAAACTGTGAGAGTGGGAAGGTTCAAA

>Ca2:1653063-1653753

GAAATATGTTTAGAAAATTTGTGAGAGGGGGAAAAGAAACTTTCACCTAACATAAGTTTGCAAGTTGCCATCTCCGAAAGTGGATTGTCCAATTGAGACCACGGTATTCCTTCCTTTCACATAAGGAAAGACAACCATGTCTTTGTCTGCCAACATAGCTAGGGAAGCAAAATAAGAAATTAAACTAAATAGTCTATCTTCATTGATCAAAGAAAGACCAAACTTATCAGAGTAGAGTCCGCCATTAGATTTGTTTCACGATGAATATCTCTTACAACAAAATGAGTCAAATAGTGCAAAGTATCAGAAATAAGTGAAACCAAATTGAAACTCACATGAGTTAAGTACAAACATTGATTAATAAACATCCCGTAGCTAACTTCCACAAGAAGCAGCTAAGCCTCTCATGCCCCTTCCACCTTCAAACAAGACTAAAGTGTATCCTTTAATACCATCATAAGTTGATATAGTAGAGAAGTTCCCATTCAGGTTCCCTAGCCCAGATCAATAAATTCATGCATGCACCTCTTATCTAGGGAGCATAGTCAAAGCAAATCTTCCTGTAAAAGACAAGTTTACCAAAAAAAACATCTTTTCCACCTTATCAAGAATGAGTTGGAAAGTATTTTTTGCTGAATATGGAGCATAGGGACCCCAGGATATGCCTTTGAAAAAAAAACTACATTTAAG

>Ca2:1784757-1785030

CTTTTCTACCCTTAGCTAGGCTTGATCTTTTCAAGATCTACTCTTTAGTAATATATTGTCAATTTATGAAAAAGATGTGTGATACCACCATGGAGAGAGAGAGGGAGAGGGAGAGGGAGAGGGAGAGGGAGAGAGAGAGAGAGATTTAGTAATATTAGAACCCTAGCTATATAGTTATAAATAGTTGAAGGACAAGAGGAGAAAAATCCGCAAATACAGTGACAATTAAGAAGAGAGTATGTAGGGAGGCAAAGAGCATTTCTGGTGAAAAAG

>Ca2:1784757-1785053

CTTTTCTACCCTTAGCTAGGCTTGATCTTTTCAAGATCTACTCTTTAGTAATATATTGTCAATTTATGAAAAAGATGTGTGATACCACCATGGAGAGAGAGAGGGAGAGGGAGAGGGAGAGGGAGAGGGAGAGAGAGAGAGAGATTTAGTAATATTAGAACCCTAGCTATATAGTTATAAATAGTTGAAGGACAAGAGGAGAAAAATCCGCAAATACAGTGACAATTAAGAAGAGAGTATGTAGGGAGGCAAAGAGCATTTCTGGTGAAAAAGAATATTGAAAAAGAAAGGAAAAG

>Ca2:1784757-1785062

CTTTTCTACCCTTAGCTAGGCTTGATCTTTTCAAGATCTACTCTTTAGTAATATATTGTCAATTTATGAAAAAGATGTGTGATACCACCATGGAGAGAGAGAGGGAGAGGGAGAGGGAGAGGGAGAGGGAGAGAGAGAGAGAGATTTAGTAATATTAGAACCCTAGCTATATAGTTATAAATAGTTGAAGGACAAGAGGAGAAAAATCCGCAAATACAGTGACAATTAAGAAGAGAGTATGTAGGGAGGCAAAGAGCATTTCTGGTGAAAAAGAATATTGAAAAAGAAAGGAAAAGACAAGTGCC

>Ca2:1979827-1980164

CTAAGAGTACAGAGGCGAGATCTGAAATTTGAAAGGTACGAAAATGAATACAGATTGGATTCAACTACGCTCGCTACTACTTTACACTGTTAAATTGCAAAACCCTAGTTCTATTGCGTTTTTGTTTTTGACGGATCAAAACCCTACTTTGCTATCAAGGCCCATTTCTCAACGGTTTTTGGGAAGCCAAAAACTGCTTTCATTGAAGCCCATTCTGAAAGATTCTTTTTCTTTTTTTCTGTACATGCGTGTTATGCACCACTTAAAGAATGAGATTGTTTTGTACATAGTTTTAGTTTAAAGGTAGTGCAATGAAAGTGTAAGATTGTTATCCAAT

>Ca2:2464593-2464965

GCCCTTAAAAAGTATAACCAATTTTTATAAAAAAAAAAATAAAGTTTTCTATAAAACTAAAAACCAAAAAACCAAAAAATCTTACCTTCACCAAGCAACAAACACCTTTCTTCTTTGTTCTCTCTCTCTCTCTCTCTCTCTCTCTCTCTCTCTCTCTCTGTCACACACACATACACACACACACAGAGACACTCAACCATCCACTCAATATTATTCTAATCTCCATTTTTGAAAAGCTCCTTCTCCAACAACAACCTTACCTCGTCACACCACCATTCTCCAAACTCTCTTGTTTGTTGTGTTTCGTGTTTGTTCTTCTTTTGTCTGCATTTGTCCCTCTTTGGTTCAACCACTTTCCCCAACACACTTTTA

>Ca2:2494491-2494693

GATTAATTAAATATAATTATGATTGACATTAAGAAAATATGTCTTTTAATTTCAACATGGACAGATAGGAAACCTAAAGTGGCCCCAAAAAACAGCATTCACAACAAATTTTTGACTAAAAATTCATCAAAAAAGATAACTAGATTCTTCTATCACACCAGCTTTTGTGGATCCCACCCACCACTGTCCAACTGTCCAAAAA

>Ca2:2494491-2495112

GATTAATTAAATATAATTATGATTGACATTAAGAAAATATGTCTTTTAATTTCAACATGGACAGATAGGAAACCTAAAGTGGCCCCAAAAAACAGCATTCACAACAAATTTTTGACTAAAAATTCATCAAAAAAGATAACTAGATTCTTCTATCACACCAGCTTTTGTGGATCCCACCCACCACTGTCCAACTGTCCAAAAACTAAAAGATATTTAGCATAAAAAACTTTGCATTCACATTCTCCTAAAAAGTATTGTGGAATAATAAAGGAAAAGAGATCAAACAAAACCAAAAGCCTTATAAAGCAAATTCAATTGTCACTCACCATGATCAAGAAGTAGACAACTAGACATATTGATGGCAAGATAACATTGACCACATAGAAAGAGACAAGTCTAACTTTGAAACAGTCTAGGCTCCAAACCCAAATTCAATCAACTGCCTTGGATTTCTCCATTGTAATCATTATGAGTGTAAGTTTGGATGAACTGCGAGTTCGACTAAATCACATTAAGTGATACCACAATTTTGTTGAAGTTCCAAAGTGTTACTTTAACCAAAAATTACGATGGTTCACCATGATCCTGTCAAACTTACTCAAACTTACCGCTAATCCAAAC

>Ca2:2497192-2497601

CTAAATGATTAATGGGTATTTGACCATTTGGGATATTTTTCTTCTGATAAAGTTAAAGTTTTAAACTTTGAATGAAGAAGCAGAAGAAGAAGAAGAAGAGAAGGTGTGTGTGGTGAAGTTGAGTTGAAAGAGAAAAGTGAAAAGTGAAACTTATTCGGTTGGGCTTTATGTTTGTGTTTGTGAGTGATGGATGGATGGATCCAAATTTGCCAAATTTCTTATCTTTGTGGTTAGAAGGAAAGAATCCAAAATCTGTGAAAGTGGGAAGGAAGCAGTTCCCAAAGTGAGAGGTTAAGGTAAGCTAAGAGATGACAAAAACAAAACAACAAACAATTTTTATGCCACTCGCTTTGAAGAGAGAGTGAGTAACAACAAACAATGTTCTTTCTTTCTCAATGTGTCTTTCATT

>Ca2:2690714-2691148

GAAGCTATAATCTTGATAAGATCAATTCATATATATGAACCACCATAAACAAAAATATAAATTAAGAGAGTTTAATACAAACAAGGGATGGAAAATCAAAATACTTATAATAGCATAGCTCAAATTAAGAACATATAAAAAGATAACAAGTTTGCATCACTTTTCATGAATACAACAACATACATAAACCCTAGACATCTCTAGATTCATAAACTTTACTTGAAAAAAAAAAAACTTAACCAACGTGAAGAAAAAGATTAGAAAAAAGAAAGATCCACTAAAGAGCTATAATAATAAAGTAGAGTGAAATTAACTTGCTTGATGTGGCAAATTAATTATGAATCGGACCAAGATGCTTGATTCCCCATAAAAGATCTTGAAAGAGAGTTTGAGAATGAATGAAAGTACATAGTGTACCAATCTTCAGTCAGAAA

>Ca2:2691261-2691513

CTTGTTTTATTGTTTTGGTGATTGTGATGAGTTGTGGTAGTAATGATGGTGCTTTTAGGTTTGTGGGGTGGTTGTGACCTAAAGGGAAATGATGGTGATAGAGAACATAATAGTGAGACATGATGATGATTTTTTGGCGTTGAAACAGAGGCACCTAGCATAAGAGGTTCAAGGATTTTCTTTATTTATCTCTGACATTTTTTGAGATAGAAATAGAGTGCATCCATTTATCAAACCTAACTTTGGTCTACA

>Ca2:2866558-2866810

GGTTAATTCATTACCTAAGACCTTGATTTACAATAATAAAAAAGGTACATTCATGGAGAAGAAAAAAAAAAACTAGAAAAAACATTAAGATCACACTTGTAGCTTTCAAAGCACTTCATGAAATACTATATGTAAAAACAATATCTTATACAAACACTCTAAATTCTTCCACACAAAAAATCAAGATTTCTTCTATGATTTTTCCTCAAAGTTCAAGCTTCATTTGGTGGTTGTCTATATTTCACTGCTCTC

>Ca2:2928579-2928816

ATTGAGAATGGACGAGCCTGAGAGACGAAAGTGAGAGTGAGAGACGAGAGTGAGTTTGAGAGATGAGTGGACGAGAGTGAGAGTGAGGCTGAGAGACCATAGTAAGGGTGAGGCCGCAAGAGTGAGTGCGAGGCGAGGAAAACGGCTCCACGGCGGCGAGAGGGTGAGAATGACAGTGAGACGATTAGGCGGTGAGAGATGGAGAGGGTCGACGGCGAGGGCTAAGCAAAAATTAAT

>Ca2:2928579-2928848

ATTGAGAATGGACGAGCCTGAGAGACGAAAGTGAGAGTGAGAGACGAGAGTGAGTTTGAGAGATGAGTGGACGAGAGTGAGAGTGAGGCTGAGAGACCATAGTAAGGGTGAGGCCGCAAGAGTGAGTGCGAGGCGAGGAAAACGGCTCCACGGCGGCGAGAGGGTGAGAATGACAGTGAGACGATTAGGCGGTGAGAGATGGAGAGGGTCGACGGCGAGGGCTAAGCAAAAATTAATGAAGTGTGGGATATGAAAAAGGGGTGGCCAAG

>Ca2:3308505-3309010

TTGGTGCATTTATTTAGATAGTTGACTATTGACTATCCTTAACAAAAGAGTGATCAAATCAATGTTACATTTGCATTCTCAAGGCAACAAGCCTTCCTAGCTACTAAAGCATAATATATAAAATAATAACAATTTTAAAGAAACCTACAACATTCAACTCTACATAAAAGGGGCAAGCATAGAAGGATTAAATGGGATTTTCCTTGACCAGCAAAATAAAACAAAATTTATAGTCTATATTTGAGCACAAATCACTTTTGGCACCATAACAATGCCCCAATTTTGACTATTTAAGAAATTCAGAAGTTACTCTAGCAATAACACGCCAATCTTCGCCAAGAGCTCACATCGATGAGAAAGTTTGACAACTCAATGTCGGCTTTTTGCCACCTGGAACTGTAGTATGTTTCTTCTCCCCACTATGAATGTCAATATCCTTCATTCCTTTCTAAAGTGCTATTTTTTTCTCCTTTTTTCGAACTTGGTCTCTAATCCACAATTTTGG

>Ca2:3308505-3309081

TTGGTGCATTTATTTAGATAGTTGACTATTGACTATCCTTAACAAAAGAGTGATCAAATCAATGTTACATTTGCATTCTCAAGGCAACAAGCCTTCCTAGCTACTAAAGCATAATATATAAAATAATAACAATTTTAAAGAAACCTACAACATTCAACTCTACATAAAAGGGGCAAGCATAGAAGGATTAAATGGGATTTTCCTTGACCAGCAAAATAAAACAAAATTTATAGTCTATATTTGAGCACAAATCACTTTTGGCACCATAACAATGCCCCAATTTTGACTATTTAAGAAATTCAGAAGTTACTCTAGCAATAACACGCCAATCTTCGCCAAGAGCTCACATCGATGAGAAAGTTTGACAACTCAATGTCGGCTTTTTGCCACCTGGAACTGTAGTATGTTTCTTCTCCCCACTATGAATGTCAATATCCTTCATTCCTTTCTAAAGTGCTATTTTTTTCTCCTTTTTTCGAACTTGGTCTCTAATCCACAATTTTGGCTACAAAAGTACAATGAGTTTGTGAAATAGCTGGTATATAGTGTTATATTGCAGCGGTAGTTGATTATGAC

>Ca2:3312219-3312468

GTGTAAACAAGTAGAAAAGAGATGAAATTGAGCCTTTTGCCATTGAAATTGAAGAACGAAATTGAAGAAGAAGAAGAAGAAGAAGAAAAATAGGGTTTATTCAAAAGGGAGAAATTGGAGAAGAAAAAAACATGGTTTTTTAGTTGAAATTTGAAAATTTTGAAGAATGGAATTGAAGAAGAAAAAGATGGGGTTTAGGTGAATTGAAAATTTATTGGTGAAGGAGAGTGAAGGGAGGGAAAAAAGTTT

>Ca2:3992649-3993385

CATTAATGAACAAAACGTGATATACCTATTTTCTTTCAACCCAAATTTCGACTTGACTTGTTTTTTTAGATTTACCTAAACAAGAAAAACAGAACACAAGACCTATTTATATGGTTCACAATGATATTAGGAAAAGAGTCATATTGGGAAATATTAGGAAACAAATTTCACAATGATATTGTAAATCAAAGTCATTCATATTGTTGGATCTGAGCAAGTAGCATCAAGTCAGCTGAAAAAACCAATGAAAACATGGGTCGGTTGGAGGTAAAGACTGATGGCAGAAAGCCAAAATACTGCCATTTTATAAACATAACGTTGTGGTCTATGGCAGTGCTTTGGCGGTCCTTTCTTCACAAATTGCCTATGATGACCGGTAAAATAAATCCAAACCGTTCTGCAATAGAGGGACCAAGGCAACACTGAATGTATTCATGGAGCCATTTGCATTATGAGGTGGATAGTTCAGAGATACATTGAAGAAGGAATCAGTACGAAGGTTCTTGAGGACTAACAAAGCATGAAGGAATTTTCCCTATGAGACATGTTTGATCCGAACTCAAGTGATACTAAGTTGCAAGAGGGTAATTGGATTCCTGTGTGGTGATTCAAAGCATTTGAAGAAGAAGGATAAAGCATAAAAGTGCATGAACTCAGAATTGTACTGAAATGAGTCCAATATTCAACATAGTAACATTAACATACTAGAACCCACGAAATAGATCACATAAGACCC

>Ca2:4018790-4019673

GTCTCGTTAAATATTTTCCAATATATTGATGGTGGAAGTATACGTAAGCGTGGAAATTAGAAAACTTAAATAAAAAATATTAGTAAACGCAAAAGTTAACATCAACAAACTGTATATGAACACGGTTTTCGTTTTTATAATATTTATCTCTCTCTTTTGATTGTATTCTTTTTCAGTTTAGATTATGTACACACATGTTCAGCGTATAAAGTCTATTTATAGCCTTACAATTGTATTGTGTAGGAATCAGTAACATGTGTCGATTATTTAAAAGTTTGTGACCAAATATTGAGATGGTGACACATTTTTAATTTAAAAAAAACAGTGGAAAATACGACAAAAATAATAATAAACACGATAAAAAAACTATGAACATGTTAGAGACTTTGGACTGGAGGAATTTTAGGAAACGAATTTTAGAATACTAGTTGACGAGTGATCCTAACATTGATTCAAGATCTAAGTTAGTAAAGTAAGGACTCTTGATTTTATTTTTTTTATGCTTTTTTTAATGTATATTTAGATCTTAAAAATATAAAAATATTGAAGTATAACAAGTCAAAGCTCTAAGAAATGTAAAATATACAACATATTAAATTAATATATGTTACATCTCAAAATTCAGAAAATAAAAATTTAAATAAAAGTTAGAAAAATATCATTATACATATTAAAAAGACCTTTATTTTATTATTATGTTTTATCAAAATAATGTTTTAAAATTTATTTTAGACCTTAAAAATTATTGAAAACAACAAATTTTACAAGGATTCAAGTCCTTGAGAAAAGTGTTGAACAAAATGATCCATTTGTTGCTTGTTCAAAGCCAATCCAATCTCAATTCCACCATCATTATTCTTATTTTCCGAAAGAGAAAAAGCTC

>Ca2:4326519-4326748

CGTTGCTTGTTTGAGAAGATGAATGAGAGTGGAGTTATGGATTATAGAGATCTGAAGGTAGGAGGAGGTGTGGCGTAGGTTGTTGATTGGTTTGGTGGTGGAATTGAAGTAAACCCTAGATCAAGATTAGGGATCTATCTTGTTCTAACAGTCAAAGAAAAACCAAAATGGAGAGAAAATAAAACGATTAGTAAAAGTAAGAAAGTGATGACGTGTCATCATATGTATT

>Ca2:4544355-4544762

ACAGAATTTACAAGCAGCGGAATAGTTTTTGAAATACGAATCCAAAGTATTTACACTTCAAAAAGTGGTACATGGAACCCATCAATAATAAAATGTCAAGCTAACAATATTGGTATAGGTACAGTCTTCCAAATTAAAATGAACACATCCCAGAAGAAAGACATCTAGTCTCTATACGTCAACCTAAACCGATCATTCTACAAAAACTATACATCCCGAGTGATCTTCACGCGCCCCGCAAGATCCTCCTAACATAGCTCCAGTCAGGTGTGTCCAACTACATTCCCATCCACAGGGTACTAACCGGTAGGATGGTCCTGGTTCTCATCTGAGGGCAAAGCCCAGATTTCCACAATAGTTGTAAAGGGTCACCAACCGAAATTAACAATTAACACATAACATTTAAG

>Ca2:4637360-4637562

CTCCTGGATTTAAGGAACAACCTCAGTGAAGAAAGAGCAAGACTTGAATCGAATCTTCTGCAAGATCTGAAGGACAACAACATATACAGCAAAATGCACTGTACGGTGTTTTTTTGACTTTTGTTGTGTTGTGATGAATGGAAATGAGAAAGCTAAGTAGAAAATATCAAAGCTTTTTGGAGTTTTTATAAAGAAAAAAAAA

>Ca2:4852217-4852551

CCCAAATAAAGTTAAACATTGTATTTGTTCGATCCCAATGGGGCCAAGAATTCAACAAAACAAGTCAACAAATTACACATGAACTTTTAATTATCCTCAACTACAGAAGGAGTCGACTGAACAATCTTCCCATCCACAACGATCTTCGTCTAATCCAACCTATCCAAGTTGAGAGCAACCGATGATTCAAGGCACCTACTTGGGCGATGGAATTTTCGAAAGAACCCTTGACAACTTTTAGAACATCTTTTTCCTTGGTATCAATCTATTTATTTGTTGTTGTCTTCCTCTAATTTCAACATATCCACTTCCTCTTTTAGAAACAAGATTTCCT

>Ca2:5207388-5207694

GGCTAATATAAAAATTATATTTCACTACTTAAATAAAATAGTAAGAGCTAAAATGAATATATTATATGAGTAGAAATCGAGTTACTCTATCTAAGAGTACCTTTGAGAGACTTTATGACACAATTTTGGCTAATTGCGATGATTCATAAAAAGGTTGAAAGAATTTTTGGATTGAAAAAATATCCTCAAATTGTTGTTTTTGCCTTCACCTCCAAATTTAGCCACTGGTAAGCTCAAAATAAGACGGAAAAAGTTGACTTTCTTGCTCACCATTTCTCCAGTTTTGATGATACGATAAGCACATCA

>Ca2:5207388-5208001

GGCTAATATAAAAATTATATTTCACTACTTAAATAAAATAGTAAGAGCTAAAATGAATATATTATATGAGTAGAAATCGAGTTACTCTATCTAAGAGTACCTTTGAGAGACTTTATGACACAATTTTGGCTAATTGCGATGATTCATAAAAAGGTTGAAAGAATTTTTGGATTGAAAAAATATCCTCAAATTGTTGTTTTTGCCTTCACCTCCAAATTTAGCCACTGGTAAGCTCAAAATAAGACGGAAAAAGTTGACTTTCTTGCTCACCATTTCTCCAGTTTTGATGATACGATAAGCACATCACTGTTGCATCAAGACCATTACTCAATTATTTAATGACTTCAAAGAAACTGTTGCAGCCTTACATATCTGAATCCTAAGCTCTTGCAACGATGGTGAATAGTGATACTACCATTACATAAATTATGATGTTCAACCACATGTTGCTTCTTCTAACCTAGAGTTAGAGTAGGAGTTTAACATTCAGCTCCATGGTCAAACCTCTCCAATCTTCAAGTTTTCATATTCATTTCATATTTATGAATAAGATTCACGTGTGCAAATATTTTCACATTATATTAATTAGTAGCTTAGACATATCTGTCAGCCA

>Ca2:5413905-5414409

CTCCCATTTAAATAATTTAAATAAATAGTAACATAAAACAAGTTAGGCACAAGCATTTTAATCCCTAGTTTACTCTCTACTCACTCTTCTCTATGTATATATACAATTATCATATATATAAGCTAAGTCCTTATTATGCAATATAATACAATTAATTAACATAACTATTTTGTTAGTCGCAAAGTCTTATAGCAACAATAATAGTTACACACTTTTAACTAAACCATAGTCACATAGACATATAGCGTTCAGCAATAGCAATAGAAGTACCTTTTTCAGTTTCATTTCATCACTTTGCATGTTATTTTAGCTCCCAATTCCATGTAATTACTTTTTCAACCCTCCGTTCTCCCAAAAATGACCAATCAAAGTGAGAATGAAGAAACCATACTCCAACTTTCCAATTTCCAAACCTGACTTCCTTCCTTAATTCTCCACTCATTTGCTCCATGACACATATTTTCTTCGCGATATATATATAATACATTTAAAGATTTAGATCTT

>Ca2:5566559-5566840

CTGGACGAGTTCCTAACTGTTTCAGGCGGCCGACGATCAACTCCGGCTTCGTTTCCCGGCGAGATTCACGGGCAGTTGAGTTCAGTTCATTTCTCCGATCCAACTTGTGTTTGTGATACAACACGGGCAGAGCGGCACACTGAACAGCTAAACCAAACCGCACCACACCGAAAAAATCTAACAGTGATGGGCAGTTCGCGAACTGGGTTCATGAACTATAACAGTTTGGTTCTTTTCGGTGCAGCTCAATTTAACAGTTCAGTGTGGGTTTTGGTTCTTTT

>Ca2:7408485-7408897

CTTCTTCCTTACCACACTCCTCTAGACACCATAACAACCATTGCAACATCAAACAACTTTCTACTATCTCATCAAAGCAAAACTTGTAAATAGAAATAAAGTACTTGTGAGGGGAAGAAATTAGTCCATAGAAGATGAAGAGAGGAAAAGGGAACTTCTTTTGAAACCTCAATCTCCAATCGACCGTGCCTCGAAGAATTCGGCGACCCCCACCTTCGACGCCTTCAATGCGAATGTCTATTGTGGGACTCCTAGTCAGTTGCGCACCACTGCCTTCATCGCCCTTCGCCACCAAACATTTATTGAGACCCGTCACAGGGAAACGCTCAAAGCATCGTTAGCCACCACCTATGGGCGGTCATGTACTCTCTCTCTCTTCTTCATTTCCTTTCTATTTCTTTCATTTTTTTCT

>Ca2:7756343-7756754

CTTGAAAAAGGGTCTCAAAGGGCTTGAAGTTTATAGAAGAAGAATTTTCACAAATGATATTATAGAAATGTCACATATACAATGATTTAAGATTAAGATCAATAATAATATGCAAACTCAAGTTTTTCTTTCTTTCTAAGTCACGGGTATGAAAAATGTACTTTTTTTAATTCCCCCACCACAACAACATTTTGATCACTTTTGGAATTAGAAATATGATTCTTTGAACTACAAGGGATATGAAATTATATCATCAACTTTCCCTATTCAATTATCTTTTCTTTTTCCTTGTGAAGTGTTTCATTGAATCATGATGCAATAAAAAAGCTCTATTTGCAATAGGGTCCATGCAAAGGGTCCCCTACTACCCACACTTGTGATGGAATACATTGGGGTGGGTGTCAATTTACT

>Ca2:8536054-8536373

CAAACAAAGGACAGTCACTAAAATATAGAAACAATGAGAACCAATGTACAGATTATGATTCTTGTATATTTAATCTGTTTACATTATACCCTTTACAAATCACACTTGTATAATAGTATAATAATGTAAAAAACAAATCACATAGTACAATAATTGTATAATAATGTAAAAATCTATACATTATTATAGGGATACCAAATAAGTAATAAAAATGTACATAATTTGGTCCCTATACTTGGAACAAGTTTTGTCACAATGTGGTTGATGACCCAATAGAAGTCAAGCCAGCAATTTGTTCCTTATTTCTCCTCAGCACAAG

>Ca2:9499165-9499466

GTTGCATTAACAATAATATACCATTCAAATCAAATAAGAAAAAAATGAATGAATACAATGAGAAATAAAAATAATCACAGAATTTTCTCAAAAGTATCAATACCCTCTGGTTCTACTCCTAACTAAGCCTAATAACCTATCATTCTCTATCTCATTTACAGACATACTTAATAATGGAATTTCGTTTGTGCCACGGCCAGTTCAAAGTCTTCAAAAAATACCAGACCTTCTGTCACATGGGTTCTAGTCTGTCCTTGAATCTCCCCCTTCACTTCCAAGTATTTCCCTTTCGACAAGCAGA

>Ca2:9834438-9834844

AGGTAAAGAGTTGTTGAAGACAAATGGTAATAAGTGCCCTAGAGCATTGTTTAACATGTCTAAAAAACAGTTGTTGGAAAATTATGAATCTAATATATTTTTTACAATGTATTTAATTTGTTGAAAAGAAACAAAGCTGATATGGAAAATTAGAAATACTTACACACAAATTAAATAAGAAAAGATGAAATTGAAGGCACATAGTTTCTAGCTGAATAAATCACTGTGGCATAGGCTATGTCCTTCAATGTTCTTCTATCACCTTAGTAAAAAGCACAAAAACATTTCAGCTGCTTCTGTATCACAAAAACACCCTCCTAATTGTTCTCGCGGCAGCACGGGATATTAACTGTCGAACCAAGTTAGCTGGCAATTGAAGTAATTGCTGCTGGGACTCTGGTGGGAG

>Ca2:9989894-9991126

GAGAATATCAGAAGTTACAATCAATAAAATGAGAAACGCTTGAATCATTTACACTTAGCATATCCTCTATTTATACACTCTTAAACAATAAATATAATAATTTGCAAAGGAAATTAATGATATTACTACTACCAAAGTGCATGAGTGATAGTTCCAAGGTATATGACTATGTAGGTGAATCTAATTGCTGCAACTCTCAACATTAACAATAATTCAAAGACTCCCCCTCAAGATTGAGTATACAAATTGAATGCTCCAAGCTTGTAGGAAATACATTGAATTCTTGGCACCTTTATAAATTTTGTAAAGATATCGGCTAGTAGATCATTGGAGCTAATAGATTCAAGTTATGTTTGGATATTTTAAAATGAGCAGAATAGAGCATAGCATAATGAAGTGGAGCATAATGGAGCAAAATATTCACTCCATTGTTTGGATATTTTAGAATGCAATAAATATTCCATCACATTCCTCCAAACCGGAGGGGAACAAAATGAGAGTAAGTGACCGAATAAGATGTAACACATTCCATTGTATTCCACTCCGTTCCATTCTCTTTTTGCCAATCTAAACAATGAAACATAAACTTATTCCATTCCATCTAATACCACTAATCCAAACATAGCCTGAGTGATGGCTTCTTTGGAGATAACTTTTTCTCAAACAAAATGATAATAATTTTTTACTTCTCTCATGAAATAATGGATTGGAGCCTATATGTAAAGCTGCTTGATTATAACAACATAATTTCATCTCTTGAGTATTCCAAACATTCAAATAGATCCTCAATATCTCTTATGTTAATCATTATAAAGACAAAATTAACCAACAAAAAACACATATTATGATTAAACTGACCAACTAAAGTAAAATTTGATGATCAAAATGACTAACAAAATATATTAGTATATTTAATTTTGATAAATTCAATGATTTTTTGTAACAGTGCCACACATTAAAAGACCAAAATAACCATTTACTCAAAAAAAATGCAGAGCCCTCAGATCTCATTGTTAAGTATAAATCATCTGCAGCTTACAACACTGGTTTCATTAACAGCCTTTGTGCACCATTTGAATAAATAAATGAATAAATAAATAATAAATATGTCCATTTATATATATAAATGGATAGAGAGAGAGTGAGAGAGAGAGAGAGAGGAAATTGTGAAGACAATGGTGCAGGTTAGGCTATGAACAAATCCATGTAGTGATGTAAGCACTCAATAGTCT

>Ca2:10218242-10218750

CTGGCATGTTTCTTTGATATTATTGCTATGATCTAAAAAAAAAAACATTCTCTTATGAAGCTGTCAAAAAAATAATTATTTCTCTAAAATAAACATTTTAATATGCAAATGGTCTAAGACAAGTAGACAACGTGCTTATGTTGTTATGTGTACAATAAGTTACTATTATACTAATGAATTAGATTGGCCAGGTATCAATATAAAAACTGCTGAAATACTCTTCGAACTTAAATATAAGCAAAAACAGTACACGAATTTGGTCGGAAAACATGTACCTTTTTACTTATATTTAAGTTCAGCATGGATAAAATATACATGAGAACAATTTTAGCTTCATACTCCTAATGAAGAACTCAGCTGATTGTTCCAAGATTTATTACCGCTGATATCAAGAATAAACAAACACCAGAGGCTACTTGCAAATCTTTGAAACTTAAGTAAGAGTTTGATGTTTCACATATTTTGCAAGGGTAGAATCCAGCTTCTCTCCGATATAAGTAATCCATGC

>Ca2:10877164-10877730

CGGAAAAATTTCTCAAAATATGAATCCAAAATATTTACACATCAAAAAGTGGTACATGTAACCCACCGAAAATAACATGCCAAGCTGACAATATTAGTAAATGTACAGTTTTCCAGTTTCAAAATAACTTAATCCAAAAAAAAAGACATAAGTTCCCTCTATGTCATTCTAATCTGATCATTCTTCAAAAAACTATAGCTATACACCCTGAGTAATCTCCATGCGCCCCGTGAGATCCTCCTAACATAGCTCAAGTCAAGCATTCCCATCTACATTCCCGTCCGTAGGGTACGAACCGGTAGGATCGTCATGGCACTCATCTGAGGGCAAAGCCCAGATTTCCACAATAGTTGTAAAGGGTCACCGACCGAAATTAACAGTTAACACATAACATTTAAGTTTTTAAATGCACAAAATAACCTTTTAACTAAGCACGCACCTTAAAAGGATTTCCATATGCTAAAAGTTCATGTAACACTTGCCAAATAAAAATGAAATCAAAATAAGGTTCTCAATTCAACAAGTAATACATAACAAATCAAAACATTGATAAGTCAATTGAGCAA

>Ca2:10877164-10877484

CGGAAAAATTTCTCAAAATATGAATCCAAAATATTTACACATCAAAAAGTGGTACATGTAACCCACCGAAAATAACATGCCAAGCTGACAATATTAGTAAATGTACAGTTTTCCAGTTTCAAAATAACTTAATCCAAAAAAAAAGACATAAGTTCCCTCTATGTCATTCTAATCTGATCATTCTTCAAAAAACTATAGCTATACACCCTGAGTAATCTCCATGCGCCCCGTGAGATCCTCCTAACATAGCTCAAGTCAAGCATTCCCATCTACATTCCCGTCCGTAGGGTACGAACCGGTAGGATCGTCATGGCACTCAT

>Ca2:11639688-11639979

GCAATATTATTTTTTTATTTATAAAAAGTTATATTTCTTAAATCTAAAATATGACTTTTACATAATACCATTAAAATTGAGTTTTTAAACAAGTTCCTACAAATTTAAATTTGAATATTACATGATGTGTTTTATATTCATTAATGGTGCTCCATTTCTTCCAAAAGTATGTATCATCCTCCATTTGTACTAACACATATAATGGTGCTCCAATGGATACAACTAGAAGTCTATGTTGAATCGAAAGACATATGAAGTCTTTATTGTTGTCTAAATTGCAAGGAATCCAAT

>Ca2:11686910-11687167

CTCCCTACTAATTGTAATTTTACACTGATATAAGTCAAAACTTATCACATCGTCAGATATACTCTCAAGTTGAAAAGCAAATAATGCACACAAATGTGTATGACTATAAAATTAAGAAAATAATAACAAGTATTCTTAGAGCACTTGTTAAATAATAAAAAAAAAAAATATAACTTTAAAAATTGTGCCATACTCAATGCATTAGATACACTGATGTCTATCTTCGGTCCATAGAATGCACCACCCCCTTCATTCAA

>Ca2:11758410-11758808

CCAATAGTAAAAAAGATCCATAGAAAGTGAAAAAAAATGCATTTTAATGAATGTTGTTGCATGCCACGCGCCCCAAGCGCAGCTTGGCGCTTCTGAAAATGTGCCTCAGGTGCGACTTTTGCGCTTCAAGCACCCATCTTCTTCTATTTGACGTTTTCTGCATTGTTCTCCTCGTTTGAGTATGAATTGAGTTCTGGTGTCTTCATGAAAGTTGTAGTTATGGATCTGATCGTTCACTTGCACTTGGTTTGTCTTCAATTTGACATATACAACTCCAGATATGTCTGAAATACTCTATATATATCATGTTGATTTCTAGCCAAAATTCAGCACTGCATTAAAACAAAGTAACAACGCAAAACTTCGAAAAATCTTTACTTAATCAAGGAAATAAGAAC

>Ca2:11882572-11883036

ATACAAATTAAAGATGGATGCTTTGGATATGGTTAAAGAGTTCTTCACTCCAGCTAAACTTTCCAAAGGAATGGATGAATCTTTTTTGCTTTGAGCTCGAAGGTTGATCAACCCTTCGTTGAGAAGTATAGGCCTACTTTGTAGATTTGCAAAACAGAGGAAGGATAAATGCTTACTATATATGAAAAGTTCACCGGATATAGACTTATGACTCAATTGGATGTTTTTAGATTACATGATGTATATTATTGGATTCTTGAACTGTGGAGGAATTGGATTAGATTTTGTGTTTTCCCTTGTTCGATGTCGATTCAAGTAATTATTGCTCTGTTCCTTGACCTAATTATTGCTGAAGGTTTTGCTGGATTTACTAAAGACGTAGATGGAGGGCTCCTAAGAGGACATGAAGTTCATCTAGGGATATCCCTTCAACCACTAAAAAAGATATTGCCACATTTATTTAG

>Ca2:12602092-12602362

CGAATGGGACGACGAAGCGGGTAACAGATGTCCGGGACGAGACACGACTACTTCTTCAAACGATAGAAGACCACTGGAAGAGACCCGAGATCAGATGAAATTTCAGCACAATAATAAAGGAAGCTAACTTACCCTTGAGAGAGAGCGATCATCAAGACAACATCTAGAACGGTCTGAGTCAGACGGAGCAAGATACAATCTGGAGGAGGTTGGAAGAAGAAGAAGGGTTTAGGGTTCTATAGGTTGCAATCTGAATGTAAGTTCTTTTGG

>Ca2:13863941-13864206

CCAGGCAATGGCAGCATGTTTCTAATCCTATGGCAATTTTGTCCTTGAATCTTTATGTTTGAAGGTCCTATTCATCTTTAAAAAGCATTATCAATTTTCATTCCTGGAGATGTGTTCATGTGAATGCAAAAATTGTTTCTTGCTTCCGGACATTGTTTGAGCGTAGCCAATCTGATCAACCCCACATTTGACATCTAAAATCATTTATAAACAATGAGCCATAATTTCAGCAAACATAGATTTGGAAGTTTGTTTTTTAGGAAAC

>Ca2:13940861-13941242

CCATTTCCTATTAAATCCATATATATCTTTCAACTGAGTTACCTTTATATGAAATCCAGTTACAACCAATGAAAACCAGTTATCAAAAGATTCTTATACATGAGAAGTTACTTACTATCCATCTCCAACTTCCTATTCCTTACGAATACAACAGTATCAAGTGTAGACAATGTTTAATTAGTACGTAAGGCTCAGAATCAAGTAAACTATTACTTAGGAAATCTAGGATGCCAATAAATATACATCTGAACAAATGCTAGATCTGTTAATAACTCTGATCAAATTGAGTACTTCCTCCATATAGGACTGACATACAAATTCACAACATCATAGCATTCACATGTCATCACACCATCATGTCCCTATATCAAAACCTTGTTC

>Ca2:14097472-14097956

CACTCCCTCAAGTACAACACCAGGAACAAGCACCGGAACAGGTATTGGAACAGGAACAGGAACAGGAACAGGTACAGGAACAGGTACAGGTACAGGTACAGGAACTGGCACAACAACAGGTGGTCCTAATGTGTTTGGAATTAGTCCAGCATCATCAACTGGTACTGGTGGATTAAGTGATTCTAATGGAGTGGTTCAATCTAAAGGCACTTGTGTGTTATTACTATCCCTTTTTTTTACTTTAAGGCTACTAATTGTGAGGGACTAAATGGTAAAATTGTAGGCAAAACTTGTGGGCGGTTTGTTGTGTGGATGAGATTTTGCCACTACATGATGGGTGTTCTTGTAATTTCATCACTTGGAATCATCCAGAAGCAAACCCCACTTTGGTCTTCTGTTCTTTTTGCTTTTGTTTTGTTCCTTCTATGTGCAAAGGGGAAAAAGTAGAGAATTTGGTTCCTTTTAAGTGGTGAATATAATGATG

>Ca2:15396079-15396365

GGATCAAATATAATGCCATGTAAATATATCAGTCAGTATACACAGCCAGTACATGCATCAATACCAAAAATAACACTCAAAATCAATTGTCTACAGCTAAACTATGTCTAGAGAGAAGCTTCCCCTCCCAACCAAACCTCACATTATTAGACCATAACTTGCTAACAAGTCATGAAGCAGGCACATAGAAGGATGGAAAGTATGACTCCTGCCAATCGACCTACTTGGATCTCTGTCTCATCGTTCGGCGCTGCCTCTTCAACCTCCTCATACGCTTCTTCTTCCA

>Ca2:16067534-16067759

CTGCATAATTTTGTTGGCGAAGCATGTTGCAGCTTTTTAAATGAGGCAATGGTATCAGATCTTTTAGGTTGTGTTTTTGTCCAAGTCTTAGTCCATTAAAGCTTACATGGATTTGAGTGGGTGGCATGTGGTCTACGTTTCATACTTTTTGTAATGGTGGTGTTCTGTAGTGTCGGTGTTTTCTGTTTTTATTAGTGGCATGTTTTTCTTAAAGGCAAATTATAT

>Ca2:16665904-16666174

ACCGTTGGTGATAAAGGAGAAGACAATGTTCCGATTGGTCGTTGAACTCCCTTTTATCTGAATTGCCACTTTGACTCTTTTCATTTGTTGGTTAGTATCCTCTGCTGCTTTGATTGAAACCGGTTAGTAGTTACAAATTGAGACATGCAAGGTTTAAAAATGGGTTTAAGGAGCTTTGTCAAGCTTTTGAGTTTGATTCATTTAAAAAAAATGAAGGAAAATGAGAGGTTGAAGATTGGGAGGATGGGAGGATGGATGCAGGTAATGAAG

>Ca2:16831453-16831693

CACAATTCTATGTTCAAATGAGTGTTCACACGATAGAGCATCTATAGCTGTAGATTCACCAATTTAGGGTTCCGACCTTCAATTTACGGTTAAGAGGAAACATGAATCATGATTGAAGAGGAAAATCAAAATTTGCCGTGAAGAAGAGGAAAATCGATTTTGTATTGAAAAAGAGGAAATTGATTTTGTTAGAGAAGAGGAAATTGATTCTGTTGTAGCTGTAGCTAAAAGTAGTATTTA

>Ca2:16831453-16831726

CACAATTCTATGTTCAAATGAGTGTTCACACGATAGAGCATCTATAGCTGTAGATTCACCAATTTAGGGTTCCGACCTTCAATTTACGGTTAAGAGGAAACATGAATCATGATTGAAGAGGAAAATCAAAATTTGCCGTGAAGAAGAGGAAAATCGATTTTGTATTGAAAAAGAGGAAATTGATTTTGTTAGAGAAGAGGAAATTGATTCTGTTGTAGCTGTAGCTAAAAGTAGTATTTAGTGACAAACAAAAAGGGCTTCACAAATTCAGAG

>Ca2:18420854-18421337

ACCAGAGACCCAAAACTAATGATACAAGTGAACAAAGCAGATTATCACACCAAGTTATAATCAACCAAGTCCAAGAGTATCAATTTTGATATAGGTGCTCAAAAGCCTTGTGTTCTGTTATCTATACTGATTTTTGTTTTAGGTTTCAGCCTTTCAGGAGAAAGGAATGGCTAAACTATAAGGCAAGGACTCAGAGCATCCCCGAACTCGGATTTAGGCAATTAATTAAATATTCACAGAATGGTGAAGCATGTAAGATGAAAGAAACATCCAAAACTGTGATATATCTTTCAATTAATAATGGATGTCATCTCATTCACATAATATATAGAAGAGTGTAAAACAAAATGGTCCAGAGAAGAATAGTATTGTGGCAAGCATGAACCTTAAGCAGTACTAGCTTTAGCAGGATGGTGAAGAAAGATAATCCATTCCAATATAAGCAATTATGAGAATAAGATGTTTCCAAACTTCAGAAAGATT

>Ca2:19465300-19465678

AGTGTTAGAAATATAATATAAAATCATTCATGTCTCTTAACTTGCAATGTAAATGCGAAGGTCCAAAAATAATCGGTTGAAATTGAAATTCAACTTCTTAATTACAAGAAAGGGGTGTTATTACAACTTATGAGGATAAAATGAGAAAAAATAATATTAAACAGGTACTGCAGCACTAGAAATATACTGAAATATTGACAGATCACAGATCCACATGCAACTTCATATATGTTCAACCTTCTAACACAATAATCACATAGCAAGTCAATGATTGCAGCAAGGAAAATGACTTACAGGGAAAATTTCTAAATGTGCAGGCCAAGTAGATAGTTTTTTCTTCCTAGGGCGAAGAGTTTTTACCACACGATGGCAGCAGTT

>Ca2:19466087-19466386

CTTGAGAGTGAGACAGCGACCATAGATGAGAGCGCGGGTTCGAGAAGGGGACGGTTCGAGAGGGGTTCGATGACGGTGACACTGAACAGCGACGAGTTCGAGAGGGGGACGGTGACGAGTCTGAGAGGGGGACGGTGACGAATCTGAGAGGGGGACGGTGACGAGTCTGAGAGGAGGACGATGATGACTCTGATCGTTCAAGAGGGGGACGACAACGATGAGAAACAGTGAGAGAAGGGTTCAAAACATAGGAGAAGGGTTCAAAACAAAAGAAGGGTTTAGGCACAATCTGATTTTAC

>Ca2:22904786-22905253

TGGACATCCAAAAATGTTGAACACAAAAATTTCATTATTCCAAAATACCTTGTTGGTTGCATAACTATCATGGCACTACACCAGTAAAAGTATAAGCAAAATAAATTTGGAACAACAATGAGAAAAACAAAGTCTTAGGCATTCTTGCACGTGAGGTTGAGACTTGGGTTTCACTAGCTTGTCGACTTCAACTATGAGCAACCAATTAATGCCCTTTGTATCAACCTTCATCGCAAGATGGAGAAGGCGAATTTGCTCCACCATGTTTTCAAACTATTAAGCCCCAATATTGATGATATCATCTTTGCGAAGGTCGATCAAAGCTTTATCGGTGGCAGGAAAGTTCTTCAACTCCCTAAGCTCATTATTCAACTTAGATCGATATTGGTTCTTGGCTTTGAGCTCCTTGACAAGCTACCTATTATTTTCATAAAATTTTCTTGGAGAATAGTCAAAACCATTTTCTC

>Ca2:22987926-22988572

GGCCTCAAAGATTCCTCAACAAGACTCAACAATGGTGGCCTCAAAGTAGAGCAAAAGATGACTCTTTGGCACCCCAACTTGTTCAGCACACCAACTCAATGAATCCAAAAAGATGCTTCTAAAAACCTAAATCTCAGGCCAAGGGATCCACAAACTTGAATTTTCTCACGAAATAGATATATTAATGAAACCAATGCTCTGAATTAGTTAAGCAATTAAGCTTACATATGCAATACAAGCAAGCAACCAAAACATAAGAAACTAAGTAAAAAAATTTAAACACATAGGAATAAAACATTGACAAACACTTGGTATATGCCTTATCCAATGTGTTCCCAATTTTAGTTTTAAAAATAATAAGTTTACAAAGCACTTAACTATTGCAAAAATACTCATCATGCATGGACTATAGTTGCTGTGAACTTTGTCTGATTTCACAAAGACAATAGAAAGACTTGAGCTGTGGAAATATTGAAGGAAGAAATTAAGTAACAAAACCCCCCTCCGATTAAGAAGGTATGCTCACTCACTTCCACCTATCTTCTGAGTATTTTCATTATTCCTCATCAGTGACTTAAGCCTAAGAGAGGGACTTCAGAACTTATTCTCACTGCAAAGCGAGTCCCATCAAAAGCCAACATAAGCC

>Ca2:25828101-25828716

CTCCTTAATATTAATCATAACAAACAAGCAGTTTTTTTGAAGTTGTTCTGCAATATCAAATATTTGGGATGCAGTCTCAAATGAGTATACAGAACCAAAACTAGAGCCAATGGTAGAAGGAGCAGAAGCAATAATATTAGACGATGGCCTCCCAAAATTCTTCTTATGTGCTCTCCCCAATTTCGGATAATGTGTTTTCCAATGTGCTTGAAGAGAATCATCCTTAGCACTAGCCATAACAAATAATAACAGGAAAATACGACAAACACAATCACTGAGACAAAATAAATTAGGGATAATCTGGTGTTGTGCGAGCCCGATTTCTCCCCCTACAGACGTTTTTTCGCCGATCCGACAGTTGAAGACGAAGACCTGAATGTTGCGAACTTGCTGTCCAAAAATCAGCTCGATCCAACGGTTAACGAATGCGCAATCAATGTTTTTGTGAGATTGGTTTAACAAAATCGAGAACGGGGTTACTCTTCTCTTTTCTCTTTTGGCGGTTGCCTTTCCTATCAAAATAATATTTCTCTTCTTTATAGTAGATCCCAAAATCAACCAAAGCTCTTTGATACCATGTTAACAATGAGAAGAGGAAGAAGAGAGACAACCACT

>Ca2:27582616-27583011

CTCGCAGCAAAGTATACTGTAAATACATAAATAGAAGAAAAAAAACATTGCAACCTAAAACAAAAACATATCTCCAAGACCAGAAAATCTAGATGATGTAGCCAGTAGCAAACAAATGAATTTAAGCACTCTTGAAAATCTGTCAAGAAAATAACTTTTTGATGGCCTTACAAGTTAGCATACAAATAGCCCTTTAAAAATCTAGGTCGTTGAAAACTTTAACTATTAAATCTAATTGCTATAAAGGACATCGGTCTACAATGACCAAATTTAGCATTGGTCAATGTTAAATTCAACCTTTATCTAAATGCCATGTAACTTACATTAAACAACCAAGGTGTTGAAAATCATTCATGCACTTAGCTATTGTTGTAGTCTCATCAACAGTACTTGCC

>Ca2:27768730-27768970

ATCTTTGGTAACATGTAGATAAGTAAAAAGGACAAAAACTATCAATAAAATCCATCATAGCTGAAACGGCTAAAGCTCATATATCACAACCAAAGGGCAAAAGCTAGTTTCAACAACGTCACATTATTGGCTGGCTAAACAAGATTAACGACAACAAACTTGAAGATTATCCAATCCATTCCCACATGACCTCAGTACCTGTGGTGCAGCTTCTCTTGTTCCTTGAAGTGCATAGCATAC

>Ca2:28100228-28100490

CTTGAATGTACAATAAAGTTAACGTCTCTCAGCCGAACAAGAACAAAATTGGATCATGCGTAATCAATGGTAATCACTCTAATGAAACACAAATAGAGATGGTTGACAATATAATAAATTTGGATGAATCATTCTCCTGTAATGAAGCACAAATAATTCACATGGGACAGTTTCACGCACAAATTTTCCTTGCCAAATAGCTCTCTAGACGATTGTGGCTGCCAGGAATCGCGTTTGTAGTTAGCTCGGCCAGCATAATTTC

>Ca2:28260986-28261466

TAATTCATTTGACAAATGCTAAAATTATTAAGTTGCATGTCATATGCTAGTTCATGACCCCGAATCTTTGATCACTATAATGTACACACTACAAACTCCTTTAGTCTTGAGAGCCTTCCACGAAACCTTTCTATGGACTATAGCTTACTAGTATGTGTTTTATCCATACAAATGAAAACCTCAAAACCATGTTTAAATATTTTCAGTTCTTCGAAGAATTTTCCATCAATCATTGTAAGAAATATTTAGCTTTCATGGACATGAAACCAAATTGATTCTATGTGATGTCAGTCTTGTCTTAATCTCTACTTAATAACTCCCCCCCATAGCTTCATATATGAAACTAAGCTTTACCCCTCAATAATTTGCAATAGATTTGTATGTCAAAACTTTTTCTTACAGATAGAGATTATAGTATCCTTTACCTTATTTTTCGCTCAATAGCCTAATTACCTGAATTAGAACTTCTTTCAAAGTCAT

>Ca2:28390189-28390419

AGGCTCATCAATTTACAATGCCAAATCAAGTTATAATTTTTTAAATTACAATTACAATCAAATTGCAACCATAAATTAAATTACAATAAGATTTAAACCAAACTAAATTAAATTACAATAATTTTTAATTTGTTGCTGTTGTTGCCTCTCTGTTTCTGATGCAATGTTTGGCTCTGTTTTGTTGCTGCCTTTCTGTTTCAGATGCAGTGTCATTGACCTTTGCTTTGTAA

>Ca2:28390968-28391180

CTTGGACGATGATGTTGTCGGCGCTGCCACCGTCGCCGAGGTTGCCGTCACCTTCCTCACCGTCGCCGAGGTTGCCGTCACCTTCCTTACTGTCGCTATTCCCTCATATCGTTTTCGTTTTCGCATCCTGGTTCCAATCGCGTTTCTGGCCGCCTCCTTTGTGTTTCAAATTGGCCGTTTTCCTTAAATGAAATTAGGGTTTCTTAGTTTCA

>Ca2:28838575-28838824

AGTGTCCATACTGCTAACATATGAGTTGCCGCATCATTATATATTTGTGGAGCCACCTAAAATCAATGCATCATATCAAATTGACAAATCATACACACAAAATTTTGTGCTATATATAACAATATATAAATCAACAGAATGAAATTTGTTTCATTTTGAACAACTTTGAACCTACATATATATATAATAGAAAAACTTGTTGGATTGTTCTCAACTCTTACATTATAAATGTTATTTGTTCCATTAAAT

>Ca2:29196586-29196865

CCAAAAAACGCTAATCCAATAAGACCCCTTTAGCATATGTATTGAATTTGTCTAAAGTGCTTTTGGGCTGCAACATTAGCGGTGGTCGATGCATCTTCTTTCTTCACGTTCAACATATTTTCCTTGGATGCTGAATTGAAGTTTGATTGAGAATCAACAAAAAATTGAGCTATGGGCTCCAAAAAGCAACTCAACACAGGAAAACTTGAGCAAACAAGTACTCCTAGTTTGATCATATTCATGGTTGAAATTTTAGTTATCAAAGTGTATAGCTTAAGA

>Ca2:29196051-29196866

CTAGTACTGAATTTCTTATATGCACACCAAAAGTCTAACATATTTTTCTTTTGAAAAAAGAGGGTTAAACCCAAGAAAAAAGAATCTACTGAATTATATGGATAGTCTTCGACATACAAGTTCCAAAACTGTCATCAAATAAGAGTCTCTGAAATTTACAATTAAGAGACTTCAAAACATAAAAAGTAAAAAAATGTAAAAATTATGATCGGCTATCAAAACATGAAATTCAACATACTTATATATTGCAACAACCACATATCAATAACGATCAATTGAGGTTAAAAAACTTATGGTATGTTTCAATCGGTGGTATATAGTGGAACGAAATAAAATGCAATGATATAACATCAATTTTTTAAAATCTAATTAATTATTTCATCTCAATCTAATTCATTCCACCCTATACCACAAAACTCGAAATATCCTAAGAGTTTTAGCTTAGACTAGAGAATATTAATCCCCCAATTTTTTTTTAAAAAACTAACAAAATCATTTTAAAAAATTAGACATGATTCCTAAATATTAATACTACCCAAAAAACGCTAATCCAATAAGACCCCTTTAGCATATGTATTGAATTTGTCTAAAGTGCTTTTGGGCTGCAACATTAGCGGTGGTCGATGCATCTTCTTTCTTCACGTTCAACATATTTTCCTTGGATGCTGAATTGAAGTTTGATTGAGAATCAACAAAAAATTGAGCTATGGGCTCCAAAAAGCAACTCAACACAGGAAAACTTGAGCAAACAAGTACTCCTAGTTTGATCATATTCATGGTTGAAATTTTAGTTATCAAAGTGTATAGCTTAAGAT

>Ca2:30323991-30324384

TCCTCGAGCAGAATCAGTTTGGAACACATCACCAATCAAACATTAAGCAATTGACAACAAGTAGAGAATAATACAAGGAAATTGGCCCATTTAATTAATTCTTATAAAATGAATACTGCACCCACCCAGCTAAAGTCGCAATAATAATCTCCATTTCTGACTACATATGTCGTGGTAGCAAGCATAATTTTGTGCCTAATATTATCAATGGATCAAGATCTCAAAAGAGATGTTTTTGATGTATATTCTTACAATTACAATAGAATTTCTCAAATATTCCTATCTAAAAACAACTTGAAATCATGGGATGTAAAACTTCAGCACCTGCAACCAGGAAGAGACTGGAATAGTGCTAAACATCTTGTGAATTCCTTAAACCGCACATGCATGATC

>Ca2:31310563-31310805

CTAGGAACTGAGAAAACAAGAAGAAAGCGTTGAATCCAAGTAAAGAGTTATATTCTGGGAAAAACACAGAAACAAGAATTGGAATCTGAAGTGTAGAAACTATATACAAATGTAAAACAAACTAATCGCATGAAACATGAAATGCTCAGAAAGATTGGTTAACGCAACTTGTCAGAAGAAGAAAAGTGAAAGAACAAATAATAATAACAATAATTTCCTAGTATTTAATTATTTATTTGATT

>Ca2:31310563-31310827

CTAGGAACTGAGAAAACAAGAAGAAAGCGTTGAATCCAAGTAAAGAGTTATATTCTGGGAAAAACACAGAAACAAGAATTGGAATCTGAAGTGTAGAAACTATATACAAATGTAAAACAAACTAATCGCATGAAACATGAAATGCTCAGAAAGATTGGTTAACGCAACTTGTCAGAAGAAGAAAAGTGAAAGAACAAATAATAATAACAATAATTTCCTAGTATTTAATTATTTATTTGATTAGTGGAGATTGGAGAATATCTT

>Ca2:31390887-31391178

TTAATTAATAAATAAATTTACATAAATACATACAACATAAAAGGCACAATTACAAATTTAATCAATAGTTGAAACAAACAAAATCTGTCATATATTACAAGTTACTCACTATTATAAAAGGAAAACGTATTACACTCACTCCTAATAACAAAAAATAAAAATTTATAGTGGCATTATTATCTTGTTTATAAGGAACTCAAAAAAAAAAAAAAAAAATTTTACAAGATAACTAATAAAAACAGAATTTATCTTGATAGTCTTTAACTTCTCTTCTCTAAGTAAGAAGAATCT

>Ca2:31613053-31613763

AAGAAGTATAAGAGAGCAATGATGGAAATGAAGAGGGACTTGGAAAACCAGCCACAAATTCTATTTCAACGCTTTAATTTGATGAATAACATTGAGTTCCTAAAAGCCGTTTACAAATGTGTTTCTGTAACTATTACTGGCAATCTCAAACCCCATATAATTAAACCAACATAGTATATAATAAGAGTAAATTAATTGCAATTAAAAATAAACTATAGTCTATAGAAATTATTATTAATTATAACAAGATGAAAATGAAAAAAAAAAATGCCTGGCTTTCCCATGTACGCCGCTAGTGATGAATTCTCCACCACAAATGAGATCGATCCTAGTCATCAAAATCTTACCTCGTACCAACAAAAAACATCATCAAACAATTGAAAATGAGTCTTAGCAAAGTTCATGAATTTCTTCTTCCTCTTCTTGTTCTTGTCCATGACCATCTAGCTATAAGTTCTCAATGCTTTGCTTATAAATATAAATATATAAATATTTGGCTCTAGAGATCCAAATTGAGGAGTAATAATTAATTAACACAAAAAGGGTATCACAATTTGTTTGTGTTTATAATAAGAAAGACATAGAGTGAGAGACAAAGAGAAAGGGAAGAGGTATATAGAAGAAGGTTAATGTTTTGTTCCCAAGACAAGAAGATGAAGAGTTAGAGGAATTCCCGTTGATGTTGTTTTCAAAGAAGGTTTTAATTTTCC

>Ca2:32295681-32296047

CACAATTATATAAACACACTAACTTTGGGCTATTTTTGGAGCATAGTCGGTTATACGCGAAATACAACTGGGAAATGCATTTGAGTGGAAGAAAAAGAGGTGCAAATCTCTATTAATAAAACACAACACACAAAAGAGGTGTGATATGTTTGTGAACGTGGAATGAATTACTCTCAGATGACTGCAATAGAGTGTGACTCTTCTTCATCAGCACTCCTACATCATCATCAGCATTTGCCTCTTTTAATTATTTAAATTATTGATACGTAGAACTTTAAACTCGTTTATATTTGTCTTATCACATCATCTACTTCTTCTCCTACCTTTCTTCAACAATGTTGCAAATGGTACAATAGGAGATGCTTA

>Ca2:32492816-32493088

ACTTTGAACATGATGATATGAATGATAAATCAAACAACGGTGCTATAACTTCATATATGATACTCATACCTATTAGCAAAAAAAATATACTCAAAGGAAGCATACTTATATGATTAAAATGATTCTAAAAAAATATAAAGACTATGAACAATAAGATTGTGACAAGATCTTGGAGATTTCTGAACAAGATGGTTTAAGATATAAAGAGGAATTCAAAGCTCATAACCTTCGGGAATAACCGGAACCTCCACATTTCTTGCAGAGAATCAATC

>Ca2:32663438-32663716

NNACGGAAATAGGGGGAAGACGTTTAGAGGCGTTAAGGACCGTTTGGAGAGCGTCAGTGACGGAGATCATTGTTTGATATGATGATGGGGTCAGTTGAGTATTGAATGTACTATAGTGGTAGAGAATCGTGTCTAATGTGACAGATAGTGTGACACTTATGATTTATTAAATTATTATTATTTTTATATATGTATGAAGAAAAGTATTTGGAGGAAAAGAAGGTGACCTTTGTCTCTTGAAGGTGGCAGGGGTTGGGAGATTTTAGGGATAACAATTC

>Ca2:32753158-32753389

CATGGAAATAAACTTCATAGGAAAAACATCCAAAGACATTTAAATTCATATCTTATAAAATACGAATAATAATTAACTAAAAATTCATTATGCTACAATTCTAAATCTCAGAAGCCTGTGATCACAGAGTGGTTGGTGACCTTAATCATATCAGCAGCACAGCCTTCTACAGGAATGGGGGCAATATCACCCACCGACGTGGATACCTCGGTCTCCCTTCCTTTCCATCAA

>Ca2:32868537-32868741

CTTACGAGAAGATAGAGAAATGGGTGTTGGAGTGTGGAGAGCAAAGGTGAATAGGGAAGAAGAAGAAGAAGAAGAAGAAGAAGAAGAAGAGAGAAGGGAAGATGAGAGGAAAGAGAGAGTTAGAAAAGAGACATGAGAGACAAGAGGGTGTTGAGCATTATCCTCGTTTTATTATATTGCTTTGCTCAATGTAAAATGTTAATG

>Ca2:32944337-32944958

ATTGTTAGTTGCTGTATATATATATTTAAGCTTCTGTCAGGGTATGAGGACACTAATACAAAATTTTGTGCCTAATCCTACAAATGCTGGTGAGCCAAGACAAAATTAAAGGAATGGTAGTTATGGATGTAATGAGGGGTTTTCCTAACCATAACTCACCTGCAGAATGGTGGTTAAGGATGTAATGAGGGGATTTCCTAACCATAATTCACCTGTTATTCCAGGTATAATCCCTGCTATTACACTAAGAAATTTAGCATACTGTTTAATTAGATAAGAACCCAAACCGATCAAACAATTTAAATTATTAAAAACAACCTTAGGTGAGGTCTATGATTGTCGCCTCCAATAATGTTAGAGCAAGTTCAATCACAAATTCCTTTTAAATTATAAATAAAAAGAAGAAGTTCCATGCATATCGCTTACACTCCAGAAGCCCATAGGAACACACAACTTCCTGAATTTTGCCTCTCAAACTTCATTGCAACCCTCATAAAATATAAGACCACAAATTATGTTCTTTTCAACTATCGTAGAATCTGTAGTCTTCTTTGGCAGCATTTCTAAATGTCAGCTCCGTGGATTAATCAAATGATGATTAATATAATAAGCCAATAATGC

>Ca2:32944337-32945730

ATTGTTAGTTGCTGTATATATATATTTAAGCTTCTGTCAGGGTATGAGGACACTAATACAAAATTTTGTGCCTAATCCTACAAATGCTGGTGAGCCAAGACAAAATTAAAGGAATGGTAGTTATGGATGTAATGAGGGGTTTTCCTAACCATAACTCACCTGCAGAATGGTGGTTAAGGATGTAATGAGGGGATTTCCTAACCATAATTCACCTGTTATTCCAGGTATAATCCCTGCTATTACACTAAGAAATTTAGCATACTGTTTAATTAGATAAGAACCCAAACCGATCAAACAATTTAAATTATTAAAAACAACCTTAGGTGAGGTCTATGATTGTCGCCTCCAATAATGTTAGAGCAAGTTCAATCACAAATTCCTTTTAAATTATAAATAAAAAGAAGAAGTTCCATGCATATCGCTTACACTCCAGAAGCCCATAGGAACACACAACTTCCTGAATTTTGCCTCTCAAACTTCATTGCAACCCTCATAAAATATAAGACCACAAATTATGTTCTTTTCAACTATCGTAGAATCTGTAGTCTTCTTTGGCAGCATTTCTAAATGTCAGCTCCGTGGATTAATCAAATGATGATTAATATAATAAGCCAATAATGCCTTCTTGATGATTAAGGTCAACTTGCAGCATCATGCCGACCAATCACATCATCAATCATAGAACGATATTTCTCAAACAGTGTAATGAATATCTCTAAAGTATGCTTTCCAATTTAACATCAAAACGTAAGCAACATTCTACAAATGTATGTATATGGTAAAAATTTATGGTTTTTGTCTCACCCCAAATATACATTTAAAGGGTGATGCTATCAAATGCCCTTAGGAAATAGAGTATAAACACCAGAATAAAAGAGCCTTAAAATACAGCTTAAAACCAGTTTGACATAGTTCCTCCAAAAATAATATTTGTAATGATAAAATTGACACATTTTCAGAAAAAGAAATATTGAATATTGATTCTGTCATTTAAGAAAATATTTATCATCTATCGCAGCTTTTGTTACCAAAACTAAATAATTAGCAATTTGGGCAATTTATCTAAAAAGACTGTCAAAAAAATAACAAAACGGAAACGGTGAGCGAAATTTGAAAGCCCAACATGACATTATCGAAAGACATTTTTTAAAAGTCAATTTAGATTATGTAAGTCAAAAGAAGAAGAAGTTCCCAAACATTCCCAGACAGTCAATAGTAAAAGAAAAGATCATACCCATTATGAACTCCAAAGACGAAGACGAGCATCAACCCCACCAAGTGTAGCCACTTTTAGCCTTGGAAGTGAATAGGGATGCAAGCTCCATCGGTGGATGCAGATGATAGAATTTACGAGCAGTTTCTAGCATTTGTTATGTGGCAAGGATCTATGTGT

>Ca2:32946269-32946757

GCAAGCACAACGAAATTCACATGATGTTTCCTTTCCTAAAGATAAGATCATACTTCTGCAAAAGCTGGTAATAGTTCTCATAAATCATGGCTGAAAGCAAGAGCCACATGTAAAATATATAACTTACACTTTTGCCAGTCATGCTTTATGAAAATAAACAACAATGATAAAACAGAATTGGAAATTGAAGAAAATGTGAAGTCCACAATAAGCTAATAAGTTATTGTAACAAAGTTTTCTCTGCCCCAAAATTAAACCCATTCCTCAAATTCCACAAAGGAACAACAAGAGGTACTACATGATTTTCAAAAAAGTCGGGGGAAAATCTTCTAAAGATAGAAAGAAAATACAGGTTTCAGAATCTGGTTCTTTAGTAATCAAATAGTAATTAAAATTACAAATTTAAAAGATGCATTTGAATAGGGCCTGGTACTTCAGAAGAATCAGCAGAGTCCAAAACTAAATGTTTGATGCATCTATGAGGGTAT

>Ca2:33352876-33353333

CCCATGACTAACATCAGATACATTTCAATTGGGCATATATCTTGAAAAACATAGCATAATAAACACCGTATAATTTGTACAGCTTCTTGTTCCTAGGTCCCTCACCTCTTCATTCAGCCATGGAACAAAAGGAGGTATCGGTAAGCCTAAATCTATATAATGAAATATAAAGGAAGAAAATTTTGGAAGAAATTCATCACCTTCTGAACTATTGAAAGAAAATGTGCAATTGTATTCAATCATGGCAAAAAATTGAAAATTTATGATGCTTTATGGTTATAGGCTAGAATTCTAAATTTGTAATTGTTACCGAACTTTATTATTTTGGATAGACATTTCAGATGGTTATCATCAGATAGTACCTTTGGACGTTTCTAACCAAAATTTCTCTTGCTTGCTGCAGCATTACTTGTGTAGAAGTCCATTTTATCGCCTTTGGTTAAAAATTGATTGTCTT

>Ca2:33649636-33650182

ATATACTAAACTTAACTGATAATTTGTTCTATCTTAAAAAATATACAACGACAATAAAATCATTATGGCATCATAAGATTTAAATATTGATATCAATAGAAAAAAAATACAATTTACAGAAATCCACATGACAAAGCATTAATGGGCTGCTTAAAAAAAGCCATTGATGGGATCATTCAAAATCTAAATCCCAACTTTGCCACCCATGAATGTAGCATAACTTTTCAGTGTTTTGGTTATGTAGGCTTCCATGCCATATCTCCATATTACAAGTTGTTGCTTTCATATGAGATGTGTGTATTTTGACACAATAACCACTGGAGTCGACCTCATATCACAGGCCTGGTTTTCAATAACTACTTAGAAAGAAAGTTAAAACATTGAAAAATATGATGCACCGCAAGTCCTTCAAGCTTGCTTCAGAAGGATTTACTGTTCTGATACACGAAATAATCTGCAGATTGTTCAACCATTGATCACACAACTTCACATCACTCTTGTAATCAGCAAACTGACATGTGTGTGTGCACATAAAGGAACTAAAAC

>Ca2:33650304-33650659

CAACAAATTGACTTGAAAAAACGTCGGATTTTCAGGAGTTCAAAATTGGGAAAGCAGCCCCATATGAATCACCTTAGAAAAAGATTTGTTGCTTGCTTTTTAAACTATGAAAAGGGCAGCATGTGTTTCATGCCACTGCCAAATCCTTTCATTTTCTGCATATAACTACTAAATAAACTTTCAATTTCATTGGCACAGAACTAGGGATAGCTGCAGCTAAAATGAACCTGAACCAGATATTAGGTAGTGATGGAGAATCATTCCCAAGAACAAGAACAAGGGGAAATACTTTAACTCTCTTGCTATGCTTCATAATTGCAATAACTTCAAGGTTTCCACTTCAACAAAATAGAAA

>Ca2:33650531-33650919

CTGAACCAGATATTAGGTAGTGATGGAGAATCATTCCCAAGAACAAGAACAAGGGGAAATACTTTAACTCTCTTGCTATGCTTCATAATTGCAATAACTTCAAGGTTTCCACTTCAACAAAATAGAAACTATGAACCAATGTGAAATGCCAAATCAATTTGCTGTTAATTCATAAGAGAATGAATATAACTAAAGCAATTTAGTTCTTTAATCAGCAGTCCCAGCCCCCAGTTGTGTTTACCAAATTATATTGTCTCCATGTTTAGTAATCAAAACTCCTGGTAAAAATACTTACAAAACTATTTGTCTGAAATTCATGAAAGAAGGTGCAGTCTCCCTCCGGTTAACCATATGCTTAGAGCCTTAGATACTTAGAGACAAGGATGGC

>Ca2:33649636-33650919

ATATACTAAACTTAACTGATAATTTGTTCTATCTTAAAAAATATACAACGACAATAAAATCATTATGGCATCATAAGATTTAAATATTGATATCAATAGAAAAAAAATACAATTTACAGAAATCCACATGACAAAGCATTAATGGGCTGCTTAAAAAAAGCCATTGATGGGATCATTCAAAATCTAAATCCCAACTTTGCCACCCATGAATGTAGCATAACTTTTCAGTGTTTTGGTTATGTAGGCTTCCATGCCATATCTCCATATTACAAGTTGTTGCTTTCATATGAGATGTGTGTATTTTGACACAATAACCACTGGAGTCGACCTCATATCACAGGCCTGGTTTTCAATAACTACTTAGAAAGAAAGTTAAAACATTGAAAAATATGATGCACCGCAAGTCCTTCAAGCTTGCTTCAGAAGGATTTACTGTTCTGATACACGAAATAATCTGCAGATTGTTCAACCATTGATCACACAACTTCACATCACTCTTGTAATCAGCAAACTGACATGTGTGTGTGCACATAAAGGAACTAAAACCTGCACAGCATACATGCACACAATTCTAAGCAGGGAATAAGTAATGAAGAATAATTAGAGGAAGCTTGAGTCAATGTATGAGCTACAAAACTTACATCCCTCTTAACTAGTGAGATACATACCAACAAATTGACTTGAAAAAACGTCGGATTTTCAGGAGTTCAAAATTGGGAAAGCAGCCCCATATGAATCACCTTAGAAAAAGATTTGTTGCTTGCTTTTTAAACTATGAAAAGGGCAGCATGTGTTTCATGCCACTGCCAAATCCTTTCATTTTCTGCATATAACTACTAAATAAACTTTCAATTTCATTGGCACAGAACTAGGGATAGCTGCAGCTAAAATGAACCTGAACCAGATATTAGGTAGTGATGGAGAATCATTCCCAAGAACAAGAACAAGGGGAAATACTTTAACTCTCTTGCTATGCTTCATAATTGCAATAACTTCAAGGTTTCCACTTCAACAAAATAGAAACTATGAACCAATGTGAAATGCCAAATCAATTTGCTGTTAATTCATAAGAGAATGAATATAACTAAAGCAATTTAGTTCTTTAATCAGCAGTCCCAGCCCCCAGTTGTGTTTACCAAATTATATTGTCTCCATGTTTAGTAATCAAAACTCCTGGTAAAAATACTTACAAAACTATTTGTCTGAAATTCATGAAAGAAGGTGCAGTCTCCCTCCGGTTAACCATATGCTTAGAGCCTTAGATACTTAGAGACAAGGATGGC

>Ca2:33650304-33650919

CAACAAATTGACTTGAAAAAACGTCGGATTTTCAGGAGTTCAAAATTGGGAAAGCAGCCCCATATGAATCACCTTAGAAAAAGATTTGTTGCTTGCTTTTTAAACTATGAAAAGGGCAGCATGTGTTTCATGCCACTGCCAAATCCTTTCATTTTCTGCATATAACTACTAAATAAACTTTCAATTTCATTGGCACAGAACTAGGGATAGCTGCAGCTAAAATGAACCTGAACCAGATATTAGGTAGTGATGGAGAATCATTCCCAAGAACAAGAACAAGGGGAAATACTTTAACTCTCTTGCTATGCTTCATAATTGCAATAACTTCAAGGTTTCCACTTCAACAAAATAGAAACTATGAACCAATGTGAAATGCCAAATCAATTTGCTGTTAATTCATAAGAGAATGAATATAACTAAAGCAATTTAGTTCTTTAATCAGCAGTCCCAGCCCCCAGTTGTGTTTACCAAATTATATTGTCTCCATGTTTAGTAATCAAAACTCCTGGTAAAAATACTTACAAAACTATTTGTCTGAAATTCATGAAAGAAGGTGCAGTCTCCCTCCGGTTAACCATATGCTTAGAGCCTTAGATACTTAGAGACAAGGATGGC

>Ca2:33650304-33650692

CAACAAATTGACTTGAAAAAACGTCGGATTTTCAGGAGTTCAAAATTGGGAAAGCAGCCCCATATGAATCACCTTAGAAAAAGATTTGTTGCTTGCTTTTTAAACTATGAAAAGGGCAGCATGTGTTTCATGCCACTGCCAAATCCTTTCATTTTCTGCATATAACTACTAAATAAACTTTCAATTTCATTGGCACAGAACTAGGGATAGCTGCAGCTAAAATGAACCTGAACCAGATATTAGGTAGTGATGGAGAATCATTCCCAAGAACAAGAACAAGGGGAAATACTTTAACTCTCTTGCTATGCTTCATAATTGCAATAACTTCAAGGTTTCCACTTCAACAAAATAGAAACTATGAACCAATGTGAAATGCCAAATCAATTTG

>Ca2:33649636-33650659

ATATACTAAACTTAACTGATAATTTGTTCTATCTTAAAAAATATACAACGACAATAAAATCATTATGGCATCATAAGATTTAAATATTGATATCAATAGAAAAAAAATACAATTTACAGAAATCCACATGACAAAGCATTAATGGGCTGCTTAAAAAAAGCCATTGATGGGATCATTCAAAATCTAAATCCCAACTTTGCCACCCATGAATGTAGCATAACTTTTCAGTGTTTTGGTTATGTAGGCTTCCATGCCATATCTCCATATTACAAGTTGTTGCTTTCATATGAGATGTGTGTATTTTGACACAATAACCACTGGAGTCGACCTCATATCACAGGCCTGGTTTTCAATAACTACTTAGAAAGAAAGTTAAAACATTGAAAAATATGATGCACCGCAAGTCCTTCAAGCTTGCTTCAGAAGGATTTACTGTTCTGATACACGAAATAATCTGCAGATTGTTCAACCATTGATCACACAACTTCACATCACTCTTGTAATCAGCAAACTGACATGTGTGTGTGCACATAAAGGAACTAAAACCTGCACAGCATACATGCACACAATTCTAAGCAGGGAATAAGTAATGAAGAATAATTAGAGGAAGCTTGAGTCAATGTATGAGCTACAAAACTTACATCCCTCTTAACTAGTGAGATACATACCAACAAATTGACTTGAAAAAACGTCGGATTTTCAGGAGTTCAAAATTGGGAAAGCAGCCCCATATGAATCACCTTAGAAAAAGATTTGTTGCTTGCTTTTTAAACTATGAAAAGGGCAGCATGTGTTTCATGCCACTGCCAAATCCTTTCATTTTCTGCATATAACTACTAAATAAACTTTCAATTTCATTGGCACAGAACTAGGGATAGCTGCAGCTAAAATGAACCTGAACCAGATATTAGGTAGTGATGGAGAATCATTCCCAAGAACAAGAACAAGGGGAAATACTTTAACTCTCTTGCTATGCTTCATAATTGCAATAACTTCAAGGTTTCCACTTCAACAAAATAGAAA

>Ca2:33798493-33798933

TGCCATACAAAACAAACAAAACACAACTATAGTTTATATAGAAGCTTACACAGATTAATTACTTTATAAATACCCCATCTCTTATTCTACAAATGCAGCACAAGTGCTATAACTCTTCATTTCTCACCTCATCAGTCTCCTCCAAATCAACTATGCGGCTGGCGCTATATCGATATCGCCATCATCATCGTCGCCATCCTTCTCGCTTCGGCCGGAAAAGTGGTGGAATTTGGTTGCAGCCAAGTCATTTCCCATGAGGAACATAGGAAGACCAGATTCCATGGAACTTAAGACCACCATTTTGCAGCACAAAAATGTTCTAAAGAATATTAAAGTGTTTTGGTTGTAATGTGAACTAAATATGTTGACTCAAGATGAGGTCATGAGTCTTCCAAACCACCTTATTTATACACTAAAAATATTATATAAATTTACTGTGG

>Ca2:34548331-34548663

TGAAGGAACAATTCAAAAATGATAATATCGTTATATGGCCCAAAAGTTTTAAGAAAACATGCCCTAAATTTAGAAATCAGTATGCTAATTATGCGATCCGGCATTCTGCAAACAATCTACACAGCCGATTTACATGTTTAATGCTCATAGTTGCAGTACAATAACTTCCTCCATAACACAAATGTTGTTCCAATATAACATTCTCATCACGACATAAAGAAAACCCTAATAAGATAATGATTCAAGGTTGTACAAGCAACAATTACTTCCAAGGTACGAGCTGTTATAAAAATTCCGACGTGCATATCTTCAAAGCTCCAGCAAGAAGAAAC

>Ca2:34606154-34606384

TCTGACAACATGGTCATGGCTTCCACATGTTTTGAAATTGCAGATTTGAAACTGATCCAGCACAGGAAGCAACTATTTTTTATGTGCTAAAATTATGAAATTTTGGCAAATTGTTTTTTGTGTAAATTGTAGAAAATCATAGAGTATCTAATCTATAAAAGGCACAGATTATCTATGTCTTATTTTAGAGAGCGTGCGGATCCACTTCCATTGATTTGACTTTTACTCCC

>Ca2:35468002-35468403

GTTCAATAACAAATTCCTAAAATATTTTATGATTTATCCACCGGATTTCATCCCTTAATTTGGCGAAGACACCATTTTGCTAGACCCTTTTAGGGTTGCCTTTCTCCCGCCTGCCAGCTACTTTTCTTGTCCTTACACAGTCTCAAGATGGCCACCACCACCACCCTTGTTTGTTTCGTCTGTTATCTTAGCAGAGTGCTTTTTTGCATCCGAATCACTTGCCCCGTTTCCTCTTTCAACAACACCTATGACAGCCACTTCTTTTCACATTAGAGCAAAAACTGTCCCGTTTTTGCTGATGGCCTTGTTTGTTTTGCTTCTTCACCAAGTGAGAGGATCCACAACGAAAATCATTTAAGAAAATGTGAACAAGCAAAACACAGATAGCTCTGCAAAAGACG

>Ca2:35954551-35954864

CTTGACAATAAAAAACTCTAATTATTAGATAAAATATTAGTCACACCAATTTATTAAATTAGTTAAATAACTAATTAATCAGTGGATCTAATTAGTGTAACTAATATTATATATACCAATACAACTTCAATACCATATTTAAGTCTAACTTGGATCTAAATAGGATTAGATCTCACTTCTAATAGTTAGGTCAAATATTTCATTCATAGGACTATGATGTCTAATCAGTTTTAATAGTTAATTAAAAGGATGAGGATATCTAATCACGTTTAAATTTTAATTATCCAAAAACTTTAGTACCGATAAAAGTTGA

>Ca2:36331805-36332083

CATTGATTTTGGGGAAAGAGATCTGAATTGAATTGTGAACTGAACGTAGCGGGTGGAAATGGAAAACGAAGATGGTTGGGAATGGGAATTACGATTTACGAAGGAAAAACAATATATGATTAAATGAGATGGAGATGTGGGTCAATGTCAGGATTAACTAACTAACTCTTCAAACTGTGGCCCATCTAAGCCCACTTTCTCGTTTGGGCTCAGATACTAATTCTTAAGCCCGTTTTCAAAGACGGCTTTTAAGCCCGGCTCGATAAATCTCATTACAG

>Ca3:549632-550021

CCACACTTTCTTTTAGTTAAAAAAATCCCCTCAAAATAACTTTCGCAATCTCATCTCACTCATTCTCAAATCGCGCCACCTCTGATCTCGCCCACCAGCTGCCCACTCATCTCCACGCCTCCATCCAGAATCAGACCCTCTCTCTACATCCCAAGATCGCGCCGCGCGCGACCATAACCAGTCTGAGTCCACCGCGACCGCCTCCCTGCACCGATCTGGACCACCACGCCTGCTCTCTCCCAACAGCACACATCTTTCATCTCTGAATTCTCCCTTTTTGCACCGCGCCGCCCAAGCTTACACTACGCCTCATTTCTCTCTTCATCAGCACAGCCCTCAACAACCTCCGGAGTCCAAGAAACCGCCGCATCCTTCTGCTCCATCAGTTG

>Ca3:556942-557281

GACATTGTTGAGGCCAGGGACTATCTGAAGCAAAGAGGAGTTGATGTTAGTACCAGCTGATGTGGTCTATAGATGTTCCCTGAAGTTTAGAATTAGCTGCCAGCGACTAATTAATTAGCTGATTGGTAGTTGGGAAGGATTTTTTGGCATTTACATTGTACTCAAAAACTAGTATACGAATGGCTTTGAATTTCCACCAATGTATGCATACTCTTTCTGTTATGAGCTATAGTTGTCTTTCTTTGGCAACACATGAAACTGAGATTTAATCAGGATAGCTCACATAGGCTTGATAAGTGATGTTTTGTATTCATCATGACCCCGTTGTCTCTATTTTAC

>Ca3:549638-550021

TTTCTTTTAGTTAAAAAAATCCCCTCAAAATAACTTTCGCAATCTCATCTCACTCATTCTCAAATCGCGCCACCTCTGATCTCGCCCACCAGCTGCCCACTCATCTCCACGCCTCCATCCAGAATCAGACCCTCTCTCTACATCCCAAGATCGCGCCGCGCGCGACCATAACCAGTCTGAGTCCACCGCGACCGCCTCCCTGCACCGATCTGGACCACCACGCCTGCTCTCTCCCAACAGCACACATCTTTCATCTCTGAATTCTCCCTTTTTGCACCGCGCCGCCCAAGCTTACACTACGCCTCATTTCTCTCTTCATCAGCACAGCCCTCAACAACCTCCGGAGTCCAAGAAACCGCCGCATCCTTCTGCTCCATCAGTTG

>Ca3:549652-550024

AAAAATCCCCTCAAAATAACTTTCGCAATCTCATCTCACTCATTCTCAAATCGCGCCACCTCTGATCTCGCCCACCAGCTGCCCACTCATCTCCACGCCTCCATCCAGAATCAGACCCTCTCTCTACATCCCAAGATCGCGCCGCGCGCGACCATAACCAGTCTGAGTCCACCGCGACCGCCTCCCTGCACCGATCTGGACCACCACGCCTGCTCTCTCCCAACAGCACACATCTTTCATCTCTGAATTCTCCCTTTTTGCACCGCGCCGCCCAAGCTTACACTACGCCTCATTTCTCTCTTCATCAGCACAGCCCTCAACAACCTCCGGAGTCCAAGAAACCGCCGCATCCTTCTGCTCCATCAGTTGGTA

>Ca3:1697431-1697732

CTAAAACACAAAAATATATAGTACTCATTTTGGGTCTATAAGCTCCAAGTCGAAAAACAAATGGTCCAATACTGATTATTATTAATTAAGGCTTCAAGTCACATATTCACCATCTTTGACCAAGAAGAGTTCTGAATTAGGCTTTGGCGTCAGTATAAAATTTGTAGCTCCACCTCTTAGCTTTCCAACTCCTACTCGCACGCGTCAATCCGCTATTTGAAGCTCAAGTTATGGTCTGCAGAGAGAGCAAGAGTCAATATGCAAATAATTTCAAAATAATGTCAAATAATTATATTAAAAC

>Ca3:2908331-2908998

GCGAGAGTATGAAAGAGAGAGAAAAAAGGAGAAAAGAAAAAAAGAAAAAAAGAGGAGATAAGAAGAAAAATGAAGAACTGAGAGGGAATAAAAGGAAGTATTTTGTTTTAAAAGAAAAAAAAAGGATAGAAGAGAAAATCACACACATGGAGTTTGGGTGTGAGAGAGTATAGAGGCGACAAAGGGCGTCGTTTACAGTGCGGCCACGAGCTCAGTAACAGTGCGATGGCGGTGTACGTCAGAGTTGCTCCTAAAAAGTGAGGTAGTTGTGGTTGGTTTGAAGTTTTCTAGCGGTGTTGTCTCTGACGGAGGTTGTGGTGATGGTTTAATAAAGGATTTGCAAAAGATGATGAAAATAGTGGTGTGTGTGGTTGAAGCTCTCCCTAGCTATATGTGTTTTTATTTTATATTATGTTTCTTTATTTTCTCTCAAAATCTCTTCCTTCTACTCTTTTATTTTCTATTCTTTGTATATGTTTCTTTTTTTCTCGTTTGGTCTGATTTTCTTATTTATACTCAAAAATTAGGTTAAAGTTATTCTTAGATGAAGAAAATGCCAAATTGAAAAGCTTGACGTTGAAGTAAAAGAGTCGGTAAATTGATGTTTTTGTTATTAACATTGAAATGTCCCTTAAAGAATTGTTGTCCAATTTTTTCTTTTGGTCCC

>Ca3:4189095-4189449

CTCTTTGTTCTGCACAAAGTGTACCAAGACTGACTAAAAAAAAATGTTTTTTATGTTTCTCTAATTAGTTACAAAGCAAAGGCCAATGACAACTACATCTGAAACAGAAAGGCAGCAACAAAATAGAGCCAAACACTGCATCAGAAACAAAGAGGCAGAGAGGCAACAACAACAACAAATTAAAAATTATTGTAATTTAATTTAGTTTGGTTTAAATCTTATTGTAATTTAATTTTGCCTTTATTTTAGTTTGTAATTTGATTTGGTTTTAACATTCTAAATAGATTTGAAATTGTAAACTTATAATACATAATGAAATTATTTATTGTAATTATTTTATTTCCTTTTTTAAAT

>Ca3:5016693-5016918

GCAGGAGTTCATTCTTTCAATTGGTTAAAGCAGATAAGAAAATCATAACAGAAAGAGCACCACCAAATCTAATGGTTTGAAATTCGAAAAAAGATATAATTTTACTCTTTATATCTTTCTTATACTTTTTTATGTTAATTTATTGGAGTTGAATTCTAGTGATGGTCTCACTCATTAGTTTATTCATATAAATCTTGAATTTTCAGGATAATATTTGCTTGCTCG

>Ca3:5102845-5103566

CTATCATCATTCTTTCTGAATCTGCTAAATTCAAGATAAAGATTTTTTAGAAGAAACATGGCGAAGGCAAAAGGTACCTCTTGGGGTGGTTCATGATATGCTATTGCGTTTGCAGGAAAAAGAGAGAAATTCTTCGAGTCATTTCACTTGTGCAGGTGAAGAATAATCGCCAGTTTCATGAGTGGTTGGTTGGTTTGATGCACGATTCTCTACCTTCCAATACTCGACTCGAAACCAGCATATAGGTGAAGAATTATCATAATTTAGGTGATTCAGATGTAGTATGATCCCTTAACGTAGGTGTTGATATTGTATTAGCGTTCACTTCATTTGATATCAACGGTCCCCATTATAGGTCTCCCATGCAGGAAGGTTCTGTTATACTGTAACAGAAATGACAGGTGGCATGGGATGATAGGCAGAGAATATTCTAGAAGGAGAGAGGGAGGACTTTATGAAGGAAAACGGTGGGTACCGAAGAGGGCATCTGAGAAATAGTAATTGGTTTTGGGCTGAGTAGAGAAGTGGGTTCTTTCTCAGAATTAGTTTGATAATTATATTGCAATGTTTATTCTGTTTGGTTTGGTTCCTATCAGATGTTTATTGTTAATTCTATTATACTCAACGACTGGTTGACATGCTTATAAACATATTTTAGCACTCTAGATATGTCCTAAGCAAGTTTGAGTAGACTTATTCCTATGTGATGCTCTAAATGACA

>Ca3:5220485-5220808

GTTATGTTGTGTTGAGATAGGATGATCAAAAATATGTGTTTCCTTACTTTGCATTCATCTATTATGGAACAGACTGCAAAGTGAAAATCTTGAAGTCACAGCTTTCTCTGGGAAAGAAATGACTGTTCTTTAGTAACCAACACTGTATTTTTGGAGAACTAGTTTATGTATGTGTATTTAAAGTGGGATTGCATTCCCTAATAATGAATTGAAAACCAATTTTATGTTGTTAGAATTAGAATAATTTTAGCCGACTTTCTGTAAATCAACTACTTGATTTGATTTTTGATACAATGATAATGATGAGGTTCTCCTTAGCCATA

>Ca3:5500591-5500798

CCCTAAGTGTTGAACCCTTCCCTCACACACTTGAACCCTTCTTTCACACTTCCCTAAATCCCTAAATCCTTGGCTTCGAATCTGATTCCTAAATCCCTAAGTGTCGAACCCTTCCCTAAATGCAGTGAGTATCGCCGACCGAATCCATCACTTTCAGAGTCGGTCACCGTCGCAGCAGTGAGTATCGCCGTCGCCGTCCCTTTCCTG

>Ca3:5663150-5663505

GCATGTCCTTGAGGTAATCCATATATAACTTGCTTGCAAATAACAAGAATCAAGACTGACAAAGATATAAGACTTCCATTCTGGTAAACACTGATGATGTGGTATCTAATAAGGCTTGCATCCTAAATTAATAAGAGTTGAATGCATTGTGAAAGCATTGTTCTTCCTCAAATCCCAAGGTATATAAACATCCTTCAACTTCCACAAATACTAGGGTTTATAAACAGTTGCAGGAGTAATTTGTGCCCGCCTATGTAGTGTTGTAGAATTATTTATTGGTTGTGAAATTGTAAGATCGTTATGACATGAGGTTCTTGATGTTTAATTGGATGAAGCTATTTGGACTGGTTTTGCT

>Ca3:6481649-6484743

CACACCCATTTATTCTTTTTTCCTATCTCCACAAACTTTTCGTCACAAAGTAAAGAAAGAAGAAAACTCTAGCCTCCTTCCCTTGCTTTTCTCATGCCATTTCCAATTTTCTTCCAACAGATGGTATCATCTTAAGGTAAATTTCTTCCATAGTTTCTTTTGATTTCTTTCACTAAGGAAAGAAAACTAGTACAAGTGCTCATTAGTAAAACTAGAACCTTTCTCTTATTCTATGATTGGTGAGTCTTAGAACGTGATGCTAGGAAGAAAAACAGCTAAAGTAAAGGCTTCTCCCAATGTAAAGTTAGTAATTTAATGAATTTAAATTAGTGGTTTCTTAGATATTGATATGTCTTGATGTCTTAAAAGAAAAGTGGTGATTTTTATATTTGTCTTAATGAACTTATCTATAAAGTTTCAAATTTTTTTTTTATGAGTAATATGTTTTTTAAATGAACTTAATTATATAGTTTCAATTTTTATTATTATAACATGAGTTATATGCTTTTTGTGATATATTTGATGTAAAATTGCAATTTTTGTGTTGTCTAGGTGTACTTAATTATAAAGTTGTGATTTTTATGATGATATCATGAGTAATATGTATGTTATGATGTATTTATTTAATGTTTTGATTTCTATGTATTTTAATTACAAAGTTTTGATTTTCATGAACAATAGTCTTGAGGCAATTGATGAAATTTTTTTATTTTTAATTATGAGAAAGTTGATTACTATGATGTGGTCCGGTGTAAAGTTCGATTTTTATGCTTAAATGACTAATAATAAGCGGAATTTTATAGTAGAAGCAATAAAGTTTGTACTTTTATGTGTTCTTGGTGATTTAAGTTTTAATGTTTATCAATACCAAGTAGCTAATCAAATTTTAAGGTTTATAAAATGAAAGTTGTGATTTTGGGATATTTATTCATGTGTATTTATATGTTCTATATTTTGGATATTGAAATTACTGATTCTTGACTTTGAGCATTCATGAGAATATAAGTTTGATGTCATATGTGATTAAAATTTAATATTGAATAATTTATTTTTGTGTTTGACTAAGTGATTTGTGTTTTGAATTTTTTTTTTCTTTATTGTTATCTAAGTGGCTGATGATTTGTGTTTTGAACAATTTGTCTTTTGGTTGCCTAAGTGGTTGAAATTGTGCTTTGTACATTTTTTGTCATGTGGCTGGTGATTTGTATTTTGAGTATTTTATCATTTTGAATGCCTAAGTGAGTGGTGATTTGTGGTTTAAACTTTGGAGTGGCCTAAGTGGCCGGTGATTTCTGTTTTGAACATTTTGTCTCGTGGTTGCCTAAGTGGCTGATTTGATTTGTGTTTTGAACATTTTCCTCTTTGTGGTTGCCTAAGTGATTGGTGACTTGTGTTTGAACATTTTATCTTTGTGGTTGCTTAAGTGGCAGGTGGTTTATGTTTTGAACATTTTATCTTTTTGATAGCCTAAGTGGCTGGTGATATATACCTTGAGCATCATTATCATGACATGACATATACATCAGTATGGGTTACTTTTGTGTGGACGACTTCGTGATTGGTTATATATAGATCATATAGTTTTAAGAGCATGCATAACATGCATATATTTTTATTTTTACTTTAATCTAAATATGAATTGCTACGTGATTTAATCTAGTACATATTAATCCTTAGTATAAATTTAATCTCAACACATTTTTGTGAATTATTCTAAAACCGATTTATTTAAATCTTTTATTTCAAAAATATTTTCTAATTATAATTATGACTCTTAAATTCTTATTTGGTTTAATTTTTGTCGTGGGATGTGTTGATTCCTTACACCAACATTTTAGATACTAATGATCATGAAGACATGCACGATTGAATTATGTTTGTTTAATGCGCGCGTGTGGTACAAGGTTGTTTTTATTTAAAAATAACGTTTTTGTATTAATAAAACCAACGCAATTCATTTTTCAATATTATTTACTTTTTATAGTTAACTTTAATTAGAAATGCAAAGGTGGATTTTTCTTGTGTTAGGAAATAATTACATCATCATTAAATTTAGTAAACCTTATTTACATTTGGAAAGAAAGACTCAACAAATTAGATTAGTAGGTGAAAGAGCTCTTAGAAATGGAAAACTATGAGATTTTAGAAAAATTGAATTAATACCTAAAATCTACAAATAACTTTCTTTGCTTCATATTCATAAAGCACATATGCATATTCAGATTGCCCTCTTAGAATAGATTTGGCATTTTAAAAAGCCATTGGTGGAATAGTGTTGGATGTACATGAAGTTGAAGACATTGCAGGTTGAAGAATTTTGTGGATTGAAGAACTTAGGTTTTATAAGGTTTGTTTAGAAAAATAGCAATAAGAAGAATGGGGAAAATGAAGATTTTATGAAACTTTATAGTTGGAATATCCTCATTTATTTATGGTGATGTTTAAGTGAACAATCTAAGATGACATATTGCAAAAACATGGGTTTCTTAGGAAATTGTGCGCAATTACTTTTTTTTACATGTCGTCTTCTCTTGGGGAGTGGGTAAGGTATAATCGTGTATCATCATTGTGTTAGGTTGAAAAGCGTTGCAATTGCGAAGTGTCATAACAGCTATATACAATGGTTGATTCGAAGACTCTAATTTTGTGTTGCTAAAACGTAAAAACTGTCAATTTCGAAGTACCTTCAATTATCAACATTTTTAGGGGCATCTATTATACACAAAATTTTAATGTAGGATTAAAAGTCAATAAGTGGGCACTTTGAAGATAGAAAATATGATAGCTCTAACGATAATTTTCTAGATAAATGTTGAGGTTAATGATGGAAAGAGTAAGATTTTAAGAAATAGGAAAAGCACTCGAAGAAAGATTAGTAAAGACATGAGTTCGAACATAGAAACTTGTAGGAGAAGTTTGGAGTTACTTGAAGAGGTTTCTAAAGGAGGAAATATTAAGAACACATACCTTACATGCAAGAATAAAGCAGTTACCGCATTTACCTATATATATAGGAGTTGCGTCAAATAAAAAAGGCATTCATAGTGTCATTAAAGCATAAATGGATTAATATCCGCTCTCAACAGAATTCTCC

>Ca3:6485449-6485763

GACGGCTTTGGTGTATAGGGGATAAAACATTAGCGACAAAATAAATTATTCTGCGTATCTTTGGTTTTCCACATTCAAATAATTCTATCTCTTTGTGTCTTTCTAGGTAACCTTCTTTTAAGAGAAAAGTAACAAATCTTTGTTATTAAAATAAGAAAAGCAACTTTAACAAAAAAATAAAGTCTTGGTCTTCCTTGCTCAACAAGAATGCATTAACATATATTTCACATTTCATTGGCTCATTAATTCAACCATTTATCTTAACAAGACTCTGAATTATCAAGCATAAGAATTATAGGTTCTACCACCTCCAA

>Ca3:6809674-6809896

GTAATGGTATTCGATGAAACGACTTAATCTTTTGTGATGAAATGTTACTGTCGTCGATGATGCCAACAACAGAAGGAAAGTGAAAATACACAAATATAGACATTGTGAATACTTCAGACAAAGCACTAATATCATGTACAGTAAGTATATACAAGTTCTATTAACAATCATGAGTGTAGTTTGATCTCATCATGTTGATTCCACATTTATACTTACAAGGAA

>Ca3:7242333-7242533

AGAATATAAGAATAAGAATTGGAAGTAAAGAAAAAAAAAAGAATGAGTTAAACCTTGGAGCATGGTATCTCGTTAGTAGTAAGAAAACAAAACAAAACAAAAAAATCTGGAAGTGGAACTGAGCCTCTCTTCGGATCTCTCCTCCGCCTCTCACGGCGGAGCCTCTGCTCTACACGCCGCCGAAACTACTCGCCGACAAG

>Ca3:8855324-8855729

ATTACAACCACACCAAGGTGTATATTTTGACTAAGTCAATCTTCAATATTAATTGCAAAAATCTCATAAGACACCGCCGCCACCTCCACATCCTCCACCGCCGCCGCATCCGCCACCATGTCCTCCACCACCATCGCCGCCGCATCCGCCTCCATGTCCTCCATGGTGGCCATGATTTTCAGCTGAAGTGGGGTCAACAGGAGGGGTTGCTATAGAGGTCATAAAAACCATTTTACCATTTTTGGCGCCTCCATTGTTCTTCTTTATTTTATAATCTCTTTTCTTTCCATGTCGGGAGCATAAATTTCCCATATTATATGTTGTCGGCTTTAAGGCATAGTTTGCTATAGCGCTTTACTCTTTATATATACACAACAAGTTCTATCTATCAAAATTCTACCAAAT

>Ca3:9990587-9990858

TGATAGAGAGAGCGAAAGAAGGTGAGAGTCTGGAGGAACTGCTGCTCAATTCGATTCGATTTTGTCTATAATTACATTAGTGTTATAGTTCTACTTATTTGTTGTGACAATGCTACGATTCAATTGATATTTAGTTGATTTATTAATTGTTATAATGAAGGAAGTGTGAAATTTAGTTAAATTTTGAATATGACTTGTTAGATTGAATCAAATTTAACCGATGTAATAAATTAATTTTGAATTTGTCTTGTTAGATTGGTTCAAATTTAAC

>Ca3:11005074-11005386

AAAAAAGATTTAACATTACAAAGCACCTGAACCTCCAAACCAATCTTCTCAAACATCCTAAATGCCCCAGACTTTTGAGGATTATACACCCCTTGACTCTACCAGCCAAGTCTTCTCTAAAAACCACAGGTTGTTGAAGGCACACTAACACCACTATTGGGTTTGAATACAAAGGTTTGAAACTTGAGTAGTTGCTTGTAACAAGATGAGTATAACAATGACTATCACACTAGAAGAAAAACATTTAAAAAATATTTCTCATTCGAACAAGTGTTTGTCTCTTTGAACTCTAAGATGAATTTGGAATTTTGA

>Ca3:13351077-13351352

ATTAATGAAAAAGAAGGCCCACACAAAAAATGAACACTACTTAGCAGCTAAACGCAGTCGTTTACTCTCCTATCAGAAAGAAACAAGTTGGAGTCACATCACTGAGCTACTCAACTGCCCGGAAAACACAGCCGGAATTCCTCGTCGGCCGCTGCCACAAACACTGAGAAATTCCGCCTCAAGGCTTTCCAGCACGATTTGCGGCCGCGATAGCCGCCACGGAAAATTCACAACATTGCTGATAACACCGCTACCTCTACAACACTATTACATAG

>Ca3:13356020-13356365

GCATTGCCTCCAGTTGATGGAATTGATGGGACCAAGAAGAAAGTAGATGAGAAAGAATGATGATCTCCTGGTTATGAATTGTTTTTCAATATCATGAAGATGAGAGACATTTAGAGGATTGTTCTTTTGTTCTTCTCATACGGTTACTTTTTGTTATCTTGGATATTAGATATAGAATGAGAATCTTAATTATTATTATTTTTTAGATTTTGGTTTGATGTGCAAAGTTGAGAAAGGGTGTGTTTTATAAACGGGAGTTATTTACTTCTTGGATTAAGAGGATATGTACTCTCACTATAAATTAGTTTTATACTAACATTGAATAAAAAATTCACGATTTTGCCT

>Ca3:13853596-13853836

AGAAGATTGGAAATAAAAATAATTTTCTAAATGAAACAAGCCAAGATTGTCATCTTTCTAACCCAAAAATCTAAACTATTATTCCACCCCAAAACGCTTTTCTTTTTCAGAGAGGTCGGCGGCAGTCGCAAACACAAACAAAATCGCCCTTTCTGTTTCATAGGAAAAGTCACGACGGTGCCGTTCAGGGAAGTCATGGTGGTGCCGCTCAGGGAAGTCGCGATGGTACCAACTATGGAG

>Ca3:13854306-13854528

GCATACAAATAAAAATGTGCAACATATGAAGTGGTAATTATTTGTATCCAATTATATTTGATAGAATATTACTGCATTTGGATGTTGAATTCCACAAGGTTACATGACACTTCGTAATTTATATTATGGATGATTATTATGTACTTGTCTTATTATGTACATGGAACATACATATTTTATGTGATTTTTCTCTCAGACTATGTTTGGGTTAATGGAAAGGAA

>Ca3:13853636-13853836

CCAAGATTGTCATCTTTCTAACCCAAAAATCTAAACTATTATTCCACCCCAAAACGCTTTTCTTTTTCAGAGAGGTCGGCGGCAGTCGCAAACACAAACAAAATCGCCCTTTCTGTTTCATAGGAAAAGTCACGACGGTGCCGTTCAGGGAAGTCATGGTGGTGCCGCTCAGGGAAGTCGCGATGGTACCAACTATGGAG

>Ca3:13854718-13855016

ATAAGAAAATTATACCAAAGGCAACAAGTGATATAAAACTCATAAATGCTGACAAAATTTTGGAAAGCAAAAACAGAACAGGTAGATCTTTCCTCAAGTATATTTTTTTAATATTCAAATAGTTTGTTCTAGAAGATAATGCATTTATATACTTCTTCGTTTGTTGTTAAAATTATAGAGGTTTAAGTAGGGAAGGAGGAGGATAAAACAAAAATACTCAACATAATCAAATTATAATGATAATATTGTGATTGTGATGATTTTGATGCAGAGAAAAAGTACTATTCATAATAGTGTT

>Ca3:14172817-14173908

TTCCCTATTGCAAACTATTGAGCATTGAGGATAAGTTGTGTGGATGAAGTGCATACAGCAGAAGTTCCACATGCAACATAATGGAGAAATATTTCCATGGATATTGGTGGCTGTGCCAACATGTAAGCAAAAGATTTGACACAACAAATGGTTGTTGTTTAAGAAACAAAAGGAAAACCATGTAATATGCCTTACAAGTAATAAGCAATGTAGCACGTGTCAGTGATATGTAGGAAGCAATTGTTTGTCACTTGCATCGGCAACAATCGATATCCTAAATAGGAATGTGAATAAATTTAGAAGAGGGAAGATTAATATTGATTCAGATTTTTCATAAAACATATTGATCAAGGTTGTTGGTGGAAAGTATTGGTCGGTTGATTGATTTCTCAGGAAAGTCTTCCTCTTTAGAGTCAAGTTGATTTAGAGGAGCTGAGATGCATTGTAAGTGGGTGGGAGAATTTCTCAGGAAAGTCGTCCTCTTCTTTTGTGCCAGTACAAGTTGATCTCAACTGCCTGATATTGAAGAGATGTAGAACCTTTGAAAGAATGAACATCAGGAAGCATTGTAACGAGCAGTTGTATATTGAAGAGAAAGTATGTAAGATGGTGGATGATGTCATGCAACAATGGATTTCGATACTGCCACCATCAGAAGATGTCACCTCCATATTCTCTTCAGACCACCAGCAGCCTTACTATTTGAATCAGCCACCATATCAGACGCCATCTCTAGACGCCTGCACAATCTCAATACCCTCCTAGTAGGACTCCTATGTGTTATGAAATTACATTTAATAGTTTGATGCAAGGAATACTACAACTTTTAGTAGAATTATGATGAGGCATACAAAGCTAGATTATTATGGTGGGGGAGTTGCATTATTAAATATTTATTGTGTTATTTTTATTTTGTTAATTTTGTAGGACTAGAAAATGCAGTTTTTTTTTATGGGGATTGCAAATGAAATTTTAAAATTTTGTAGGGATTAAAAAATTTATTTAACTCAAAGAAAAATAGAGTTCAAGGAAAGAAAAAAAGTTGTTAAATAATCATCCTAATTTTTGAAAAATTGTGTCGTTGAATGGAG

>Ca3:14203383-14204232

TAACATGTTTGAAGTACTTAAAGTTGGTTCCTCAATTTTGAATTTGAAGCAGCAATTGAATAATTGCTTTTGTTTTTCTTCTTAATCAATTGCTTTTGTTTTTTGATTTGTGAATCCTTTTTAAATCAAGCCACAATGGATGTGTTCGCTTTGGTCTTCAATTCCCGATCCTCTTTGAGAATTTTTAGAAGAGGGATGAATCTACATAAGCCTTTTATTTGTTCGTCGTTCAGTGATTCGATTTTCAATCTTACTCATTATTGATTTATTTAGCTATTATAATTAATCGGTGGGATGTGTCAATTGCATTACAAAGTACATTTATTGATTATTTTGATCATTTTGTTGACTGTGTATATATGGAACAAAGGATGTAGAGTGTGCACAAAAAATTTGTTACACCATTGATGTTCTCTAGTGTGTATAAGAGGTAAGAAAGGGGGGATAGAACACTGTAACGTGGTACAACACCCAGAGAGAGTGAGAGAGATTTGAATATTGGTTGGAAATCAAAGATAAATGCTATCATTAGCCAGAAGTATGTCATATTATGCCTGCATTGCTATCTTCCATTGTGAATCAACTAAAGCTTGTTTAGAACTTAGAAGTAGTAGGTTCAGCTTTTTCTAGGAAAATGATGGAATTTGTTTGAAACTCCAGACTTGGATCTGGTTTGCGTAGGACGAGAAGGAACACGAGATAATGATGACTTTAAGGGTGATAACTTGGTATCTCTAGGTGAAGATGATTTTCGTGTTAATCTCTATATTACTTTATCTAAACTTTGTTAAATGTAATTTATGATAACTTTATGTGTTGATTTGGATAGGGATACTCTTTTTATTATCA

>Ca3:14811897-14812766

GTTTGTGAATTGGAAAATAGGAAGTGGAATTTTTGAAGTAAGGGAGTAACTGATTAGGAGAATGTGGGGTACACTCACTTGCACATCACAACCACTGATGAGTTACTTCCTTGGTACTTTCAAAGCTGCGTACAACTGATTTGGTGAGTGGTTTTCTTGGAGTAAATTACCATTTTGTCCCTAAAAGTATAGGACTTTTGTCTCTTATTAAGTCAAAATTCGAAATTAGTCCTCGCATGATGTTAGTCACTTATACCCCTTACTTTATAATAATCAAATACTATTTTGATACTAAATTATATTTTGTAGATATTTTTATGATAGTAAATTGTATCTTTTAAGAATTATTTGGATTGTTTCAGAACTAATTTTAGTTTTTGGCTGAGTTAGAGATTAAAGTAACATTACTTTTAAGAACTAAAATGGTGCTTTATCTTGTGTCCAATGGTATAATCAATCTCTACTTGCTGTCAATTTTTATTTCTTTAATAATTTGTTTCTTCATTAGTTGCGTATTGCAATGTATGCATATAAAACTGATTGCTCCAGATATGTTGTTTCGGTGAGGTTGTGATCATAGGGGTGAGACCGAATTTCACTTGGACGAAATATTTGGCAAAATTGAAGAATTTATTAATACTTTGGTATCATTTCTTGATCAAGTTCAAAACTATGATTTGTTGGTTTTCTTTCTTTCAGTTTCAATTAATGCTTAACTTTCACCCTATTTCCTTAAAATTAACGAATGTAACAAGTTAATGGGCTGGAAAGAAGCTGGAAAAATGAATAAAACCAGAACCACTTCAAGGTTGGTGAACATTTGCACAAGCGAAAAAACATCAAATATATAAATAGTATTGTAATCAAAC

>Ca3:14814136-14814687

GTGGAGGTGAACAAGCCGTTTGGAGAATTCTATAATTGGGACTAGATGTGTGAACCCTGCACTAGGAACTACTACTATGTGATGTTTTCCCATTTAATTGATGGGAAATTATGATGTAATTATTACATTGGGTTTTCTGTTTATATTATTGAAATATTTGGCAAAAAACATGTTGGTAGGTGAAACTGTTGGTAGTTGAAAATGAGAAATTGTATGCCAACATCAAATTTATCCTAAGATTAAAAAAAAATTGTAATTTGGATCCTTGTCGACAAATTATTACGTTATTGGTGCACATCTGTTTTAAAGGATCAATATTATTGAACGGTATGGTTGTATATTATTAAGTTTCTTACTATTGACTCAAATCTTATAAAGTTCAACTTTCACACTTATTTGATGGATGATATATAAATACTGATAGAGTCACAGAAAAATAGGGATCTGTGAACGTAAAATGATAGCTAAAATGGAAATTTATTGATTTATGGAAGAAGGGAAAATTCTGATACAATGTATAATCCAGAGCAAAGCTCCTCCAGAGAAAATAG

>Ca3:18327953-18328213

ATTTGGAATAACCATGTTTTGAAGGTTATTTATATTTGGGTACTGGTTTGGTTAATTCTATTTCTGATTGAGGATACAAACAGTGTCCTGTATGCTATACTAATTCAAGCACAAGTAGTAAACTTTCACATTCCTAAAGATAAAAGAATTATAGAAACCAATCTGTTTTGCTTTTGCAGGATATGAACAGAGGAGAGGAGATTGCAGGTTTCTCTATCACATGTGTTATTAATCTTTAGGAATTTTTCACCAATTTCGTA

>Ca3:18328449-18329114

GTGTTTGGAAGAGAGTCTCTGGCTCTGCGCTCTGCACTAAATAGGAAAACTGCTGTTGAGGGATCAAACTTCAAAATTGGTACTCTAGGTTGGAATACTCTGTGTTTTCCTCTCTACTTCCATGATCTCAATGTTCCTTTATTCTGTCTATAATAGCTTTTGATTAGTAATCTATGAAGAAACAAGTAGTATAGTTTTTCAATGCTATTTTTCCTTGCCTTGTTCTTGCTCTCAATGCAGACTACATCAGTAAGGGAAGAACACAACAAATAAGGATATGATTCTTGCAGGCTGCATCTTTTGGTGAATTTTCTCTTTGAAAATTCACTAACCAAACCTGCTGACTCATCTATGAACAATGTGATGTGCATTTATTTTTTACTATCTAGTTTATGCCTTGCTTGTAGATTCTAAAGTCATTTTAGCTACACATTCACGGGTTGTAGATGATGAAGTTACTAAGAATAATAAAATATTCTTGAAGAGTGTGGAAAGTTAAAGATTCATGAACCATCTTTATTACTTACACTCCAAGAAAGCCAACAAATTTCGACACAATGCTATAAGTTAAAACACCATTCAAAAGTAGAATGTGGTCCACAGCCTATGAAGAATAACTATCTAGTAACATGTACATAACAGTTGGTTAAAGGGCATGTCCACTC

>Ca3:18530447-18530871

ACAAATGTGAAACAGCTGGTGGCTTGGCGTGAAGATAGAGAATGCATATTAAGCTATATAGAACGGTATATAGTGTATCACACTAGCAAAATAAACAAATTTCAAAAGGCACAAGTAGCAAGAAGCAGATTTCACACATCAGAAATTACTCACAAACAAAACACTAACCACACATCAAGCATCAAGATGGTCTCAACCAAAACTCAATCACATATAACACAACAAAATTTCACTCTTCAATAATATACCGCTTTAAACAGGACAACAAACATAAAAGAATGAGAAAATATCTTCTTCCGATGCGCGGTGGTGTGCGGATGTACTAGGTTCTCTCGTCCGGTTGTTGTTACAATGGACTTTAAGGTTATGGGTTGATGGGAAAGAATATGATGATAGATCTTTCTTCTGTTTGTTATTCTTTGTG

>Ca3:18531003-18532270

GCAAAAACGAAGAATGATTGTGTTCACTGTATAAACTTAAGTTCTTGATCAAACAAACTCATACAATTAAGGAAAAAGGGGGTTGGTCTTTTTGGTTGAGTATATCTATTGAGTTCAATTCTAACCATTGAACATAAAACTGAAACTTAGAATTTTTCTAAGTGTTTTTGGAATTCAATTTTAGATGAAATAGAATGCATTAGATCCAGAAGTGTGAACACTAGATGAATGGTTTCTTGACTAGAAACAAGAACATCATAATCTTGTTTTCTTGATTAAAAGCAAGAGAAAAACGTTTACTGGACCAGATGCAGGAACACCATATACCTGATTATTGGACTAGAGGCAATAGAAATCAGAGGCGCGATTGCTGGACCAAAGGCACAATTATTAGAACAAAAGGCAAGAGCAATTAGAGCATGATTTATTGATCAGATGCAGGAACACCAGAGACATGATTTTTTGACCACATGCAATGACCAAAAGCAATATTAATTGACCAGAGGCAGAATTACTATACCAGAAGCAATAACCAAAGCCAGGTTTACTAGACCAGAGGCAACAACAAGAAACAAAGGTAAATGACCAAAGGCAGACTATCAGCGACACTGACTATGTTAGAGTTAAATTTAACCAGAGGCGTTGACTTTTTCCAATGTAGACTTTTTTGCCATATGTGTTTATGTTTTCCAAAAGTTGACTTTTTATTGGATGTTTACTTTTTCCTAGACGTTGACTTTAACCATAACATTGACTTTTTTGGAAGTTGGCTTTTGTCATAATATTGACTTTCACCAGAGACATCTTATCAGAGGCAAGAAGTTCAAATGAAATCTCTAGAGACAAATTATCAGAGGCAACTGGTCAGAGGTAGTTTTGACCAAAAGCTTGATTGCTTCACCAAAGGCAAACTATCCCAAAGTCTGTTTTGCTTCATCAAAGGCAGAATTTCCCAAAGATTCTTCACCAGAGGCAGCCTTTCCCAAAGTATGTTTTACTGCATCAAAGGTAAACTTCAACAAATGCACTGAGAGCATAATTGAATCAGAATCAGATGTAGTCTTGGCAAACCTTCTTGTACCACCAAATGCAGAGGTAGTCTAATGTTTTGTATCATCAGATACAGTGTTTCAGATGCGCTTTTTCATCAGTTTTGAATGCAAAATTTTCATGACTTTTTGACTAAATCATCAGAGGATATATCCTAATATCCTACAACGTTGCTTGTGGTCTAGGAACTGCACACTTAAATAAAATATTAGTGCCT

>Ca3:18531311-18532270

ATGCAGGAACACCATATACCTGATTATTGGACTAGAGGCAATAGAAATCAGAGGCGCGATTGCTGGACCAAAGGCACAATTATTAGAACAAAAGGCAAGAGCAATTAGAGCATGATTTATTGATCAGATGCAGGAACACCAGAGACATGATTTTTTGACCACATGCAATGACCAAAAGCAATATTAATTGACCAGAGGCAGAATTACTATACCAGAAGCAATAACCAAAGCCAGGTTTACTAGACCAGAGGCAACAACAAGAAACAAAGGTAAATGACCAAAGGCAGACTATCAGCGACACTGACTATGTTAGAGTTAAATTTAACCAGAGGCGTTGACTTTTTCCAATGTAGACTTTTTTGCCATATGTGTTTATGTTTTCCAAAAGTTGACTTTTTATTGGATGTTTACTTTTTCCTAGACGTTGACTTTAACCATAACATTGACTTTTTTGGAAGTTGGCTTTTGTCATAATATTGACTTTCACCAGAGACATCTTATCAGAGGCAAGAAGTTCAAATGAAATCTCTAGAGACAAATTATCAGAGGCAACTGGTCAGAGGTAGTTTTGACCAAAAGCTTGATTGCTTCACCAAAGGCAAACTATCCCAAAGTCTGTTTTGCTTCATCAAAGGCAGAATTTCCCAAAGATTCTTCACCAGAGGCAGCCTTTCCCAAAGTATGTTTTACTGCATCAAAGGTAAACTTCAACAAATGCACTGAGAGCATAATTGAATCAGAATCAGATGTAGTCTTGGCAAACCTTCTTGTACCACCAAATGCAGAGGTAGTCTAATGTTTTGTATCATCAGATACAGTGTTTCAGATGCGCTTTTTCATCAGTTTTGAATGCAAAATTTTCATGACTTTTTGACTAAATCATCAGAGGATATATCCTAATATCCTACAACGTTGCTTGTGGTCTAGGAACTGCACACTTAAATAAAATATTAGTGCCT

>Ca3:18530553-18530871

AGGCACAAGTAGCAAGAAGCAGATTTCACACATCAGAAATTACTCACAAACAAAACACTAACCACACATCAAGCATCAAGATGGTCTCAACCAAAACTCAATCACATATAACACAACAAAATTTCACTCTTCAATAATATACCGCTTTAAACAGGACAACAAACATAAAAGAATGAGAAAATATCTTCTTCCGATGCGCGGTGGTGTGCGGATGTACTAGGTTCTCTCGTCCGGTTGTTGTTACAATGGACTTTAAGGTTATGGGTTGATGGGAAAGAATATGATGATAGATCTTTCTTCTGTTTGTTATTCTTTGTG

>Ca3:18531311-18531580

ATGCAGGAACACCATATACCTGATTATTGGACTAGAGGCAATAGAAATCAGAGGCGCGATTGCTGGACCAAAGGCACAATTATTAGAACAAAAGGCAAGAGCAATTAGAGCATGATTTATTGATCAGATGCAGGAACACCAGAGACATGATTTTTTGACCACATGCAATGACCAAAAGCAATATTAATTGACCAGAGGCAGAATTACTATACCAGAAGCAATAACCAAAGCCAGGTTTACTAGACCAGAGGCAACAACAAGAAACAAAG

>Ca3:18531801-18532270

AGACATCTTATCAGAGGCAAGAAGTTCAAATGAAATCTCTAGAGACAAATTATCAGAGGCAACTGGTCAGAGGTAGTTTTGACCAAAAGCTTGATTGCTTCACCAAAGGCAAACTATCCCAAAGTCTGTTTTGCTTCATCAAAGGCAGAATTTCCCAAAGATTCTTCACCAGAGGCAGCCTTTCCCAAAGTATGTTTTACTGCATCAAAGGTAAACTTCAACAAATGCACTGAGAGCATAATTGAATCAGAATCAGATGTAGTCTTGGCAAACCTTCTTGTACCACCAAATGCAGAGGTAGTCTAATGTTTTGTATCATCAGATACAGTGTTTCAGATGCGCTTTTTCATCAGTTTTGAATGCAAAATTTTCATGACTTTTTGACTAAATCATCAGAGGATATATCCTAATATCCTACAACGTTGCTTGTGGTCTAGGAACTGCACACTTAAATAAAATATTAGTGCCT

>Ca3:18625053-18625434

TTGGTATGGATCATTGAAGACTAGCATTAGTGAGGCATTCTGTGATATGACAAATTTGGAAATCAGGGAATGATGATATTTTCTCTACAAAGGTTTGTTGTGTTGACAAAGAGTGGATCAAATGCAACATTTCTTCTTGGAATGGATTTTGGCTAAGTCCTTAGGATATCATCGTGTACATTTTATGAATGGGTATCGCGTCCCTCCTTATGTTTCCTCGTTTGGTTTATGTTGTAATGGATTGGAGTAGGTTTTGCCTTATTTTTGCTTTGGTTATCTCGTTTGGTTTTTATTGTAATAGGTTGGAACATTCACTGTACTCATTTTCAATATATATATAGTCTTCCTTACCATATATATAAAAAAAAAAACTATTCCATG

>Ca3:18625019-18625434

ATTGATTTTGATTTTTTTTGCTCATTTGGCAAAGTTGGTATGGATCATTGAAGACTAGCATTAGTGAGGCATTCTGTGATATGACAAATTTGGAAATCAGGGAATGATGATATTTTCTCTACAAAGGTTTGTTGTGTTGACAAAGAGTGGATCAAATGCAACATTTCTTCTTGGAATGGATTTTGGCTAAGTCCTTAGGATATCATCGTGTACATTTTATGAATGGGTATCGCGTCCCTCCTTATGTTTCCTCGTTTGGTTTATGTTGTAATGGATTGGAGTAGGTTTTGCCTTATTTTTGCTTTGGTTATCTCGTTTGGTTTTTATTGTAATAGGTTGGAACATTCACTGTACTCATTTTCAATATATATATAGTCTTCCTTACCATATATATAAAAAAAAAAACTATTCCATG

>Ca3:18624630-18625434

ATAAAAGTAGTATTTCAAACTTTGTCATTAGGATAAATAAGAATTGCACAATGTTTACCTCTACTTCTAACCAAATAGTCATTAGGAGGATTGTGAAGTGGATCCTCTGAACCAAAATGAAGTGAGATAGAGAGTGTGTAAAGAGGGTTGGTGGGGAAGATCCACATCCGGATGATTGTGTAAAAAGTGAAGAAAAATTGAGTGTGGATAATATATCATATTGCAATGAGGTTTAGATATATCCAATAGATGAAACTTAAAATAATGAGTAGGTCTATCAAAGAGTTGGCTACACATCTTTTTGTTACTTTTGATTTTTCATCACAAGTTTAGTTTAAAATCTTTTGTTGACTTGGGTGTGTGATGATGGCATTTTCACCCTTGTTCAGATTGATTTTGATTTTTTTTGCTCATTTGGCAAAGTTGGTATGGATCATTGAAGACTAGCATTAGTGAGGCATTCTGTGATATGACAAATTTGGAAATCAGGGAATGATGATATTTTCTCTACAAAGGTTTGTTGTGTTGACAAAGAGTGGATCAAATGCAACATTTCTTCTTGGAATGGATTTTGGCTAAGTCCTTAGGATATCATCGTGTACATTTTATGAATGGGTATCGCGTCCCTCCTTATGTTTCCTCGTTTGGTTTATGTTGTAATGGATTGGAGTAGGTTTTGCCTTATTTTTGCTTTGGTTATCTCGTTTGGTTTTTATTGTAATAGGTTGGAACATTCACTGTACTCATTTTCAATATATATATAGTCTTCCTTACCATATATATAAAAAAAAAAACTATTCCATG

>Ca3:18656062-18656422

ATTTGCATCCCCAAATACAAGCACATTGGACATTCTTTATGGAGAGATAATTGGAGATACAAAGGAAGGTGAAAGGCATTTGATATCATTAGAGGAAGATGATGGATGAGACAATCAGTTAGTTAGATTACTTACAAGGATCCAAGCATAAGAATTAGGAAATAGAAAAAAAATACATTTTCCATTTTCTTAGATGCTATAATGCTAAGGGGCTGAACATCTGATCTTTGTGCTGGAATGATTTTGTCATATTGTTTCATGACTTAGATGTTGAATTACTTTGATTTTCATTTATGATATGTCAACTTATATATTGGTTTATGTAGGTTATTGGACCAAACTTTGTAATCAATTCTTGTG

>Ca3:18821946-18822212

AAACCAAGCATCCAAGCGCAACGACTTTCCGGCAACGCTTCTTCAACTGCTCCGATGACGACGCACCGACAATCTTCTCTTTCCGATCCTCTGCATGCAAAAGACGATCAAAACTCTTACATCTGTCCCTATTCTTCTTCTTCAATCTACTTTTTTTTTTCTTTCTATTTTTGTTTTTTTATGAAAATTCTATATATGGTTCCTGTGACTTGTTCAGGTGAAAGGAAATCTGAATCTGCAACTTGTTAATGAATCTGCTATTGAGG

>Ca3:18825610-18826274

TCCTGGGCCAAGTGGCTGTGTGACCCCACTCAATTCTAGTGGATTGAAAAGTGCAAAACCAGGCTGCTGAAATGTGTTCTTGATGTTGTGGTCCTCCTGCTATTCATTGCTGATAATATCTTCCAGTACATGTTTCCTCATTCAATGCAGCTATGAAAAGAATATGCCAACAATTCTTGCTGGTTAGCATTCTGGTGGTTGGTTTTCCTGGAACACAAAGGAGACAAAGCTGGAATGAAGGATTTCTCAAAGAGAAAATTTTATAGCGGGGTATTGAGAATAGGTTTGTTCAAATCAAACCTTTTTTTCCTCCCGTTTAAACTCAATACAGTCATTGGATTTATTCTTGAGTCAGTTACTTTTATGAAGTGTGGAGTATATGTATACATGTGATGGTGGTGGTAAAGTTTTAGGCCTAAGATGATGAAGAGTTGTTGCCACGTGTAATGTGTCAAAGTCTCACTGTATTTGATATTTATTCAAGATGGTTAGATAGCATAGGCTGCTGGATTCTTTTTAATTGTTTGCCAAAAGCATTTGTATTATCGTCCGAGAGGTATGCGCTATATTTGGAATGATCCATGCCAGATGAATATCTTACCATGTGCCATTAATGAAAAAAGTTGTATTTTGTTTATATACAATGGTTACATATAAATGTTCT

>Ca3:18825760-18826274

CTATGAAAAGAATATGCCAACAATTCTTGCTGGTTAGCATTCTGGTGGTTGGTTTTCCTGGAACACAAAGGAGACAAAGCTGGAATGAAGGATTTCTCAAAGAGAAAATTTTATAGCGGGGTATTGAGAATAGGTTTGTTCAAATCAAACCTTTTTTTCCTCCCGTTTAAACTCAATACAGTCATTGGATTTATTCTTGAGTCAGTTACTTTTATGAAGTGTGGAGTATATGTATACATGTGATGGTGGTGGTAAAGTTTTAGGCCTAAGATGATGAAGAGTTGTTGCCACGTGTAATGTGTCAAAGTCTCACTGTATTTGATATTTATTCAAGATGGTTAGATAGCATAGGCTGCTGGATTCTTTTTAATTGTTTGCCAAAAGCATTTGTATTATCGTCCGAGAGGTATGCGCTATATTTGGAATGATCCATGCCAGATGAATATCTTACCATGTGCCATTAATGAAAAAAGTTGTATTTTGTTTATATACAATGGTTACATATAAATGTTCT

>Ca3:18821811-18822070

ATTAAATAGCAACTTGTTAGACAACATTGCCAAAACCCCGGTATAGTGACATTATTTTCTTATAATATACTTTCAAATTTAAAGTACTTTTGGTAAAATAAGAGAGGGTGTGATGTTTTGCATTGCATCACTCAGAAACCAAGCATCCAAGCGCAACGACTTTCCGGCAACGCTTCTTCAACTGCTCCGATGACGACGCACCGACAATCTTCTCTTTCCGATCCTCTGCATGCAAAAGACGATCAAAACTCTTACATCT

>Ca3:18822163-18822525

GTGAAAGGAAATCTGAATCTGCAACTTGTTAATGAATCTGCTATTGAGGGTACACCATACTTATGTTAGGATCAGATTAATCCAAAGGTTGGAAATATTTGTGGTCCATGCAATTGAATTGGTTGCCACATTTTTCATCATCATGCCACCACCAATAGATGGATACCATGTTGATTTCCTATACCTTACTATATAACATTGAATTTCTTCACTCACTTCAATTACCTAGGGTTTCACTATCAACTCTCCATAACCTTCATAAAATTGTTAAGGTTTTTCCTTTTCCTTTTTCAGCTATTTTGAGGTTTTTACTTTACAGTTGATCTTGCTCTTCTCTCACTCATTCCACTATCTAAATTCAG

>Ca3:18821836-18822525

ATTGCCAAAACCCCGGTATAGTGACATTATTTTCTTATAATATACTTTCAAATTTAAAGTACTTTTGGTAAAATAAGAGAGGGTGTGATGTTTTGCATTGCATCACTCAGAAACCAAGCATCCAAGCGCAACGACTTTCCGGCAACGCTTCTTCAACTGCTCCGATGACGACGCACCGACAATCTTCTCTTTCCGATCCTCTGCATGCAAAAGACGATCAAAACTCTTACATCTGTCCCTATTCTTCTTCTTCAATCTACTTTTTTTTTTCTTTCTATTTTTGTTTTTTTATGAAAATTCTATATATGGTTCCTGTGACTTGTTCAGGTGAAAGGAAATCTGAATCTGCAACTTGTTAATGAATCTGCTATTGAGGGTACACCATACTTATGTTAGGATCAGATTAATCCAAAGGTTGGAAATATTTGTGGTCCATGCAATTGAATTGGTTGCCACATTTTTCATCATCATGCCACCACCAATAGATGGATACCATGTTGATTTCCTATACCTTACTATATAACATTGAATTTCTTCACTCACTTCAATTACCTAGGGTTTCACTATCAACTCTCCATAACCTTCATAAAATTGTTAAGGTTTTTCCTTTTCCTTTTTCAGCTATTTTGAGGTTTTTACTTTACAGTTGATCTTGCTCTTCTCTCACTCATTCCACTATCTAAATTCAG

>Ca3:18821856-18822212

GTGACATTATTTTCTTATAATATACTTTCAAATTTAAAGTACTTTTGGTAAAATAAGAGAGGGTGTGATGTTTTGCATTGCATCACTCAGAAACCAAGCATCCAAGCGCAACGACTTTCCGGCAACGCTTCTTCAACTGCTCCGATGACGACGCACCGACAATCTTCTCTTTCCGATCCTCTGCATGCAAAAGACGATCAAAACTCTTACATCTGTCCCTATTCTTCTTCTTCAATCTACTTTTTTTTTTCTTTCTATTTTTGTTTTTTTATGAAAATTCTATATATGGTTCCTGTGACTTGTTCAGGTGAAAGGAAATCTGAATCTGCAACTTGTTAATGAATCTGCTATTGAGG

>Ca3:18821880-18822525

CTTTCAAATTTAAAGTACTTTTGGTAAAATAAGAGAGGGTGTGATGTTTTGCATTGCATCACTCAGAAACCAAGCATCCAAGCGCAACGACTTTCCGGCAACGCTTCTTCAACTGCTCCGATGACGACGCACCGACAATCTTCTCTTTCCGATCCTCTGCATGCAAAAGACGATCAAAACTCTTACATCTGTCCCTATTCTTCTTCTTCAATCTACTTTTTTTTTTCTTTCTATTTTTGTTTTTTTATGAAAATTCTATATATGGTTCCTGTGACTTGTTCAGGTGAAAGGAAATCTGAATCTGCAACTTGTTAATGAATCTGCTATTGAGGGTACACCATACTTATGTTAGGATCAGATTAATCCAAAGGTTGGAAATATTTGTGGTCCATGCAATTGAATTGGTTGCCACATTTTTCATCATCATGCCACCACCAATAGATGGATACCATGTTGATTTCCTATACCTTACTATATAACATTGAATTTCTTCACTCACTTCAATTACCTAGGGTTTCACTATCAACTCTCCATAACCTTCATAAAATTGTTAAGGTTTTTCCTTTTCCTTTTTCAGCTATTTTGAGGTTTTTACTTTACAGTTGATCTTGCTCTTCTCTCACTCATTCCACTATCTAAATTCAG

>Ca3:18821887-18822250

ATTTAAAGTACTTTTGGTAAAATAAGAGAGGGTGTGATGTTTTGCATTGCATCACTCAGAAACCAAGCATCCAAGCGCAACGACTTTCCGGCAACGCTTCTTCAACTGCTCCGATGACGACGCACCGACAATCTTCTCTTTCCGATCCTCTGCATGCAAAAGACGATCAAAACTCTTACATCTGTCCCTATTCTTCTTCTTCAATCTACTTTTTTTTTTCTTTCTATTTTTGTTTTTTTATGAAAATTCTATATATGGTTCCTGTGACTTGTTCAGGTGAAAGGAAATCTGAATCTGCAACTTGTTAATGAATCTGCTATTGAGGGTACACCATACTTATGTTAGGATCAGATTAATCCAAAG

>Ca3:18825736-18826274

TACATGTTTCCTCATTCAATGCAGCTATGAAAAGAATATGCCAACAATTCTTGCTGGTTAGCATTCTGGTGGTTGGTTTTCCTGGAACACAAAGGAGACAAAGCTGGAATGAAGGATTTCTCAAAGAGAAAATTTTATAGCGGGGTATTGAGAATAGGTTTGTTCAAATCAAACCTTTTTTTCCTCCCGTTTAAACTCAATACAGTCATTGGATTTATTCTTGAGTCAGTTACTTTTATGAAGTGTGGAGTATATGTATACATGTGATGGTGGTGGTAAAGTTTTAGGCCTAAGATGATGAAGAGTTGTTGCCACGTGTAATGTGTCAAAGTCTCACTGTATTTGATATTTATTCAAGATGGTTAGATAGCATAGGCTGCTGGATTCTTTTTAATTGTTTGCCAAAAGCATTTGTATTATCGTCCGAGAGGTATGCGCTATATTTGGAATGATCCATGCCAGATGAATATCTTACCATGTGCCATTAATGAAAAAAGTTGTATTTTGTTTATATACAATGGTTACATATAAATGTTCT

>Ca3:18821893-18822212

AGTACTTTTGGTAAAATAAGAGAGGGTGTGATGTTTTGCATTGCATCACTCAGAAACCAAGCATCCAAGCGCAACGACTTTCCGGCAACGCTTCTTCAACTGCTCCGATGACGACGCACCGACAATCTTCTCTTTCCGATCCTCTGCATGCAAAAGACGATCAAAACTCTTACATCTGTCCCTATTCTTCTTCTTCAATCTACTTTTTTTTTTCTTTCTATTTTTGTTTTTTTATGAAAATTCTATATATGGTTCCTGTGACTTGTTCAGGTGAAAGGAAATCTGAATCTGCAACTTGTTAATGAATCTGCTATTGAGG

>Ca3:18915382-18915709

CTTTACACACCATCTTACTCACCTCACAAACTCAGCTACCTACTTTCAAAAACAAAACCTCAACTACCCTATCCTTCTTTGTTTCTTTGAATCTCGTTTCTTTGTTTTTCTTCTTTTCATCTTTATTATATATTAACTACAACCTCTTATCCACATATCCTTTCATTCTTCCACATACACAAACACCATAGTAACCACATATTTCTTCCACAAATTAATTTTTATTATATATCATTTTAATTACCCTTTTTTTTACAACAAAAATTACATTTTACCACAAGATTAATGGAGGAGTCGGGAGAAAATTGGCCTTCTGATTCTGATTTG

>Ca3:19486475-19486771

GTAAAACAAATCCTAAATTGAAACAAGGCATAGGCAAAAACCAGAGCGGCGGCTAGGCAATGAGGGTCGCAGATCAAAGGTTTCAAAAGAGAAAAAACAGAGCCATGCGAACTTCATCATCAAGACCGTAAATAAGAGAAAAAACCGAGCCATGCGAACTTCATCATCAAGACCGTAAATAAGAACGGCGTTGGAGTGAGGCCGCTGGATTAGGTCGTTGAGGAAGAAGGGGAGTCAGACGAACGGAGTGACCAGGAAACACGGTCGGCATTTCGGCGACGACAACGGCGTTCAAG

>Ca3:19788764-19789027

TCTTTCAAGAACCAGTGATTCTGTCATTTTTTGCTGCTTTTGCTGAAGGATAATAAAGCCTGATTCTCTATCCCAAAATGCTAATGGAGACTGTAAATTTATTTTTGTTGTTGATTTTGGTTTGTGTCTCATGATTTCATGGCTTCTTCCAGTTTGTTTGCTGAACCAAAAGCAAGATTAGGATACTAATTATAGTTTATGATATTGTGCTACTGATTATCAGTCTGTTTCTATTTGAAACTATTGAAGGCTGAGTACACATT

>Ca3:20185100-20185786

GTTAAGCAGCAAAAGCAGCCCATGGGATATGATCTGGTATAAAGATGGAGCAGAAGATTGATTTGGAAGAGCTCCAATATGAGGATACTAAAATGTTCTGCACCCTATCCTGCTTACCAAATCCTTGCACTTTAAGAGAATCCAATAGCAGTACAGGGCTATAATAAATTAATAGAATGTCACAGAATTTCCTGCAAACCAAATGCCAAAATCTTTCTACTACAAGCAGAGAGTTTGAATGGCTAAAAGAGCACAGGGATGGAGAAACTTGTTGAATGACCGGCATGCACCATAGAAGCTAAGTCTTTTAACTTTCTGGCATTTTTTGGAAATATTCTTTTATCAGGAGATTGTGCATTCCTATGCATATTTCCGTTGCTGAAGGAAGCTTCATGTTAGAGCCTGATTTCTGGTATTGGCATGTGCTCTGTTATTTGTGGTGTATATGTGAGGTTATAGGATCTTAATTTCTTTTCTAGAAATATATCATTATTGTTGATGTAATATTCGATGACCAATTGGTTAAAGGGTCTAAGTTTCTTTGCTTTTGGTGATATACAATTTAGTAAATGTAAATGTGTTGCCTTGGTTCTCGGAAGTGTCTGTAAATAAATAAATATCTAAATATGTTGATTTCTTCACAAATCATCTGTAAATAAATAGCTAAATATGTTGATTTCTTCGCA

>Ca3:20180418-20180975

CTAATGAAGATGATGCTTATCTTTGATTCCTATGCTAGCACGTGCCTTTGAGTTTTAATATTTAATTATTTAATTATTGTGTTAAAGACTTCAATGTGCATCATCTTTATTTCCGTGATTTTTGTTGTTATTTCAATGTATGTTTTAACTAAGAGTAAGACTCGTGGATTCTTATTACTAATTATGAATTTTTCAGAGTTTGAGTCATTTGTTAATGTTGCACTAAATATAGGTTTACATAGTATTATTCATGTAATTTGTTCAAAAAAATAACAATTATCCCGCCTATCTCGGAACGCGCTATCCTGCTATCCCACTTTTGGGGTTGGCCGCCACGCACCATTATCTGGGATTAACTATCTAGGGATATTAGTTTCCACGTTATATCATTGAAATGCGCATGATAGTGGATATCAAATTTAACTTGATGAGTATCTCAATAATGTCTTATCTTTTGAACTAATCAACTCAATAAGAGTATGTGGATAACATAATTTTTTCGCTTTTAATGATTTTATGCTTAAATGGTTTGTCACTAAACATATATATGATACGAC

>Ca3:20378810-20379394

TTTTTCAAGAGTGCTGGAATTCATTTGTTGGCTACTGGCTACATCATCTAGATTTTCTGGTCTTGGAGATAAGTTTTTGTTTTAGGTTGCAATGTTGTTTTTCTTCTTTTTCTGTATTTACAGTATACTTTGCTGTGAGGTACTTGGGCACAACTAGTAGTTTGTTTTTTTGTTACTTTCATAGTTTTAGTTACACTTTTTATTGATGACTTCTGTTTGTTTACAAAAACTAGTAATTGTATTTTTTGAGCTAAGAATACTAACCTTAGAAAGGAATTAAAACTTCAAATTAAAAATTAAAAGAGCAAAAAGAAAGAACAAAAATTTGTTCTTTCTAATTGATGTATTACTATTGAATTGTATACATAATATTTATATACAAATTGATTGTGCAAAAATTTTCTATCCTCGGAAGATCCCTAAGATATAATCACAATGTTCAAAAGACAAACTCAACAAACTCTGGTTTTAACTAAGCGTCTAAATACAGATTGACTGCTGCTTGTTGCTCTGAGATTTGTGCAGTGGATTGTCTAAATATGAAGGAGTTGCATGCTACAATATGTCGTTTTATTATAAGGTTG

>Ca3:20380280-20381479

AGGCGATAGTGCATATCAGGGGTGATAGGTGATAGTGCATATCAGGGTGATAGGCGCTACCGCTTATAGAGGGATAGACGCTGCCGCCTATCGGGGGCAATATGCGATAGTGCCTATTGGGGGCGATAGGCGCTGCCTCCTATCGGAGCGATAGACGCTGCCTCCTATCGGAGCGATAGACGCTGCCGCCTATCGGGGGCGATAGACGCTGCCGCCTATCGGGGGCGATAGGCGCTGCCTCCTATCGGAGCGATAGACGCTGCCGCCTATCGGGGGCAATAGGCGCTGCCTCCTATCGGGTTAATATCGGGGCGATAGGGGCTGCCGCCTATCAGGGCGATAGGGACTGCCGCCTATCGGGGCGATAGGCGCTGCCGCCTATCGGGTTGATATCGGGGCGATAGGCGCTGCCGCCTATCGAGACGATAATGCTTATCGGGGTGATAGGGGCTGCCGCATATCGGGGAGATATACAGTGCCTATCGAGGCAATAATGCTTATCGGGGCGATATGTGATAGTGCCTATCGGGGGTGATAGGCGCTGTCGCCTATCGAGTTGATATCAAGGGCGCTAGGCGTTCCCGCATATCGGTTGATAGGCGATAACGCGTATAGAGGGCGATAAATGTTGTCGCCTATCGTCCGATAGCTGCTATCGCCAAGCGATAGACGATAGCAACTATCGATTGATAGGCGATAGATGACATCAGCTATATAGTCTGATAGACGCTGTCACCTATCGCCTGATAGGCAGTGGCTATCGCATGATAGTCGACAACGCATATAGCCCGGTAGCTACTATCGCCTGATAGGCAATAACATCTATTAGCAATAGGCTTTGTTGTCTATCGGGCGATAGACGATAGCCGCAATTGACTGATAGGCAATAATATTAAAAATAATTACTATGAGGTGACAATCATAGAGTGAGGGAATTTATTTTAAATATATATAATAGATAAATAATAGATTTGTAATGAGATCATAAGTCGACTAATGGAAAATTGAATTTGTTACCTTTTTGTATTACTCTTATAGTTTTTTAGAAACTCAATTTTACATTAAATATATGGTTTTCAAAATGACAAACACAACTATTTAGATTTCAGATTTCGGATACAAGTTGGATTTCAGGATAAAAGAATCCAACTAGGTATGTAAAAAAAATCACTCATGTGGGAGTTAAAAAATACAAGCTG

>Ca3:20549873-20550186

TTTGCTTTTGGATGGTACTGTTACTGGAAGCCATCTAAATTTCAAAAAACTGAAGCTAAATTGCTGAGTAATATTCAGTGGCAGTGTGGGAATTCATATGAAAGAAGCCATGGGTGAGCACTCACTACTGATCGACTAGTCATAAATATATCAACCATTCATGTGGTGTAATTTAGGATTGCAATTCAGTTTTTTCTACTTTCGGTTCCTAGAAATTTCTTGGTGTTAATTTCCTACATCTTTTCAGTTTTCTCATGTTCCTAGAAATTTCTCATGGAATGTTACACTTTCCAGTGGATGATTTTGTCCCCAT

>Ca3:20759815-20762228

TGATGATGAGACTAAGAAGAATATTTGTTTTCTTAATTACGAATCGATAAAAATTGAATTTTATAGATCGTTGCAGTAAAGTCACTATATATATGCCAATATTTGAAATCAATGTAAAATACAAAAAATGAAGAGAAAAAAAAATCATGTTGGATAAACATTGAGAAAACTTTAAGAAAATTATTAAGGTAGTGACGGATTTGAATGTCGGTGTGTTTTTGAAAATGTTATGGTTGATAAACATCGACATAACTAGTTGATTGTGTCATAATCAAGAGAACACTGATATTTTAAGAGCAATCATTATGGATCATATGTGTTGATAAAAAATGACAAATAGTGTGGTTTTGATTGAGTCAATGTCTAACTCGTAGGTTTTCCTCTCTTCTCCTTAGCTAATCACTTTCACAAAAAAAGAAAGAAAAAAAACTTTCATGTGGAAAATCTATATATGCTACTTAAATATCACCATGCAAGCATAATAATAAAAAATTGAACTCATTATTCAGTTTCATCTAATTAATTACTTTTTCAACAAAATATAATGTCATTCTCAATGGAAGCACTAATTCATGTTAATCAATATCCTCACCTTAATTCATTCTCACATATATGAATATAATTATTGAAAATGACCAAAGTGGTATATGAGTCAGTGGATGTTTTATCCTCATAATAAAAAGACTCATTTGTTGGGCGCACTTTGAAGGAACTACTTACATAGTGACATAAACTTTTCATGGGTTAACTACTTGAAAAATCTTATTAAAAGTTGTCATTCAACTTTTTACATTAAATTTTAATTTATTCATTTAATAATTTTTGATCATTTTCTAGTACTTATAGTATCCTAGAGATCATGCCATACCATGATGATATTACCAATTAAAAGGAAAACTTTGATCAAGACAAATGAAGAGAAGGAATTTTGACATTTTATATTGATATATGCTATTGGAAGTTGAAAAGGTAATTAAAAAAAAATGAAAGTTTTTCATTTTCCACTTGTTATTGTACGTGCTTGTTTCCACAATACATAGTATTCTTCAGTTTCTTGTTCATCATAAAAGAAAAGATATTGTTCATGGATTTCTTGTGTGGCCAACTTTTTTAGTGATATTTATGGTTTTAAATTGCATTAGTAACTATAATTGTAATTGTAAGGTTGTGATTTGAGAAGACTTCACAACTACATCACCATCACAATGCTATGGGTTCAACAACCATAATTTTAAACTTTTTAATCATTTCAACTATGGAACTTTTGGTAATCCAATTATTTATTACGTTCATATTTACTTTGCTTAGCATCCTATGTCTTGAGACAATAAAGGTAGTCAAGTGAGATAACTCGTCAAAGTTGCATAAAACAACTTTAATTTCACTTTTTGAAATGTTGGTATGATAATTATTCCCTTTCTCTAATTTTTTAAGCTAGTTAACATATGTAATTATCATGTTATTGCTACAAAGTATTTACAAACTTTCTCTAATTTTTTAAGCTAGTGAACATATCTAATTATCATGTTATTGTGATGAAGTATATATTAATCGACTTTTATTGATATTAATCTTTAACAACGAATTTAATTGACCACTTTTAGTGTAGTATTTTCTCTCTATTATATTTTTTATCCATAGATTTTAAACTCGATTCCTTATTCAAATCTAATTCCACTTAGATCAATGTAAAATGGGTAATATGTATAACTGTTTAGTTGATAAACACATGATTTAGTGAAATTTTTACGACTCTTCTTAAAATATAGATAGATTCCTATTCACTAAGATGTTAAATTCGAACCATTATTAATAAAATTTTACTTATTAAAAAATGTTTAATTTTTTAGTTGGTAAACTTAATAACTTTTTTCTCTCATTAATTGATTCTTAACATAAAATTAATGGGAAAAAACCGACTTAAGCACATTTATAAAACATTTTTTGCGACAAGAATTGATAGTGATGTTGAGAAAATTGGAAATGGAGCCAAAACAACACAAAGCTATCAAGAGACAACACAACCATATGATGCTTTGTTGCCGTACACTTGGTTAAAGGAATTCTCTTTAATTAACCACACAAGAAAAGTTTTTGAGCCAACTTTGGAAATATGTGGTGATATATGTATGTGCCTTGACACCTATAGTTTCAATGAGAGTGTCTATTTTGTTGGCATATATAGCATCTAAGAAAACATAGGGTAGTGGATACAGCTTATGATGGGAAAAGACTTATTGAGTTTACCATTATTATTGTGCATATATCAATTTAATATGCCAATTTATGGTAAATTACATTAACTCACTCACATTTTGGAACACAATATGGGACCATTTCTATTTCCATTAAAACTCAAATCTTCTTTTATGAATTCCTATTGCTATATTGATGTCATAACATTGTGTCTAGC

>Ca3:20952343-20952729

AGTCACAAGGTCGACAGTATCGACGCGCAAGTGGTGAAGCAAGCTGTGTTGCAAGCTATCAAGAACACGGACTAATCAAAATATATATTAATTCCAGTACACTTTTTTGTCTTCTTTTATGATCTTGAAATTTTCACATATATATATATCCAAGTGTTAATCTGCCAAAAATATGAGGTGCCTACCACTTCGATCAATGGCCCTTACATATATTCATATGGCTTATCAGCATCTTATAATTGGATAGTGGAAAACAAAGAATGAATGGACACACTTCTTCTCCCTCATTATGTTTTTGTGGTTATTATTTATTTATTTTTTGTATAATATTACGGTTAATTGTTGTTAATATTTGAGATAGTGTAGTTAGTTTAGATTGTAGTTGG

>Ca3:21315997-21316270

ATTTCAATTTTTTTTCCCTTGTATTTTTCTGCTAAATTCCTGGTAGTTTTGTTACTGCTGCATACTATGTTCTAAGTTCCATGACATTTTCTCAAGATTATTATATATATCTTGTCGGATATCCTAACATTCATGTAATCTGTATGTTGGATGGTTTGTATGATTAGTTTATCTGTAAGATATATATGATTGTCTTAAGTAAATGTCCACGGAACAAAAAATAATATCCATAGATTTAAACAAATGGGATTCTTGTATTTCGATCCTCTCTTC

>Ca3:21318262-21318614

AGATCATTTCTCCAACACTCTACGCAACAGCATCCGCTAGTGTCATTAGTCTTGACGATTAAAGACATTTGTTTCTATTTTGTTTAGCATCATTGTATTTGAAGTAAAATTGTTGAACTAAATAGATTAGTTGAGTTTAGGGGTCGTACTTTTAGAATATTGAGGAATTTGTTTCTGTTCACCTTTTAGTTACATGTGTGTTGGGCAGATATTTTTACAAAATTCATTAGCCTATTAAGGTTTGGTGATGTGCTGAGACTAATTAGACATTGTTGTGATGATGTTTCTGATTAATTATGATTGATCAACATTGTTGTGCCAATGCAAGGCCGTATGTATCTGGAAATGCTAT

>Ca3:21526808-21527225

ATGACGCTGTCAGCTCACAAAATAAGACTTGAAAAGAAGTAGTCTATTGTTTTATGGCACATGAAACCTGGCCATTGGAGTTCCATAAACGTGAAGGTATGACCAAGAAATAGATTCTCAAGGAACCTTCTCATTTGAAACTTTAAGTTTCGAGCTAACGATTCACTAGTTTTAGTCAACTCATGTATATCTATCTATATGCACGAGGGACGGTGTTAGCCCAATAATGTTTGGACAAGAAAGCGTTAGGAATCCAAAATTTGTATCGGACAGAAGAACACTTCTATTGAGAAAATATGAAAAAATAGGAGAAAATCTCTACTCCAAAGTTTCCCTTTCTACACTAGTCACTACTATATTAAAAAATTGTTTACAATACTCAATTTATCTCTAATTCAAGATAATCTTAACTGTCCC

>Ca3:21560690-21560918

CCTCAAATATTTGCTTTGAAATAGATGGAAGGAAAGATTCACTTGCAAGGAACTGTCAGTTGATACTAATTACTGACAATTTAACTGCACAACTAAAAGCTGTACAAGCAAGTTATGAGAATGTTTTAAACTGGTATGGGGATGCACTAACCCCTACACCAGGAACTGCGTTTCGGAGCTATTTACGGGTTGCAACCTATATCACAGAGTAAGAACGTTGATTGCAAG

>Ca3:21561783-21562715

AAAATTGGAAAAGGTGATTAATTCTTGATGATACAAGATAGGATTTAATTTCAAATAAAATGATTTTAGGGCAGAATTTTGTTATGACCTTAAGATGTGTTTTAAGCACTCTTTAAAGTGGAAATTGTCTTGGCTTGGAAAGAAAGGGGAAGACAGTCTCCTTGTGTGTTCCGGCTAAATAATTTGATTGAGACAGTTTACAAAATGCTGATCAGGTTCGTACCTGTTGTCGGATTTGGTTGAGAAGTTTATAACTTTAGCACTCAGTTTTTGCCATGATAACACAGAAGAGACCAATGGGGGTTAGATGTGATGGTTGAGCTATTAAAAGCAATGCTGACAAGAATTGAAAGTTCTTTCTTACTTCTTAACTAATATGCTAGCAAAGTGGCATCATCAGTATTCTATCTGATTGGAGAGGTCATCTGTCTCAGAAAGTGGTCTTGTCATCGGGCTTTGAGAGGAGATGATTTGAAGGGGTTTGTTTTTATTCATAATACAAAATCCTCTTATTTTAGGGGAATTTGAAGGGGTTTGTGGGATTTAAATCCTCTTATTTTCATATTGTTATAATATTTTATAAATTAAAAATATATTAATCATAAACATTCTATTATTATTTTCTACAAAATCACTTTCTAAAGAAATGTAAAAAAATTCTCTTAATTTTTCTATTTTCTTCTCCCTCCATTCCCCTTCCCTCTAAACTCCCAACCAAAGCCTAAGAAACCAAAAACCCTCGTCTTCAATCATTCCAAGCAGCAGTTTGAGTTGATTATTTTCACTGCAGTGTAAGTGTAATGATAGAGAATGAAGTGAGATTGTTGTGTAATCTTGAAATGTTTATACTTCTTGATAGGGCAGAAGTTGACTCTAGTGTATATGATTCCATTGGGATTGAAATCCAGTTTTAAATGTATTTTGAATGCAAC

>Ca3:21645867-21646076

CGAAAATCGAAGTATAACGAATACAAATTGTCTACCTTTAATTACTAAAATATCCATGCTTATGATTTCAAAATTACAATAAAGCCATCCAAATCCTAGTAGTTCTGTTTGCTTTCTCTCTTGCGACTCCTCCTCTCCATTCTGGCCAAACACTGCAAGCCGTCGCCTTCCCTTGCCGCCGCTATCGTCAGCCCTTCCACCGCTAACTG

>Ca3:21649546-21650285

GAAAATATGCTGGCTGAGCTAATTACAGCGGCGAATCTTAGCTGCCACAACCGTCTAGAGGGCTATTTGGCAAGGCAAATTAGTACGTATATTTCTGGTTTCTGACTTCTATGGTGACTATATCTCAGTTTTCTGTATTTCTCACTTGTAATGGATATTGTTCACCACTTTGATAAACTCAACATTGGCTCTATGACTAAATCACTTAGATTTAGTTCAATGGAGGTTGGAATGAACTTGCCTCTTATTCCTTGTGTAGTAGCCCATATCTTTATTGCGATCTGTTGATATCGGATATGTTGATTTAATTAGTTATTGGTAGAGTCAGATGAAGAGGAATGTCCTGAAATTGATGAAGCTAGAAAAACCATTGCACGGATAGCCTTTTAAAGAAGTTTCAGCAGAAGCTTATTTGTTGATGGCCTTAAACGGTGTTCATGAAGATACCTTGATCTTAGATTTATACACATAACTCCAAACATTATTCCCTCTGACTCATTTGTTGTTGTAATGAATTTGTACCCAAGTTCATTGACCCAAGGTCGGCGTCTTTGTTTATTGTCTTGCTCACCATGCGCCAAATCATTTATGTTGGATTTATATGGAAATTGATTATGAAGTAGTTATCATGTTTAAATGTGATGTTTTTAGATTTTTAGCTCATGGGATTTATTGTCATGTATTAATTTTGGGCTTGACAAAATTAGGGTTAATATTAATTCATTGATTTGTAAAAGCG

>Ca3:21649858-21650285

TTATTGGTAGAGTCAGATGAAGAGGAATGTCCTGAAATTGATGAAGCTAGAAAAACCATTGCACGGATAGCCTTTTAAAGAAGTTTCAGCAGAAGCTTATTTGTTGATGGCCTTAAACGGTGTTCATGAAGATACCTTGATCTTAGATTTATACACATAACTCCAAACATTATTCCCTCTGACTCATTTGTTGTTGTAATGAATTTGTACCCAAGTTCATTGACCCAAGGTCGGCGTCTTTGTTTATTGTCTTGCTCACCATGCGCCAAATCATTTATGTTGGATTTATATGGAAATTGATTATGAAGTAGTTATCATGTTTAAATGTGATGTTTTTAGATTTTTAGCTCATGGGATTTATTGTCATGTATTAATTTTGGGCTTGACAAAATTAGGGTTAATATTAATTCATTGATTTGTAAAAGCG

>Ca3:21942476-21942939

GCTGAGGCATTCAATTTTTTGCGGGCTAATCCATCTTTTAAACCGAAGTCCATGGGCAAGAACTGTGGTTAGAGTCATCGACGACGACCCTTGCTTTTTACAGAGGGATATGTGACAATCAGTGTTGACGCTAAGCCAAGAATGAGAGAAAATGAAGCTTGAGAAGGTGTGATACATATTCTTGGTGAAGTTTTAGATTTTTTTTGAGTTAGAGTGTTTTAGACATGAAACAAAAAAGTATGTTTAGATGATAATCATTTAGGTACAAACATGTTGCTTTTCTGCGATATATGCATATTTTTACTGTGTTATTTGCTTCCTTGAAAAATTCTAGAAACAAATGCTAGAAACACTCTTCAACTAGATTATAGCTTATTTGGTAAATGAAATCTGTAAATATTATATTTAGATTAACTTATACGGTAAAATCCTCTTAAACTCAAACTAATCAATGGATTATCAG

>Ca3:22622169-22622528

AAACACTTTACCCATTCCTTGTCCTTTCTACAATCAAATTCTGAACCCTTCTCTTCTCTCTCTTCTTACCTTACAATTCAGAATTCAGTACTGAATTTGTTGAACGAACGAATGAATCTTTTGGTAACACAGAGAAACAGTGGCGGTTAAAATAACAACAACATTGCCACTTCCACGCTTTTTTTGTTTTTCTCCAAATCTTAATGCCAACAATGCAATTAAAGTAAACAACATTCTTTGAGATTTACGCAACATCATCATCATCATCATTATTATTATTCATTTCTGTCATGTTGCGGTCGTTAAAAGCTTCCCATAATTCTATTCTCAGATCGGACTCACGCTGACTGAATCAACAG

>Ca3:23397727-23398237

TCATTGCAAATAGTGAGGTGTGGTTGCCGTTTGGTATGTAAGCAAGATATTGTAGATTGGAACAAAGTTATGAGTCAATTCAATGAGAGTTGAAGATATTTGGAGATCATATAGTTAGTACTTGACAATAACATCATTCCTTGATGAACATGAAGCTGCCAGTAGTATGATGTCACCAATTTGACCAGCAAGATGCTGCACAAGCAAACAACAAGAAGCTGTATCAGATGCAGAATGAATCTTAGATATTAGAAAGTTTGAAACTTGATTCAGTGGCTATTGTTTAAAAAAGCTGGTTAGATTCAGTTCAAGATAACCAGCAACTTAGTTTGGTTTAAATTTGAACTTGATTTATTAGACCGTTTGAATGATTTATAAGTTCTGTCTCACGTAATATTCCATCATATTTAGGTAAATTTGAAATGAACCCTTTTTCTTGTCTTTGTTGTCTTTAATGACAGATTTTATTTTCTAAATTCTATACACTTTTCTTTGATTAGACTACTTGAT

>Ca3:23538276-23538501

AGATTGGAGGAAGAACAACTAGGACAATGTGAGAAAAAGAAGAATTTGAAGCAAAATGGTTTGAATTAGTTAAAATTTTAGAATAAAAATTGTCCATGTGGTTTGGGACATTATTATTTCTTTGTTTAATGTACTTTATCATAAACCTCGTTTTTAAGAAAAATTTTGTTGACTTGGATGTGAAAATACCTTATTGAAAGGAAGTTTGATCTTGTTTTACTCTTT

>Ca3:23548630-23548842

AGATTGAAGGAAGAACAACTAGGACAGTGTCAGAAAAAGAAGAATTTGAAGCAACATGGTTTGAATTAGTTAAAATTTTAGAATAAAAAGTGTCTATGTGGTTTGGTATATTATTATTTCTTTTTTTAATGTACTTTATCATAAACCTCATTTTTTTAAAAAACTTTGTGGACTTGGATGTGATAATACCTTATTGAATGGAAGTTTGATCC

>Ca3:23556581-23556888

ATATGGTGCACACTATATTTACTGATAGAGCATATCGAGTTATAACTAATGATACAGCTGATGAGGGTACTTCTAATGATGAGGGTCGTCGTGTTAGATCAACTGCTTCTACACGGACTTAGCGTATTGAGGAGCAGGAGGAAATGTAACGGGAGAGGCGGGTGAGGCGACAACAACAACAATTACAACAAGAGGCTCACCATGAAGAGGAGACCCAACAGGAAGAGCAAACACAACATGAGGCCCAACTTGAGCACATAGATGAACTAGTCCTAGCTGATGGATATCTTGGTGGTCCGGTTGACAA

>Ca3:23723221-23724457

CATAATAACCTTAGGCTTCAAGTTGTCATTTCAAGGAGCGAAGAAGAGATATATCTGTAGTCGCTGATCGTTGAATCGCCGATACGAATTCGATTCCTCACGAGTGCGAGTGCATATTTTCATCTGAATTCAAGATTCCACGAATCTGTATCTCTTTTTCTTAATTCAAGTTTCGCATTCAGGTTTCAATCTTTCCGATTTCTTTTTGAATTCTTTATAATTGTTTCGACTGGTGGCATCTTCGTATTTGACGTCAATGTTTTTAGGCAACTTTTTTCTGTTTCCTTTGCGACGGTGACATCCCGTTCTTCATGTTCAGTTCCATGAATCTTGATTATTCGAATGCTAGAAATCTGATCCATGAATCTTGATTATTCAAACCCTAGAAATCTTATTTAAATTCAAGTTTCTGCTGTCCATGGCTTGCCTCGATTCCAAAGAGAGATATAGAAACGATCGAAAGAAATATAAAGCTTCTAGCTCTTCTGAATTTGATATCATTATTCACTGGTTCATCTCTCTTTCTTTCATTGTCAATCCACTCTCCTTTAGATTTCTGATGTATCCATTCTTTTCTGATGCGTAATCTTGATGATTCTTGATTATTCATGTCTAAGAGTTGATAAAAATTGAAAAGTTTCCATGTGTATACTTAAGACAAGTTAGTTGCTCTGATTAGATGAAACAAAATTTAATGAATTATTGACTCTAAATGTGACTTAGAATGAGTTTTTATTCTTATGAAATTGACTTAGTATGCTTAGATGGATTCTTGACCCAAAAATGTGACTTGCAATTTAAATTTTGTATTCATATGAAATTGAGATTTATGTTTGGGTGACCCTAAATTTAATCAAGAGATAGATGCTTGACCCAAAATGTGACGTGCAATATAAATGTATGTGACTTGCAATATAAATGTTTTATTCATATGAAATTGAGAATGTATGCTTGAATGACCCTAAATTTAATCGCGAGATGGATTCTAGACCCAAAAATGTTGCTTGCAATATGAATCTTGTATTCATATGAAATTGAGATGTATGCTTGGATGACCCTAAATCACGAGATAGATACTTGACCCAAAATATAGCTTGCAATATGAATCTTGTATTCATATGAAATTGTGATGTATGCTTGGATGACTCTAAATCACGAGATAGATACTTGACCCAAAATGTGACTTAATATGAATCTTGTATTCATATGAAATTGGGATGTATGCTTGGATGACCC

>Ca3:24088179-24088414

ACCAACCAAATCCAACATCCCTTACTATAGCACAGATTTTGCCAAAACCATAGGCCATTGTTGCTGACCACCAAAATTGTCATCCTTTGTCCCCTGACAGAGGCGATTGATTCCAATCCAAGCAGATAATAGAAAAAGACTGTGACTGTACAGAAACAATATCACAAGACCAACCACAGGCACAGCTAGGCTGATTGATCTATTTGATCTATAACTCTGAAATGCAGCAACATGC

>Ca3:24238669-24238951

CCTAAAAGCTGCTGCTGTTTGATAAATACATGCTTTACGAGATTTCTCTAAATTCAAGTTGTGATACTGTATGATATATAAAATCCTTGTAAACAATTTTTCCAAGAAGGTGGGGTATATATATATAAACCACTTTGAGTGGGGAAACTGTAATTCTTTGACAGGAAACAATGTCAGTCAGTGTCATCAATAATTAATTACTTTGGACACACTAAAGTGCTTTTAGAGACAATATTTGTTTATACAATTTGATGAAGATATAAAAAATGGTGAACCGAGCTG

>Ca3:25030817-25031291

CAAGGTGTGTGTAGTTTTTGTCTGTTTCACCTTATCCTATATACACCTCTCTGTCACTCTCCCACACCTGCACGCGCCACACTATACAGCACAAAAAGAGGAAGAAGAAGAAGAAGAAAAAGTGAAATAGCTGTGGTGGGAGCATTTCTTTGTACTATATCACGATGCTCATGATTTGTTAACGTTTCTTTGTATTTTTAGCAAGTGGGTTGTTTTTCTTTTGCTCTTTGGTTCTGCTGTCAGTCGTCGTTTTGCAGTATCAAAGGTTGCTTTCTGCTTCACTGCTGTCGTGTGAGTAGCACAGAGGTTTTTGCAGGGTTTGTTTTTGGATTACATTATTGTATCTTCTTTCCTTAAATAATGAGTTTTTGGGATGCTCTTGTTTCACAATGGCTGCCATTTTTGGAAGAAAAAGATTGTTTTTTTGGGGTCTTGGTAAGGGAATGTTAAATTGCAAAGTTTAAGAGAGTAAAG

>Ca3:25084985-25085393

GAGAATAATAATGAATAAATTTGGGTTATTTTTTGTTTGTTTTATTTGTGGGGGGAGATGGTTAGATCTGAGAAGGATGGGATGGCGTTGGAATTAGAAAGTGAGAGGAGAATGATTGATTGGGTTGAGTAAGGAAATGGCATTACATTATCGAGGGAATATTAAAACATGTCTTTTTCTCTAGCCCTTATTCCTAATCCTAACTCTTACGCCTGTTTGGAGCACTAGAAACTAAGTGGAAAGAAAGTAAGGGAGAGGAAGCACGTTTTTCAAATACCTGTCCACCGTTTGAAAAAGCAGAAAATCGGAAAGGATTGAAGCCTTGCTGCTAGGTTCCAAAAAGGATCGGTTCCTCTCAATTTTGAGCGCAGAAGTGCTAGTACTAATAAATTATAATTTTATCCTCCT

>Ca3:25173499-25173826

TTTTTGGATGTTATTTTGTCCATCCTAATGTTCACAACAGTTTTTATCTTCTGTATTTTAAGAGTTTCATCATTCTTGATAGCTAGGTTGAAAGTGATTTGGTTAGACCGGACTTTCTAATGCTCAAGCCTGGCATGCATTTTATGAGTTTATAAGGACTTGGATACCCTGTGGTCGGAGAAAAAGAACACATTCACTCGGTCAAGGAATTTCAGTTGTTGGGTGCAAATCAGACAAATTGTGTTCTGACTTTACCAAATTGGCTTTAGAATACGTCTGGGGTTGCTACTGATGCTTTGCAAGAATGTAAATATTTGAAGAAAGAAG

>Ca3:25567673-25567888

CGCACTAAAACCATATTTCTACCGTGTTACGGGATAAATAGCTCCATAAACCCTTAAAACTCACTACTATTGTCTCTTACCATTCCATCTACCCTTTCGCCCAAAACTCCAAAACCAGACTTTGCCGTCACCCAAGACCACCTACCATCCAACTTCCGGCAGCGTTTTTCTGCAACCGGCACCGTTCTTTCTGCTTTATCAATCGTACATTCCTG

>Ca3:25870537-25870737

ATTGAGTAGTTCGAAACTTTCTCCTTCATCCTCGCAAACCCTAGTGCCTCCATTTTTCGTTCCCGCCGTTCTCCTTCATCCTCCATTCACTTTCTCCTTCATCTCCATCATCTTTAAGGCCTCAATAAACACCATCTCCCATTCCATTAGCATCTCCATCTTCGCAACCTTCAATGTCCCCAGTAAACGCCAGTTACGTG

>Ca3:25872103-25872497

GTGAGTGTACAAGTCAGGGATAATGAAGATCATAGTGTTATTGTGAGTATTAAGAACCTGCTCAAACACTTCAAGAGATGAAGTTCCAGTTTTCCACTGATTTTATCTGTTTACTTTGCTGCTGACCTAGTGCTACCACTGCATTACACAACATATAGAGATAGTTTTCCACTGATTATAATTATTTGGATTTTTATTATTGAATAAAAGAAATATTGTATTTGGCATTTTTTTTTGTATTTGTAATTGCTATGTTTGTTTATTTTATTATTTTGAATGGTTTTTATATTATGGGATGTACTTTTATTGGTGTGAAGGCAAACTAGTAATGTGTACAAGATTGTGAAGGCAAACTAGAATAAGATTGTAGTTTTATTTGTAATATTGAGTACTT

>Ca3:26225020-26225882

TGTGTTGCTCTTCTCGTATTCTTGTTGGACTTGGACAATTCATCTGCTGGAATATTTGTTTTGCATTTGTGTATTTTGGCTTATCTGGTTGGTTTGGAATTGAAGCTTTTGAATTTGTGGCACCGAAATTTTGGAAACGGCTAGATTCTTTCTTGTATCTCACTAATCTTGTCATAAGAGTTAAGTTTTGATTGTGTTTTTGTGGGACTTGGCAAAATGCGTTCAAAACACAGTTAAAGGTGAACTGCTTGATGAAGACCAGAGGGTTCAGATTCACATTAGCGTTAATGTCTGACTGCACGCATCTATTTTTGCTTTGGTGGGAGGAAAAGATATGATGTGTGATGTGTTGCTGGAGATAAAAATGTACAATCCAGTAGTGCTTGAAAAGCTCTAGCTAAAGAGTCTAGGTTGAGCCAGCTTGGTTTATGACTGCTTCATGGAGAGCAAATATTGAAAAACAACAAATCTTATTTGGAGGAGGGTAAATGAACATAATCAAAGTTCATGGTTTCGTTGTTCTTGTTCTTCAACTACCCTTTTAACTCAGGCTATTTTTCATGGATTTTCAAGTTAAATTGAAGACATGAAAGTAGTACATGTTTGGTTTTGCATTTGAAACATTGCATATTATGGTTTCGGCAACAGTTTGACTTTTTTAGTTGTAACTTTTGTCCTAGCCATGGTTTGAAATTTGAAACGGCAAACCAAATACATGATCCAGTAGATGCACAAGGTTGATAAACCATTTTTTTTTTTGTATTCCTGACTGATAAGCTAGCATCGCTATTTCAGAGACGTGAATAATTCTGCTTCTGTTCCAGCTGAGACCATCTGTTCAATTTCTCATTTGTGATCCAAG

>Ca3:26225253-26225882

TTAAAGGTGAACTGCTTGATGAAGACCAGAGGGTTCAGATTCACATTAGCGTTAATGTCTGACTGCACGCATCTATTTTTGCTTTGGTGGGAGGAAAAGATATGATGTGTGATGTGTTGCTGGAGATAAAAATGTACAATCCAGTAGTGCTTGAAAAGCTCTAGCTAAAGAGTCTAGGTTGAGCCAGCTTGGTTTATGACTGCTTCATGGAGAGCAAATATTGAAAAACAACAAATCTTATTTGGAGGAGGGTAAATGAACATAATCAAAGTTCATGGTTTCGTTGTTCTTGTTCTTCAACTACCCTTTTAACTCAGGCTATTTTTCATGGATTTTCAAGTTAAATTGAAGACATGAAAGTAGTACATGTTTGGTTTTGCATTTGAAACATTGCATATTATGGTTTCGGCAACAGTTTGACTTTTTTAGTTGTAACTTTTGTCCTAGCCATGGTTTGAAATTTGAAACGGCAAACCAAATACATGATCCAGTAGATGCACAAGGTTGATAAACCATTTTTTTTTTTGTATTCCTGACTGATAAGCTAGCATCGCTATTTCAGAGACGTGAATAATTCTGCTTCTGTTCCAGCTGAGACCATCTGTTCAATTTCTCATTTGTGATCCAAG

>Ca3:26398447-26399232

CCTAAAATCAACTCTCTTTTTTTAACTAACTAAACCAAACTCTTTTAGCATAAATCAATTATTAGTTTGAAATTAAAGTAGTTTAGTGTGTCCTACTCTGTTGGGACTTGGGAGAAGAGTAGGGATACTCAGGTCCTTTCCCAGACAACAGAAACTAGCGCTAGTATATGTTACGAAATGCAACAGCAAGAAAAGCAGGAAAGTGTGCGAGACCACATCTCTCACGCTTGCAGTTTTATTTTTTCCTGAAAAAAAAAAAAACTAAAAACATACCAAAAACACCCTCTTGAATTAGAAGATAATATCTCCAGCTGTAGTGGAAAAGTAAAATATCTTCATCACACTGTTCTGCACACAGTGGCTACCGTCACATACCAAATCATCTCTCTTAGTAAGTTACCACAAATTTTTCCTTGAAACCCCTAAATATTTTTCATCTCCTTTTAAAATTTTACCCTTCCCCCAAATCAGAAACCACAGAACAGAAAGTTAGAAACAAAGTGAAGAATGAAGATGCAAACAGTTAGTTGAAAAGAAGAGTTCCTACTCAAATCAGAAACCCTAACTAACCAACGGTTCACTGATATTGATGACGGTGGCGGACATGGCGGATGCCACGCGGTGGTGGTGATGACCTCCGTCCTGTGACGGTGGTTTTGTTGATGGTGGTGATGCAAATCTTATTATGACGGTGGTTTTGTTGATGGATCGATTTGTTGTAGTTCCGGAACGCTTTCCACCTTACGATATTCTCGATCTACAGTTCTACACATGCTGATTTCCAT

>Ca3:26400370-26400616

TGACCCATGCTTTTCCTAAGACAGGTTTGTGTGAAGGAGTGTGAGAACCAGCAAGATGCAAGTGGTAGCAGGAGTCCTCTAGTTGTTGAATGTTGAGTGTGTATTTAAACCTCTCCAAGTTTCCAATCAATCAAAGAGTGATTAAAGACGGAAACAACCTCAGGAACTTGTACATCCTGATAATTTACATTAAATGGTTGCCATATGCCTGTATTTAGTTTATTAATAACGTATTGTGGTCTTTAA

>Ca3:26398447-26399237

CCTAAAATCAACTCTCTTTTTTTAACTAACTAAACCAAACTCTTTTAGCATAAATCAATTATTAGTTTGAAATTAAAGTAGTTTAGTGTGTCCTACTCTGTTGGGACTTGGGAGAAGAGTAGGGATACTCAGGTCCTTTCCCAGACAACAGAAACTAGCGCTAGTATATGTTACGAAATGCAACAGCAAGAAAAGCAGGAAAGTGTGCGAGACCACATCTCTCACGCTTGCAGTTTTATTTTTTCCTGAAAAAAAAAAAAACTAAAAACATACCAAAAACACCCTCTTGAATTAGAAGATAATATCTCCAGCTGTAGTGGAAAAGTAAAATATCTTCATCACACTGTTCTGCACACAGTGGCTACCGTCACATACCAAATCATCTCTCTTAGTAAGTTACCACAAATTTTTCCTTGAAACCCCTAAATATTTTTCATCTCCTTTTAAAATTTTACCCTTCCCCCAAATCAGAAACCACAGAACAGAAAGTTAGAAACAAAGTGAAGAATGAAGATGCAAACAGTTAGTTGAAAAGAAGAGTTCCTACTCAAATCAGAAACCCTAACTAACCAACGGTTCACTGATATTGATGACGGTGGCGGACATGGCGGATGCCACGCGGTGGTGGTGATGACCTCCGTCCTGTGACGGTGGTTTTGTTGATGGTGGTGATGCAAATCTTATTATGACGGTGGTTTTGTTGATGGATCGATTTGTTGTAGTTCCGGAACGCTTTCCACCTTACGATATTCTCGATCTACAGTTCTACACATGCTGATTTCCATGTTGA

>Ca3:26400394-26400616

GTTTGTGTGAAGGAGTGTGAGAACCAGCAAGATGCAAGTGGTAGCAGGAGTCCTCTAGTTGTTGAATGTTGAGTGTGTATTTAAACCTCTCCAAGTTTCCAATCAATCAAAGAGTGATTAAAGACGGAAACAACCTCAGGAACTTGTACATCCTGATAATTTACATTAAATGGTTGCCATATGCCTGTATTTAGTTTATTAATAACGTATTGTGGTCTTTAA

>Ca3:26398482-26399230

CAAACTCTTTTAGCATAAATCAATTATTAGTTTGAAATTAAAGTAGTTTAGTGTGTCCTACTCTGTTGGGACTTGGGAGAAGAGTAGGGATACTCAGGTCCTTTCCCAGACAACAGAAACTAGCGCTAGTATATGTTACGAAATGCAACAGCAAGAAAAGCAGGAAAGTGTGCGAGACCACATCTCTCACGCTTGCAGTTTTATTTTTTCCTGAAAAAAAAAAAAACTAAAAACATACCAAAAACACCCTCTTGAATTAGAAGATAATATCTCCAGCTGTAGTGGAAAAGTAAAATATCTTCATCACACTGTTCTGCACACAGTGGCTACCGTCACATACCAAATCATCTCTCTTAGTAAGTTACCACAAATTTTTCCTTGAAACCCCTAAATATTTTTCATCTCCTTTTAAAATTTTACCCTTCCCCCAAATCAGAAACCACAGAACAGAAAGTTAGAAACAAAGTGAAGAATGAAGATGCAAACAGTTAGTTGAAAAGAAGAGTTCCTACTCAAATCAGAAACCCTAACTAACCAACGGTTCACTGATATTGATGACGGTGGCGGACATGGCGGATGCCACGCGGTGGTGGTGATGACCTCCGTCCTGTGACGGTGGTTTTGTTGATGGTGGTGATGCAAATCTTATTATGACGGTGGTTTTGTTGATGGATCGATTTGTTGTAGTTCCGGAACGCTTTCCACCTTACGATATTCTCGATCTACAGTTCTACACATGCTGATTTCC

>Ca3:26398502-26399238

CAATTATTAGTTTGAAATTAAAGTAGTTTAGTGTGTCCTACTCTGTTGGGACTTGGGAGAAGAGTAGGGATACTCAGGTCCTTTCCCAGACAACAGAAACTAGCGCTAGTATATGTTACGAAATGCAACAGCAAGAAAAGCAGGAAAGTGTGCGAGACCACATCTCTCACGCTTGCAGTTTTATTTTTTCCTGAAAAAAAAAAAAACTAAAAACATACCAAAAACACCCTCTTGAATTAGAAGATAATATCTCCAGCTGTAGTGGAAAAGTAAAATATCTTCATCACACTGTTCTGCACACAGTGGCTACCGTCACATACCAAATCATCTCTCTTAGTAAGTTACCACAAATTTTTCCTTGAAACCCCTAAATATTTTTCATCTCCTTTTAAAATTTTACCCTTCCCCCAAATCAGAAACCACAGAACAGAAAGTTAGAAACAAAGTGAAGAATGAAGATGCAAACAGTTAGTTGAAAAGAAGAGTTCCTACTCAAATCAGAAACCCTAACTAACCAACGGTTCACTGATATTGATGACGGTGGCGGACATGGCGGATGCCACGCGGTGGTGGTGATGACCTCCGTCCTGTGACGGTGGTTTTGTTGATGGTGGTGATGCAAATCTTATTATGACGGTGGTTTTGTTGATGGATCGATTTGTTGTAGTTCCGGAACGCTTTCCACCTTACGATATTCTCGATCTACAGTTCTACACATGCTGATTTCCATGTTGAG

>Ca3:26773098-26773374

TAATTTTCGAAAAATGATATATACTCGCGCACGCAACACGGAGGAAGGAAAGAGGCACCGTCAATGTGGTTGGGTCGACACGACTTTTACCGTTGAAAATGAAAACTCTATTTGTTGCTTACGCGTAAATGCCACGTCACAAAATCTTACGGTTATGGATGATGAAAAAATCTTATAAATATTACAATTAACCGAAACTCATACTACTAAGCTACCAACACTTTTCTTCTACCGAAAGCACCATTCACTACACCTACTATTACTGTCATCGTTACG

>Ca3:26783995-26784957

AGCGAAAAAGATATACAAGCGTGACGAAACTGAAAAACAGATTTAGCTTCTTTATTCAAGTTCGATGAATCTGTTGTCGCCGAATCAATGAATCATCAATCCAAATTCGATTTCTCGCCGCTGCTACATGTTTTCATCTGAATTCGAGATTCGATGAATCTGTAGTCTCTTTTTCTGAATTCAAGATTCGCATTCAGGTTTCAATCTTTCTAATTTTTGAATTGAATTCTTTATAGTTGTTTCCACTCGTGGCATCTTCGTGTTTGACATCAATGTTTTGAGGCATCTTTTTTTCTGTTTCGTTTGCGACGGTGACATCCCGTTCTTGAACGTTCAACTCCATGAATCTTGAATTTTTAAACCCTAGAAATCTGATTTAAATTGGTGCCGAAAAAGACGACCAGTGAGTCGCAAGCTTGGTTATTCGGATTTAAGTTTATGCTATCCATGGAATTGGAATTTGCCTCGATTCGAAAGAGAGATGTAGGTATGAAGATTCTTGATTGGACCTTCAATTAATCTGCCGCCCTCTCAAGTTTTGCATTGCATCTCTTCTGAATTTGATATCTTTATTCACCTGTTCATCTCTCTCTCTCTTTCCATTTTCAATCCACTCTCCTTTGGATTATCGATCTATCAGTTTTTTTCTTTTGTTGGGCAAACATTATCCATCTCCAAAACCATGTTCGACATGAATGTTTTGAGGTAGTTTTTTTGTTTGCAGCATTAATGTAATATTATCAATTGTCAAGAATGTATCATCTTGATGATTCTTGATTATTCATATCTAAGAAAGTTGATAAAAAATGAATAGATTAAATATGCATCCTTAAGACAAGTCGCAATGATAGATAGTTTAAATTTTAATTATGAGATGGATTCTTGACTCTAAATTTCTGAGGCTTGCCATATGAATTTGATATAGTATGTTTGGATGATTCTAAATTTAATCAATAGATGGA

>Ca3:26962188-26962389

CCCGTTAGGAAGCGGGTCGGGTTTTCACAAAAAATTGTAGCATATCTTATGAAAACCACTGGAGAGTAAGAGAGTTAGGGTTTCTTGACTCTCCATTTCTGCTCGATCTTCATTTACGTAGCAACTTCCGACGAAGGCCAACCCGCACTCCTCTGCCCAACGTCAAAGTCCATCTGCAGAGTAGCACTCCGCCGCAGCACC

>Ca3:27077485-27077843

TTTGTTGTACAAGGACGTATAGGTGTTGACTGACAACAAATATTTTACTTCGCATTTTCTGGTAAAGATTATCCTAGCAAAAGTAAGTCATTGCTTGTTCTCTGGTTTGCAGTAGGATATTATATTTGTGAGTTAAGGTGCTTCTCTTCTCACAAGCCTAAGATGAATATTCCATTTCACTTTTTTGGTAACATATCATATTCATTAGTTTTTGTAGGCCATTATGAAAACTAGATGTTAACCCAGGTGTTGCACGGGAATATTTTTTTATTTAATTTAAGTGAATTTAAAAAAAAATTAATATAAAATTATATGTCTTAATTTAGTATTTATATCAATAATATATATATAATTTTAC

>Ca3:27313892-27314226

GTTTCTGTTTGAGGGAGAGAAAAAGGGGACTAACGCGACCTTGAATTTCGATGAATTTGTAGTCGTTGAATCTGAATCGTTGAATCGGTGGTCTCTTTTTCTGAATTCAAGTTTCGCATTCAGGTTTCAAGATTCTTTATAGTTGTTTGGCGTCAATGTTTGGAGGCAACTCTTCTCTGTTTGGTTTCCGACGGTGACATCCCATTCTTGAAATTCAACCAAGAACGTTCAACTCGGTAAATCTTGATTATTCAAACCCTAGATATCTAATTTGAATTGGTACCAAAAAGAGACGGATTCTGAATTGATGCTAGGGCTTCATACTGGTTTGCAA

>Ca3:27323681-27324047

GTTCTCTATTGCGGTACTAATGTCTTATCTAGTGTAAGCACAAAGAGTAGAATAGATAGTTTTTGAGAAAAATGCAAAGCAGTTCCAAGGGAGGGTGCATTGGAGATAGATGAGCTTAATGTCATGATTTGAAGTACTTTGTTGGAACTAATTGACGGTTGCTGCAGAATTTGGTTGGAGATTGTAGCAGGATTTTTTTAACAGACCTGCAAAATTGGTTTGGGTTAGAAAGGTTTGGAGCAAACTTGGGTGCTGTAGTATTGAATTTTTTGGGAGGTTCAATCACAAAAATTTTCCTTTAACTTTCTCAACCTTAATTTGTTTAATTTCTAATTATTTATAAAAAAAAATTTCCATGTGCAAGGC

>Ca3:27669810-27670015

CAGATGCGGAGACATAATCCACTTCTCTAGCATGATCGTGAGACAGAATCCACTCTTCTAGCATGATCATAGAAGTCCGTGTAAGCATGCACAGCAACCTCTTATTTCCACGTATATTTGATATTTGTTTTTGTTTTGAGAGTACTTTGTCACTCGAATTCTTATGTTATTCTACCAAGCCACTTTATGGTATACTCAAATTCCT

>Ca3:27669813-27670015

ATGCGGAGACATAATCCACTTCTCTAGCATGATCGTGAGACAGAATCCACTCTTCTAGCATGATCATAGAAGTCCGTGTAAGCATGCACAGCAACCTCTTATTTCCACGTATATTTGATATTTGTTTTTGTTTTGAGAGTACTTTGTCACTCGAATTCTTATGTTATTCTACCAAGCCACTTTATGGTATACTCAAATTCCT

>Ca3:28393126-28393342

TAAACACAGGTCTTTTAAAAAAGAAACACTTGCCCATTCAGAAACACCTATCGTCCTGGCTCATTCGGGAACATTGCGCTCCCTCTCTGAATTTCCCTCTAAGAAGTTTCTCGCTCTCGCTCATAAGAACACTCATAATAAATTCCTTGCTCTCTCAATCTCACTCACCCGCACTCAGAATCTCTATCTCGTTTATAAAAAATCTGGTTCTAATAG

>Ca3:28394353-28396100

TTGAATTCCTAAAAAATAGCATCCAGACAAAATATTGGAAATACATTTTATAATATCAAAGGTGAAGAACCTTTTGGACAACTTGTGACGTTGAAGCTTAATGAGGAGGCTTTGGGACAACTAAAAGGTGCACTATTCCAAATTACTATGAATCTAATTTTAGTAACCTGATATCCTGTAACAGATCATTTTTCCATCAAGACTTGAGTTAGGATATTGTATTTGTCTTTCACAGTTTACTCTCTAGTTGATTTGTTGGTGGTGCAGGTTCTAGGCTCCAAGTTTCAAACTGGTAGCATTTTTCTAAAGTTACAACATTGTTTTGTATATTATATACACTGTAACCTGATTCAGAGGTTGACAATTTGATACTTGCTTAGATTATAATTTTGAAATTCCATTTCTGACACTATATACATGGCAACCTGATGCAGAGGTCCACTATTTGCCGTCATATCTAAAACTCGTAAGGCATATTTGGAGCTTTAATTTATCATATAAGAACAGTAATATTCACTATTATGAGTTGAAAATCTGTGTAGGGACCTCTCTTCTAACAATCGTAGTGGTGGCTTCTTACCATGAATATCAACAAGAACAATCAAATAAGATCCCTTCACTTTTCTTTTATTGTTAATTTTCTTGTAGTCTTTTAGTTGTATATAAAAGTTTACAACATAGTGGTAGAAAGTTGCCAAGGTAGTAGTCAAGACATACAAATACAAGAAATAGGAGCTATTATTTATTTGTGTTATTTAATAGGAAGTTGTAATTAGTACATGTGATAGGTGCTAAGTATGATCCATTTGATACATTAAATGGCTTTAACAATTTTAAATTTATGATCAATTGGCGTGACTAATAGAATTATGTTTGTGAAGCTCAAGTTAGTAGTTACACTTTTTTTCCTACTGACATATATGATGGATCACTTGATAATTTCATATATTTATTGCATGTTTTTTCATCTAAAATAGGCAGCCATATTTTTTTGACTTGTGCACTTACCTAATGCTCGTATAGCGATTCACCTAGTTGTTTCATATCAGGCACATACAATAAATGTACCATCATAAAATTTTATAATGAACTGGTGCACGCATCAATTTTTTTTCTTCCTTTAGGAACAATTCTTACCACCGGAAAATGTATTGAACAAGAACAGGAATCCACATTAAAGTTAGAGCCAAATATAGGGTTGAGCTTAAGGCATATCTTTGAGTCCTAAAGGTTAAGTGCAGTATGATTTACTAACATAATACACAGTATCACTTTCTTAGTTTCATGATCCATATTGACGATTGATGATATTATGTCTATGTGATAAACGTTTGTGTATAGCTTCTAGTCTAGATGCTACCACTTTGGCCTTATAAGTGATTTATGATTAGTTGACTTGCTACTAAATAAACTTCTTTGGAATGTTGCTTTTCTTACAGGATGTAGGACTGTCTATTCTTGAAAGTTATTCGAAGGTAATGGAGAGACTGATATCTTCTTAGTGGCTCATTTCTTTATATTGTTAGATGTATTGGAGCCTATTTTTTTTTTTAGAGTTTTAGAGATACTTGTTTTGAACAAGTCTTTGAAGATTGAGACAATGACGTATATGGAATGAAATTCTAACAAAGTTTATATTACTTTAAGGTGTACTAGTTTTAGTTATATGTTTAATTTGCACATTTTAGATTTGAGGCGGCTTTCCTATGTGATTATGTTATTTTAACGGAGTATACGTCAGTTGAAG

>Ca3:28395792-28396100

GATGTAGGACTGTCTATTCTTGAAAGTTATTCGAAGGTAATGGAGAGACTGATATCTTCTTAGTGGCTCATTTCTTTATATTGTTAGATGTATTGGAGCCTATTTTTTTTTTTAGAGTTTTAGAGATACTTGTTTTGAACAAGTCTTTGAAGATTGAGACAATGACGTATATGGAATGAAATTCTAACAAAGTTTATATTACTTTAAGGTGTACTAGTTTTAGTTATATGTTTAATTTGCACATTTTAGATTTGAGGCGGCTTTCCTATGTGATTATGTTATTTTAACGGAGTATACGTCAGTTGAAG

>Ca3:28394620-28396100

GTTCTAGGCTCCAAGTTTCAAACTGGTAGCATTTTTCTAAAGTTACAACATTGTTTTGTATATTATATACACTGTAACCTGATTCAGAGGTTGACAATTTGATACTTGCTTAGATTATAATTTTGAAATTCCATTTCTGACACTATATACATGGCAACCTGATGCAGAGGTCCACTATTTGCCGTCATATCTAAAACTCGTAAGGCATATTTGGAGCTTTAATTTATCATATAAGAACAGTAATATTCACTATTATGAGTTGAAAATCTGTGTAGGGACCTCTCTTCTAACAATCGTAGTGGTGGCTTCTTACCATGAATATCAACAAGAACAATCAAATAAGATCCCTTCACTTTTCTTTTATTGTTAATTTTCTTGTAGTCTTTTAGTTGTATATAAAAGTTTACAACATAGTGGTAGAAAGTTGCCAAGGTAGTAGTCAAGACATACAAATACAAGAAATAGGAGCTATTATTTATTTGTGTTATTTAATAGGAAGTTGTAATTAGTACATGTGATAGGTGCTAAGTATGATCCATTTGATACATTAAATGGCTTTAACAATTTTAAATTTATGATCAATTGGCGTGACTAATAGAATTATGTTTGTGAAGCTCAAGTTAGTAGTTACACTTTTTTTCCTACTGACATATATGATGGATCACTTGATAATTTCATATATTTATTGCATGTTTTTTCATCTAAAATAGGCAGCCATATTTTTTTGACTTGTGCACTTACCTAATGCTCGTATAGCGATTCACCTAGTTGTTTCATATCAGGCACATACAATAAATGTACCATCATAAAATTTTATAATGAACTGGTGCACGCATCAATTTTTTTTCTTCCTTTAGGAACAATTCTTACCACCGGAAAATGTATTGAACAAGAACAGGAATCCACATTAAAGTTAGAGCCAAATATAGGGTTGAGCTTAAGGCATATCTTTGAGTCCTAAAGGTTAAGTGCAGTATGATTTACTAACATAATACACAGTATCACTTTCTTAGTTTCATGATCCATATTGACGATTGATGATATTATGTCTATGTGATAAACGTTTGTGTATAGCTTCTAGTCTAGATGCTACCACTTTGGCCTTATAAGTGATTTATGATTAGTTGACTTGCTACTAAATAAACTTCTTTGGAATGTTGCTTTTCTTACAGGATGTAGGACTGTCTATTCTTGAAAGTTATTCGAAGGTAATGGAGAGACTGATATCTTCTTAGTGGCTCATTTCTTTATATTGTTAGATGTATTGGAGCCTATTTTTTTTTTTAGAGTTTTAGAGATACTTGTTTTGAACAAGTCTTTGAAGATTGAGACAATGACGTATATGGAATGAAATTCTAACAAAGTTTATATTACTTTAAGGTGTACTAGTTTTAGTTATATGTTTAATTTGCACATTTTAGATTTGAGGCGGCTTTCCTATGTGATTATGTTATTTTAACGGAGTATACGTCAGTTGAAG

>Ca3:28394353-28394645

TTGAATTCCTAAAAAATAGCATCCAGACAAAATATTGGAAATACATTTTATAATATCAAAGGTGAAGAACCTTTTGGACAACTTGTGACGTTGAAGCTTAATGAGGAGGCTTTGGGACAACTAAAAGGTGCACTATTCCAAATTACTATGAATCTAATTTTAGTAACCTGATATCCTGTAACAGATCATTTTTCCATCAAGACTTGAGTTAGGATATTGTATTTGTCTTTCACAGTTTACTCTCTAGTTGATTTGTTGGTGGTGCAGGTTCTAGGCTCCAAGTTTCAAACTG

>Ca3:28712114-28712356

GGGTATTCTATGATTATGCAAAACATTGCAAAATCTACTTTACCCCATATTTATTTTTATATGGGGTCAATCCATGTTGTGTGTATATATATATATATTGAGAGCTAGAAGATTAACATGACAGTATAATTCAGCACATACACATACCTTGATACAAGTGCAATCAGCCACCTTTACACAACTCACTTTTTCATTCTCCAAAATGCAGAAGCCAGAGACCCAGAGAAACCAGCCACCACCAG

>Ca3:28806130-28806639

AAAACAAGATACGAGGGCTTCAATTGGTATATTCTGCAAATTCTTGACCAATCAGATGATGAGATGAATATTATGGTATGGTAGCTGCATTGAGTTAGACCATAATAAGCAATCATAAAAATGGTTTTCTGAATATTATGTTGAGCTTTTAGTGTAAAAGGGGAAAAAAGTACTGAAACTTGTAGGATGTTATTATGTATTATGTGTGTAAAAACATTGCATTCTGATTCTATTTTGATTTAGCAGATGATGCTTAGAAAATTCAAGGTAATGTAACTGTCCTTCTACATATTTTATTGCAACAGCAACACTTTTCATAATCACTAGTGTGTAGCTACAATTATTGTTAACTGGATATCAGTTTGATACCAGCTGGACTCAATAGCTGTCATTGAGCTGGAACATAGTTTTGTGTAGAATTCTATTATTATCCAATAATAAAACGCATGTTGAAAACAAAGTACTCTAGCATTTCTCTGTTTTGCTGATTTTGTGAGAATGAAATTTTG

>Ca3:28887232-28888000

GGGTATAATACAAGTAGTTTTATTTTGATGCAACGTACATTTGAGTTGGTTGAACCAAGACATTTTTTTTTATCTTAGACAATGACTCCTTTGGCATTTACAATGATAAAGACTATGTCCTCAAAATATCCTTGTCCCTTTCTAAAAGATTAACAATAAAATCCCCCCTCATCCCTCTTCACAATTCCTTCATCTTTTCAATTTCCACAAAACTCTCTCACAAATAATAAATAACACACATTTATAGCCACAATCATTCACCATACCCACTATATTGATCTCAATGTTCTTCATTCCATTGTCAATGTGGTTTTTTCATGGTGTGGTTCAACATTGAAGATGATGATGGTAAAAGAGAGCACTAGTACTAGTAGTGGATGCATGATTAGGTACTCTTTCTTGATTTTGGTTCTTCTGGCTTTGTCAATTCTACTTTTACCTTTGGTGATGCCTCCTCTACCACCACCACCTTTGATGTTTCTCTTTGTTCCTGTTTTTATATTGTTGCTTTTATTTTTCTTGGCATTTTCACCTTCTACCGTTCCAAATATGGCTTCTTTTGTATCTTGATCACTTGTAACAATTAACTAATTAAGCATATAGCTAGCATTTTCAAATCTATACTGTTATGCACTTGTAATTAAGTTTGAGGAAGATTAATTTGTGTGGAATATGATTTTCTTAAGTATCATCTTGAAGAACTTAATTATGAGGTAGTATTCAATTTTGTACTTGTTAAGGTTTTCCTAGTCAATAATTATATACCCT

>Ca3:28986375-28986645

CATTAACACGCTCATATTTAAAAAGACGTTTAATTAAGAAGGTCAACAAATATATACGCGAAGAGTTGACATTGACCTTAGAAGCCACCACCACGTTGCGTCCGAACCAAGACGCACACTGGACGGTGTTTTCACCTAACCTACAACAGTGGGACCCATTTCTTTCCCGAACCGACTTAGGTTCGGACTTCCCTTTCCCCTTCTATAAATTCACGTTCGCGATTTGCCTCGACGCGTAATTAGTAACGACTCGTTAACCAATTTTCTCAG

>Ca3:28987997-28988222

AGGAAACTTTTGTGGAGCCATAAGTCAAAGATGGAGCTTCCAACAATTCTATCTACCAAAGATGCCTTAGCTTTCGGTCATTTAGCTAATGTACCATGTCAGCTTTAACTTTCTGTAACTTCTGTATAGGCAAGGTTTCAACTTTCAATCAATCCTTCTTACCAAAATTTATGGTAAAATTTAGACAGGGAAAAGAAAAAATGTAAAAATATTTCATGTACTCAT

>Ca3:29022853-29023286

ATTGATCATTTTATGGTATATGCGAATTAATTTGAATGTATCAATGGGAAATTTATGATCTGAAATTGAGGTTCCACTTCTTACTCCAAGCCTCCATTGTCTGTATACAATGTTGCCTTCTCAAACCGGAACATGTAAATATGCCTGAAAATTTATAGAGGCTCCAATTTAAGGAGGATAAGTGAATGATATAAAGTGACAACAATTAAATTCCATAACTGTGCTTGAGGGAAAATGTCATTCTACGAGGTTGTTCCAATAGCTTTATCAAGCTTTTGTTCAACAGTTTAAAGCCTAGACTTATGATCTCACACATGCTATGTGAAATTATATAACAAGTATCTTTGAAGTTTTTGGAAGTTTTGGGAGTTTTTGGAGCACTTGTTAAGAAATTAAAAAATTAAAAGTGAAAATATAATTTTGAAAGTGGTGC

>Ca3:29175058-29175354

CTAAACTTTTTGGTGTTCCCTTTCTCTCTTTCTCGCGACAGCACCGCTACCTGCACGAGGTAGAAAAATCTTCTTCTCTCATTTATCTCCACGCGTATTTCTCTCCAAGCTTCCACTCCATCTCCATTTACTCTACTCTTTCATCAATACACGAAAAAGACAACCAAACCCTAAATTCATCTCCTTCTCTCCCCTTAGTAATAACGCCGTCGTAAGAAGCTAACACCGCCGTCGTAAGGAGCTAACACCGCCGCGCCCAGCTCTCCATCTCTTTATCTCCCTTTCTCCTCTACTTG

>Ca3:29454128-29454667

GGGTTCTGTTGCTAAAATTACTGAAAAAGAGGGGTTCTGCTTCTCTGGTGAACCTTCTTGTGCTGTTTTCTTGGAGTTATAAAAAAAGTTAACTGTGCTCCCATCATAGTTTTCCATATCTATGTGGCAAGCCAATGTGGTCGCTTACACTCCTGAAAAGGTGACCATATCTTATATCTTATGTTTGCTCTCAATTGTTTTTACAGATATCCAATTGTATTGCTTCTCTTATTGATATTCTGGTTTGGATGATCTTTGGAGGCTATGGTGCTAATTATCATTTTGGTTTCTTTCTGATATATGTGGAGTTTGGGAGTTTAATTTGCTCGACTGGCAACTCATTCAGTGGCGACAAGTTTTTACATCAGTAATGAGTGGATTAAGCTTGGAAATCTCATTTGTTAAGTAGCAGCATGTGCTAATGTTTTGGTGCAAGATTGGTGATAGGTTTTTGTTTTTTTTCGTGCTAGCAATTTTGTGGTGCTAGTTTTTTTTAGCAGCAGCATGTGCTCTAGGTTTAGACGAGTGATGGAGTTTCG

>Ca3:29530813-29532339

GTCTATCTTAACACTGTATGAGAAATAGTGCTAGTTAAAATTCAAATATAGGGGAGTAAAGCAAGAAGATGCGGACAAGCACCTCGTAAAATAAACATAATGCAGAGAAAGGACTGAACTGCTTGAAGAAATCCTCAGCATGAATGAATTAATAATCATGACTTACAAAATCTAATATGAGGAAACTAGTAATCAAGAAAACTACGACATTAGGAAAATAGTACAAATCAATTATGAATTGTTCATAGAAAATATACAATGCTAAATTAGAGATATGAATTATTAGAGTTGGTGATTGTTGGTTAGAATTCTAACAGAATTTGATACACATAGACTATAAAGAGATATGAATAGAAGGTAAGGGGTATCATGGCAGCTCAAAATCGGACTTAGAGGGAGGTCTTACGCTCTCAACATACATCAAGGAACTTGTATTCGTTCTTTTTCTTCCCAACTAGGGCTCGTCACTAATTTGGGCTTAATGGAGATCTAAGTTATTTCTAAAACTCCGATCCAACATCTATCAATTGGTTCATATGTAAGGCCAAGAGATACCATGATAAAAAGTCAAGATTTTAGGGATAAACTGTACAGTTCTGTGGCCTTTTCTTATATAATCAGCAGGAGTCTAATCCTTTACAAATATAGGAGAGATTAAATAAGATTGTCTTAAAGTCTAACCTATAAGTCTATAACAAACAACTAACTAACTAACAGTTAACAAAACAACTAACTATAAGTCTATAACTGACCAAGACTAGCCCAGTCTGTTAGCAGACTCAGTTAAGCTGAGCTGGCAGTGCCACCTGTTTGACCTAACAAGCTGAACAGATTATGCTACGATTACAGCACTTCAAATGCATATTTTGGTACAAATAAACATATTATAACAGGATACTGTAATCATATTGTGGTACTAATACAAACATGCATCAAAAGATCAACATAAAACTTGGATATGATATTTAATTTTAAATTTAAATGTTTAGTAAATGAAACTAGAAGTTAAGGTCACTAAAATCAGCACTAGAAACTATTTACTACTTAATCTTGTCCAAACAACCGTAAATGCACTTTTCAGATGTAATGAAGAAAAAAGTAAAAAGTGTTATCCTCTTTCAACACAAGTCTCTTCTAGTTCCATGCTTCTCATGACCTCAAATTTTTCACAAATTTTAAGCATAGGAAGAAAAACAGCATCTATAAGAGTGGAAAGAATGACTGAACCTCTACAAGCTGCATATTCTACTGCATCAGTGTCACAAGTAACGATGCTCTCTTGAAATTTCCTGTATTAAATGAGGGATCAGTAACTGTTGTAACTTGTAAGATTGTGAAAGCTGATGCTATAAATGTACAATTTACAGATTACACTTTCCAGCTTGTGCATTGAGTATTAAAGAAGTGACTTGGTGCAAGATACATGAATGAAAGTTATTAGAGAAGATTGAGGATTTTCAAGAATACCTTGTGTATGGTAAAGTTTTCAATAGATATGCAATATAGTGCGAGGATTTTCACACATA

>Ca3:29566939-29567380

TCTCAGTGCGCTTCATCTTTTTCTCCTTTATTTTACTTGATTAATTAAATTATTTTTATTAATTAGTATTTTTTAATATTGTCCCGGCGATTCTATCTAATCTGAAAATAATAATTTAGAAACATTTATTATTTGATTATTAAGACATGAAAAATTATTTTGTGTGTCACACTCCATGTTTTGTTTTTTTAATTTAATTAATTAATTATTTTTGGAAAGAAAAAAAAAAAAAAAAAGAACGCATGGGAAATGGAGAAGGAAAGAAAGAAAGAAAGAAGCAGAAGCTGTTGTTCTCATTTCTCAAAAATTTGTCCTGAGATTTTGTTAGTATCCAAACACAGCTGAACTTATTTTCTCAACTCAGTCGTGAGATCTAAGGACACGTCACTCTCTTAGCTCGATCCTTTTTTACTTACTTGTTTTGCCACTGAATTATAAGTG

>Ca3:30286842-30287268

ACAAATCTCCATTGGAAGTTGGAGAAGAGTGGAAGAACAACGTTTTAATGCGTTTTATAGTAATGATTTTTTCATTTTTTGCTTTCTCGTGAGTAACGGTGTGTGGTTTTAATGGCGGAAGTGAACAATTATCACTATCAATATCAAATCAACTAACTCTAGCTGGCTTGGAAGAGAAGCATTCTTTTGTGAATAAATTTTATTGCTTTTGTGTGAATGGTTCTGCTTTTTCATGATTTTGCCTTATACCACAAGGCGAAACAGAAGAGTCACCGCCACAACCAAACTTCTACTTGTTTGCTCTCTTATGTTAGTTTAATTGCTCATTAATGTTTAATTTATCACTCTTATCAGCTTCGGAATCAAACGGTTCTCCGAACCAAAAACCTCCATTCCATACCACTAAGACTATGTAATACTTCCTCT

>Ca3:30647658-30648017

CCAACTGTAGTCTCTACCCTAATTCCCTTTCTAGTTCTGGAAGGAAGTTGCTTGACACAGAGCAACAGTATCTTTCTGTTCCCATTTGTTCAATAGTTTGATGGTCGAAAAATATAAGTGACGGTGTGGAAGTCCTCGGAGGGATGTAGGAACTCTATGTTACGGTTTTGGTTTTAGGGATGTGGGTCGCAAAATACGACCCGAAAATCATGTTTTTTGGAATCGCGTGAGAACGATTCTGTTGCAATCGTAGATCGTTGAGAGTGAGCATAGTTTCAATTTCCAGTAGCAACCATGCTCTCTCCCTCGCCATGACGCCTCAATTTCTTGTCAATCGCCTCAATTTCTCGTCCCACAAG

>Ca3:30650127-30650895

ATGTTGGACCAAAAATTGAAAGGAATGGAGGGAATGAGTTTTTGGTCACCTTGTTTATTGGATAGTCTCCTCATATATCTGAAACAAGCAATATGAGCACAAAATTACATTAAGCTATTGCCAAGAGTTAGATGTGAAGCATTTTTTTGTTGCTTTCCACACTTGCTTAAATCAGCTGCTTTGCTTCTAGGTTTCAATGAAAGCAATTTGTCTGCTGTCATGTTCCAGATGATTATCAAGAAGAGTGTCCCTTCACCGTCAGATTATATTTTTGTTTGCTCATTCGTCGGCTGGGTATTTTGTGCCAAGTTAACATGTGATGGGCAATATTTTTCGTCATGCAGAATGTGGTGATCTGGATGAGCCGATTTTGAATATTTACAAGCTATCCTTAACATGTATGGATGTTATTGAGGTATTATGATATTGTCAGTGCAATATGCAGCCGTATTCACCACACTTGGCCAGCTCTTTGGATCCCTTCCAGACGACTATGATGTTTTGAATATTGAGTTTGTTGTAAGATAACATCTTTTTGGCCCCGAGAAAGTGAAAAGCAGCGGCTTCTGATCGTGATGAAGATACATCGAGGTTGACTGATAATAATTCTTTTAATCTTTATAGTGCAATCTGTATAGCATTAATTATCATTTGTTTCAAAATATCCTAGGTGGAAGCTGAGAAACAAATCATTGATTCCTTGAGGACTTTTGTGTAACTGCATGGTTCACTATATTTAATGAATTATTTGCCTCAAGGCTGCTGGTA

>Ca3:30647667-30648017

GTCTCTACCCTAATTCCCTTTCTAGTTCTGGAAGGAAGTTGCTTGACACAGAGCAACAGTATCTTTCTGTTCCCATTTGTTCAATAGTTTGATGGTCGAAAAATATAAGTGACGGTGTGGAAGTCCTCGGAGGGATGTAGGAACTCTATGTTACGGTTTTGGTTTTAGGGATGTGGGTCGCAAAATACGACCCGAAAATCATGTTTTTTGGAATCGCGTGAGAACGATTCTGTTGCAATCGTAGATCGTTGAGAGTGAGCATAGTTTCAATTTCCAGTAGCAACCATGCTCTCTCCCTCGCCATGACGCCTCAATTTCTTGTCAATCGCCTCAATTTCTCGTCCCACAAG

>Ca3:30733483-30733820

GTAGGTGTAACTGCTCTGATGCTATTTTCTTCTACAGATGTCATCTTCTACACTGTTGGTTCGACTTCAAGATTGTCTTTGTTTGTGAAGTCCTTAGTAGCTATAAACTTTCTTGTGTTTCTTTCTTTGTGCTTTCTTAACTTGTGGAACTTGTTTGATCTGCTGAGATAATTGTTTAACAACTGTGATCTACATTTCATGTTTTATATCTTAATTAAGAACTAAGTACTTGTATTCTCTTCTCTGATTATAGCTATAGATTTGCTTTGAAAGCTATGAACACCATTTTGTGGTTTTAATCTAATGTGGATGTATTTGCTTTGTTCTTAAACTGAAA

>Ca3:30787152-30787406

GTTTTTGTTTCCACTTTAACAGTTCTTTCTGACATGCCGAGGGACAGTACCACCAAACCAACCTATACTGCGTAAAAAGCAAAGCATAACAAGAGAGAGAGAGAGAGAGAGAGAGAGAGAGAGAGAGAGAAATAAACACCATTGTTCACATAACACAGAGATCTAAAACCAACATGGGGTGAACTTGTTTTTCGCTGATACTGTTTATTTGGAACAGAACAATGGCTTGTTCAACTTTTTATTTTTATTTTTCT

>Ca3:31054527-31055162

CGAAGTGTGGAGCTGTGGAATTTGAAGATGAAGGTGAAGGGAGGGAACTGTAATGTGGTGCTGAAGAAGAAGAAGAAGACATGACATCACAACTCAACGAAGAGTTTGATCGAATATAGTTGAAATGGATCGAAATTGATAATTGAATTAACGAACCCTAATTTTGTATTGTGAAGTTACAGTTGATAACAGTGATTGTGAGAAAGACGGTAAGAAGAAGAAGAAAGTTGTGTGTTGTGGAATGAACGATGTCGTTTTGTGTGTAATAAAAGAAGGCACGAGGGACGAGTTAGGTGTCGTTTACTTGGTAATAAGCAATGCCAGCGAGTGCATGAAATCCACCTGGAAGGCACTGAAGTGTGGTGGAGTGGGTCCCGTGTGATGCAAAACGATAAACAAAGTGACTAACTTCCAGCGAAACTATGCACGATCTGCCTGCATATTTGCGTGTTTATAAGAGCATCTACAACGGGAGTGAAAATGAGTTCGTCCGTCAGCGTGTAATTACTTAACTTCCAGTTTTTTGTGGGTTCACCGTTGGAGTTGTGCCAATGTGTCATCACGGTACTGTCAGGAAGGAACGGTGTTTCATAGCAGTTACGTTTTTTTTTTTTTCTATCTACTTTCACAATTTA

>Ca3:31096186-31097317

CATCTATATGTTCAAAAAATATAAATCTTAAATAATCTCTTATGACTGGAGTTATAATCCCTTCCTATATAAGATCTCAATTTTGATTACTTGAATGGGAAGATGTAAGTTCTGTTCTTAGTTACGTGTACCATGGTTTGCTATGTCGCTGTGTTTTCTTGGTATGGAAATACTAATAACTATAGATTTATTTGGTACAAGTGGTACACTAAGATGCTGGTATGATGTTCTCTGATTTTGATGGTAATTGCGATAAGAGTTTTGGGAAAATGAAATGAGTAAGAAATTTTAGTTCTTTGAATAAAGGAAATTGTAGTTGTGCATGTTAGCTGTTAATGAATGCATTTAAATTCCTGTGAAGTGAAAGATTGTGTTGAAGAGATCAAAAAATGGTAGTAAATGACTTCAGCTCTAGAAGCAATTAAATTAATGAACCCGGCGGGACAAGCTTTATTATTAATTATTGTGGTGATCTACTGAACATTCTGAACGTCAGCAGCTAAGCATGTGTGTACTTTTGGTCTAATCATGTAACTTAGATGTTTCTAAAAAAATTGTATTACATGCTGCCTAATGTTTTTCTTATCCAATCAGCATTTTGGTTTGCCTTCAATGGTTAAAACTGCCACTATTGATTATTGTTGTGGCCGAGGTTTTTGGAAGTCTTATGTTTGTCTATTTGGCAAAAGATCTTGCACAATGAAATTGGAGATTTCTAATTGGTAACGAACCATATATGTTTAGAAGGTTCAAACAATCGTAATTGTTGACTGGTAACCGATACATACAGATAAAATTTTATTGTAAACTGAGAAAACTGGTATGTGAGGGTTCCAAGTGATCCTTTAAAAAAACTGTGTTTCCTTATAAGATATTTGAATTAGTTAGGTTATATGATTTAAGTAAGGCAATGAAAGAAGTCATATGGGATAATACATTTTGCAGAGACAATCTTGAAAGACTGTAGTATACTGGACTATGTAGTATACTGTGGCAGAAATCCTTTGGTCTCTCTGTTTTTCTTCTTTTTGTTAGCAAAATAAATAGATGTTGGAACTAAAATCATGTGATTTTTCACAGATTTTCACATCAAAGCATCCAGACACAGCAACAAGTGAGTTGCAGGGTCTG

>Ca3:31727320-31727612

AGACATTTCACATTTCAACAAAGAATAGAGGGAGGAGATCTCCGATAAAGAGCTTGTTGAAGGCGCGTGATCATGCGATACAGGTTATTACCTACCACTTCTTCTTTTTGTATTCATGTAATTCTACTTTTGGAATACTTCAATTTTGTAACGATCATCTATTTTCTTAGTGTTTGAAACTCGCATAACTGCAAACCTAGGTTAAACGTAAGAGTTAATGTAGTTGCATTTTATTATTTTAGGAAAAATTTGATATGCATGAAGATCCGTTTTTGAGAATTTTATGCTTCAG

>Ca3:31856080-31856368

CACCATTACCTCTGGGAAATATTAAATAGTTTTAAAGTTTTGATTTTTTTCCAAGGGTTTTCATTTTAAGCTTCTCATTGTTTTATCTACATTATTTTTATTCACTTCATTTAGATATCAATTTGTGTACCCTTTTGTTTTCTAAAGCGATATTTGTAAAACTTGTGCTTGAAAACGTGGTGGGGAAGTGCAAAGATCTTTCATTTTTTAGTGAATGGTGGAGTAGCTGTTGGAGGTAGATCAGGTTGCAAGCTGGGGTTTTATTTATCAGATCTCTCTTAGAATATG

>Ca3:31856083-31856368

CATTACCTCTGGGAAATATTAAATAGTTTTAAAGTTTTGATTTTTTTCCAAGGGTTTTCATTTTAAGCTTCTCATTGTTTTATCTACATTATTTTTATTCACTTCATTTAGATATCAATTTGTGTACCCTTTTGTTTTCTAAAGCGATATTTGTAAAACTTGTGCTTGAAAACGTGGTGGGGAAGTGCAAAGATCTTTCATTTTTTAGTGAATGGTGGAGTAGCTGTTGGAGGTAGATCAGGTTGCAAGCTGGGGTTTTATTTATCAGATCTCTCTTAGAATATG

>Ca3:32564079-32564356

TGGTTTTCTCCTTTTTTCTCTAATCTTTTTCTTCTATCTTATGCCCTCTCCTTTTTCTTCCCCATTCAAGCTTTCCTTGCTTCTTCACTCTGCACCATATGCAAAATTTTTATTATATTAATAATCAATTAGCTTAGATCATATACAAGTACATCATCACCATCATATAATAAAAATACACAAGTATATTTTTATTGTTTTCCATAAAAAAAAATACTAAAATCAAATTAAAAAATCGAAGGCCATGGAAGACTCCCCTGCCGCTTATATACACCTG

>Ca3:32573050-32573970

TATACTTATGTAACTCCATGGGATGCATATATAATATAAATCCGGGAATGAAGGTTATGATATTTTCACACGATTATGTGATCTAGTTATTTCATTTTCTGAGGGGATTCCAGGGAGTTCTCATGATCTGGACCAAGGTTTTAAATTGCGGTTGTGATTACATGCGATCTTAAGGGTGTGGATAAATGTGTTTGATGCAGCCGCAATTGTGGTCTCAATACAATCATGTAGGTCCCAAAATCCTAGTCGGTCAATCATGTCAAGCTTCTCAGCTTGTGTGTGTGGTGCCTGTGTTGGGTAGTCAGTACACATTTTTTTACCGTTGTGTGTTTGATACTGTTATAACACAATAAATGAGCACCTTGAGTAGTCTCTTGAGTGCTTCTAAGGTTTACATTTTTGTACGTGTTTACATTCATAGGGTACACTAATAGTAGTTGTTTCCTAACTAATACTCAATATGTTTGAATTGATGTCAATTTTCATGAAACATGTTATGACTTTAAACCTAGTTGAATGTCAAAATGCAATTTTGTTGATGAGTCTGTGGATCTAGACGACTTCATGTATTTTCTCTTAATCTACAAGTTTTCGTCTATTTCTATACCTGGATGACTGGTTCTTTTGCCCCTCATTTTACTAGGTCCGTTCTGGTTCTATGTTGTAGTAAACACTGTAATTACTTCAACACTCAAATCTCAGCGATGAAGTCCTACCATCTAGCAACGGCATACATAGTTGGACTAATCAGAAAAAGGTTTCAGAAGGGCCATGTGAAATATGTAAGGGGTGCGTGAGTTGAGCATGCAAGAATCTGCAAATATCTCCTCTTAGATTTCTCTTAGATTTGATAACCCTCACTTTGTCCTCTACCCCAATACTTAAAAAAAAAAAATAAATAAATCCATTTTACTCATCAT

>Ca3:32573693-32573970

GTCCGTTCTGGTTCTATGTTGTAGTAAACACTGTAATTACTTCAACACTCAAATCTCAGCGATGAAGTCCTACCATCTAGCAACGGCATACATAGTTGGACTAATCAGAAAAAGGTTTCAGAAGGGCCATGTGAAATATGTAAGGGGTGCGTGAGTTGAGCATGCAAGAATCTGCAAATATCTCCTCTTAGATTTCTCTTAGATTTGATAACCCTCACTTTGTCCTCTACCCCAATACTTAAAAAAAAAAAATAAATAAATCCATTTTACTCATCAT

>Ca3:32571218-32572026

TAAGTAGTGGCTGCTTTGGTGTCGTATCGTAGCGGCACCTTGGAGTGATGCAGCGCTGCAAATTGGGGTCGCAATTTGGGCTGCAATATAATAGTTTTTGATGTCAAAACTGCATGCGGCCGCAATTGAGGCTTCAACACCTGCAACAATTGAGTCTGCATCGGCCACATTGGGTCACAATTCTCCATAATTTCAAGGATTGCGACAATAACCATTACTGTGACCGCTATTTAAAACCCTAGCGACTGCTATGATGAAGCTCTTGTAAAAGCCCGAATATGCCCTAAACTTATGTTCATTCTTGCTGTTTTTTTTTTTGTCATTTCCTTCCTGAAATTCTAAATGTCAACTAAACGGGGATTTACTGTCTTTTCATCTCTTCTCCAAAACAGCTTCTTATCCCCCCTAAACCCTAAACGGCTTTGTCTTGCCTCCTCCTTGTGTGACTTTCCGGCAACCTGTTGCAAGACTCTCTGGTGGCCATTGTACTTATTCTAAATTCTAAATGCTTAGATGAGTCATTTTAATCCCCCAAGATTAAAATTAATTTGCTTTTTATTGGTTTTGAATATTGGTTATAATGAATTATGATTATTAGTTATGAATATGCCATTAATTTTGTGTTATCTCCTATTTTTGAGCTTTTACATGTGTATGGTACAAATTTCTTGTCTTGTTTCCCTAAACATTGACGTAGCTGCATTACTGCTATAGCATTTTGAGAGTCACTCGCCGCTATTCGAGATTGACTACATAGGGTTTTTTAAATATATTCTTGTATAAGTTGAAATCAATTTCTACACCTCGG

>Ca3:32620526-32620984

ACCTGTCGAGAGAAATGAGTACTTTGTCCTTGATGAGGAGGAAGAGGATGCAGCTGAAATTGCTCTCAGGGATGAAGAAGAATTTGAGCTTTGAAGTAAAAGTTTCTGTTTTTGGAATTTTGCACTTGCCCTTTTTGTCGATCTCTCTCCCATGCCACAGATATCAGGTTGTTAGGCGAAATCGATACTGTATTTGTGGTGGTTGTGGTCCAGGTTCCTCTTTTTATCGGTTTTTCGCTTACCCTTTTCTTCGTTGTTTCCCTCCAAGCCTTCAAATCAAATGTTGTAGAGAAGTGTCCATAAAGGAATACTCAATACTGTCTGTATGTCTAGCTTTCTTTTGTTGATGAATTATGCGGGATAAACAAAGCTTTGATATATGTATGTCTCTTTAGAAATTGTACACTCAATTATTACTTACATCATGTTCTTTGACTGATAATTCTTCTGTGGATTTT

>Ca3:33059150-33059741

GTATAAAAAATAAAAGAAGAAAAGGGAAAGTTAGAGAATGGGGTTCGTGGTGGTGATATCGTTGCCATTAATATTGTTAATTTTGATATTGGCTATTGCTTGCTACTTGTTGGGTAGGGCCAGGGGTCGCAAACAATCGTCTTCCCATCCTCAGCAGTTTGGCCCACCAGCCCCTCCACCACAAGCCCAACTTAGTTAACAACAGGCCCAAAAATGTTACACATTGGTTTGTTTTTATAATATATTCAAGTAGTTTGCTCATTGCACATGTACTCACAAAATTAGCTTTAGTCCTCATCTACCTTTCACTTCTCTTTATCATAATCTTTTTTAATATACAAAAGGAGTGTTTAAAGTCCATTTTTTTTGTTCTAAAACCACTCGTTTACGTCATTTTTATCTTCTATGTCATTTTATCTAAATAAGTAATTTTTAAATGAATCATTTTTTTCTTTATATTTTTCTGATATCCCGCCAACTAAATGCGAACTTTGTTTTTATGTATCATAAATATTTATTTTGTTTATATATACAAATTATTTTAATTTAATACATATTCAATCAATGGTTTTAGTGTTATCAATATTGTAG

>Ca3:33059267-33059741

GGCCAGGGGTCGCAAACAATCGTCTTCCCATCCTCAGCAGTTTGGCCCACCAGCCCCTCCACCACAAGCCCAACTTAGTTAACAACAGGCCCAAAAATGTTACACATTGGTTTGTTTTTATAATATATTCAAGTAGTTTGCTCATTGCACATGTACTCACAAAATTAGCTTTAGTCCTCATCTACCTTTCACTTCTCTTTATCATAATCTTTTTTAATATACAAAAGGAGTGTTTAAAGTCCATTTTTTTTGTTCTAAAACCACTCGTTTACGTCATTTTTATCTTCTATGTCATTTTATCTAAATAAGTAATTTTTAAATGAATCATTTTTTTCTTTATATTTTTCTGATATCCCGCCAACTAAATGCGAACTTTGTTTTTATGTATCATAAATATTTATTTTGTTTATATATACAAATTATTTTAATTTAATACATATTCAATCAATGGTTTTAGTGTTATCAATATTGTAG

>Ca3:33752226-33752601

GGAAAATAGAAACCCTTGACATTTCCTTCGTTGAGTTATGCGATTCATCTCCTTCCTTCTTCTTTTTGTCTCTGTTCACAATCCTTTCATTCTTTCTCTTCCTCCTTCCAATTCCACCATAAAATCTATCTCAATCTTCTCTTTGCTCTTTCTCTTTGAATCATTTCTCTTTTCTCTTTGTCTCTGAATCACTCCACCCTTGCAACTCTTGAATGAATCGTTTATGGTTTCTCCCAAATCAGAAAAACACACCTAATCGTTCCTATTATATTCGCTCTTTCTCTCTGAATCGTTCCTCTTCTCTCTTTCTCTCTGACTTGTTCTATTCCTTTCACACAAAGACTTCATTTGAGGGCAAGATTACAGGTTCAAAAG

>Ca3:33764567-33764878

TTGACGATGAAGGCGATGAATTTGAAGAGGATGACATAGATGACAAATTTGAAGAGGACAACATAGATGCTAAATATGAAGAACACACACTTGAATGATTTAGATGATGATTTTAGATTATGCTTTTTTGGATGATGTTTTTTTTATTAGGCATGTACTTGATTGAAGCATCAAAACTTCAACAAACTATATATTTTGCTAGACTTAGTACTTTAAGTATTAAACTTTATTTGAATACTACCATTTATTTGTTTACTATGTATGTTACTAATCTTAGACAATTTGTTTATTACCATTTATTTGAATACAAC

>Ca3:33752265-33752601

GCGATTCATCTCCTTCCTTCTTCTTTTTGTCTCTGTTCACAATCCTTTCATTCTTTCTCTTCCTCCTTCCAATTCCACCATAAAATCTATCTCAATCTTCTCTTTGCTCTTTCTCTTTGAATCATTTCTCTTTTCTCTTTGTCTCTGAATCACTCCACCCTTGCAACTCTTGAATGAATCGTTTATGGTTTCTCCCAAATCAGAAAAACACACCTAATCGTTCCTATTATATTCGCTCTTTCTCTCTGAATCGTTCCTCTTCTCTCTTTCTCTCTGACTTGTTCTATTCCTTTCACACAAAGACTTCATTTGAGGGCAAGATTACAGGTTCAAAAG

>Ca3:33764579-33764878

GCGATGAATTTGAAGAGGATGACATAGATGACAAATTTGAAGAGGACAACATAGATGCTAAATATGAAGAACACACACTTGAATGATTTAGATGATGATTTTAGATTATGCTTTTTTGGATGATGTTTTTTTTATTAGGCATGTACTTGATTGAAGCATCAAAACTTCAACAAACTATATATTTTGCTAGACTTAGTACTTTAAGTATTAAACTTTATTTGAATACTACCATTTATTTGTTTACTATGTATGTTACTAATCTTAGACAATTTGTTTATTACCATTTATTTGAATACAAC

>Ca3:33764540-33764878

GTAACAATGACGATAAATTATTTCAAGTTGACGATGAAGGCGATGAATTTGAAGAGGATGACATAGATGACAAATTTGAAGAGGACAACATAGATGCTAAATATGAAGAACACACACTTGAATGATTTAGATGATGATTTTAGATTATGCTTTTTTGGATGATGTTTTTTTTATTAGGCATGTACTTGATTGAAGCATCAAAACTTCAACAAACTATATATTTTGCTAGACTTAGTACTTTAAGTATTAAACTTTATTTGAATACTACCATTTATTTGTTTACTATGTATGTTACTAATCTTAGACAATTTGTTTATTACCATTTATTTGAATACAAC

>Ca3:33753283-33753902

AACCTTTGATATTGCTTTTTCAGACCCAGAACCAACGATTATTCATAATCAATAGAAAGCTGAAAAAGACTTCTTGAGAGAAAAAGAGAAAGATCTCACAAACACTCAAATGGGCAAACAACTTCTTGTGTGAGATTCATTGCAAGTGATTGAGACATATTCTGTATATCTTTTATATTTTCTGTAACAAAGATTTTGTAAAGAAAGAACTGAGAAAAGTCTAGTAGTGAAACTGGTAGTTATCTTCATTGTTGAGAGGGCTCATCAAGAAGAAGCTTTACTGTGATCTTGGAAGAGTTTGTAGAGAAACTCAGGGATACAGTGGTTGCGAAATAGAATAGAGAGAGAGTTTTAAGATTGATAGGTCGGGAGCGCTGGAACATCTGTAAAACACTCTAGAAGATTCTATTGAAAAGGCTGAAATAGTTTAATTTCGGGACTGGACGTAGGCCGTCAGATAGACGGTTGAACCAGTATAAAATCGCGGTGCACTACTCTCTATCCCTTACTCTTTATTTCCATCTTGCTTTGTATCATTGTATGATTGTATGATTAATCTTGATTGCATGCTATAGACGTATTCTGTTTTCATTGATATCGCTTGAATGGTTAAATTGGC

>Ca3:33894765-33895010

GAAGATGGTTGAATCTCAAGTCTCAACATAGGTTGTTTGGGCATATGTGAAGGAGATAGAAAGTAGAGTAGTAAGTAGAGTATATCAGGAGACATGTAGATTCCGTAGTAAGTAGAGTATATCAGGGTACCCTAATTAGGGTATGAATTAAATATGAGAAATAATTATCTCTCCTTCTTTATGAATTAAATATGATTTTTGAAGACAGACATGTCATGTGAAAATGAATATTATATATATTAGAC

>Ca3:33943904-33944144

CCAAACACCCATCACCAGACCGACCCGGTGACAAATACCAACCAAACGACTGAGCCATCGGCGGCGACTCCGGCGGAGAAGAAACGAACGTCAACATTGAAACGCAAGCAATTAGTACCGTCCTGCATGGCTGAGTACAATTCCCCTTTCAGTAAAAATGTCTTTTCACTTGGAAGTTAAAAAGAAAGGGTCGTAAAAGAGGAGTATGGGTGTACACCCATAATTTTTCAAACAAAATAG

>Ca3:33946540-33946946

GTTCTATGTCTATTAATGTCTGTGACATTGAGCAAGAACAACAACATCAATCCAAGAGCAAAATGATGGAGTGACGGATTCTCTCCAAATTGTTACTTGTAACCATTGTGGCACTTTTATGCACATAATATAATCTCCATGAGGGCACCACATTTGTAATTTAAACTCAAGATTCAATTTATATAGTGTAGGATTTATATGTTATTTTTAGGTTCTTATATGTTAACGTCGACAACAAGAAAATCAATGCAAGAGCAAAATGGGCGAGTGATAATAGCTCAAAGATAGATATTCTATCCAAGTCATTAGTTGTAACCATTGTAGTATTTTTTGTTATCATAATATAATATTCATAAAGTCACCATATTTGTAATTTAAATCCAAGATTCAATATTTGTAATGTAGT

>Ca3:33946413-33946946

ATCTTGTGATAAGATAATTGTGAATAGGATGGACCGACTATAATTGTATGTTCATCACTTGAACTTTTGTTTCCAACAAGAAGTATATAGTGCTTCAATTTGTTTTAAAATTGAAAACTATTTTTAGGTTCTATGTCTATTAATGTCTGTGACATTGAGCAAGAACAACAACATCAATCCAAGAGCAAAATGATGGAGTGACGGATTCTCTCCAAATTGTTACTTGTAACCATTGTGGCACTTTTATGCACATAATATAATCTCCATGAGGGCACCACATTTGTAATTTAAACTCAAGATTCAATTTATATAGTGTAGGATTTATATGTTATTTTTAGGTTCTTATATGTTAACGTCGACAACAAGAAAATCAATGCAAGAGCAAAATGGGCGAGTGATAATAGCTCAAAGATAGATATTCTATCCAAGTCATTAGTTGTAACCATTGTAGTATTTTTTGTTATCATAATATAATATTCATAAAGTCACCATATTTGTAATTTAAATCCAAGATTCAATATTTGTAATGTAGT

>Ca3:34352123-34352514

TATAACGTTACACCATAATCCAACTGTTTTTGACACACACAAAATTCCAGCTATTCATAAAAAGTAAATAAAGGGTCCGGTTGTGTACACTTAACATATTGTGGGTTTCGAGTGTGCTTCACGGAGAGATAAATAGGTGCGAACATGACGAATTGGAGAAAAAAATAGCTGCGAACGAACACGACGAATTGGAGAAAAAAAATAGCTGCGAACGAACACGACGAATTGGAGAAAAACGAATTGGAGAAAGAAAAAGAGCTGCGAACTTGACGAATCGGAAAAAGAAAAGATTGGAGCTTCTTGATTGGATTTTGATTATTCTGAATTCGTTCTAGTTCGTTCTTTGATTGATTTCGATTGTTCTTACAAAATTGGAGAAAGAAATACAGAG

>Ca3:34360802-34361352

TGTTGCTTCTTCTAATGAGATTATTGAGAGGTTGGTGTTTAAGGTGGTGAGTTATTGAGAAAATGAGATTGTAGATGATTTGAATAACATGAAATAGAAAAGAAACAAGAGAGATAAGAGCTGGTTTGAGTTTGCATCTAGTTTGGGTTTCGAGTCTGCTTCACAGAGAGAGAAATAGGTGCGAACATGACAAATTGGAGAAAAAAAGAGCTGCCAACGAACATGACAAATTGGAGAAAGAAAAAGAGCTGCGAACTTGACGAATCGGAAAAAGAAAAGATCGGAGCTTCTTGATTGGATTTCGATTATTCTGAATTCATTCTAGTTTGTTCGTTGATTGATTTCGATTGTTCTTACGAAATTGGAGAAAGAAATGCAGAGGTGTGTTCTTAATTTTCGATTTTCAATCTTCTGCACTTTGAATCAAATTCTTTATAATTGATTTGACCTGTCATCTTCGTGTTTGGCATTGATGTTTCGAGGCAACTTTTTCTTTTCTTTTTCATTTGCGGCGGTAATGGTAACATACCGTTCTTGAAATTCAAACAAG

>Ca3:34360443-34361352

GAAGATGTGTTATGTTTCATCAGATTGGTGTTATCCAGCTATTTATAAAGGTACTGAAATCTCAATTTAGCTTTTATTGATTTGATTTCTAGGCTATGATATGATTTCTAGGCTATGATATGATTTCTAGGCTATGATATGCAATGAAAGTTTAAAGACCTGACAAGCTCTACATCTTTCATTTTATAAGTACACTTTTTACCAAAGTAAGTTTAATAAAACCTAGAATAGTCTTTTGTATGTGTCTGTGTGTTCATGTAATACCTTTCATCTATTTTGTTGTGATGTTTGCTTGTTGCTTGTTCCCTCCATTTGTTTTTTTAATCTTCTGTTTGCTACTTTTGATTGTTTTCTGTTAGTGTTGCTTCTTCTAATGAGATTATTGAGAGGTTGGTGTTTAAGGTGGTGAGTTATTGAGAAAATGAGATTGTAGATGATTTGAATAACATGAAATAGAAAAGAAACAAGAGAGATAAGAGCTGGTTTGAGTTTGCATCTAGTTTGGGTTTCGAGTCTGCTTCACAGAGAGAGAAATAGGTGCGAACATGACAAATTGGAGAAAAAAAGAGCTGCCAACGAACATGACAAATTGGAGAAAGAAAAAGAGCTGCGAACTTGACGAATCGGAAAAAGAAAAGATCGGAGCTTCTTGATTGGATTTCGATTATTCTGAATTCATTCTAGTTTGTTCGTTGATTGATTTCGATTGTTCTTACGAAATTGGAGAAAGAAATGCAGAGGTGTGTTCTTAATTTTCGATTTTCAATCTTCTGCACTTTGAATCAAATTCTTTATAATTGATTTGACCTGTCATCTTCGTGTTTGGCATTGATGTTTCGAGGCAACTTTTTCTTTTCTTTTTCATTTGCGGCGGTAATGGTAACATACCGTTCTTGAAATTCAAACAAG

>Ca3:34352146-34352514

CTGTTTTTGACACACACAAAATTCCAGCTATTCATAAAAAGTAAATAAAGGGTCCGGTTGTGTACACTTAACATATTGTGGGTTTCGAGTGTGCTTCACGGAGAGATAAATAGGTGCGAACATGACGAATTGGAGAAAAAAATAGCTGCGAACGAACACGACGAATTGGAGAAAAAAAATAGCTGCGAACGAACACGACGAATTGGAGAAAAACGAATTGGAGAAAGAAAAAGAGCTGCGAACTTGACGAATCGGAAAAAGAAAAGATTGGAGCTTCTTGATTGGATTTTGATTATTCTGAATTCGTTCTAGTTCGTTCTTTGATTGATTTCGATTGTTCTTACAAAATTGGAGAAAGAAATACAGAG

>Ca3:34352156-34352514

CACACACAAAATTCCAGCTATTCATAAAAAGTAAATAAAGGGTCCGGTTGTGTACACTTAACATATTGTGGGTTTCGAGTGTGCTTCACGGAGAGATAAATAGGTGCGAACATGACGAATTGGAGAAAAAAATAGCTGCGAACGAACACGACGAATTGGAGAAAAAAAATAGCTGCGAACGAACACGACGAATTGGAGAAAAACGAATTGGAGAAAGAAAAAGAGCTGCGAACTTGACGAATCGGAAAAAGAAAAGATTGGAGCTTCTTGATTGGATTTTGATTATTCTGAATTCGTTCTAGTTCGTTCTTTGATTGATTTCGATTGTTCTTACAAAATTGGAGAAAGAAATACAGAG

>Ca3:34352162-34352514

CAAAATTCCAGCTATTCATAAAAAGTAAATAAAGGGTCCGGTTGTGTACACTTAACATATTGTGGGTTTCGAGTGTGCTTCACGGAGAGATAAATAGGTGCGAACATGACGAATTGGAGAAAAAAATAGCTGCGAACGAACACGACGAATTGGAGAAAAAAAATAGCTGCGAACGAACACGACGAATTGGAGAAAAACGAATTGGAGAAAGAAAAAGAGCTGCGAACTTGACGAATCGGAAAAAGAAAAGATTGGAGCTTCTTGATTGGATTTTGATTATTCTGAATTCGTTCTAGTTCGTTCTTTGATTGATTTCGATTGTTCTTACAAAATTGGAGAAAGAAATACAGAG

>Ca3:34352184-34352514

AAGTAAATAAAGGGTCCGGTTGTGTACACTTAACATATTGTGGGTTTCGAGTGTGCTTCACGGAGAGATAAATAGGTGCGAACATGACGAATTGGAGAAAAAAATAGCTGCGAACGAACACGACGAATTGGAGAAAAAAAATAGCTGCGAACGAACACGACGAATTGGAGAAAAACGAATTGGAGAAAGAAAAAGAGCTGCGAACTTGACGAATCGGAAAAAGAAAAGATTGGAGCTTCTTGATTGGATTTTGATTATTCTGAATTCGTTCTAGTTCGTTCTTTGATTGATTTCGATTGTTCTTACAAAATTGGAGAAAGAAATACAGAG

>Ca3:34359727-34360313

TGCAACAACTTTCCAAATGTTCATCCAAGGGTCACTAGGTCATGGGTTTTCTTTAGTAGGACATCTTCTCTAGACTTACGCAATCACAATTATTGAGTTTGAATTGGTTTCTTCAACTCGTGTATGGACAAGCAATGTAGTGGGGGATCGCTGCTTCTGTTTGTAATGCCAGTTAGGAGAGTCTGAGAAAAAAGATATAGAGAGAGAGGTTGAGAGAGAAAAGGGGATGGGGAGGGAGACCATCTGAAGGGTCCTTCTAATTGAGGTGTATTGTCTGATTGGTCCGTTAGTACGCTTCATGCACGTTTCCGATAGACACAACCTCCAGCAAGTGAATTTTCAAATACCAGCGGGCAAGCTTGTAAAAATGTGCAAGTGACTATTGAGAGTATCGATAGACTTTGAGGAAATTCACTTACCAACGAGCATGTCGGTTGAAAATGTGGTAGCAAATAAAGGGCACCAAGATTAGCAATAAATTGGTTATGAATCAGAGACTAGAAGTTGATTCCAAATGATGTCCATATCTAGAGATGGCTCTTAGTCTTAGTTGAGAGCTTTACCACTCCCTAACTTAGATTTTGAG

>Ca3:34575056-34575537

NNCAAAAATATGGTGACATGGCAAGGAAAGCATTAGAAGAAGGTGGTTCTTCTTACCGGAACATGACTCTACTAATTGAAGATATAATGCAGCATGTAGAGAACAAAGTTAAAGTGTAATTGAAACCAACTCAGTGATGACGATGTACTTGTTTTATGCTAATAATTCATTTTAAGAATCAAGTTGTCTCCTAGATACTTTATCCACAATGAGTCTTTTACTAATGAAGGTGAGTCTAACCAAAAAGAATAGATTTTTATAATGTAGTAAATTTGAATTTATTTTGAAAAAGTTACTTTATATATAATGTGTGACACACTTTCAATAGAATTATTTTCTTAAAAAAAAATCTATAAAAATAAATAAATATTTTGATATAATTTTTATAGAGATATAATGATGTGAATATTAATCTCCAATATTTTTTTAAAATTGTTTTATAAACAATGTAATATGCCTAGGATAAATTGTTTATAGGGAT

>Ca3:34575624-34576026

ATAATATGAGAAGTTATCTCCTTAAAAACTTAAATGCCTAAAGATTATTTGTTTTTCAAGCCTTATAAGAAACATGAAATCATGATTATACACGTTGTTATCAATACAGAAAAATGTTGGGTGTATATTATGAGGCATATGAGAAAGAAACAAAAAAAGTAATATGATAAACAGAAGTTGGTTCAACTTGTTTTTGATCATCTCATTATGAAACAAACAAAAGTGAGAGTGAGAGGAGAGAGAAAAAAAATATGATGACAAATTGAAGAGAGAGAATGAAATGATTAAGAGAAGAAGGCGGAATCGGGCGGTGGTGGGTGGGTTGAGATGACGGCTGCCGCCTGCGGAGGAGAGAGGCTGCACAGCAATCTCTTTGGAATTTCTTCTGTTCAACTCTCACAG

>Ca3:34575743-34576026

GGTGTATATTATGAGGCATATGAGAAAGAAACAAAAAAAGTAATATGATAAACAGAAGTTGGTTCAACTTGTTTTTGATCATCTCATTATGAAACAAACAAAAGTGAGAGTGAGAGGAGAGAGAAAAAAAATATGATGACAAATTGAAGAGAGAGAATGAAATGATTAAGAGAAGAAGGCGGAATCGGGCGGTGGTGGGTGGGTTGAGATGACGGCTGCCGCCTGCGGAGGAGAGAGGCTGCACAGCAATCTCTTTGGAATTTCTTCTGTTCAACTCTCACAG

>Ca3:34711113-34711364

ATACAGGTATGAAGCAGATACTTTTTTTGAGGAAATATGAAGCAGATACTTTTTTTGAGGAAATATGAAGCAGATACTTACCGTAAGTTACTATATATCATAATGAAGGAATTCTGAAATCGAAATTCAACTATACGTAATACTTTTTTTCCTACGAGGAATTGATTTGTTAGGTATATGAATAAGAAGCTTTTGATTATTTTTTCACGCACAGTATACAATTTTCCCCCTAGAAGTTATTATTGCAGAAC

>Ca3:34825389-34825993

ATCTACTAGAAGGATATGATTGGATGGAGGATTTTGGAGGAAGATGGAGGGCATCATTTATTTATTTTTTAAAAATTAAGTTATAAAGTGCTTCTAAAAATAACATATCCGAAATTAAAATATTATATGATCATTTATTATGTTATTTTTATATCTCAAATATACACCAAAATATAGACTTATCTCGCTCTAAAACCATCCCTTGTAAAAACAAGACATCCAAACATACTTCAAGGGTTTGTTTGGTTCAACCAAGGGGAATTGAGGTAGATGAGGTGAGGGATTTTTAATGGTTTTGTGTTTGGTTTAAGTGGAAGGGAGGAATTTTAAATGGTTTTTATGTTTTTTTATTCTCTTCCCTTCAAATTCCTCCAAAATAGAGAGAGAGCAAAATGAACCCTATTAGTGATTTTGGCTCCTTCTCCTCTCCCCCTCCCTCCATTTGTCTCTAACCCAACACACCCTAAAGTTTCCTATGTAAAATGGGTCTAATCCTCTAATGAATTTTAACATGGTGGAATCTATGTAGCGAGGGTTGTCAATTGTTAATATAGTCTGATTTATGATATTGTTAATAAGTTTAAATAAGCAACTCCTGATTTTT

>Ca3:34931576-34931912

TGAAAATCTTTTCAAAAATCTTTTATTAAGGGGAAAACGACACAAAATCAATCCCCTCACTCAATTTAGAGATACTCTTTATGTAGCCAAGGAGAATAGGTACATATTTTTTAATTTTCCCGAAGATATGAAAATAGGGTATATAGATTCTTTTTATTTACGTACAAACAAATTGATATGATATATGTATGTATCGGGTGAGAGATGGAAGAAAATGGAGTAATAGACAGAATTTTGGAGCAATAATGAGGCATGTTCAATTGTTGATTAGGAATGTTCCACCATACTGCAATTGTGTGCATAACTATGACTATATTCGGCAACACCGTCTTTCAG

>Ca3:34932214-34932886

ATCTGTCAATTCATAAAAATACCATCTAAAAGAACCGATCTGATAAGGATATTTTTGGGTTTTTCTTAGGCCTTTGAACATATAGTGAGGGGTGGGATGAGTAGTTTTCTATTTTCCCGTCCCGATCCATGCTCAAATGATTACCACACTGCAATGGCCCAAAGAAATATAGAGCCCAAGCCCATACTCCAAAGCAATGGCCCGAAATATACATATAATGAGAAGGCGATACTGCAGTGACCAAAAATAAAAAGTTGGAAAATTGTTGTTAACTTGCTTTGCACCTCGTTTGCAACACAGTAAGTCACATAATTTTTTGGTTATGGTTTTTAATATTACTAGCAATATATCCGTGCACCTGAACGGAACGGACCGCACGCAAACGGGATGGGTTATTGTATGAAAGTAAATAATATACAAATAGTCCAAAAATTAAATTGAATTGAAAAAATATACGAAAAAAATTAATATAATTGAAATGATTTTCCCAATCTGCTAATTAAGTAATGAAAAAATACATTAAAGGTTAATTATTCTATCAATATAACTATTGCAATCATTATTATTTTGTTCTTGCTTAGGTTTCAAACAAAAACCCATATTTAGTATATAAGTGAAAAACATTTTTCATATGTATAGTTTGTATAGAATAAATTTTGAACAATGTATTAT

>Ca3:34931617-34932091

ACAAAATCAATCCCCTCACTCAATTTAGAGATACTCTTTATGTAGCCAAGGAGAATAGGTACATATTTTTTAATTTTCCCGAAGATATGAAAATAGGGTATATAGATTCTTTTTATTTACGTACAAACAAATTGATATGATATATGTATGTATCGGGTGAGAGATGGAAGAAAATGGAGTAATAGACAGAATTTTGGAGCAATAATGAGGCATGTTCAATTGTTGATTAGGAATGTTCCACCATACTGCAATTGTGTGCATAACTATGACTATATTCGGCAACACCGTCTTTCAGGTTCGTTTTTTAGGAACATATAAAATTTAATAAGCCCAGCTTTTTGGCTTAACTTAGTGGTTATTACTTCGTGATTTTTATGCACGAGATAAATTTTAAATTGGTGTATGATCGAATCCAATGTTTCTTTTTTGTCCATGGAATCTTGGTGGCGTTGGGTCGTGGAAGCTTCTTCCTGG

>Ca3:35111955-35112469

AATCAACACCGTTCATTAGAAAAATGAGTATTCAGACAAAATATAAATAAAATCTCAATTCCATAAATTTTCATGTACGTCTAGTTTCAATGGATGTGGAATAAGAAATTAATTAAATTGATAACTATAATTATAAACGTTGAGCTTTCCCAGCCGTTACGTTCTTAACCACCACACCACTACCGTCACAGCCAGCCTGTCTGTTGTTCTAATCCCATACATTTCTTATTAACTTATAAATAATTTACCTATTTAATTAACACTAATAATTGAATTATTACTTTCACCTTTCAAAAAACCCACAAACACAACTGTTTACTCCATTAATTAACATTTTGTTGAAAACGATCAATACCAATTTGTCTCGTTTCGCCGTTTCTTCCTCTTTCTCTCTCATCATAACCTTTCAGACTCTGTTATCATTTCCCTGTTAAGATTCTTTCTCTTCCCTTTTCTCCTTCTTATCTCTTCCTTCTCAATTCCACCACAACCACTCCTTCTTATCATCAATCAG

>Ca3:35408824-35409240

TGTATTTAAACGAAGCAGCAAGAAGCGGTGGGTAAAAGGGGACAAATACGATAGACGGTGGAGGGGTCAAAGAGAATGCTTGGAATACCCCTTAGTTATGTGTTAATTACGAGACTACCCTTCTACCTCAGTGCACTTACTACGACAACTTCAGATTCACGGGCCTGCTCGGTGTTCGTTCATTTTCACTGGCAGCAGAGCTCGCACGGCTCGTTTGAAACGTGCATTTATTAATATATAAAAATCATGACTATTAATGAACAGAGGTTGTATTCTGTTAGGTACTACAACGTGTTTATCCTAAGAGATAGTATTTAATATACGATTACGACGTATTTCCAATAAAATTATCTTTAAAAAAAATTATGAACACTTATAAATAAGATAAATTGTAAGGTCTCAATCCAAAAAATGCG

>Ca3:35471819-35472288

GAGATGATTAAGGGTTCTGCACTTTGTGATCTCCCATGACTTTTCCTTCCTCGTATGGGATTCATGCTGGCTACATTCTCGAGAAATTTATAGCAAATTTGCGCCACCATTTAAATTCGAAGGTTAGTCTCAAATGTGAGGTGCTTCAAACAAACAATATCAAAGCTGAGGAAACATTGTGAAAGGATATAAACTGATGTTTCAGTCTTATATTGACTAACTTGGTGAGGAAACAAAATTCATATTAATATTAAGGACAAGTAGTAGAAAGAGATAGATATAACCTGGTTATTTTCTTTTGAATGCTACACAAGCTTGGAATTTTACTGTTCTTAATTTTACTTTGGGAAATACATGGCTTCCTGATCTAGATATACCTTACACTGAAGGATTATTGAAATAGATATAGGTAAGTAGAAGTTTATGTTTGAAAATGTATAATTTTGGTTCATTTTGTTTTTGTTAGATG

>Ca3:35519587-35520327

TGATTTGTTTAACATAAACAGTTATGTTAGAGTTGCACTTAGTTCATGACGATTGATTCAGCTTTAAAATGTCAAGCCACCCTGTCAAAAGGTAAGGTTGGTTAGTCTGTTTGGTGACTTCTTTCATCTTTGTAGTTAAACAGCATAGCATGGATGGTATGTAAAATTATGAACTAATACTGTTTCGACTATTAGTATATCCTTTATTGTTTAGTTTTTTTTTTTAATTACTTTTTCCATTATCCAGTCTTGCTCTAAGAAAGTCATCTCTCTTTCTCTCTCACTAACTTTTAATTGGTTGAATAGAGATGGAGTGTTTTTCATTTGACAAATTTCATGAATCTTTATTTTTCATTTGAAAGTGACAAATAAGAACTGCTCTTCTATGTTTTTTTAGCTGTTCCCTTTATATAATATTGAGTCCTGCAGCATTTATTGAAACAAATTAAAAATAGGCACAGAAATAATTGGTTTTCCATATTTTTGAAAGCTATTAGTAAATAATTTAGTATTCTATGATCTGACTGAAAATTCATTTTGTTCTCCTAATCCATGTCTCCGCTTTTAGGAGAATGATATCATGACTGCAAATATGGTATCAGTTCTCCTTGAGTATTGGTTGGAAACTGGAAATAACACCCACTGTCCACCTTTCATTTGGTTGACTGCTTTAATAGTGCCATGGGAACTTGCTACTGCCAGGTGTATAACACGTCAAATCTTATATTTCAACCTCTGAG

>Ca3:35652910-35653604

CGATTTCTTGGTCTGTTTTAAAGGTTTTAGTAGACACGGGGAGATTGATATTCATTTTTTGGATGTGATTCAGTCTTCAGTTCTTTATCTGGTAGCTTTATGTTGTGATAGGGTGGTGAATCTTGTGATTTTCATAATTCTATTGTCCTCATCTAAGCTGTTTGAAGGGAGTTTACATCTCTGATTTGCAGAGACTGGACAGTGGTTTCATACTTTTTTTCATTCTTTCGTTTTCCTTTCTCCTTGTTTTGGAGGATATCTTATCTTCTAGTTGATTATGACTTATTTTATCTCTAATATAACTTTTCAATCATAAAAAGACTTAATTTGTTCATTTATCATTTCATTGTAAATTTGCTTGAATTTTACATCCATCTAGTCCACATTGTAACAGAGTGGAAGCTATGTGCATGCAAGGGGGTTTTCATTTCATCATGTGTATAGACATACTATGGTAATGGTCTCTGTTGATAACTTCTTATTTGCTATGATCCTTTCAGTGACTGTTTGAAGTGCTCTGGATATAACTTTTTTTATCTTCCACGTCTGGAATATGCAGCTATAGCTCTGCAGTATTTTCTTGGTAACCAAGCTGAAACATCTCTGTATTCTCTTTTGGTACTGAAATTATGTAGCTTTGGCATATCATGATACTTATTTTGGTTAGAAGCTATTCATTCTGGACATACTCAAG

>Ca3:35654507-35654862

CGATGTTGCCTACACATCTCTATCTTCTCTGCATCTACTAGTTTCTACGTCCATGCATAACAGAAAATAGTTCAAGGGGGGCAACTGAGACTCATCTGCGATTGAGGCACATGTAAAGTTAAAAGGTGTGGTGGATGCAAATGTTGCCTTGGTATTGAAGGCTACTCCAGTGGTCCTGCGAATGTAAGTATTCTGATGGAGGATGATGTACTTATGCTCTAAAACAACAACAACACACTTTCCAAAAAGTGCTAAACATTGTAAATGTGTTCATTATCGTGATTTAAATGCAGGAGTTGAATCAGTTTTAATTAGTTAACCTTATTAATGAAAACCAAATACAACTAAGTTCCAT

>Ca3:35879601-35879860

CTACATTTTATAATGAATAATTATAAAAAAGTTGTTTAACACACAAATAAATAAAATTACCCCCTTTGGTTTTGTACCATGAAAATGCAACTAATCCTCGCCTTCCACAAACAGTGATAAAGTTTTTTTTTTCCTTTGTATTTCTCAAACATTTTTCTCTCTACTATCTTCTACCAACCTTAATATCCTAAAACCCTAAATTTATATTCGAAAAACCCTAAAATCATCTCTCGTTTCACTTCGCGATTTGCAGCAGCAA

>Ca3:36045697-36046059

CCTAGCTCCATCAAATCAAAAAAGAAAATGCAGTCTTGATGCTTTGCTCTCGTACAAGAAATTAGAGTCACAAGTTGGATTGAAGTCGCACTGAACACAAACAATTGGCCGAAAAACGAGGCCAGACTTCGCCAGAAACAGAGCGCAACTCCTCCAGGTTTTCCTTTCTTCTTCATTTTTCCGATAAGCGCACCATAGAACTGCGTCGCAGCAGAGCATACCTCCACTCCGTACCCCGATTCCGCGCGATTGATAACATAATACCTAACGGAAAATTAATTAAAGCTCGTCTCAAATCATTCATAGTGTAGATTCAGTAGCTTGTTTGTTCTTGTAAATTTAAGAACATAACAATAAAGGGT

>Ca3:36093683-36093927

AGTAATTGTGCAGGTTGGTTAATAGAAAAGATGGAAGAGAGTTGCATACCTACTGATGAAGCGGAGAATATGGGACCCGAGAAGCTTTAGTCAATCGGAGAATGATTTGGCGGCGGAGGAACGTTTGATTGCGTCGTTGAAATCGGTTTCTTCGTGGATGATATGATCGAAGCTTCGGTCATTTTAGACGTACTTTTGCTATTCCACGCAATTGGTATTTGAACTTCATCAATGGAAGCACGCC

>Ca3:37088835-37089184

CGGAGGGAGAGGTGGTGAAGGAAAACGGAATCTACACCGACACGCGCTCTCTCCGTTAAACAAACCAGATCTCGTGGTAACCGGTAAGGGTTGGTAATGACCGGCAGTATCCGTTATTAGATTAAATCCCCTCATTTTTTAGACTCACCAACCCCTTGATTTGAGCGTCAAATCTAACGGCATACCGAAGGGTCCGGTAACACGTAATTTAGTTGGCATTGTTTAGTATAAAAAGGAAGATAGAGTCGTTGAACAAACATCCGTCATTTGATTTTCATTTCACAACGTAGTCTGGTCTAGCGAGTGTCGTTTCATTTCATTTCTACTTGCACCCCCTCACTCGCTCCAG

>Ca3:37369249-37369473

AGCTAAGAGAACTTGAAAAAGTTGAGACAGAGTGTGTAATAAGTAGCATTGAGGACACCTATTGAATGACAAGGCAAACACAATTTTAAGGATGTCAAAAATCTTAAACTAGCTTCTCTTCTTCATGTTATGCTCTCAACATTTTGGGATATTTGTTCACAAACACCATAACATTTCAACTTCATAGTCCCTTCTATAACTTAACTTTCAATATCTCTATAATG

>Ca3:37459389-37459744

TTGAGAAGTTTCAATATCTTCTTTCTGATGAATAAAATGTGATTGCTCGATTGCTTGCAAGCTTGGGTAGTTGTGATGGTTTCGGTACCATGGACTGCAGGTTTTGCCGGTAAACTACAGCAATTGCTCATATTAGCTTTGCTGAGTAGAATTCCCCCGTAGTGAGTAACTTCAATATTGAGATCTATGGATTTATCCTTGTAAATATAATTGTGATTCTCATGCAATCTAATTATCTTAACCGCCTAATGCCAACTGCTTCTTCAGGTTTATATCAAATTCTACCATAAACTTGAGGTCTATATCAAATTCTACCATAAACTTGATGTTTGGCAATGATATATTTAATAAGAGT

>Ca3:37919150-37919355

AAGTGAGAGTTCTTCTGATAGCGAGCAAGAAACATCTCATCGTTCAAAGAGGCACAAAAAAAGAAGTGAGAGTTCTTCTGATAGCGAGCAAGAAACATCTCATCGTTCAAAGAGGCACAAAAAAAGAAGTGAGAGTTCTTCTGATAGCGAGCAAGAAACATCTCATCGTTCAAAGAGGCACAAAAAAAGCGATAGACCGAAGAAG

>Ca3:38479801-38480154

TACAACTTGTTTAATCTACACTATCACTCATTTATCTCTTCGTGCATTTTCTTTCTTACAAGCATACACACCACTTGAATCACGGGCTAAAGATGCCAAAATCTAATATAGCCTGCTGTTAGAAACAGAACCTGTGGCAAACAGCCTTCTCACTATCCCTAACTATCTATAATTCTTACACATATCCTGCTCGATCTTCAAAAGGTTTTCAGTTTTACAATACTTACATACAAATTACAAGTTTCTTGGCTTCCTTCTCAATGCCATTCTGGTTTGGCGTTGTATTCAGTTGGCGTGCTGGGGGCAACAGGAACAAAACCCCTGCCACCAAAAACTGGACGCATAAATGCATC

>Ca3:38617310-38617921

GTCATATAAAATAAAATAGATTTTTAAATAGAATAGCAAGCCATACTAGACTTTTATCGATGTCAAAACCAAATCTAAAAAATAAGTTTACGCCATAAACTTGACTTAGATGTTAAATTTTTTTAATGGACCAAACTCAAACCGTGTAAAATCTAACTTATTCTCACCCTAATAAAAACACACAGTAGAACATTTAACCATAATGGACTACAACCCTACAATATCCCTCCACCTATCTCTATATGTATTAACTTTTTAAATATTAATATTATTTATATTTTATTTCGGAAGTAGTATATCAAAAACCAAAGACCCAATACACCCTGGCAAAATTGTCTTCTCCCGTAAACCCTAGAGTAAGAACTTGTAAATCAGTCTTCTAACAAACCTCCGTACATCCTATGTCATAAGCAAAACGTAGCACGATGAGGAACGAAACCGACAGTTGCATCTTCACAGGAACATATGAGCTCTTTTCCGTAAATTTGTGCACATGTATTTCTATCTTCATAGCAATAGGAGTTTCTATCCCACCAAATATTGTATAGCATTATAGCTGCAACTTATAGGACATGTAAATGACCTTTTGACAAAGCTCTTTCTATCTACAT

>Ca3:38728992-38729281

AAGATTATTTGTTGGGGACCTTGAAAGATTAGACTTTGAGTTTTGTCTTCAAGATCTACTGAATAAGGATATATATATATAAACATGTGGGGAAATGGAGCTTCTTTACTTGAAATCTGGTTAGAAGCATAAAGAGTAGTGGAAAGGAATGTCGAGAGAGATCCACAGATCAAACCCTATAAGCTCAACTCAAGAAAGGGAGGCAATGACAGGTGGAAGGGCAAGAAGGAAAAGGAATGTTGTGAGTGTTTTTTTCATTATTAGTATGATTTTTAACAATATGTGTGAG

>Ca3:38796502-38796890

GTTTTCATTCAAGCACACTCTATTAGCCTGCTTAAATAAAAAATCCTATTATTAGTTTCTGAGGCTCAACAAAACAAAAAAGACGAGAAAAAAAAGATAGTATATTTTAAAAAATGAATTTTAGCACAAAATATAGTCATTTTTCTCTCTTCCTTTTTTGGGCCACATGGCCCAATTCCTGATACCTTCTTTCCCCTTCAGCTATCTAAGAGTTACATTTGCCTCTATTTTTTCGAAACCGCCGTAGAGACGCCGCATCGCCGCCGGACGGCTGCTATCACTGCCGTTGGGCCGTGATTTTTTGCCGCCTCCAGAAATTGCGACGCAACGGCAGATTGCGTAGCGCCTAGGGGTCGCTACGGTGCTTTCGACGCTATTAACTTCAAAG

>Ca3:38801535-38802334

TTCAAATGAACTTTGGATCTTACATGATTCAAACCCAAGTAAACCACTATCAAGCTTTCAAAAAGTCAACACACCTATTGACTGAATTCACAATTCCTATTGCACCTTCTTCATTGACGCCAGCACATTGGACATACGAAACCAAATCCTTGAAGTTGTTATTTGCACCTCTGCATGATAAAGAAAACCTACTCCTCCCAATAATTCTGTAATGTCTAGATTCACAAAATTATAATTGATGATAGTTTTTTATTTTTTATTTTCTTTATTTCGTTATCTTAAATCACAATTTTATGACATATTGGTTAAGATTTATTGTTTTGAGATATAATAATCATAGTGTAAAATTTCAGTAATTTTAGGAGTTAAATGATTTTGTTTTTTCTTGTCAAATCTTGAATCTAATTGAGTGTTTAAGCCTTCATTTTCACGTTAACCATGTCCTTTTTGTAGTTTTGTTATGTTTTCATCACCTAGCGAGGAAGTTCATGTAGGAAACATGAACCTAACACTTCACATCACATGGCAAAGGAATAAAGGTCTCTTGGCAATGCAACTTGAAATTTTGTTTGTTCATTTGGCTTTGGGAGTTGAAAAAGGACGGTTCTAATATTAAAAGATATCAAAAGTTGAGAATTCAACCTTGGGAAAAAAGATTTTGAAGACTTAATGGATTTATGTTTGCGTATTTTATTCTCTATTATATGATGTTTATTTCCATGAACCTCCTCAAAACTATCATGTAATTTTAATTCTTGTTTTTAATCAATACAGTTTGGTTTTATGTTGAAATTGAGCT

>Ca3:39096893-39097111

ATTTTACCGAGGCTTGGGCTCGGACTGGGACAGCTGATCAATTTGTGGTTTTGTCGTTTCATAGTAAACGACAGTGTCGTTTAACATCAACCAAAGCATTTTGAATTGAAACACTAGGAAATGAAGCAAACATAAACCCCATTTTTCGCACAGAAAAGAGAAATCTTTCCAAAAGCAAAAATGTTGAAGCTATCTCGCGCGCTTCGTTCATATTCCAG

>Ca3:39399297-39399520

GTATTGCATTCTGTCATTTGGGGTGCCATTTCTAGGTATCTGCATTCCTTCAGTTTTGGAATCATAACCCACCAAACAGTTTTTCTGCCAAATTGCAAGCAGGAAATGTTTTAGGTTGTTTTACTTATGAATGATGACAATGACTTGTGAATGTCAGGTGGGTTGCTTCTGGATTCGGGTTCTTCATATAGATTCAAAGTTTGAATAGAATTTGTTCTTTGAG

>Ca3:39399294-39399520

GAGGTATTGCATTCTGTCATTTGGGGTGCCATTTCTAGGTATCTGCATTCCTTCAGTTTTGGAATCATAACCCACCAAACAGTTTTTCTGCCAAATTGCAAGCAGGAAATGTTTTAGGTTGTTTTACTTATGAATGATGACAATGACTTGTGAATGTCAGGTGGGTTGCTTCTGGATTCGGGTTCTTCATATAGATTCAAAGTTTGAATAGAATTTGTTCTTTGAG

>Ca3:39573154-39573363

CATGGAAGGGATAAATGATCTGAATTCGTGCAGAATGTCAGAGAAGAAAACAAGAGTAGAAGAGCAATTGAATTGTCCAAGGTGCAAATCAAGCAACACCAAATTCTGTTATTACAACAACTATAGCCTCACACAACCAAGATACTTGTGCAAAACTTGCAAAAGGTATTGGACACAAGGTGGTTCACTCAGAAACATTCCTGTTGGAG

>Ca3:39597504-39598935

TTGTGAAGTGATGGAATTCACGATTTGTTGTTGTTGGTGGTGGTGGGAGTGATGAGATTTTGAGGGTTGAAACAGAGCTTGAGAAAGTGTCCATGGAAAAAGAGAGAAAAGAGAATTATGGAGAAACACAGAAGAAGCTAAGGAGAGGATAAGTTTTGGCCACTGGGTCTTGAAACTTGAAAGCAACACTTTGATTTTATTTTCTTGATGAACGATGACTGGTGAATGGTGATTATTAGGTACAAGGTGATTAGTGACAACTCCAACGTCGCTCCTCTTTTATTCGTTTGTATTTTTACTAAATAAAATTGAATACCATATTTTCATAAGTTTTAAAATTGATATTTCGAAAGTTTTAAAGTTTCAAATTTTCGAAATAAGGACTACATTTCAAAAATTCTCAAATCTAAAAAATTTTGAAAGTTTCTAAATTTGGAAATTTTTCAAATATAGGTAGTTTTAAAATTTTCCTATTTCAAAAGTTTTGAATTGGTTTAAAATTTTGAAAATTCTGAAATTTTCTATTTCTGAAATTTCAAATTGTTTGAAAGTTTTAAAAATTCTAAAAATTGTCATTTCAAAACTTTTGATAATTATGGAAATTGTCATTTCGAAACTTTGGAAATTTTTAAATAATTTGAAAAGAGGGATGAAAAATGAGAAGTTAAAAAAGTTGAAGGTTTTATAAAAGACAAATTTATATTTTCACGTTGATGGAGAGGGTGACAAGTTTAGATGTAGAGATGCGGGTAGAATTCTCAAAAGTTAAATTAGTATAAGACTTGTTTAAAAGTTTGTGAATTTATTCTTTAGTGCTAAGTTTGACTTATAACTCTATTAATTTAATGATGTAGATAATTATGTATCACGTGTTATTCATCTTCATCTCTAACCTTAAACTTAAAATTTATTTTTCCTCAAATTTCTTCTTCGTCTTGTTACCCTATTTATTGTAAGTTAATTTTAATATTCCTGGTGATAATGAGAAACTTATAGCAAATTTGCAAAGAATGCAATGGAGGTTATGGAGTTAAAGCAAATTTGTAGGAAAATGCAAACACAAATGACAAAAATGATAAAATAAAAAAGCATCGGTATCACTTTTGGTCCCTCCCTATTTAACTACACACATGACAGAATGACACATTATAAGTCAAGTAATCGTCATCGTCACTAAGTTAACAATCACATTAAATATTAAAGTAACATACGTTTGGATAATCTATGGAGATATAGACTAATATGAGTTTTTGACTGAGTTATAGACTAAAGTAACAACACTCTCTTCAGTCGTAGATCAAAATAATGGTTTACTTTTTTCAAATCTAAAACATTAGGAAAGAAGTCATTTACTTATCTGAAATTGATCAAAGTTGATATGTCAACATTTCCTTACAGAGAATTATAATAAAAAATAAATTCAATTATGCA

>Ca3:39726183-39727094

AAGCTACCAAACTGTAGGTGACCTCTATCCTTAGAAGAGTAGTGTAAAATGACATCATTCAAAGCTGAGAAACATACTGAGAAATCTAGTAGCTCTCAACCATCAGGAATGATTATGATGAATTTTTTACTTTACAGACATAAGCAGAAGCTTAAGGAGTCAGAGACCTAAAAAACGGGGCACCTTTGGATTCAAAGCATGGACCCAACACCAAAACAGATCTAGCAAAACATTTCTCTTGTACATGTTGCATTTAACTAATATAAAATATTCACACGCAACTTAGGCATAACTGTCAGCAAATAGAAAACAAACTAAATCTGAAATTGGAGTAATATGCTGAAGCAGTACTGAACAGAAACTGATTCGAGATTTGAATAACAGAAGCAGAAAACATAGCATAATGAAGATATTACTAACCTGAAAGATCGATTGACCAAGGTCTCAGCACATACTAGAGGTCCTAAACGCAGATATCTATCAGGCAGGCTTTTGATGTTTTTGCTTTAGTCAATATTTCTCATTACACTTTTAAGATTATGCATCTCAGGTTTGAAATGCATCCCTTTTCCCAAGGTGCATCTCCAATATAAACATGACAATACAACTTCAAAATCAAAAGTATACATACTTAAACCTGTCATAACTGGTAGTTTGGAGAGACCACCAGTGGTTAAAAAATCGATCTTCAATGATTTGAAATATCAACTAAGTCTCAAAAATAATTCAAAAAAATGAAAATGGCTGGTAAGTAATGAGGAACAATGACCGTTTCCACAAGATGGGTCCACGCATAAACTAGAACTACCATGGGTGGTAAATCTGATTTCAGTAGTGGATTATTTTGCTTTTGAAGCTAGTATAATGAAAATAGCATCATAAAAAGTATAAAACTGTGTGTTCCTTTCTTA

>Ca3:39970422-39970686

TGTATTCATATAAAATTGGCATAATATTTTTTGGATGACCTGAAAATTAATTGAGATAGATTTTTTACAGTAAATGTGGCCTTGAATATGAATTTTTTATTCATATGAAAAGAGATTGAGAATTTGAGATAGTATGATTGGATTGTTTGGAATTTAATCACGAGATAATTCTTGACCCAAAATGTGGCTTGCAATATGAATTTTTTATTCATATGAAATGAAATTGAGATAGTATGATTGGAAGACCCGAAACTTAATTACGNN

>Ca3:39970788-39971101

TGGACCCTTGATCCAAAATGTGGCTTGCAATATGAATTTTTTACCCGTATGAAAAGAAATTGAGATAGTATTTTTGGATGACTTGAAATTTAATGAGAAGATAGAATCTTGACCTTAAATGTGACCTTCAATATGATTTTTTTATTAATATGAAATTGAGATAGTATTTTATGAGATAGATTCTTAACCATAAATGTGGTTTTCAATATGAATTTTTTATTCATATGAAATGAAATTGAAAATTTGAGATAGTATGATTGGATGACCCAGAGTTAAATTACGAGATGAATTCTTGATCCAAAATGTGGCTTGC

>Ca3:86375-86616

ATAATTTACAAGCAGCGGAAAAGTTTCTCCAAATATAAATCCAAAGCATTTACACAACAACAAATGGTACATGGAACCCATTAAAATAAGATGTCAAACTGACAATATTAGTATAAGTACAGTTTTCCAAATTAAAATACTCCAACCCAAAAAGAAGGACGTCTAGTCCCTATACATCAACCTAATCTGATCATCCTACCAAAAAACTATACACCCTGAGTGATCTCCACGCGCCCCGTGA

>Ca3:452668-452967

GCCAAAGAAAGAACATTCAACCATATATTATTATTGATATATAAGTAACAAATGTATAGCAACTACAAAATACTCCTATTAAATAAGAACCCAGCAAAAAGTGAGCTTGAACATAGTACATGCAATTCTAAAAAAGCACAATTATTCAGTGCAAAATTTCAACAAACACATTTTCAAAGAAATCCATAAGAGACATCAAGAGGGCCATAACTATCTTATACAAAATGAATATAATCCTATTTCCATGCCACCATCTACCATCAATAACACCTGATGTGTCACCAGTTTGGGTTGTAGGC

>Ca3:500489-500735

GGTAAAATGCGTACTTGAATCACTTAAAGAATTAGAAACTTTCATGGGCCCTTACACAAATAAGAGTACAGTTGTTAAAAAAACAAAGATATTTTGGGATAAATTTCTACAAACTATTTTAAAGAAATCCAAAAGAGGCATCAAGAAGATCATAAATGTCTAATAAACCTATTTTTTCCTTTCTTCTTATCATCCTGCCATCGACCATCTATAATGCATGATGTGCTTGAAATTGGGGTTGTGGTC

>Ca3:1910472-1910900

TGTGTTGTTAACTTATCCCATGAAATTGGTATAGATTGATCATCATTTCAATATTACAACGAATATTATATGTTTTTTCTAAAGGTAAAAGTAAATTAAAAATATATATAAATCTTACCCAGAAAAAAGTCATATATGATATATGGCTACACTCTGGAGAGGAAATCAGAGTAACACATAATTAATTAACAGCATATATGGCTCTGCTACAAAAACCTACCGCAGACCTATCAAGCAATCGCCCAGCATCAACAATTTACTGGTACGTTTACCATCAGCAATTCAGGAGAAAGAACAAGAATTTGGATTCATGCATAGCTTCAAGTCCTCTTGATCTTCGAAAATGTTCCTGTTTCAGAGAAGTAGGTGCTTTGAGTTCCACTACCAAGTTGGTGTGCATGAGCCTGTGGATTTGTCAGCTCAATCCC

>Ca3:1920733-1920957

CATGGAAGAAGACGACAGTAACAGAGGTCCGAAAATGACCGTGACGGTGGCCGGAATGAGAAGTTACGGCGCGCCGCTAGGGTTCCAAATCGTGTGTTCCCAAATCGTGAGAAGTTGTGACTTGCGCACTGTTATGTTACACTATCGTTTAGAGATTGTAAGAATTGAACTCTTTCATTTTATGTTTCATTCTAAAAGCTAACTAGTTTCATTTTATGTTTCAT

>Ca3:1920733-1920989

CATGGAAGAAGACGACAGTAACAGAGGTCCGAAAATGACCGTGACGGTGGCCGGAATGAGAAGTTACGGCGCGCCGCTAGGGTTCCAAATCGTGTGTTCCCAAATCGTGAGAAGTTGTGACTTGCGCACTGTTATGTTACACTATCGTTTAGAGATTGTAAGAATTGAACTCTTTCATTTTATGTTTCATTCTAAAAGCTAACTAGTTTCATTTTATGTTTCATTCTAAATGCTCAGTAGCTATTCAATGTTACTT

>Ca3:1920733-1921024

CATGGAAGAAGACGACAGTAACAGAGGTCCGAAAATGACCGTGACGGTGGCCGGAATGAGAAGTTACGGCGCGCCGCTAGGGTTCCAAATCGTGTGTTCCCAAATCGTGAGAAGTTGTGACTTGCGCACTGTTATGTTACACTATCGTTTAGAGATTGTAAGAATTGAACTCTTTCATTTTATGTTTCATTCTAAAAGCTAACTAGTTTCATTTTATGTTTCATTCTAAATGCTCAGTAGCTATTCAATGTTACTTGTATTTTTATTTTTATTTTTGTATGAAAAGTAAAC

>Ca3:3722863-3723211

GCCGTTATTGCTATTGCAGCGACTTCAAAATTTATAATATTGCAGCTGTAATCGCAGTTTAAAACCATGCTCAATACCTTTGGTTTGTGATTTGTTGATACTACTTATAAATGAAGGTACATGAACAAGTCACAATTCATGAATAAACAAATCACATTAACATGTTGTTATTGACACCTTAAATAAATAAGTCACAAAAACCATGCTAAATACCTCTTTGATTAAAGAATAGTTAATCGTTCTGCCTTAGCTTTCGATCCTCCTTTTTGCACCTATTCGCATGCGCTCACTTGAAGAAAACACATTTATTTTTTTTGTCAAGTCTTTGCATGTTATCTCCATCAACAT

>Ca3:4161015-4161256

CAGAACAACATCTTACTCCATTTCTAACCCAGCACCTTACAAAAATATTTGCAACGGATATAGCAGATTGAAGGTGTGTCCGGTATCCAACATGTGTAAATGTTTGAGCTTGACACGTGTAGTTACATTTAGTTCCATTTCTCAAATTATGATCGGTTTCAACAGTCTTGGTGTTTGTGTCATGTCTAGTGTCAACGTGGAGTGCGTCTATAGTAGTGTTTCATAGTGAACAATTTCAATC

>Ca3:4162164-4162514

GGTTCTTCGATTTTCCGAAATCGAGGGAATTCGCCATGGATGGTGGTTGGTCAACGGAGGAGTTGTAGAGAGAGATTGAGTTATGGAAATAGACCATTTCAATCACAACAACCCCAAATCAAAGAAACCCTAAAATTGGATCGACCCAATTCGATTAAAACTGAAAAGATTATAAATTCAGAATGATCAATAAGATTGAATTGAAGAATTGTTGTTTTTGGGGTTCCCTGATGGATCCAAATTAAAGGATCAATTTTTTGGGGGATTTTGATTTTTTATGTAATTGATTGAAATTGAAATCGAAATTGAAATTGAAATTGAAAATTTAGAGGAGAATTGAAGAAAGAAAG

>Ca3:4207471-4207944

GTTGGTGAAAAAAGAACTTGATAATGCTAAACAAGAGCTGTGCTTTGTCTAACTGACAATTCAAAACAAAAAAATGTGTCAGAGCATTTGGATTGTATGCAGTCGGACTCTGATGCATTCATGTAGTTAGCAGATCAGTGCTTGTAGGATCATGATTGGATGACCCCGATTTGAAGTTTGGTATTTTAGATTTATTTTTTGCACAGTCTGATCTTGATCCAACGGTCAAAATTTTGAATCCATGACCATTATGGAGTATTGGTTGAGTGGAGCGGCAAATGCTTCCTTTTATCACCATTAACATCATTGATATAAATGTCTTTCTCTTTGGCTTAATTCAAATCTTTGCTTCTCTAGCTCATCAATTTCATCATCAAAATTGGGATCAAAGTCAACTAACAAAGGTTGTTGGTTCTTCTGCTCTTCTACTGGTTCGTGTAGTTCTCCTTGACCACTCTGAGAAGCAACCATTG

>Ca3:4207471-4207927

GTTGGTGAAAAAAGAACTTGATAATGCTAAACAAGAGCTGTGCTTTGTCTAACTGACAATTCAAAACAAAAAAATGTGTCAGAGCATTTGGATTGTATGCAGTCGGACTCTGATGCATTCATGTAGTTAGCAGATCAGTGCTTGTAGGATCATGATTGGATGACCCCGATTTGAAGTTTGGTATTTTAGATTTATTTTTTGCACAGTCTGATCTTGATCCAACGGTCAAAATTTTGAATCCATGACCATTATGGAGTATTGGTTGAGTGGAGCGGCAAATGCTTCCTTTTATCACCATTAACATCATTGATATAAATGTCTTTCTCTTTGGCTTAATTCAAATCTTTGCTTCTCTAGCTCATCAATTTCATCATCAAAATTGGGATCAAAGTCAACTAACAAAGGTTGTTGGTTCTTCTGCTCTTCTACTGGTTCGTGTAGTTCTCCTTGACCACT

>Ca3:4438935-4439272

CATCTTTCAAAATCATTTCCAATGAAGCAATCTTCCCATGTTCTGGTTCACTGAGCATAATTTCCCATTGCCAAATCCACAACCATTTACCATCAACAAAGCTTCTCATATTAGTGATCATACTTTACTTTCTTCTAACAAAAATAAGCCAGAATACAATTCCTTCAATGTTCTATCACGAATCCATTTCTCCTACCAAAAAGAAATTTGTAACAGTAAACAAACAAATTATTATTAACATTAAAAGAAAACAAATATGGCCTCGTATATTATTCCCTATATTTATAGCAAGAGTCCACCGAGTGAAGTCCATCCTATTAAACTCATGATATTCCCT

>Ca3:4582481-4582783

GCAGTTTGTATTTTGATGTTCAAAATAACACAACTGATTTGAAATTGCTGCAGATTATAGATTATAGGGGACATGTAGATTCTGAGACCTTTCTTTGGGAAGAACTTTGTACCTCTTCACCTTGTCCAATTAACTCCTCAACATCATGCTCTTCGCAAATTGCTTCAGTTCCTTTTTATGGGGTCTCCAAATGTTGCCCATTTACTTTGGCCAGAGGTCACAGTATCCTTAAAAAGAATCTTCATTTCAGGTAAATTTCTAATCCGCCTGTGTCTTTGAACTTTGCAGCTTTAAGCATCAAA

>Ca3:4582371-4582783

CTTGACATCAAAATTTATATAAGTGTTATTTGATTTTTATGTGTACTTTGAAATGTGTATATATATTGTAGAAGATAAACAGGAAGTGAGAAATAGGGATAAATACATACGCAGTTTGTATTTTGATGTTCAAAATAACACAACTGATTTGAAATTGCTGCAGATTATAGATTATAGGGGACATGTAGATTCTGAGACCTTTCTTTGGGAAGAACTTTGTACCTCTTCACCTTGTCCAATTAACTCCTCAACATCATGCTCTTCGCAAATTGCTTCAGTTCCTTTTTATGGGGTCTCCAAATGTTGCCCATTTACTTTGGCCAGAGGTCACAGTATCCTTAAAAAGAATCTTCATTTCAGGTAAATTTCTAATCCGCCTGTGTCTTTGAACTTTGCAGCTTTAAGCATCAAA

>Ca3:4584720-4585024

CTGGAGTTTGATGTAGGCCTTGAACCAAGACAACAAATTGAGCATCATGTACTAACAAGTAACATTTTCCAATCAAAAAAATAATAATAACAATTCCAAATAAATGTTATGACCTTCCTTCCACTTGCGAATTGTGAGCACATTGCAGATCATTAAGTTAAACTTAACACCAATATCACAGCAGCAAATAGATCAACTTACCTACCTAGCATTGATACTGCCATAGCAGAGATTCCTGTATTGAGTAATTGGAAGATTTGAATTGTTGAACAAGTTCTGAACTCAGCTGCAGACCCTACTTTAT

>Ca3:6252388-6252714

GCACTGTTAAAGGTGCGCTATAATATCTATATTTTGATGTAGTGACGGTACATATATAAAACAATATAATCCCTGATACAGGTCTTCTTTGTTCTTCATGACTGTCAATAACTTAATATATAATCAATCACACACATTGTTGCACTAATAAATAGAACAATCAACTAAGTAACTTAAAACCGCACAAGCATAGTTATTTTTCAAAAACACAACATAGTAACACTGGATTAAATCCAAATTAAATAAAATAGAAACACTGGACAACTTCGTTCCTCTTCATCAATTGTCTTTGTCATACTTCATCAATTATTGGAGTTGAACTTCAC

>Ca3:6282875-6283329

ATCTCCAGCTTCAAAAGTGTTCATCACAGCATGTTAATCAAACTTTTTCTACCTATTTAACAAAGAGTCTGCCCTGGAAATTTATTCATATAGAATCAGCACATGGCAAGGAACAAATAGGATTCATTCAAGCTCAAAAAGGTTATCTGATCTCTAAAATAAAAGGACTCAAAGTCAGACAAAAATCAAGACAGTTTGTAACTCTAAAAATCAAACTGTCAAGAAAGTTTGATCAACCTTGATATATGATAAGACATTGCAAAAGACATCCAGAATGATTAAAATAGGAACTCCAGATTGATTATTAAATCTCAAGAAAGTTCAATTTGACAGACCCAGATTGATAATCAATCTCCGTTTTCTGCATAACTTGAGCAGCTGAGCATTCTTTTATGCAATGGCTTATAATTCTTCTCCAAACTCCTTTAGCTACATTCAAGACTCAAGATCAAGA

>Ca3:6292492-6292692

CTCACTACTTGGAAATGGATGACCATAAGTTGTGATTTGTATTCATAATTTGTTCATTGTTACAACTAGTATACGAAAAACTAAAGTGAGGATTATTACCAGAAGAATACTAAAAGAGAAAATGAGACGTGGTATGAATGAGATCAAATGAGATATTCACTGAATATAAATGTTTGTAAACTAAGCTTTATATAGAGTAA

>Ca3:6286507-6287778

AACCAACTCTTTAAATTAGGAACAATCATGAACACCACATACCCGTCACATATGGTCAATCACAATTCCCGAACTACTCACCAGTATGAATATAACAATTATAGTTTAAGACATCTGAAAGTACTATTGTCCTCAAGGATTTAGTCTCTGTTACTTAATTTTAAATGGTGTTTTAGTATATTATTTTATAGTTGTTTCTATAATTAGTCTCGACCACAATTAATGTTGAATAACAAAAATTTACTACGAGACATTGTGTGAACTTTTCAAATGACATGACCTCTATATGACAACTGATATTACTTTGATAATTTATTTAATTATTTTATATTGATTTAAATAAAAAACAATCAAATAAAATACTAAGTGGATTAAGACCCATTTTCCTATTTCATTCATCCTTTTGAAGCTTTACATTTTTTACACAAATGGTTTAGATTGTTCATAGTTAACACTTAACAGGGTTTAATGACTATATATAATTAGTGTAAATAATTTTACAATAACAATAAACATAAATCATTTATTGAATAATAATTTGATGATGAACAACCAAACGTAAACATAAATTTTTTAATTATATAGTTAGATGATAAAATCAAAGTTTACTAGACACGTGTAAAATAAGGTTACACTTACGAAACATCTTCATTTTTCTCAAATTAAAACGCCATTAGTTTAATCACATGTTATATTATGAATTTATGATAGCTTTGATTTCAAATATTTTTTTTAAGAAATAGTAAAAGCAAAAACAGCAAGTCATTAAATAATAGTAAATACAAGCTCACGCGTTTGAGTTGGCGATATTTGTTTTGGAGGGTCGTTACTCACTCTCTAATCTCATCTCAATCTCGATAGTATACACCTTTCCACTACTGTTAAGTCGTTTTTGTGTAGGTGGCATGCTCTCAACACCTCCACGCCACATCACACTTCCAAAAAACAATATTTTATTTGCCATACTTATCAAATCTCAACTTCCCCACTTCCAAAAAATACTTTATTTTAATATCGAGCTAGGCACAAAGCAAGTACATTTTTACCAAGGACACCTCTCACCTTCACCTTTTCCATACACATAATTTTTATCCTAAATTAGCATTTACAAATCTAAACAAAACTAGCAAACAATAGCATTGCAATCAAACACTACCATTTAACCAAAGTGAAAAATATTTATACATCTAAAATAATACCTTTTCTCCCTCATCCCATACTAATGCAAGTTAAGCACCTTTCCCTTTCAATTGTTGCCGTAAATCAAACCT

>Ca3:6291576-6291858

CACTTTTTAATTTTAAGAGGCATAGTAAAAAAAACTTGAAATTTCTCACAATTGTGTCAGCTAATGATCAACTCTACAACGTTTACTAGCAAACAATCTAATAGATTACGAGCAAACAAACGATCTTTTAGTACCATTGTAAATCACAAAATGAGTAATGAGTTGTTAAAATCTAAAACCCTAACATTAGATTAAACTCTACTAGATAATGAGTTCATTCAAGATTCTAACCTTGGGTGGTGGCGTCTGGTAGATTTTAGCCGCTGTTGCAGATTCACAATG

>Ca3:6283521-6284213

AAGGCCTTTAATGCCTATAAATAGAAGGCCAATATCCAGGATCAAATAAGAACTTCAAGTGCTATCAAACTCTCATAAATCATTCACATTCACATACTTGAAATTCTCATTTTCACAAAAAGCAATCAGTTCTGATCAATTCTATCTCACTGTGATATTGCTACTGATTTCTTTTTTAAGAATCGAAACTCAAACAAGAGTTGAGATATCTTAGATTCTGTCAAAATCAATCTTATTGTAAAAACTCAAACTATAAGAGTTTCTTTATAGTTTGTTTAGATTGCATTGAATCTTATCAAAGTTAGATAGGTGTTGTTGTTGCTTTCTGGGTGAAAAGTAATAGAGATTAAAACATATCAAGGTTGATCTAAGCTAGGTGCTGTAAAACCAAGAAGGTTCTTTGTTTTAAACATAGTGGAAAATCTCACGGTTGTGAGGACTGGACGTAACCCGAGTTGGGTGAACCAGGATATATCGTTGTGTGTATTTCTTCCCTTATCTATTTACTTTTAGTTTGATTGATTATCAAGTTAATTGCATAAACTGAATTATTGATTTTAACTAACCAAGAACGTTCTCCGTTTTCTGTTTTCACAATCAAGATCAATCTTGAGTTATTTTCTAAAGTTTTTAACATGAATTTTTAAAAAAGTGCATTTACAATTCAAACTCCCCTTTGTTGTAAATTGAAA

>Ca3:6285210-6285633

CGATAGTTTAAACCGTCACCAAAAGTACATAGAGGCAGTTTGTAACCGCTGCAATACAATCCTAGAAGAAAAAAACTAGGTGCATTTAAAGACTTTATTGGAGGCGGTTAGAACTCTGGCAAACATTGGCAGCAGTTGGAACGACAATTTTTTTAACCGTCGCAAAACAATCTTGTGATGTATGAATTTGTGACAGTTGGAGAATTATCACAAACATCAAATTAAAACCGCTGCAAAATATTTTTTTCTTGTAGTGAATTCAGAAACTCTATCATACCATATCTTTCTAGTTAAAATTTTTATTCTAAATACAAGCTAAGTTAAAAACTAACATGATGTGATAAGAACAATAATTTACTCAATTACTCTTATTAAATATAATTTAAAATATAATAAAATATAAATATAAAAACAATTTATAAA

>Ca3:6291211-6291526

ATCTACATAAAACTTCAAGCTAATACTAAAAAATCAATCATTTTAAAATCAAGAGCATCCCAATTTCTTGGATACATCAAACAAATGTTCAAATGGGCTTACTTGCTCTACATTGTCCTCTTCATTATCAAGTGAATTATTCAAATTAAAATCATCAACATCATCACAAATTCACAATGAACAATATAAACATCATCATAATCATTTTCAATGGACACGTTATCAAGAAATTTGAACATCAACATTATGATTTTCAACTAAAATTAGTGGTTCTTTTATAAAAACTTTATCAAGAGCTGATGTTTGAGATTGAGT

>Ca3:6975812-6976119

TACCCAAGCAAAACAGGTTGTAGTGAAAATGACATAACACCATATGTGTTCAAGCAATTGCAACACCAAGATACATAACAACAAGGGCTCGACAAATCATGCAAAGTACACAAATACTTAGTCAAACACACACAGAAGAACAAGATATGTCATTACATAATCCTTAATTTAATATAAAACTTATTCAAAACAGAATACACAAATACATACACACAGAGAAGAAAGAGATAACAATTACATTTTACATTTAACCAACTCGAACTTGGTGAGTTCTAATCCATAATCACTTTCAACTCCACTAAATATT

>Ca3:6976211-6976482

CATGTCAACATTTTCTATATTCTCATGTTCTTCATCATGATGACCATACTCATTATCAAGGGAGTTTGACTCAACCTGAGTATCATTGAAATAAGGGTTTTGACTTTGACGAGTACTAGATTTTCTATTATGTCATTCACCCCCTATATAGGCCACAAGCTTGTGATTTCCTTCCATATGGCTTATTTTTTGTTTTTGTAGATGATCGAACCGCATCACATCCTTTTGTTCTAACACGAGTTGGGTTTAATATCGCTTTATCAAAAAAGGT

>Ca3:6979155-6979400

CTTTCTTCTTCGATCTTTGTTCCTGCGACTCAATCTCTCCATTTTCGATTCAACACGTTTGAAATGGGAAAATAAAGGAAATTTTCAAAAATGAATTTTATAGTTGAACCCTTCACTTGCAAATTCTCTTCAATGAATATCAGAGCTAACTCAAGATTATCTTTTCCTGAAATTCTTTGTAGAAAAAATCTAAGATTGAAAAAAAGCCACTTTTTTGCTATTCATCCTACTCGATTTTGATAACG

>Ca3:7048782-7049131

CTGGATCATTGTCGCCAACCTTTCTCCATCGCGGAGGAATAACGAGATGAGAAAATTGACGAGTGAAGAGAAGGTAGACGAGAGAGAAGATTGAGGGTCGAGAGATGGACGAGAGAGAAGATTGAGGGTCGAGAGATGGACGAGAGAGAAGCCTTAGGGACGAGCGAGCGACGAGAGAGAAGCCAGGGAGATGAGGGAGGGATGAGAGAGAAGCTTGAGGGGCGGGAGAGGGACGAGAGGCAAAAGTTTAAGGAGCGTGAGAGGGACGAGAGGCAAAAGTTTAAGGAGCGTGAGAGGGACGAGAGAGAGATTACTTAAAGGACGTCAGAGGGGCAAGGGCTGAGGGGCA

>Ca3:7048439-7049132

ATATAACGACTCACTAAAACAACTCATTTTTATAGACAACAAGAGTACTATATTTTGTTTTGAACTTACATAGTGACACCAACAAACCAGAAGCATAAGCAAAAATACTACACCTGAGATTCAGCACCAGATGTATAAACTCATGTACTTGAATCAAAAGTAAAGAACACTAAACTGAAGAAGAAAAAACCCTAAATTGAATAACCAAAAAACCCTAAATGAAGAAGCAAAAAATCAAACCCTAAATTGAAACAAACAACAATCACACAAAGATGCAACAACAATGATTAAGAGAAGTAAATTTACAACGGGAAGACTCAACAACATGTATCAATCGCACAACCTGGATCATTGTCGCCAACCTTTCTCCATCGCGGAGGAATAACGAGATGAGAAAATTGACGAGTGAAGAGAAGGTAGACGAGAGAGAAGATTGAGGGTCGAGAGATGGACGAGAGAGAAGATTGAGGGTCGAGAGATGGACGAGAGAGAAGCCTTAGGGACGAGCGAGCGACGAGAGAGAAGCCAGGGAGATGAGGGAGGGATGAGAGAGAAGCTTGAGGGGCGGGAGAGGGACGAGAGGCAAAAGTTTAAGGAGCGTGAGAGGGACGAGAGGCAAAAGTTTAAGGAGCGTGAGAGGGACGAGAGAGAGATTACTTAAAGGACGTCAGAGGGGCAAGGGCTGAGGGGCAA

>Ca3:8131758-8132216

ACTTCACCGAAACACACAAATGAGTAGTGCTTCTTGAAGGTTTCGACGAGAGGAATCCAAAAATGATGTCAGTTTTTCAAGATGATGAACGTGTTGGCCGGATTTTGGCAACACAGCCGCGGCGAGGGTGAAAACCCATTTTTTTAAGTACAACGATTGGCAATTTCAAGCATAGGTAATGGGAGAGGGTGTTTTGTGGAGTCCAAATATGGTCTCAGTTTTCATTGGGGATAGTCGTGGTGGCCGGAATCTTGATCAAAAGGTGGAGGTGTGATTTTTGACAGAGAGGAATATGGCGTTTAAGTTTGCGATTCAAGTTGGAAATGAAAGAGAAGATCACAACGATCACAAATGTGGTCTCGGTTTTACACGAAACTTGGTATAATGGTTATAATTGAAGAGAGAAGGTCAAGAAGATATAAGAATGGAGGCGGAGGCTAGTCTACGAAGAAGATGAG

>Ca3:8529477-8530719

GTGGTTTGAATTGGAATTGTTGTGGTAATTGTGTTGGAATTGCTAAGTGTTATGTATTGATTATCGTGTGTGAGTGCCATGGATTGTAAAACTGTTTCGAAAATCAATTCGTCGTGGTGATTGTGTGGGCTTTGCTAAGCATTAAGTTTTGGAGTTATGTGTGAGTGTGATTGAATTAGTTTAAGTATTTTTGGAAGAATAAGCAGGGAAGTCGATTTTCCAATCGATTGGATGAGTTGCAGGAAGTTCACTATTTTAACCTAATCGATTTGCCAATCGATAGTATAAATATGTCTTTCAAAATCTTTTAAGAAATCGATTTGGAAATTGATTGGCAGAGAGGCACGAACTCAGCAAAACTGCCAAAATTGATTTGGAAATCGATTTGGCAACTCAGGGGCTCATCAAAACTGACAAAATCGACTTAGAAATCGATTTGGTCATGCAGAAACTCAGCAGAACTGACCAAAACGATTTGGCAATCGATTGGCTCAGCAAAATTACTTATTTTGAGTTAGATAATCGATTGCTCAATTGATTTATCAATGCTTTGATCAGTTATGTATTACTTGATGGTTTGAGAACTTCATTTTGAGTTCATTGTTAATTGGCAAGCATTATATGAACTTTTAGCATATGGAAAATCCTTTTAAGGTGCATGCTTAGTTGAAAGGTTATTTTGTGCATTTAAAACATAAATGTTATGTGTTATCTGTTAATTTCGGTTGGTGATCCTTTACAATTATTGTGGAAATATGGGCTTTGCCCTCAGATGAGAGTCAGGACGATCCTACCGGTTCGTACCCTACGGAAGGGAATGGAGATGGGAACGCTTGACTGCAGCTACGTTAGGAGGATCCCATGGGGCGCGTGGAGATCACTCAGGGTGTATAGTTTTTGGTAGGATGATCAAATTAGGTTGATGTATAGGGACTAGACGTCCTACTTTTTGGGTTGGAGTATTTTGATTTGGAAAACTGTACTTATACTAATATTGTTAGTTTGACATCTTATTTGAATGGGTCCCATGTACCATTTGTTGTTGTGTAAATGCTTTGGATTTATATTTGGAGAAACTTTTCCGCTGCTTGTAAATTATAATGACTCAATTATTAATCCAAAGGCATTTTCTTGTTTATTTCTCTGTCTCAGTTTAATTTCTTTTTAAAAAACAATTCTAAGAATCTTAGATACAATTTGACTTTGATACATTAGTCTGGCTTTGCATATTGTATAAGTGAA

>Ca3:8672819-8673302

CAATGATCATTCTATTCAAACTAGTATATATACAAAGTTATCTAACCAAACCTAATCAAATTCTACGATCTAGAAGCATTTCACTTGTTCCTCAAACCTTTTTCCTCTCATGTTTCCAACATTTTCAAAGTGGCAAAAAAAATGTCAAGTGTTTATTTATGCATAGAATGAGAAACATATAAAATTAGTACAGGCATATTAACTCTTCCCTATTGAAACTAACTTGGCATTACTAAGGTTAAGTGTTGATAGAAGCCTCTTTTTGAATTATACAAAATTAGTGAACAGAACTTTTAGCTACCCAAAATTTCCCATTCTTGAGTAATCACAGTAGAAGATTGAATTGCTATTGGAGACTTCAATCTTCCAAGTTCTGATTCAATTGTGTCATTATTAGTTTGGTACTGAAACTGCATTGTTAGCAGCAGAATTTCTTGCCTTCATATTGTCAATTGTGTCTACATAACTTTGTACACGTTGAAC

>Ca3:8779335-8779785

TCCATAATAAGGAAACGACGAGAAGAGAACGGAGGGATTTATTTATTGCTAAACAAAAAAAGAAAAATAAAGAAGAAACAACACAACAAAAAACAAAGAAAATGATGTAGAGAGATCTAAAAATTCACCATCTACCTTCATCAAATAAGTTATGACCATAAACAAACACAAATCTATTTTAACAACATAACTAACAAAAATCAAATAAAAAAAACTGAAAACGGGAGAAGAGAATCACAACTAATTTCTTCTTATTTGATGTCTGCAGTACGCTGTTCATTACAGCCTCCATCTTCACTGCAACCTTTGTTATACCGGGAAAGGCATGTACTTCTTCCCTCTCTTCTTTTTTGCTTTCCTTTTCAAACTTTATCTTATAAGATGTAAATTTTATTTTTCTAGATGAAAACTCTTTTTTTTTAGAGCGACTAATTTTCGCATGTAACTCTC

>Ca3:9143576-9143788

CTGAATTTAGTGTCATATATTCGGTCATTTAGCAATAGTATGTGAGGTTTTACTTGCGCTCCAGCAACCAAGAACTAGATTGGTGGCGAAGAAATTGGAGCGTGAGAAACAAGCCACCAGAACTAGATTGAGAAACAATATAGGTGAAATAAAGTCATAGCGTTGATCGTATTCAGTATGCTTTCCACTTGAGGAATCTTTCCATTTTGTTG

>Ca3:10598828-10599058

TGTCTCATAAATTTACAATTTCAAATCAAATTACAAATTAAAAATAAAATGAAATTATATTACAATAAAATTTAAACCCAATCAGTTTAAACCAGCCTGCTTTGGCTTTGTCTTGTTGCTGTCTTTGTTGCTGCCTTTCTGTCTCTGATGCAATATCATGGTTGAGGCACTATCCTTTGCTTCGTAATTAATTAGAGAAACAGAAAAGCATAATTTTTACTAGTCAGTCT

>Ca3:11930029-11930324

CAAAATGGACCTTAAAATTTGAACAGAATCACTAAGCCAATGATTATCTAACCAGAGCTTTCCGACATTACCTACGATGTCTATCAAACATCTAACACACATATCATTCGGGTTCTCCATTGGAAACTGCTGATTCAAGCACCGCACACAGAACTTTGTTCAGCGACCATAGCTTCATACTCTTGGCCAAATGCCAAATATTCCATTCATGTTGTGAAGGAAGATTGTTGAGAACGTTTATGTGAAGGAAGATTGTTGAGAACGTTTATGTGAGGAAGATGAAATGATAGGTTCA

>Ca3:12143761-12144558

ATCTCTCTATAAAATCAGCCAATAACTGTAAGAAAACTAACACAATCTCCTGGAATTAGAGAGACTAGTCTCTCTCAACCAAACCAACAAAAAATTACAGAATGATAATTTGTCTTCCCATCGAGCCTTATTCTATTTAAAGATACAAACACAGGAAATATCCACAGAGAATAGAAGGTTAAATTGTTTGTTTGGATTAACATACATTGAACTCAAAATTCAAAATGACTTACAAGCACAAAACTTTTGAAACATGAACCTAGAAGGAATTCCATTTTGAACCAACTAGACCGCGAAAAGAACTGGATGCCACTTCTGCAATATTGTTTGACAAGGCCATATTTCTTATAATCCAAATCAATACAATACATCGTCTCCCATCCTTTTCTCTAGAATTTTCTTCCAAATCAAAGTTCATTCAACTCTAATGATATCCTGAAATTGTTTTTTTCTCGAAAAATAAAGAAACCCATAAATTTAACGGATAAGATAGTATGTAGTAGGTAGCACTGACACTTCAGATTAATAACGTGTCTGGTACCGACACAACATCGACATATATTGTTCCGTTCAATCACTTTTTCATTTTCTCAAAATATAATTGATGTCAAAGTGCCAGTGTCGGTGCTTCATAAGTATCCCTCATTCTTTGACAATAGACTTACTATTTGAATGTGTAGAGAAGGACTTTTTGAATGTACTGCGTAATTTACCTCCTCTGTTAGTTCATATTATCTCACAACATACTCAGACAATACTCACTGGTCCTGACAATTTAGTATCCCAAACTTGGTGCC

>Ca3:12145127-12145470

GGATAAAATAAATATCAAACTAAAGAAGAACCAAGTTATTTAACTTGTAGAAATTTCACATTTATATAATAAAATAATAGGTATTACATCCATATTCATTTTAAATTACTAAATAAGTACATATGCACGTAAGAATATAAGATTACAATGCTACCACCATCTAACCAATAGAATTTTCCCCATTTTGGTGTATGCCATCAAGAATATGAAATATATAAATAATATGAAGAATTGAATTTCACGAGGTACTTATTACTGACAGTATAAAGTCCACAATTCACTTAACTTGAGAAGTTACTCTCTACTCTATCTTCACAGAGGCAGACGCACCATTGTCAATCTA

>Ca3:14235647-14235914

TTGCTACTGCAAATCGCAAAGTGATACGAGAGAGATGAATTTAGGATTGTTCGAAATTGAATTTAGGGGTTTTGGGGATTATGGTTAGTTGAAGATAGTAGAGAGAAAAGGGTTTGAGAAATACAAAGAAAAAAGAAAAGATTTCTCAAGTGTTTGGTGTAGTTGCATTTTCATGGTACACCCTGAGATAAGTTCCCCACTGAGATTGAGCACCAGCGATGATGAGTGATCTTACCAGCTGCAATAGGGATTCAGCCTGAAGGAATT

>Ca3:15098642-15098908

CATAGAAAGGAACAAATCTGTTTGGGAGAAACAAGAAATCGACACCTCTCGATAGCAACACTTCTCGGCAGATGGAAAGGGAAGACAAAGAAACTCGTTGCCGGTGCGGAAATCGAAAAACGTAGATTAGTCGTCGCTGGCGCTAAAGAGTTCGTGCGTGAGGGAGGGAGTGAAAGCAAAAATTAGGGCATACGAAGGTGAAGGTAATTCAGGAAGTTGGTTCTCGAAGGTGAAGTGATTCAGGAAGTTGGTTTGTGTAGATGAAG

>Ca3:15098642-15098938

CATAGAAAGGAACAAATCTGTTTGGGAGAAACAAGAAATCGACACCTCTCGATAGCAACACTTCTCGGCAGATGGAAAGGGAAGACAAAGAAACTCGTTGCCGGTGCGGAAATCGAAAAACGTAGATTAGTCGTCGCTGGCGCTAAAGAGTTCGTGCGTGAGGGAGGGAGTGAAAGCAAAAATTAGGGCATACGAAGGTGAAGGTAATTCAGGAAGTTGGTTCTCGAAGGTGAAGTGATTCAGGAAGTTGGTTTGTGTAGATGAAGGTGAAAGCCAAAGAGTTCGCGCGTTTTTTT

>Ca3:15098642-15098939

CATAGAAAGGAACAAATCTGTTTGGGAGAAACAAGAAATCGACACCTCTCGATAGCAACACTTCTCGGCAGATGGAAAGGGAAGACAAAGAAACTCGTTGCCGGTGCGGAAATCGAAAAACGTAGATTAGTCGTCGCTGGCGCTAAAGAGTTCGTGCGTGAGGGAGGGAGTGAAAGCAAAAATTAGGGCATACGAAGGTGAAGGTAATTCAGGAAGTTGGTTCTCGAAGGTGAAGTGATTCAGGAAGTTGGTTTGTGTAGATGAAGGTGAAAGCCAAAGAGTTCGCGCGTTTTTTTT

>Ca3:15093313-15093845

GTATTAATCAAAGATAGGATCCAAGCAAAGCAAAGAATAAATCAACATGAAAAGCAAAAATTTGAACAAACAAGAACGAAAAACATTCTTGACAAAATCTGAAAAACGGAGAATGTTCTCCAGGTCTAAGAATTATCAAGTCAGCTTCAAGAACAATCAATCTCCACTGATTAAAAGAAGATATTGCTTCTGAAATTAACACTAATATTATCAGTACTATCCAAGGAACAAACATAATCCATTCCAGTCAAATAAACGATCCGAATCACAAAAGAAAAAAGATCATGAATCAAGCAAGATTAAACAAGACTGATCTTTCTCCAGATTGACTGCCAAGAACGTTGAACGATCTTGAGATATGTAAGACATTAGAGCACCCTCAAGAACGATAAGGCAAACGTCCAGAATGATCAAGATTAAATCTCCAGATCAATTATCAATCTCCAGAATGATCGTTGAATCTCCATAATGATCAAACATTCTCCAGAATGTTCAACAATCGTTCTGCAACAGATTACCATCATTCTCGAGC

>Ca3:15098642-15098940

CATAGAAAGGAACAAATCTGTTTGGGAGAAACAAGAAATCGACACCTCTCGATAGCAACACTTCTCGGCAGATGGAAAGGGAAGACAAAGAAACTCGTTGCCGGTGCGGAAATCGAAAAACGTAGATTAGTCGTCGCTGGCGCTAAAGAGTTCGTGCGTGAGGGAGGGAGTGAAAGCAAAAATTAGGGCATACGAAGGTGAAGGTAATTCAGGAAGTTGGTTCTCGAAGGTGAAGTGATTCAGGAAGTTGGTTTGTGTAGATGAAGGTGAAAGCCAAAGAGTTCGCGCGTTTTTTTTT

>Ca3:15095248-15095664

GTCATTCAAAAATTGATTTATTACATCAGTTAAATTTAAACCAATCTAACAAGTCATATTCAAAATTTAACTAAATTTCATATTTCCTTTGTTATAGCAATTAATAAATCAACTAAATATCAATTGCATCGTAGCATTGTCACAACAAAGATACATAACACTAATATAATTATAGACAAAATCGAATTGAACATCCTACTTGAGGGATCAATGCATGCTCATTGTTGAAAGAATTCACAGGTGGATAACGTAAGGGATCAACGAGTCTAACGTTTATATCATAAAGAACCAAATGAAACTTAAGGGACCAAAAGTGAAATTTAGCCTTTAGTAAAATGAAATTTATACCTGAGATAAGATGCCATTGCAGCCATCTTGGTGCAAGCCACAAATAACACTTGAAGCCTTTGGTAAAT

>Ca3:15094383-15095031

GTCCTATCTAGAGACTAATAGAAGATTGTTTCGAAAGCCTTTCTGGGTGGAAAAGACTTGTTGTGTAACAGATCAAGGTTGATCTGGAGAAGTGGTGTAAACCAAGATTGTTCTTGGTTTTGAACACTTAGTGAAAATCTCACAGTTGTGAGGACTGGACGTAGCCCGAGTTGAGTGAACCAGAATATATCATTGTGTGGTATCTCTTCTTTTAGCTCTATTTACATTAAGTTTGATTGATTATCAAGTTAAATATATTGTTGATGCCATCAGTTTAATATGCAATTTTGATTCTTGCAAAACAGGCTGAAGAATGATAGTAATCTGTTGCGATTGCTGAACATTCTAGAGAATGTTTGATCATTTTGGAGATTGATAATTGATCTGGAGATTTAGTCTTGATCATTCTAGATGTTTTCCTGATCGTTCTTGAGGGTGTTCTGATGTCTTACATATCTCAAGACCGTTCAACGTTCTTGGTAGTCAATCTGGAGATAGATCAGTCTTGTTTGATCTTGCCTGATTCATGATCTTTTTTCTTTTGTGATTCGGATCGTTTATTTGACTGGAATGGATTATGTTTGTTCCTTGGATAGTACTGGTAATATTAGTGTTAATTTCAGAAGCAATATCACTAGTGCAATTAAT

>Ca3:16227471-16227791

CGAGAATAATTGTTTCATTATACAAAATGGTTTCGACACAACAGACTTACTTACAAGACAATTCCCGTTGACTAAAAAAATTACAAATGTTTCAAATACATAAACTTCATTGCGACAAAAACATTGCAATACGAAAGGGAGGATAAATAAAATTCTTAAATAAAATCATAAGATGATCATTAATCTAACTAAGGGTGATTGGGTACTGAGAATCCACGAGTGGCATCAAGCTGCTTCCGATTACCAATCTAAAATCATCGCTATGTCACGTCTACGACATCGTTGCAATTCCACCACAACTCAAAACTCTAATAACCTAG

>Ca3:16950638-16950918

CTGGAAAAAAACTGTTGGTGCAGAAGGTGCACCTCGACGGCGACCTGAGACGGCGGGCGGCTAGGGAGATTTCGAAAATGAGATACTGCTACATCAGACAACGGAGATCGGAAGTGTAACCGAAAACCTAAGTTTATTTTTTAGATAAAAAAAAGAGGAAATTACTAAATTATTCAATAAAATTATAGAAATCGGTCAATTACTCATCTTAATTTTCAAAATAGAAAAATAATATTGTGAAATTTAAAAACAATATAGTCTTTTATAATTTCATCAGTTA

>Ca3:16966226-16966512

CTACCGACTAGCTACCGGCTATCAGGCGATAACAACTACCGACTATCTGACCATAGCTGCTACCGGCTATCAAGCCATATCTGCTATAGGCTATCAGGCAATACCAGCTATAAAGTGATATGAGCTATCAGGCGATACAGACTATAAGACGATAGGCGATACCAGCTATCAGGCAATAACAACTACTGGCTATCGGGCGATATCATCTACCGAATATTAGCCGGTAGCAGCTACCAGTTATCAGTAGCTATCAAACGATAGTTGATAGCTGATAGCTCCTATCGTG

>Ca3:17316609-17317085

GGGAGCAAGATCTAAAGATAAACGGAAAGATAAAGTATAAAGTATATATCAAACAACTCAAACACATGAATTTGAACTCTTAAATTTTTCATTGCAATAAGCCAAAAATAAATAAATAAATAAATAAATAAATAAATAAATAAAGCACATAAATAGATGAATTTAAATGATTATTCCACCAACACAATAACCAATTTCGCCAGCATCTATATATTATATATTATAATATAATTAAGAAAGAAATAGAGATCCGTACATACAAACATGTAGAAAAATAGTGTTTCTCGCTATACATTTCCACCCCCAAAAAGAAGAAAAAAGAAAATTAATATGATGATGACGATAATTAAGAAGAGTAACAAAGTTCTCTTAATGAAAACAAGCAATATTAATTAATGATTGAAGATCTAAAATAAGAAATATGAAGATGACCATATATATAAACCGTTACATCTCCTTTTCATCTTCAATATTAG

>Ca3:17647087-17647359

ACACGATATAAGTTTCACATACTATGCAACAACCTGCCAAATCACCCAGAAGTTTAGTGTTCCCAAGGCTAAATGCTGTTTACATAAAAAGCAAGCTTTAAGAAAAACAATCATTCCAGAAATTCTTTGTTTCATTGAAACTTGTGTAAGAGGATTTGGCCATTATTTATTCTGAACTTTGCTGTTTCAGCACACTCTAAGGGTCCAAAGCTGAAACCATGTTTGCCATATACAACTTGATGACCATGTTCAGCATGCTTGCTCAAACAATT

>Ca3:17649783-17650002

CAAGACAATAAACTCTAGCTATATTCCCTACCAGAGGATTCTGAATCTTGGAATGTAATTATAGTAACTTTACTTGACTTCCCAAAAGAATCACAGGGCAATCACTCATGCAATGCTCCAAAGTACCTTATAAATAATGAATCAATGTACACTACAATCTTGGAAATGGATTTTTATTGAACACTTCTGTACAACAGAATCACCAACAAGCAACAAAGT

>Ca3:17651445-17651647

AAAGGGGCTTGAATTTTATTTATTTTTTAATATATATTTTTTGCTTCTTCTGGGTTGGTATCTCCAAAAGATCCCAAGGATGTAGAGAAAGAAACAAGACAAAGACACGGAAGAAAATTGGAAATGCAAAATAACCGGATTTTATTTTATTTATATAATTAAAGTAGAAAATCATTTATATAAACAGATGATTGGTTTTTTT

>Ca3:17651445-17651662

AAAGGGGCTTGAATTTTATTTATTTTTTAATATATATTTTTTGCTTCTTCTGGGTTGGTATCTCCAAAAGATCCCAAGGATGTAGAGAAAGAAACAAGACAAAGACACGGAAGAAAATTGGAAATGCAAAATAACCGGATTTTATTTTATTTATATAATTAAAGTAGAAAATCATTTATATAAACAGATGATTGGTTTTTTTTAAGGTAAGATGAAA

>Ca3:18356226-18356444

CTGAGCGTGGTGGTTGGTGTTCAGAATCGCCGGAGAAGAAGATCGGAGTAGGATCGGCGATAAGCTTCGCGATCGTAGTAAGAAGAGAAGCAGCACTGCAGTCGGGTTTGGTGACCCGATTCTTTGAATAAGCTAAAGAATATAGCGCCGTCTATGGAAAACGTAAATGCTATTTTTTTATTTTTTTTAATTAAAACCCTATGCTACTAAAAACGCCG

>Ca3:18528719-18529123

ACCGCCACAATTCACCACTATGTATTTTATTAATTGAATCGTCAAATTCGCAACAACAAAAGAATTGTCATAATATTTAACTCTAAAATGCAACTATATTTGACAAAACTATTAAACCTATTATTGTGAAAAAAATTAAAAACTAAAGGAAAAATACATTAAGAGCATAACTACTTAATAAATAAATTTGATACAAATATCTTTAGCTATTCCCAAAAATTAAACAAGTCTCAACCTAAAAATGACAAAACCATTCACATGTCATTATCTTAAGCTATTCTTGAAATAAACCAATTTCAAAACATAAAAATAACATAATCAATTCAAGTGTCATTAGAAAGACTTTGATTTCCTCCATGTACATTACTTGCATCCAATTATCTTATTAGGTCCACTTCCTACAT

>Ca3:18531330-18531645

CTGATTATTGGACTAGAGGCAATAGAAATCAGAGGCGCGATTGCTGGACCAAAGGCACAATTATTAGAACAAAAGGCAAGAGCAATTAGAGCATGATTTATTGATCAGATGCAGGAACACCAGAGACATGATTTTTTGACCACATGCAATGACCAAAAGCAATATTAATTGACCAGAGGCAGAATTACTATACCAGAAGCAATAACCAAAGCCAGGTTTACTAGACCAGAGGCAACAACAAGAAACAAAGGTAAATGACCAAAGGCAGACTATCAGCGACACTGACTATGTTAGAGTTAAATTTAACCAGAGGCG

>Ca3:19095315-19095521

TATGTATCATAAATTTACAATTTCAAATCTAATTAGAATGTTAAAACCAAATCAAATTACAAACTAAGAATAAAGGCAAAAATAAATTACAATAAAAATTAAATCAAATCAAGCCATGCTATGTTGACTTTGTTCACTGCCAAACCCTGCTATGTTGATGATGACTGCCATTGGTGCACAAGCCATGCTTATTGATACATCTGTTA

>Ca3:19402958-19403367

GGTTGCTGTAATGCAGAAAGTGACATCTTTACCGAGAGTTGAAGTAATTCCCCCAGCCCCATTTTATCTTTTTAGTACCGATGGTCTTGTAGTGCACCCTTCATCGAAAGAGTAGGCGGTTGAAAGACCCACCTCTGAATAAACCTTTAGGTTGGGAGATATCAAAGGGGAACCTCGCCTAGCTTATCTGTCCTTGTATTATTCAACTCATCTGTCCACTTATCTTGTCCAAAGCAGAAGGAATATAAAAATCCTTTGTTTCGAGGGTAATCCGCCAGTCTTTGAGGTCGCCTTCCTTGCTTGACAGTATAGCCTGCGGGTGAAATCCCCGATGTCGTATTGTACATGGGAATTAAAATTGTGAGGCCATTGAATGAGAAATCACGACCTTAAACACTTGCCTGTAGCT

>Ca3:19815636-19816226

AGACACTTTAGAGGCATAAAGGAGAACACAATTTTCATGTTACATAAATCAAAATTTTGTCAAAATATTTATGAGTCATTTCTTCTTTTCTTTAGTGCACGAGAGTAAATAAAAGAACTTTATTCTGTAAGAACTTTATTCTGTAAACTACCAATGATCGATAGACTTGATGTGAATGTGCAATTCACTTTAAAAATTTCAAAGTATCTTGAAGGGAATTCGTCGGTTAACCCGTTTACATCCAATATATATAGATTGGTGGAGGCGAATTGAACCTAAAACTTAGTGATACACACGCTTTCGAATTTATTCATGCAACCTTCATCATCATCAATTTCATTATCTTCTTGTTCAAAATATGCAGCATACCCCCGACAAAAGATCCAACGGACACTTGCATCTGCATGACAATTATTTACCTCAAATGCTACATACACAAAAGGCATAGTCGACCCAATCAACATGAGTATCAAGGTAGTCTTTTACCCTTATTACTAAACCCCATTAAATCGATCGTCAAACCACTCTGGAACATATTGCTCAGAACAAGGAAGAACAATGTCAAAGCCACAACAGAAGTGACGAGGTTC

>Ca3:19815636-19816036

AGACACTTTAGAGGCATAAAGGAGAACACAATTTTCATGTTACATAAATCAAAATTTTGTCAAAATATTTATGAGTCATTTCTTCTTTTCTTTAGTGCACGAGAGTAAATAAAAGAACTTTATTCTGTAAGAACTTTATTCTGTAAACTACCAATGATCGATAGACTTGATGTGAATGTGCAATTCACTTTAAAAATTTCAAAGTATCTTGAAGGGAATTCGTCGGTTAACCCGTTTACATCCAATATATATAGATTGGTGGAGGCGAATTGAACCTAAAACTTAGTGATACACACGCTTTCGAATTTATTCATGCAACCTTCATCATCATCAATTTCATTATCTTCTTGTTCAAAATATGCAGCATACCCCCGACAAAAGATCCAACGGACACTTGCAT

>Ca3:20808119-20808473

CAGAACTAAGCTATTGAGAAAGAAGGAAGGTGCCGGATTGCCGGAAAACACTGCCAGAAGTGGAAGGTCGGTTTGCCGGAAAACGCTGCCGGAAGGTGGATGGTCGGCTTGCCGGAAAAAACTGCCGGAAGGTGGAAGGTCGGTTTACCGAAAAATACTGCCGGAAAGTGGAAGGTCGGTGGTCTTGGGTGACGGCAAAATCAGAATGGTTTTGGAGTTTTGAGGTTTTGGAGCTTTGGGGATTTAGGGTTTTGGAATTGAAACTTGAGAGTTGGGTGGATAAAATCTAAATATTTAAAATGGATTTAGATCCGGTTTTAGATTTGACTCATAACCAGATTTTTTAACACATTC

>Ca3:21420365-21421526

GTTGTATGTACCTACTTATAACTTATAAGAGCTCACTTGTATTCAGTCACTAAATTCAAGACTTGCATCTAATCAGCTGAGTTGTTAGAAGATAGAAGGCTAGAGTTATCCTTCTCTTTCTTCACCTGGTCTCATCTAATTCTACTTCTATAATATTGCCAACAATACCTTACAGCATCTTCTGCAAATATCCAAATACCAATTACTTAACAGTTGATTAATTTTATGCAAAAAAAAATCATAAAAGGGTGCTAATACTATAATATGTCCTCTTCATAATCAAGGCTTTAAAAATAGAAACTATATATTCAAAAACATCCAATGTTGTGTTTTCTACTAGTGGCTATATATTCAAAAATATAACAAGTAGACTAAAGACACCTCCATCCACCAATTCTTAATAACCCCACCAACACAGATCAAAATTTTGTTATAACTGTCATGATAATAGACTGACTAAACCAGCAAGAAAATGAGCTCGCCACTTGCTGGTACCTGATCAATCACATACGGTACAAAACAACTTTTCACAGGGTATATAGCATAATAGCAATGCTATATCTTCTTTTTTAGATAGCATTTGGTAATGGATATAGCAATGCTATATTCTACTTGTTTCCCACACAATAGTGCTTTCTTATCAAACACTTGATCAATTTTAACAACGGCTTAGGCTAACATGTAGAAGAGACAGCTACGTGAGACTGTGCAGCTATATGGAGCTGATGTTGTAATTAATATAAAAGGTTAATGGGTCGGTGTCACAGGTCATGACTGCAGCGGTGGAGCCAGCTTTAGATACAGAGGATAAGGCTAGCTGCTGATTTTAGTTCTGAGTTAGCAATGAATCCATACCTGGTGCTGTGTTTTCTGTCGTGTGTTGCTCAGGTGGTTAGGCCAAATCTGGTCTCAGATTGTATGGCCTGGAAAACTTCGCTGCATTGTGATGTCTTAAGCAACTGGAAGTTAATAATGTGATTGTAATTTGTAGGGAATAGTTATGGGGTGTTTTTGTTTGTGGTTGGAGGGGTGTTATTAAGGTGTTTGGCTGCTGCTAAGGATGCTGGTATGCGACATTCATGCTAATAATACATTTAAACCCTTAACAGCTAGAAATTATCAAATTCAGAAATTGGGTTCAGAATATGCAGCCAATTACTA

>Ca3:21424524-21425006

TTGGTACGTAGTTGGGTCTTGGCACATAAAATGTTTTGTTCAATTAAGGGTCTTTTTTCTCGAGCAGATAATTGAACATTCTTGGCTAATCAAGAATGAGAAAATATGTATATCCCAACAGGTGTGAATTCAAATACTTTGCAAAGTAAATTCCAAATTTACCATAATTAACGAAAAAGAAGTTTTTCAAAAATTGATGCAAAACAGAGTTCGAACATTGCGGGACAAAAACGTTCAAGAAGGCTAGGGTTATGGGTCGTGTACTCCACAGCCTTTTTGAAAAAAGATTTCATCTCGTTTATTTTTCTTAAGCAGAAGATTGAATGTTCTTGAACAATCAAGAATGAGAAAATGGATATTCCAATTATAAACCAGACGGGACAGAATTTGTTGAAATGAAATTCCAAAATCGTGTAACAACGTAAAAAGTGTATTTCAAAACCTTGGTACCAAATAGGGTTTTCAGAGACAGAACATGTTTC

>Ca3:21606632-21606861

ATTTTATTCAAAGGAATTAATGTAGCTTTGAAGACTATAGTATACTACAAATTTCATGTGCTATAGGAGAAAAAGTAGTAGTATTAAATATGATTTGGCATTTGTGTTGTTTTGATGGCTTCACAGATGAAAAGTTATATGGCACAATAATAGTATTCCTTCCCATGTGCTGTCATTAGTCTTTGCTGTCAAAACTATGAAGATTTACTTCATGTATTGATTTTGTTGC

>Ca3:22541574-22541959

CTTAAAGAACCAGTAACTGAAAAGCAACCCGGTTTGGCTTAATCTTCACAGTTAACTCTCTCTCTCTCTCTCTCTCTCTCTCTCACACACACACACACACGAAAATGGCTCAACAATAATACAAATTATTGGGATGTTTCTAGAATAATGAATGAAGAGACACCTTGATCTTCATAAAAAACGTGTCTTTCATTCTCCAAAGACACTAAGACAAAGACCCAGAAACACTTTTCTCAAAATTAGAAACACCCAAAAATGGTTTCTTCTTCATTATTGAGAAACAGACAGAAAACACACATGATCTCATTTAATAATAATAATTTCCAAAATATTTATAAAAACAATAAAAATCAGAAGAATCTTCCACCTAGAATGTGATTTGATG

>Ca3:22665515-22666797

TTTCAACATGTCAAAACCAAGCTTTCAAGATATACATCCATTGCACATGCCTCGGGGTATGATGAAGTGCCACATGTCAATGGACAATGGTTAGTGTGTTGATGGACATACTCTTTTTTTTTTTTTTAACGATTTTAAAAGAACACATTTTGTTATATGGCGGATGACTAATTTCGAGTTGAAGAGCTCTCGACCAAGTTTCTCAATTTCAGCATTTAGAATCTCAAAAAATGATATTCCAACCCATCAAGTAATAATCTATTTGAAGACTGTCATAATCAAAATACAAATTTTGTCATATGCCATATAACAAAAAGCGTTCTTTTAAAATAAATCGTTACAAAAACAAAGTAAATGCATCAACACGCAACCCGTTGGCATTACATGTGCCACTTGATCAAAACCCCGAGGCAAATCCAACTGGCTGTATATTAGCATCTCGGGATTCGGATGGCAAACTAATTGAGAATGCCATTCAGAAGTACATCTCCAATACAGTTCAATCAAAAAGGAATCACGTGGAGATATTCACTATTTCTCCCAACTGTCAACTTTCACATCTTCATAATTGAGGTTCACCATAACCATGTAAACAAAGACGCCACCACCTTGAATCATCATAAACAAAGGTGAACTCCAACAGAGAGTTGTCAAAACCGCGAGACATTGCACATCACGATGGAGCTTGTCACCGAGATCTGGTGGATCCAGATCTAATTGAATCAAATTCAGTTACCGATCATAACCTGCTGGCTCCAGATCTGAATCTGGAAGATCCACCGTTTTTTACCGTCATTGCTCCAATTTGATCGAATCAACTCCACCTTCTTCAAATGAAAGCTTGATCTGGTTCCCAGTGGAACCGTGGATCATGATCACTGTTTTCACAGTGGAAAACCGCGATTAATTTTCCGATCTCCTCCACGACAGTACCGCCGCCGCCGTGGAACTGATAAGTTTCTAATAAATTATCGGTTGGATTGAACGGCATCTCCACGAGCTTTTTCTGTGTTTGAGTCTAGAATATATAGGTGAATCTATGACCTACGTAATCGGGTCTAGTACAAATTCTACGGTTGATAACAAAATGGGAAATAGGCGGCCATAAAAGCTTTCTAACAGCCAACTTTTCATTAAATAGAACTCACTCTATCTTGCTATCTTGAAGCTCATTTATTTTTTGCTTCCATGGTTTATCTCTTAAAGAACCTTACTGTATTTTCATGCAACACCAACACATGACATACAATTGGATTTTCATTCCTCACAATTTAACTCAAAC

>Ca3:23462124-23462390

CTGAGTGGAAAAGATCGGATCGGAAAAGTGAACTAGGGTTTGAGAATTTGCCTTGTTTTTGGCCGTTGGAGATATTAGATTGAACGATGGATCCGATTTTTTTTTTTTTTTTTTGAGAAAGATGGATCCGAATTAGTTAATGTGGTATTTCTATTTTTTTCTATATGTATTGTGAAAGAAATGAGAAGAATGTGATAAAGTTGCCAAAGTTGGCCTCACATTTCTGTCCAACTCCTAAACGTGTTTTCGTTTACGAAAAATTAATA

>Ca3:23709575-23710582

CTCACATATTAATTCTTTTTCTAGTAGTATTTAGTTTTGCTAATTACAGTCAATTTAACAAGAATAGATTAAAAAATAAACATTACTCCTAGCTAAGGGTTAGAACTTAGAGGTATATATTTACAAAAGAACAATGATGATCGTTACGAAGTTAAACCAAAACATAAGATAAAAGCACGTTGAAGAACAAACAAAATATTGGGCTTCAAGTTGAAAGAGTAAAATCCTACTTATATAGCATTAAGCACCAGCACACTTATCCTAACCTTGGGCTTCAAGTTGCCGTAATAAGAGCGAAAAGGAAATATATATATATATATAGGTGTGTCGCGAAACCGAATGAAAGATTTATCTTCTTTATTGAATTTCGATGAATCTTTAGTCGCTGAATCGCCGATCCGAATTCGATTCCTCACCATTGCTGCATATTTTCGTCTGAATTCTAGATTCCATGAATTTCTAGTCTCTGTTTCCAAATTCAAGTTTTGCATTCAGGTTTCGATCTTCCGAATTTCAATTTGAATTCTTTATAATTGTTTCGACCGTGGCATCTTCGTATTTGGCGTCAAATTTTTAGGCAACTTTTTTCGGTTTTGATTGCGACGGTGACATCCCGTTCTTGAAAGAACGTTCGATTCCATGAATTTTGATTATTCAAACTCTAAATCTTATACATGAATCTTGATTATTAAAACCCTAGAAATCTGATCCATGAATCTTGATTATGAAAACCCCAGAAATCTTGTTTAAATTGGTGCCGAAAACGATGACCAGTCACAAGCTTTGTTATTCGGATTTTTGATGTATCGATTTTTTCCTTCAGCGCAAACATTTTACGTCCTCAAAACTGTGTTTGGCACTAATGTTTTGGGTAGTTTTTTGTTTGCAACATTTATATTATTATTGATTATTCAAGAATGTATAATCTTGATGATTCTTGAATATTCATGTGAAGGTTGATAAATTGAAAAGTTTCAATTTCTATACTTACGACGGTTGGCCCAATA

>Ca3:23809275-23809479

ATATACTCATAAAGAATAGACATTTGTGGACACAAATGTATGTATCATACAAATATATTTGGTCCATTATCCCCAAGCCATGGTATAACTTAGGGAGAATTCCTCATCCCCAACCCATTGCTTCCATCTGTTCATGACCAATTAGGCTTCAAGAGCACATTGCCTTCTCCACCAGTTCCTGCAATTCCCCATTCTTATATGCCT

>Ca3:24096546-24096789

NNGAATGAGAAAAGCGTGGGGGTTTGGTGGTGGTGGTAGGAGGTGAAGCAGTGACAGTAGCAGAAGTGGAAGCCAGTGAGGATGCCATCAATCAAGTAAACGAAACAACAGAAAAGGAAAGAAGTAAAGTTTGGATGTTGGATCGATTAAATCAAAGGTTTAGTATAGTAAATGTTGTGTTTCATGACACTGGGGCACCTAAACTCCACAAACCTCACACATATCCTATGTATCTAATTCTTC

>Ca3:24450662-24450872

TTAATAAATAACTCTTTAATTTATAAAAATAATGTAAATAAGAAATATATGAAAACATTTGGATTTGTGAATCTAACGTACTTAGTTATATAGAGAAAACGACCTAAAAGTAATGTAACACACGTAATTACTTAGAACCTTTTGCCCTCTCACTGTACAAGTAAATTTGTTCTTCCAGCGACGCATTTCTCTTCTCCATTTGAATCGAAT

>Ca3:24501263-24501532

ACCAAAATAAGATAACCAAGTTTTTTTTTTTACCAAGTATTCAATTGACAAAACATAATGTGTTTTGTGACAAGTTTTAACTACACTTTCATCCCAAAACACATGTCCATCATCCTGGAAGGAACAAATGGCTTAAGCCCCTAAAAAATAACAAGAACCATCAACAGATATTCAGAACTTTACTTTCAAATTTCAAATATTGACATTGTGAACTCCTAAACTAAAACAATAGTCTTTCTGCAATGAAATCTTCAGCAGATTCCCTTTAA

>Ca3:24798515-24798826

CTTGAGAGTGAGACAGCGACCACAGATGAGAGCGCGGGTTCGAGAGGCGGACGGTTCGAGAGGGATTTGATGACGGTGACGACACTGAACAGTGATGGGTTCGAGAGGGGGACGGTGACGAGTCTGAGAAGGGGACGGTGACGAATCTGAGAGGGGGACGGTGACGAGTCTGAGAGGAGGACGATGACGACTCTGATCGTTCGAGAGGGGGACGGCAACGATGAGAAACAGTGAGAGAAACAGTGAGAAAAGGGTTCAAAACATAGGAGAAGGGTTTAGGCACAATCTGATTTTACGATTTACGATCTAAG

>Ca3:25104539-25105077

AGCATGTACATATATCAAACTCTCATCAATGACCCCAAAGGTGGTGATTATGCCTCACAAAGCATTAAGAATAGGGGTGGTCAATATCAAATTACAAGAATAAAAATTAAGTAAACTAAGTCAATAATTACAAATATCTCTTGAAATCCACTCAACTTAAGAAAGGGGTTTAGTTCAACATAATAACAGAAATCATAAAGTAAAGAGACGAGCAAAGAAATTCATGCACAAATGAAGGTAGTCAAGCATCTACTAATCCTCTCTTAGCCATTCATCAAGATCTTCTGTCAAGGTTCTGAAAACACTCCAAAACTGTATTCGAATAGTATTGATAAACTGAATGAAAACATAACTCGTATATGATCTAGGATGCTAGGCCGCCCAACTATGGGCCTCAACATGGCCACCTAACTGTCAACCAACTGTTGGCGGCCTTGCTTGGGAGTGTGGCCGCACAGTGATGCTTAAGACACTTTTTTCAACTTCATTTGGTCCTTTTCTCTTCAATTTTCAAATTACATTTGCGCTCTTTATTCCC

>Ca3:25456769-25458923

TATTCTGATTGTAATTGCAGATATAACATAGTTGAGGTTGCTTACAAACGAAGCGACAAATTATTCACTAAGAAAATCACAGGGCCCAAATTCACTAATCCAAAGTAACTTTTAAATTCAACTAACATTAACTTTTCACTTGGTGGACTTGGTTGAATCTCTAAGGCATGCCAAGTTGGGCCAATCAATGCATGAATATTGAAAAACATATGCTCCATATTGATTCGTGTTTGGGGTAGAGATCATCACTTATTAGCTTTCTTCTTAGTAAGTTTCCTTTTATTGGCAACAGATTATAAGAACGTCTCCATATTGATGTTGCCTGAGACTTCAATTCTTCATATCAATGTTCACAAATTGTGTGATATTGACACAGATATTAGATAGACCTTGATTACTTGATTACTTGATTCCTCTTCTCTCTAGCCTTATGATAGTCAGACTGTGCTATGTAACCTCTTGATTTGGTACAAGTTTATATCAAGTTATCAACTTATCCTTCCCTTATGTATCTCTAACTGGTATTCACATTTCACAATAGCCCATATTCTAGTTTCATTGGGGAAAAACTTCTCTACTTATTGCTATCTCATGCTCTCTGATTTTCCTAAAACAAACTCTCTAACTTCTTGTTGACAAAGTCCAAGAGGCATTGAACCTTCTCCCCATCTAGCAGCTAGTTATCTAATCCAATGGAAATGGTTTTTCCATTCTCCTATTATCTACAACCATGCCTCTTCCAATCTCCTATCGTTGCAATTAGAGGAAGCTCCCCACATTCTCTTTGCCTCCAATAAATCCTCTCATCAACTACTCAACCCAGAAGGCATCAAGACTCTTTGTCCCCTCTCCATGATTGCTTTGTTGAGAAAGTTGTGCTCATATGCACAAAATCTCTAAATCCTGTAGCAGCTGAGTGGAATTTTTGGCCCAAAAACAGTGCTATTATTGACTGAAGCATCAATGAAATATTGACCACTCTTAACTCCACACATCTCCTGGGGAATACCTACACTGACTACAATAACTTATGCAGGATCATAGAAAATGAAAGAAATAAAAACACAATAAACAAAACAAACAAGGGCTTATTAATTAATACTTGCTGTGAATTCATTATACAGCCTGATGTTGTTTAATTAGTTCTTGTCTGTGCTTTTCTTCAATTTACCATGGAGAAATCATGAGACTTGAATGTGAGTAAGAGTTAAAATGAGATCACTGTTATCCCTATGTCATGCACCGGATAATTGAGCACCCAACTGTGTTGTTCTAATTTTAATGGAGCGGTAAAGAACGTTAGAGCTGTAAAAAGAATAAGATCCCTTACCAATTTTCAAAAACAATAAAGCATTATGTTGAAGATATTTTCCATATCAGCTTGTAAATTGAACGCATAATGGGTCAGGAGACTTCCTATCCAAGAATTAACAATAGCACAACAAAACAATATAAGGTAATAAAATGATATAAACATACAATCATGATTCATACCTACCTGCTTTCTCTCTGCAACATGTTCAGTTCTTAAATTACTTTGACAAAATAAACCTTGCCTTGGTTTTGATGGTGAGGAAGAAAAAAAAACTATTCAAAGTTCTGTTAATTCTTGCCTTGGTTCTGACGTCTAGCACTAGTGGCCTGGCACAATATTTGATCCATTATTTTTGTTTATGGCCTGATAGTTTCAAACGGATTCGTAACTTATGGCACATGATGTCTCAAAAAGTAGTTGGATATTGGGATGTATCCAAAAAGTTGAAAGTTGTATGCCACATACCTAGCTATAGCCAAAGTTATGATGCTTCATATTGGCTATGAACTTCCAATCAACAAAGCCAATTTAATTTTGCAAACAATTGCAACTCAAGATTGAAACATAACAAGTGGAATGCATATGTTAAGTGTTCTGTTGTAGAGTGTATTACAAGACCATAGAAAAATCAGACATAATGTGTGATATGTGCATAGCCTGTAATATCCTCAGTTTCATGTTATAAAAGCAGAACAGTTTGGTTTCTGTTTTCTTTTATAGGAACGTAGAAGTCTACTACTCGTGCTTGAATTAATATTTCATACAACACCTACCATGCAACATTATCATTCAAATTAACTGAGTAAAGTAGAAGTGAACATACTATCAATGTAGAATTG

>Ca3:25456769-25457787

TATTCTGATTGTAATTGCAGATATAACATAGTTGAGGTTGCTTACAAACGAAGCGACAAATTATTCACTAAGAAAATCACAGGGCCCAAATTCACTAATCCAAAGTAACTTTTAAATTCAACTAACATTAACTTTTCACTTGGTGGACTTGGTTGAATCTCTAAGGCATGCCAAGTTGGGCCAATCAATGCATGAATATTGAAAAACATATGCTCCATATTGATTCGTGTTTGGGGTAGAGATCATCACTTATTAGCTTTCTTCTTAGTAAGTTTCCTTTTATTGGCAACAGATTATAAGAACGTCTCCATATTGATGTTGCCTGAGACTTCAATTCTTCATATCAATGTTCACAAATTGTGTGATATTGACACAGATATTAGATAGACCTTGATTACTTGATTACTTGATTCCTCTTCTCTCTAGCCTTATGATAGTCAGACTGTGCTATGTAACCTCTTGATTTGGTACAAGTTTATATCAAGTTATCAACTTATCCTTCCCTTATGTATCTCTAACTGGTATTCACATTTCACAATAGCCCATATTCTAGTTTCATTGGGGAAAAACTTCTCTACTTATTGCTATCTCATGCTCTCTGATTTTCCTAAAACAAACTCTCTAACTTCTTGTTGACAAAGTCCAAGAGGCATTGAACCTTCTCCCCATCTAGCAGCTAGTTATCTAATCCAATGGAAATGGTTTTTCCATTCTCCTATTATCTACAACCATGCCTCTTCCAATCTCCTATCGTTGCAATTAGAGGAAGCTCCCCACATTCTCTTTGCCTCCAATAAATCCTCTCATCAACTACTCAACCCAGAAGGCATCAAGACTCTTTGTCCCCTCTCCATGATTGCTTTGTTGAGAAAGTTGTGCTCATATGCACAAAATCTCTAAATCCTGTAGCAGCTGAGTGGAATTTTTGGCCCAAAAACAGTGCTATTATTGACTGAAGCATCAATGAAATATTGACCACTCTTAACTCCACACATCTCCTGGGGAATACCTACACTGA

>Ca3:25456769-25458185
[truncated: 1,400,291 more chars]
